# Supplementary material for: Acaricidal and insecticidal activities of entomopathogenic nematodes combined with rosemary essential oil and bacterium-synthesized silver nanoparticles against camel tick, Hyalomma dromedarii and wax moth, Galleria mellonella
Source: PeerJ. 2025 Jan 14;13:e18782. doi: 10.7717/peerj.18782 (PMC11740739; doi:10.7717/peerj.18782)

Library Search Results - NonTarget Hits with Details

Agilent Technologies

**Batch Path**	D:\MassHunter\GCMS\1\data\2021\February 2021\14.02.2021		
**Analysis File Name**	Unknown analysis.uaf		
**Analyst Name**	owner	**Analysis Time**	2/25/2021 12:21:38 PM
**File Name**	Sample 15.D	**Path Name**	D:\MassHunter\GCMS\1\data\2021\February 2021\14.02.2021
**Sample Name**	Sample 15	**Sample Type**	Sample
**Acq. Method File**	Ahmed taif Slow Ramp SCAN	**Acq. Method Path**	D:\MassHunter\GCMS\1\methods\
**Acq. Date-Time**	2/15/2021 12:13:44 AM	**Acq. Operator**	
**Instrument Name**	GCMS	**Dil.**	1

Component RT	Compound Name	CAS#	Formula	Component Area	Match Factor	Estimated Conc.
6.0792	Bicyclo[3.1.1]hept-3-en-2-one, 4,6,6-trimethyl-, (1S)-	1196-01-6	C10H14O	5803973675.9	91.4	
6.3234	2-Heptene, 5-ethyl-2,4-dimethyl-	74421-06-0	C11H22	234194769.0	66.8	
6.9946	Geranyl formate	105-86-2	C11H18O2	720266743.8	76.1	
7.0317	6-Methyl-2-(piperidin-1-ylmethyl)-4-pyrimidinylamine	112860-60-3	C11H18N4	50857691.7	76.6	
7.7313	1H-Pyrazole-4-carboxylic acid, 3-amino-	41680-34-6	C4H5N3O2	16402844.8	69.1	
7.8561	1,7-Octadiene-3,6-diol, 2,6-dimethyl-	51276-33-6	C10H18O2	403789701.6	83.5	
7.9053	Formamide, N-methyl-	123-39-7	C2H5NO	5003176.0	72.6	
8.0794	Cyanogen bromide	506-68-3	CBrN	18653312.1	67.5	
8.3380	Benzene, 1,2,3-trimethyl-	526-73-8	C9H12	9722788.4	65.3	
8.3502	Thymol	89-83-8	C10H14O	96926703.6	82.0	
9.2609	Benzaldehyde, 4-benzyloxy-3-methoxy-2-nitro-	2450-27-3	C15H13NO5	9540564.0	71.9	
9.5695	Benzenamine, 4-ethoxy-	156-43-4	C8H11NO	5218086.8	69.4	
9.6134	2-Methylbicyclo[4.3.0]non-1(6)-ene	60223-07-6	C10H16	37456296.8	80.0	
9.9797	Eugenol	97-53-0	C10H12O2	77500138.1	84.8	
10.0968	p-Mentha-1(7),2-dien-8-ol	65293-09-6	C10H16O	28571758.4	82.0	
10.4002	2,6-Octadienoic acid, 3,7-dimethyl-, (E)-	4698-08-2	C10H16O2	70257881.8	80.8	
10.5575	Furan, 2-(1-pentenyl)-, (E)-	20992-69-2	C9H12O	7757846.8	65.9	
10.6136	Bicyclo[2.2.1]heptane, 2-chloro-2,3,3-trimethyl-	465-30-5	C10H17Cl	94067837.7	73.8	
10.9035	(3E,5E)-2,6-Dimethylocta-3,5,7-trien-2-ol	206115-88-0	C10H16O	495867967.9	84.8	
11.6076	Methyleugenol	93-15-2	C11H14O2	435789812.2	92.2	
11.8291	Succinimide	123-56-8	C4H5NO2	10467697.7	69.3	
11.8375	Cyclopropanecarboxamide, N-tetrahydrofurfuryl-	1000307-18-4	C9H15NO2	38050668.1	68.2	
12.0535	Heptyl methyl ethylphosphonate	169662-35-5	C10H23O3P	18077542.4	68.0	
12.2308	Imidazo[4,5-d]imidazole, 1,6-dihydro-	35369-36-9	C4H4N4	23364294.9	72.4	
12.2405	3,7-Nonadien-2-one, 8-methyl-, (E)-	35408-14-1	C10H16O	40152413.4	71.1	
12.2464	2-Pyrrolidinone, 5-(ethoxymethyl)-	64749-67-3	C7H13NO2	13450746.9	65.9	
12.3542	Ethane, 1,2-dibromo-	106-93-4	C2H4Br2	8408546.6	65.1	
12.3636	Formamide, N-methyl-	123-39-7	C2H5NO	9211691.7	66.1	
12.3685	8-Hydroxycarvotanacetone	7712-46-1	C10H16O2	36992552.9	73.8	
12.7619	2,6-Octadiene-1,8-diol, 2,6-dimethyl-	26489-17-8	C10H18O2	585937479.7	85.3	
13.4756	Acetophenone, 4'-hydroxy-	99-93-4	C8H8O2	203883884.4	92.3	
13.5267	Glycine, methyl ester	616-34-2	C3H7NO2	8616387.8	67.7	
13.8200	1-Decanol	112-30-1	C10H22O	27360201.0	80.9	
14.2688	Ethanone, 1-(2-hydroxy-4-methoxyphenyl)-	552-41-0	C9H10O3	69695815.7	82.3	
14.3578	Hydroxychavicol	1126-61-0	C9H10O2	234244947.6	86.6	
14.4513	(1S,2S,4S)-Trihydroxy-p-menthane	22555-61-9	C10H20O3	157460019.0	85.6	
15.0231	2-Caren-4-ol	6617-35-2	C10H16O	82688352.5	77.0	
15.8057	2(4H)-Benzofuranone, 5,6,7,7a-tetrahydro-4,4,7a-trimethyl-, (R)-	17092-92-1	C11H16O2	28315348.1	74.8	
15.8725	2,4-Di-tert-butylphenol	96-76-4	C14H22O	45393295.5	83.6	
16.7055	2(3H)-Furanone, dihydro-5,5-dimethyl-4-(3-oxobutyl)-	4436-81-1	C10H16O3	87657582.3	81.0	
16.9017	6-Hydroxy-3,7-dimethyl-2,7-octadienyl acetate(E)	33766-43-7	C12H20O3	58854598.0	87.8	
17.0768	Phenol, 4-pentyl-	14938-35-3	C11H16O	42825874.6	80.0	
17.4597	Caryophyllene oxide	1139-30-6	C15H24O	500658899.7	89.0	
18.4895	(1R,3E,7E,11R)-1,5,5,8-Tetramethyl-12-oxabicyclo[9.1.0]dodeca-3,7-diene	19888-34-7	C15H24O	115334471.5	78.6	
19.3722	11,11-Dimethyl-4,8-dimethylenebicyclo[7.2.0]undecan-3-ol	79580-01-1	C15H24O	341386339.2	92.8	
19.7293	2-Butanone, 4-(4-hydroxy-3-methoxyphenyl)-	122-48-5	C11H14O3	251489371.8	93.9	
19.8476	Methyl jasmonate	1211-29-6	C13H20O3	59918382.7	82.0	
19.9903	Caryophyllene oxide	1139-30-6	C15H24O	345099318.1	85.9	
20.4148	Caryophyllene oxide	1139-30-6	C15H24O	247469818.7	87.0	
20.7426	L-Leucine, N-methyl-N-(but-3-yn-1-yloxycarbonyl)-, hexadecyl ester	1000392-38-3	C28H51NO4	1461943.0	67.9	
22.2324	(3E,10Z)-Oxacyclotrideca-3,10-diene-2,7-dione	144403-15-6	C12H16O3	17105796.8	70.8	
22.3419	2(1H)-Pyridinone, 3-hydroxy-	16867-04-2	C5H5NO2	4779062.5	66.2	
22.3545	1,3-Cyclohexanediol, 5-methyl-2-nitro-, monoacetate (ester), [1s-(1.alpha.,2.beta.,3.alpha.,5.alpha.)]-	114454-85-2	C9H15NO5	14946021.0	68.8	
22.9996	Drim-7-en-11-ol	468-68-8	C15H26O	76372837.2	71.3	

Component RT	Compound Name	CAS#	Formula	Component Area	Match Factor	Estimated Conc.
23.2402	Benzyl Benzoate	120-51-4	C14H12O2	69586326.0	93.9	
23.9727	6-Hydroxy-4,4,7a-trimethyl-5,6,7,7a-tetrahydrobenzofuran-2(4H)-one	73410-02-3	C11H16O3	93889305.7	87.7	
24.1506	2-Ethylhexanal ethylene glycol acetal	1000431-01-2	C10H20O2	52729202.0	74.7	
24.9110	Caryophyllene oxide	1139-30-6	C15H24O	94785407.6	77.3	
25.1584	Caryophyllene oxide	1139-30-6	C15H24O	120035671.3	82.1	
25.7494	Neophytadiene	504-96-1	C20H38	19882967.8	75.4	
25.9197	2-Pentadecanone, 6,10,14-trimethyl-	502-69-2	C18H36O	25388415.5	82.7	
25.9254	2-Heptanone, 6-methyl-	928-68-7	C8H16O	18963734.4	65.8	
26.3348	Benzoic acid, 2-hydroxy-, phenylmethyl ester	118-58-1	C14H12O3	72961635.6	88.1	
26.8667	(3S,3aS,6R,7R,9aS)-1,1,7-Trimethyldecahydro-3a,7-methanocyclopenta[8]annulene-3,6-diol	2649-64-1	C15H26O2	150284933.2	88.9	
27.0619	Ethaneperoxoic acid, 1-cyano-1-[2-(2-phenyl-1,3-dioxolan-2-yl)ethyl]pentyl ester	58422-92-7	C19H25NO5	5590160.0	77.8	
27.7857	Phenanthrene, 7-ethenyl-1,2,3,4,4a,4b,5,6,7,9,10,10a-dodecahydro-1,1,4a,7-tetramethyl-, [4aS-(4a.alpha.,4b.beta.,7.beta.,10a.beta.)]-	1686-56-2	C20H32	23458675.7	70.3	
28.9257	4-(6-Methoxy-3-methyl-2-benzofuranyl)-3-buten-2-one	10444-37-8	C14H14O3	46521483.9	80.0	
29.0920	Oxo-(6-phenyl-imidazo[1,2-a]pyridin-2-yl)-acetic acid, ethyl ester	1000318-40-7	C17H14N2O3	3993381.6	70.4	
29.2854	6-(n-Butyl)oxy-4-methylcoumarin	1000395-87-3	C14H16O3	58238616.2	70.1	
29.4912	4-Methyl-2,4-bis(p-hydroxyphenyl)pent-1-ene, 2TMS derivative	1000283-56-8	C24H36O2Si2	3763798.4	68.3	
29.6487	Phthalic acid, butyl cyclobutyl ester	1000314-89-9	C16H20O4	12710075.9	82.7	
30.2811	Kaur-15-ene	5947-50-2	C20H32	33051872.3	77.9	
30.8526	Cannabidivarol	24274-48-4	C19H26O2	51391774.3	65.4	
31.4334	Verimol K	85985-75-7	C14H12O4	12745130.2	74.4	
31.7823	7-Isopropyl-1,1,4a-trimethyl-1,2,3,4,4a,9,10,10a-octahydrophenanthrene	109680-01-5	C20H30	56661634.8	94.7	
32.6676	(3R,6R)-3-Hydroperoxy-3-methyl-6-(prop-1-en-2-yl)cyclohex-1-ene	77026-88-1	C10H16O2	10718472.6	68.4	
33.2000	9,12,15-Octadecatrienoic acid, methyl ester, (Z,Z,Z)-	301-00-8	C19H32O2	21305253.3	74.8	
35.4762	4,6-Dimethoxy-1-naphthaldehyde	65565-33-5	C13H12O3	96326222.1	70.0	
36.2537	Kaur-16-en-18-ol, (4.alpha.)-	2300-11-0	C20H32O	102194459.1	85.5	
37.4176	4-Hydroxy-4'-methyldiphenylamine, O-trimethylsilyl ether	1000417-59-7	C16H21NOSi	215856697.7	67.5	
38.0240	3-Trifluoromethylbenzylamine, N,N-diundecyl	1000310-29-9	C30H52F3N	13229292.3	71.2	
38.1122	1,4-Dimethyl-8-isopropylidenetricyclo[5.3.0.0(4,10)]decane	1000140-07-7	C15H24	75912392.9	78.6	
38.2360	Acetic acid, 2-(1,5-ditert-butyl-3-phenyl-3-piperidyl) ester	328282-96-8	C21H33NO2	8643996.4	66.1	
38.4444	1,2-Bis(diethylamino)-1,2-bis(4-cyanophenyl)ethane	1000193-30-0	C24H30N4	1331846.3	66.3	
38.7934	1,3,6,10-Cyclotetradecatetraene, 3,7,11-trimethyl-14-(1-methylethyl)-, [S-(E,Z,E,E)]-	1898-13-1	C20H32	68877614.6	77.3	
39.1906	Ferruginol	514-62-5	C20H30O	784231253.5	92.6	
39.5511	4-Acetylphenyl 5-acetyl-2-methoxyphenyl ether	7251-24-3	C17H16O4	50766154.7	65.8	
39.6357	Trifluoroacetic acid, 2-methylpropyl ester	17355-83-8	C6H9F3O2	12150521.2	75.4	
39.8748	4,5,6,7-Tetrahydroxy-1,8,8,9-tetramethyl-8,9-dihydrophenaleno[1,2-b]furan-3-one	2582-86-7	C19H18O6	159889981.2	65.4	
40.0515	4-Phenoxy-2-phenyl-1-naphthalenol	253801-45-5	C22H16O2	628144.6	66.9	
40.1437	6,8-Dimethyl-5-oxo-2,3,5,8-tetrahydroimidazo[1,2-a]pyrimidine	26955-15-7	C8H11N3O	21959557.6	66.0	
40.2330	12-O-Methylcarnosol	85514-27-8	C21H28O4	241176231.5	77.9	
40.3933	(tert-Butyl)(4,6-diimidazol-1-yl-[1,3,5]triazin-2-yl)amine	1000304-62-9	C13H16N8	10323728.5	69.8	
40.7060	.delta.9-Tetrahydrocannabivarin	31262-37-0	C19H26O2	1045443725.7	80.7	
40.8016	Pyrimidine, 4-(2-hydroxy-5-methoxyphenyl)-	97630-77-8	C11H10N2O2	20394122.2	69.5	
41.2933	2(1H)-Phenanthrenone, 4a,9,10,10a-tetrahydro-6-hydroxy-1,1,4a-trimethyl-7-(1-methylethyl)-, (4aS-trans)-	18326-19-7	C20H26O2	2429782318.8	78.4	
42.1646	4'-Amino-6-methoxyaurone	77764-96-6	C16H13NO3	27771080.6	81.1	
42.4540	2-(4'-Methoxyphenyl)-2-(3'-methyl-4'methoxyphenyl)propane	1000283-53-6	C18H22O2	3185706.5	66.0	
43.4056	6H-[1,3]Dioxolo[5,6]benzofuro[3,2-c][1]benzopyran-2-ol, 6a,12a-dihydro-3-methoxy-, (6ar-cis)-	30461-92-8	C17H14O6	27691440.2	67.8	
44.1592	Benzo[1,2-b:5,4-b']difuran-4,8-dione, 5-methyl-2-(1-methylethenyl)-	26962-40-3	C14H10O4	3015723.7	72.2	
44.1829	13-Isopropylpodocarpen-12-ol-20-al	24035-37-8	C20H28O2	550679394.3	90.3	
44.6501	Phthalic acid, di(2-propylpentyl) ester	1000377-93-5	C24H38O4	47610761.1	76.9	
44.8575	2-Hydroxy-3-methylanthraquinone, O-trimethylsilyl	91701-15-4	C18H18O3Si	8921824.7	65.8	
45.1100	2-(2',4'-Dimethoxyphenyl)-6-methoxy-benzofuran	67685-23-8	C17H16O4	2501061634.3	79.0	

Component RT	Compound Name	CAS#	Formula	Component Area	Match Factor	Estimated Conc.
45.5944	2(1H)-Phenanthrenone, 3,4,4a,9,10,10a-hexahydro-6-hydroxy-1,1,4a-trimethyl-7-(1-methylethyl)-, (4aS-trans)-	472-37-7	C20H28O2	123725426.8	70.2	
45.7139	(.+/-.)-Demethylsalvicanol	177019-45-3	C20H30O3	1248028356.4	96.1	
45.9197	Propenoic acid, 2-cyano-3-(1-methyl-5-phenylthio-2-pyrrolyl)-, methyl ester	94008-41-0	C16H14N2O2S	41650451.7	73.0	
45.9872	5,6,4'-Trihydroxy-7,8-dimethoxyflavone	76844-66-1	C17H14O7	1936142.4	70.2	
47.4100	Chroman-4-one, 2,3-dehydro-7-hydroxy-2-methyl-3-(2-pyridyl)-	65047-28-1	C15H11NO3	5350379.7	65.5	
48.0260	2,4-Difluorobenzoic acid, 2-biphenyl ester	1000331-58-3	C19H12F2O2	8473089.1	73.4	
48.0841	Silane, dimethyl(2-naphthoxy)dodecyloxy-	1000347-21-9	C24H38O2Si	74691550.2	73.8	
48.1373	tert-Butyldimethylsilanol	18173-64-3	C6H16OSi	49745965.6	69.5	
48.7676	Androst-5-en-7-one, 3-(acetyloxy)-, (3.beta.)-	25845-92-5	C21H30O3	132522809.7	69.8	
49.8853	12-O-Methylcarnosol	85514-27-8	C21H28O4	80971832.8	85.0	
50.1294	1,4-Bis-(4-fluorophenylethynyl)-2,3,5,6-tetrafluorobenzene	332148-91-1	C22H8F6	1967954.4	68.0	
50.8649	.alpha.-Tocospiro A	601490-40-8	C29H50O4	124175709.1	78.7	
51.2936	.alpha.-Tocospiro B	601490-41-9	C29H50O4	136877861.0	90.3	
52.0430	Oxazole, 2-[1,1'-biphenyl]-4-yl-5-phenyl-	852-37-9	C21H15NO	1242429.9	72.1	
54.8045	Isophthalic acid, di(2-methylprop-2-en-1-yl) ester	1000343-95-8	C16H18O4	4592828.9	73.8	
56.1138	dl-.alpha.-Tocopherol	10191-41-0	C29H50O2	145133668.8	93.8	
57.0375	4H-1-Benzopyran-4-one, 5-hydroxy-6,7-dimethoxy-2-(4-methoxyphenyl)-	19103-54-9	C18H16O6	112768464.1	86.3	
59.3927	.gamma.-Sitosterol	83-47-6	C29H50O	36034478.5	72.5	
61.0418	Lupeol	545-47-1	C30H50O	39047163.6	68.8	

Agilent Technologies

Sample Chromatogram

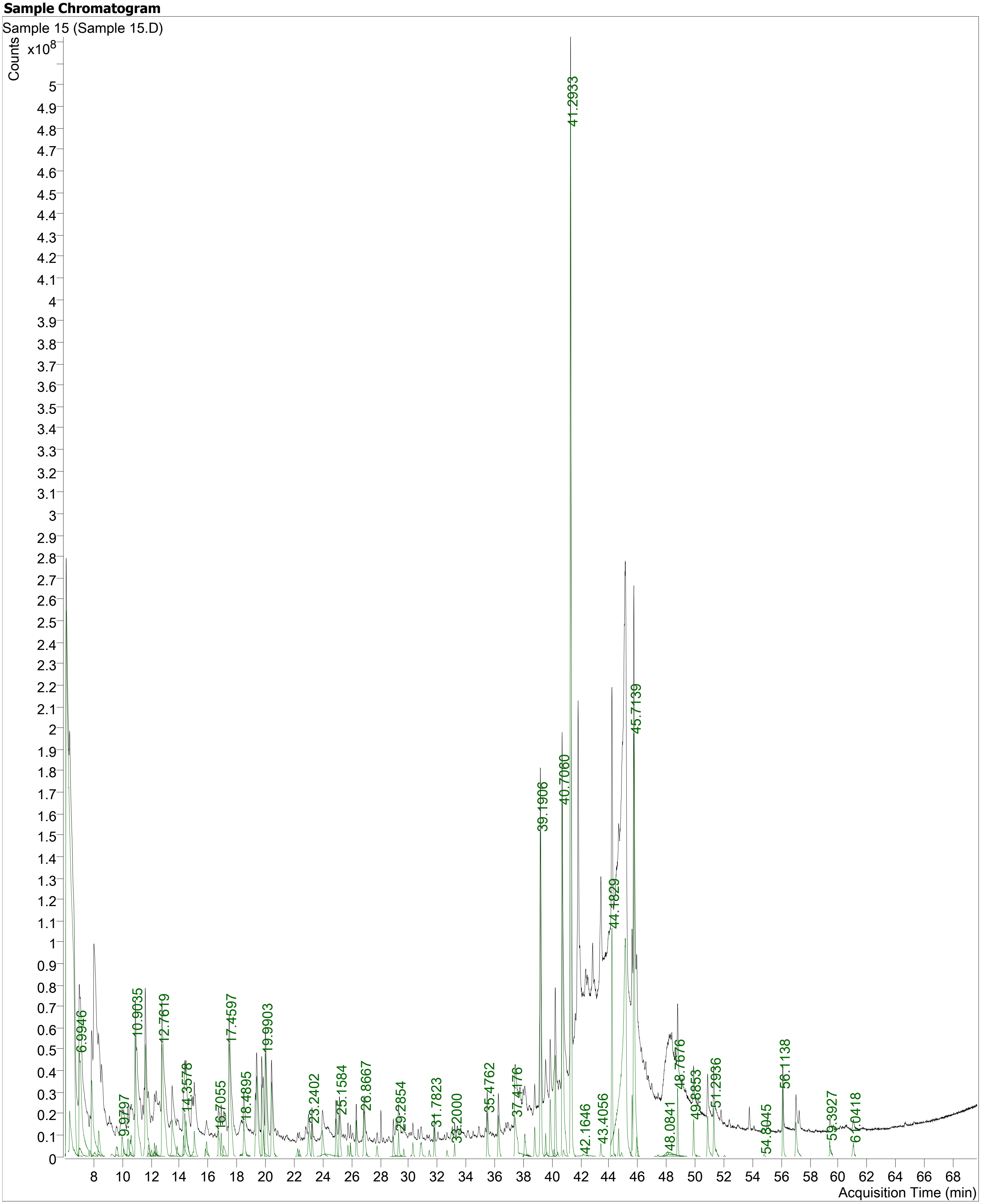

 Generated at 12:24 PM on 2/25/2021

Component RT	Compound Name	Component Area	Match Factor	CAS#	Formula	Estimated Conc.
6.0792	Bicyclo[3.1.1]hept-3-en-2-one, 4,6,6-trimethyl-, (1S)-	5803973675.9	91.4	1196-01-6	C10H14O	

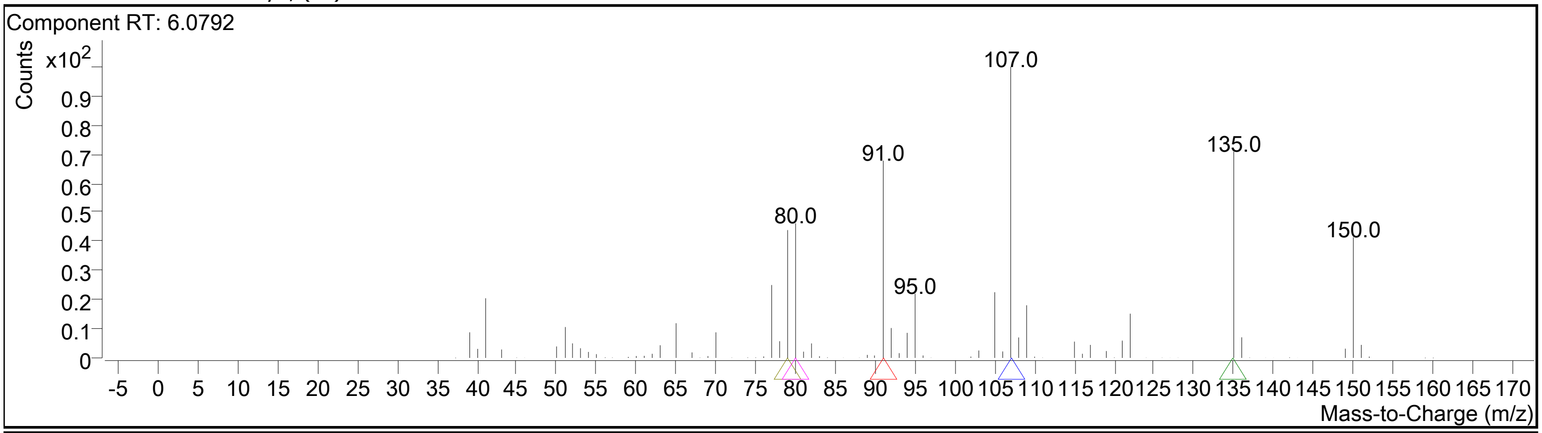

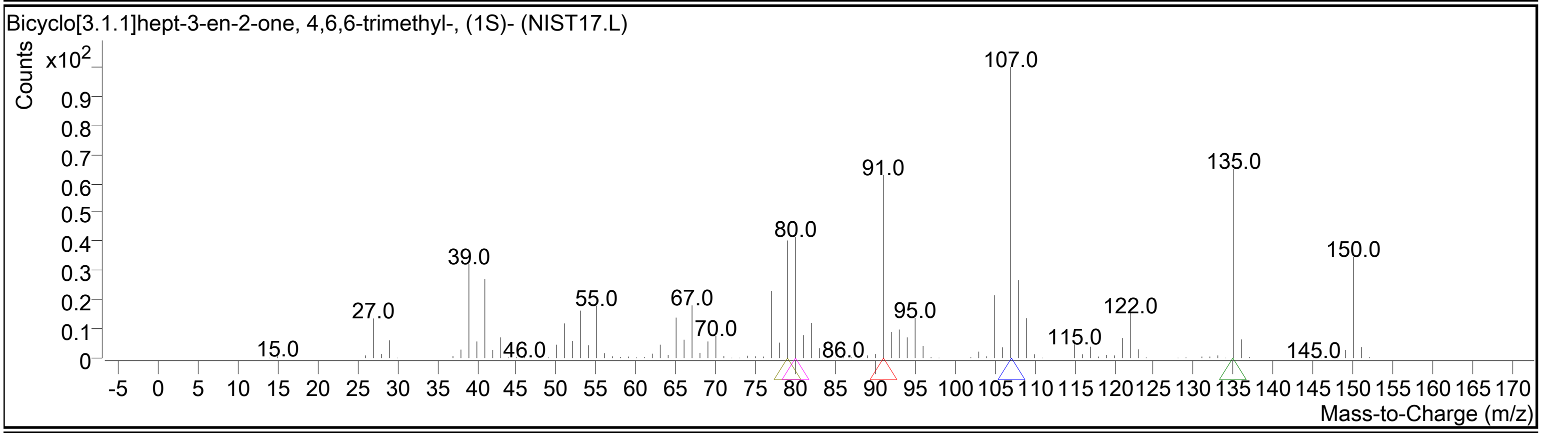

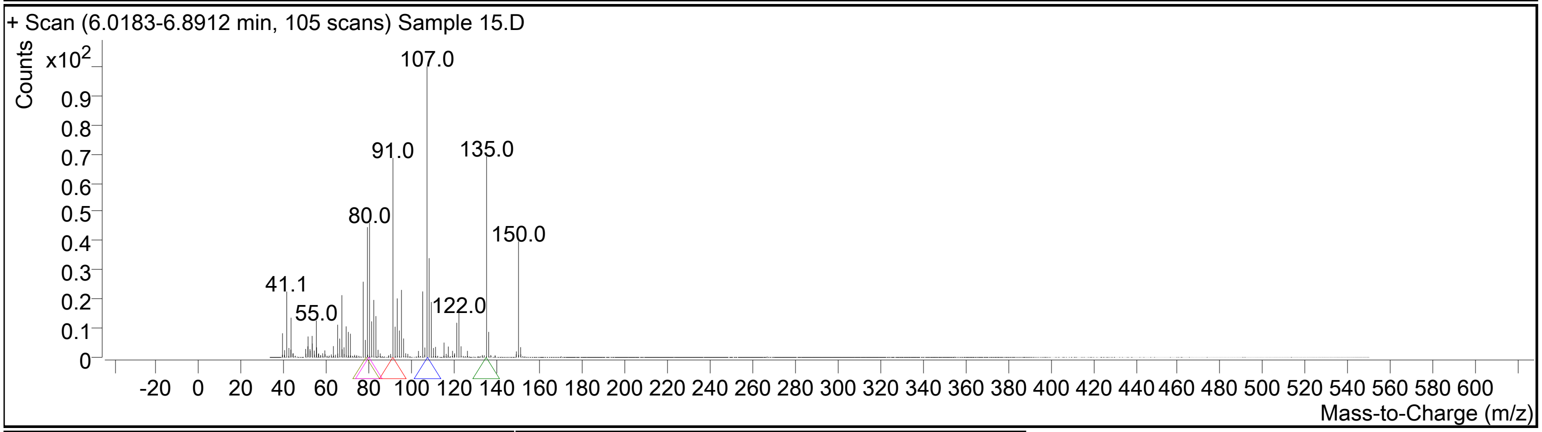

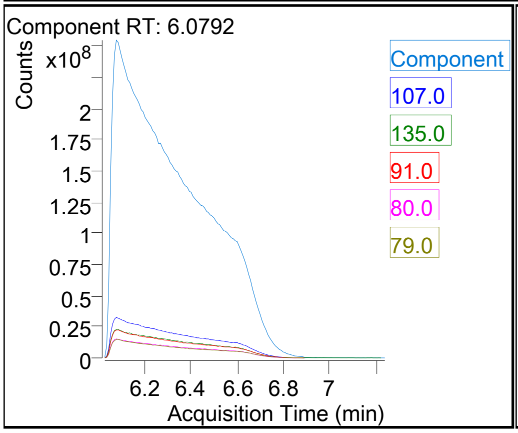

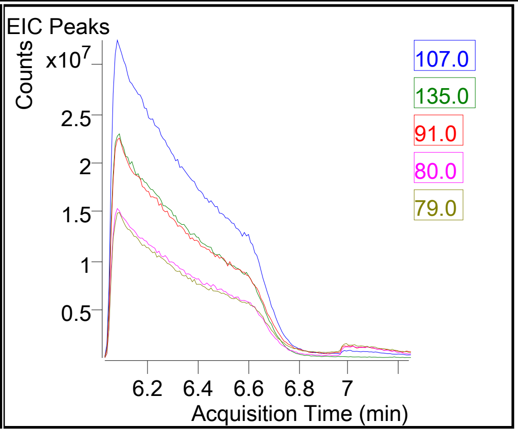

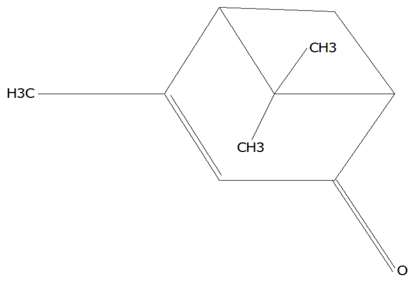

Component RT	Compound Name	Component Area	Match Factor	CAS#	Formula	Estimated Conc.
6.3234	2-Heptene, 5-ethyl-2,4-dimethyl-	234194769.0	66.8	74421-06-0	C11H22	

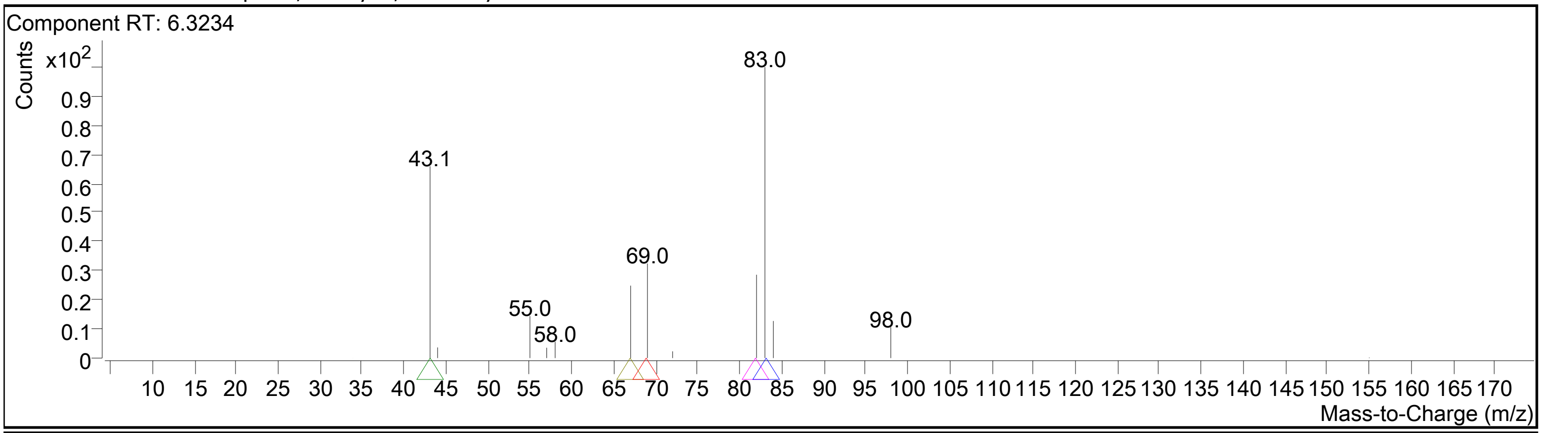

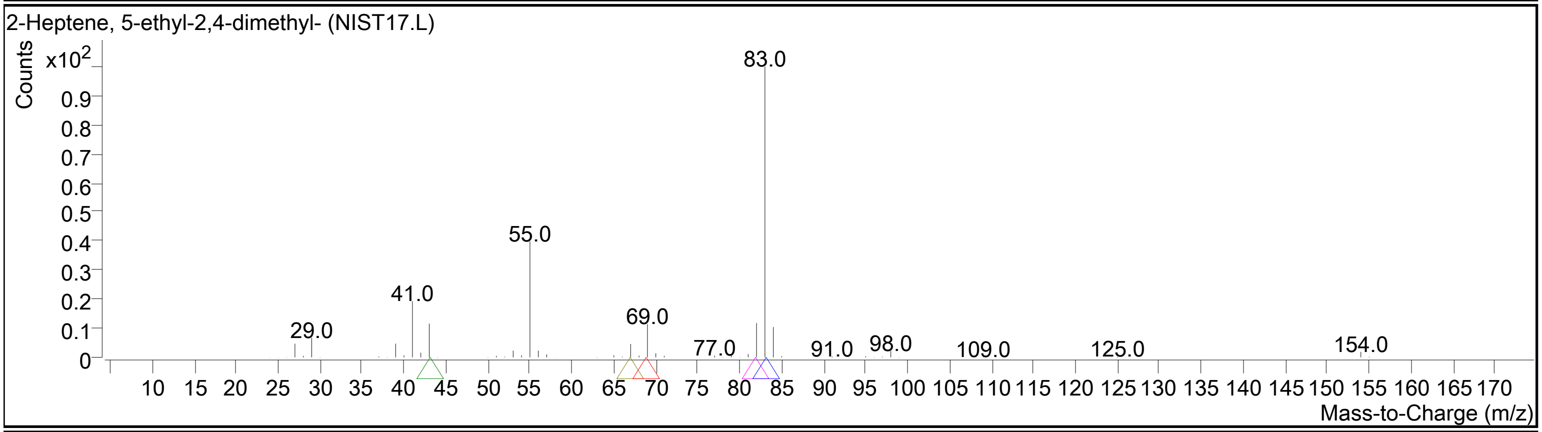

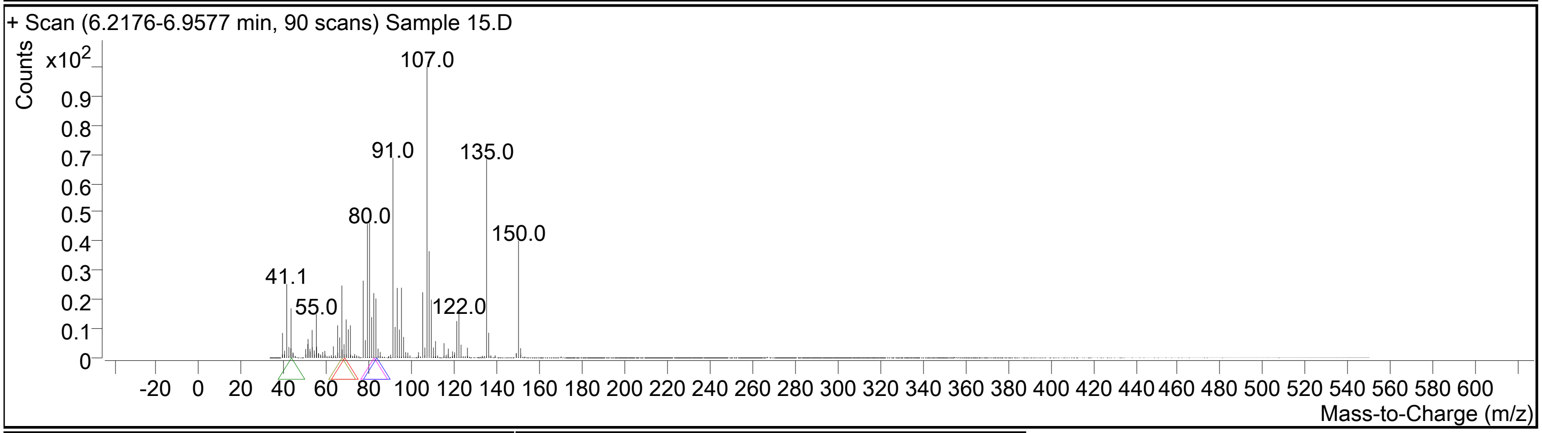

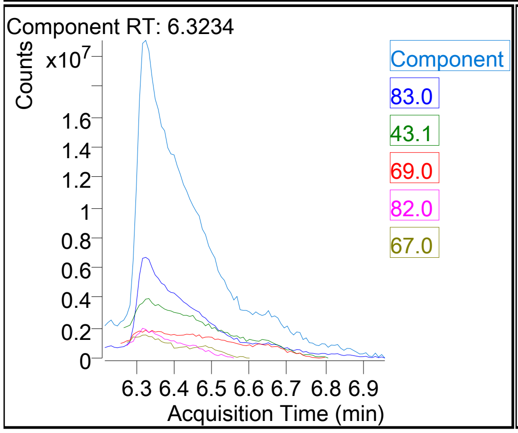

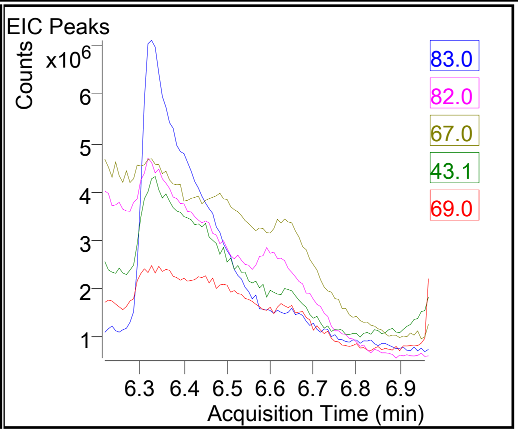

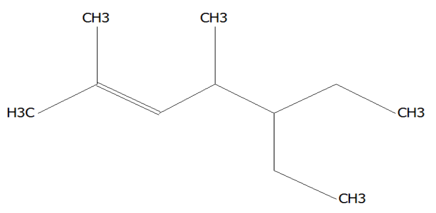

Generated at 12:24 PM on 2/25/2021

Agilent Technologies

Component RT	Compound Name	Component Area	Match Factor	CAS#	Formula	Estimated Conc.
6.9946	Geranyl formate	720266743.8	76.1	105-86-2	$C_{11}H_{18}O_2$	

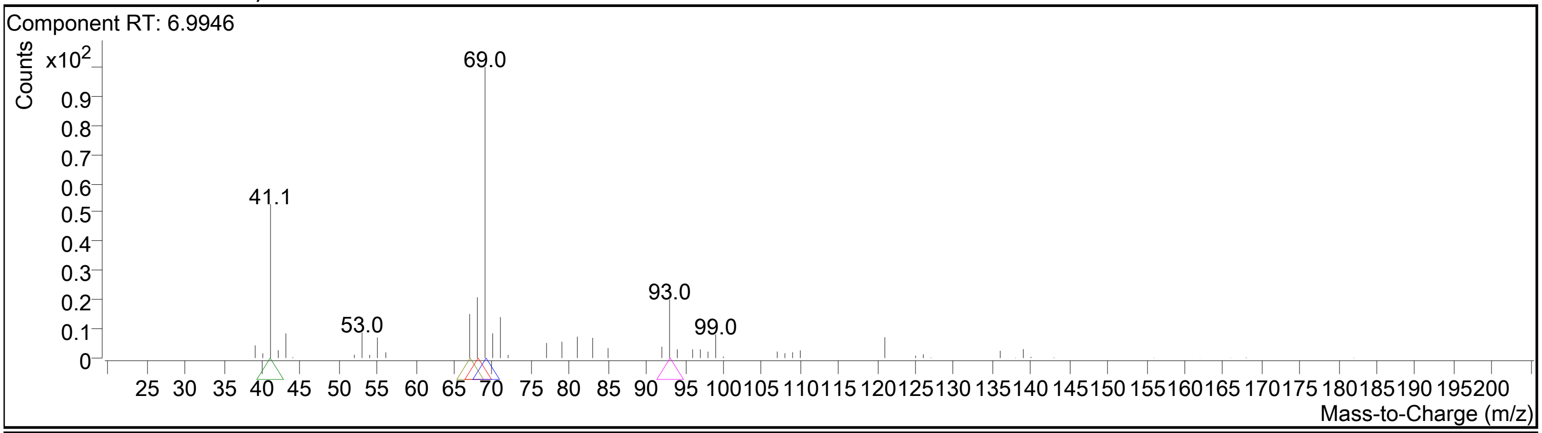

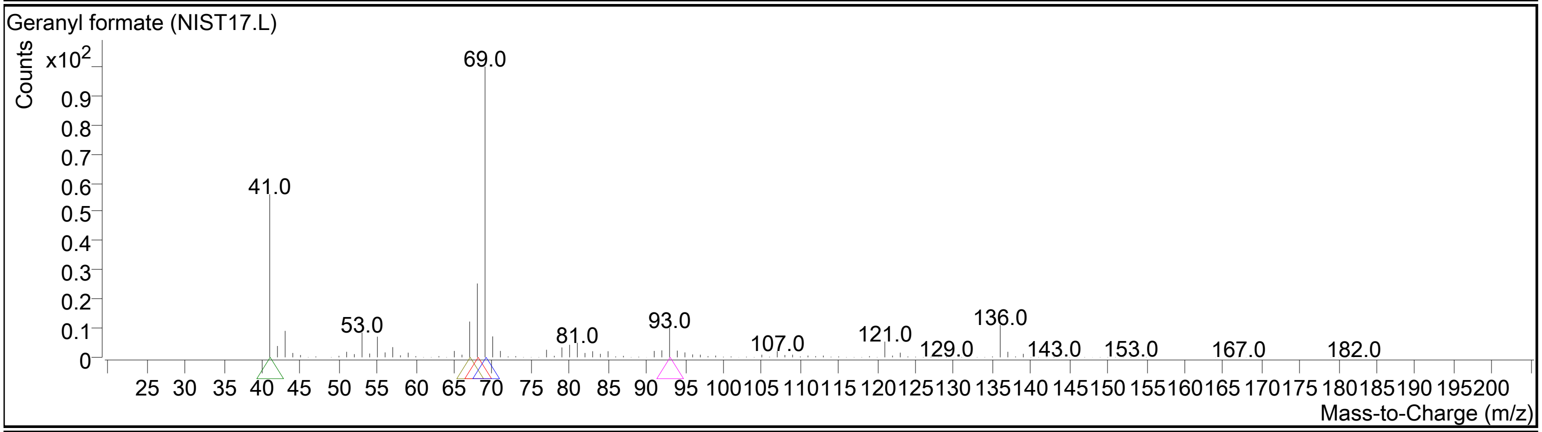

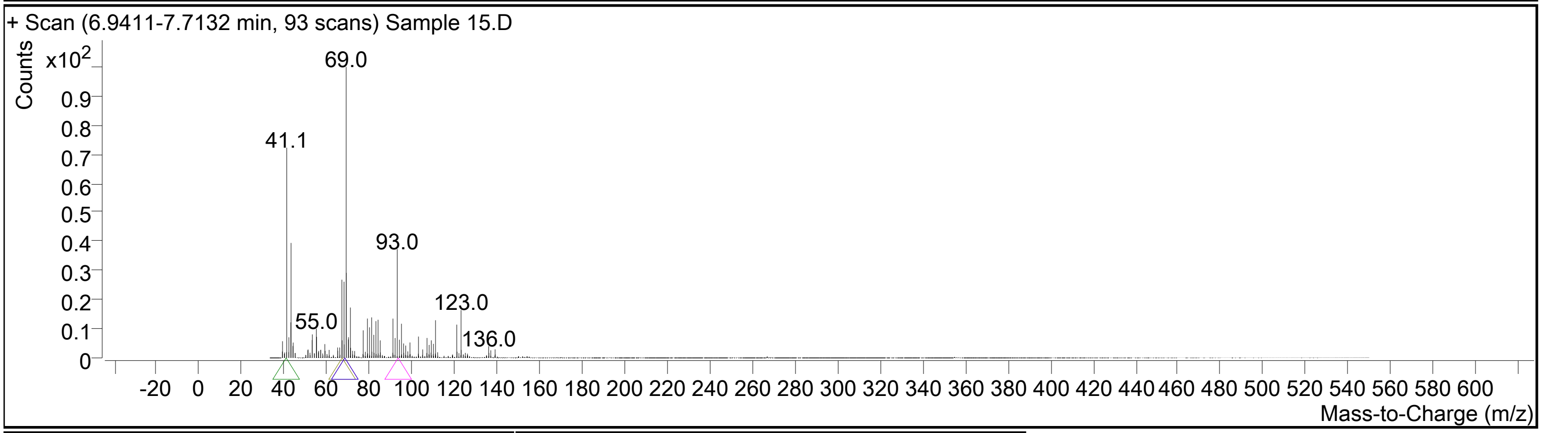

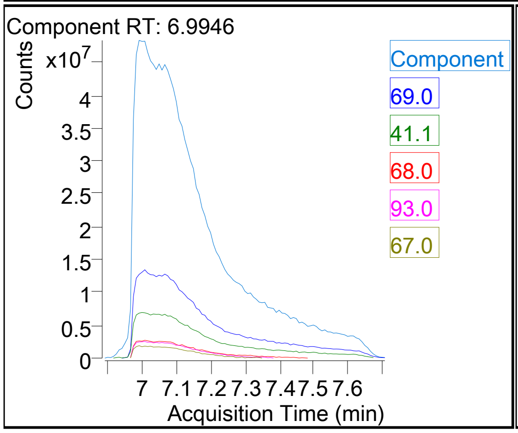

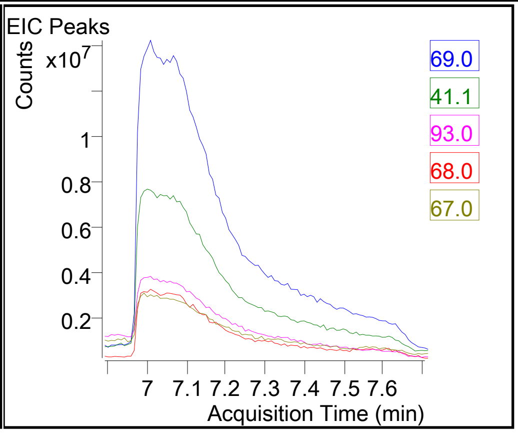

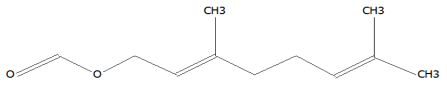

Generated at 12:24 PM on 2/25/2021

Component RT	Compound Name	Component Area	Match Factor	CAS#	Formula	Estimated Conc.
7.0317	6-Methyl-2-(piperidin-1-ylmethyl)-4-pyrimidinylamine	50857691.7	76.6	112860-60-3	C11H18N4	

Component RT: 7.0317

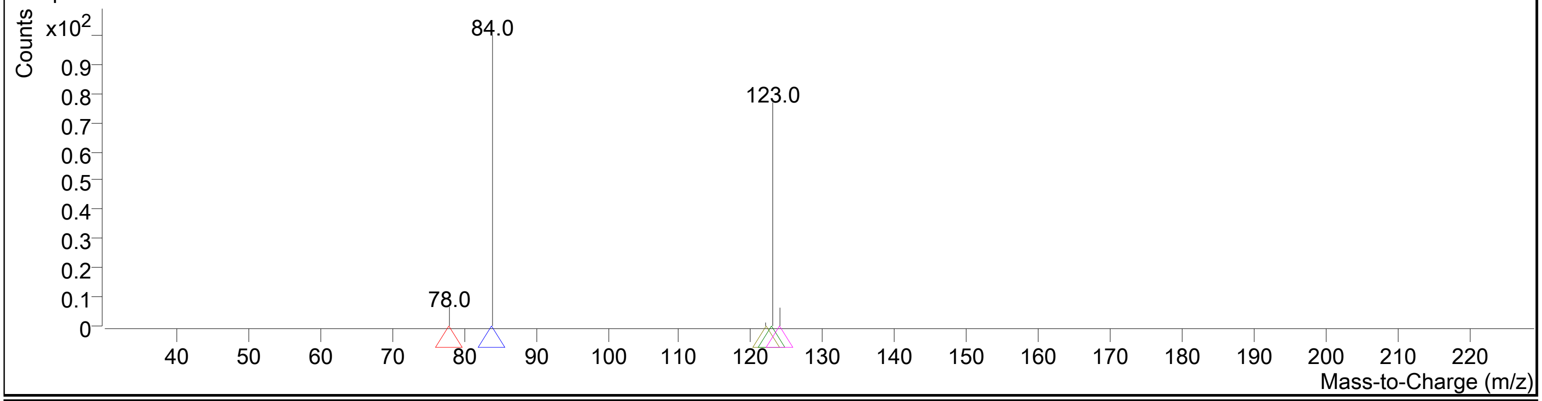

6-Methyl-2-(piperidin-1-ylmethyl)-4-pyrimidinylamine (NIST17.L)

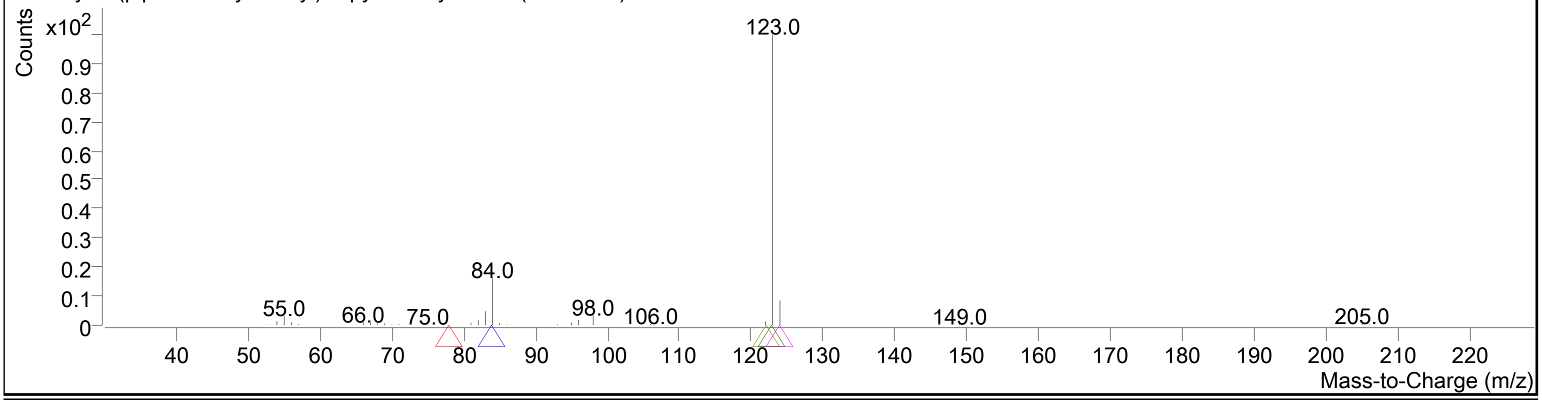

+ Scan (6.9327-7.3818 min, 55 scans) Sample 15.D

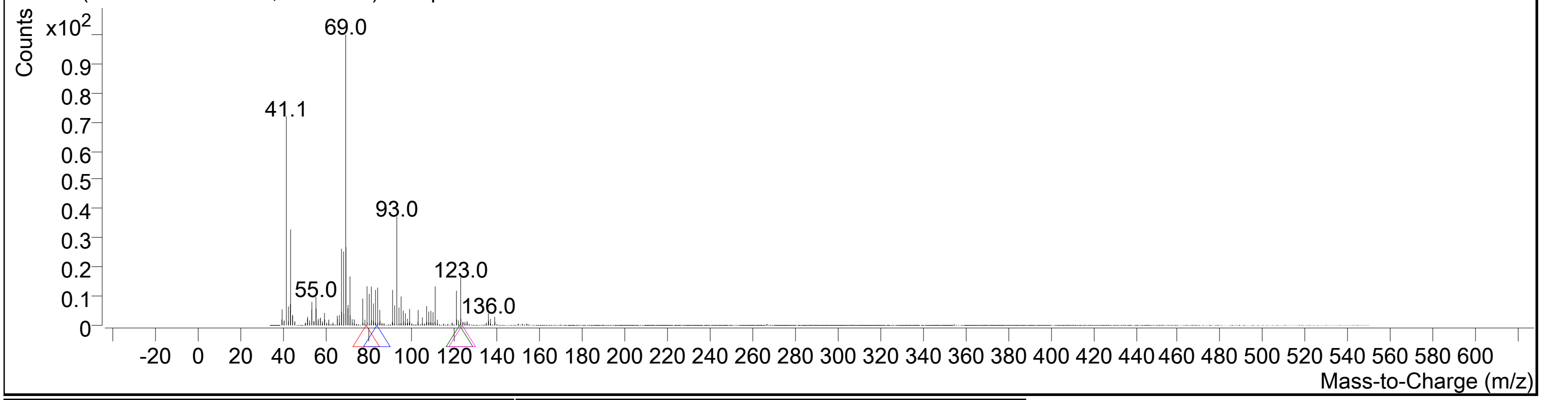

Component RT: 7.0317

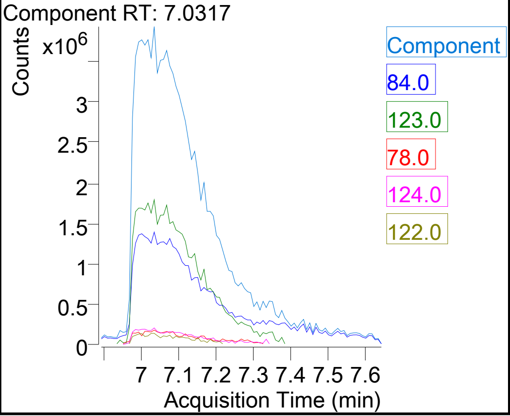

EIC Peaks

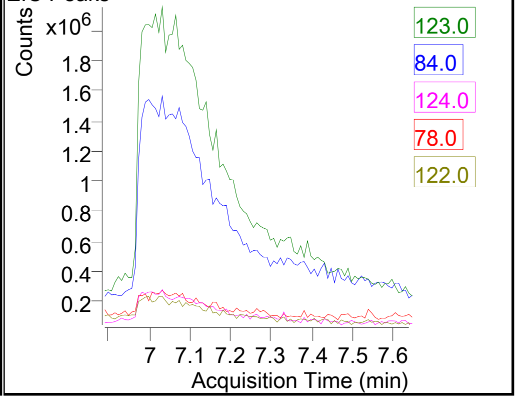

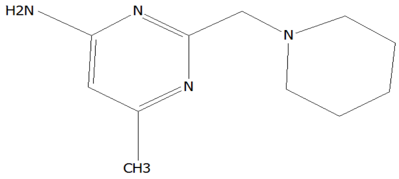

Component RT	Compound Name	Component Area	Match Factor	CAS#	Formula	Estimated Conc.
7.7313	1H-Pyrazole-4-carboxylic acid, 3-amino-	16402844.8	69.1	41680-34-6	C4H5N3O2	

Component RT: 7.7313

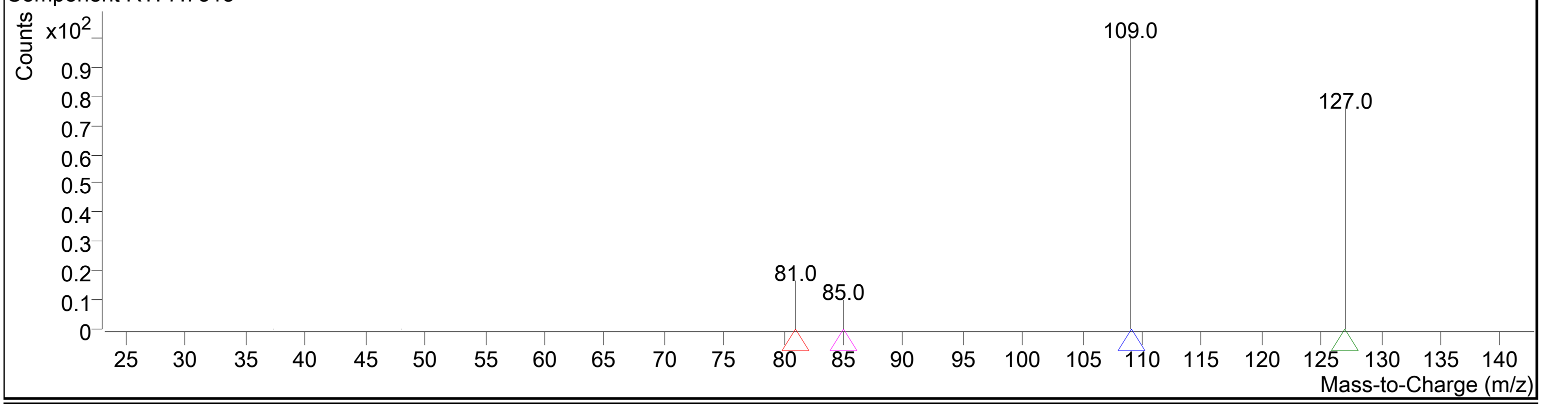

1H-Pyrazole-4-carboxylic acid, 3-amino- (NIST17.L)

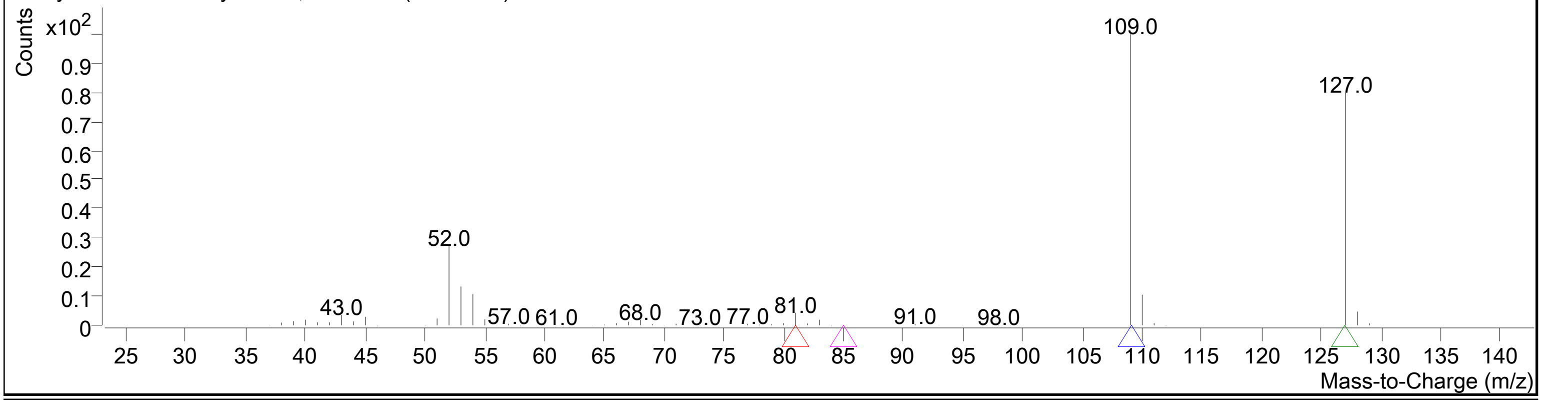

+ Scan (7.6921-7.8918 min, 25 scans) Sample 15.D

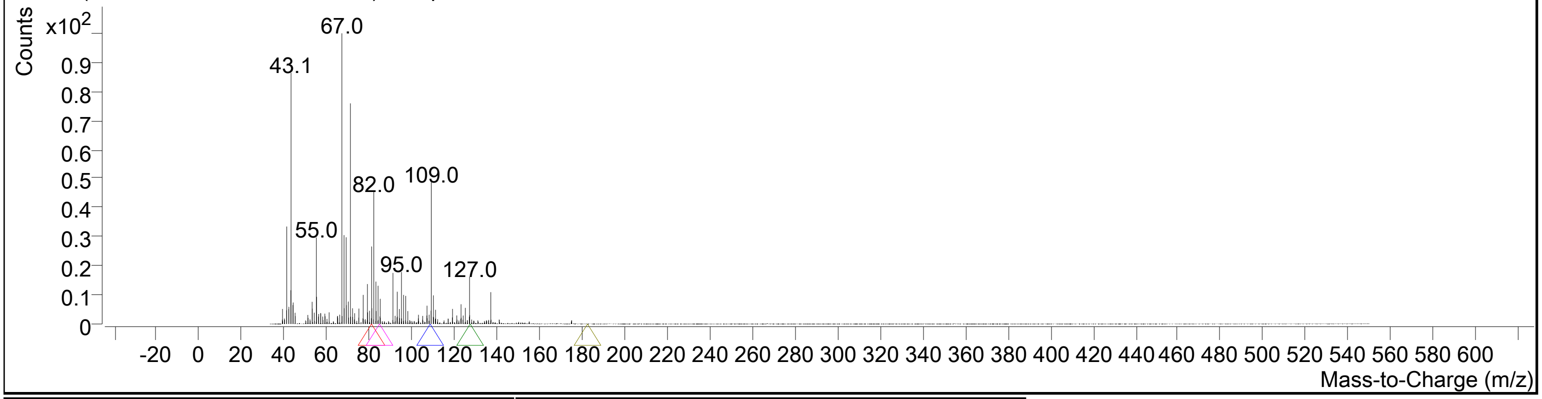

Component RT: 7.7313

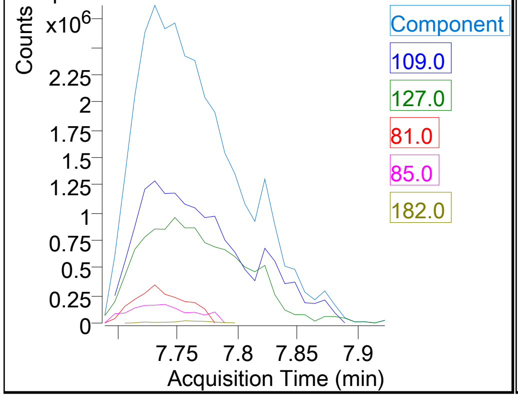

EIC Peaks

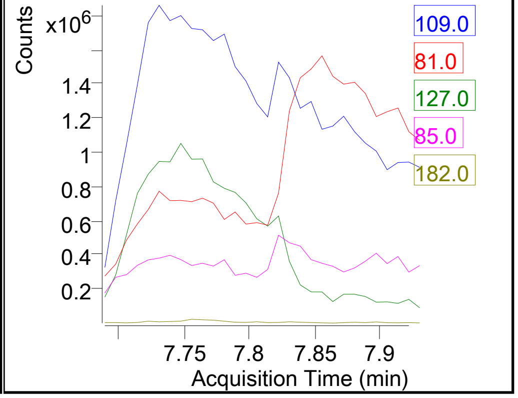

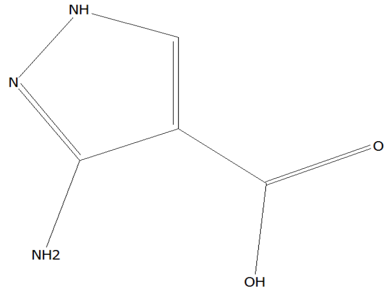

Component RT	Compound Name	Component Area	Match Factor	CAS#	Formula	Estimated Conc.
7.8561	1,7-Octadiene-3,6-diol, 2,6-dimethyl-	403789701.6	83.5	51276-33-6	C10H18O2	

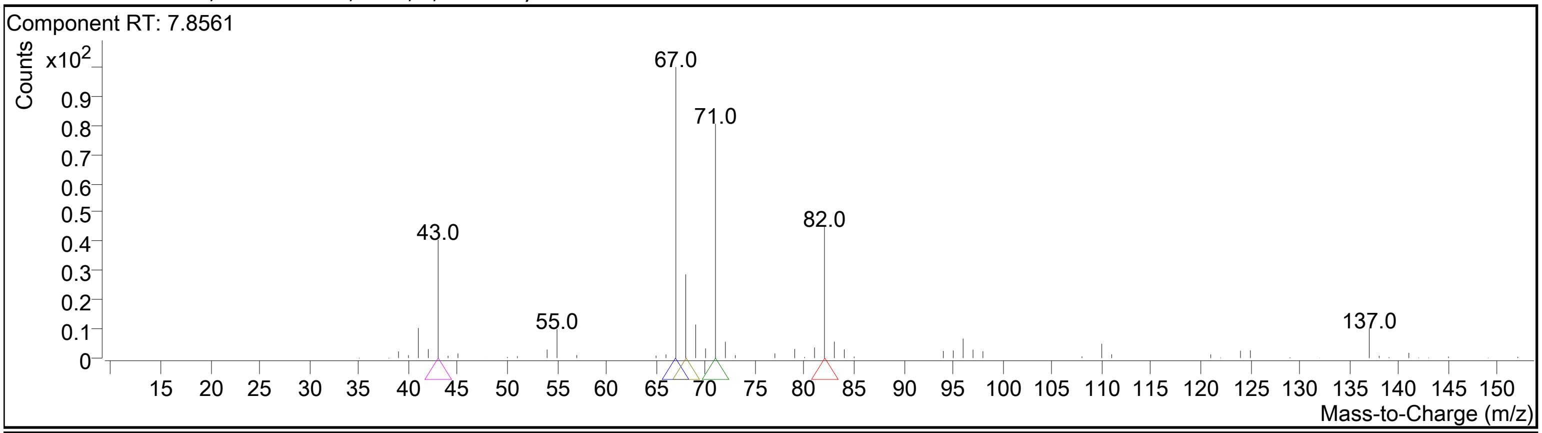

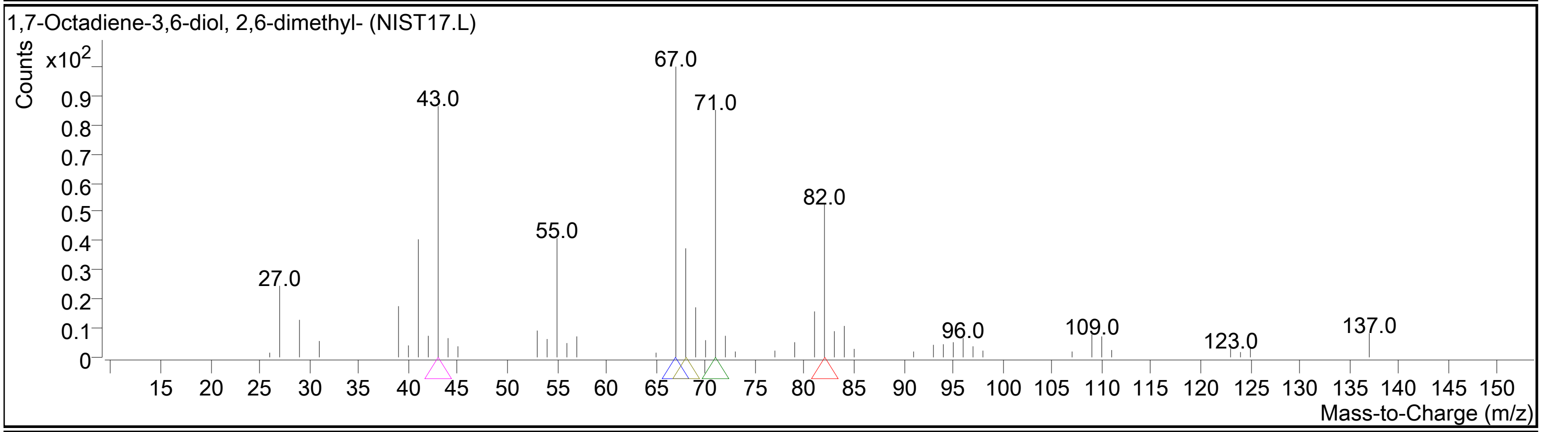

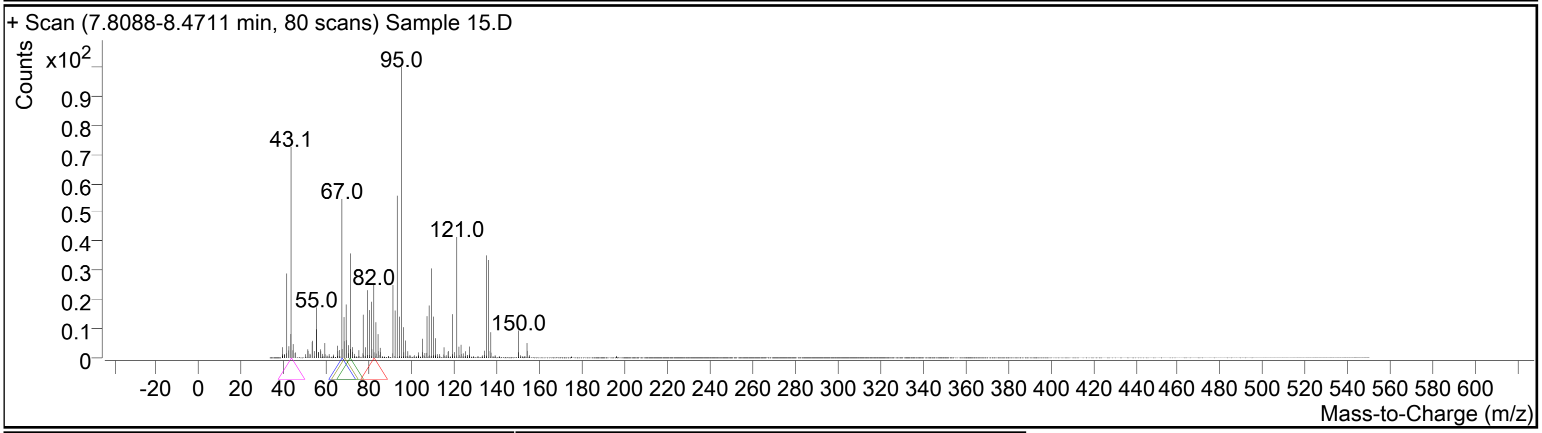

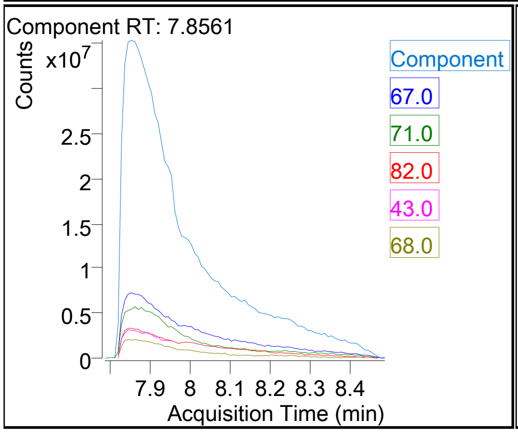

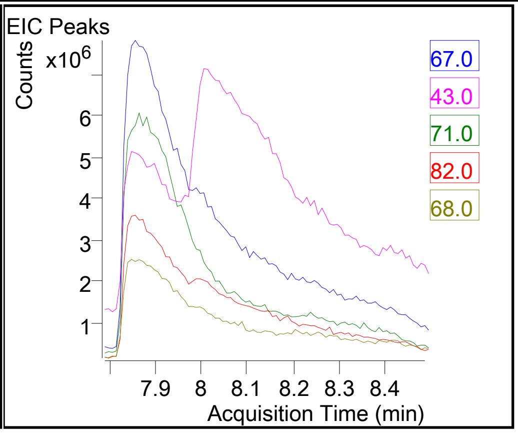

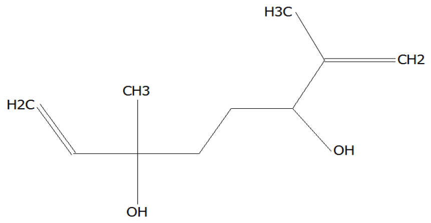

Component RT	Compound Name	Component Area	Match Factor	CAS#	Formula	Estimated Conc.
7.9053	Formamide, N-methyl-	5003176.0	72.6	123-39-7	C2H5NO	

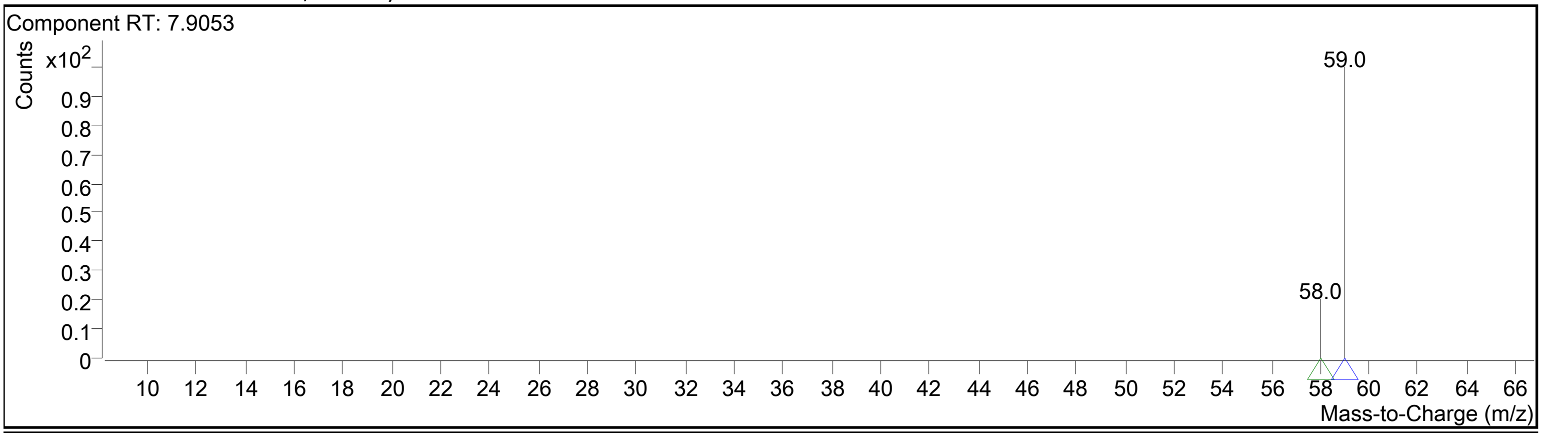

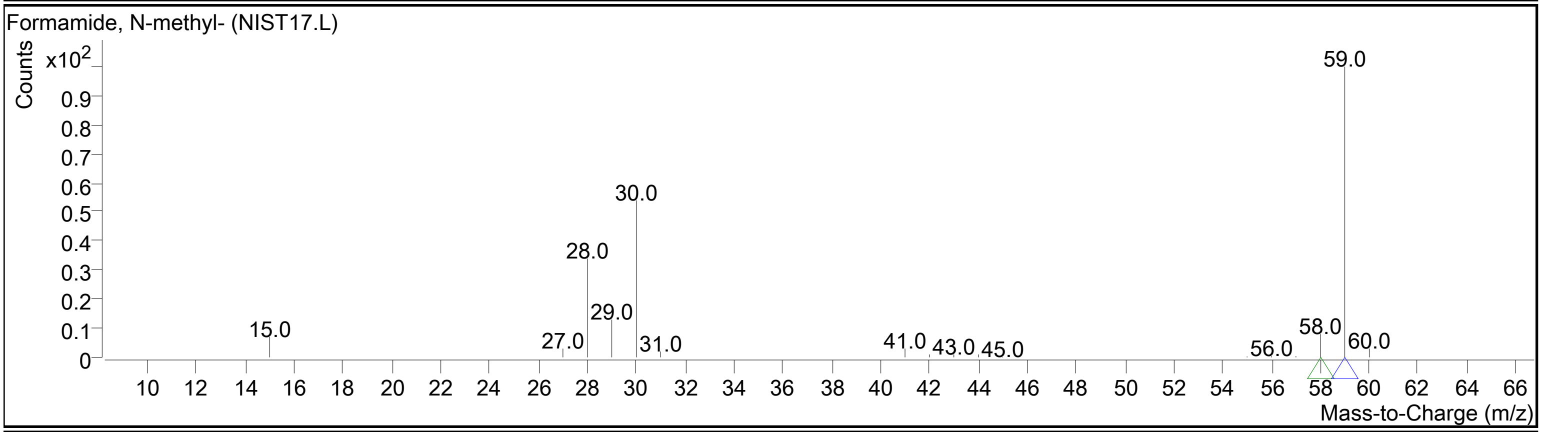

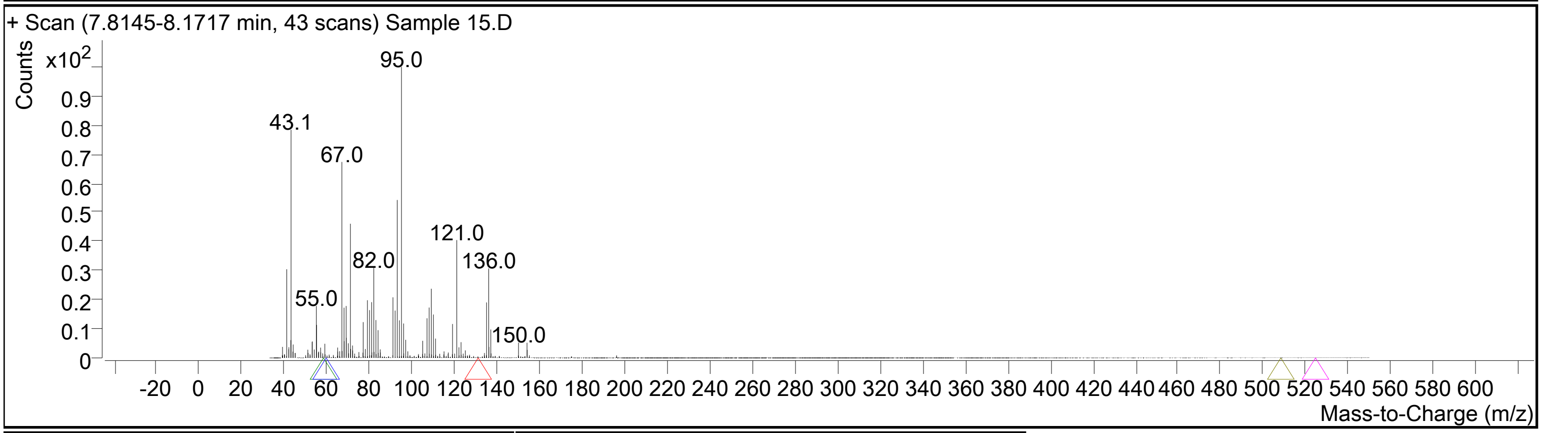

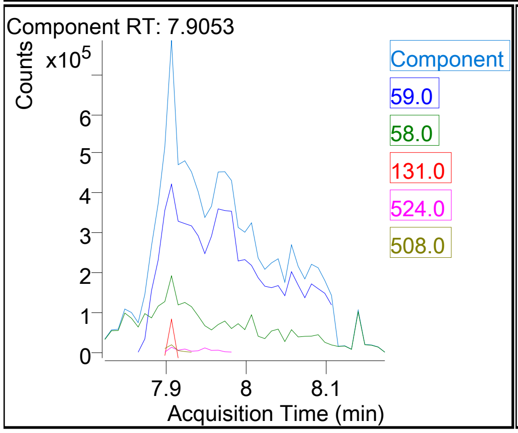

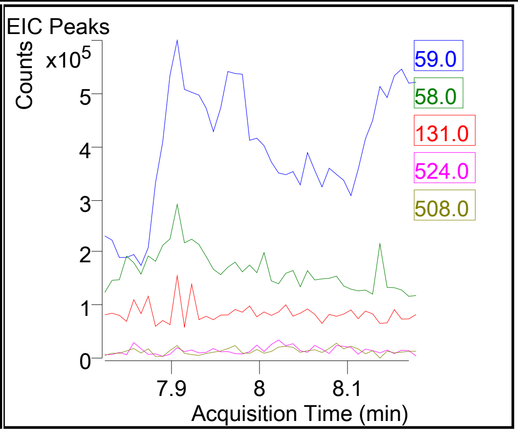

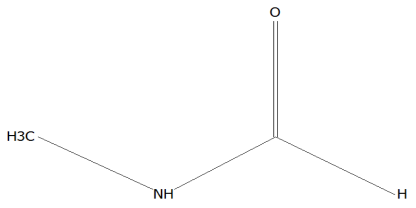

Agilent Technologies

Component RT	Compound Name	Component Area	Match Factor	CAS#	Formula	Estimated Conc.
8.0794	Cyanogen bromide	18653312.1	67.5	506-68-3	CBrN	

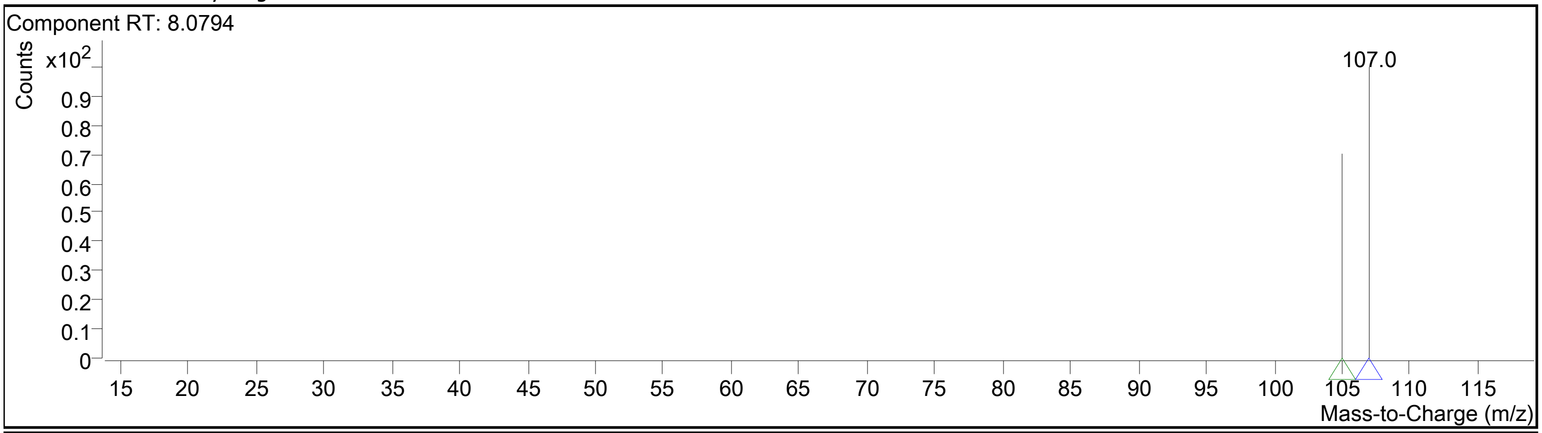

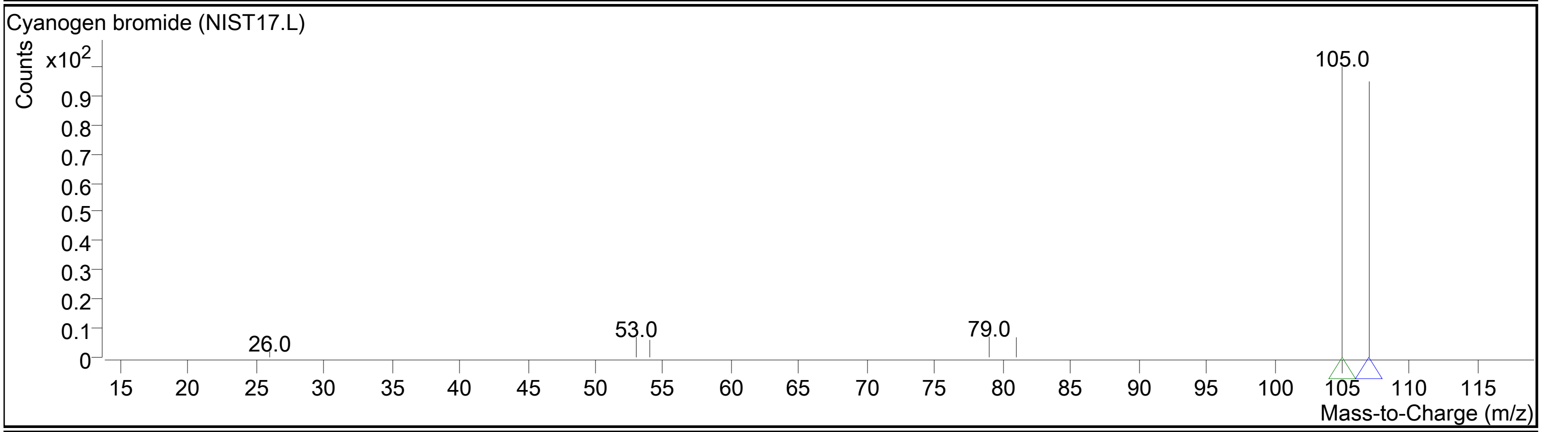

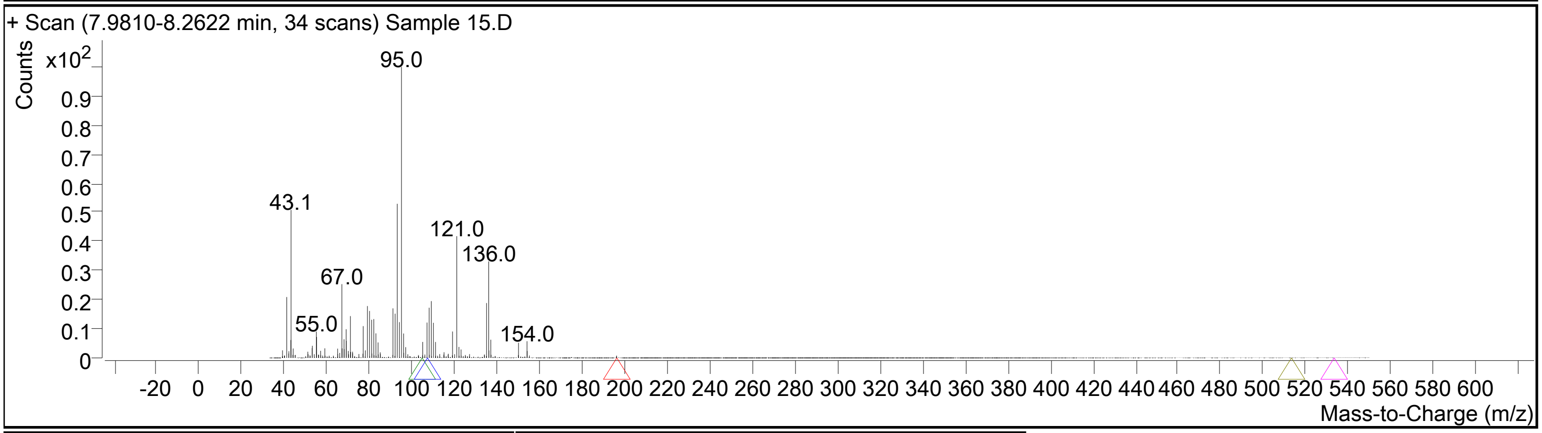

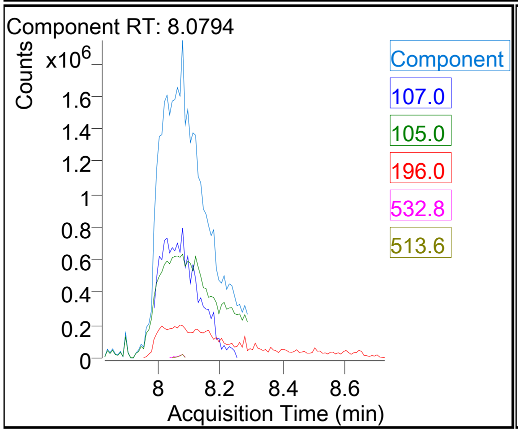

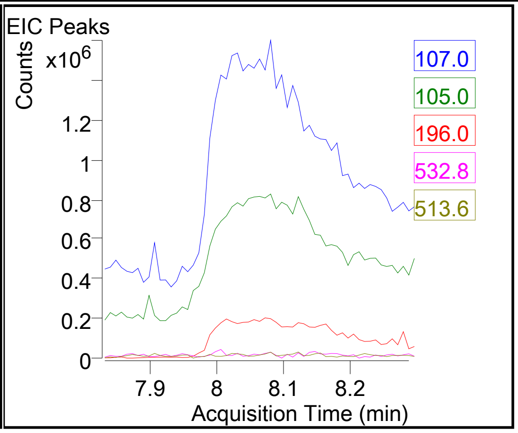

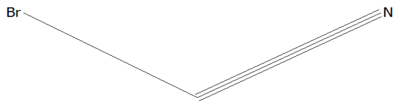

Agilent Technologies

Component RT	Compound Name	Component Area	Match Factor	CAS#	Formula	Estimated Conc.
8.3380	Benzene, 1,2,3-trimethyl-	9722788.4	65.3	526-73-8	C9H12	

Component RT: 8.3380

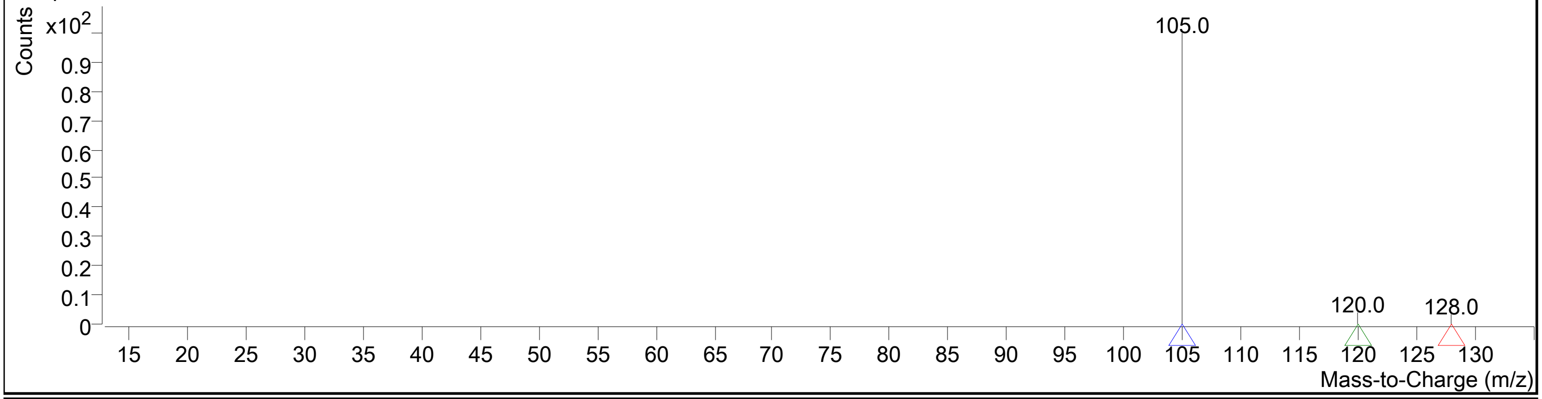

Benzene, 1,2,3-trimethyl- (NIST17.L)

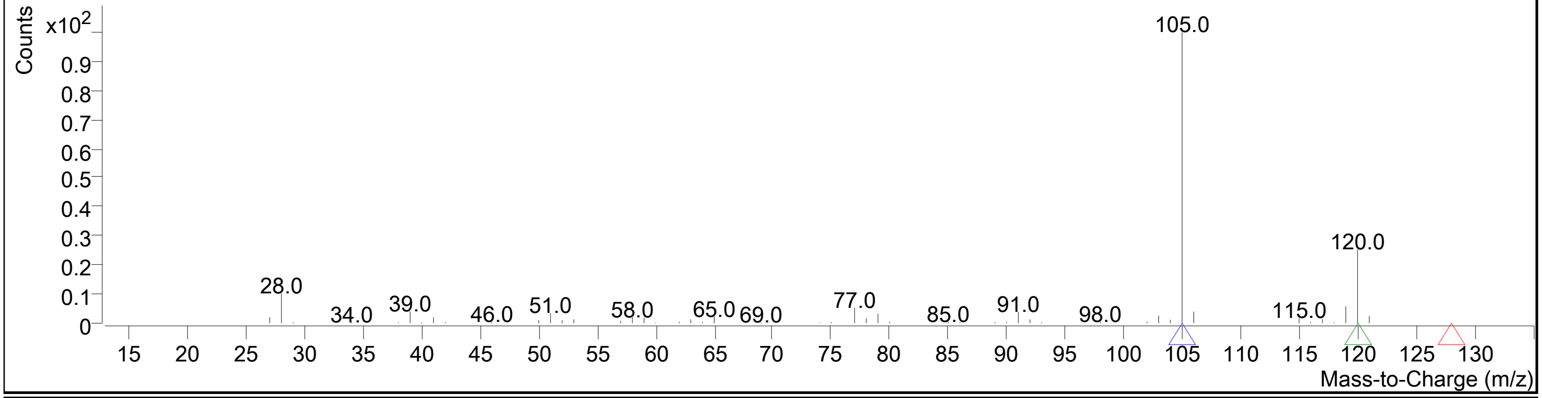

+ Scan (8.2833-8.4544 min, 21 scans) Sample 15.D

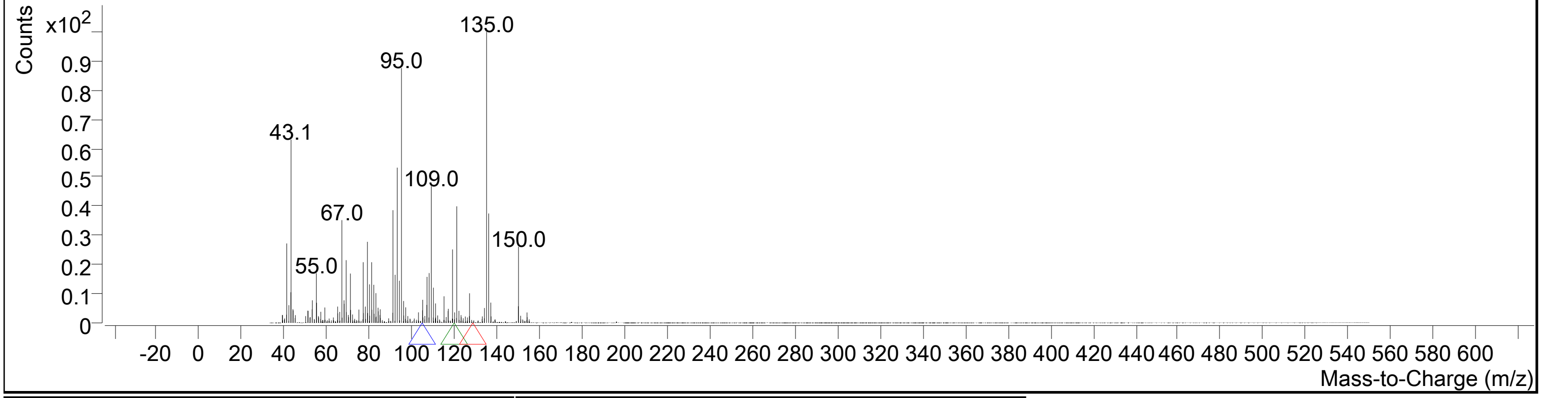

Component RT: 8.3380

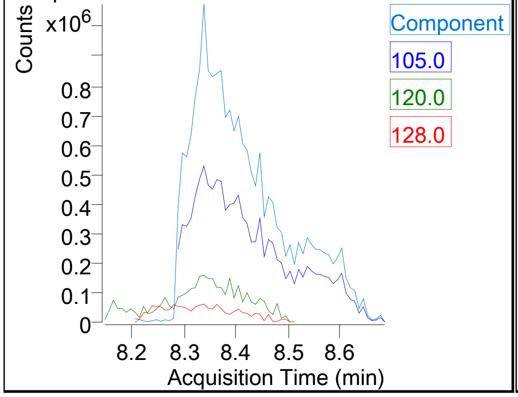

EIC Peaks

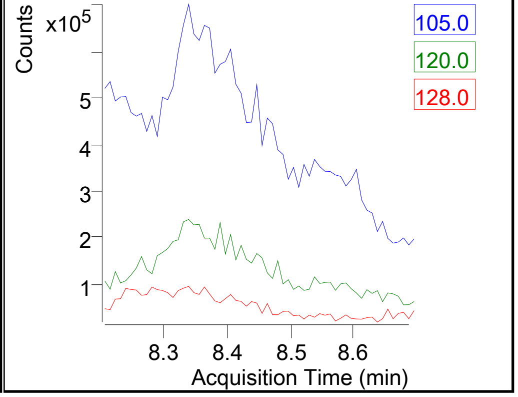

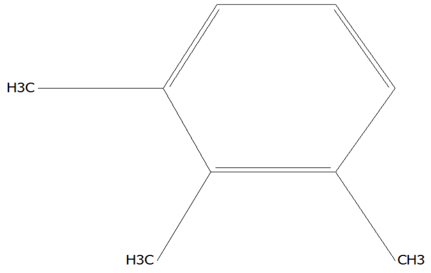

Component RT	Compound Name	Component Area	Match Factor	CAS#	Formula	Estimated Conc.
8.3502	Thymol	96926703.6	82.0	89-83-8	C10H14O	

Component RT: 8.3502

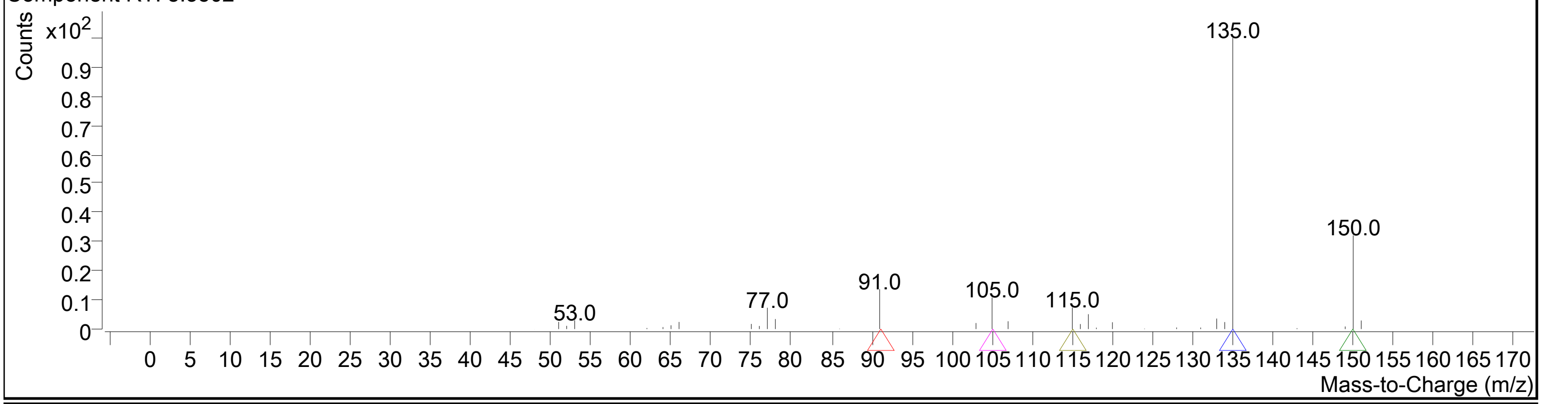

Thymol (NIST17.L)

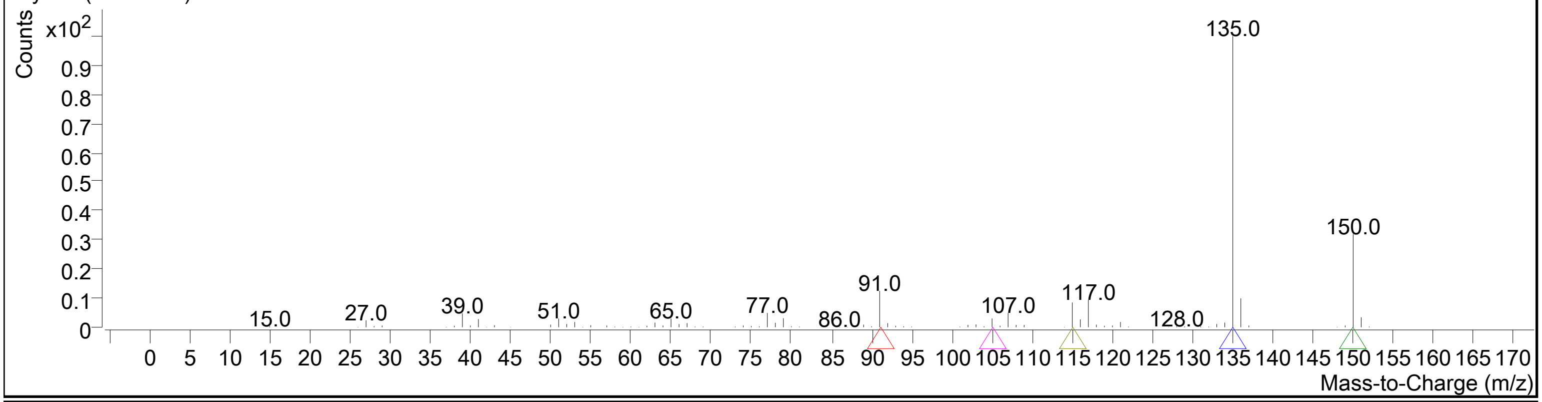

+ Scan (8.2800-8.7952 min, 62 scans) Sample 15.D

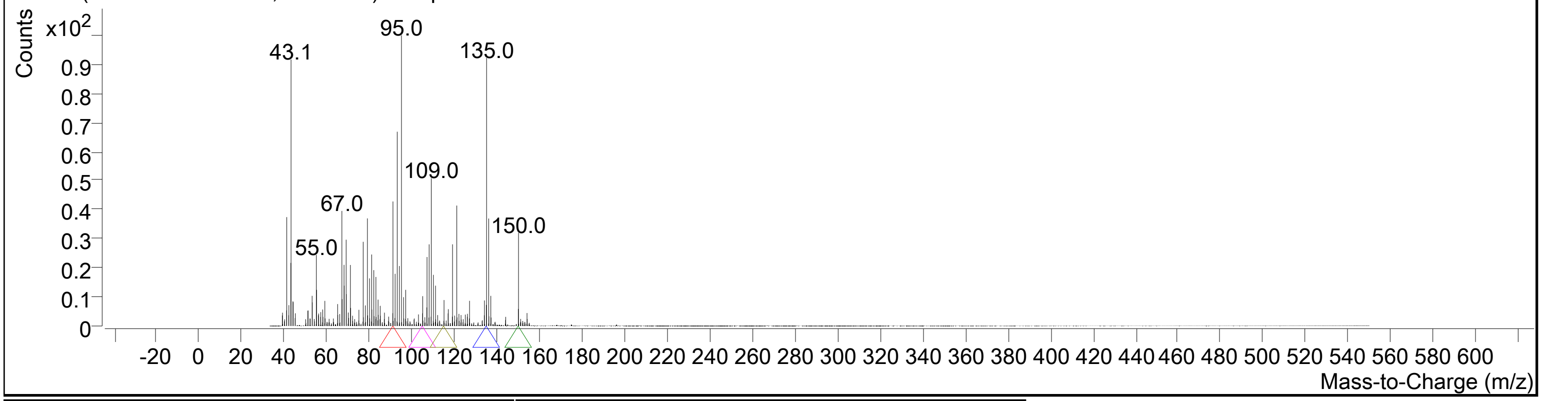

Component RT: 8.3502

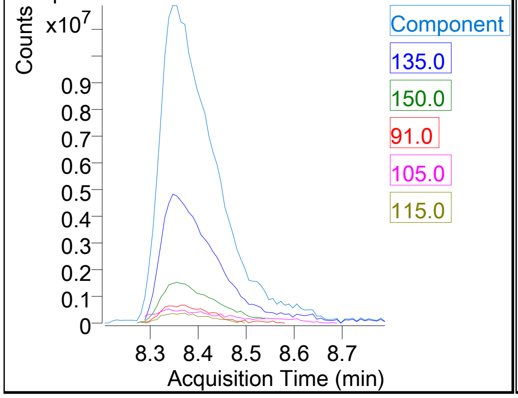

EIC Peaks

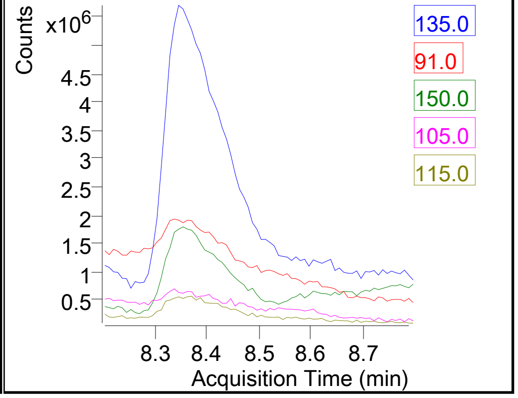

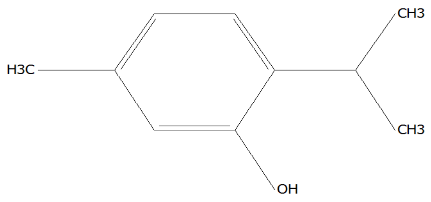

Component RT	Compound Name	Component Area	Match Factor	CAS#	Formula	Estimated Conc.
9.2609	Benzaldehyde, 4-benzyloxy-3-methoxy-2-nitro-	9540564.0	71.9	2450-27-3	C15H13NO5	

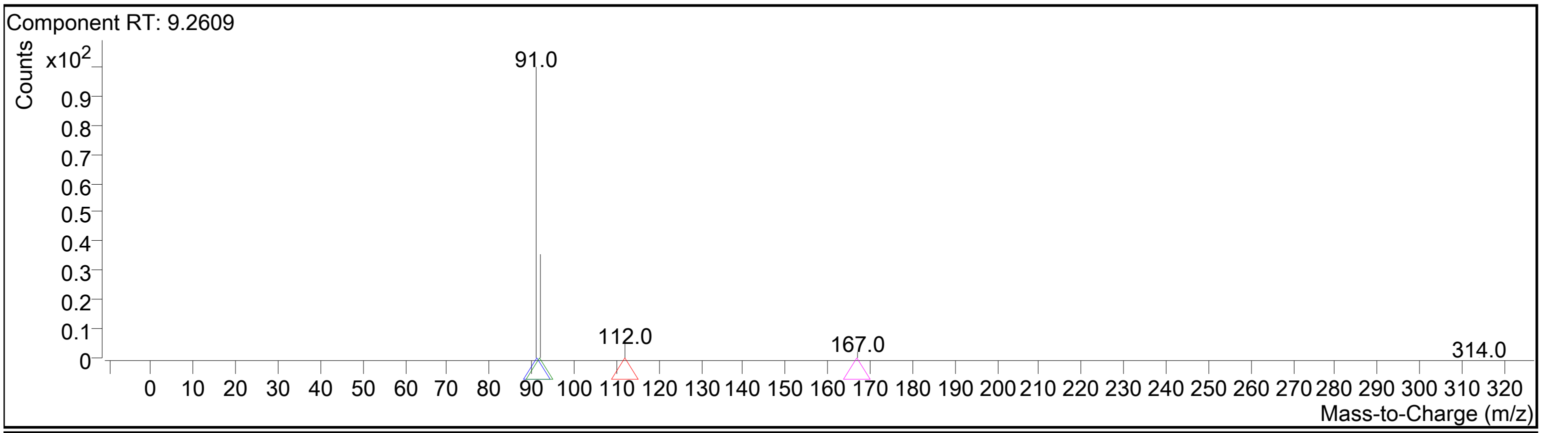

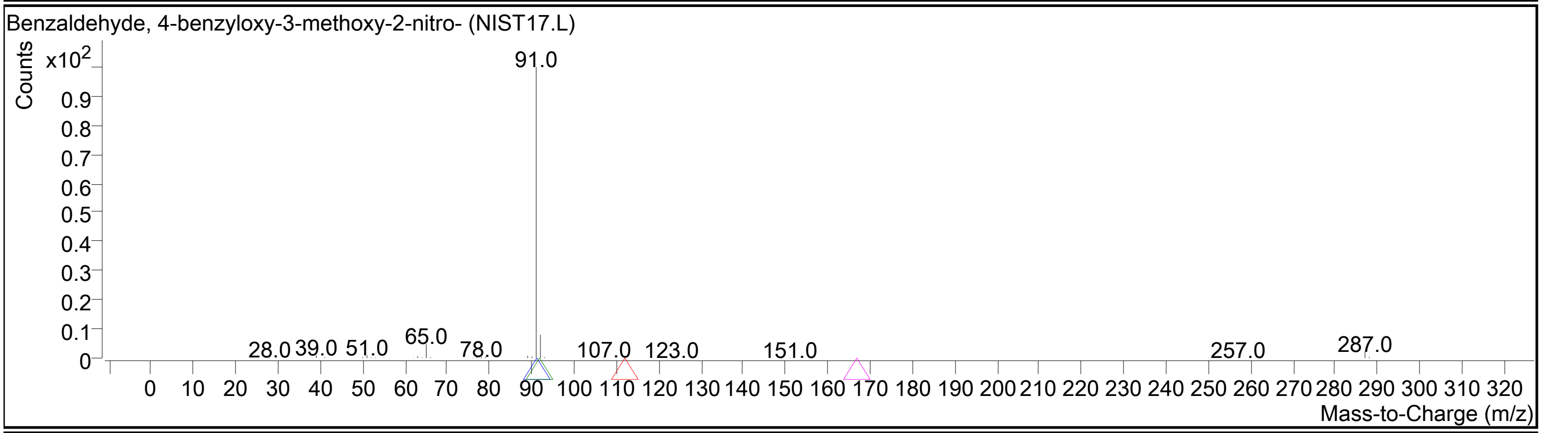

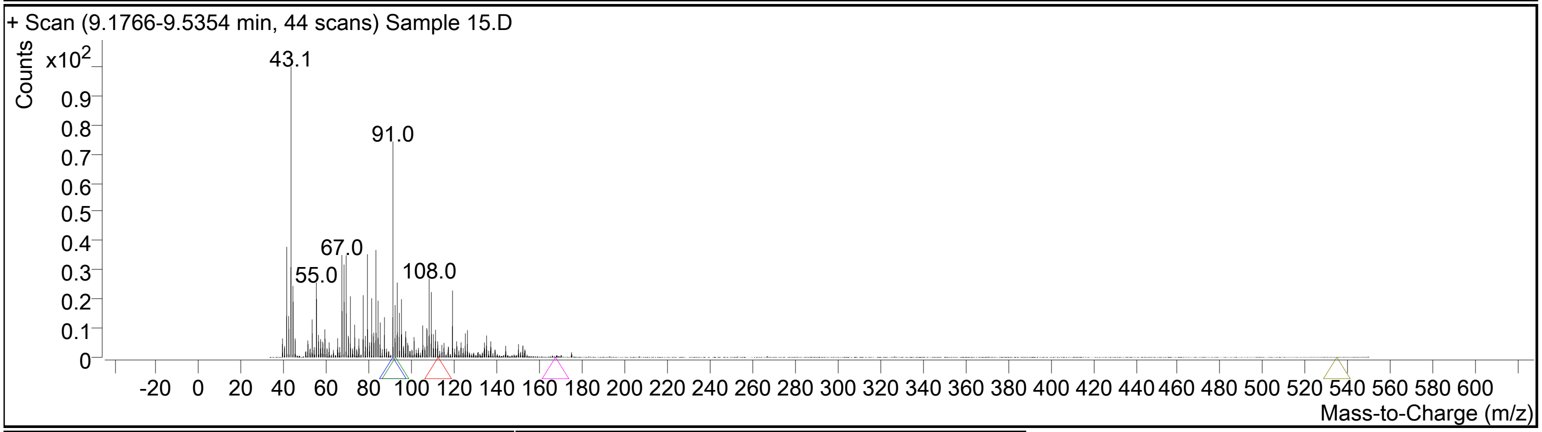

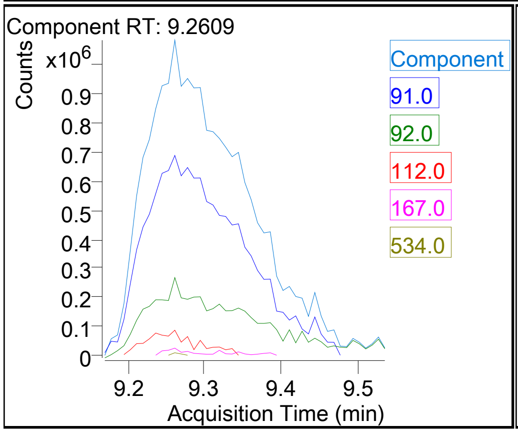

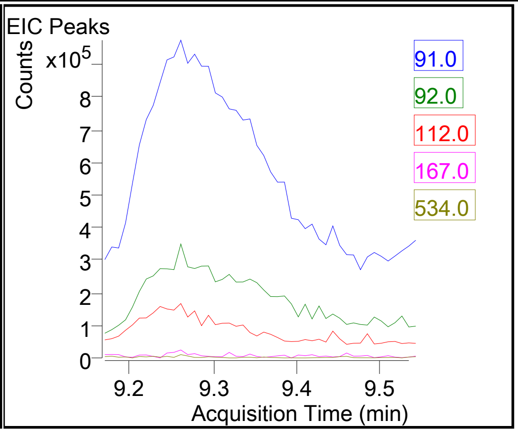

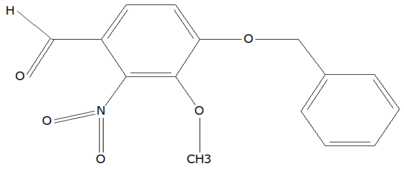

Component RT	Compound Name	Component Area	Match Factor	CAS#	Formula	Estimated Conc.
9.5695	Benzenamine, 4-ethoxy-	5218086.8	69.4	156-43-4	C8H11NO	

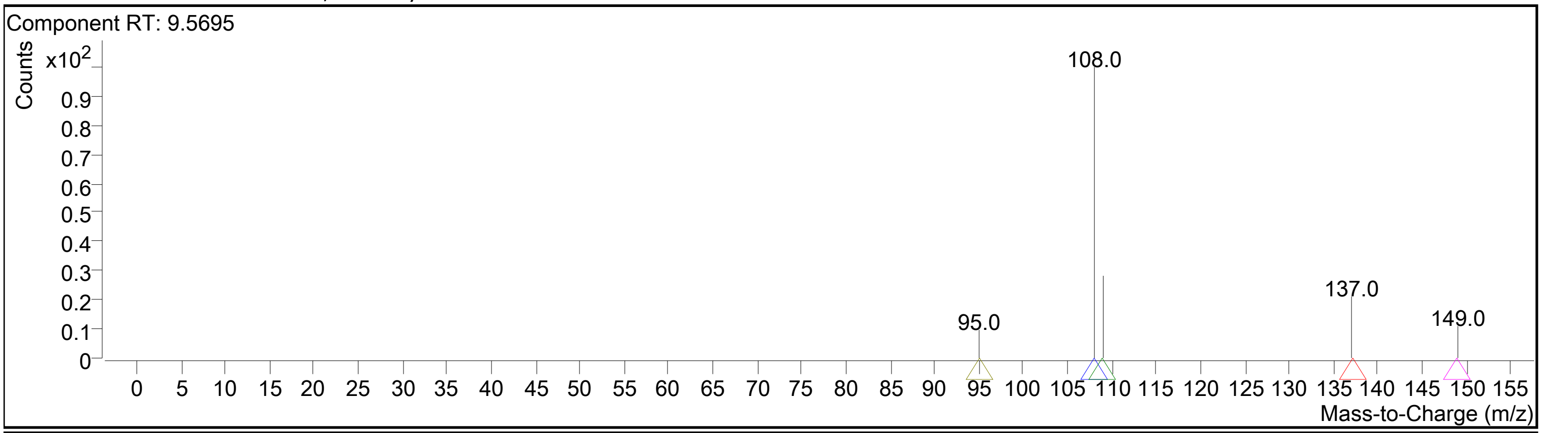

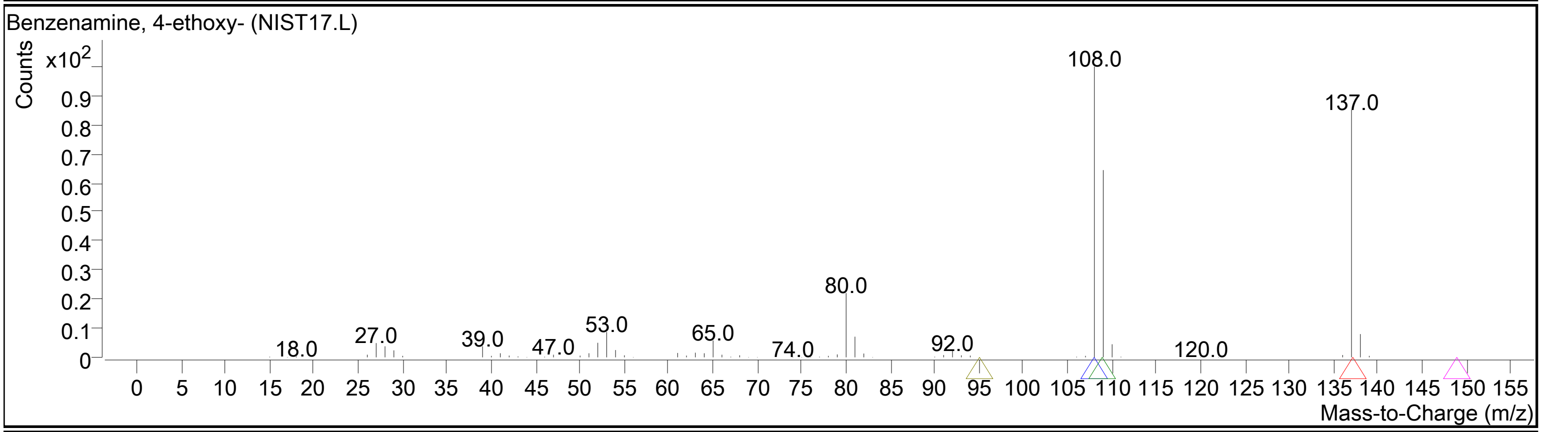

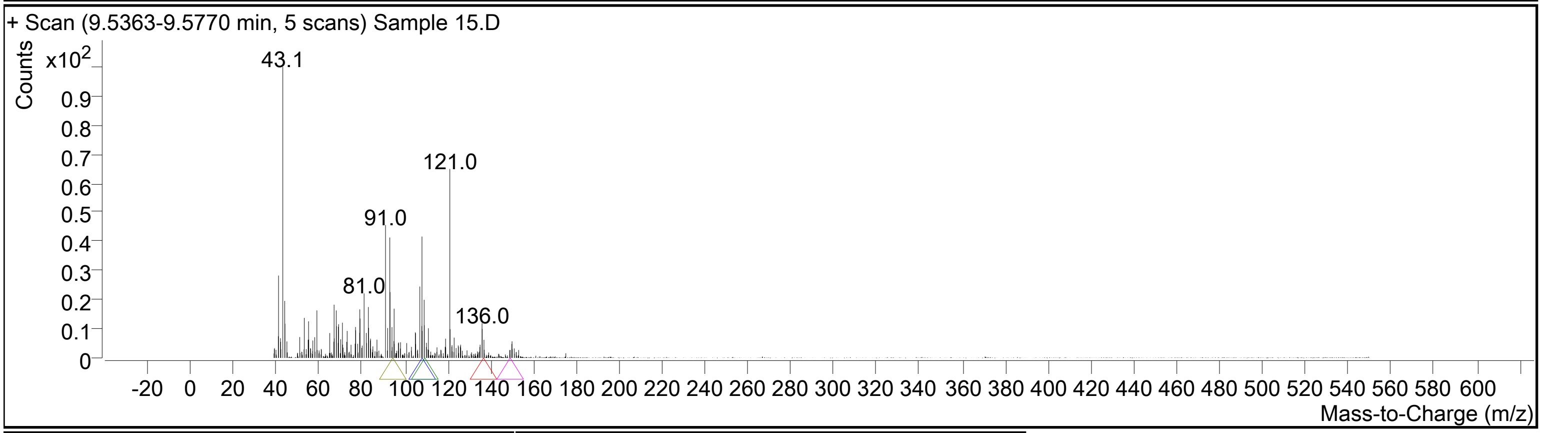

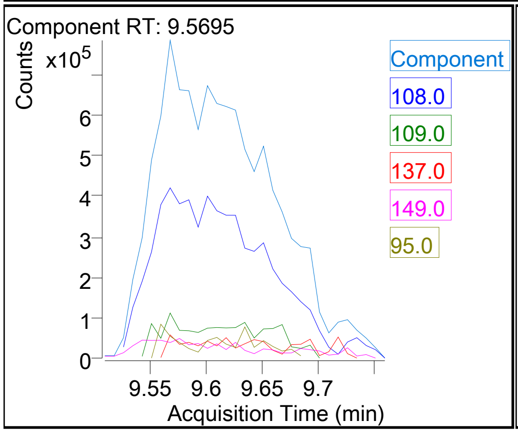

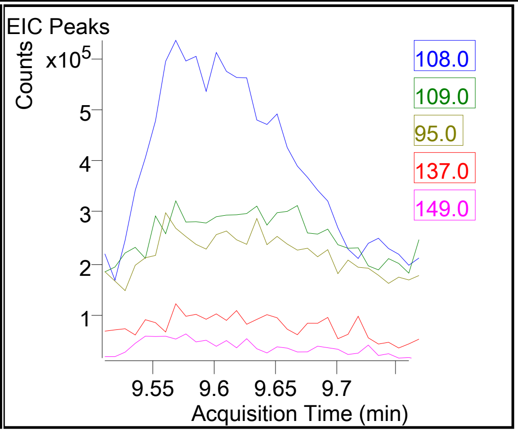

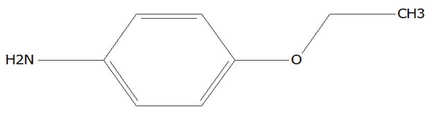

Component RT	Compound Name	Component Area	Match Factor	CAS#	Formula	Estimated Conc.
9.6134	2-Methylbicyclo[4.3.0]non-1(6)-ene	37456296.8	80.0	60223-07-6	C10H16	

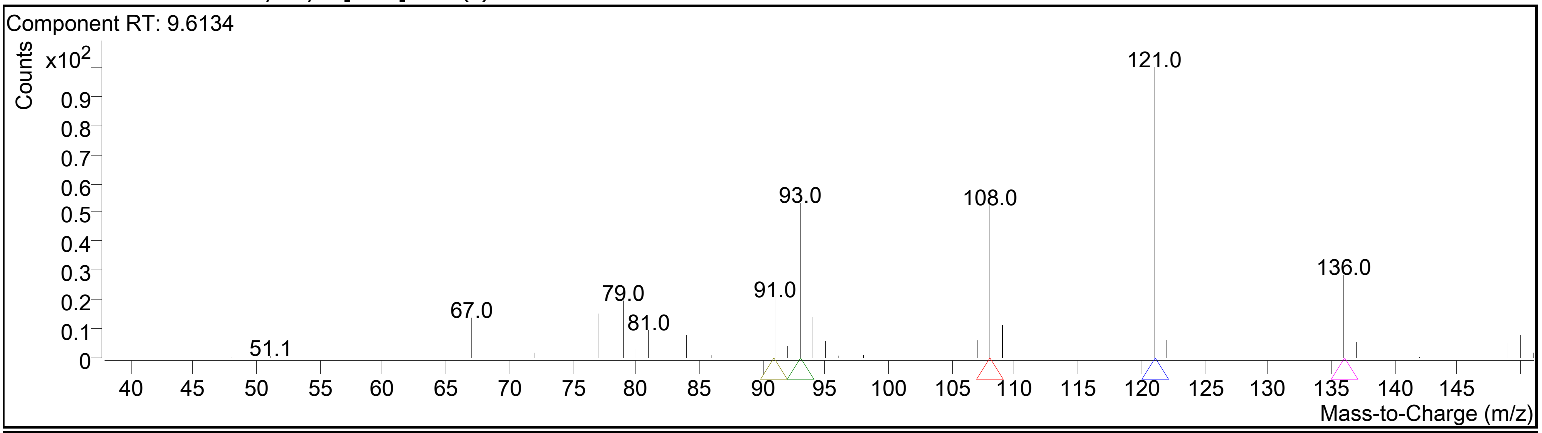

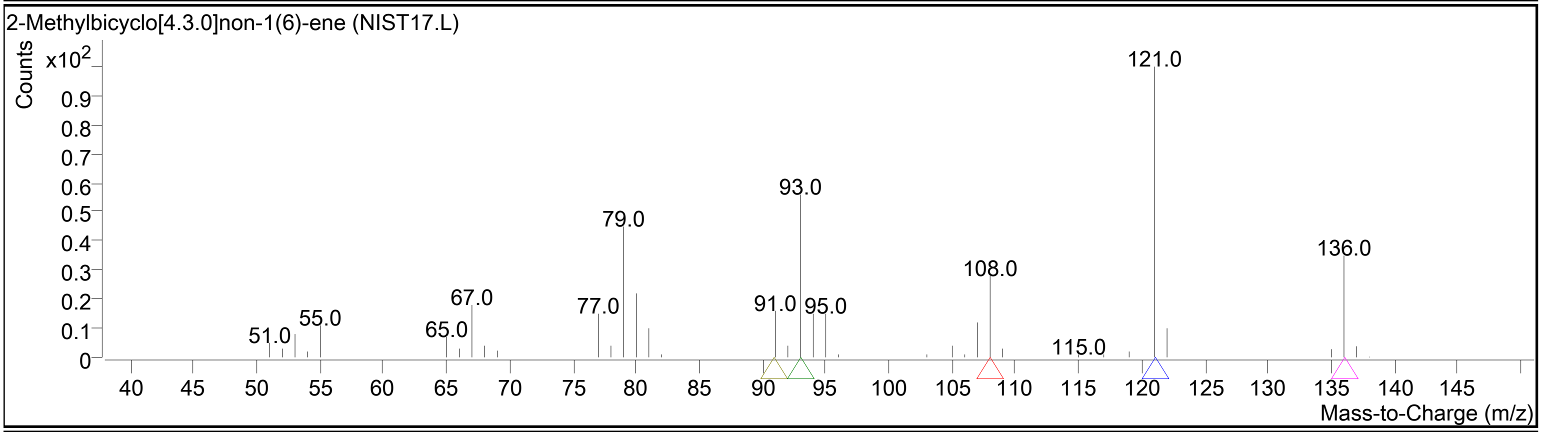

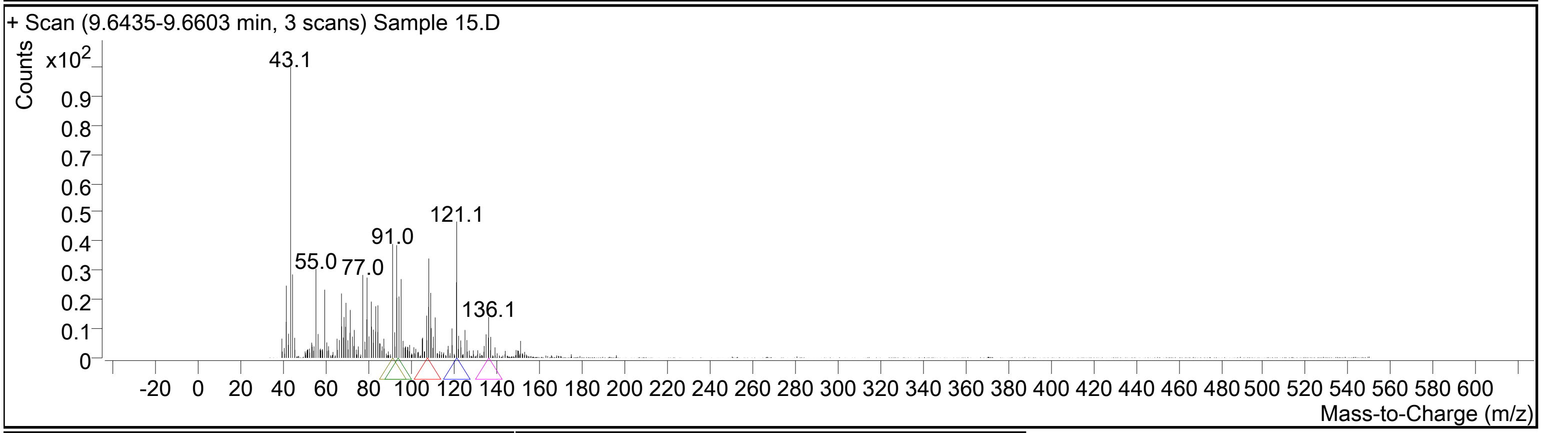

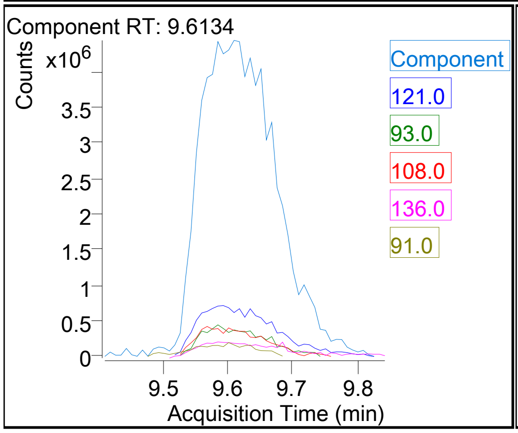

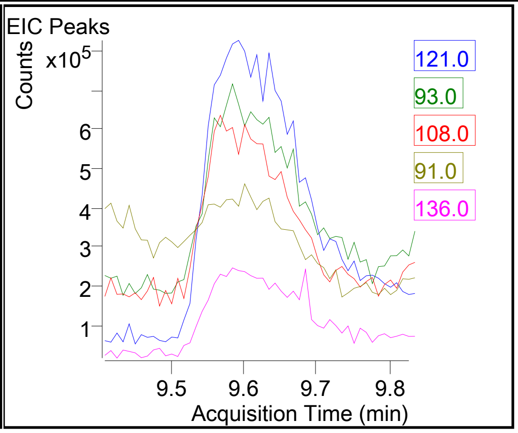

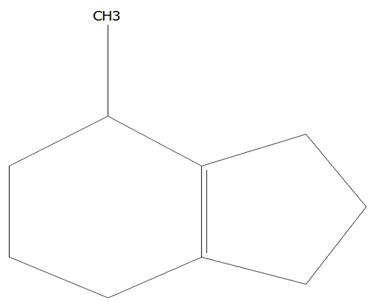

Component RT	Compound Name	Component Area	Match Factor	CAS#	Formula	Estimated Conc.
9.9797	Eugenol	77500138.1	84.8	97-53-0	C10H12O2	

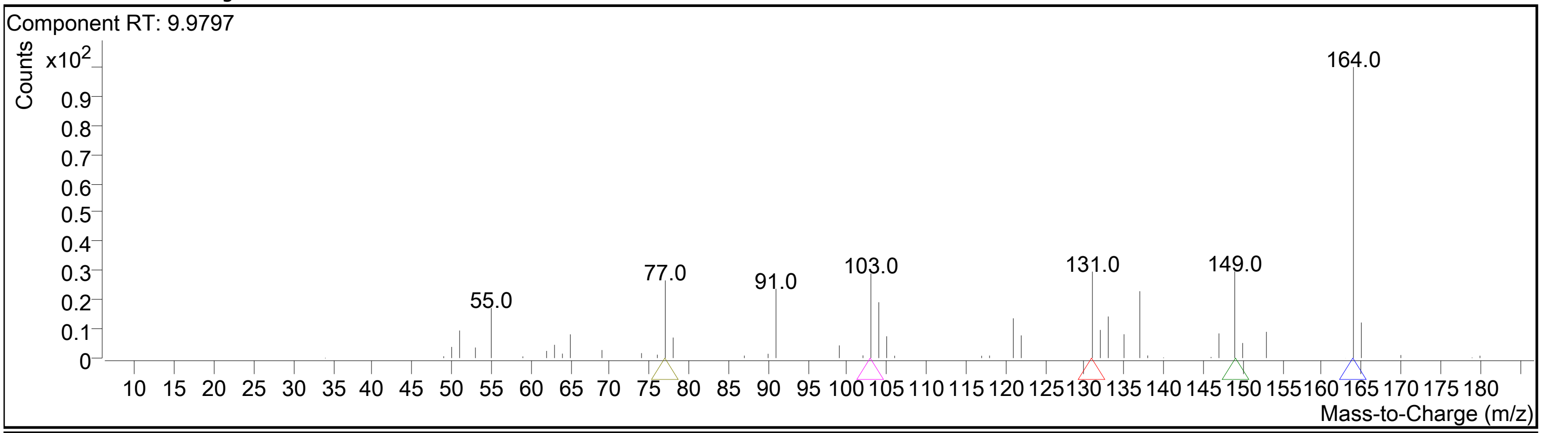

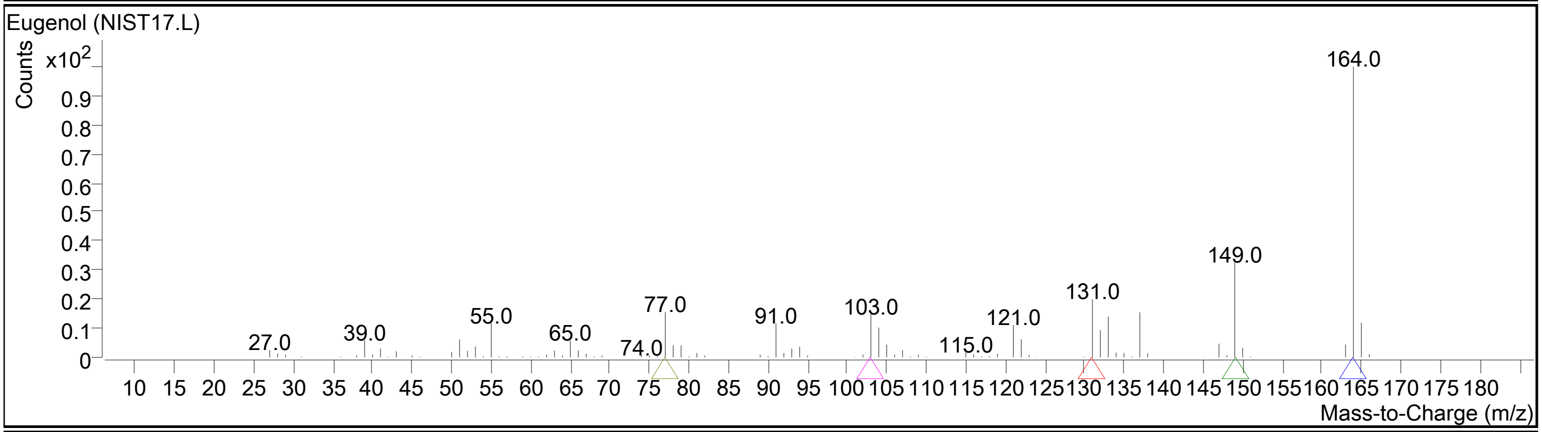

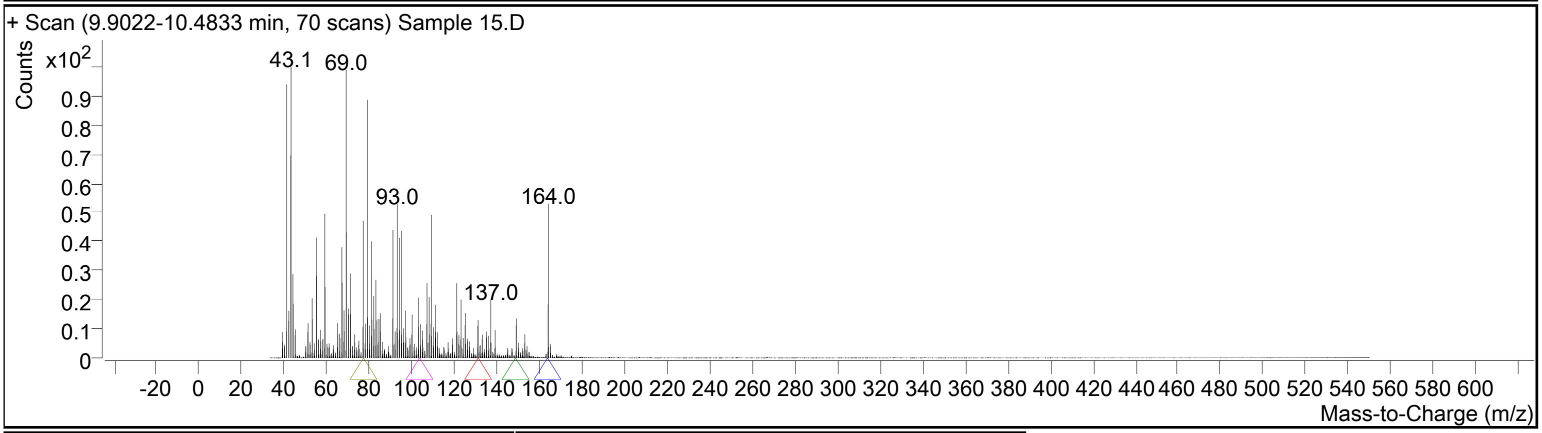

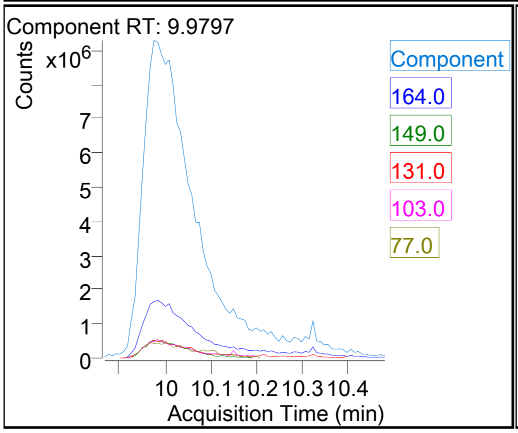

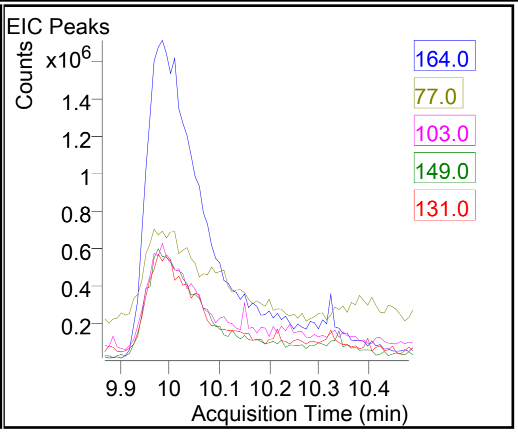

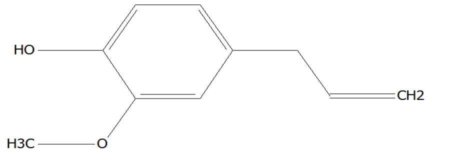

Agilent Technologies

Component RT	Compound Name	Component Area	Match Factor	CAS#	Formula	Estimated Conc.
10.0968	p-Mentha-1(7),2-dien-8-ol	28571758.4	82.0	65293-09-6	C10H16O	

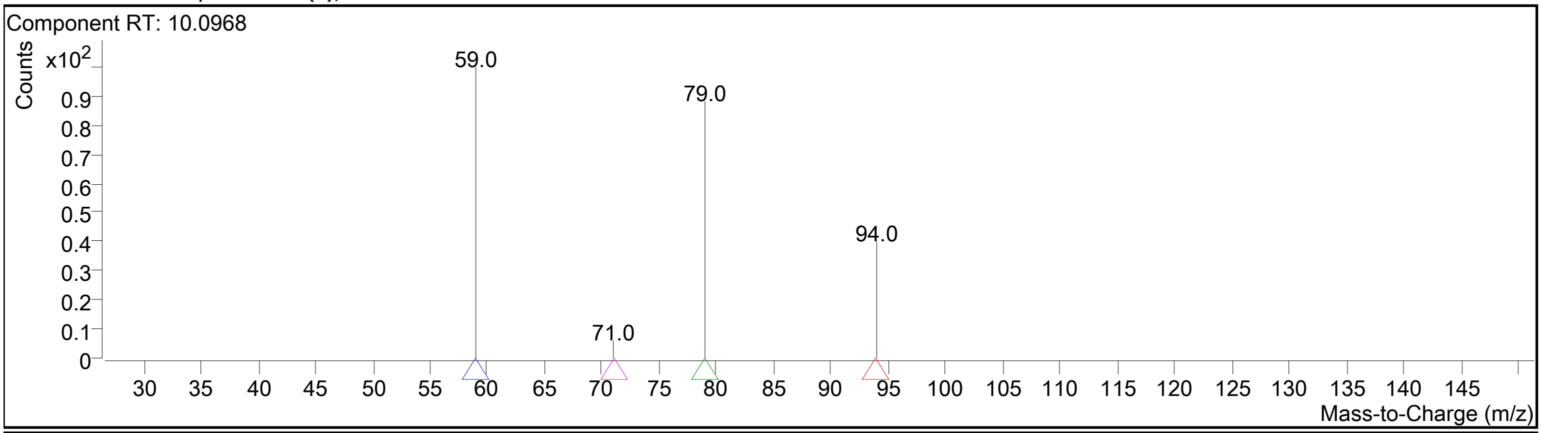

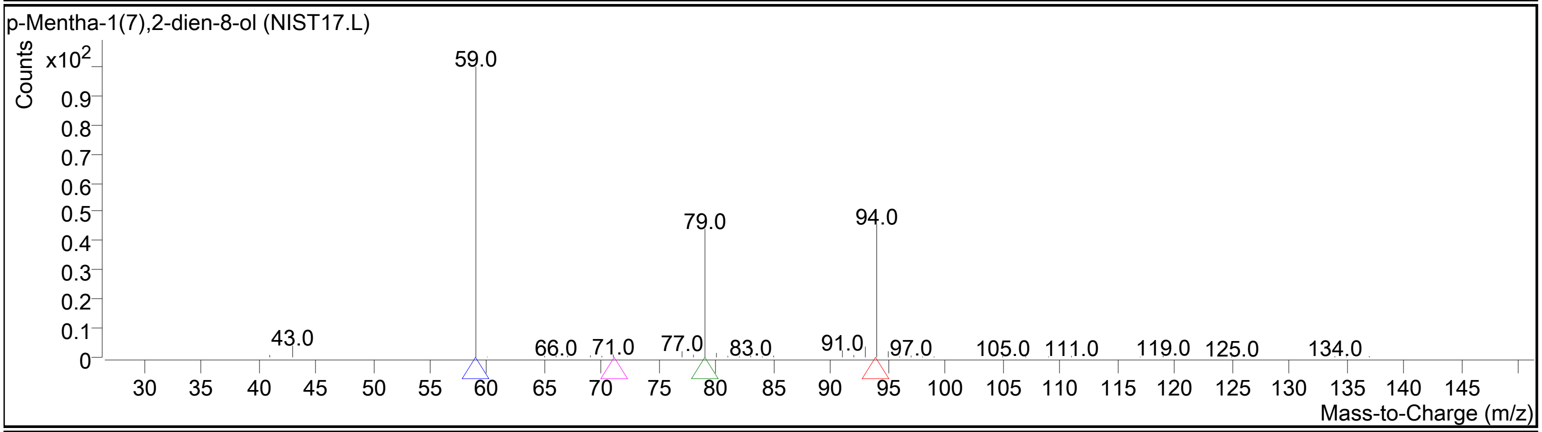

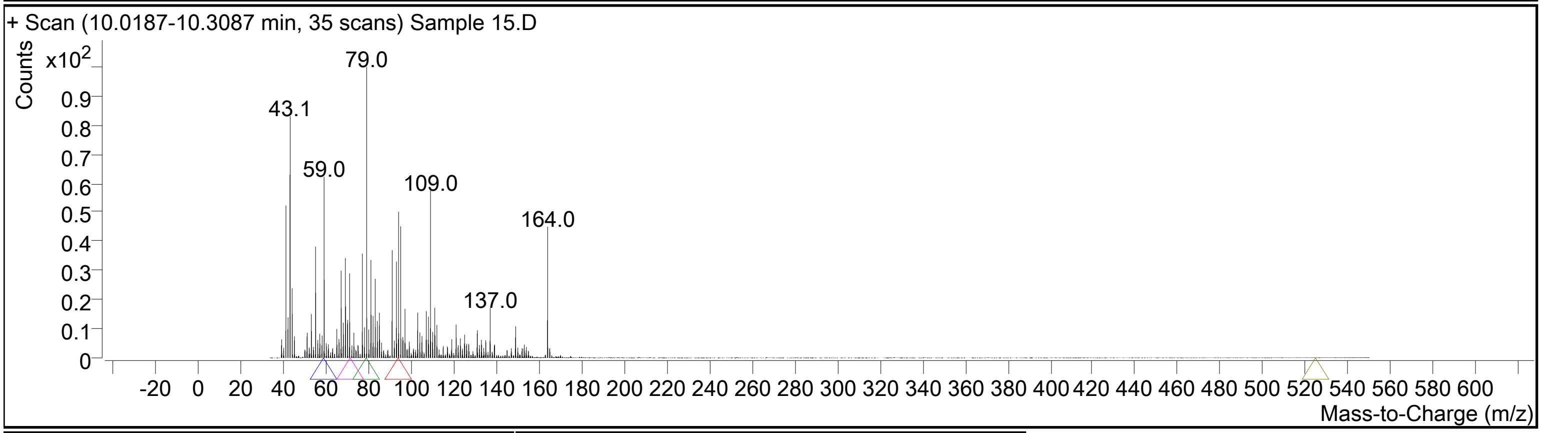

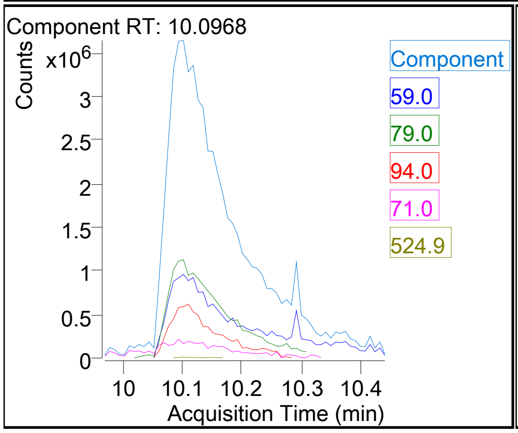

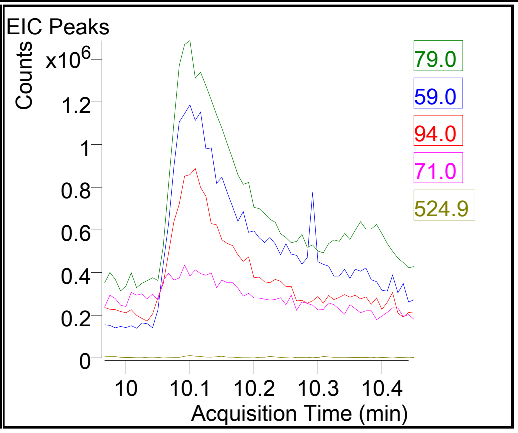

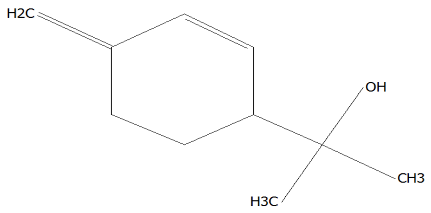

Agilent Technologies

Component RT	Compound Name	Component Area	Match Factor	CAS#	Formula	Estimated Conc.
10.4002	2,6-Octadienoic acid, 3,7-dimethyl-, (E)-	70257881.8	80.8	4698-08-2	C10H16O2	

Component RT: 10.4002

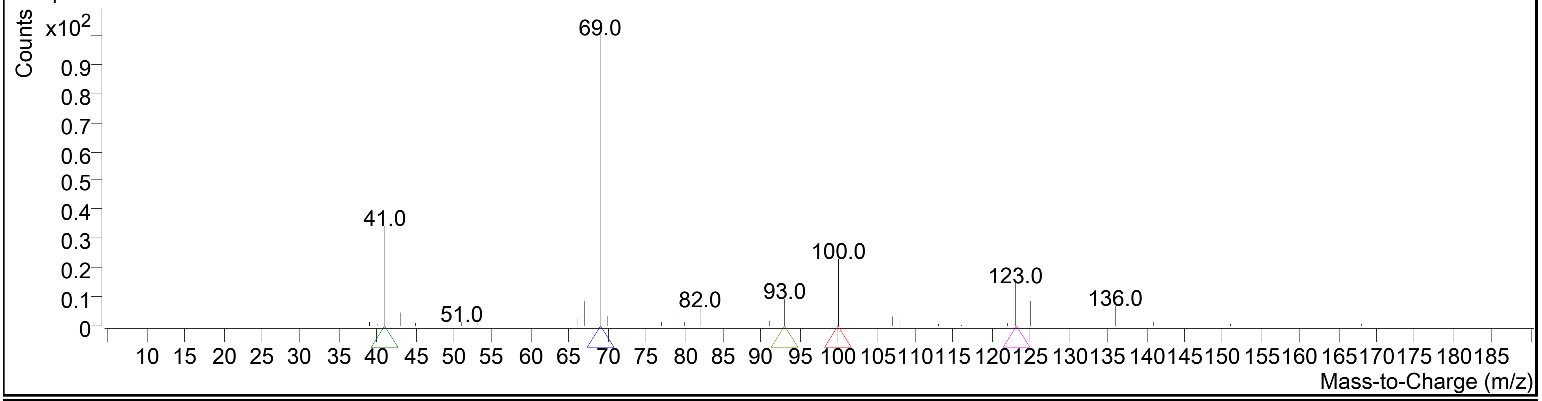

2,6-Octadienoic acid, 3,7-dimethyl-, (E)- (NIST17.L)

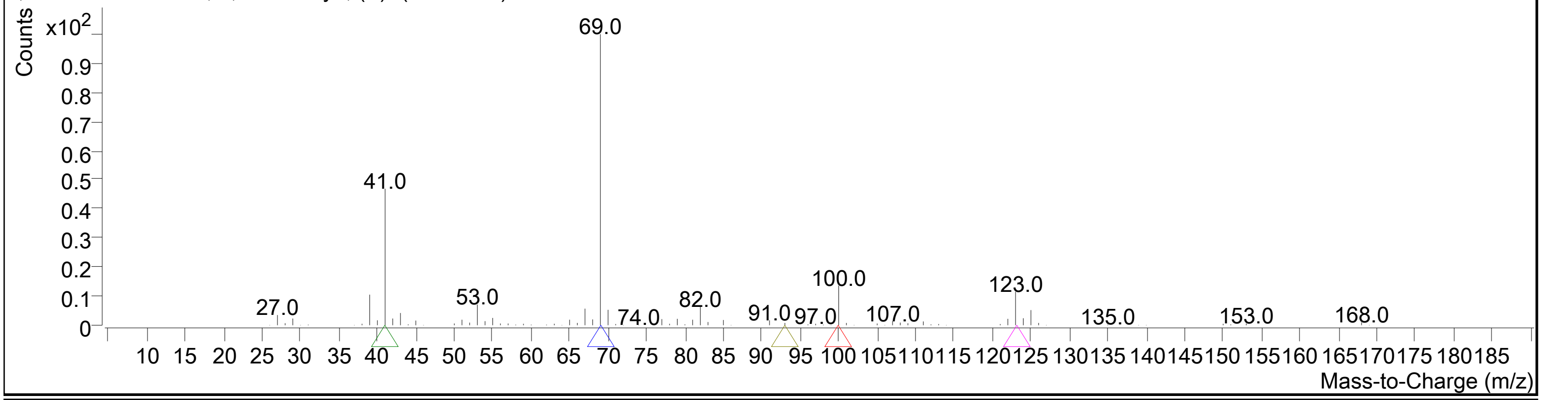

+ Scan (10.3419-10.4645 min, 15 scans) Sample 15.D

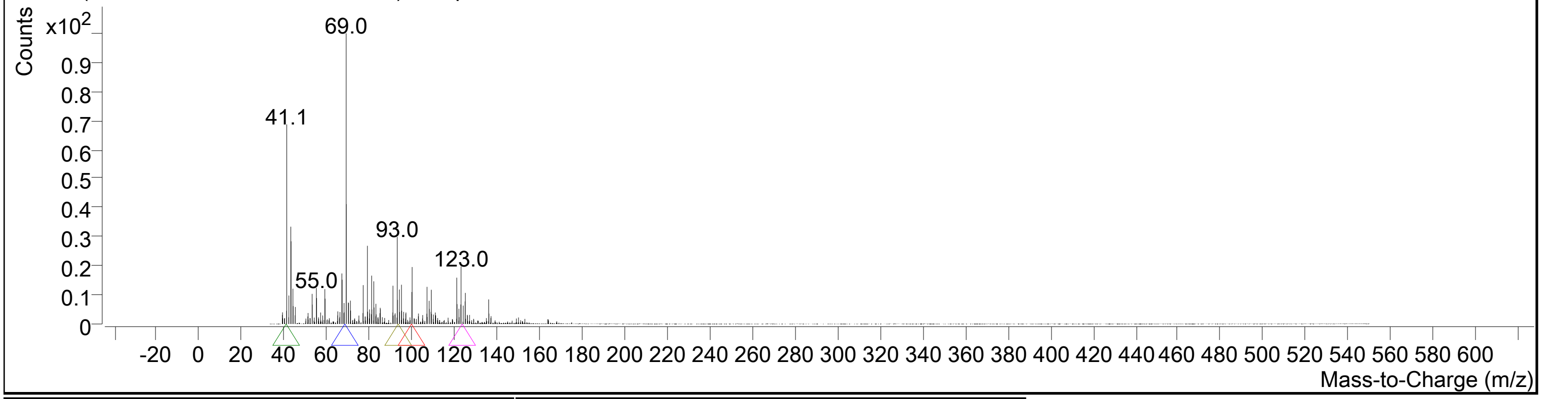

Component RT: 10.4002

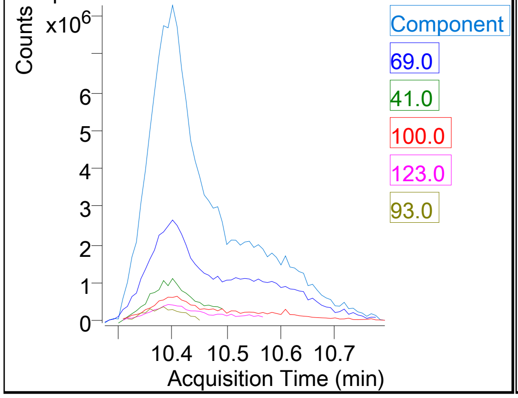

EIC Peaks

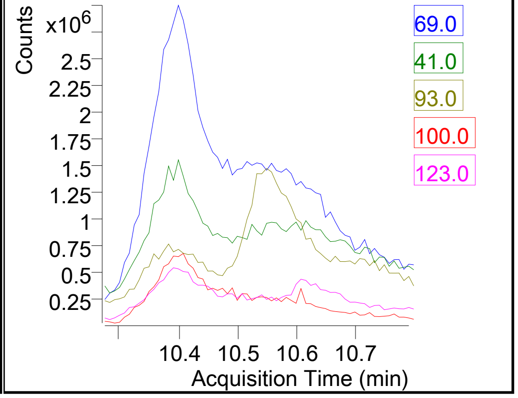

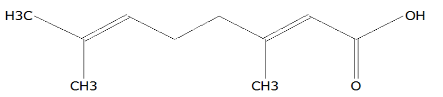

Agilent Technologies

Component RT	Compound Name	Component Area	Match Factor	CAS#	Formula	Estimated Conc.
10.5575	Furan, 2-(1-pentenyl)-, (E)-	7757846.8	65.9	20992-69-2	C9H12O	

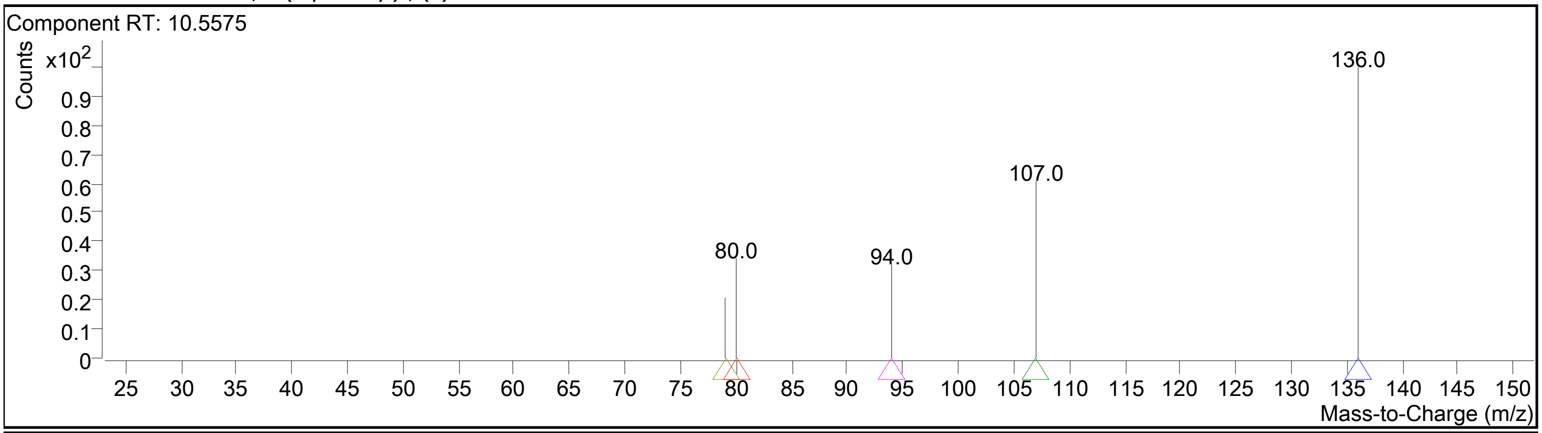

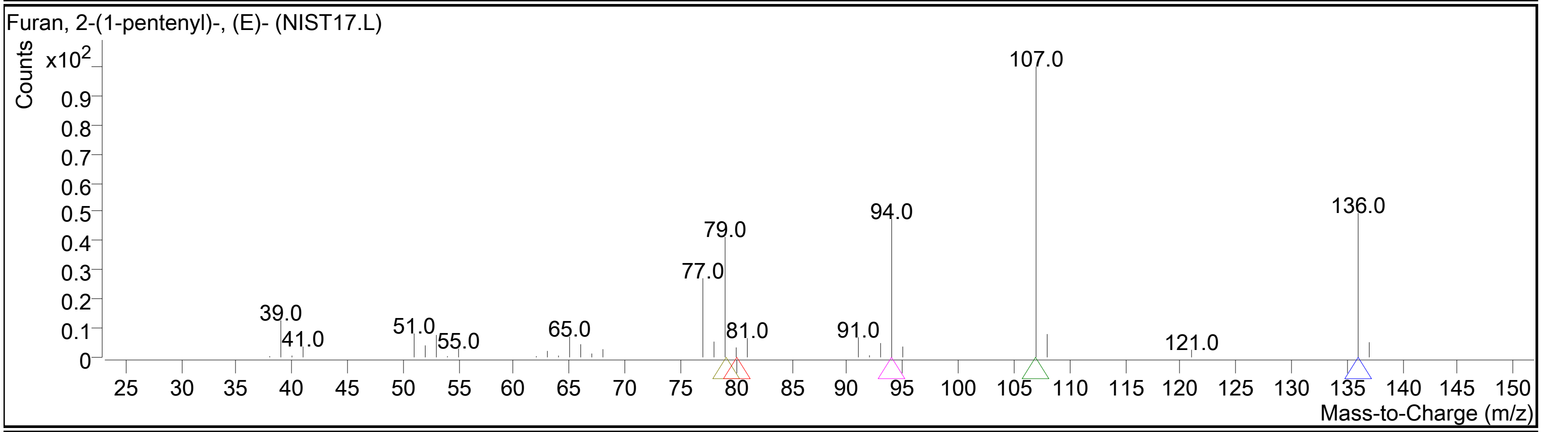

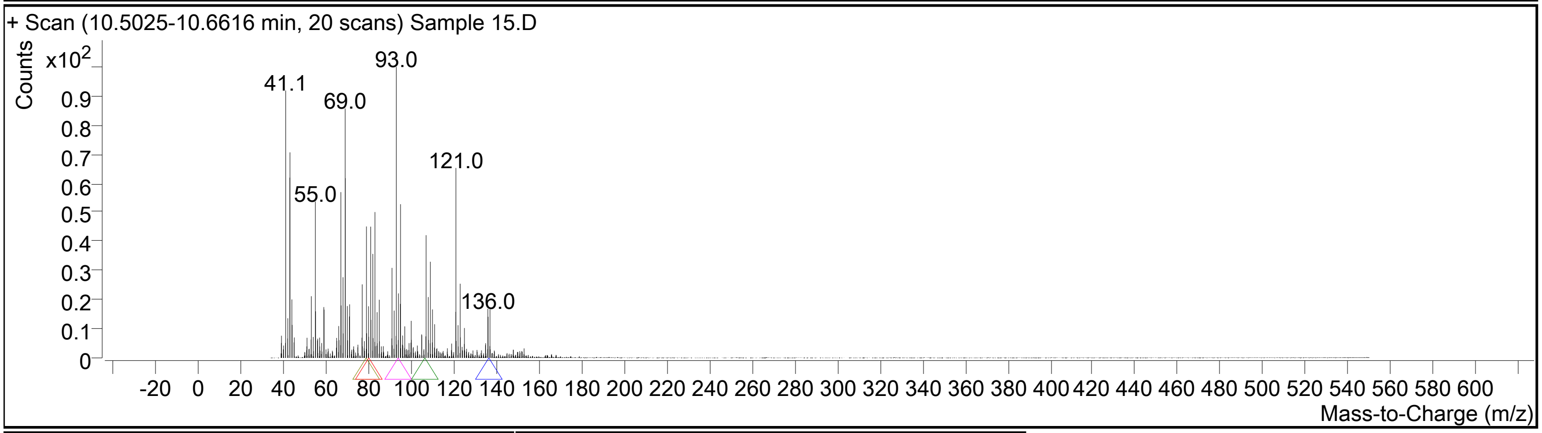

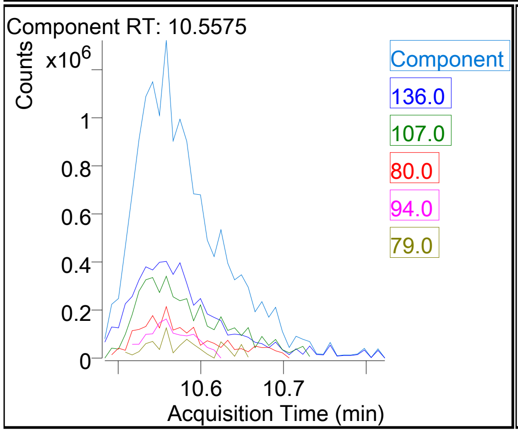

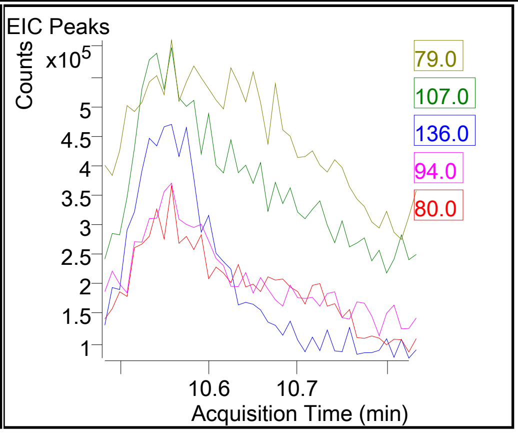

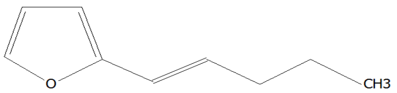

Component RT	Compound Name	Component Area	Match Factor	CAS#	Formula	Estimated Conc.
10.6136	Bicyclo[2.2.1]heptane, 2-chloro-2,3,3-trimethyl-	94067837.7	73.8	465-30-5	C10H17Cl	

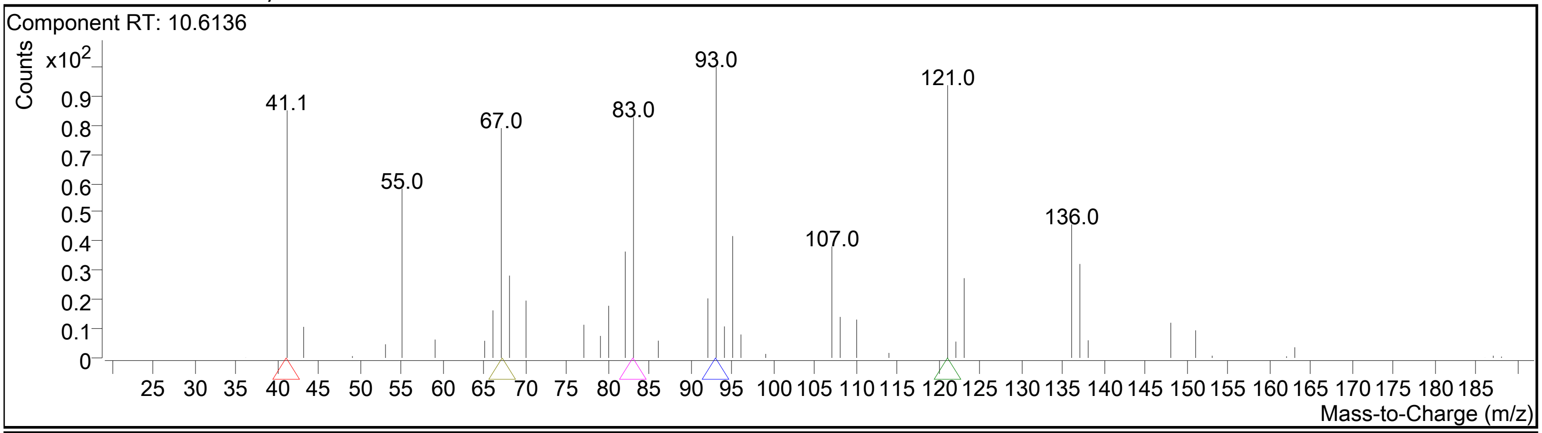

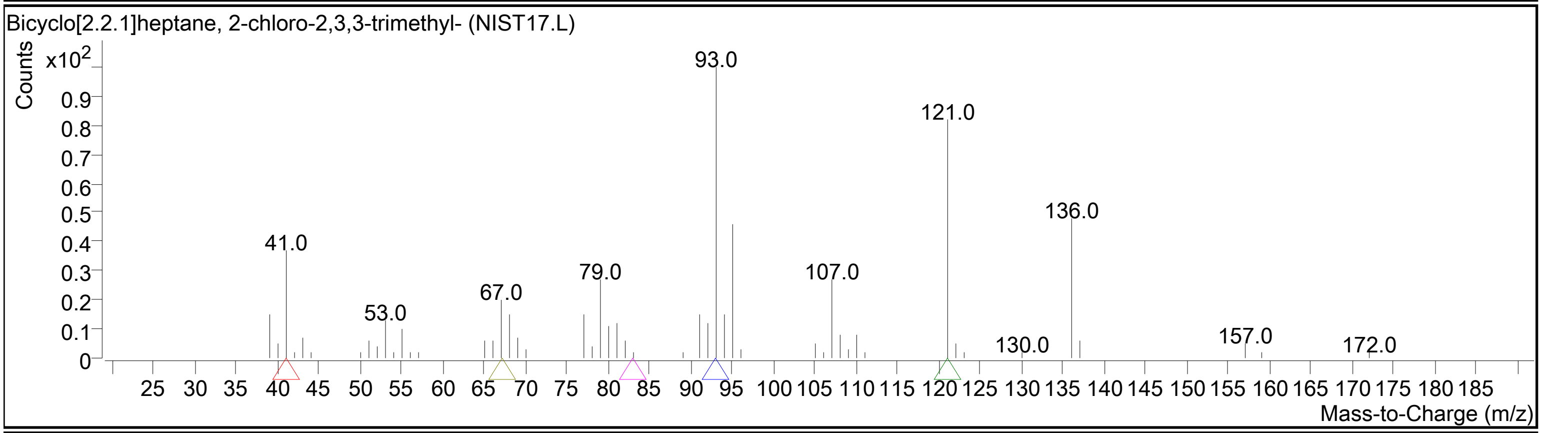

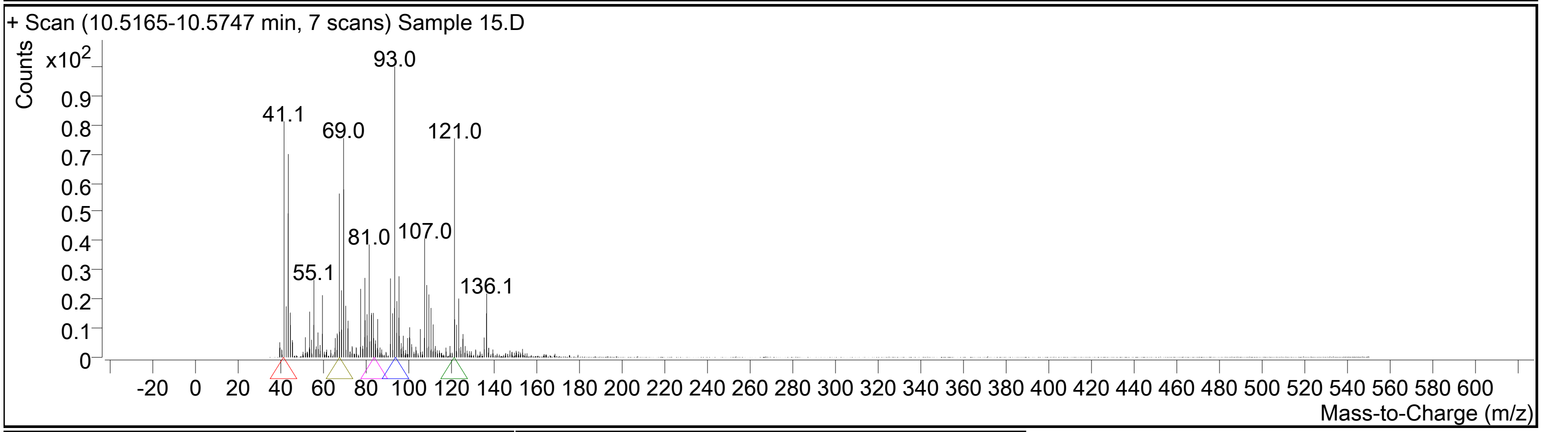

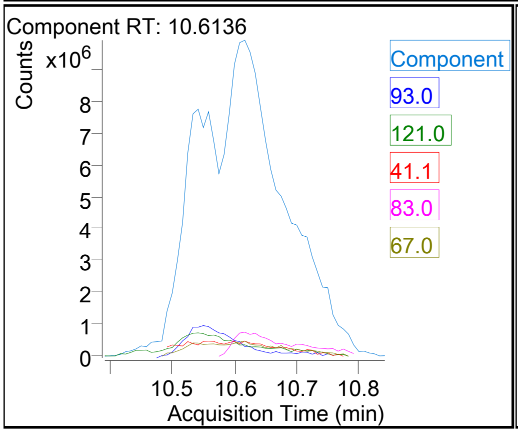

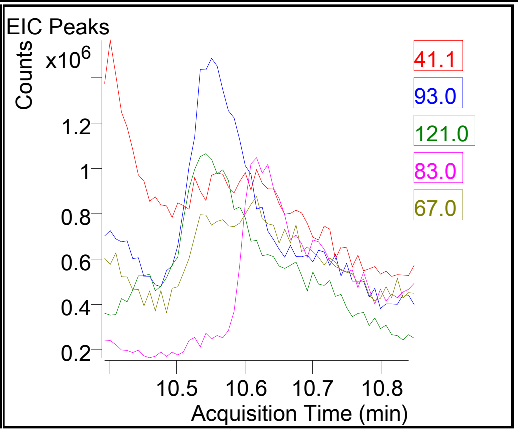

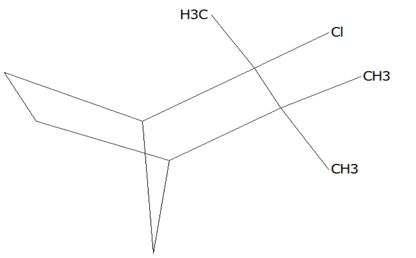

Component RT	Compound Name	Component Area	Match Factor	CAS#	Formula	Estimated Conc.
10.9035	(3E,5E)-2,6-Dimethylocta-3,5,7-trien-2-ol	495867967.9	84.8	206115-88-0	C10H16O	

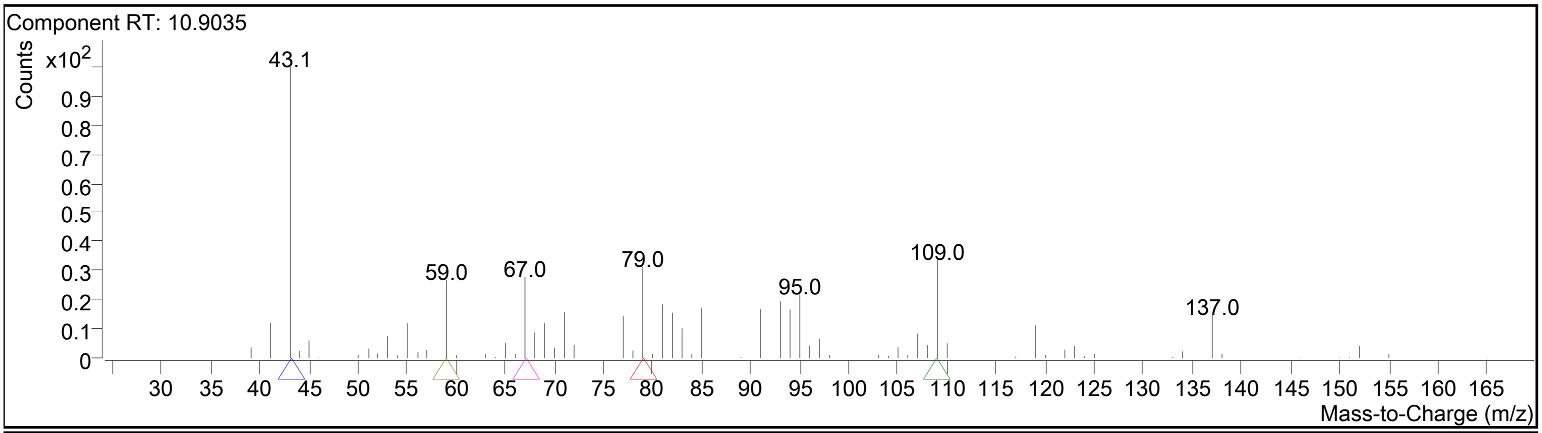

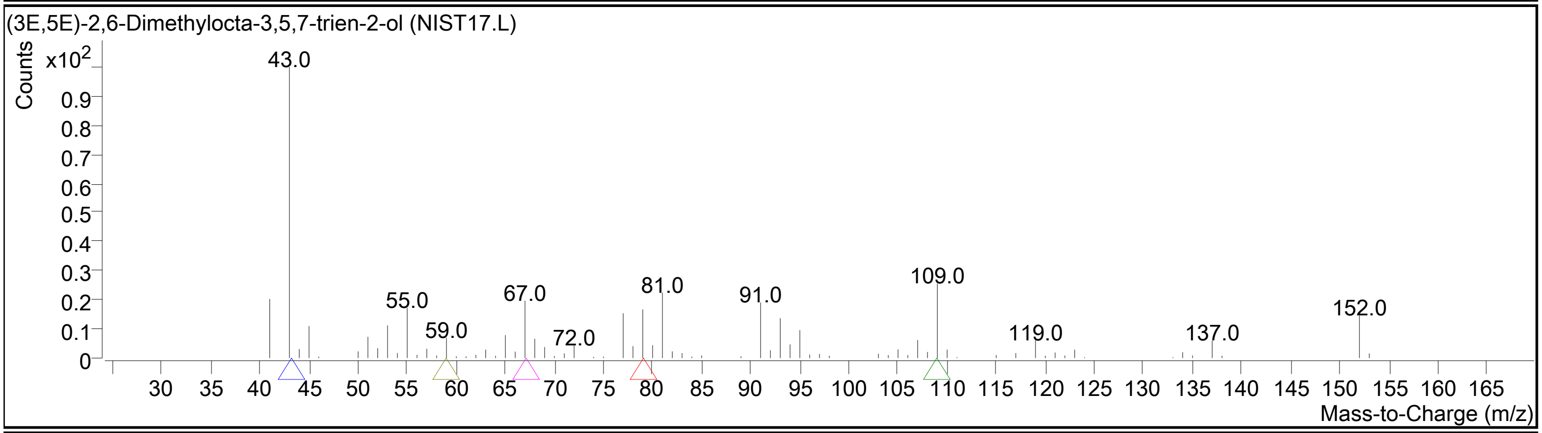

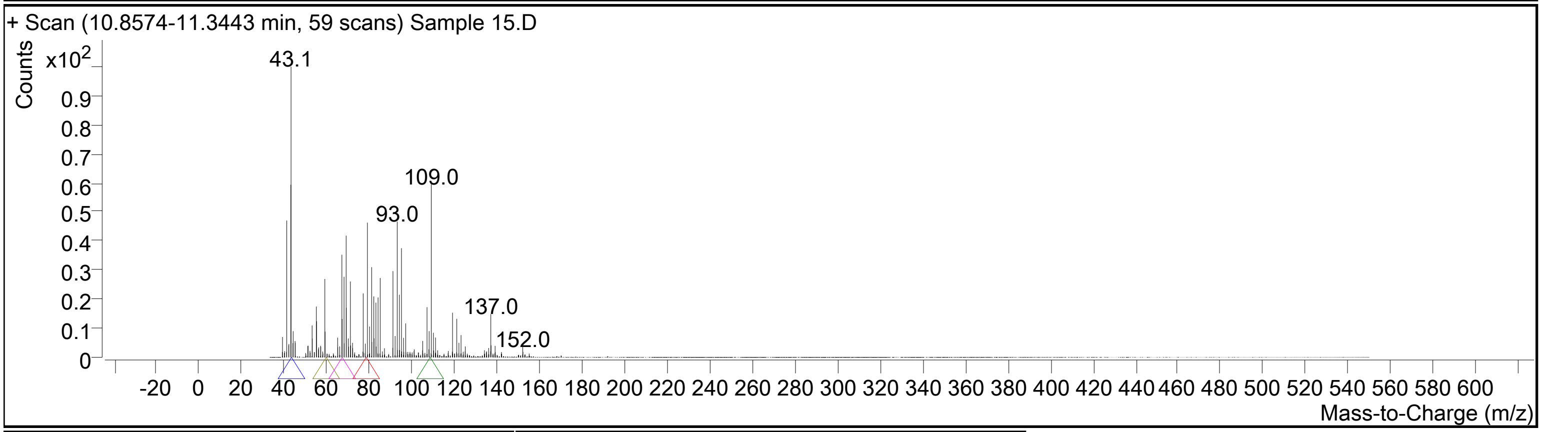

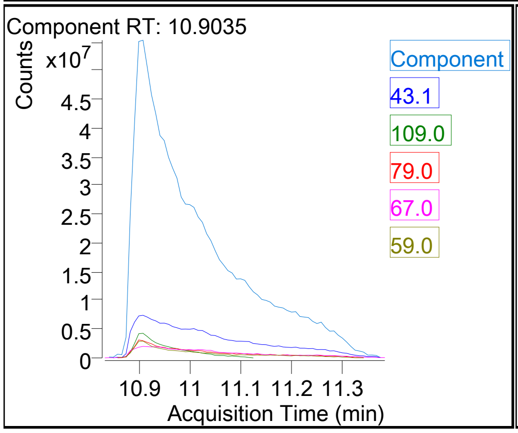

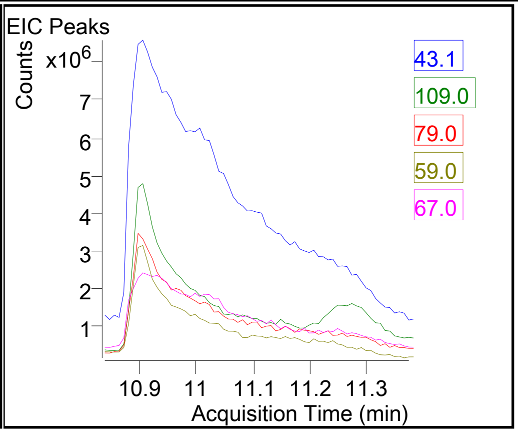

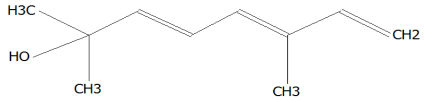

Generated at 12:24 PM on 2/25/2021

Component RT	Compound Name	Component Area	Match Factor	CAS#	Formula	Estimated Conc.
11.6076	Methyleugenol	435789812.2	92.2	93-15-2	C11H14O2	

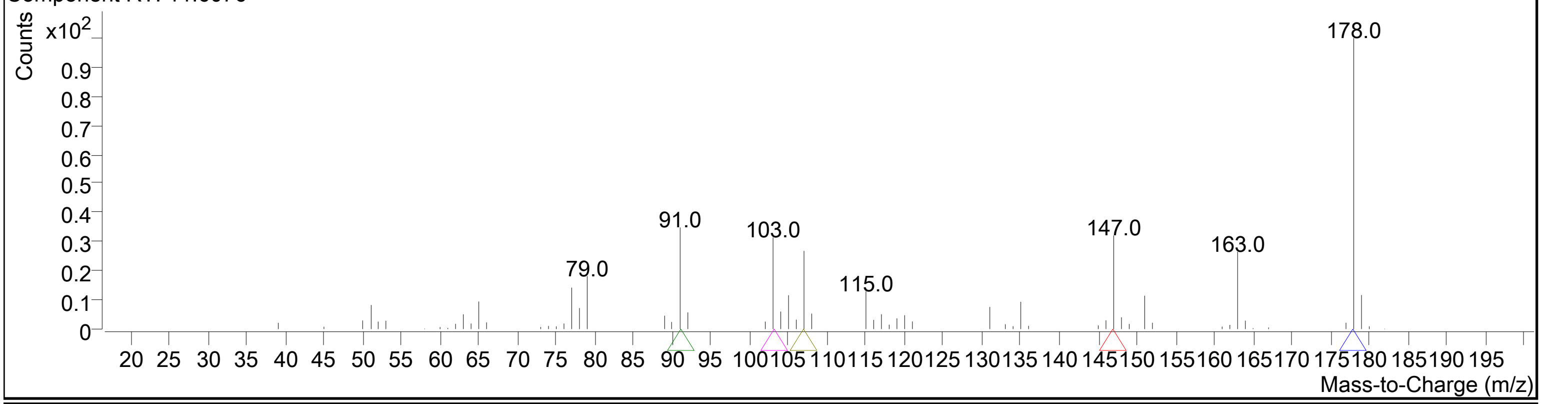

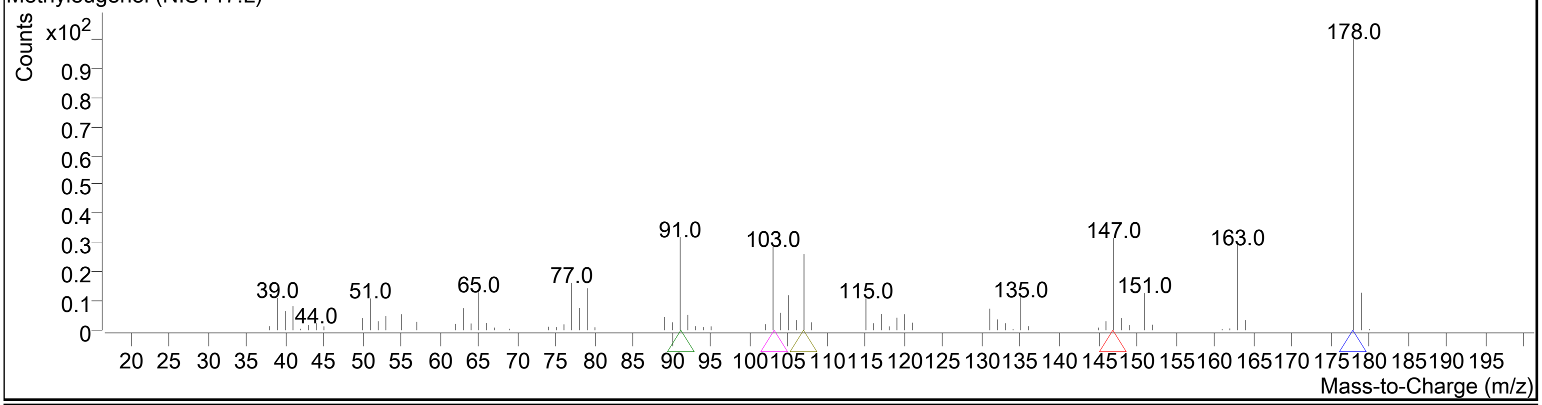

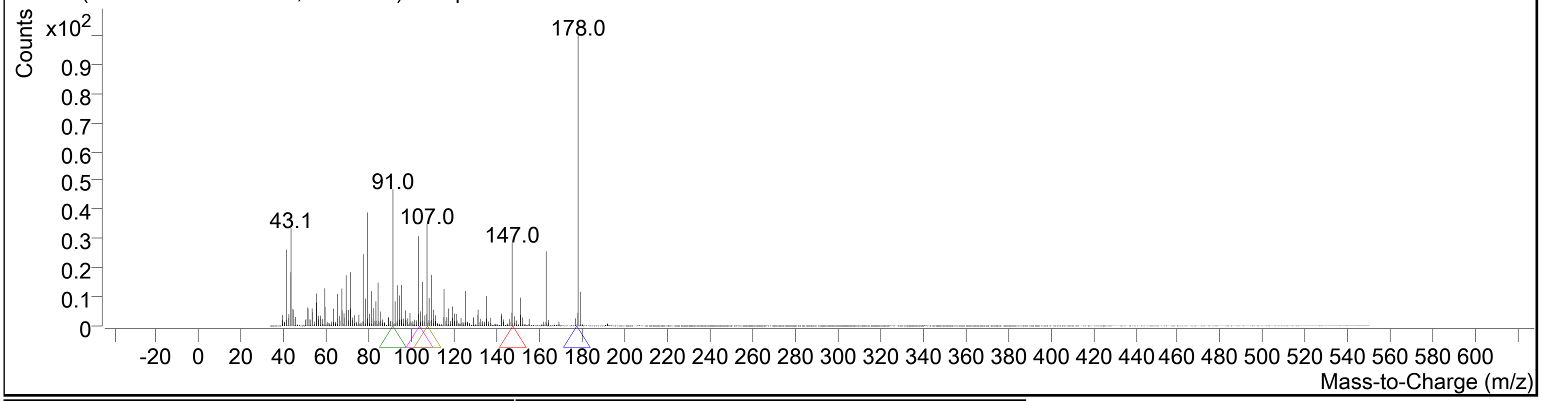

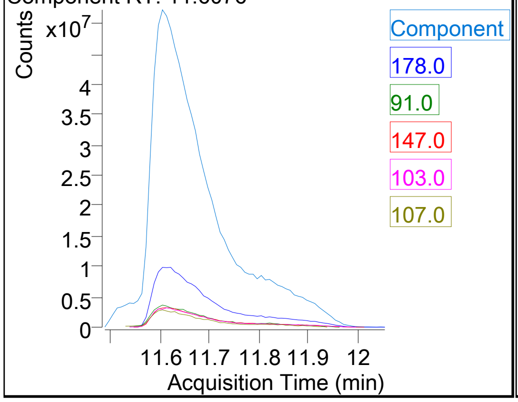

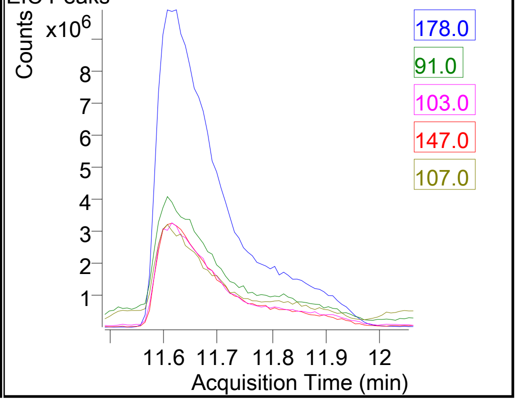

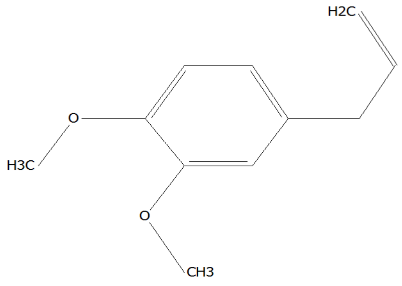

Generated at 12:24 PM on 2/25/2021

Library Search Results - NonTarget Hits with Details

Component RT	Compound Name	Component Area	Match Factor	CAS#	Formula	Estimated Conc.
11.8291	Succinimide	10467697.7	69.3	123-56-8	C4H5NO2	

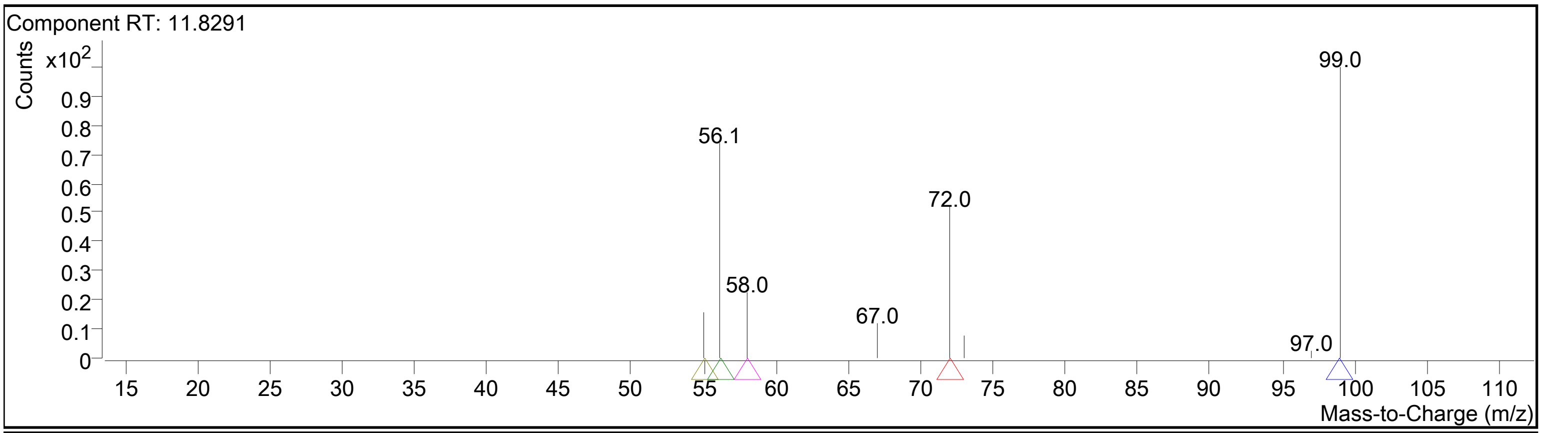

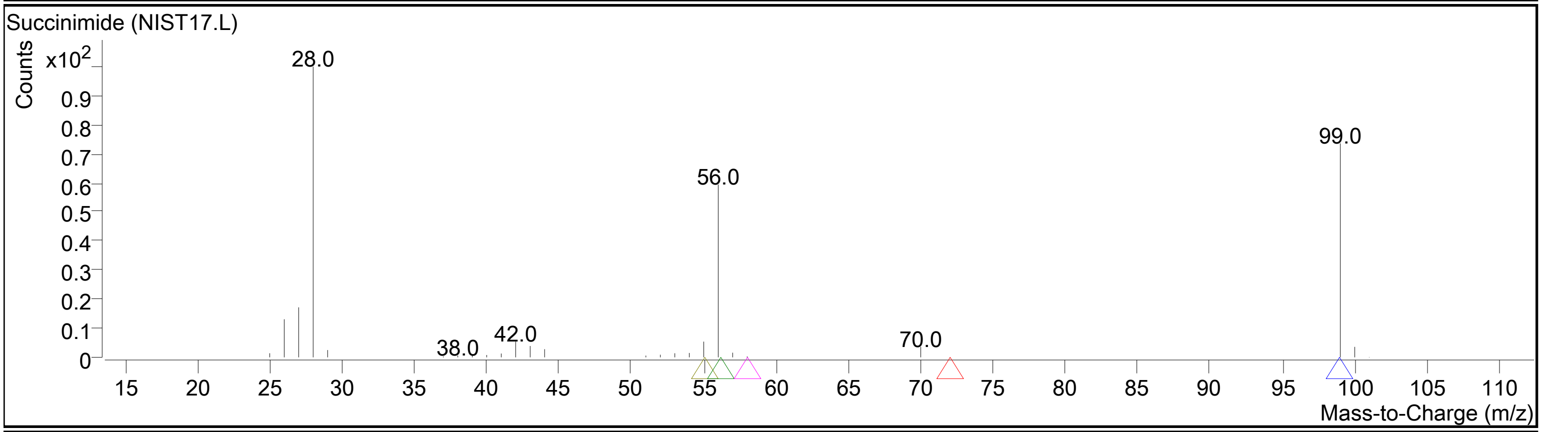

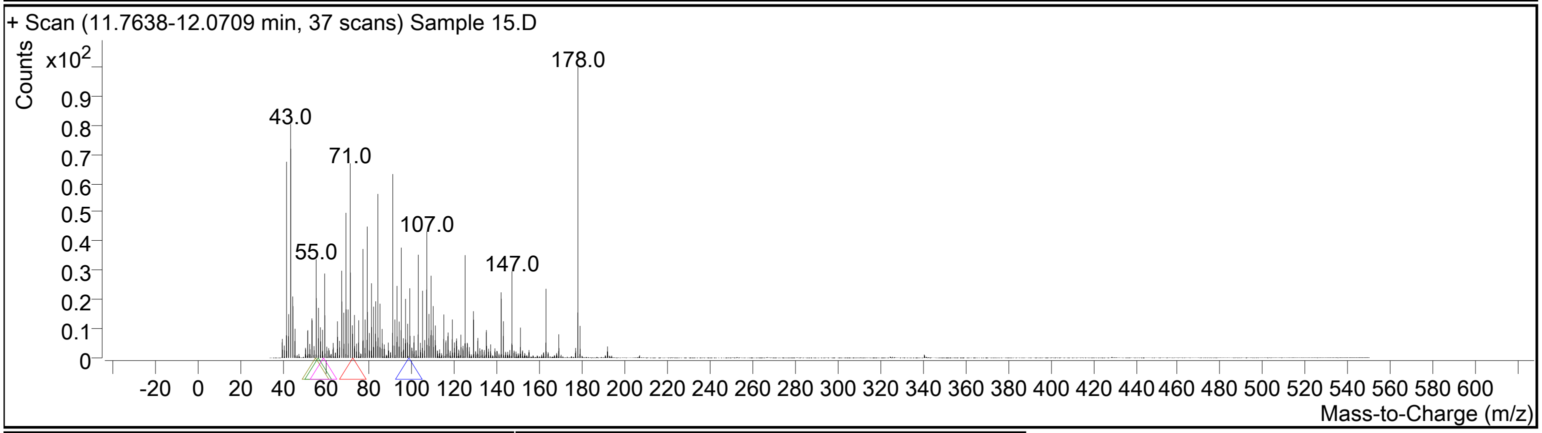

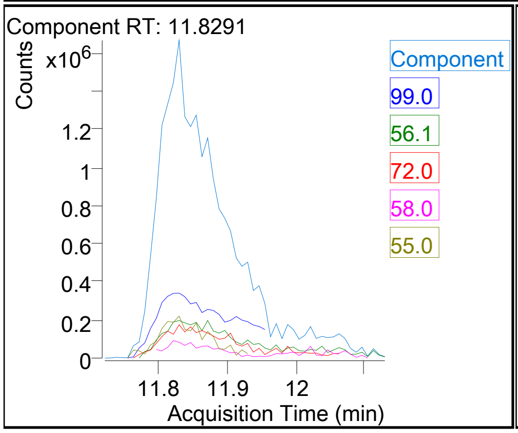

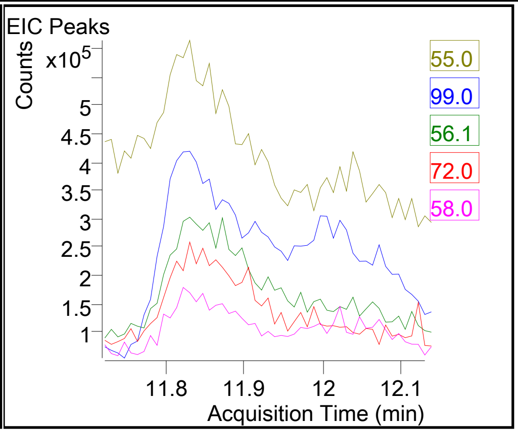

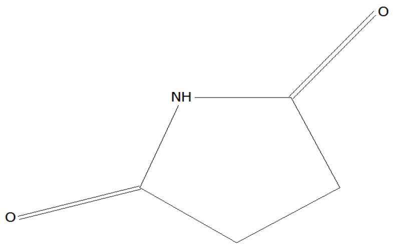

Component RT	Compound Name	Component Area	Match Factor	CAS#	Formula	Estimated Conc.
11.8375	Cyclopropanecarboxamide, N-tetrahydrofurfuryl-	38050668.1	68.2	1000307-18-4	C9H15NO2	

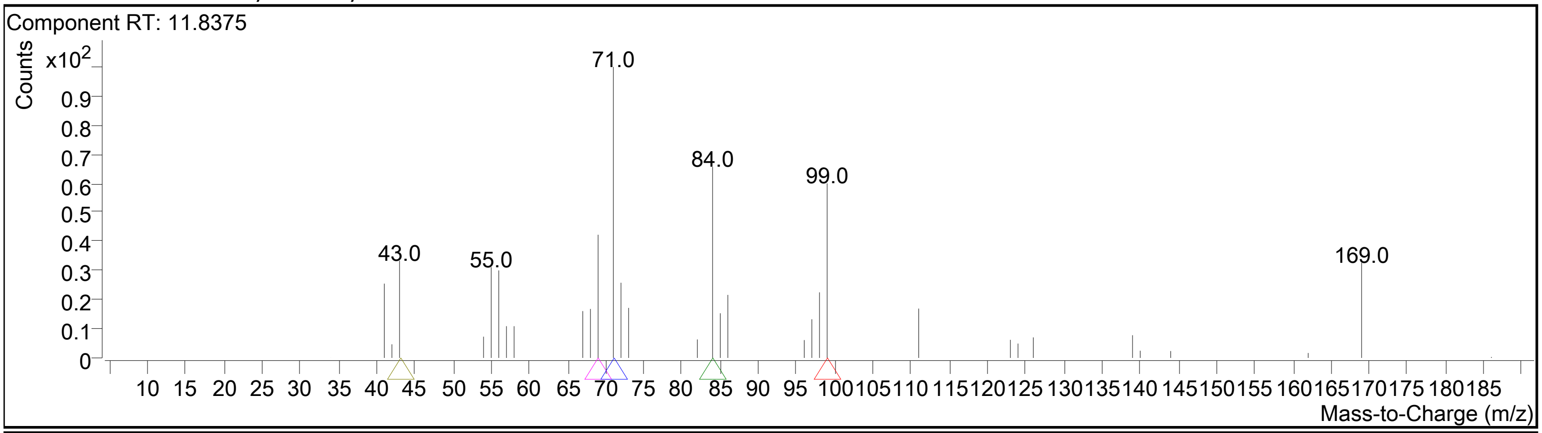

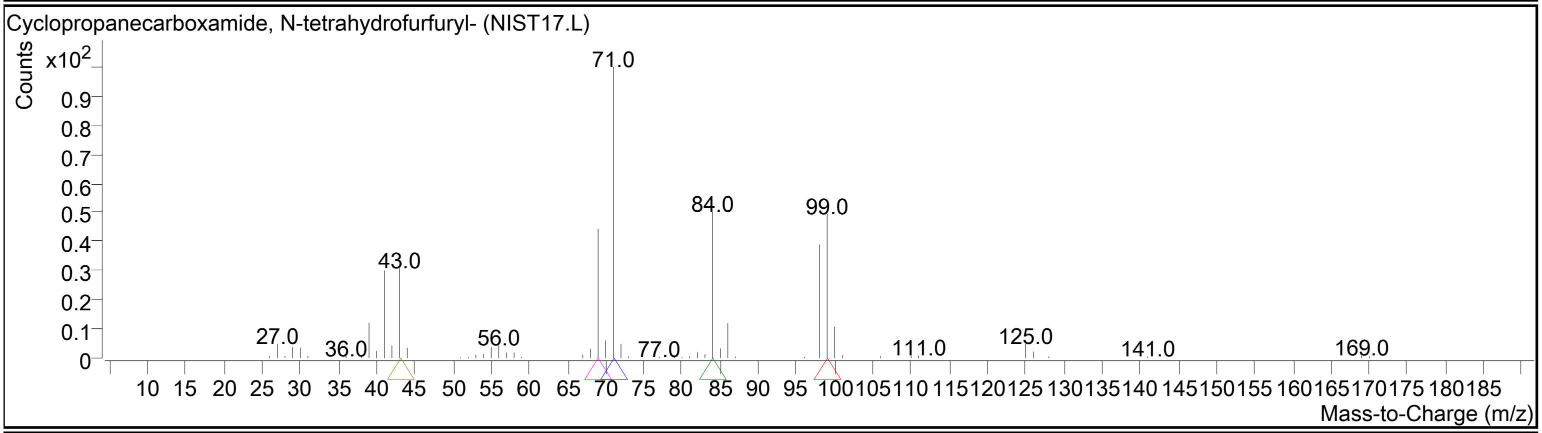

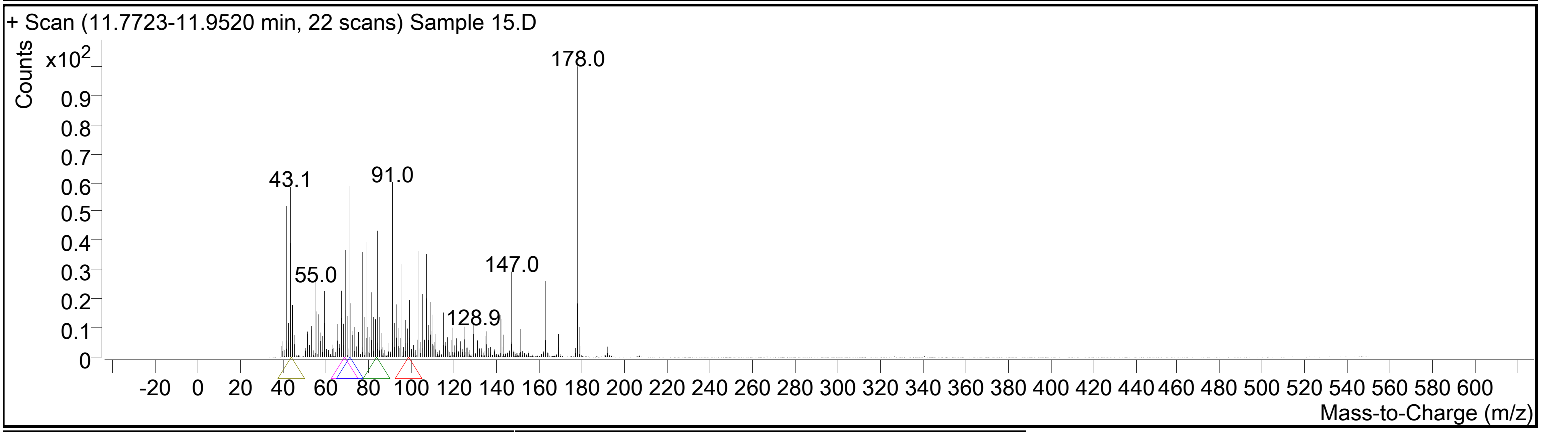

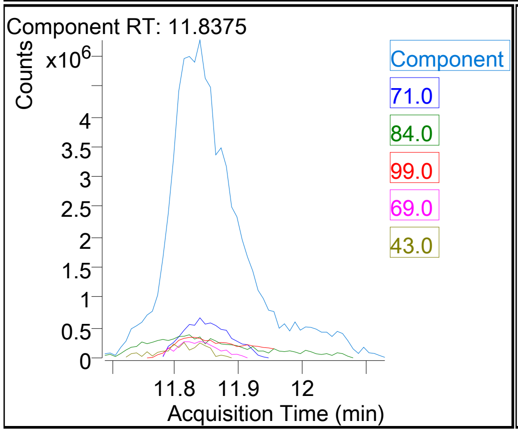

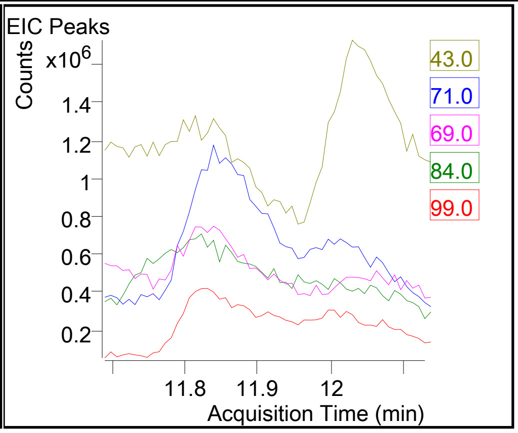

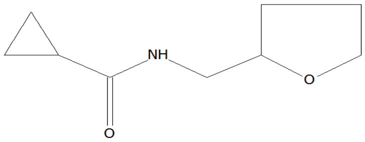

Component RT	Compound Name	Component Area	Match Factor	CAS#	Formula	Estimated Conc.
12.0535	Heptyl methyl ethylphosphonate	18077542.4	68.0	169662-35-5	C10H23O3P	

Component RT: 12.0535

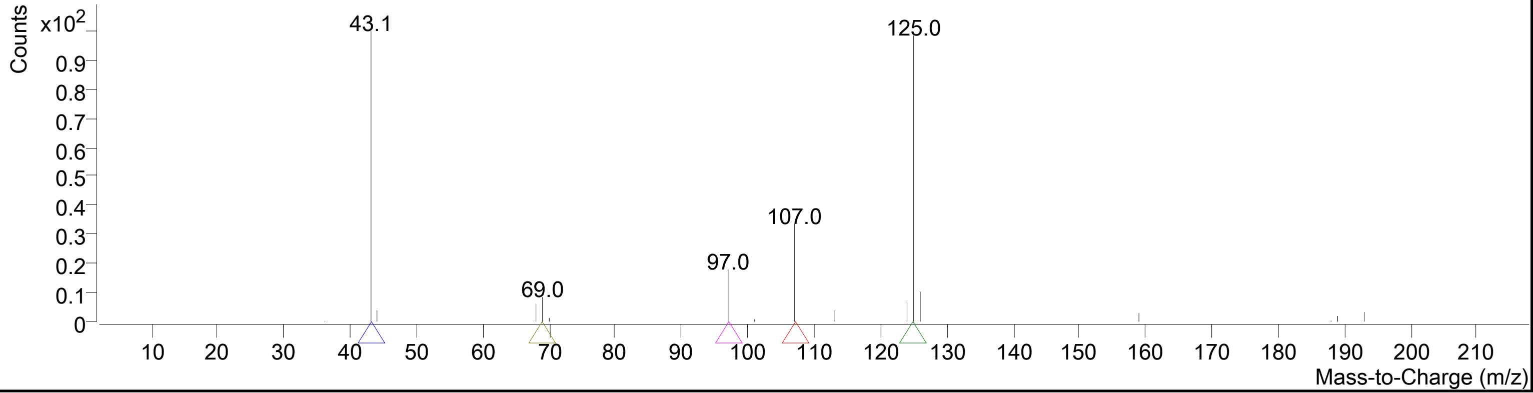

Heptyl methyl ethylphosphonate (NIST17.L)

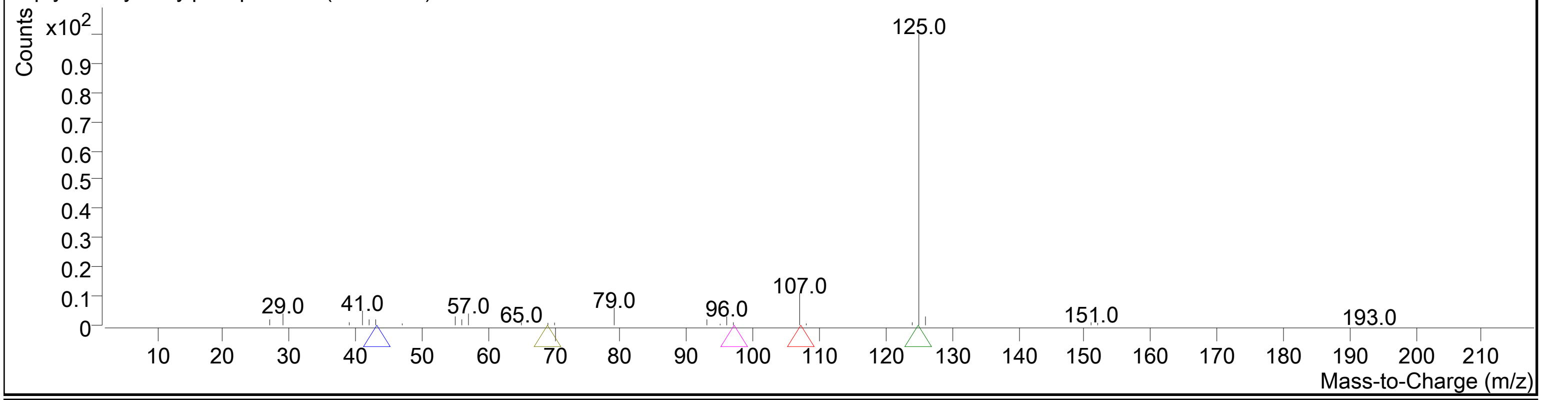

+ Scan (12.0299-12.1047 min, 9 scans) Sample 15.D

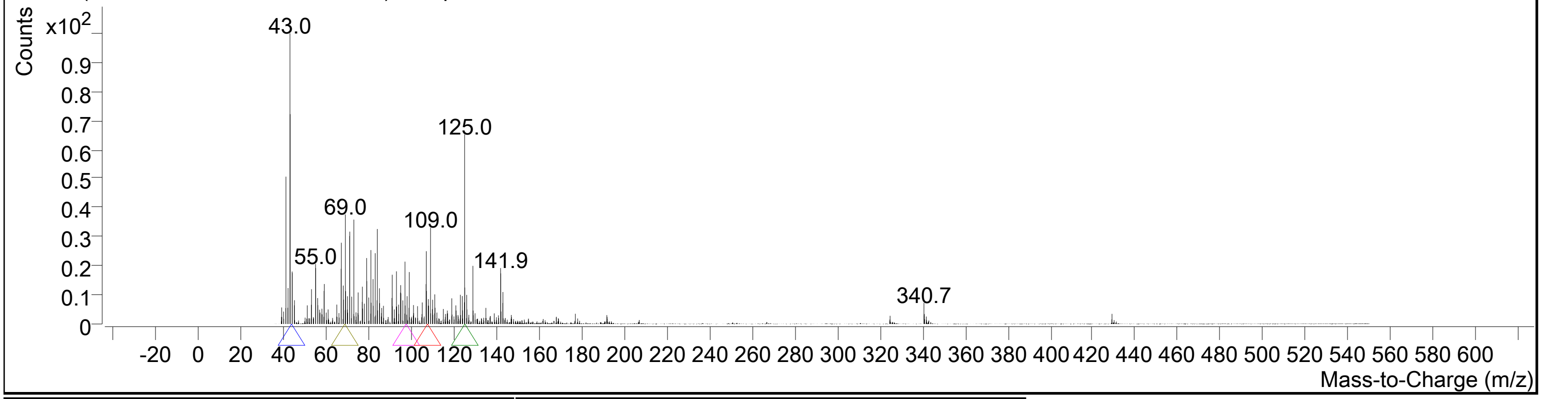

Component RT: 12.0535

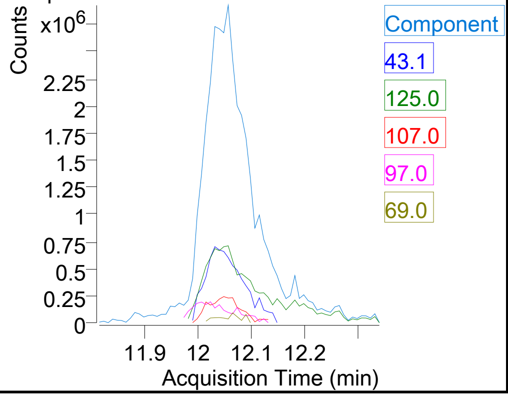

EIC Peaks

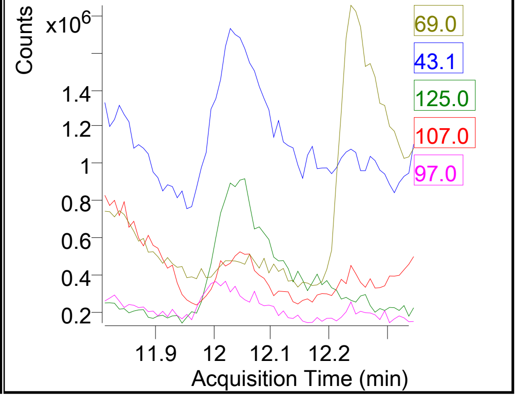

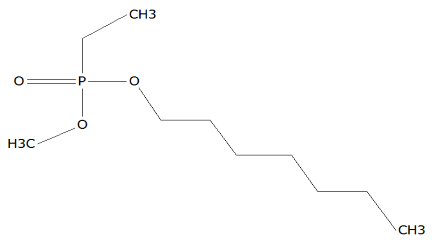

Agilent Technologies

Component RT	Compound Name	Component Area	Match Factor	CAS#	Formula	Estimated Conc.
12.2308	Imidazo[4,5-d]imidazole, 1,6-dihydro-	23364294.9	72.4	35369-36-9	C4H4N4	

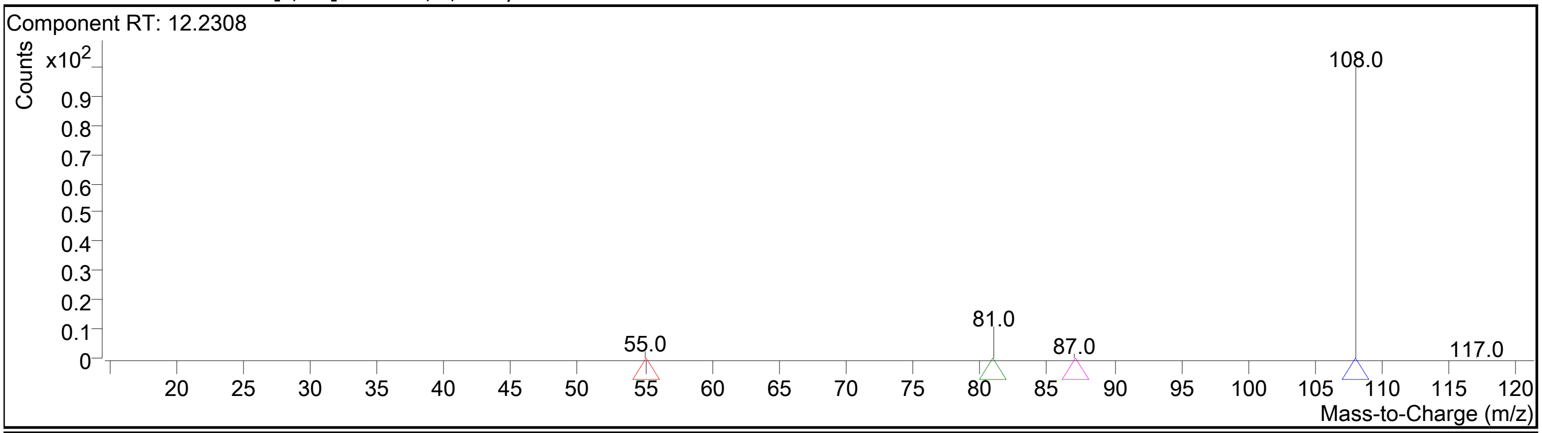

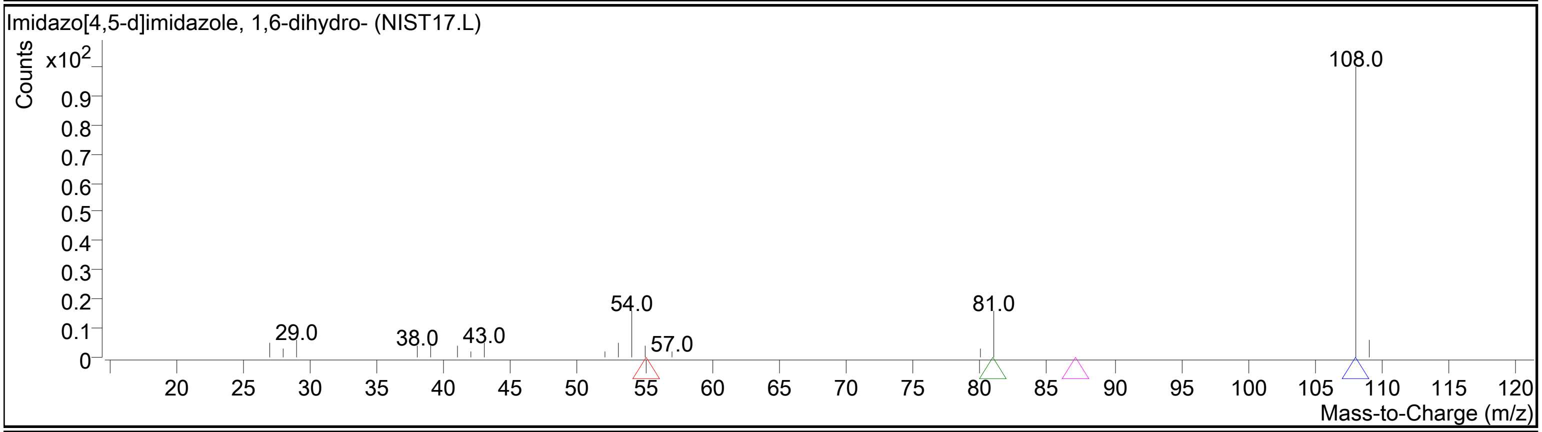

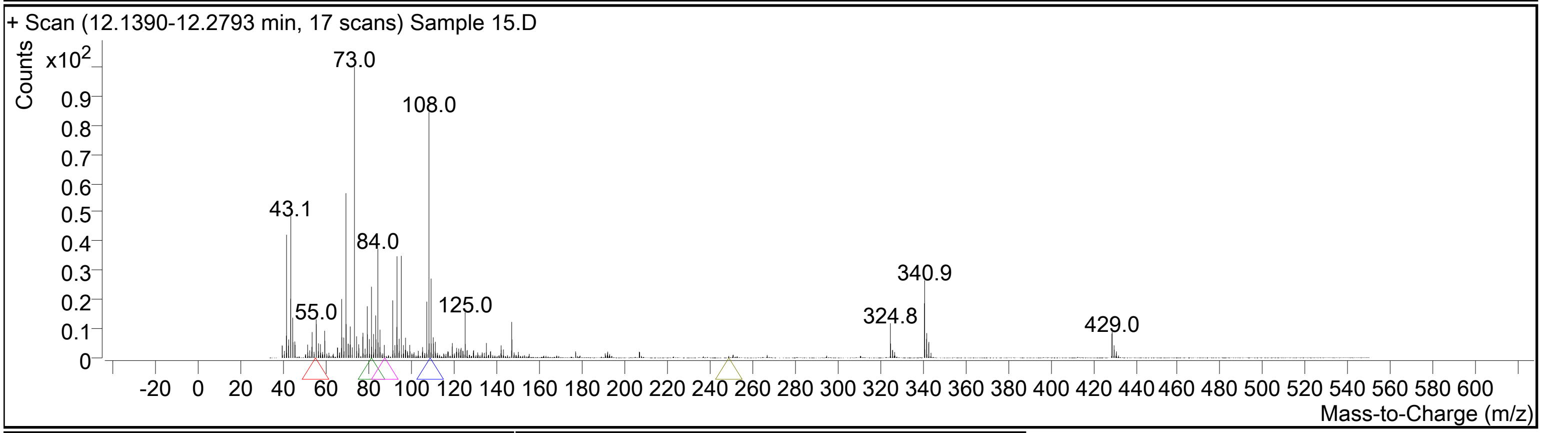

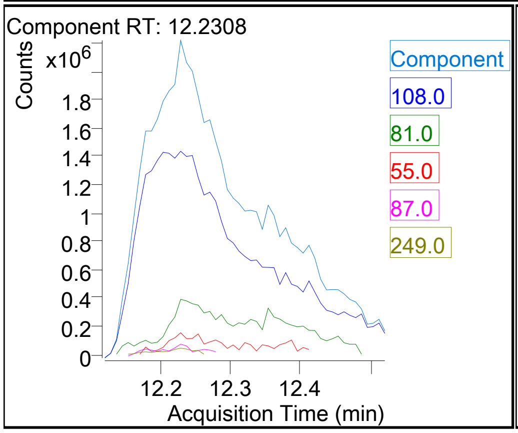

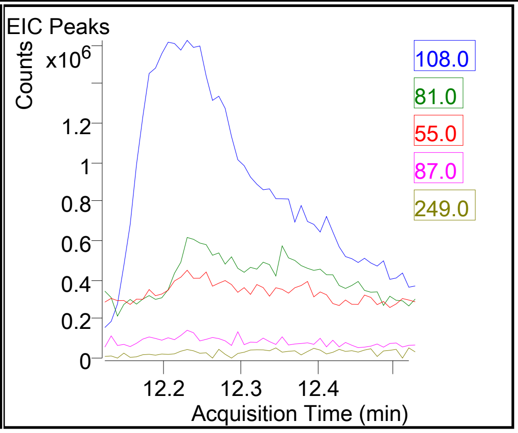

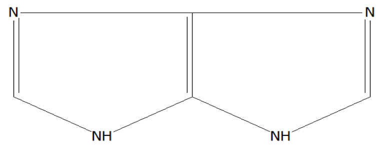

Component RT	Compound Name	Component Area	Match Factor	CAS#	Formula	Estimated Conc.
12.2405	3,7-Nonadien-2-one, 8-methyl-, (E)-	40152413.4	71.1	35408-14-1	C10H16O	

Component RT: 12.2405

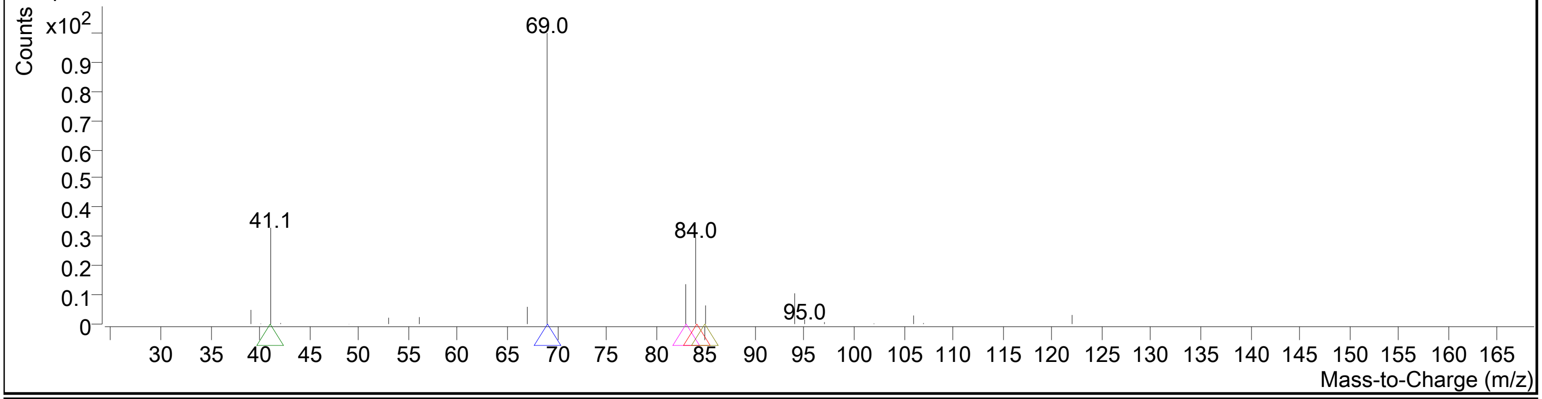

3,7-Nonadien-2-one, 8-methyl-, (E)- (NIST17.L)

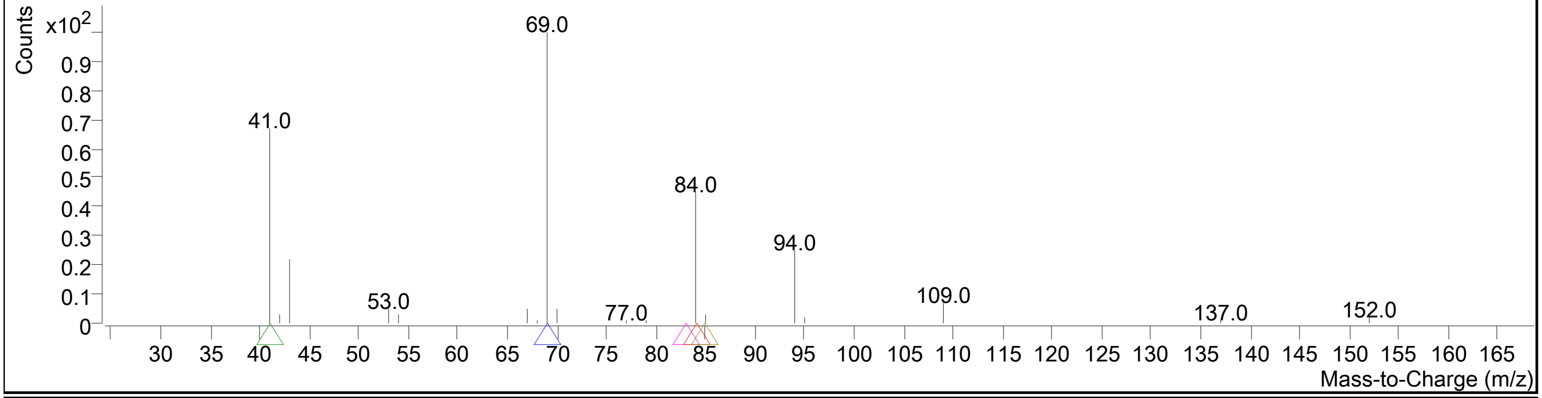

+ Scan (12.2006-12.4373 min, 29 scans) Sample 15.D

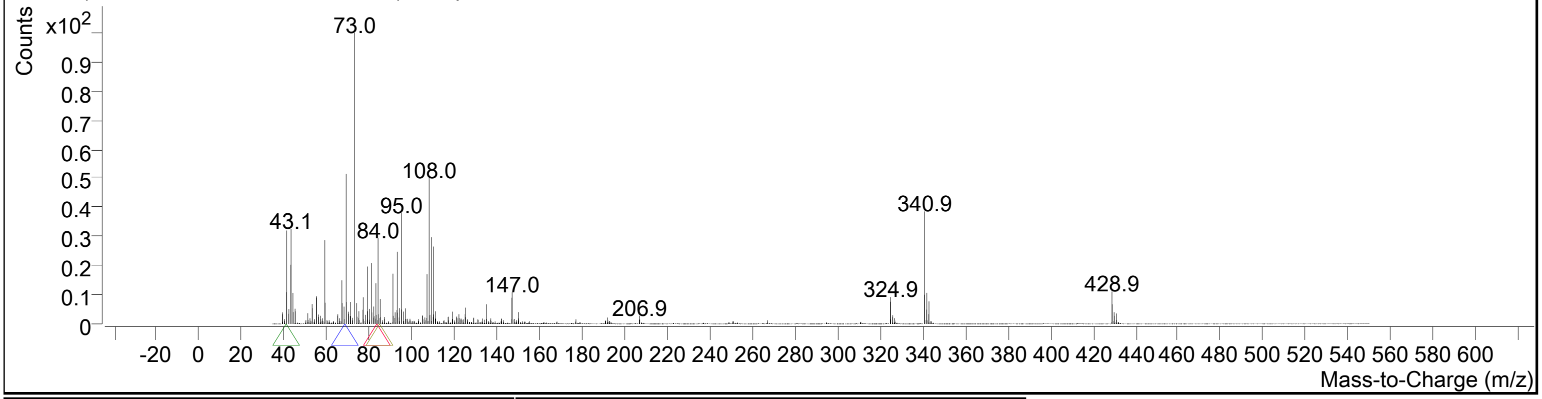

Component RT: 12.2405

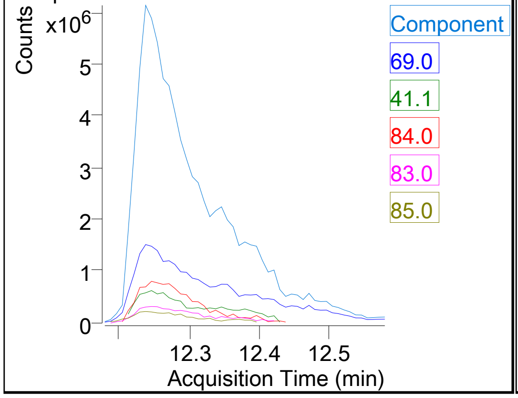

EIC Peaks

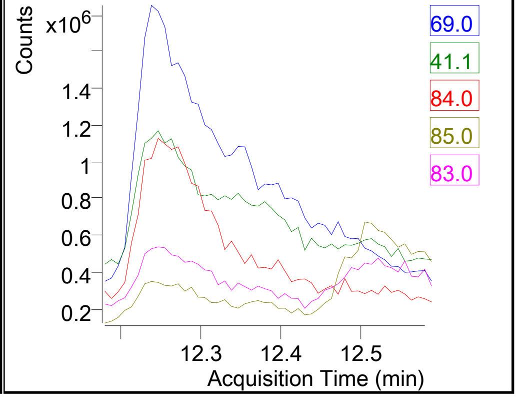

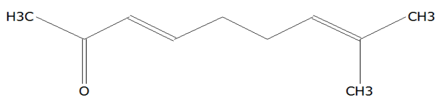

Library Search Results - NonTarget Hits with Details

Component RT	Compound Name	Component Area	Match Factor	CAS#	Formula	Estimated Conc.
12.2464	2-Pyrrolidinone, 5-(ethoxymethyl)-	13450746.9	65.9	64749-67-3	C7H13NO2	

Component RT: 12.2464

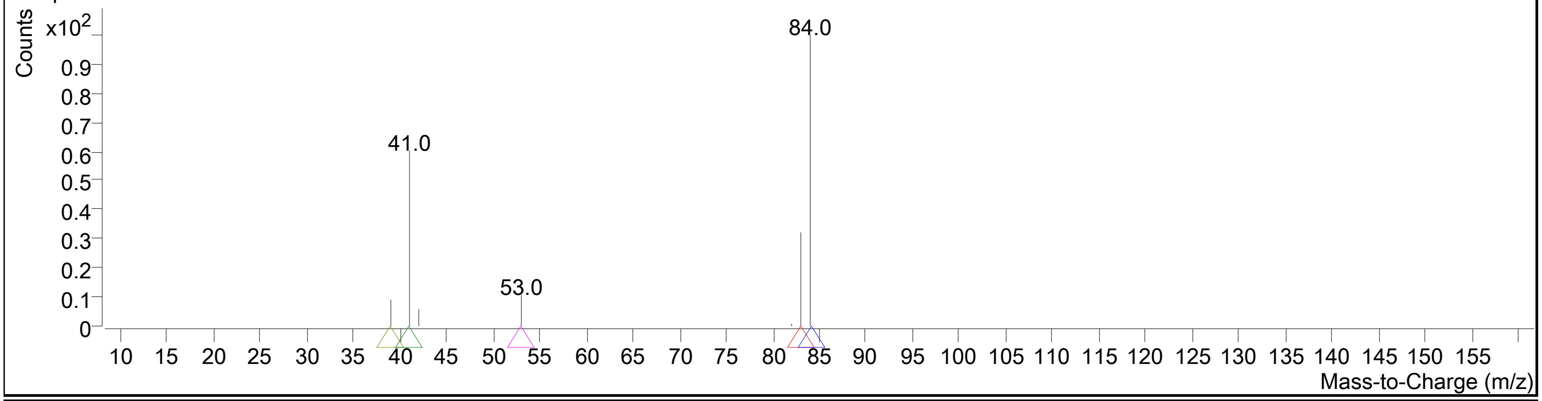

2-Pyrrolidinone, 5-(ethoxymethyl)- (NIST17.L)

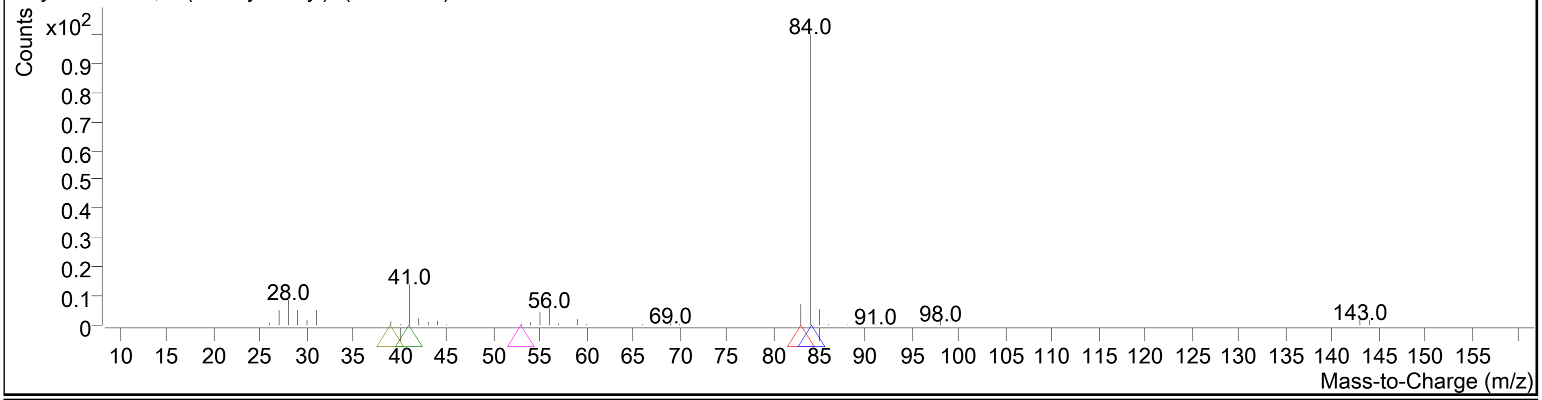

+ Scan (12.2006-12.4373 min, 29 scans) Sample 15.D

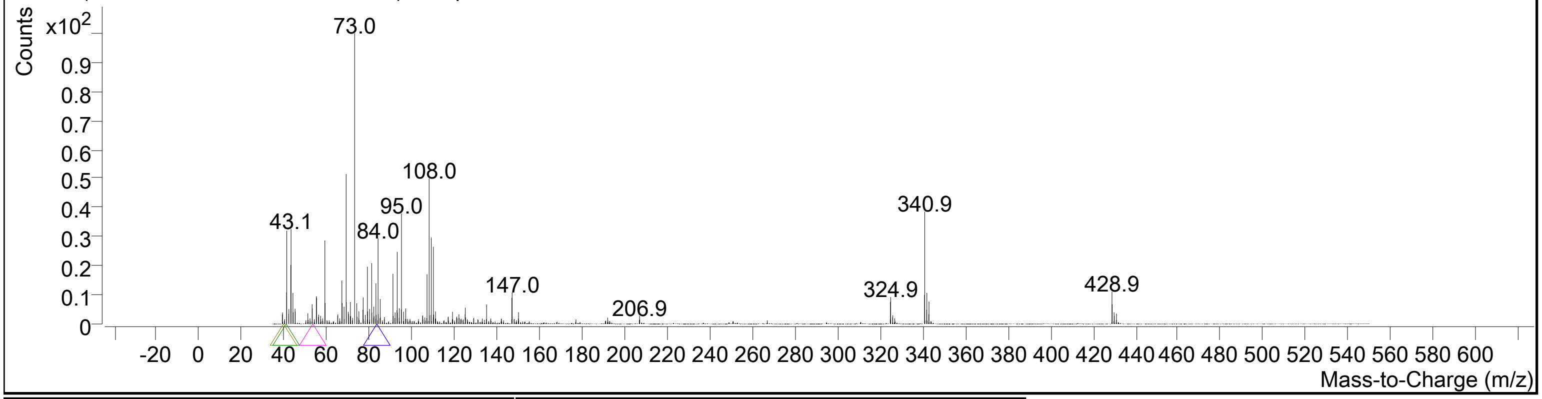

Component RT: 12.2464

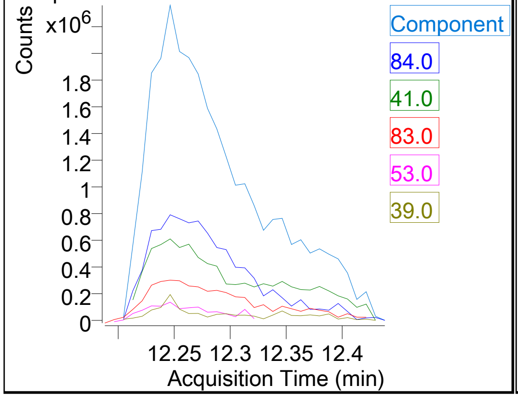

EIC Peaks

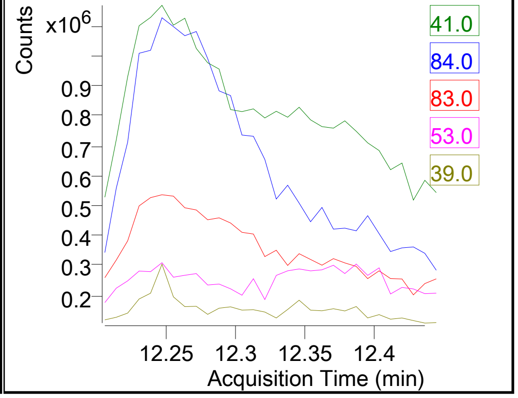

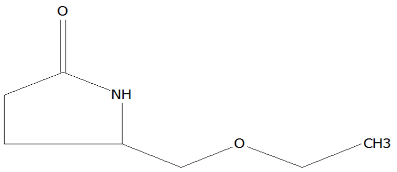

Component RT	Compound Name	Component Area	Match Factor	CAS#	Formula	Estimated Conc.
12.3542	Ethane, 1,2-dibromo-	8408546.6	65.1	106-93-4	C2H4Br2	

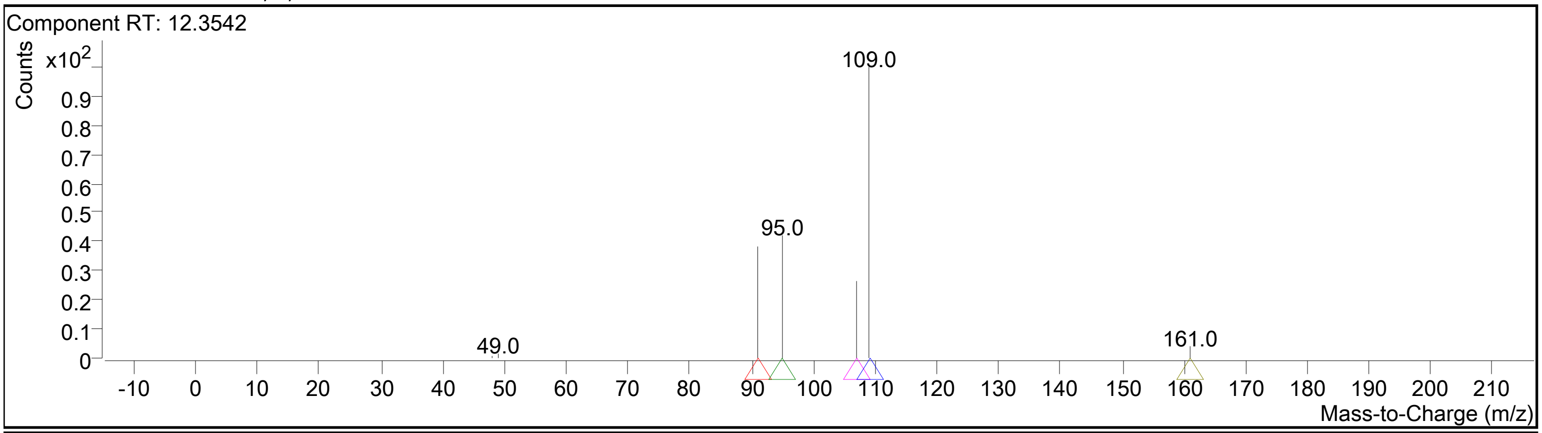

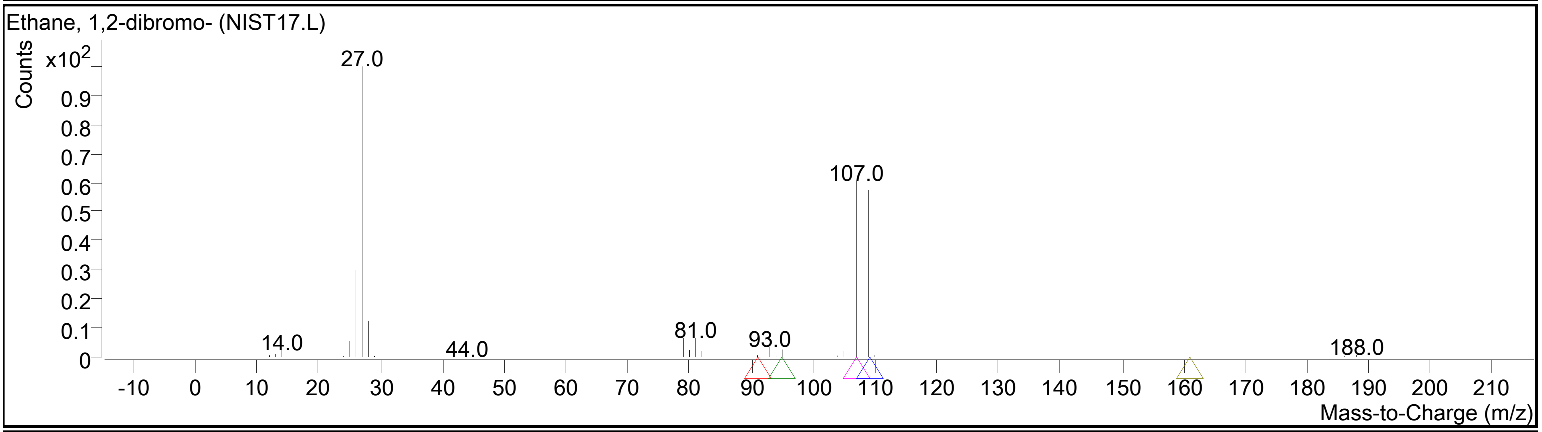

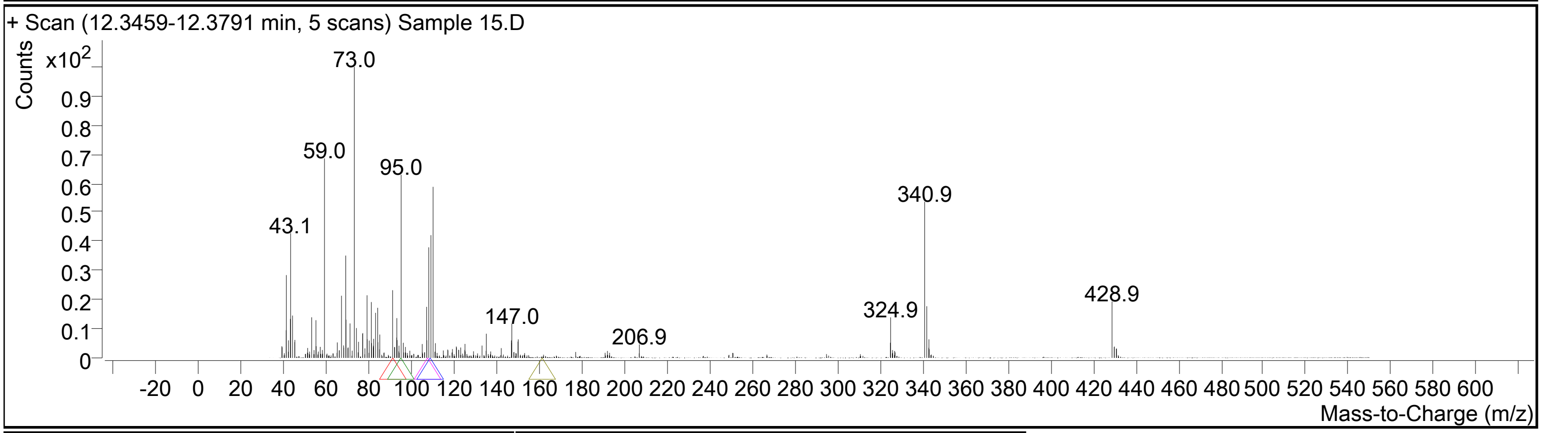

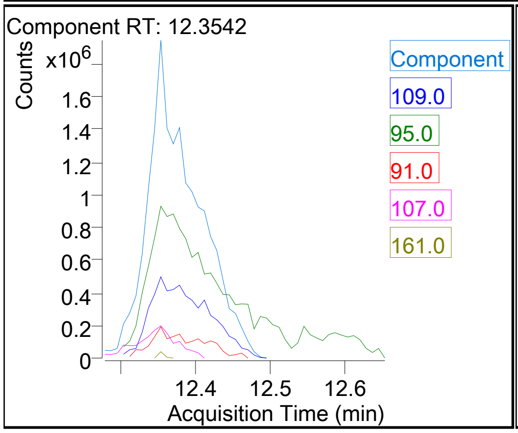

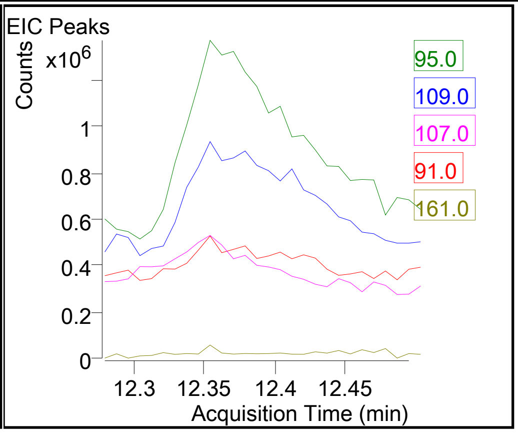

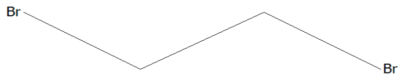

Component RT	Compound Name	Component Area	Match Factor	CAS#	Formula	Estimated Conc.
12.3636	Formamide, N-methyl-	9211691.7	66.1	123-39-7	C2H5NO	

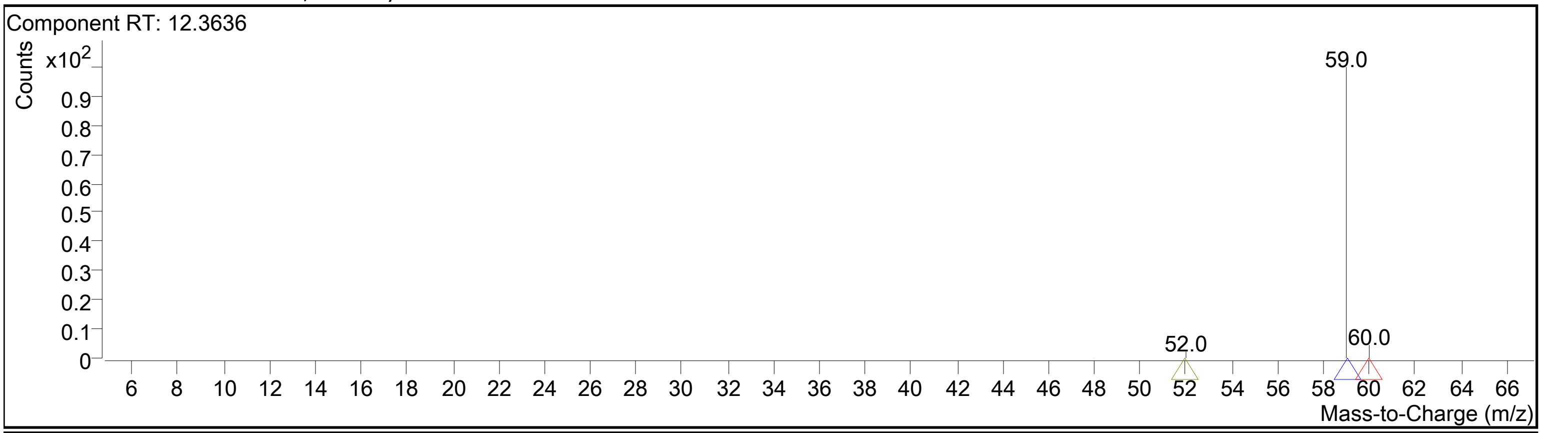

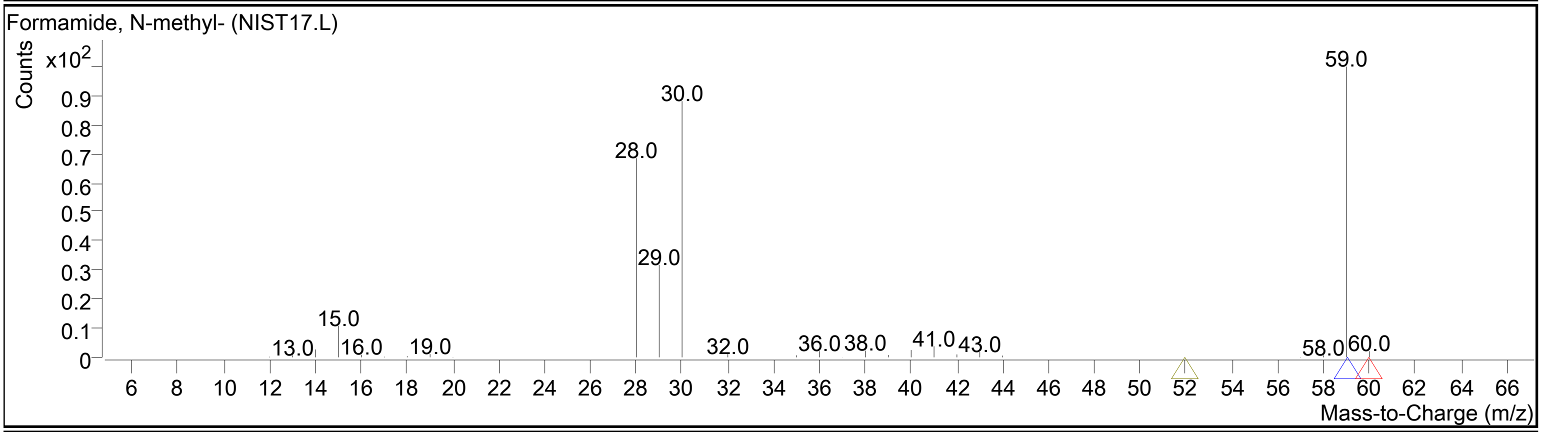

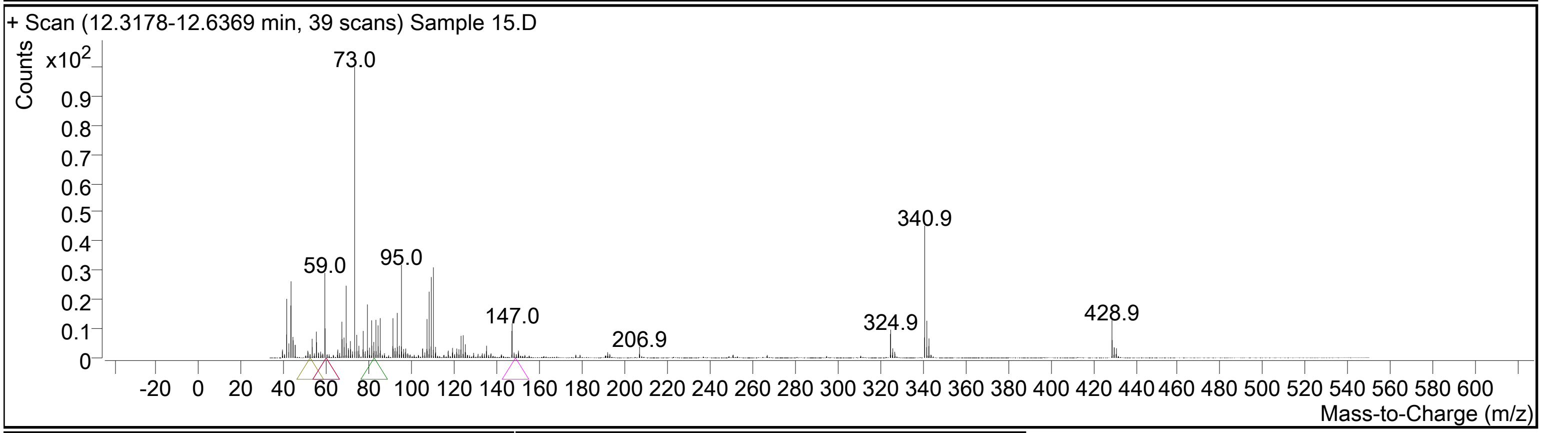

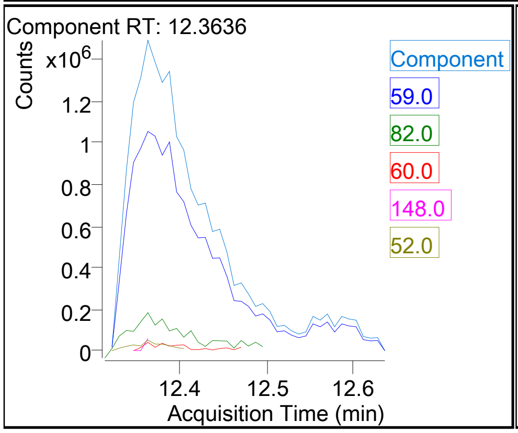

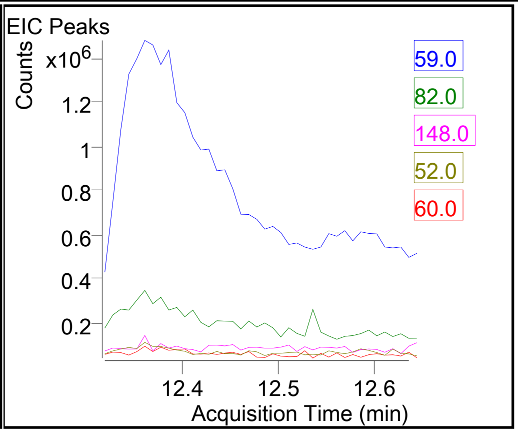

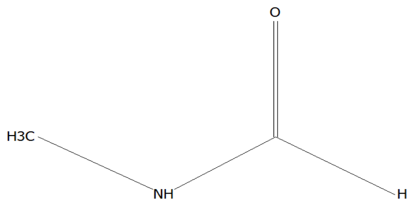

Component RT	Compound Name	Component Area	Match Factor	CAS#	Formula	Estimated Conc.
12.3685	8-Hydroxycarvotanacetone	36992552.9	73.8	7712-46-1	C10H16O2	

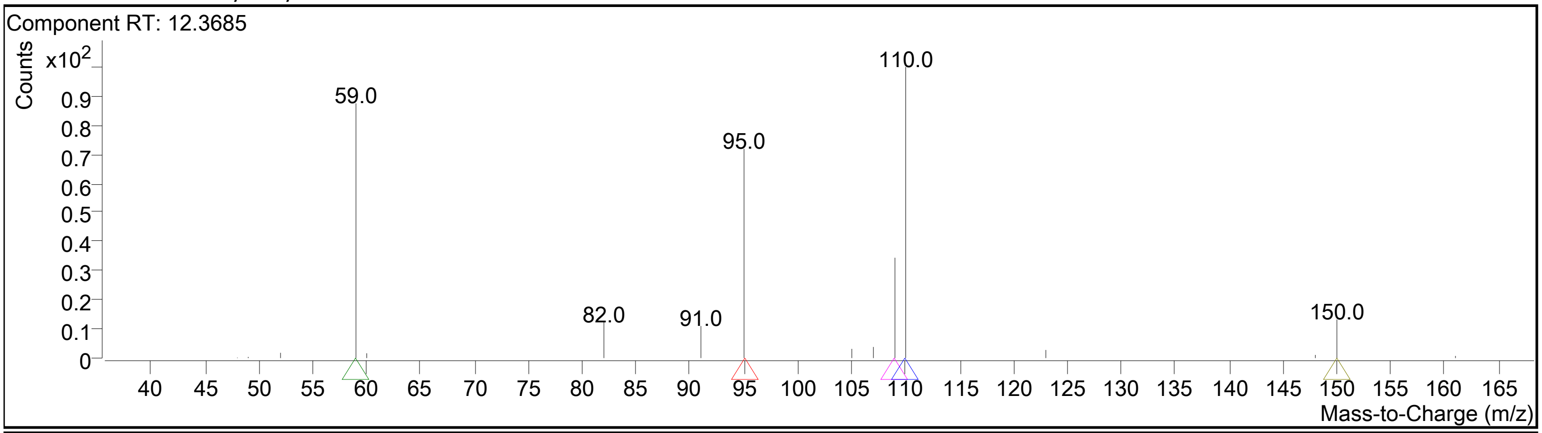

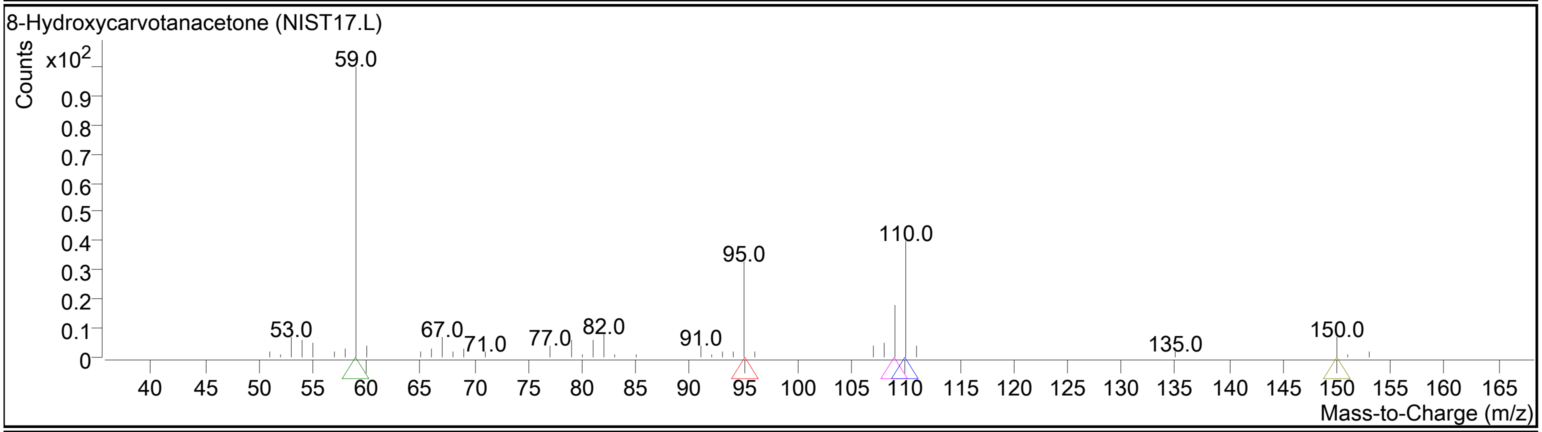

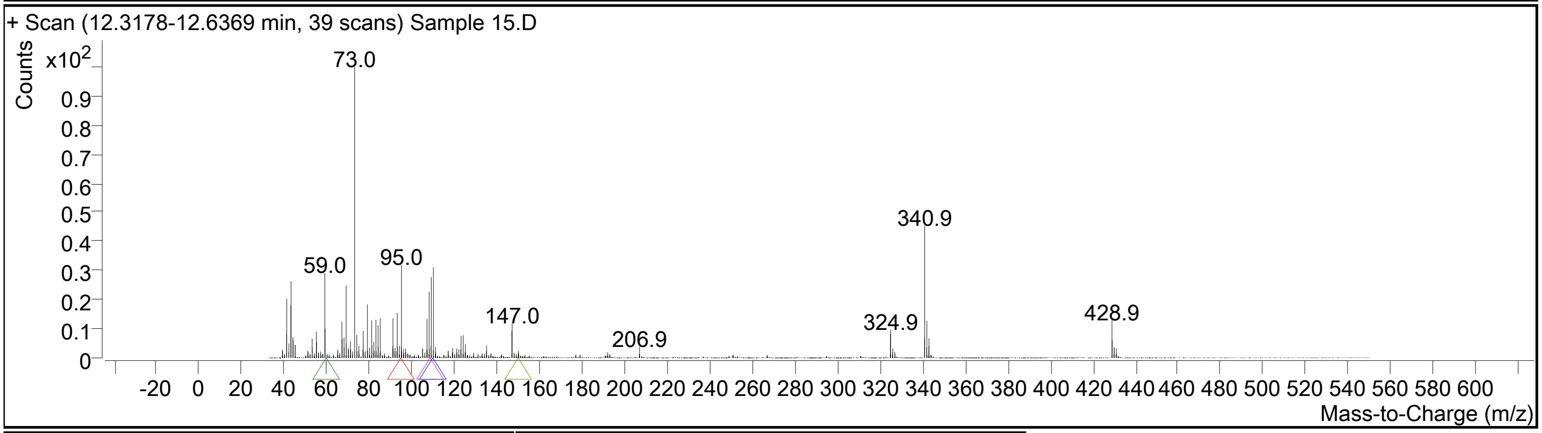

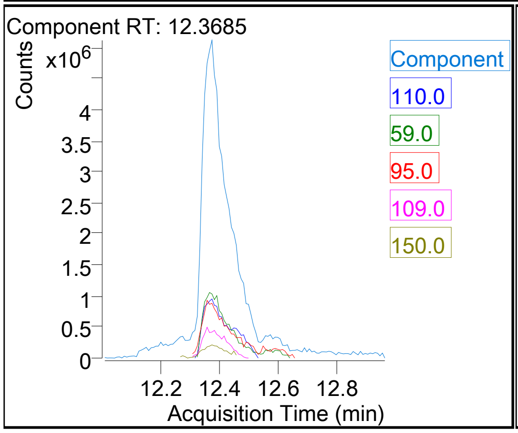

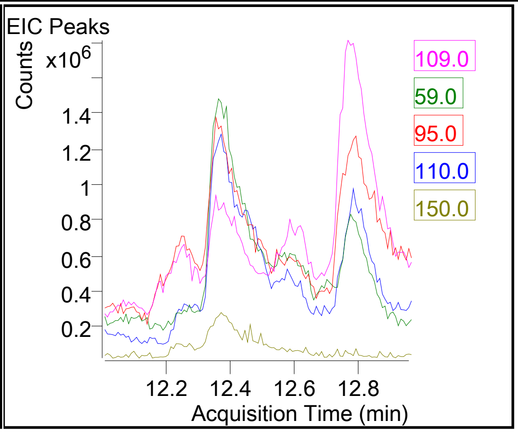

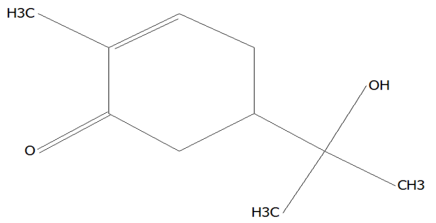

Library Search Results - NonTarget Hits with Details

Agilent Technologies

Component RT	Compound Name	Component Area	Match Factor	CAS#	Formula	Estimated Conc.
12.7619	2,6-Octadiene-1,8-diol, 2,6-dimethyl-	585937479.7	85.3	26489-17-8	C10H18O2	

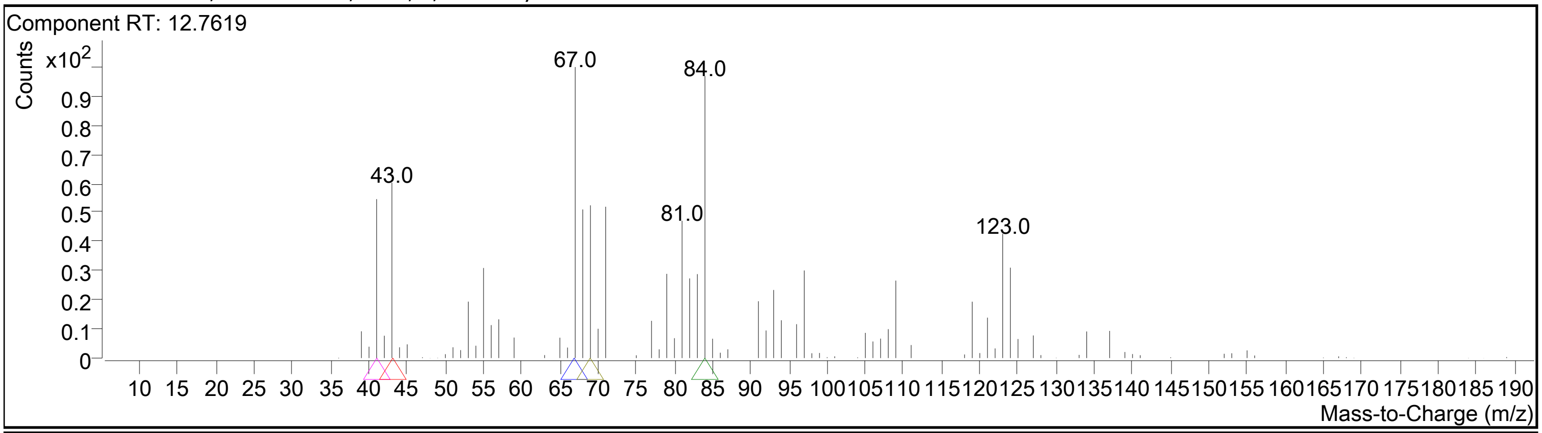

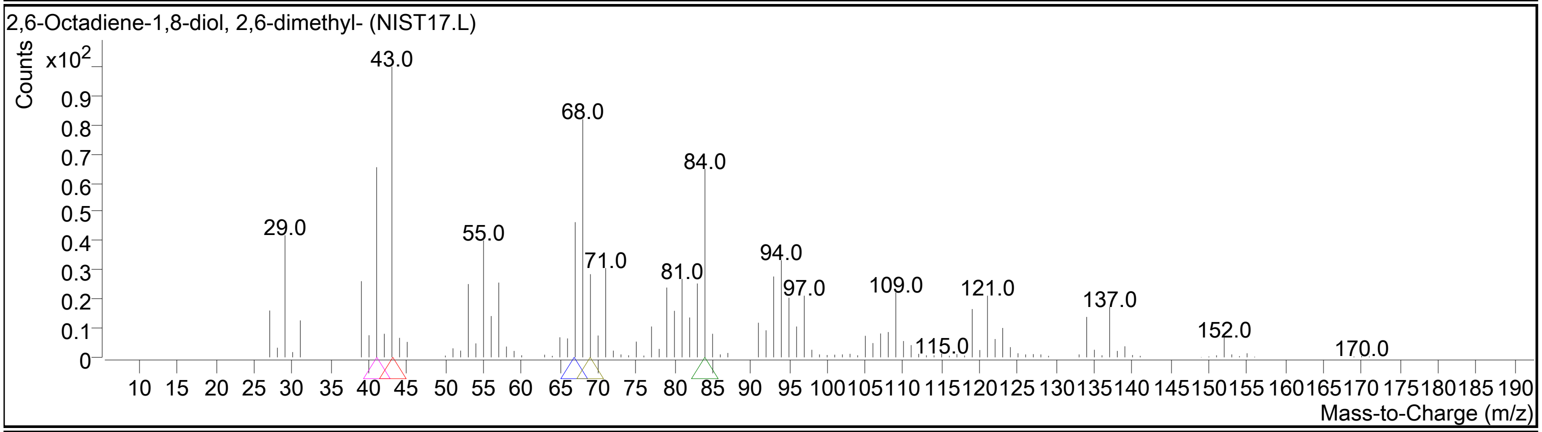

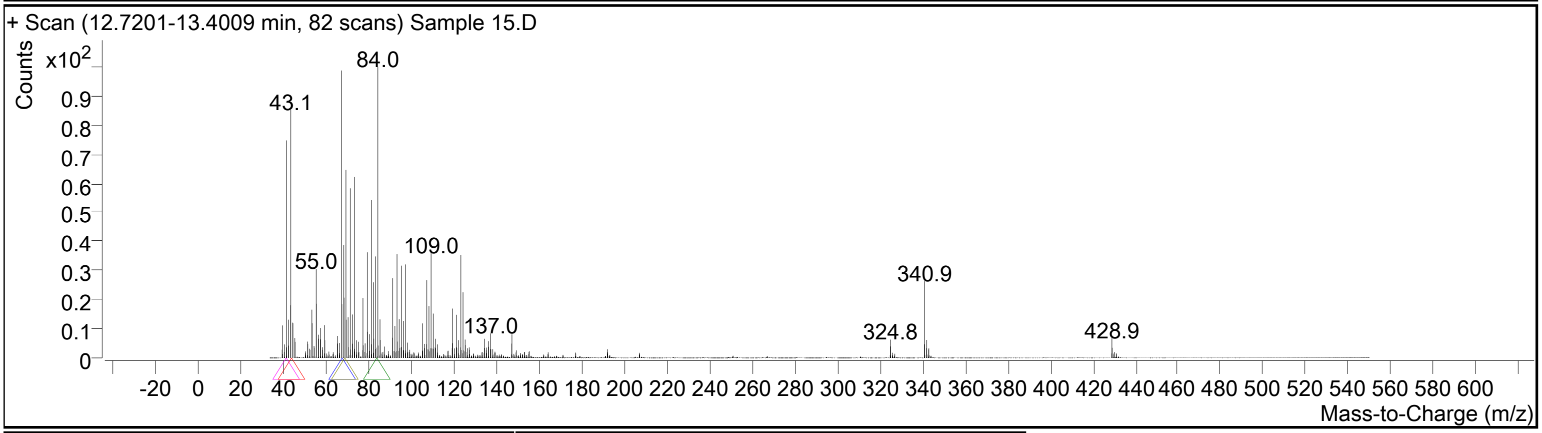

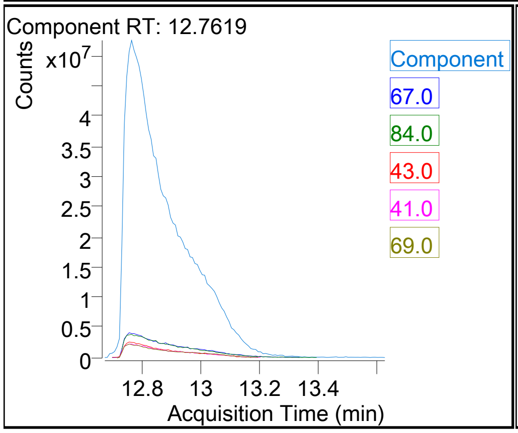

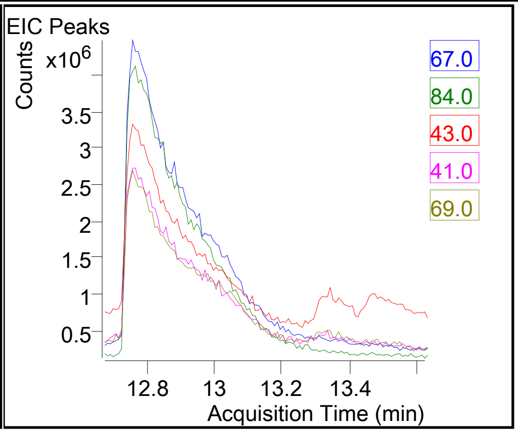

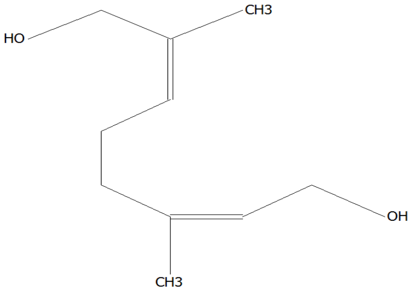

Library Search Results - NonTarget Hits with Details

Component RT	Compound Name	Component Area	Match Factor	CAS#	Formula	Estimated Conc.
13.4756	Acetophenone, 4'-hydroxy-	203883884.4	92.3	99-93-4	C8H8O2	

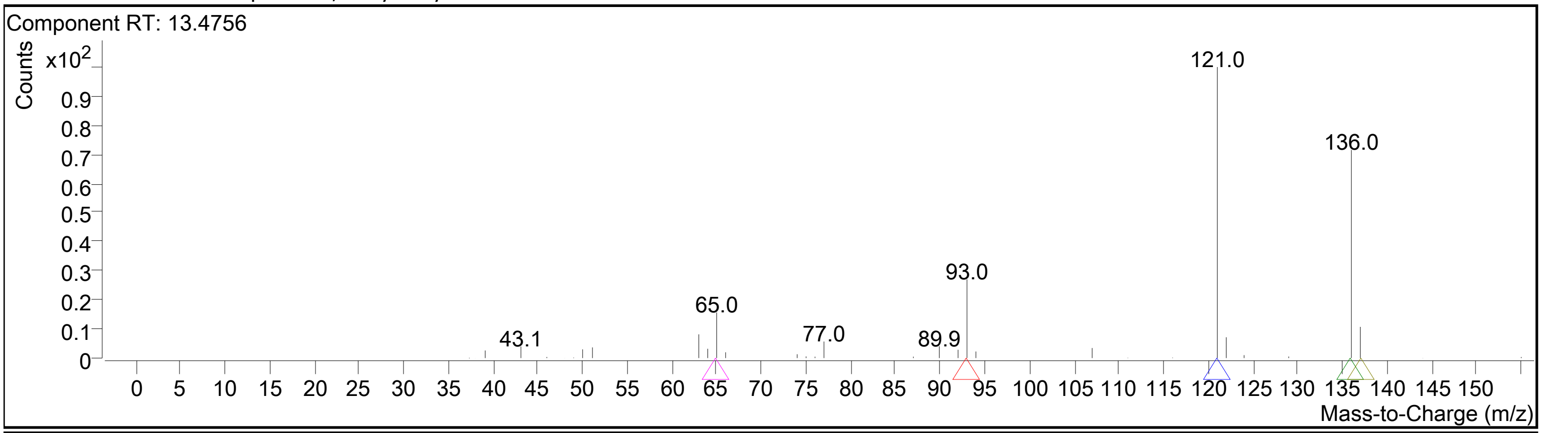

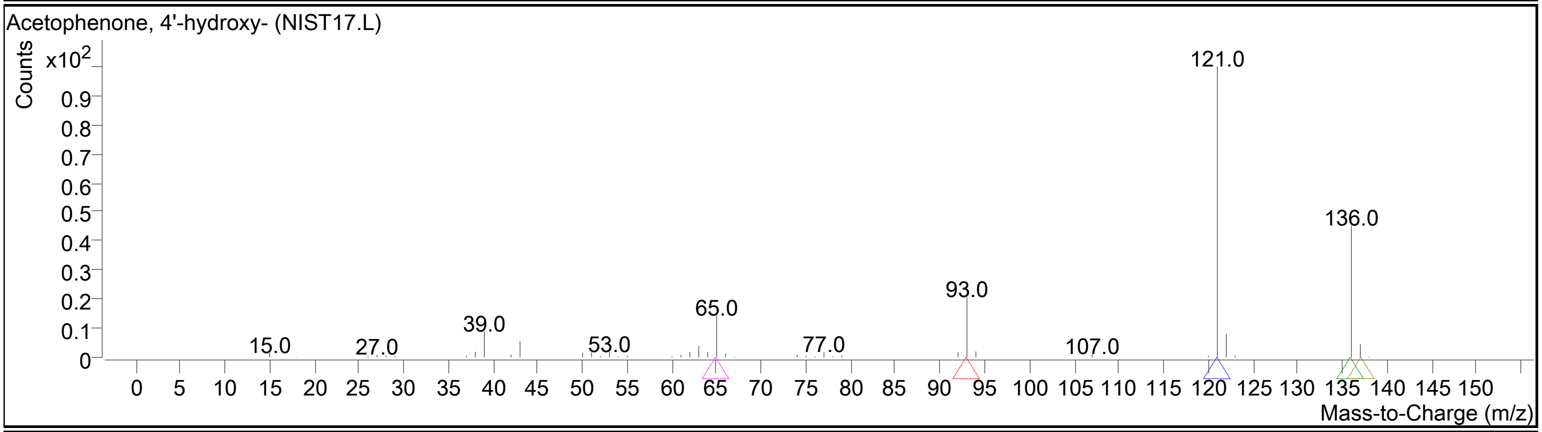

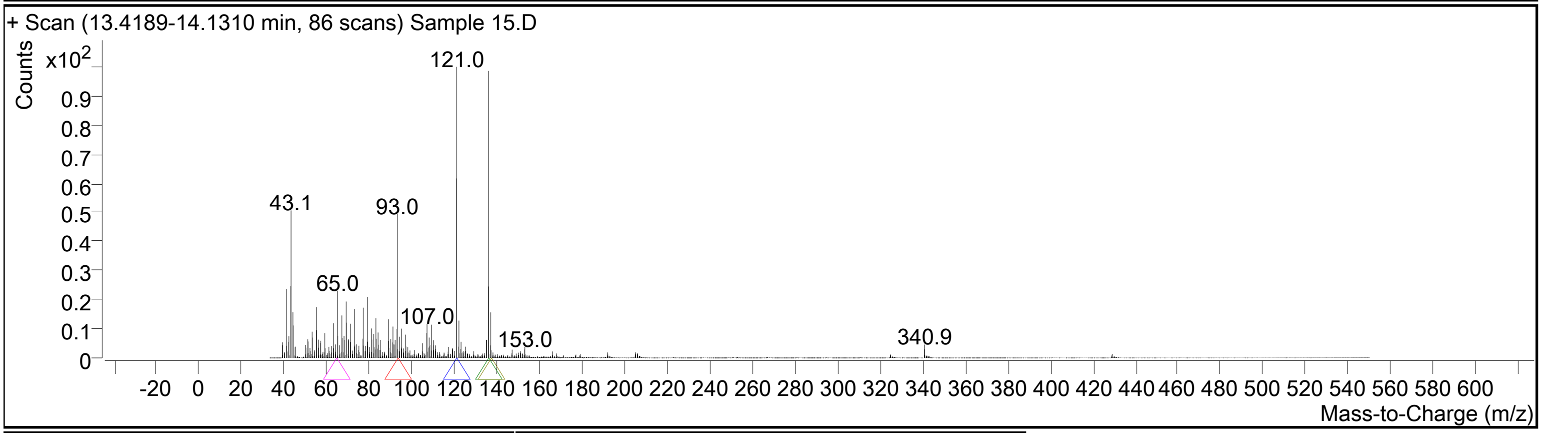

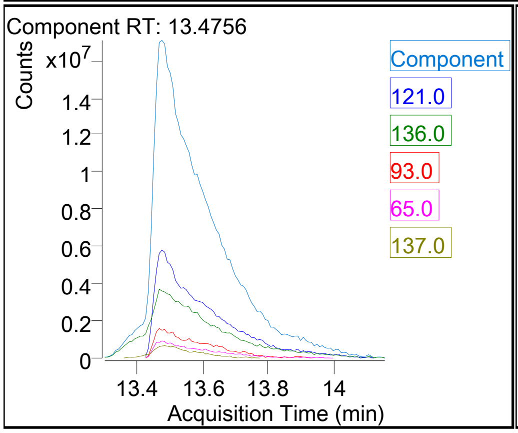

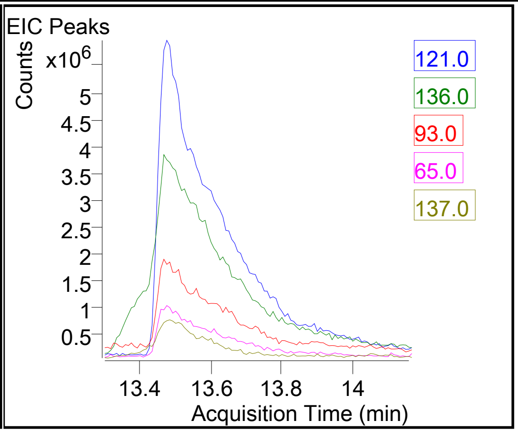

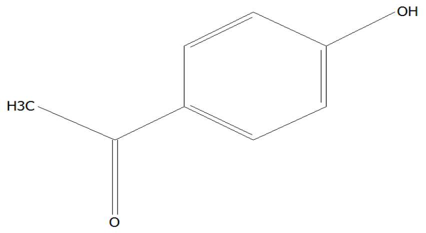

Component RT	Compound Name	Component Area	Match Factor	CAS#	Formula	Estimated Conc.
13.5267	Glycine, methyl ester	8616387.8	67.7	616-34-2	C3H7NO2	

Component RT: 13.5267

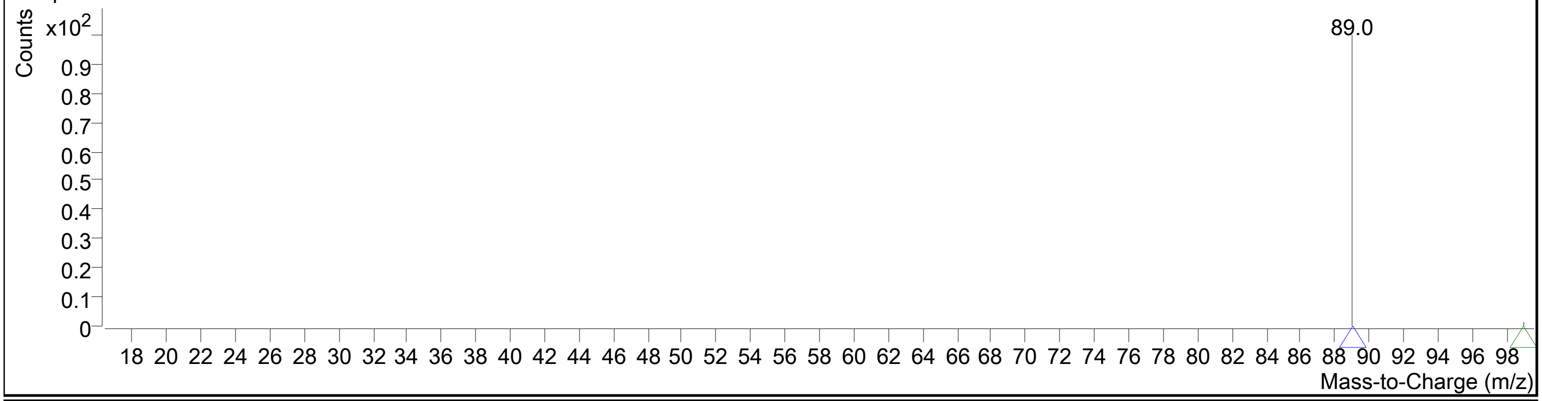

Glycine, methyl ester (NIST17.L)

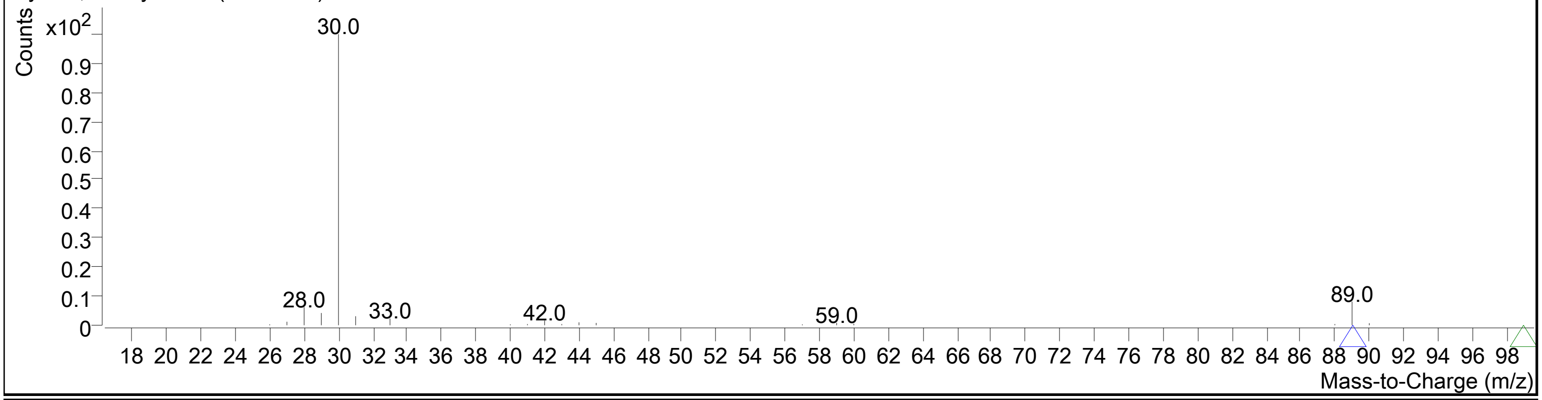

+ Scan (13.3131-14.0474 min, 89 scans) Sample 15.D

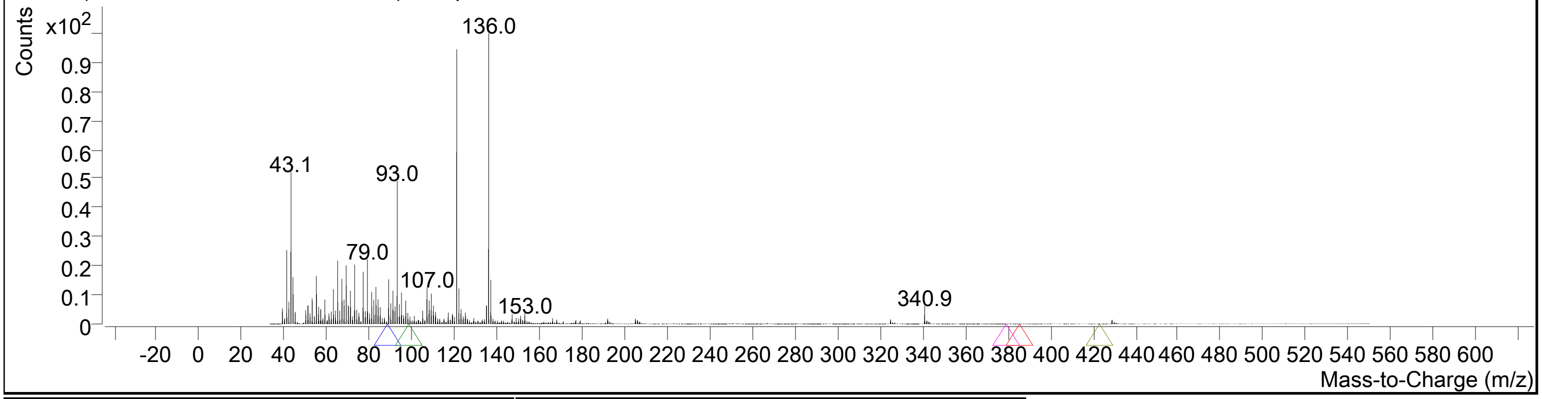

Component RT: 13.5267

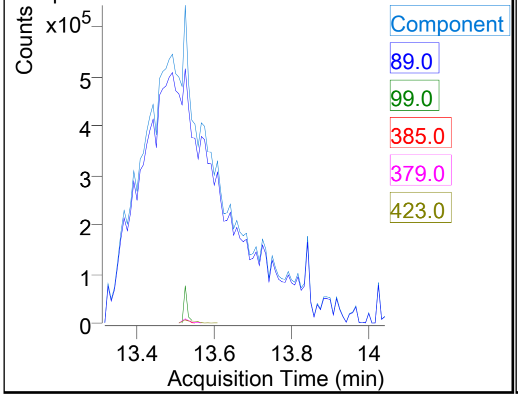

EIC Peaks

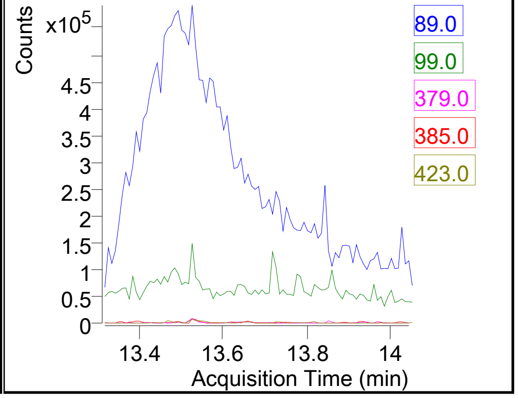

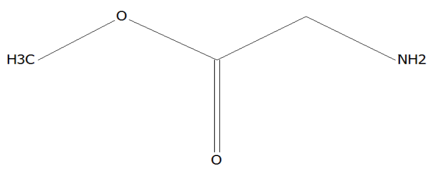

Component RT	Compound Name	Component Area	Match Factor	CAS#	Formula	Estimated Conc.
13.8200	1-Decanol	27360201.0	80.9	112-30-1	C10H22O	

Component RT: 13.8200

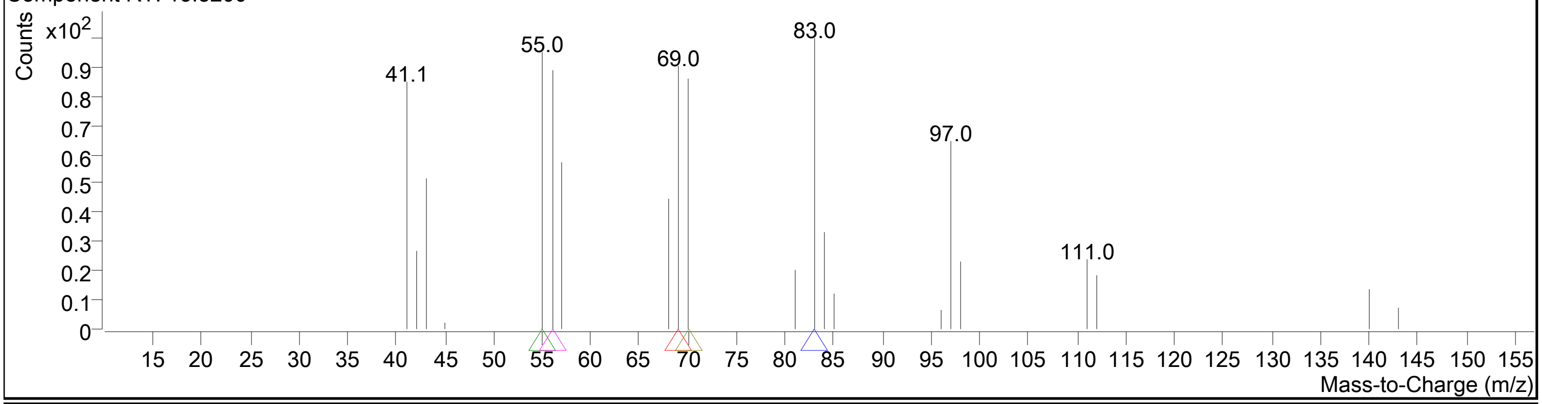

1-Decanol (NIST17.L)

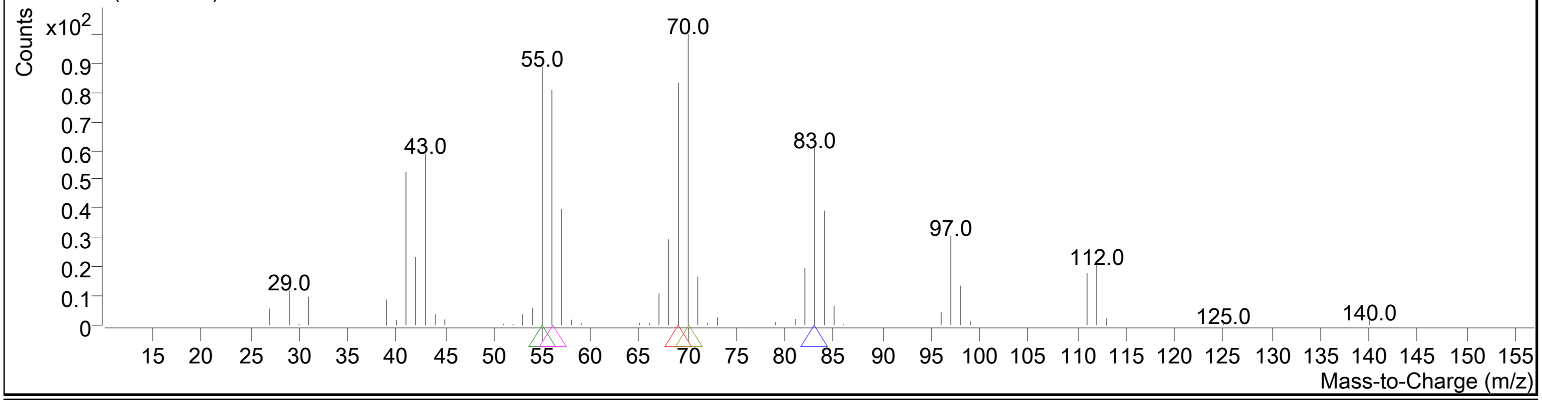

+ Scan (13.8343-13.8509 min, 2 scans) Sample 15.D

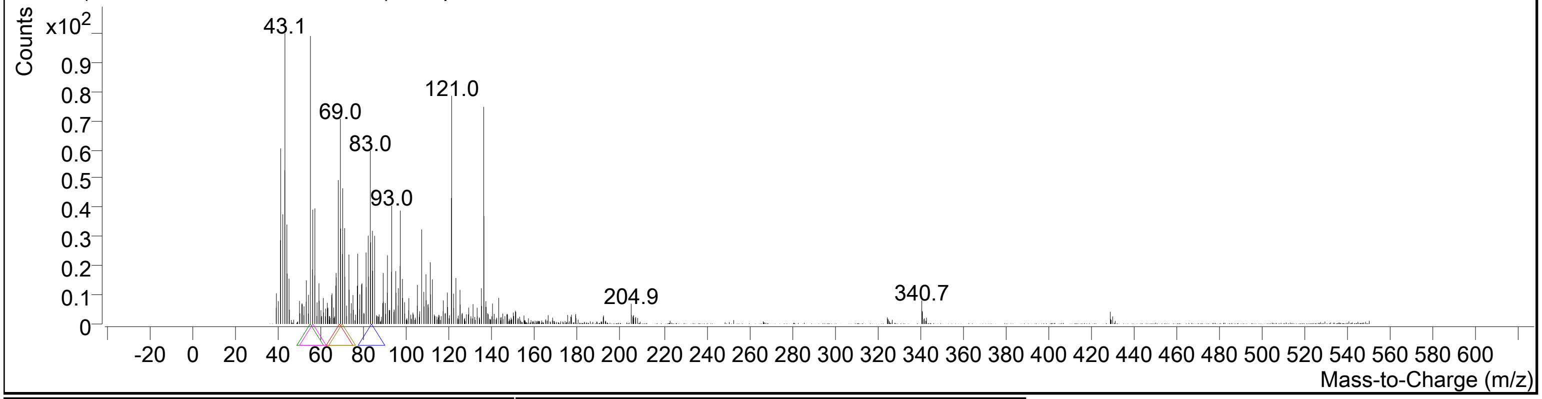

Component RT: 13.8200

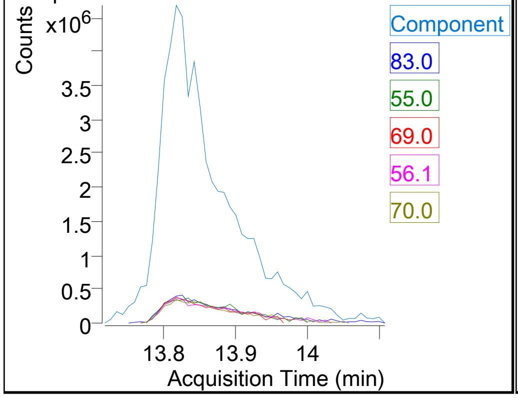

EIC Peaks

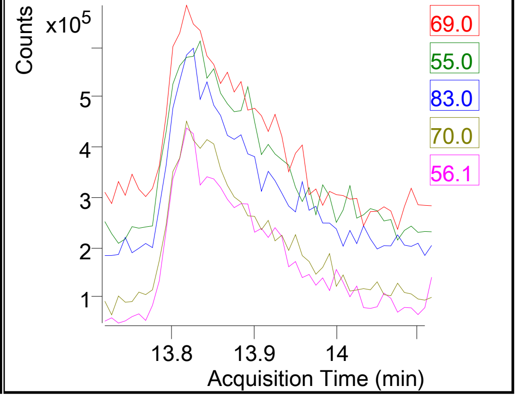

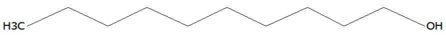

Component RT	Compound Name	Component Area	Match Factor	CAS#	Formula	Estimated Conc.
14.2688	Ethanone, 1-(2-hydroxy-4-methoxyphenyl)-	69695815.7	82.3	552-41-0	C9H10O3	

Component RT: 14.2688

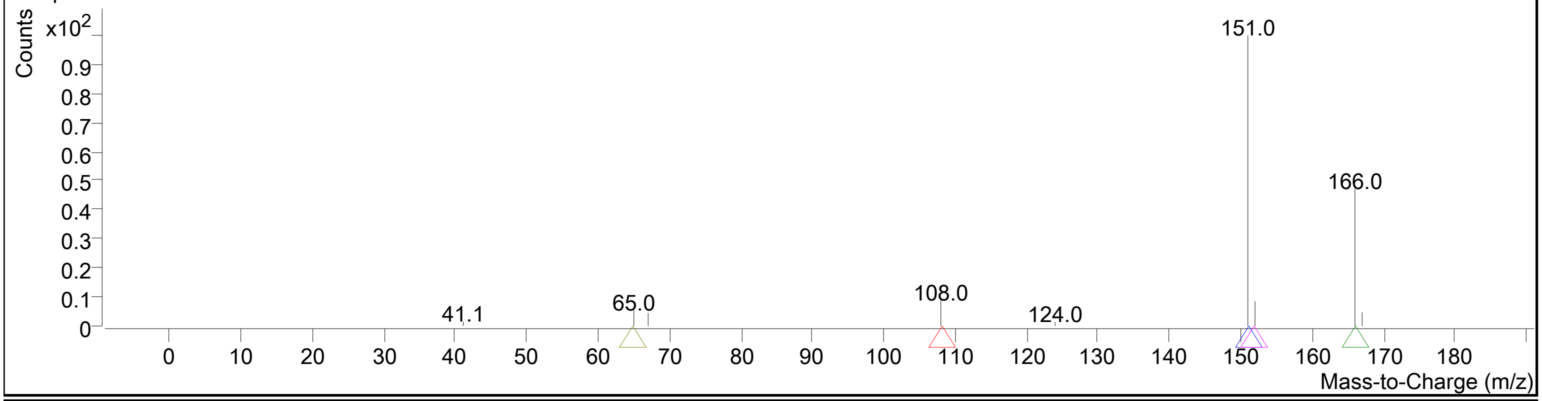

Ethanone, 1-(2-hydroxy-4-methoxyphenyl)- (NIST17.L)

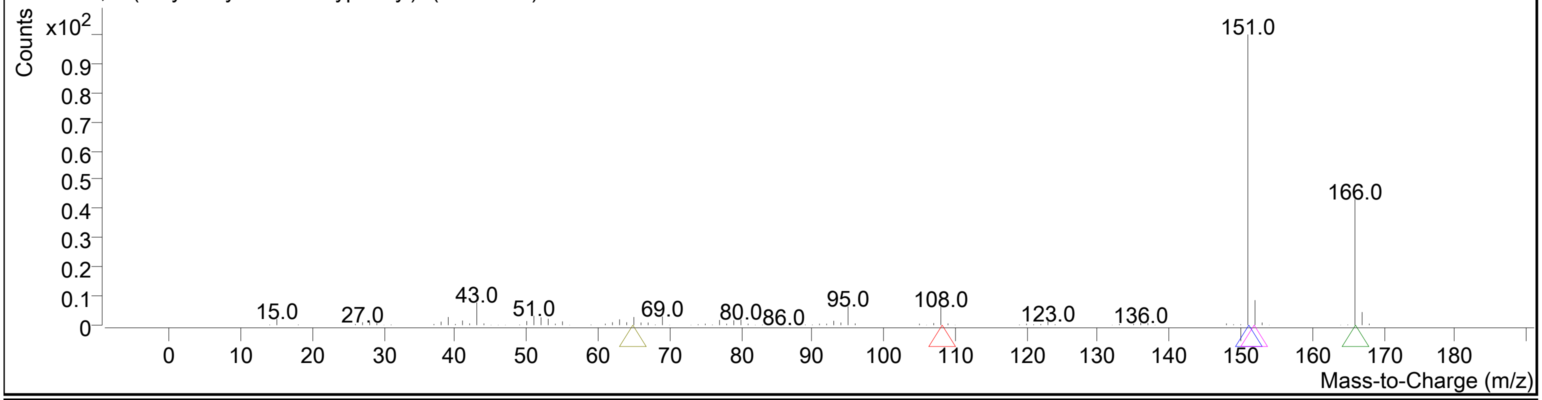

+ Scan (14.2085-14.5743 min, 44 scans) Sample 15.D

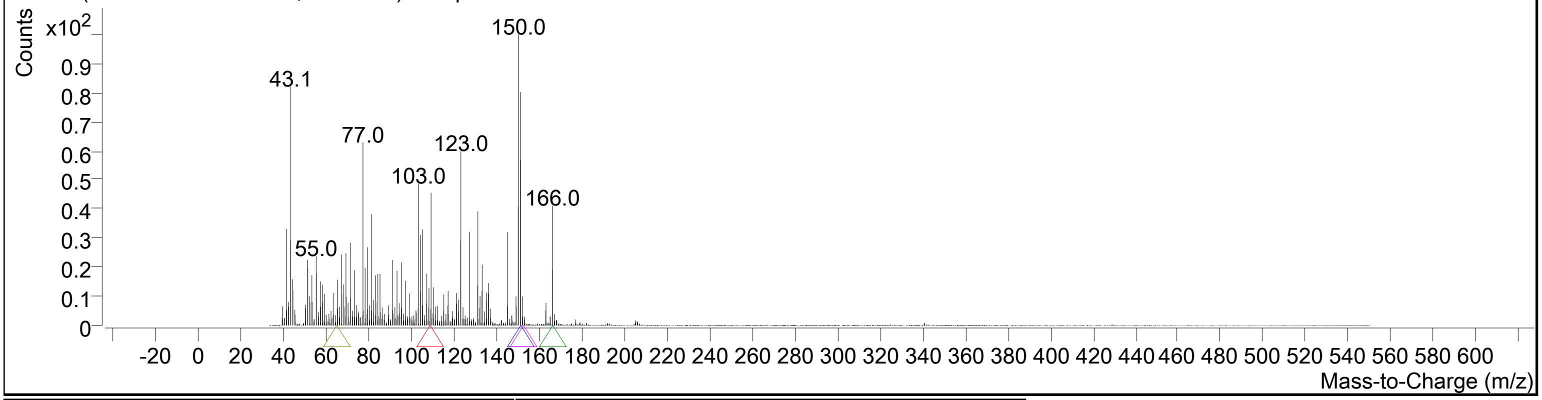

Component RT: 14.2688

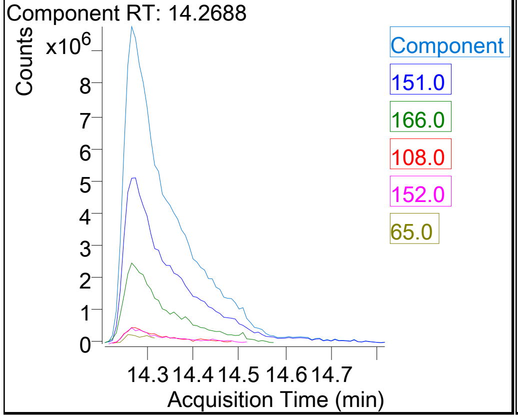

EIC Peaks

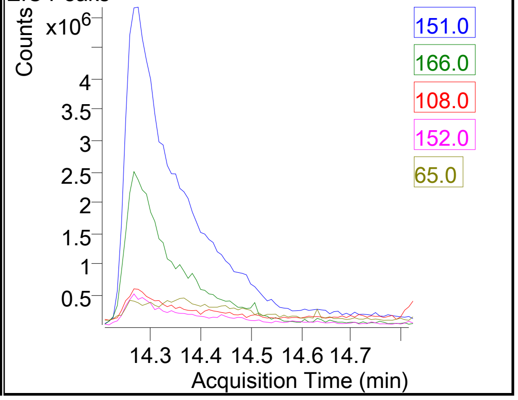

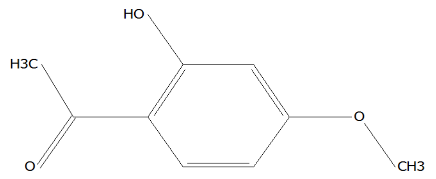

Library Search Results - NonTarget Hits with Details

Agilent Technologies

Component RT	Compound Name	Component Area	Match Factor	CAS#	Formula	Estimated Conc.
14.3578	Hydroxychavicol	234244947.6	86.6	1126-61-0	C9H10O2	

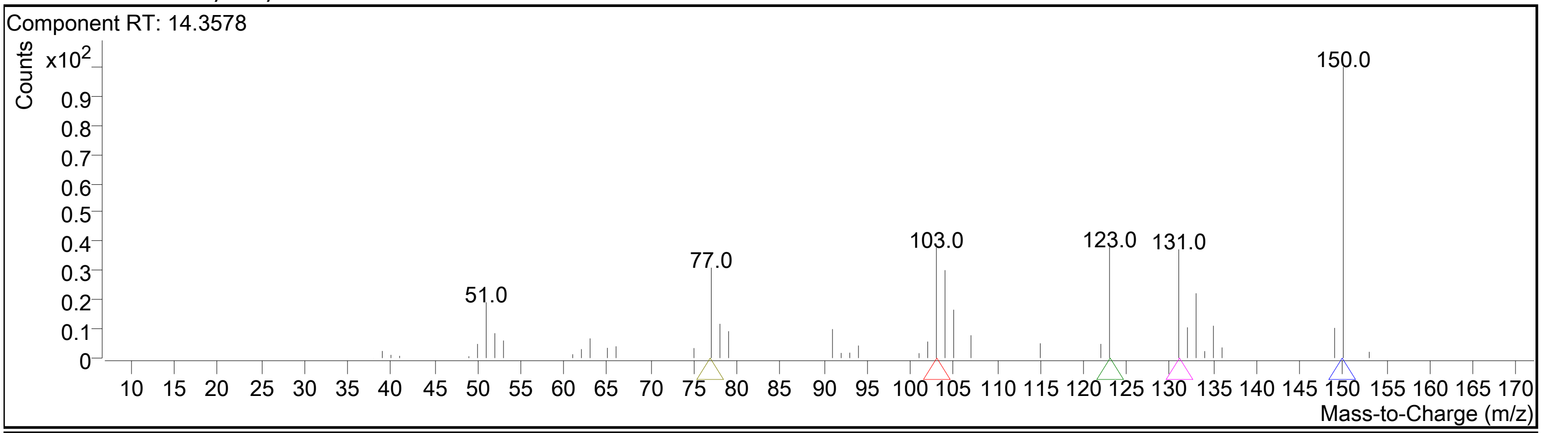

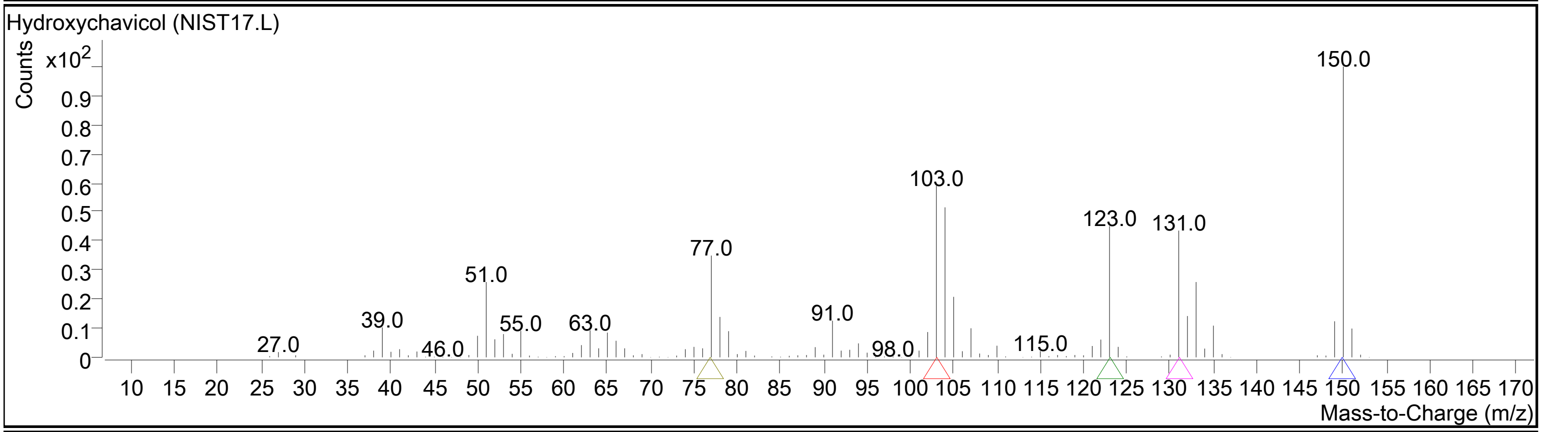

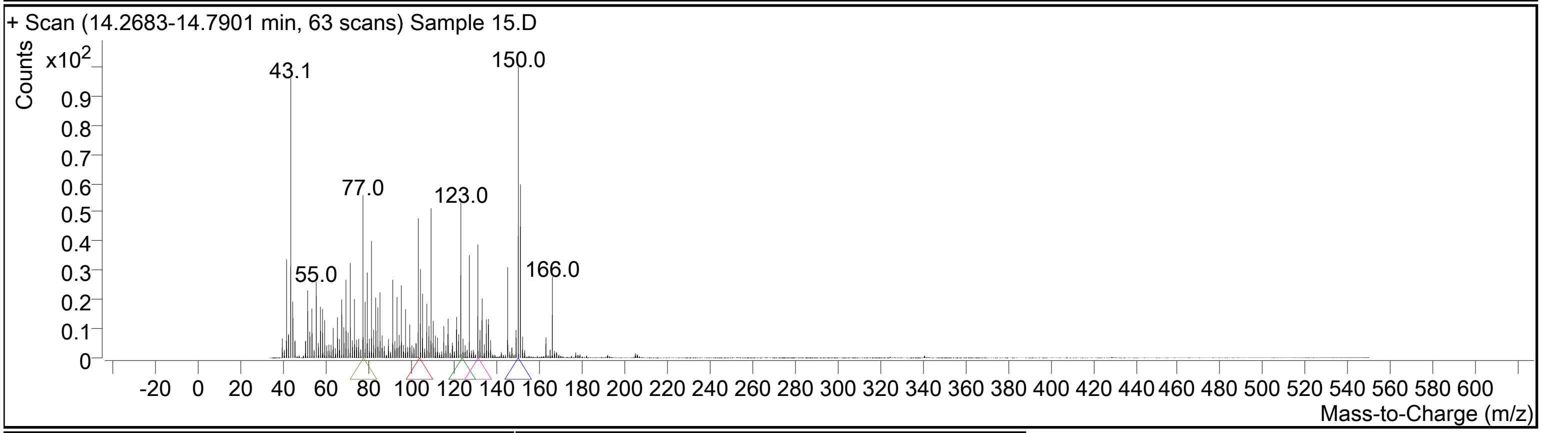

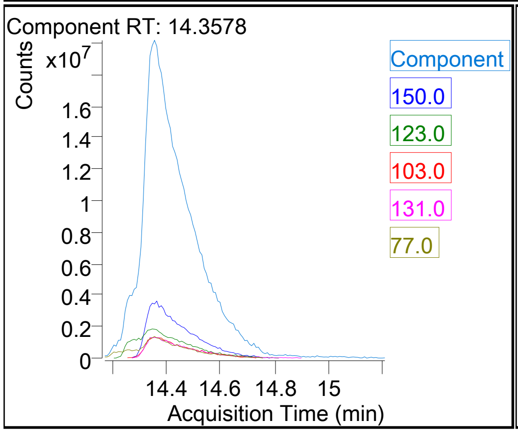

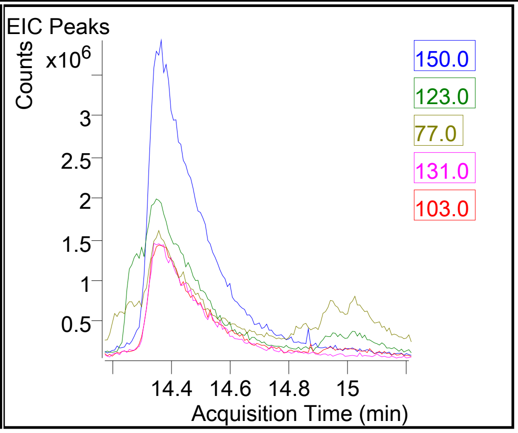

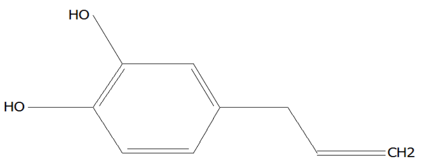

Component RT	Compound Name	Component Area	Match Factor	CAS#	Formula	Estimated Conc.
14.4513	(1S,2S,4S)-Trihydroxy-p-menthane	157460019.0	85.6	22555-61-9	C10H20O3	

Component RT: 14.4513

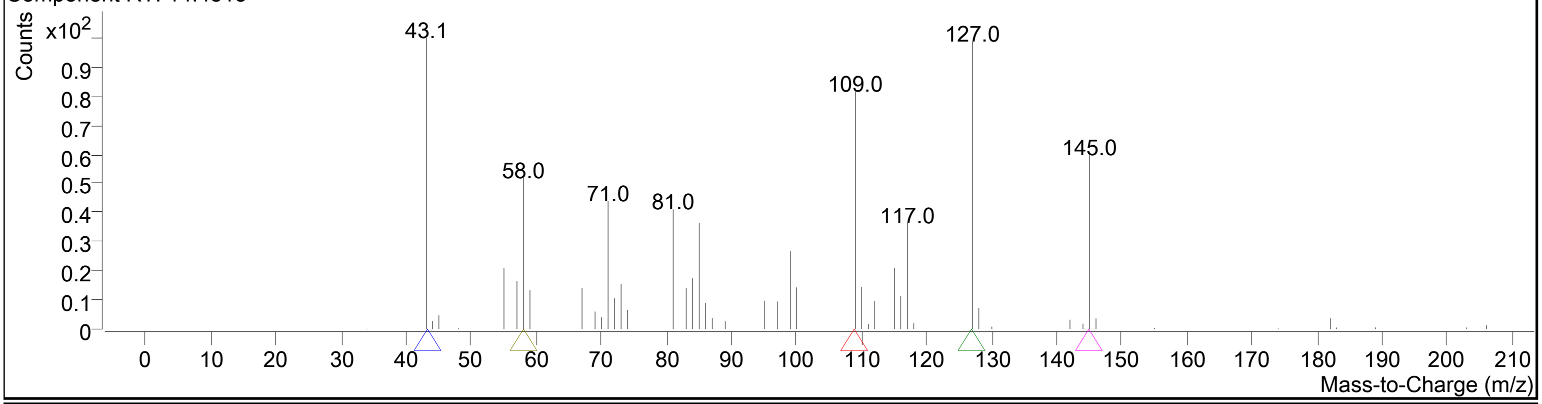

(1S,2S,4S)-Trihydroxy-p-menthane (NIST17.L)

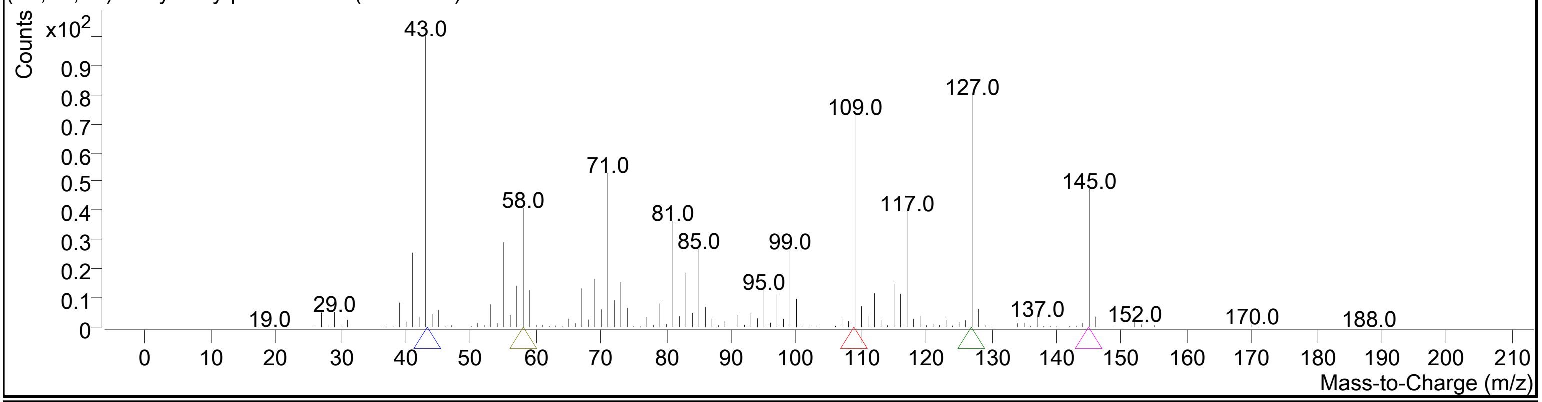

+ Scan (14.3748-14.7739 min, 48 scans) Sample 15.D

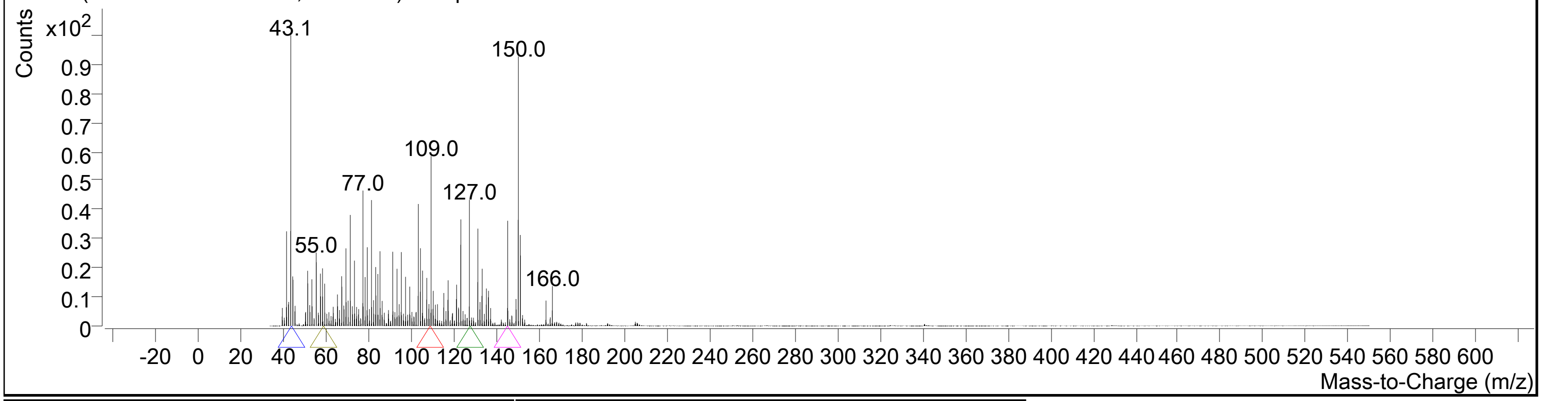

Component RT: 14.4513

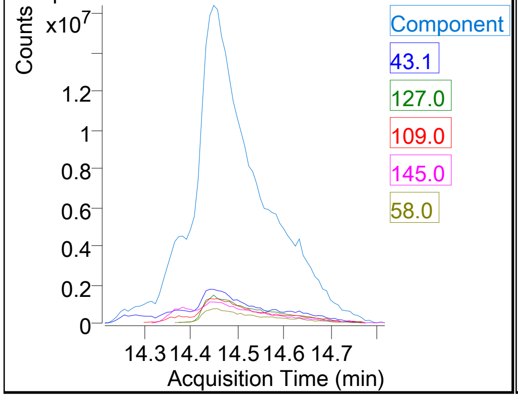

EIC Peaks

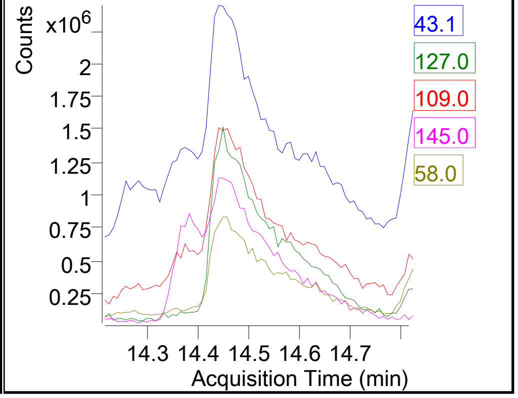

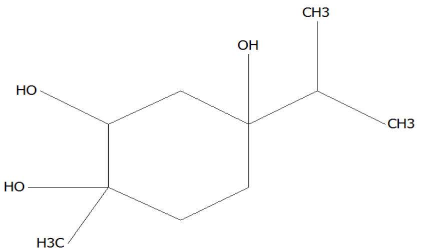

Component RT	Compound Name	Component Area	Match Factor	CAS#	Formula	Estimated Conc.
15.0231	2-Caren-4-ol	82688352.5	77.0	6617-35-2	C10H16O	

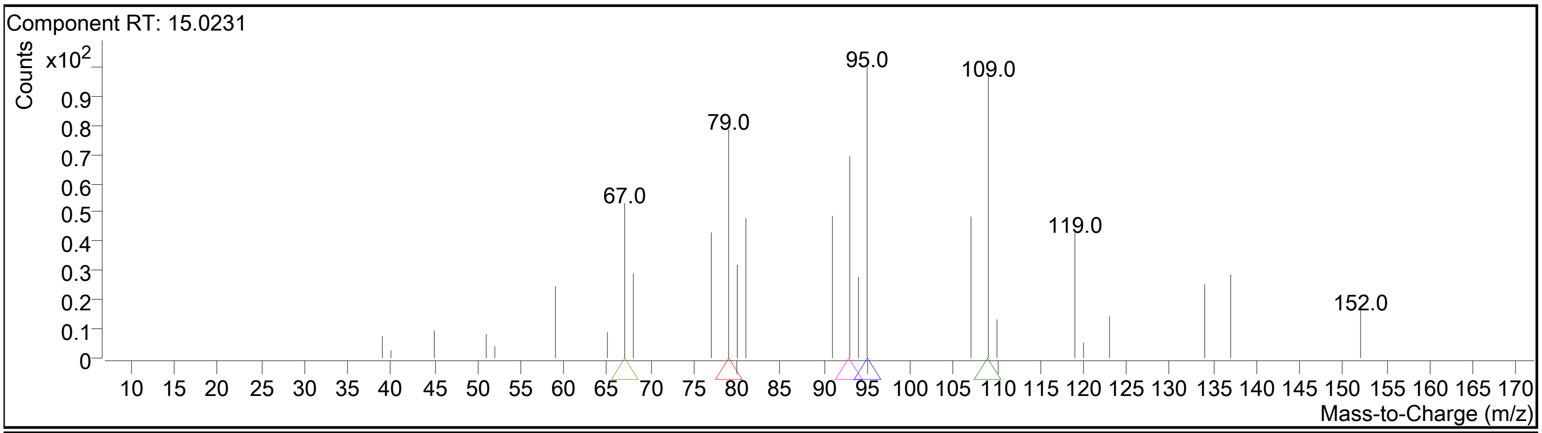

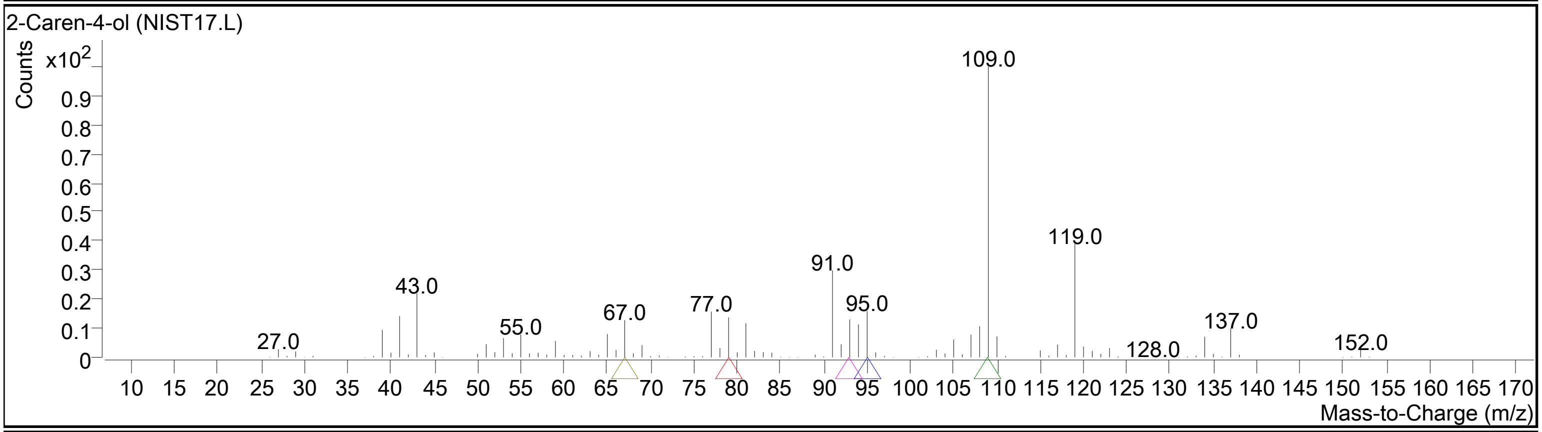

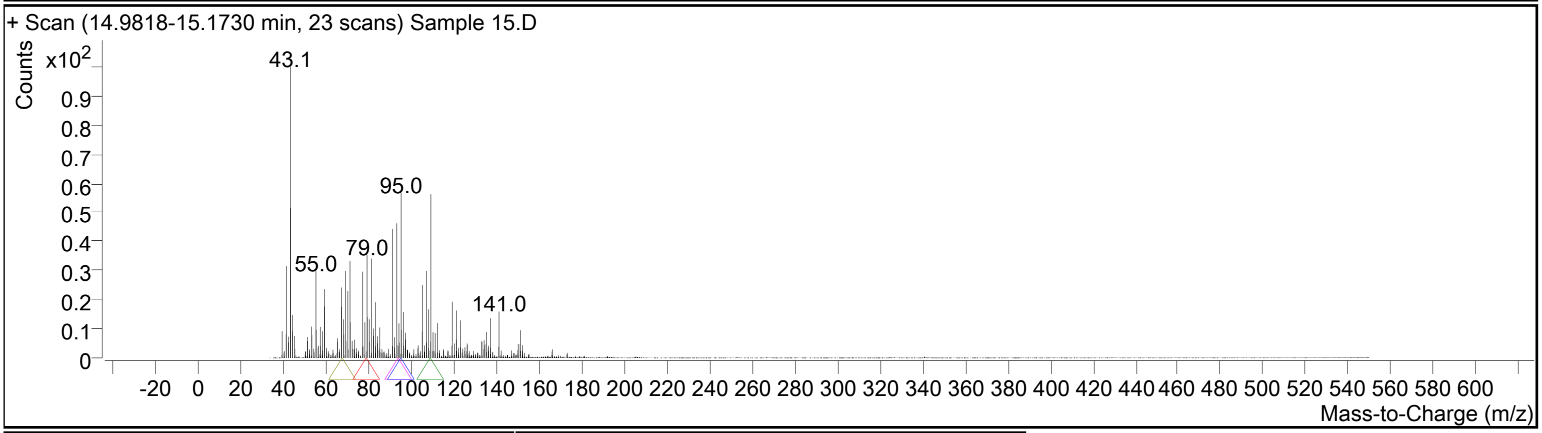

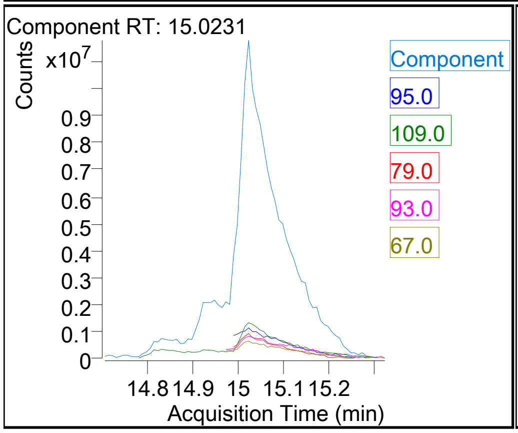

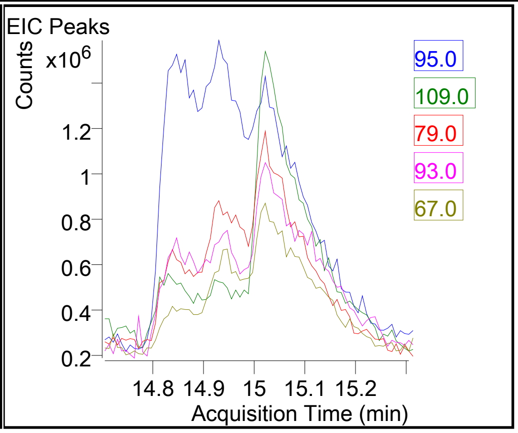

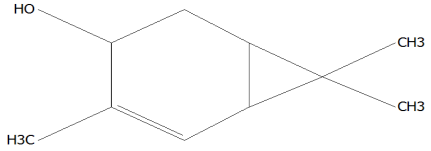

Component RT	Compound Name	Component Area	Match Factor	CAS#	Formula	Estimated Conc.
15.8057	2(4H)-Benzofuranone, 5,6,7,7a-tetrahydro-4,4,7a-trimethyl-, (R)-	28315348.1	74.8	17092-92-1	C11H16O2	

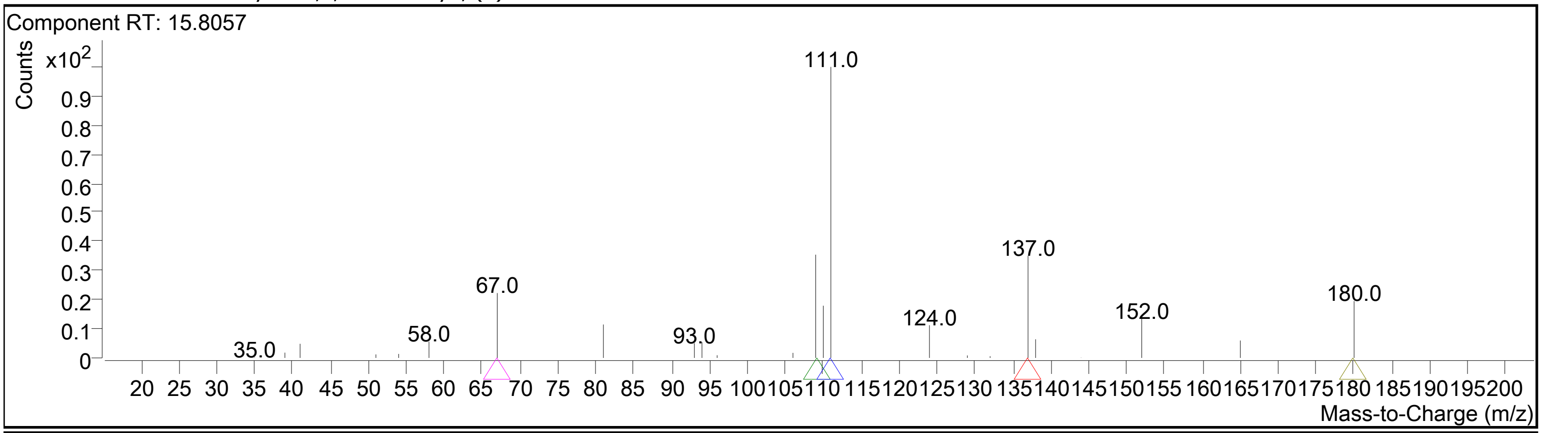

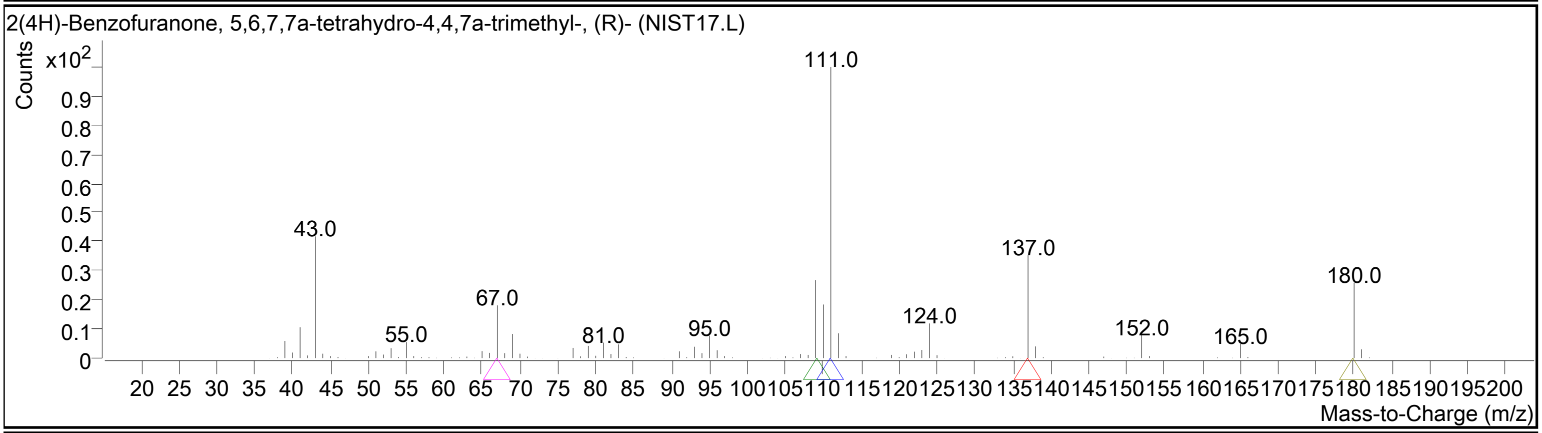

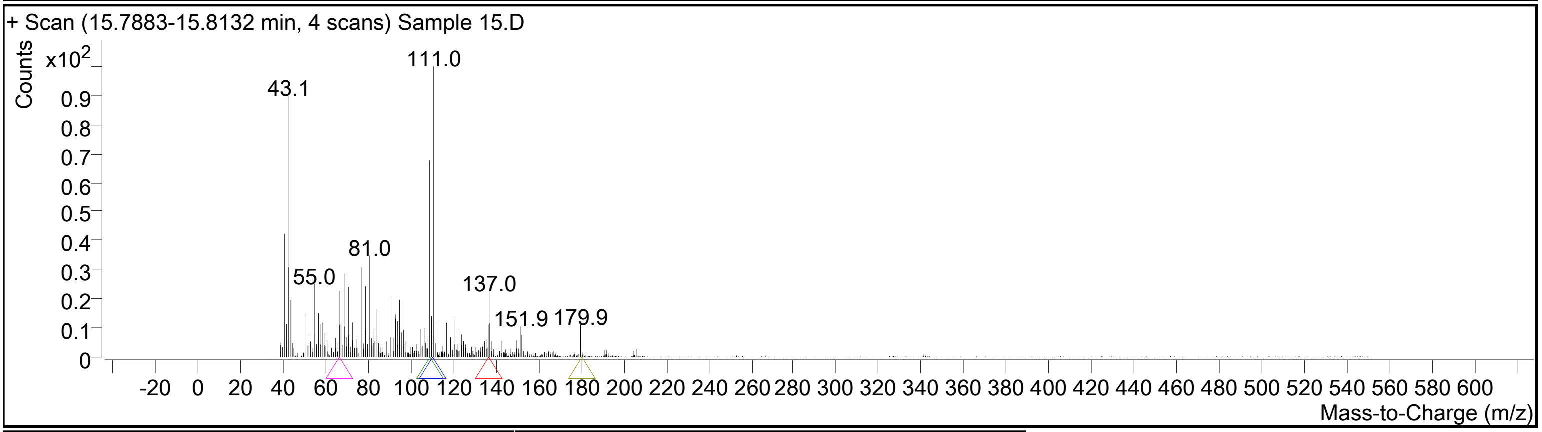

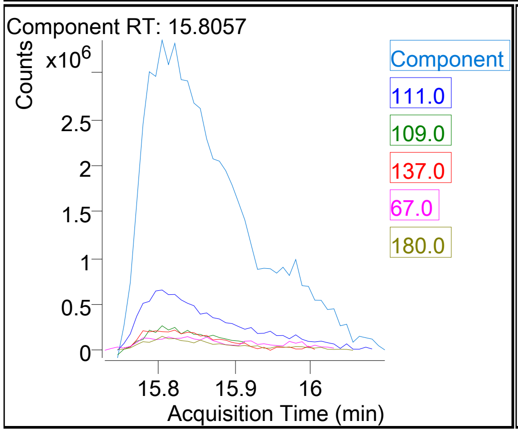

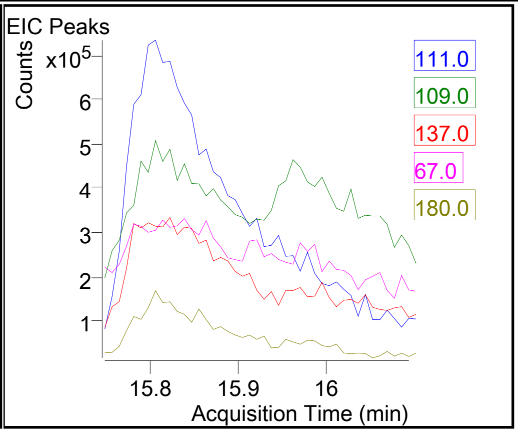

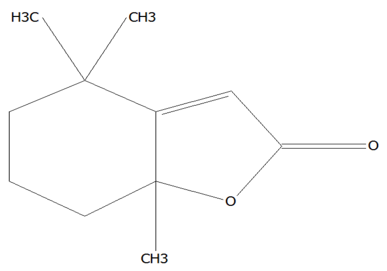

Generated at 12:24 PM on 2/25/2021

Agilent Technologies

Component RT	Compound Name	Component Area	Match Factor	CAS#	Formula	Estimated Conc.
15.8725	2,4-Di-tert-butylphenol	45393295.5	83.6	96-76-4	C14H22O	

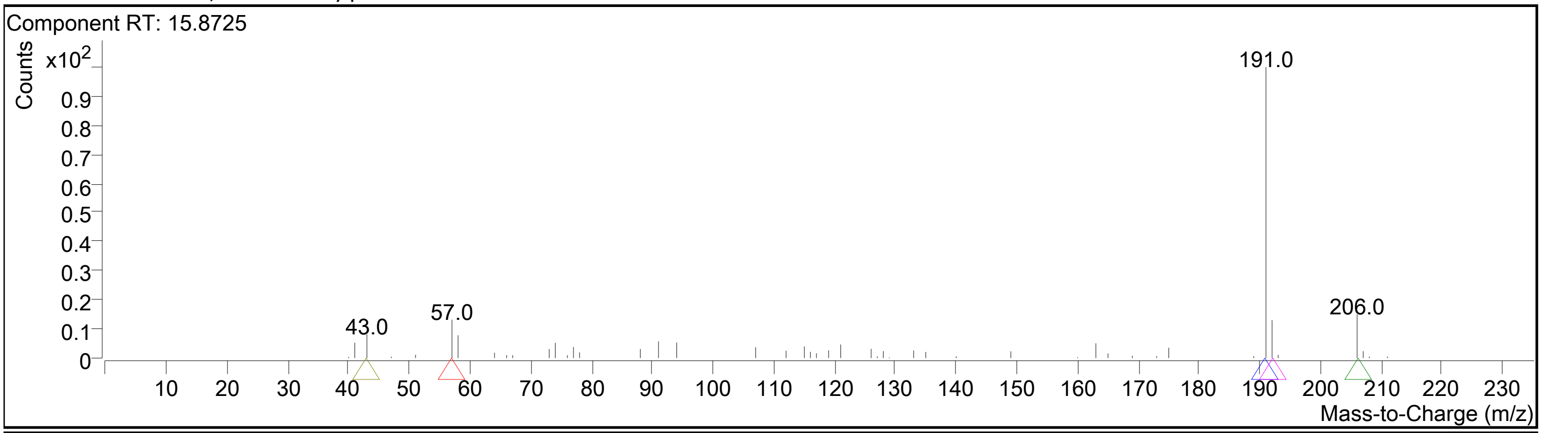

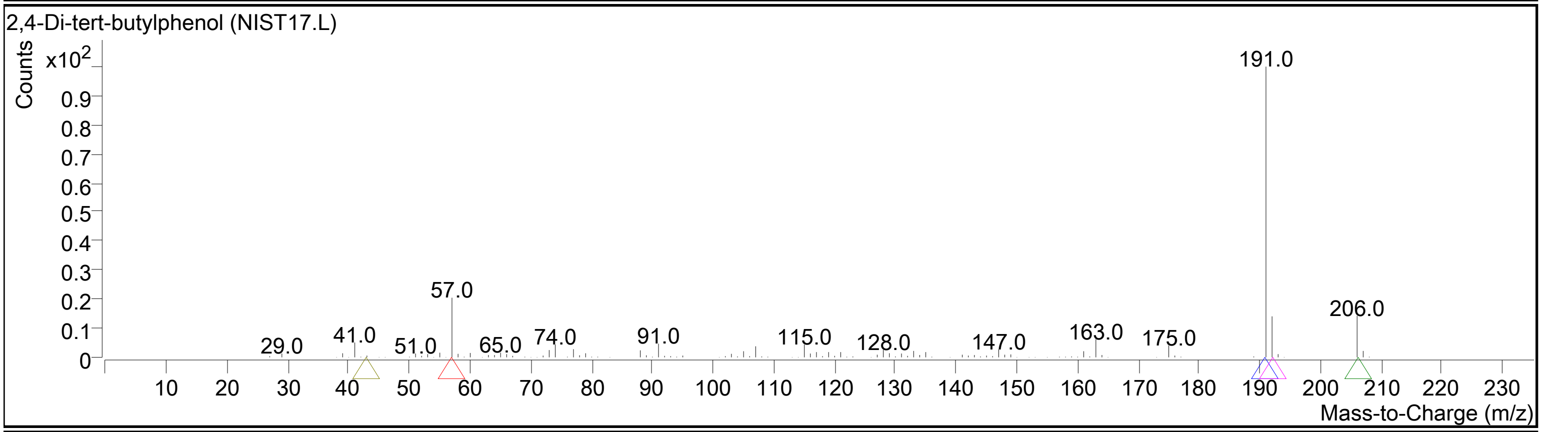

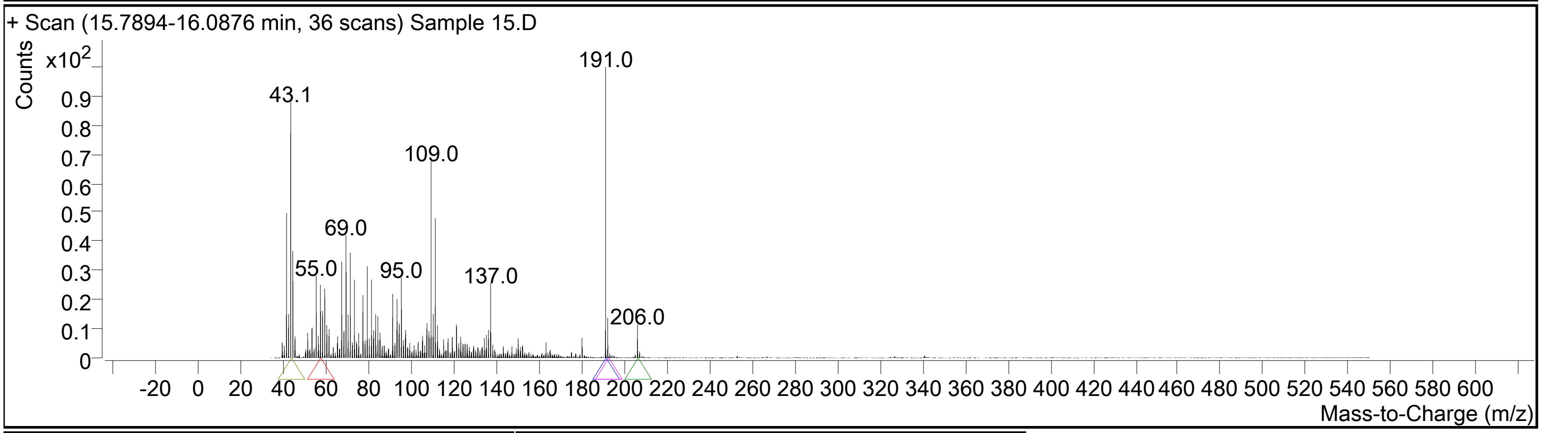

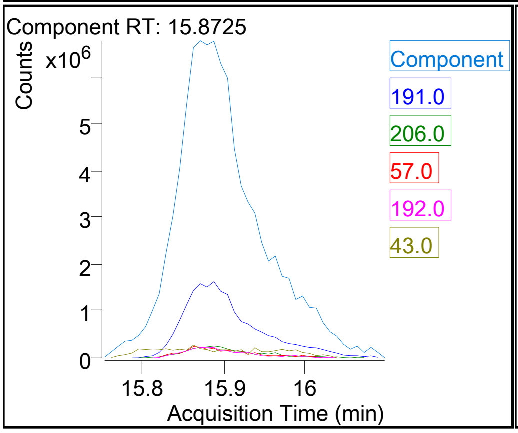

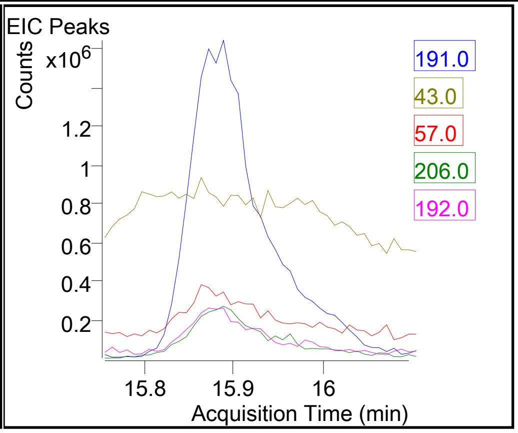

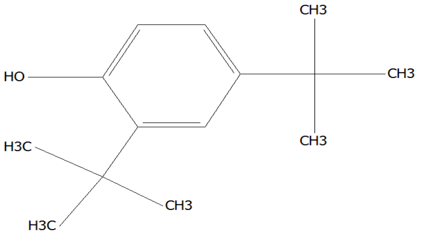

Component RT	Compound Name	Component Area	Match Factor	CAS#	Formula	Estimated Conc.
16.7055	2(3H)-Furanone, dihydro-5,5-dimethyl-4-(3-oxobutyl)-	87657582.3	81.0	4436-81-1	C10H16O3	

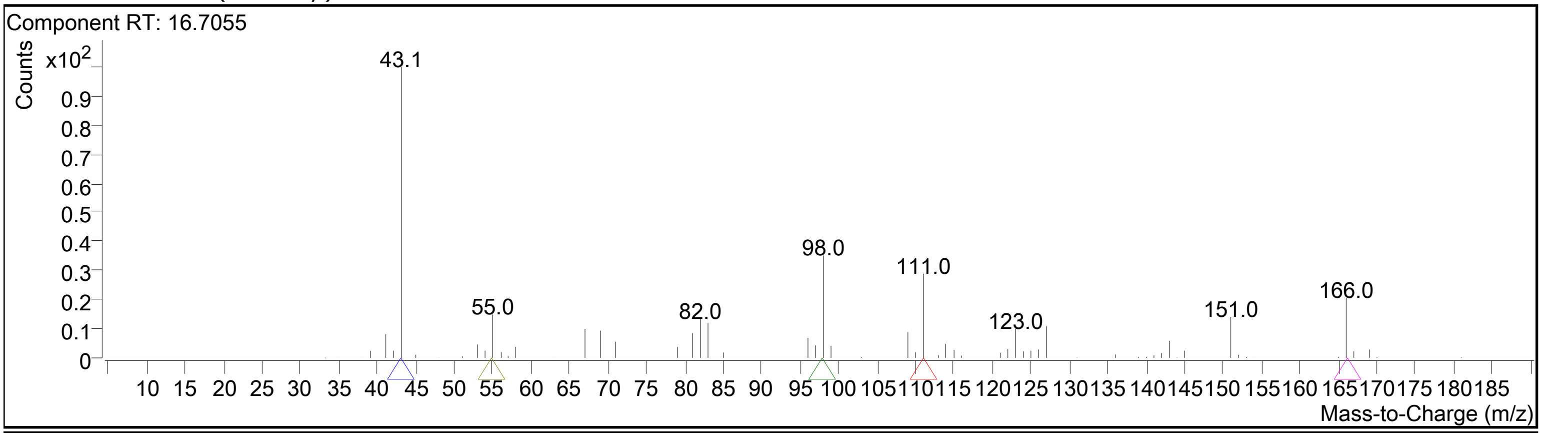

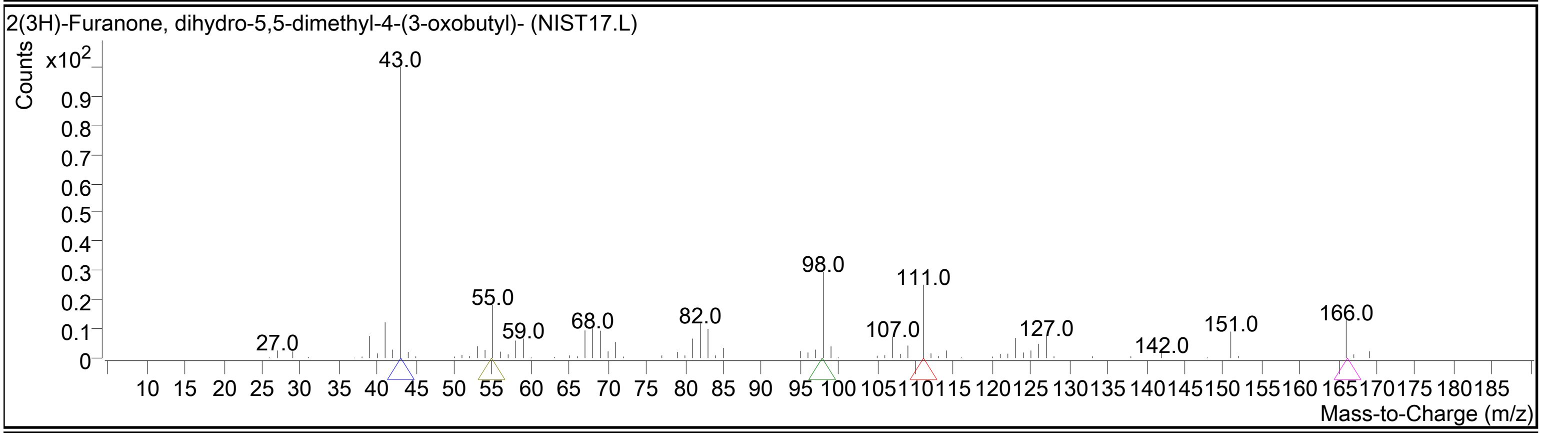

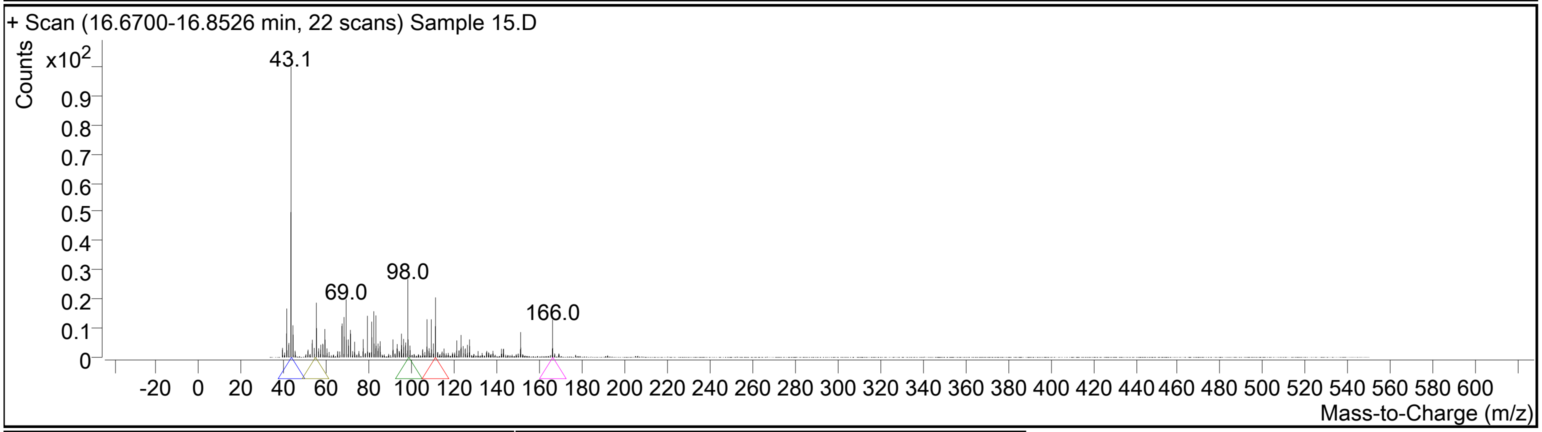

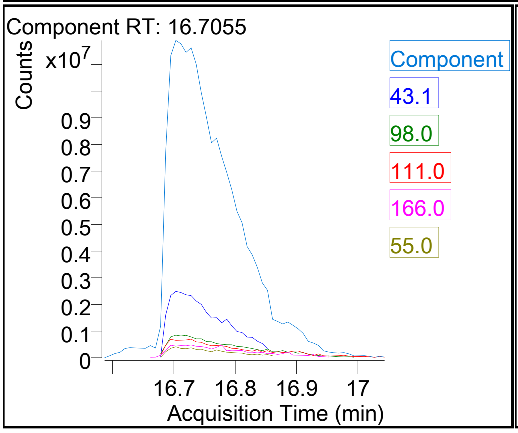

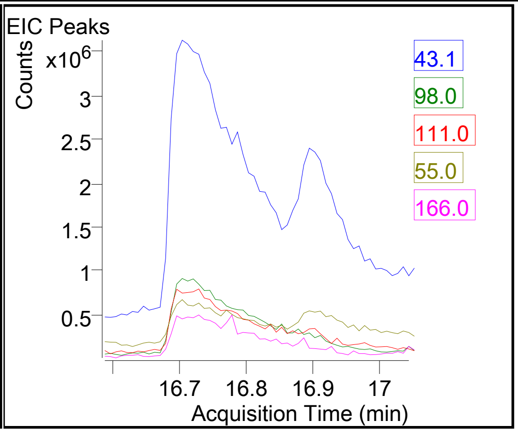

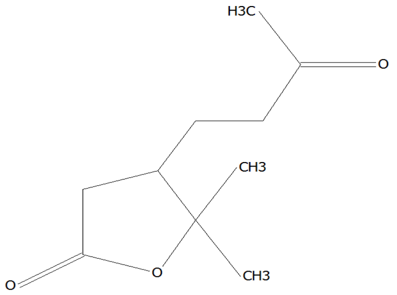

Agilent Technologies

Component RT	Compound Name	Component Area	Match Factor	CAS#	Formula	Estimated Conc.
16.9017	6-Hydroxy-3,7-dimethyl-2,7-octadienyl acetate(E)	58854598.0	87.8	33766-43-7	C12H20O3	

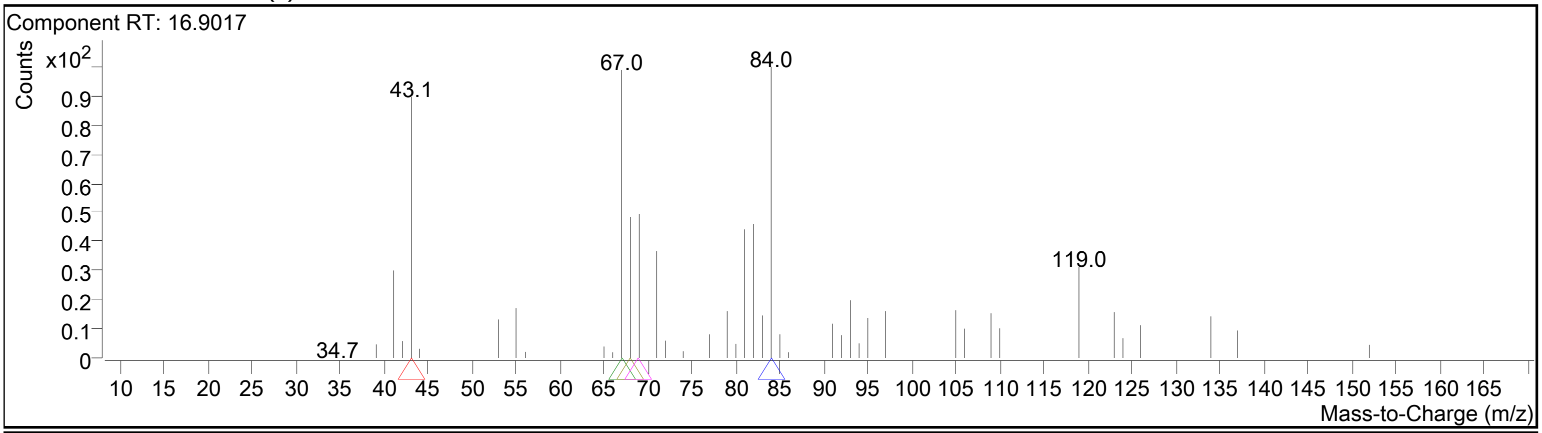

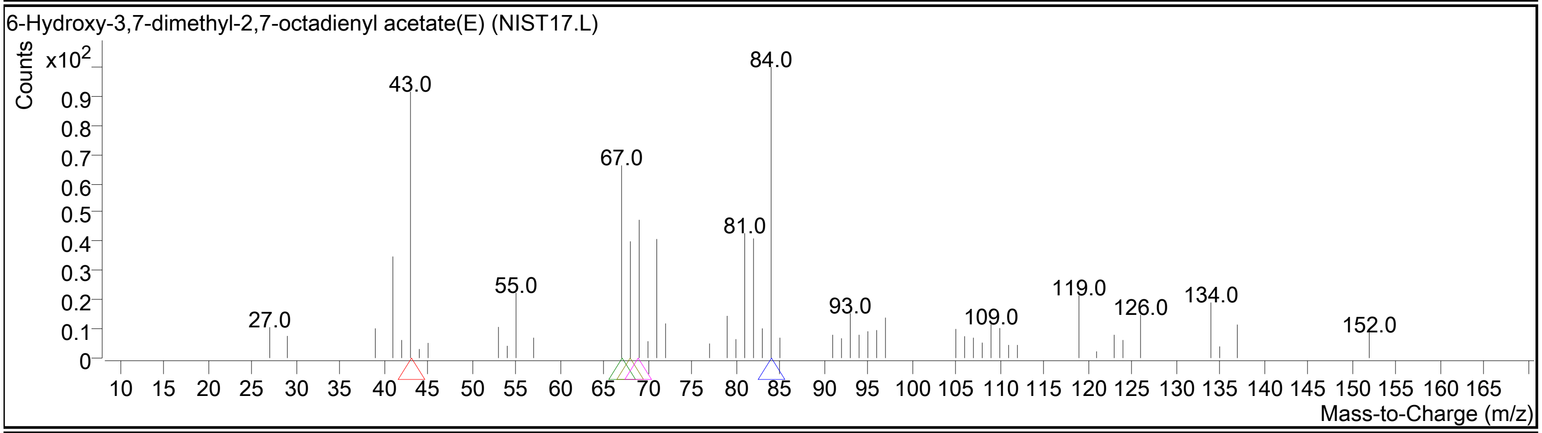

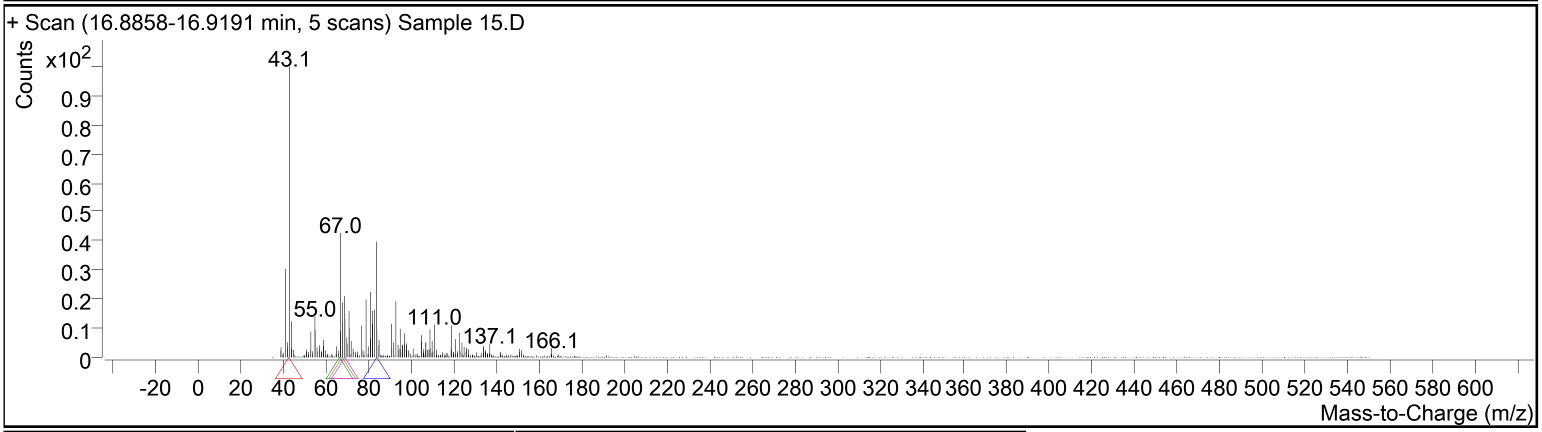

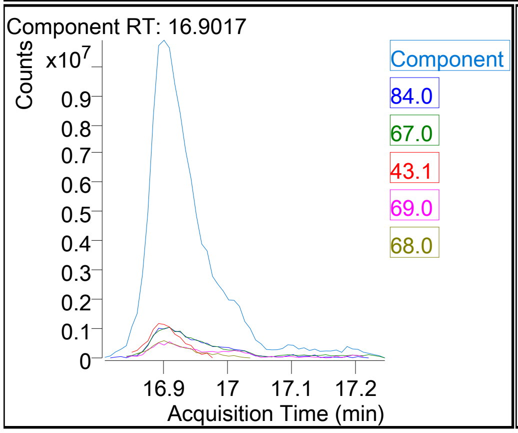

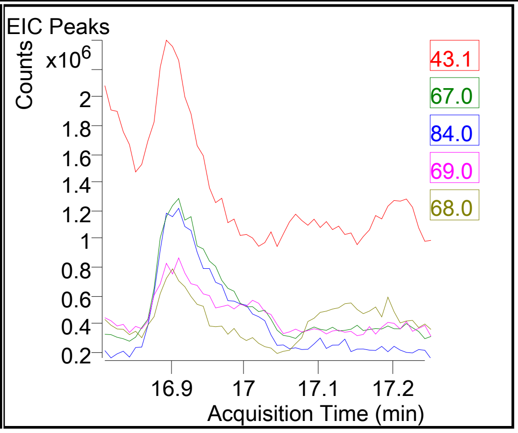

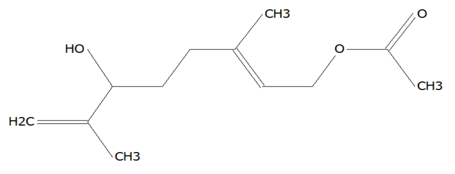

Generated at 12:24 PM on 2/25/2021

Library Search Results - NonTarget Hits with Details

Component RT	Compound Name	Component Area	Match Factor	CAS#	Formula	Estimated Conc.
17.0768	Phenol, 4-pentyl-	42825874.6	80.0	14938-35-3	C11H16O	

Component RT: 17.0768

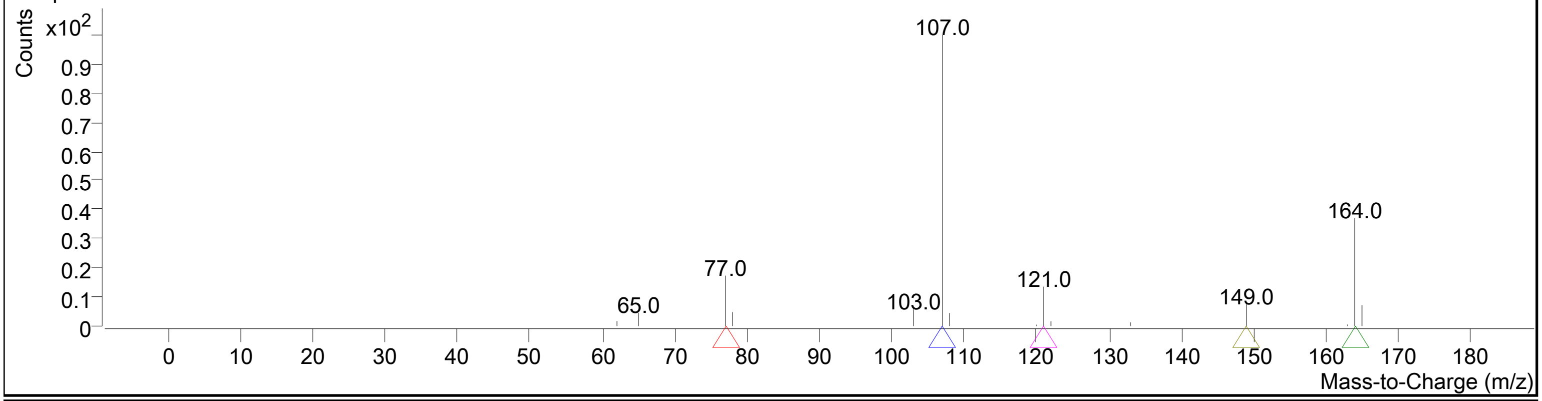

Phenol, 4-pentyl- (NIST17.L)

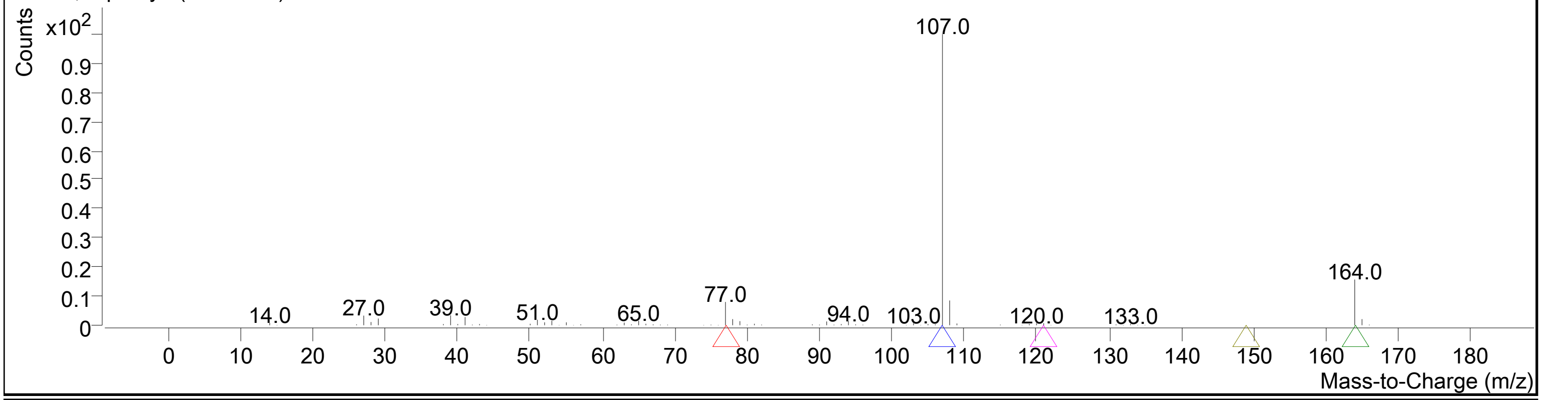

+ Scan (17.0272-17.2257 min, 24 scans) Sample 15.D

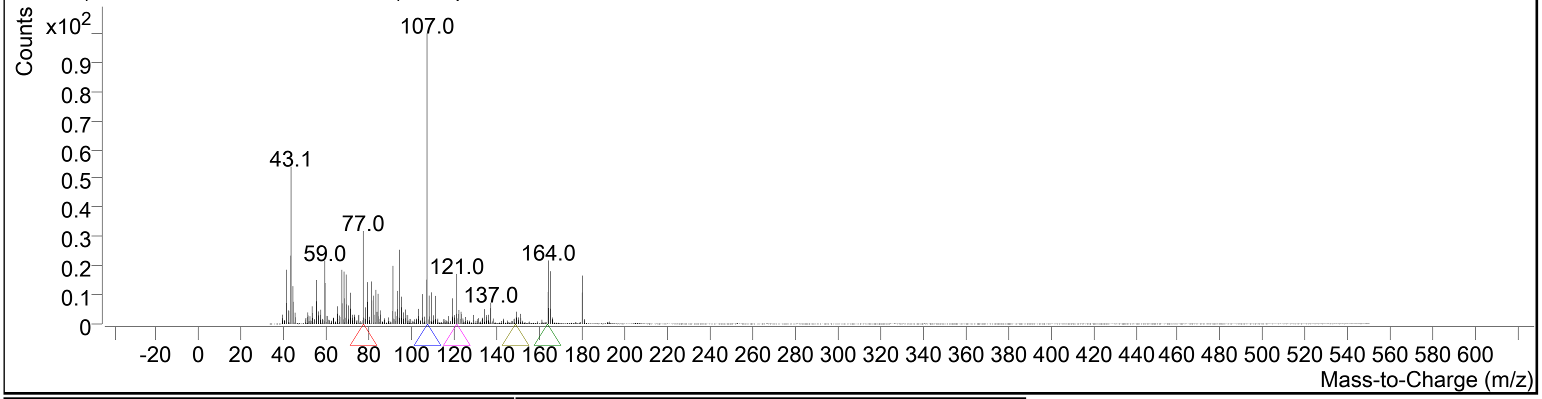

Component RT: 17.0768

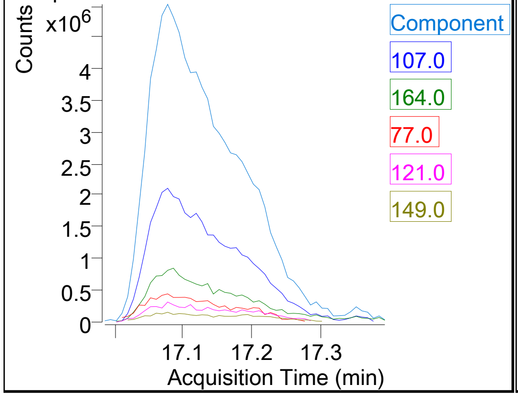

EIC Peaks

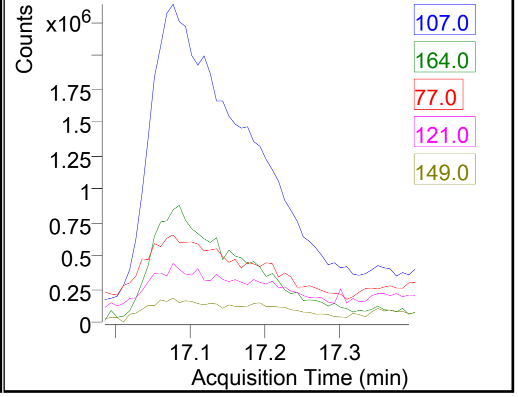

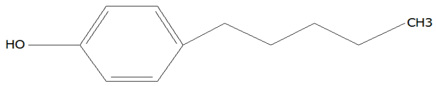

 Generated at 12:24 PM on 2/25/2021

Component RT	Compound Name	Component Area	Match Factor	CAS#	Formula	Estimated Conc.
17.4597	Caryophyllene oxide	500658899.7	89.0	1139-30-6	C15H24O	

Component RT: 17.4597

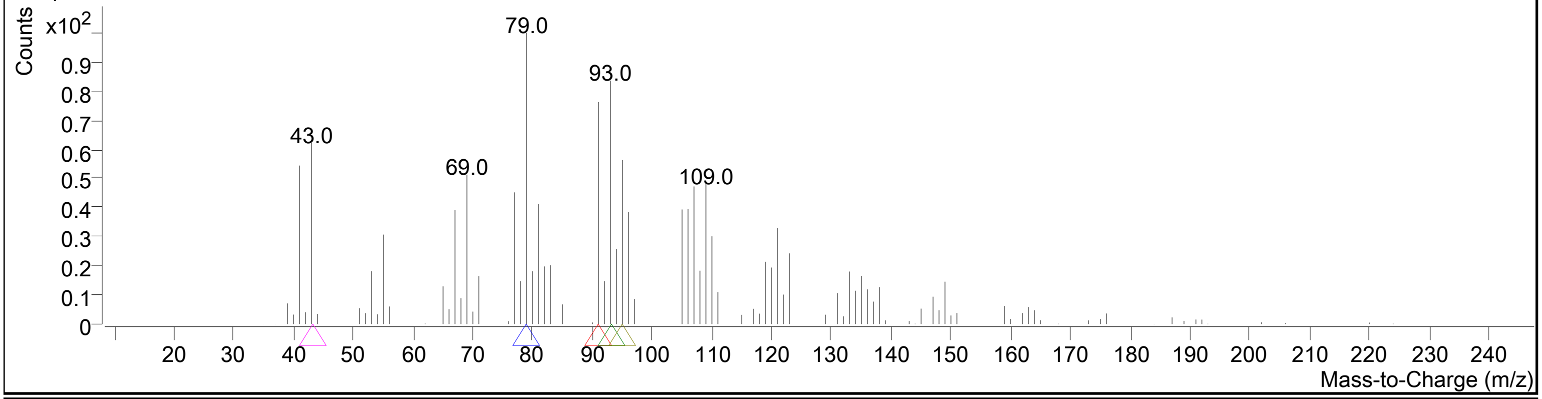

Caryophyllene oxide (NIST17.L)

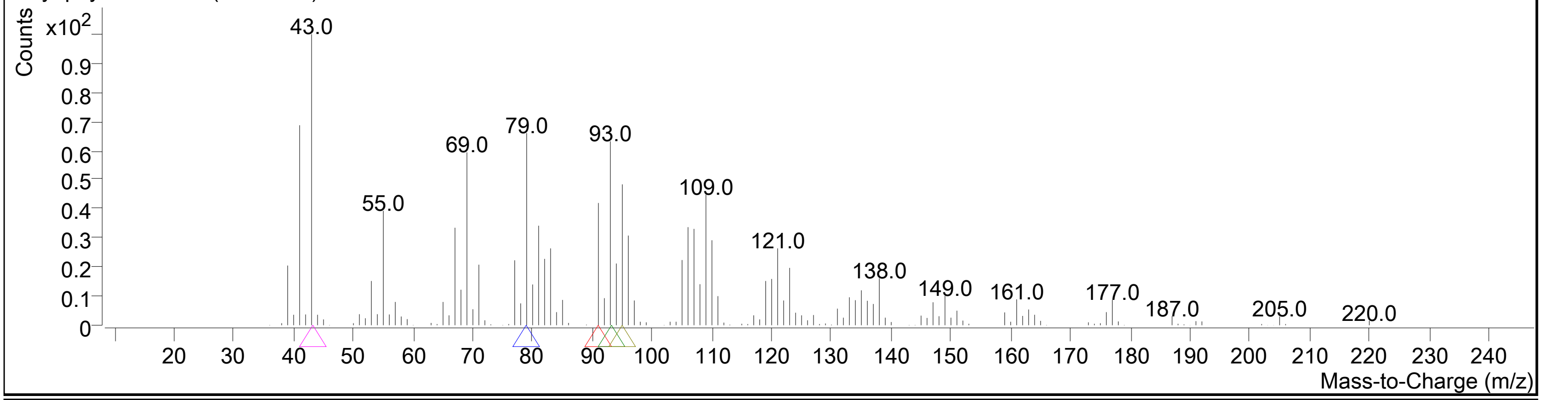

+ Scan (17.3847-17.7979 min, 50 scans) Sample 15.D

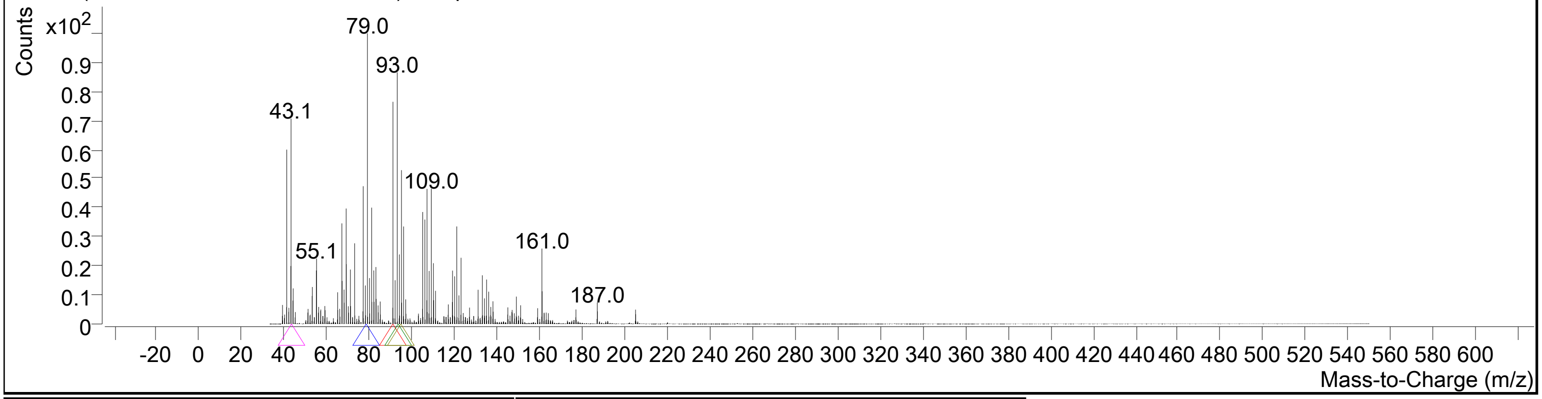

Component RT: 17.4597

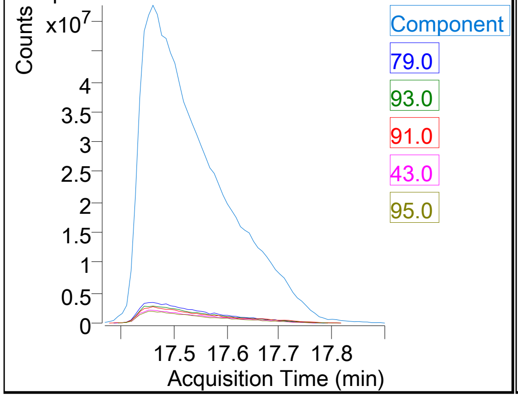

EIC Peaks

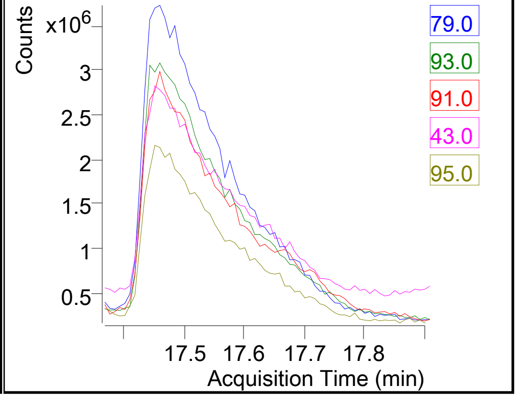

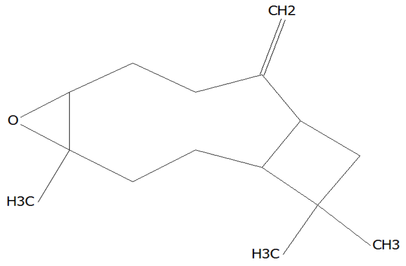

Component RT	Compound Name	Component Area	Match Factor	CAS#	Formula	Estimated Conc.
18.4895	(1R,3E,7E,11R)-1,5,5,8-Tetramethyl-12-oxabicyclo[9.1.0]dodeca-3,7-diene	115334471.5	78.6	19888-34-7	C15H24O	

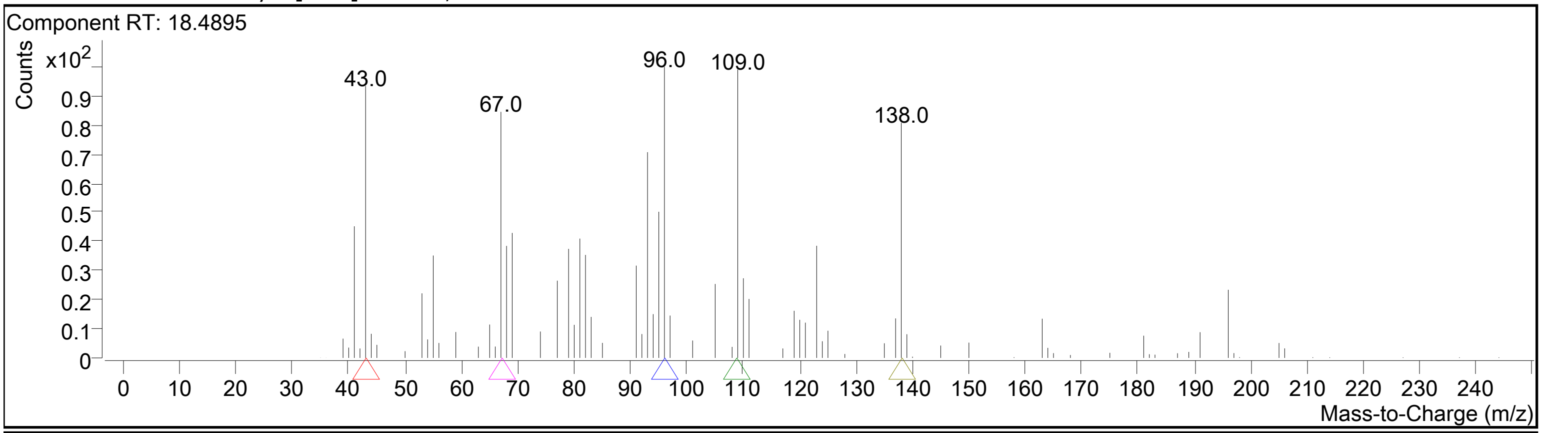

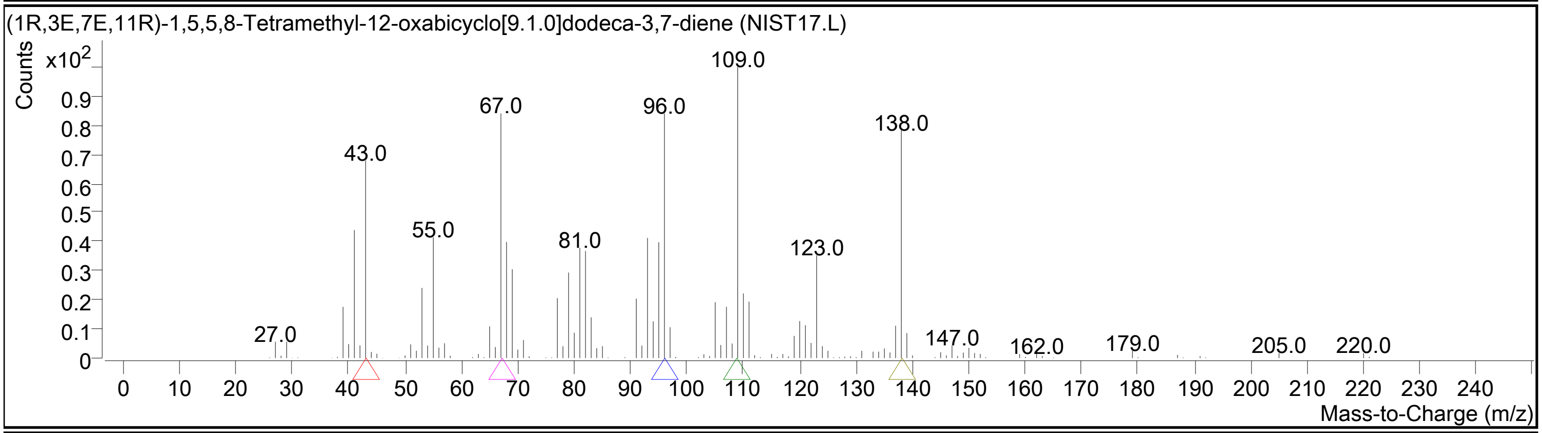

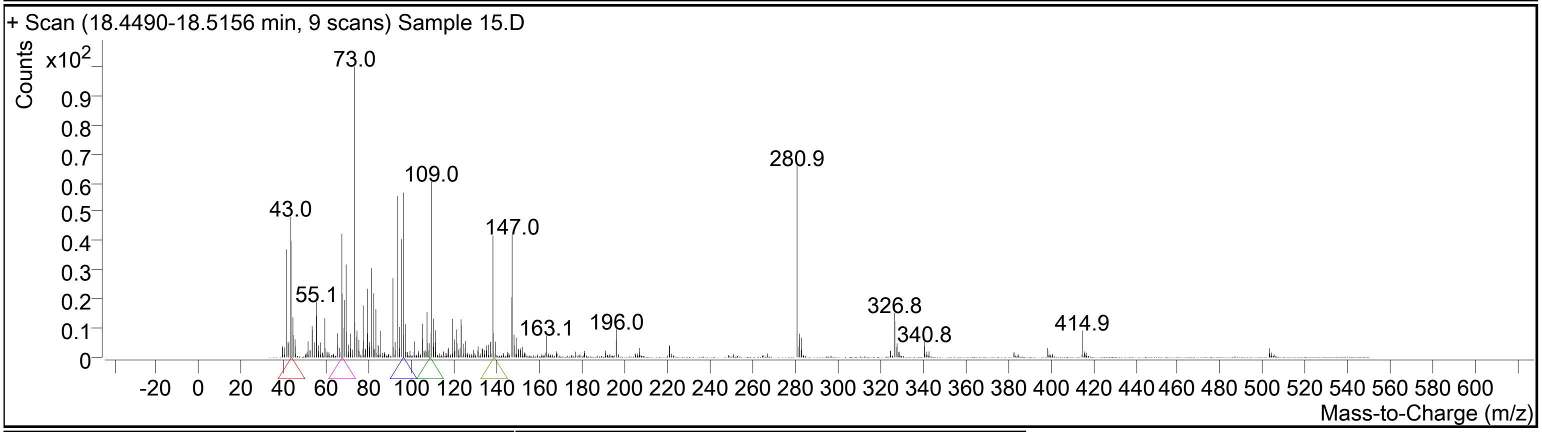

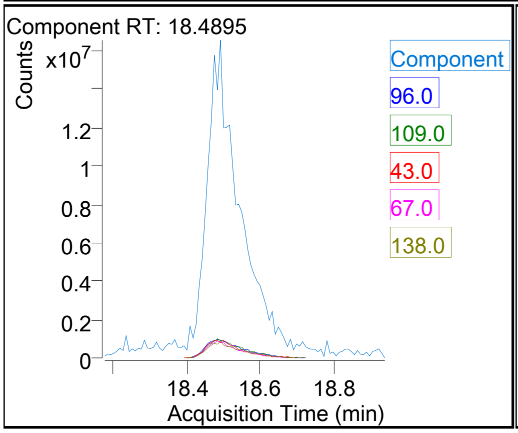

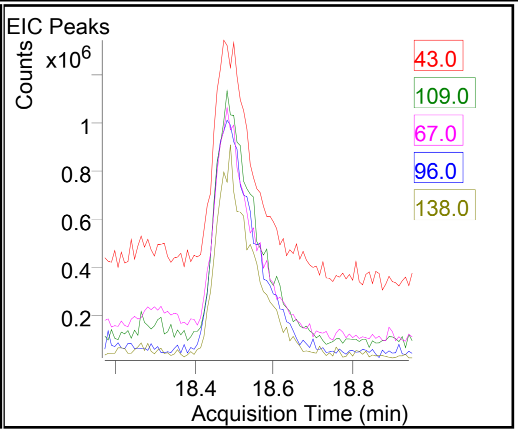

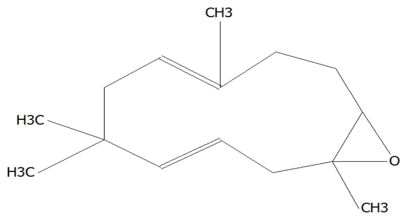

Library Search Results - NonTarget Hits with Details

Agilent Technologies

Component RT	Compound Name	Component Area	Match Factor	CAS#	Formula	Estimated Conc.
19.3722	11,11-Dimethyl-4,8-dimethylenebicyclo[7.2.0]undecan-3-ol	341386339.2	92.8	79580-01-1	C15H24O	

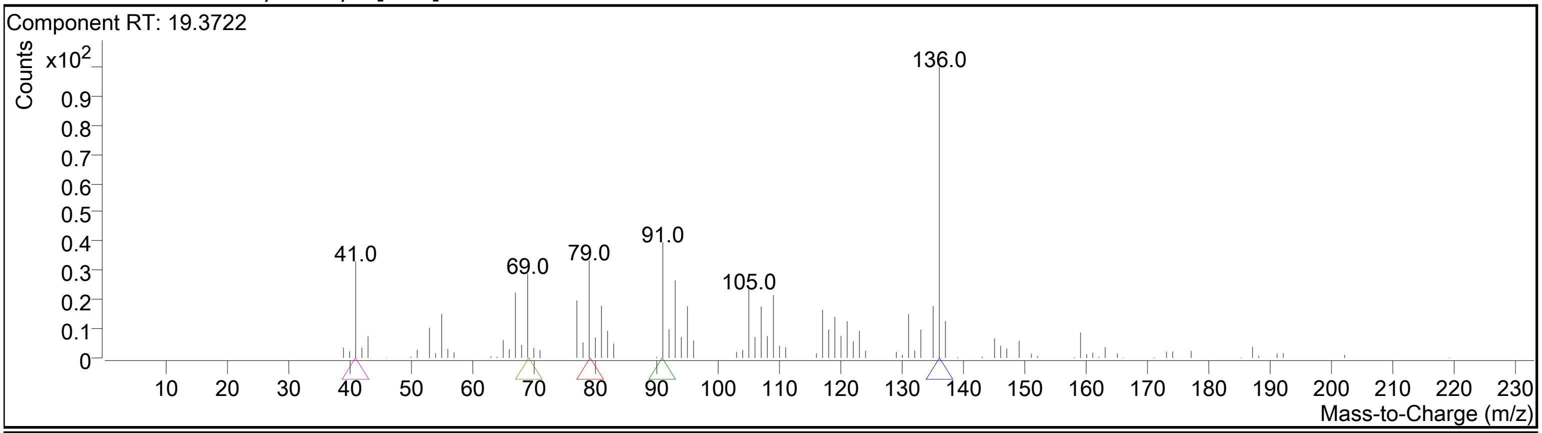

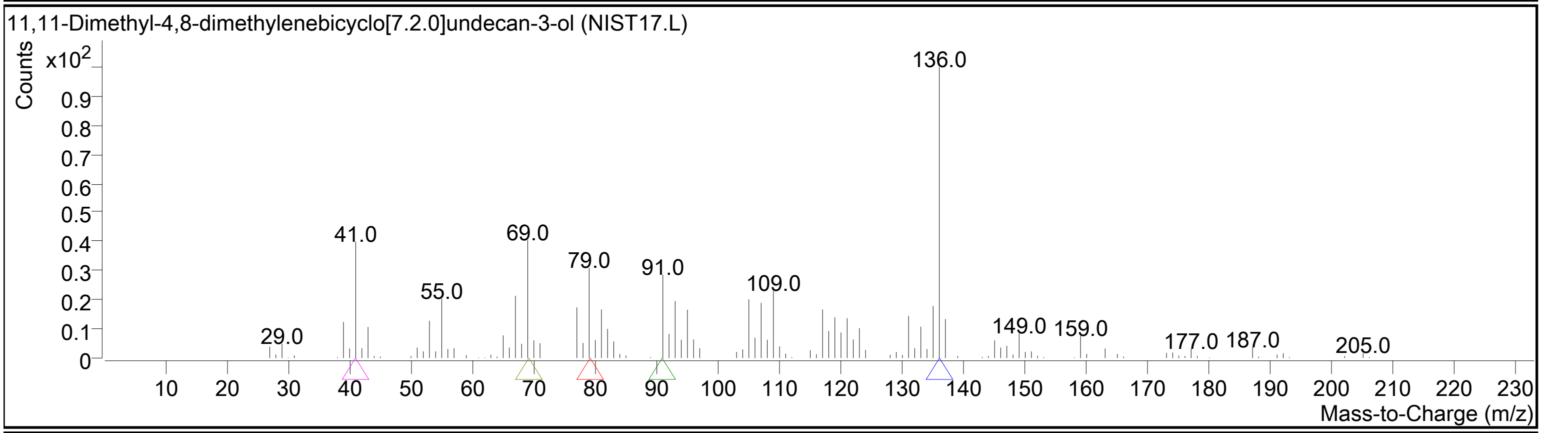

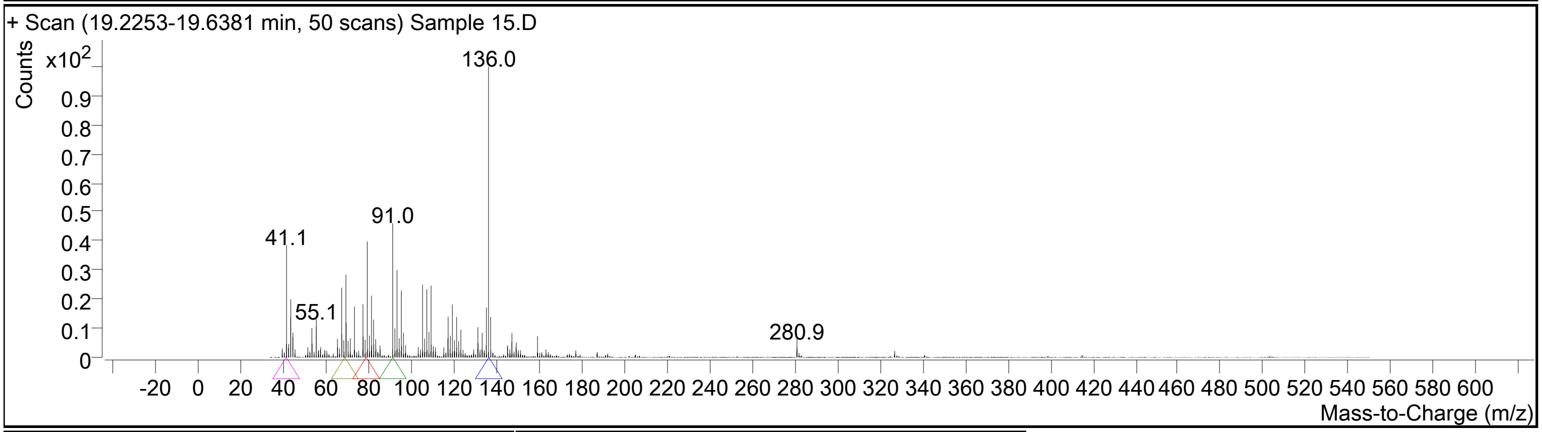

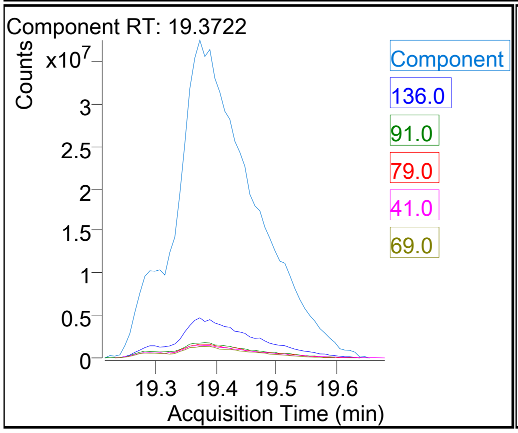

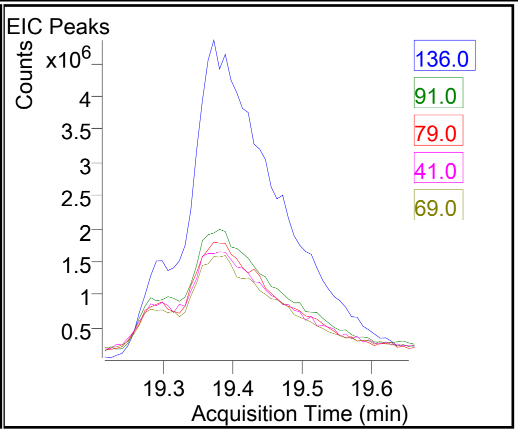

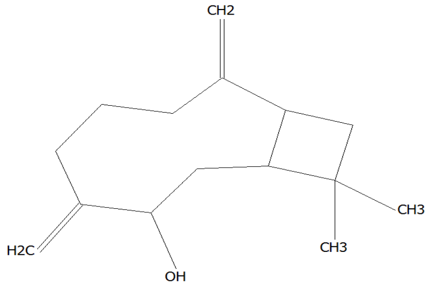

Generated at 12:24 PM on 2/25/2021

Agilent Technologies

Component RT	Compound Name	Component Area	Match Factor	CAS#	Formula	Estimated Conc.
19.7293	2-Butanone, 4-(4-hydroxy-3-methoxyphenyl)-	251489371.8	93.9	122-48-5	C11H14O3	

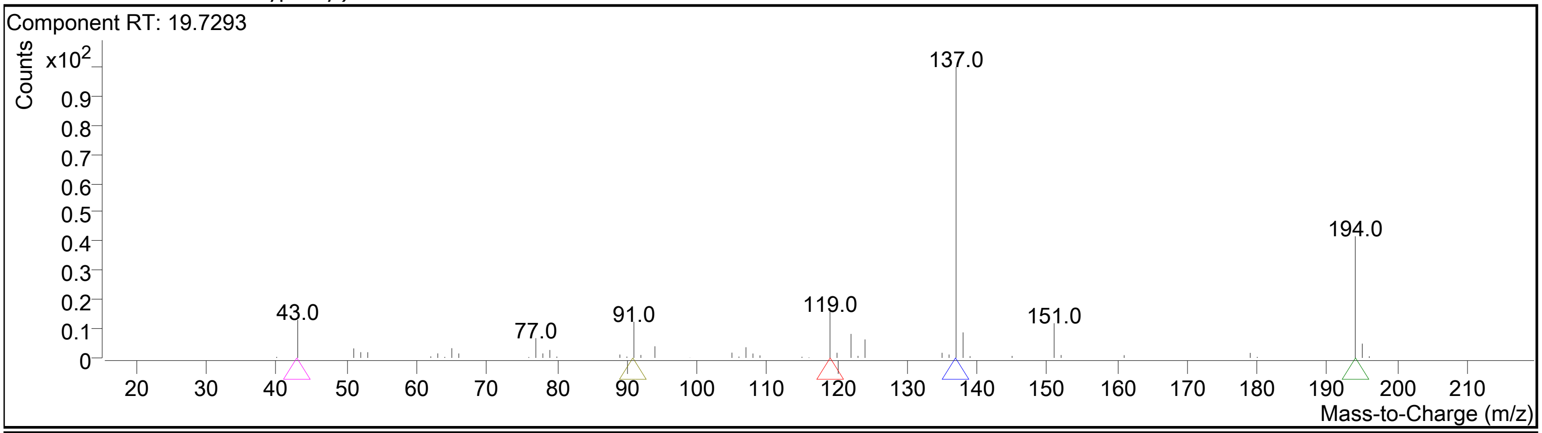

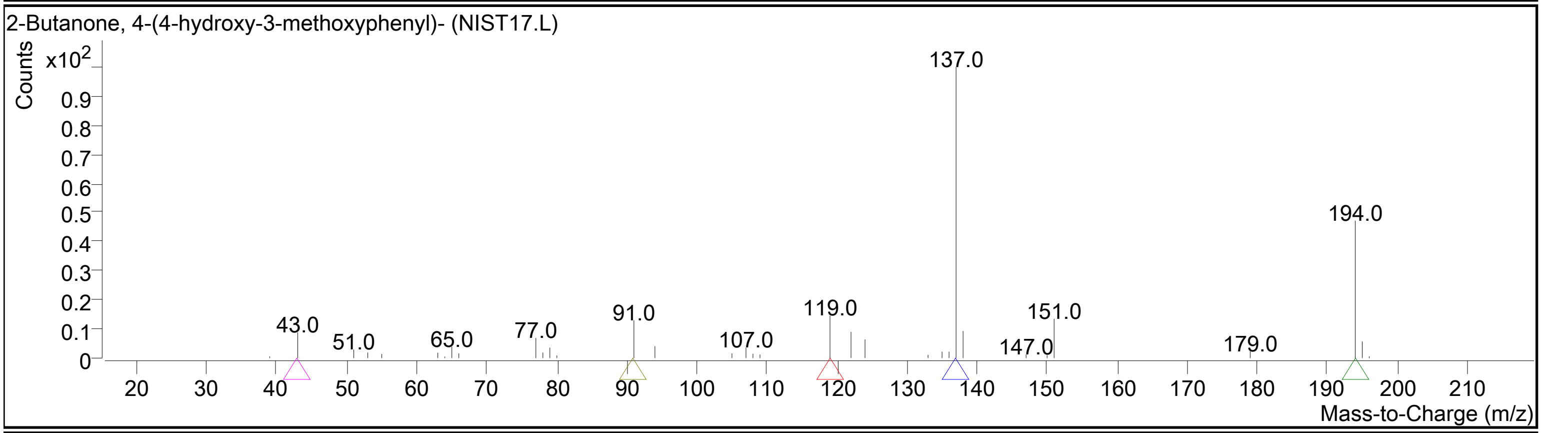

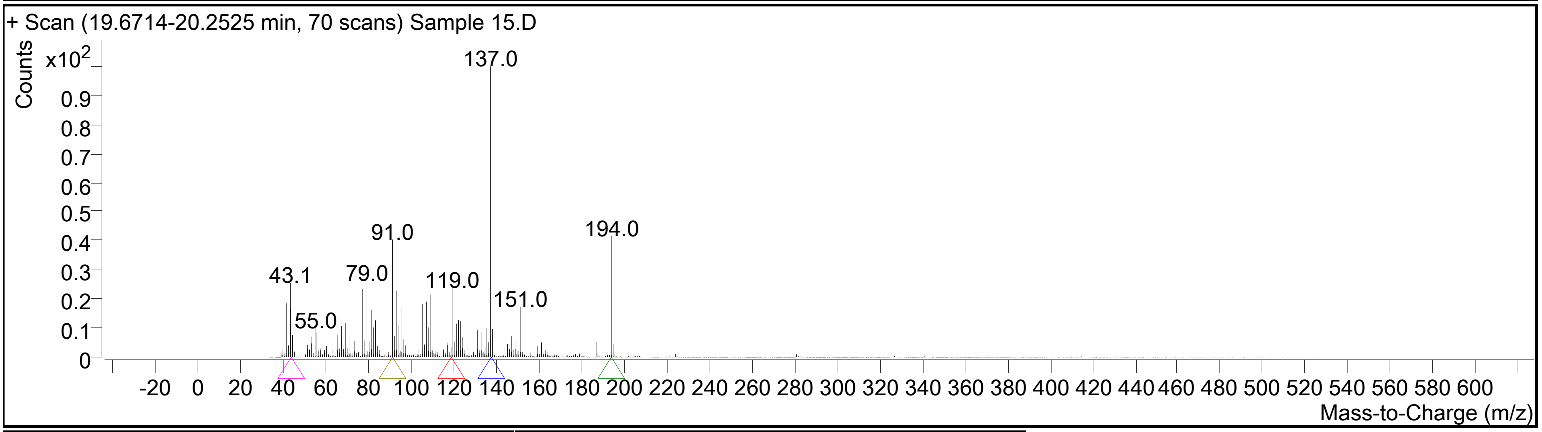

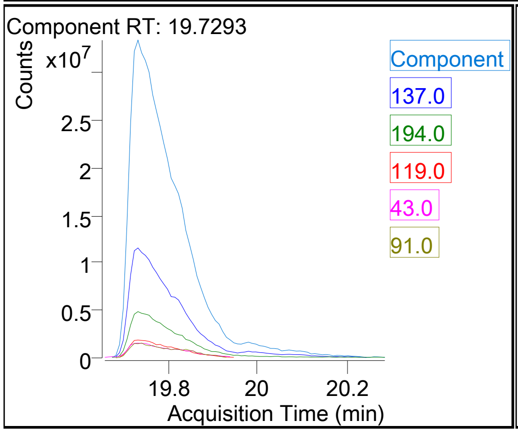

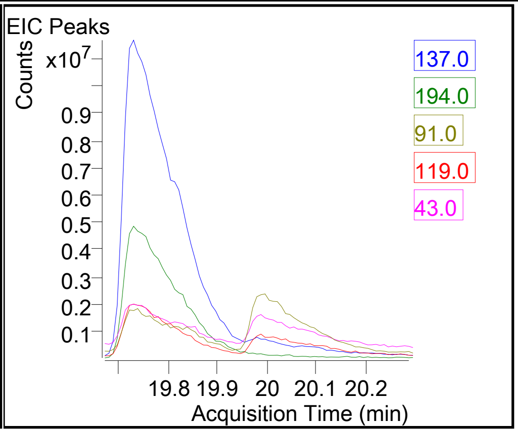

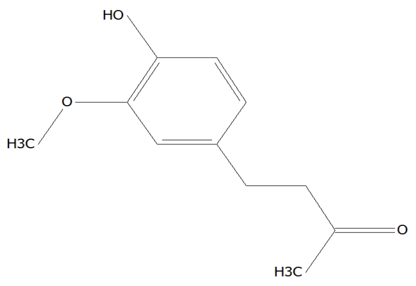

Generated at 12:24 PM on 2/25/2021

Component RT	Compound Name	Component Area	Match Factor	CAS#	Formula	Estimated Conc.
19.8476	Methyl jasmonate	59918382.7	82.0	1211-29-6	C13H20O3	

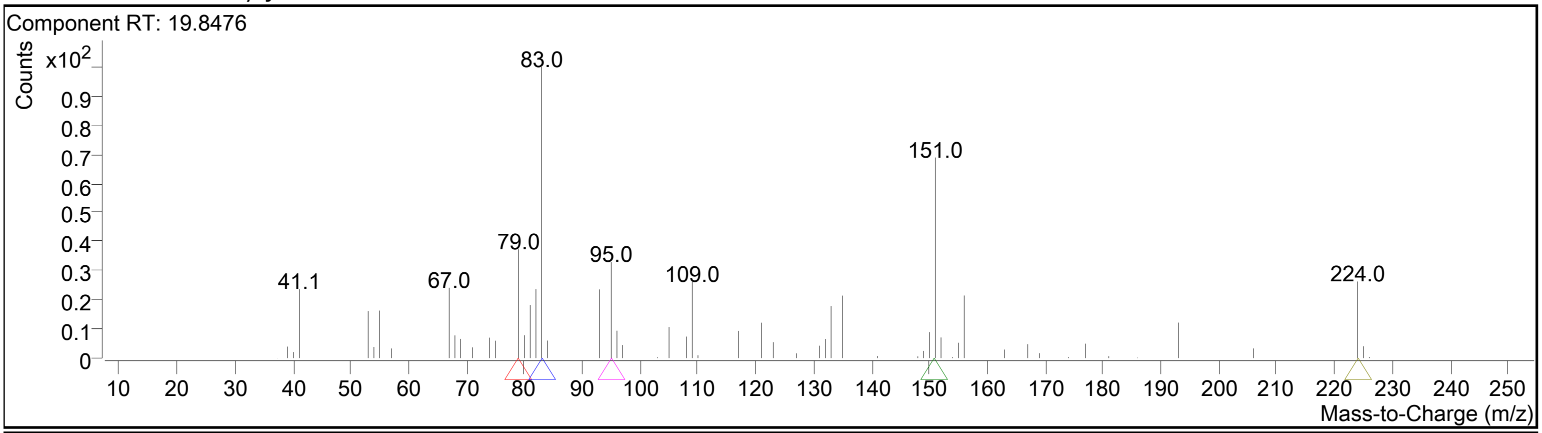

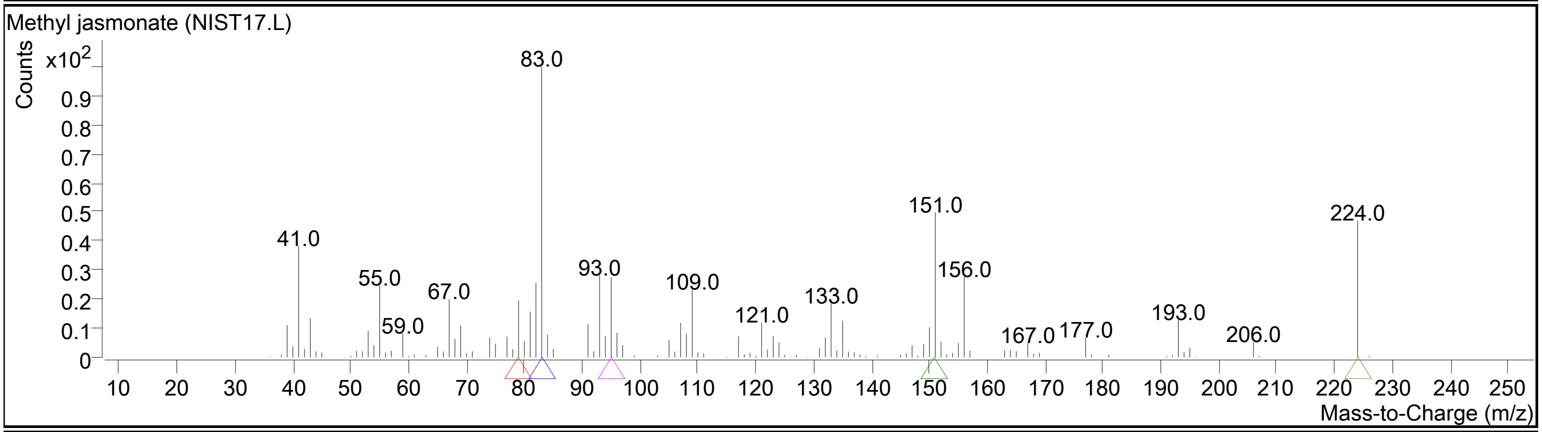

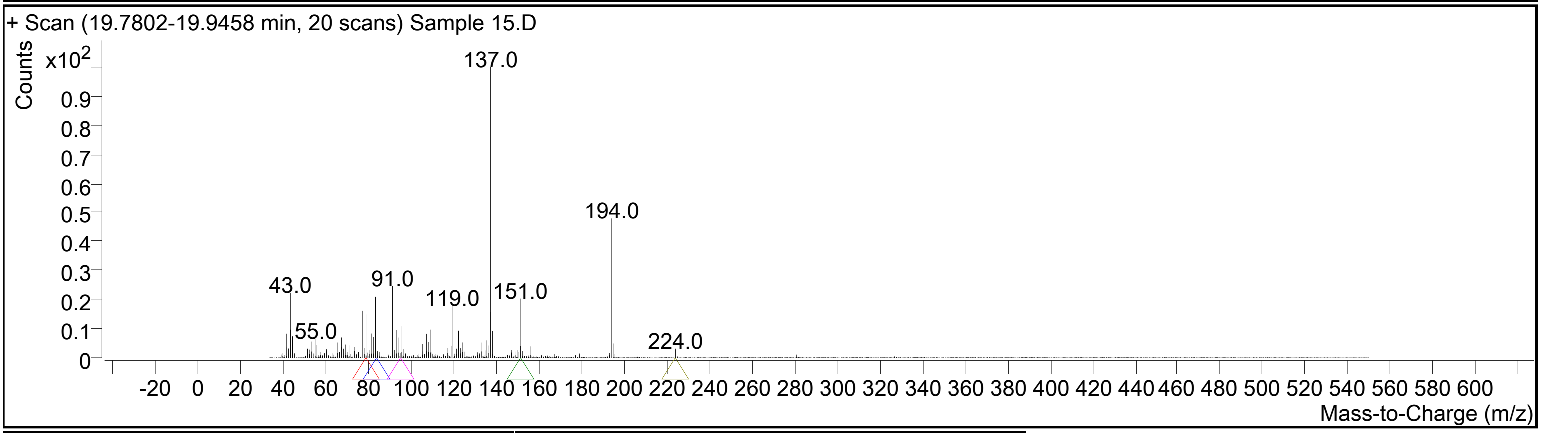

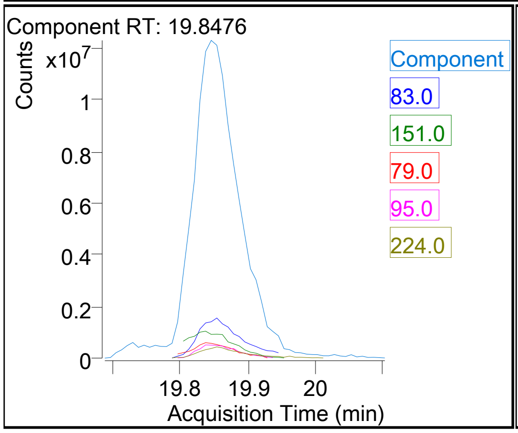

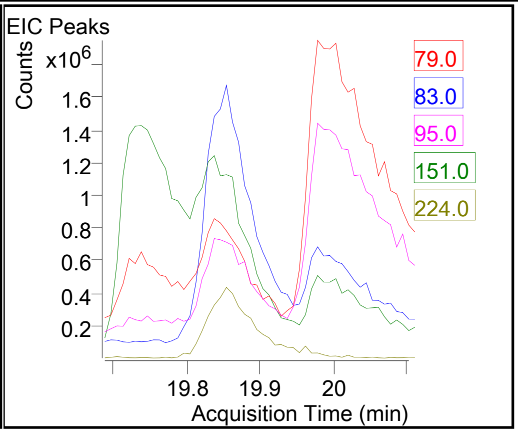

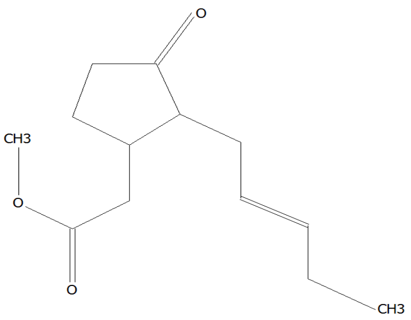

Generated at 12:24 PM on 2/25/2021

Agilent Technologies

Component RT	Compound Name	Component Area	Match Factor	CAS#	Formula	Estimated Conc.
19.9903	Caryophyllene oxide	345099318.1	85.9	1139-30-6	C15H24O	

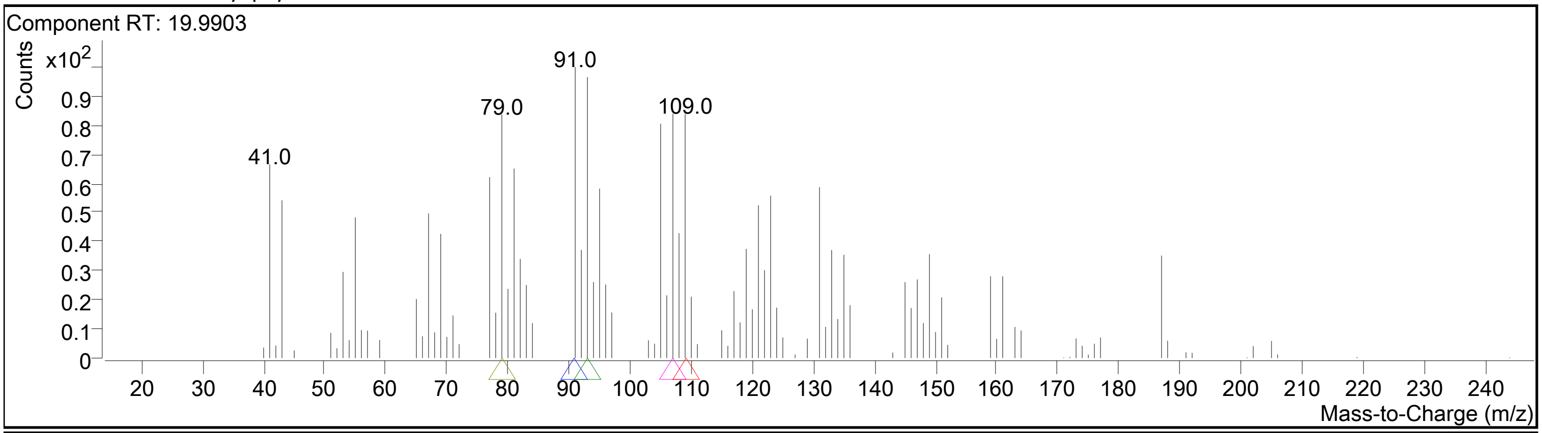

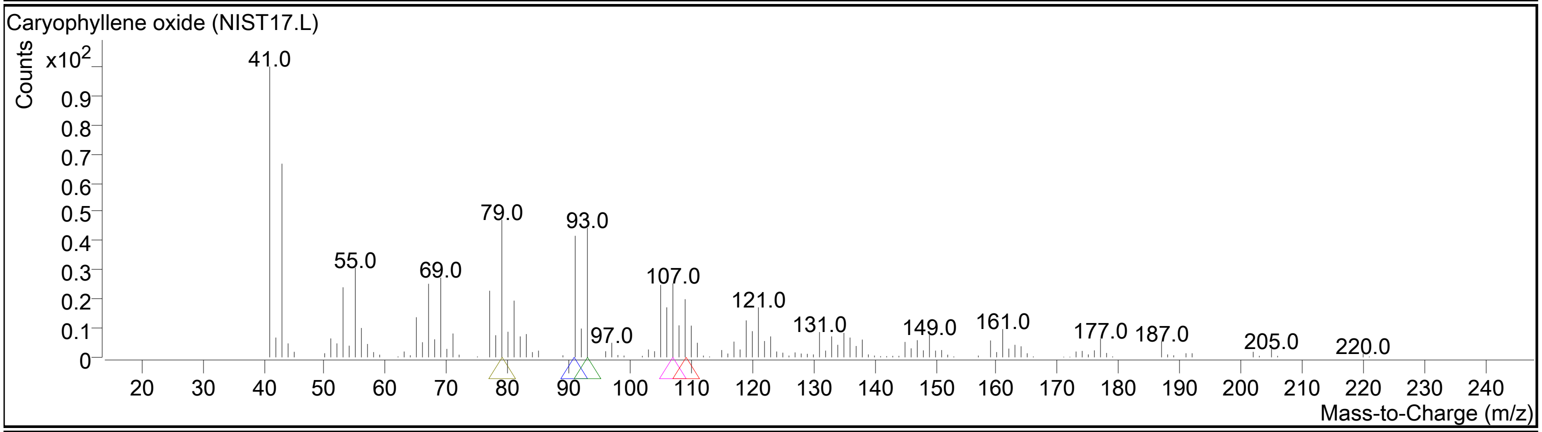

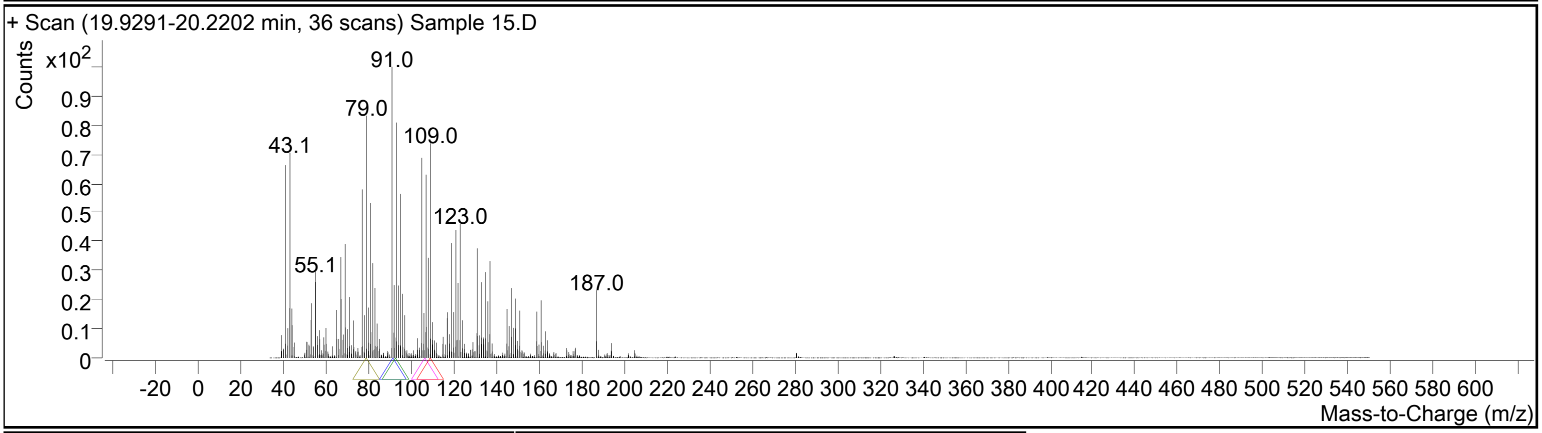

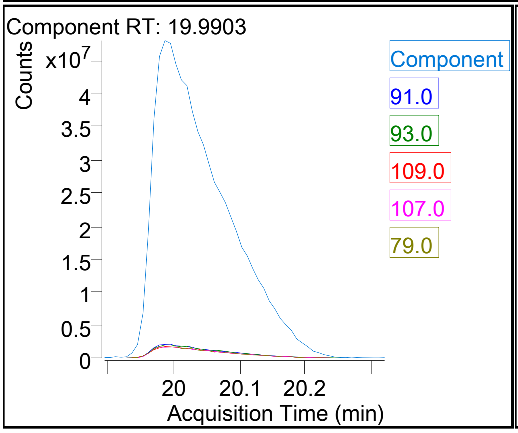

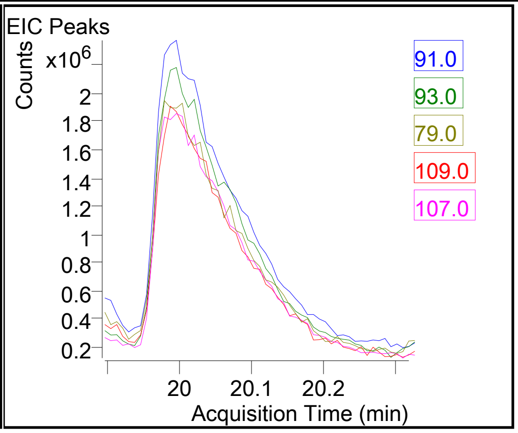

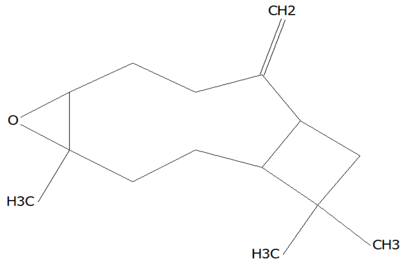

Component RT	Compound Name	Component Area	Match Factor	CAS#	Formula	Estimated Conc.
20.4148	Caryophyllene oxide	247469818.7	87.0	1139-30-6	C15H24O	

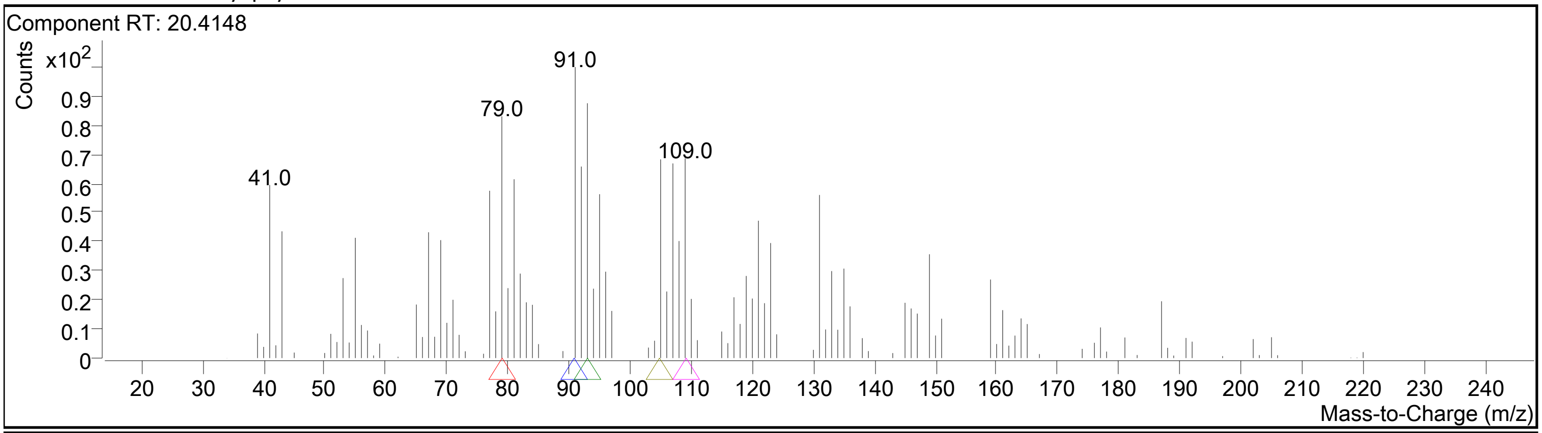

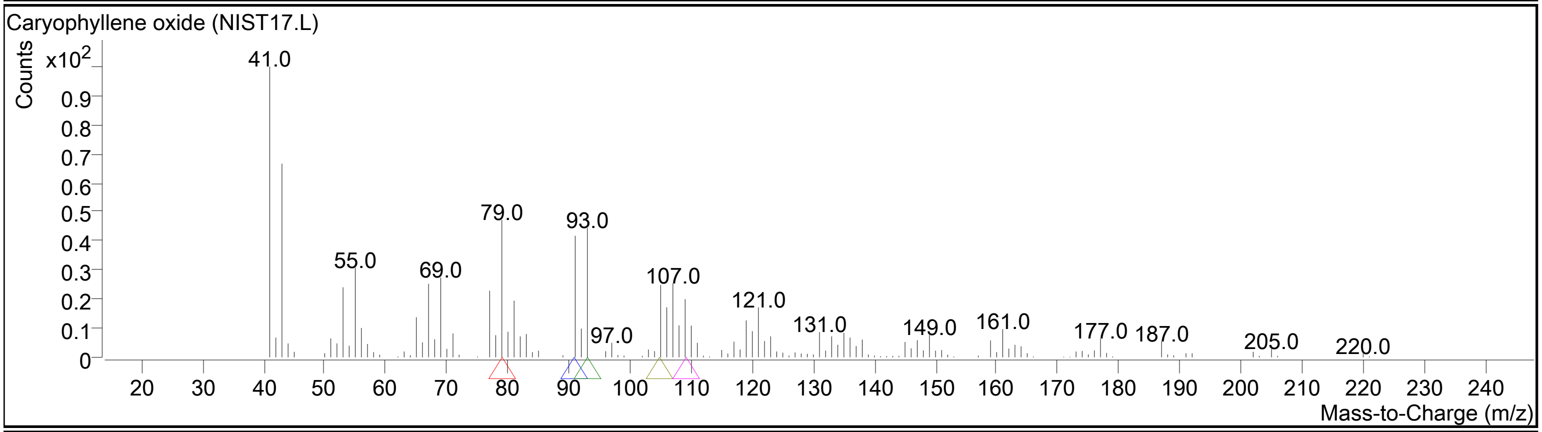

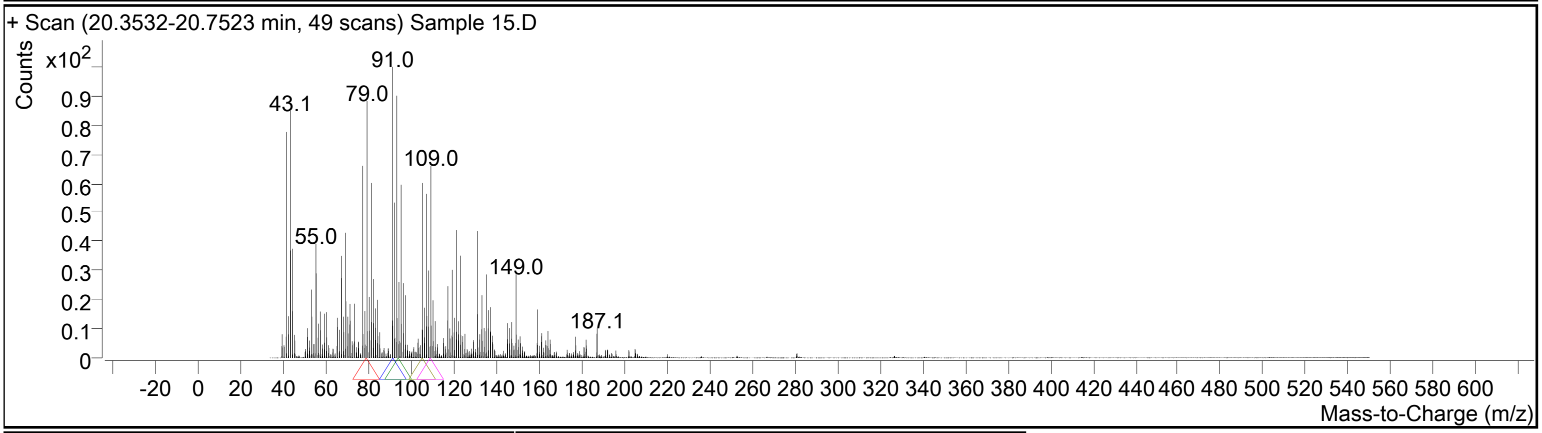

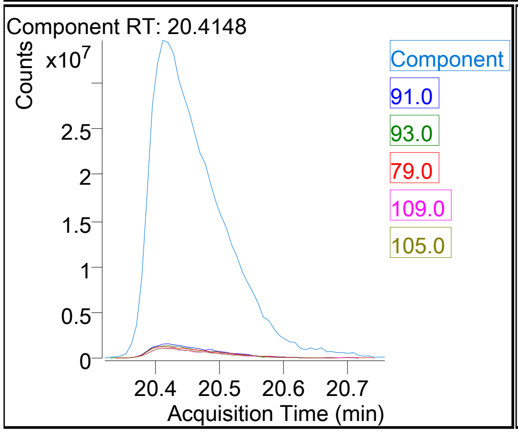

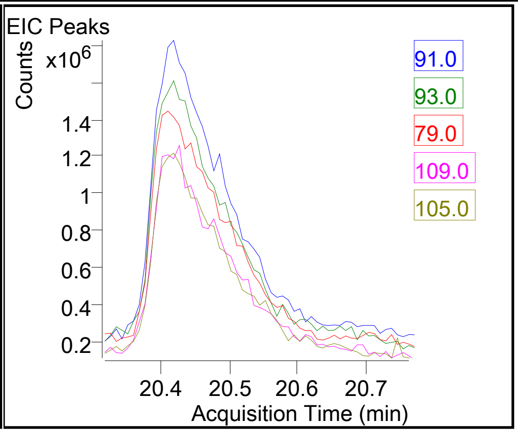

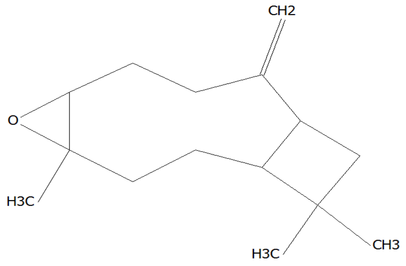

Component RT	Compound Name	Component Area	Match Factor	CAS#	Formula	Estimated Conc.
20.7426	L-Leucine, N-methyl-N-(but-3-yn-1-yloxycarbonyl)-, hexadecyl ester	1461943.0	67.9	1000392-38-3	C28H51NO4	

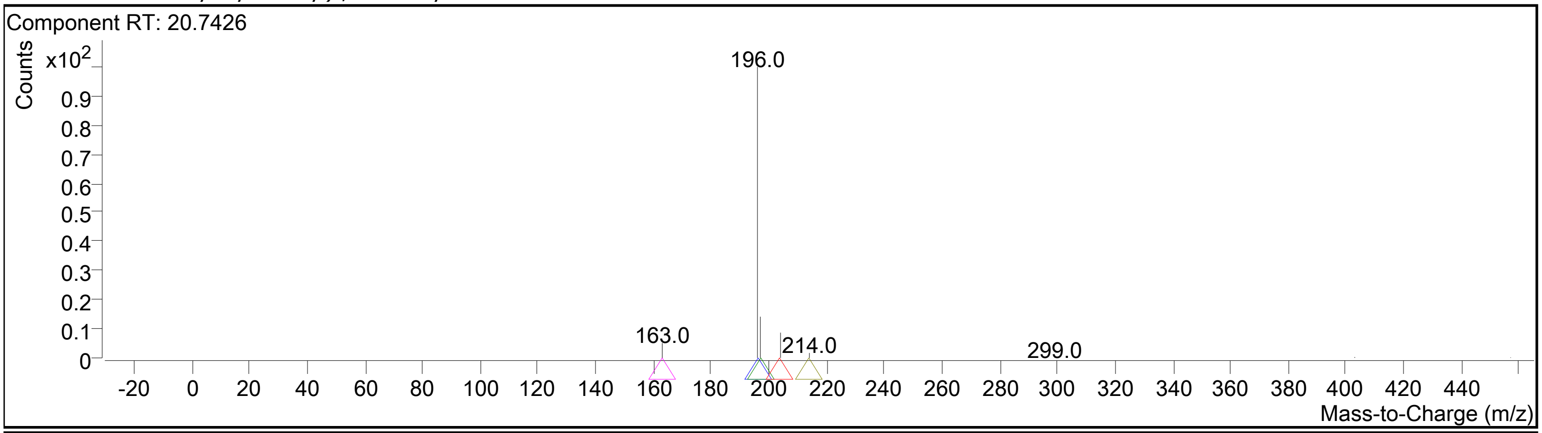

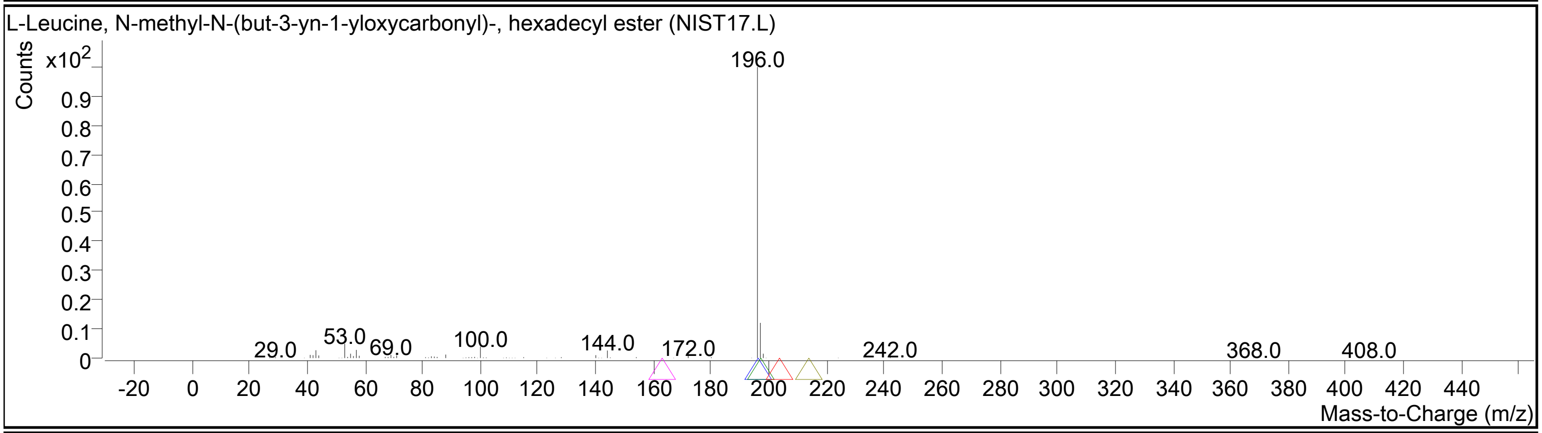

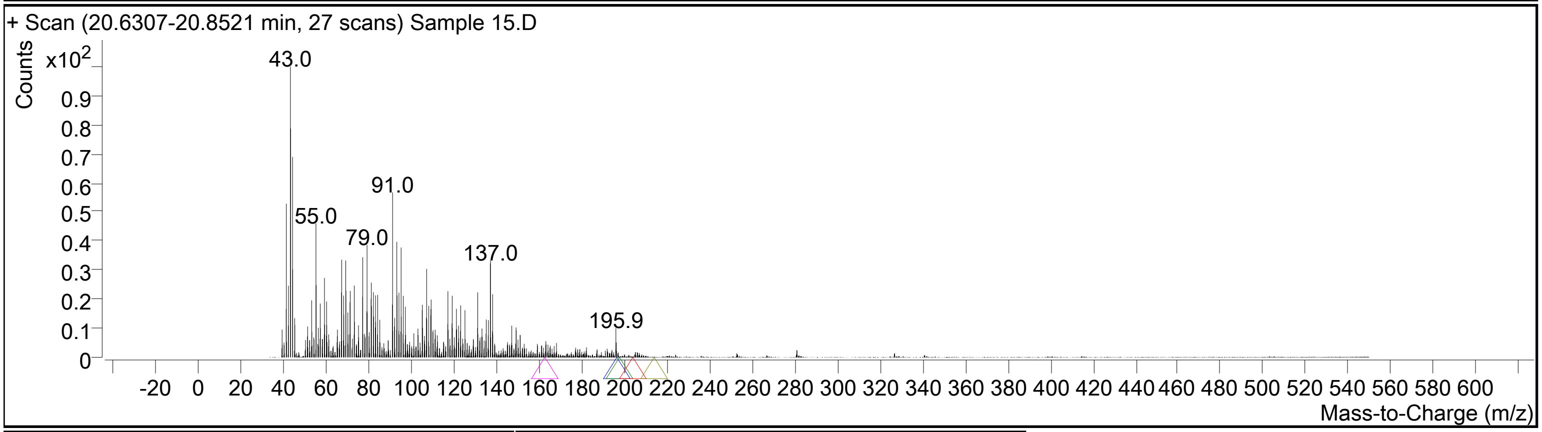

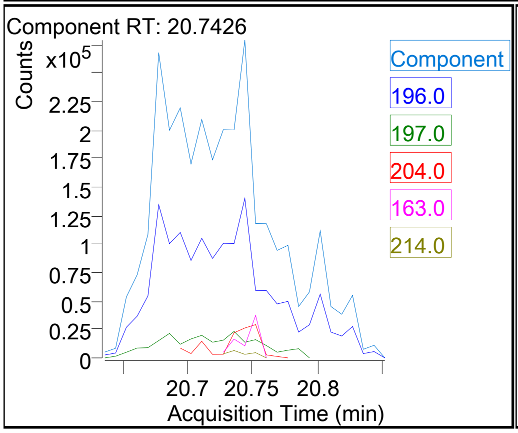

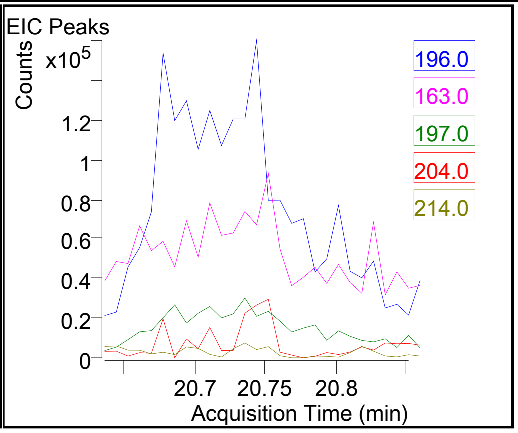

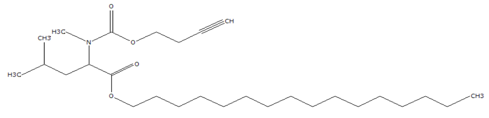

Component RT	Compound Name	Component Area	Match Factor	CAS#	Formula	Estimated Conc.
22.2324	(3E,10Z)-Oxacyclotrideca-3,10-diene-2,7-dione	17105796.8	70.8	144403-15-6	C12H16O3	

Component RT: 22.2324

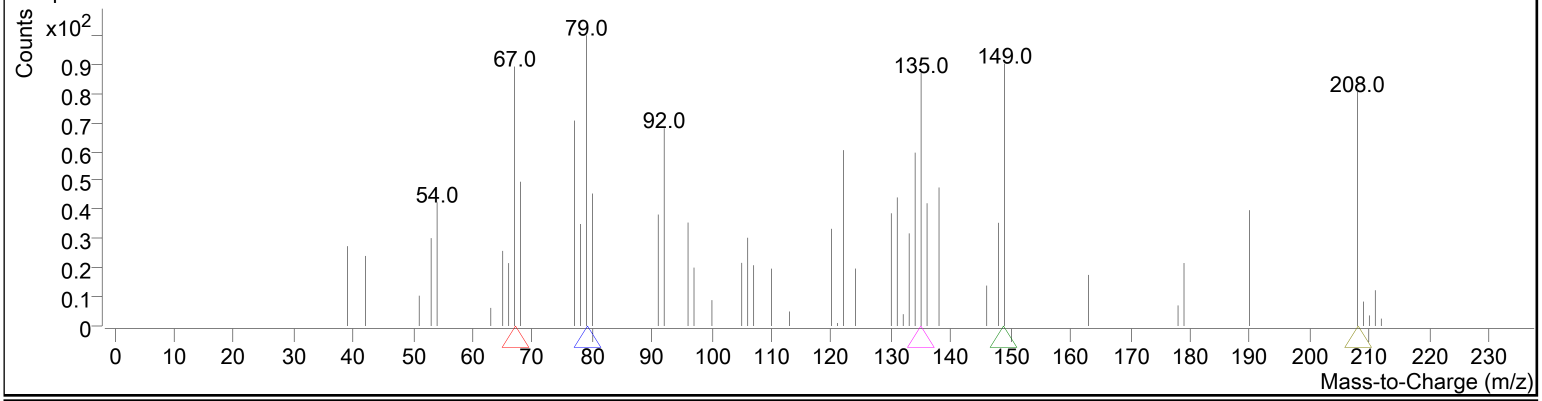

(3E,10Z)-Oxacyclotrideca-3,10-diene-2,7-dione (NIST17.L)

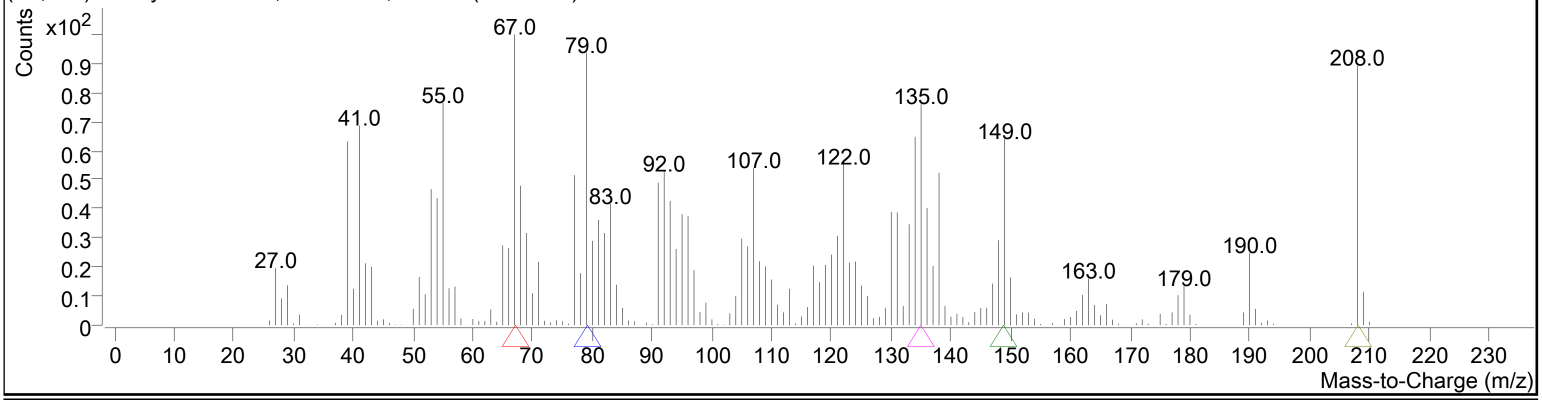

+ Scan (22.2036-22.2406 min, 5 scans) Sample 15.D

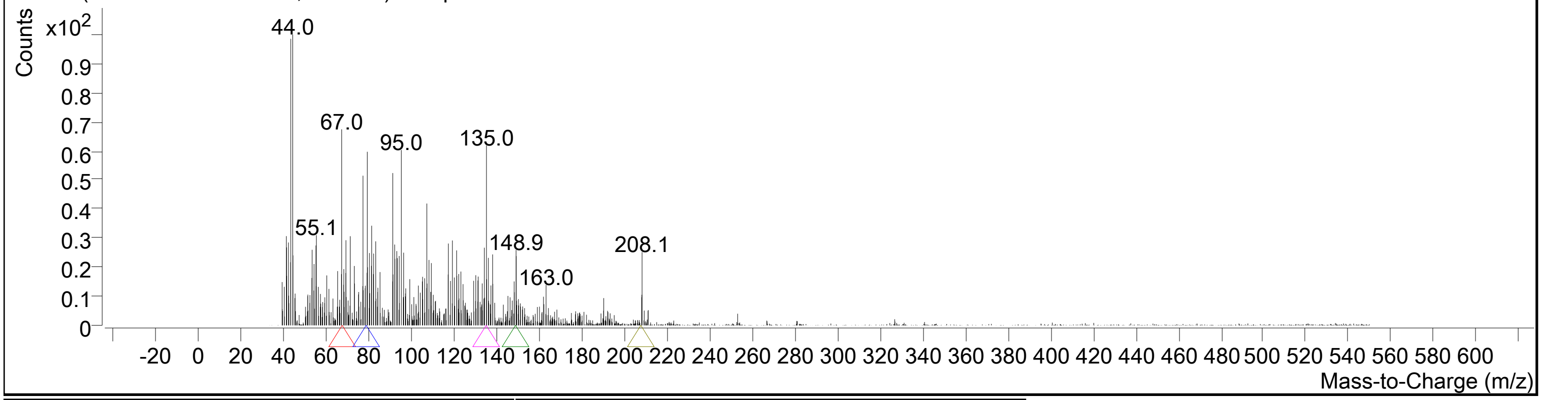

Component RT: 22.2324

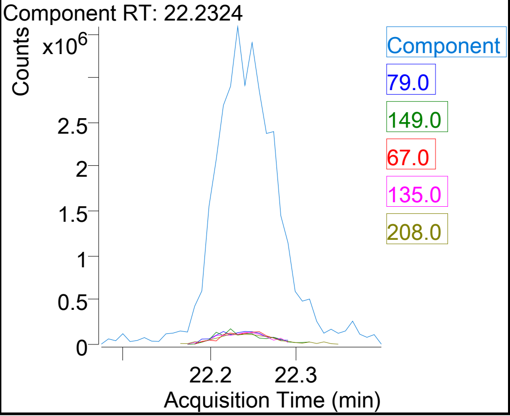

EIC Peaks

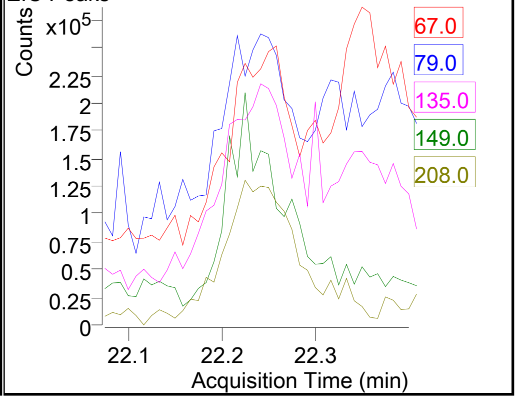

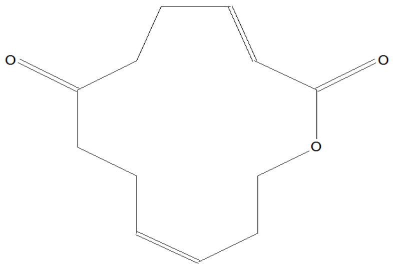

 Generated at 12:24 PM on 2/25/2021

Component RT	Compound Name	Component Area	Match Factor	CAS#	Formula	Estimated Conc.
22.3419	2(1H)-Pyridinone, 3-hydroxy-	4779062.5	66.2	16867-04-2	C5H5NO2	

Component RT: 22.3419

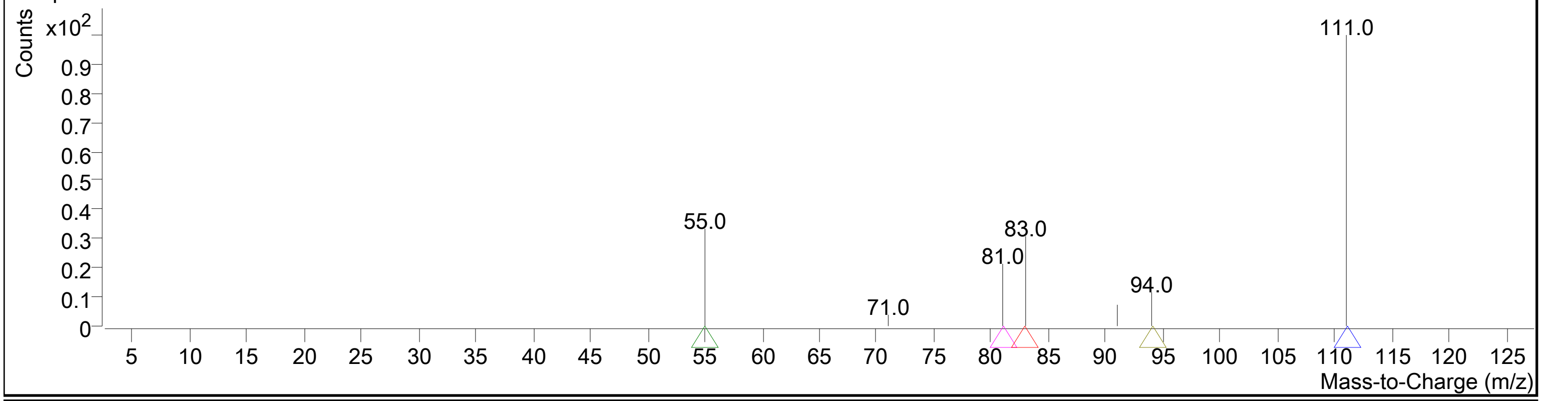

2(1H)-Pyridinone, 3-hydroxy- (NIST17.L)

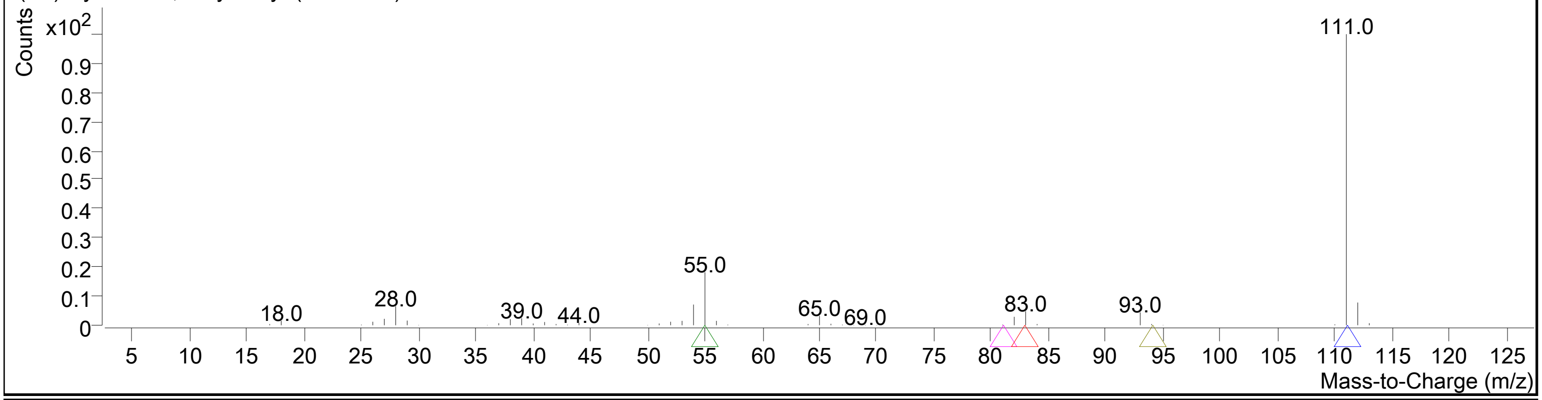

+ Scan (22.2701-22.5045 min, 29 scans) Sample 15.D

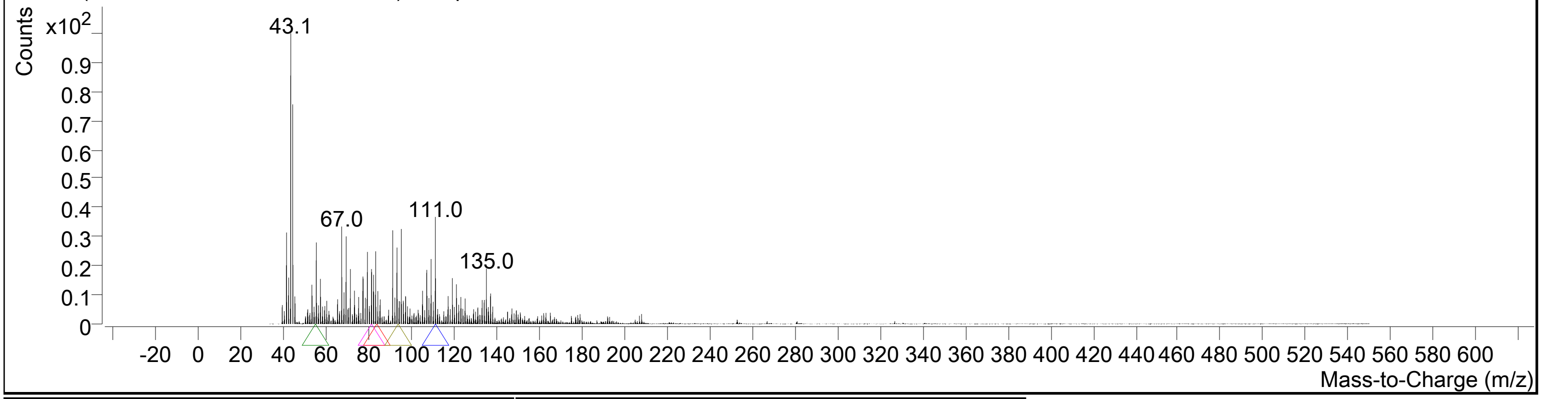

Component RT: 22.3419

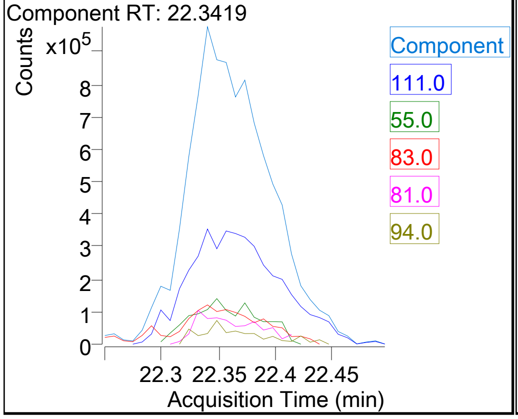

EIC Peaks

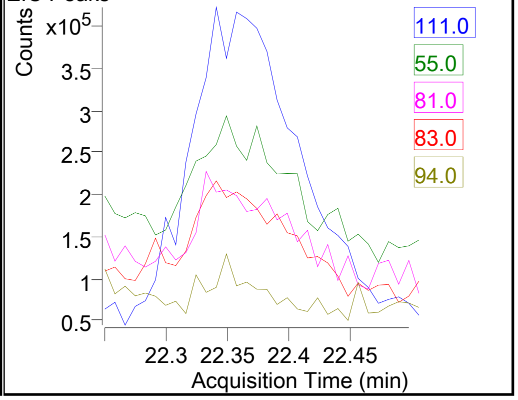

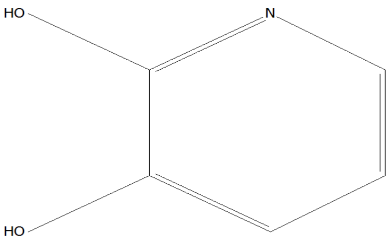

Agilent Technologies

Component RT	Compound Name	Component Area	Match Factor	CAS#	Formula	Estimated Conc.
22.3545	1,3-Cyclohexanediol, 5-methyl-2-nitro-, monoacetate (ester), [1s-(1.alpha.,2.beta.,3.alpha.,5.alpha.)]-	14946021.0	68.8	114454-85-2	C9H15NO5	

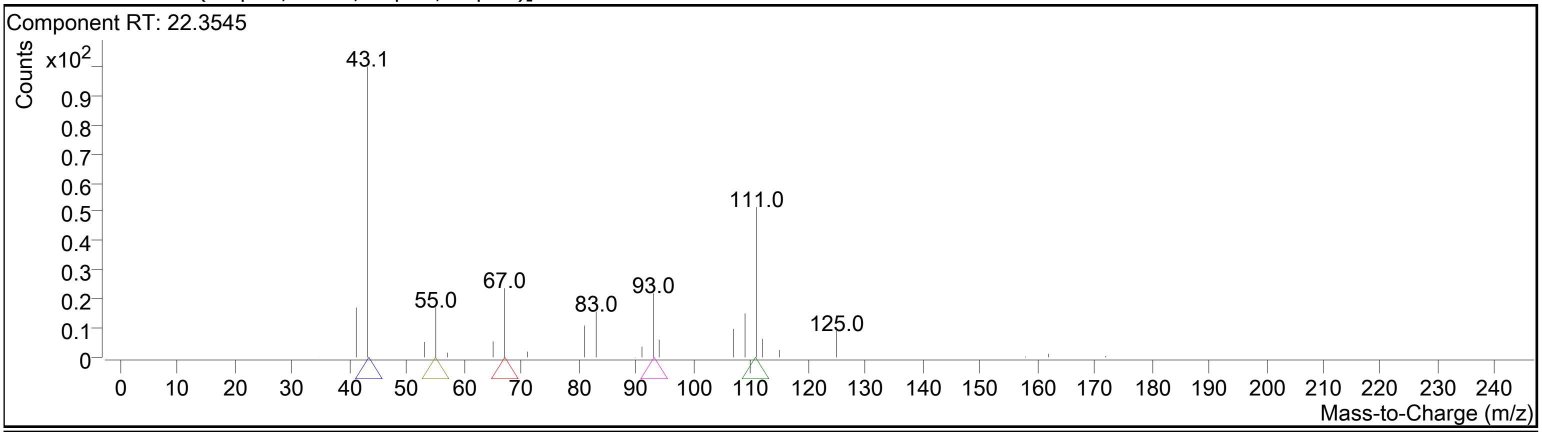

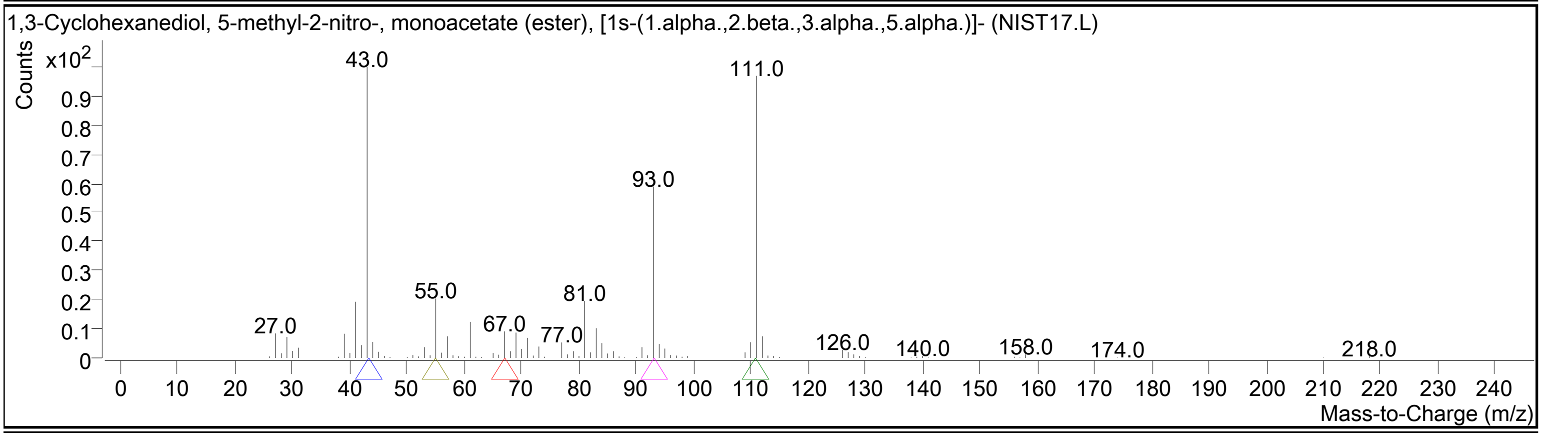

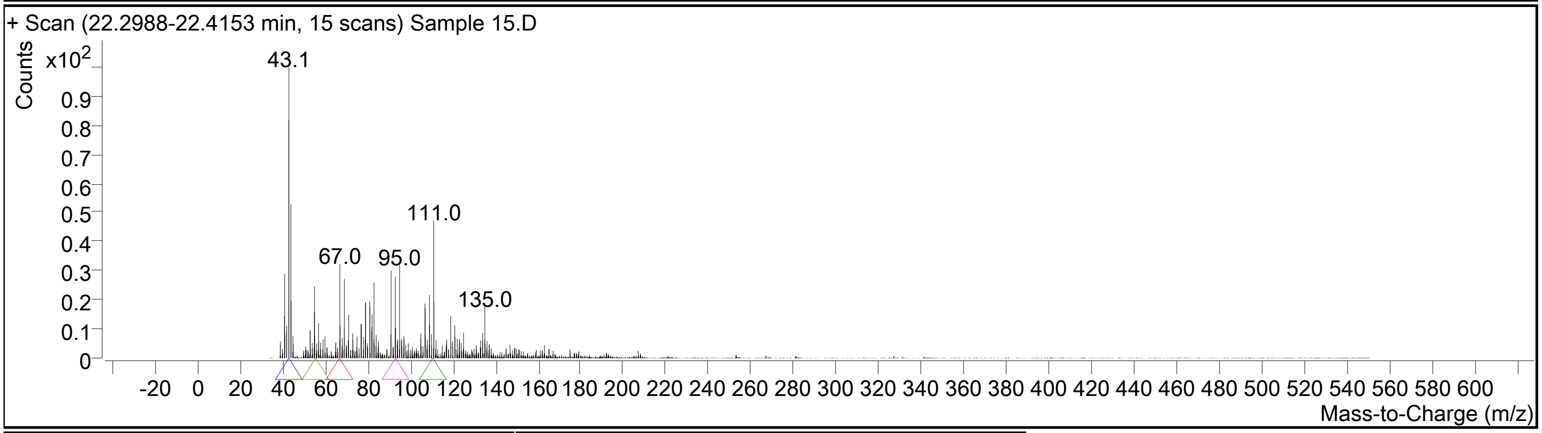

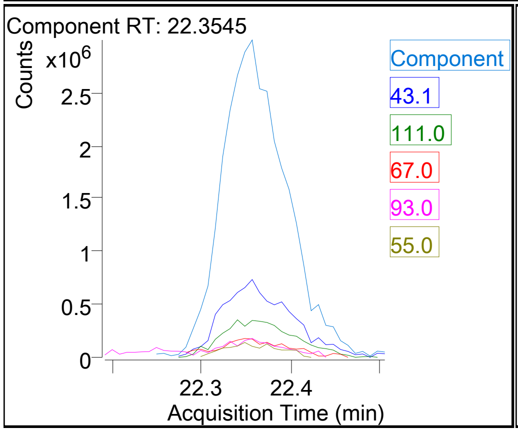

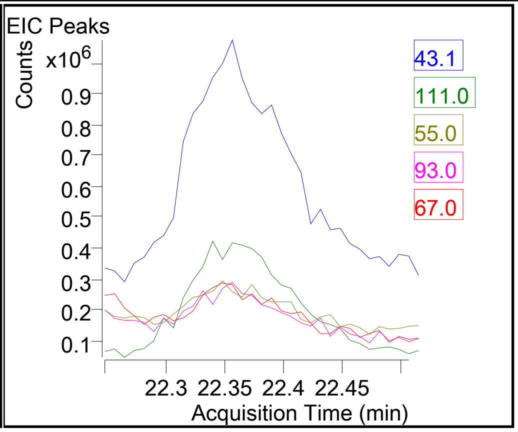

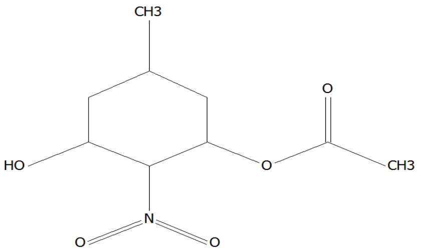

Component RT	Compound Name	Component Area	Match Factor	CAS#	Formula	Estimated Conc.
22.9996	Drim-7-en-11-ol	76372837.2	71.3	468-68-8	C15H26O	

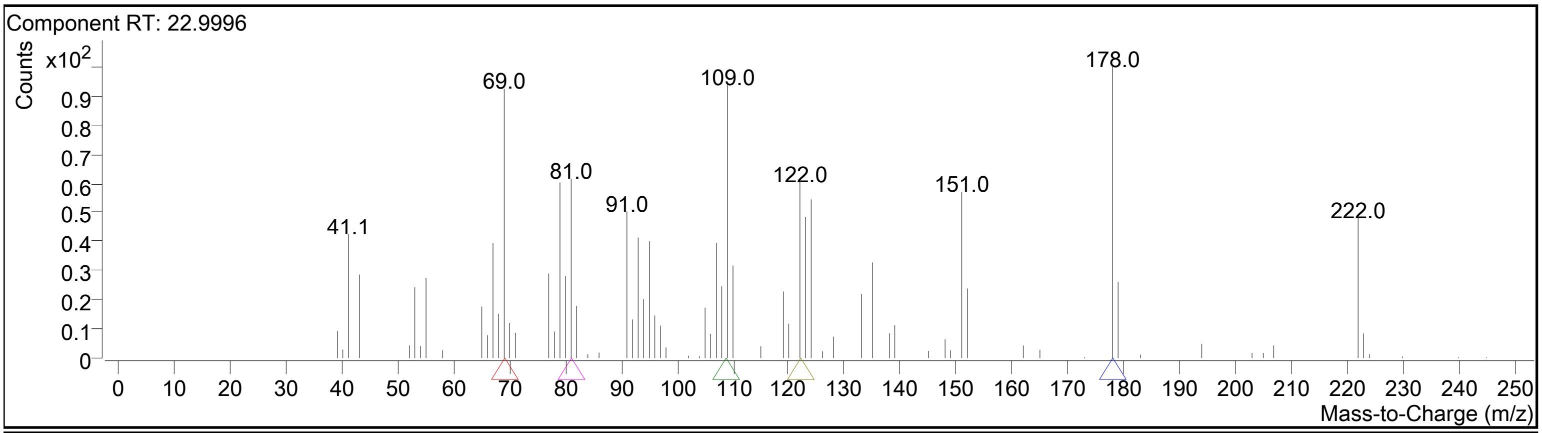

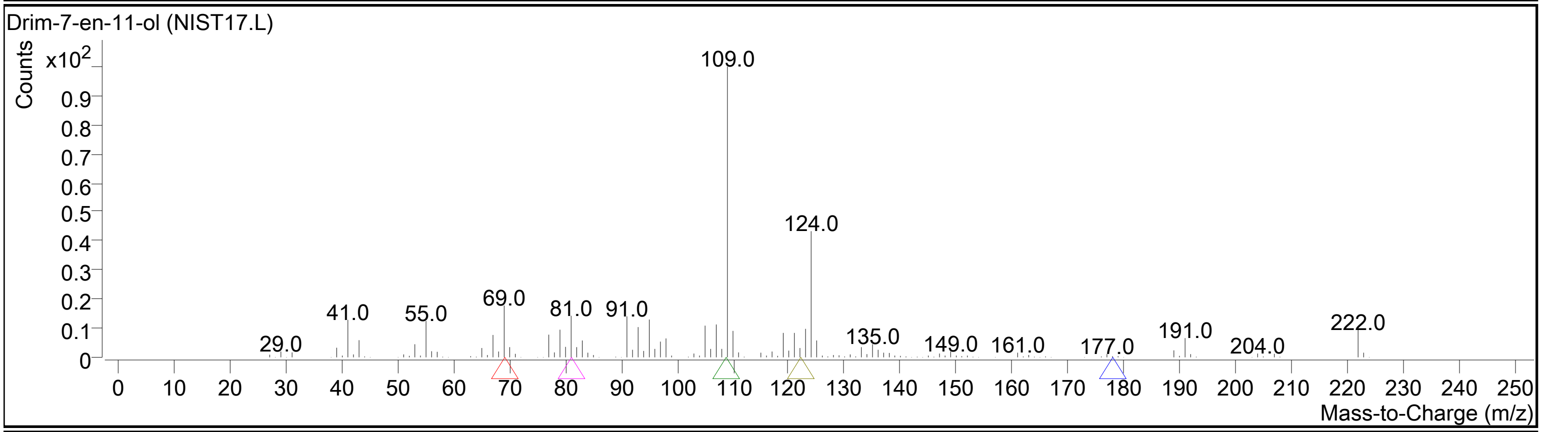

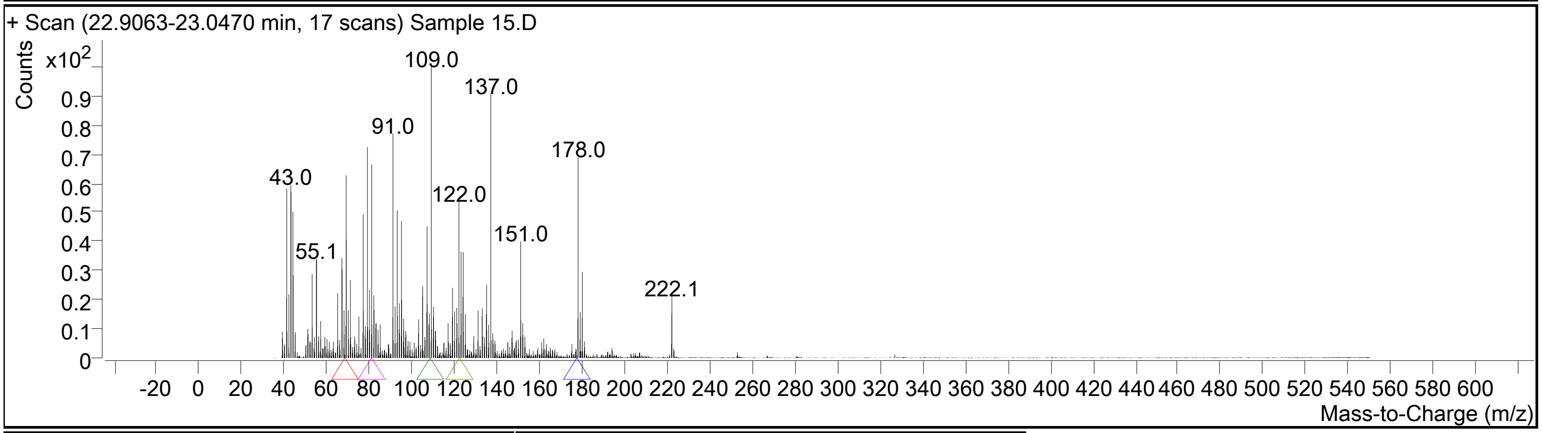

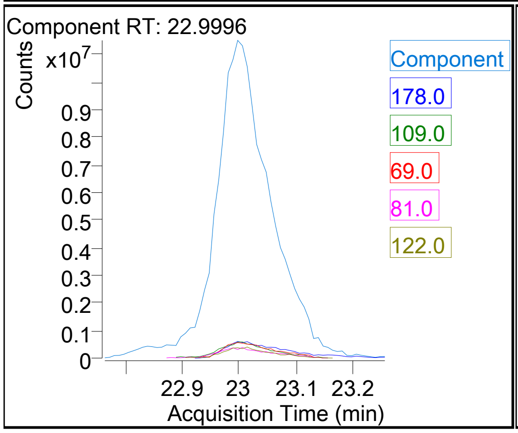

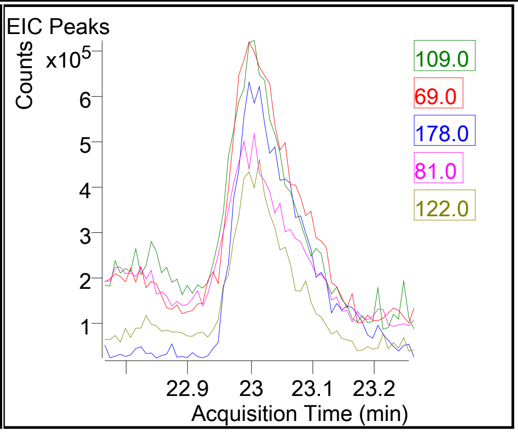

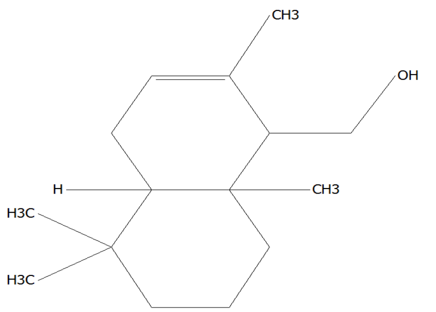

Component RT	Compound Name	Component Area	Match Factor	CAS#	Formula	Estimated Conc.
23.2402	Benzyl Benzoate	69586326.0	93.9	120-51-4	C14H12O2	

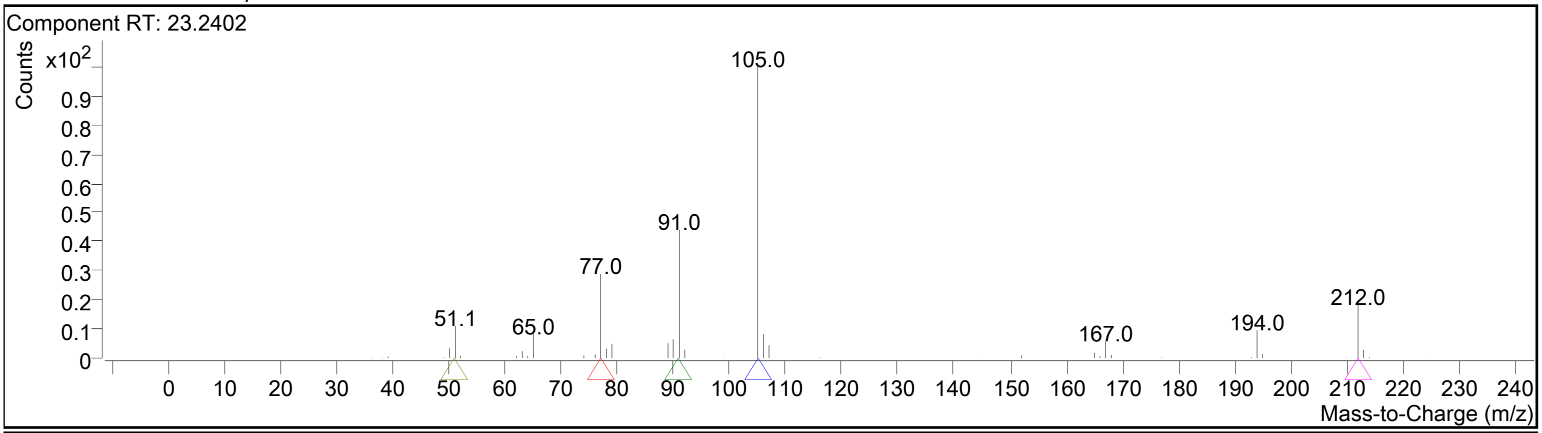

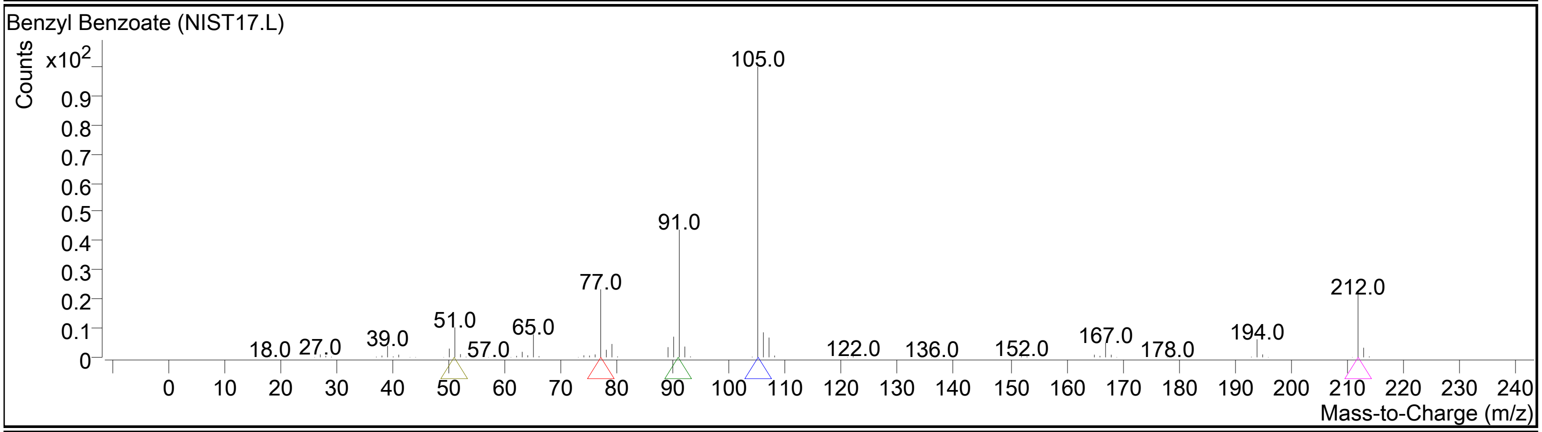

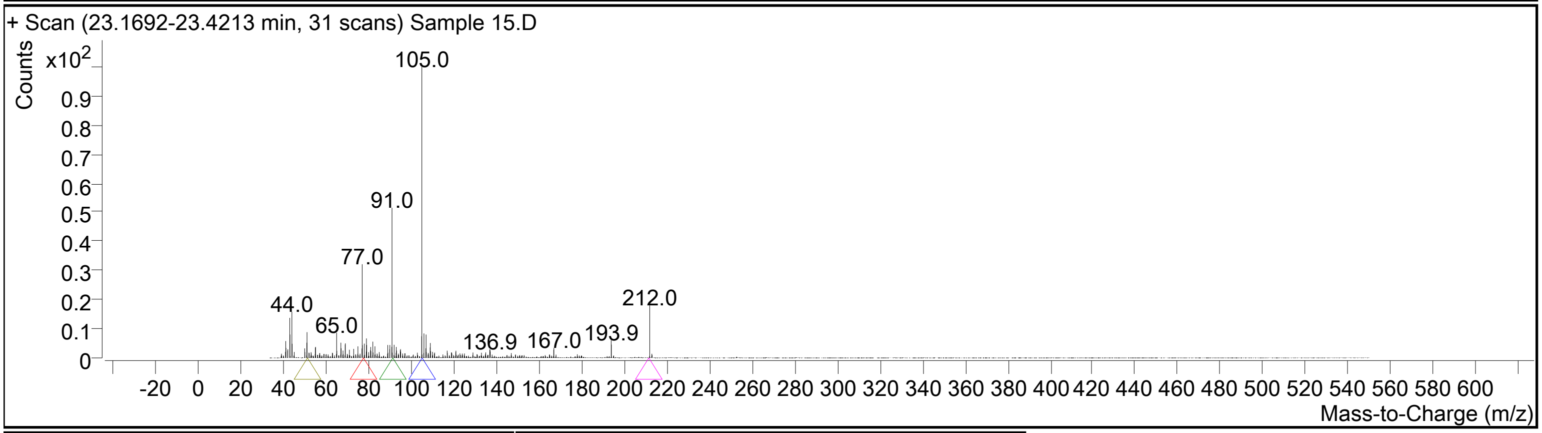

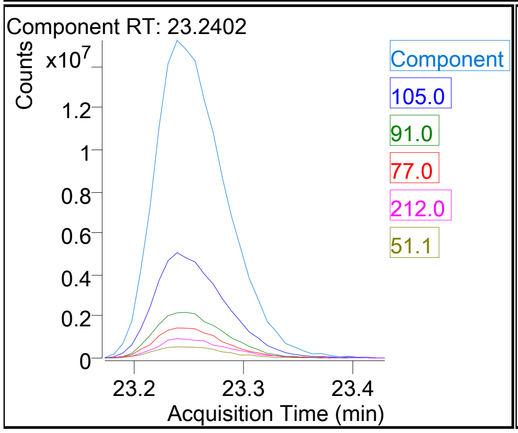

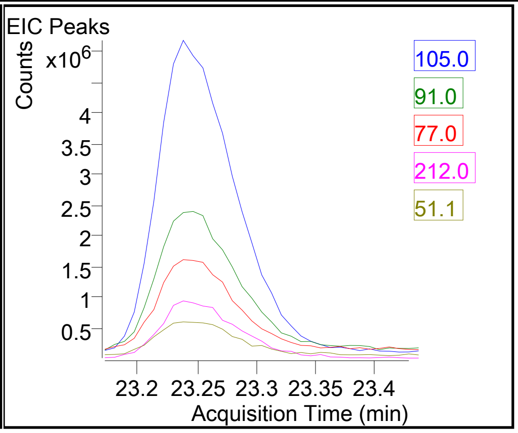

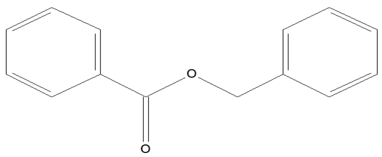

Generated at 12:24 PM on 2/25/2021

Library Search Results - NonTarget Hits with Details

Agilent Technologies

Component RT	Compound Name	Component Area	Match Factor	CAS#	Formula	Estimated Conc.
23.9727	6-Hydroxy-4,4,7a-trimethyl-5,6,7,7a-tetrahydrobenzofuran-2(4H)-one	93889305.7	87.7	73410-02-3	C11H16O3	

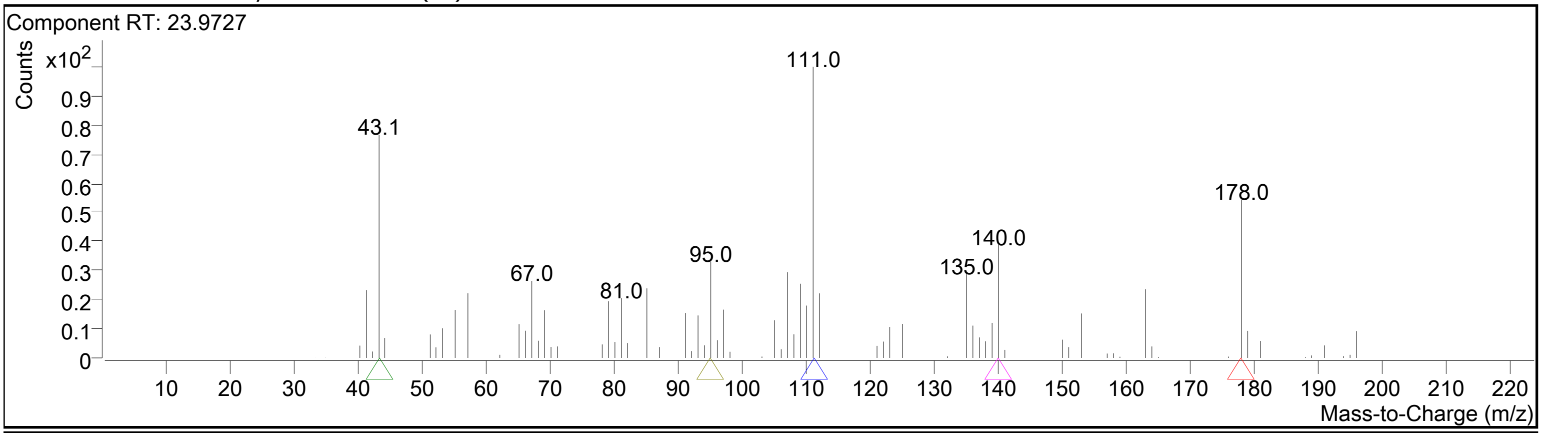

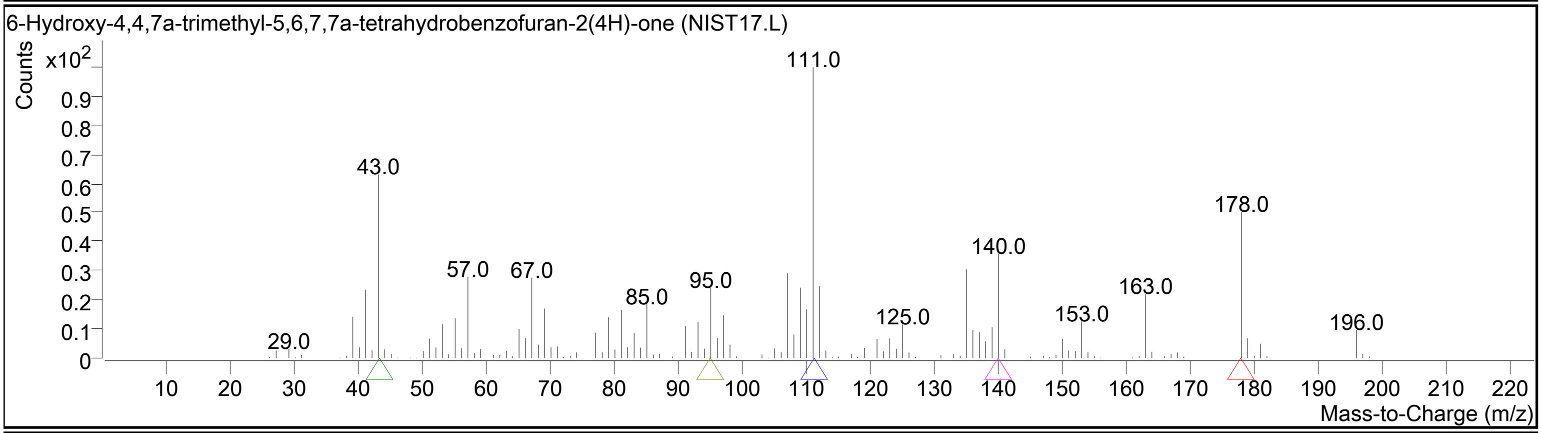

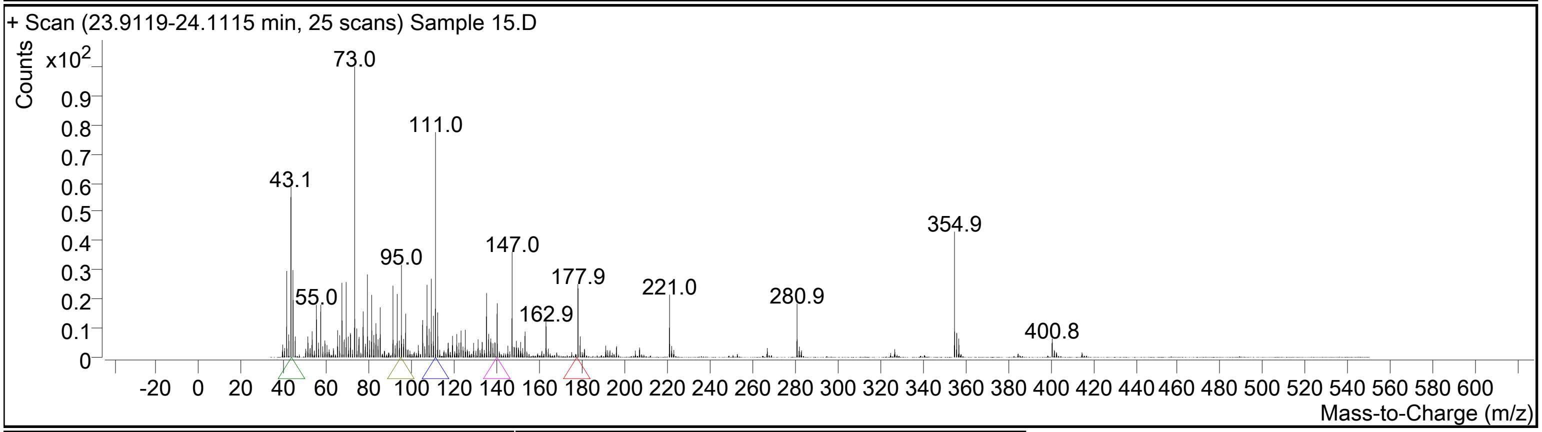

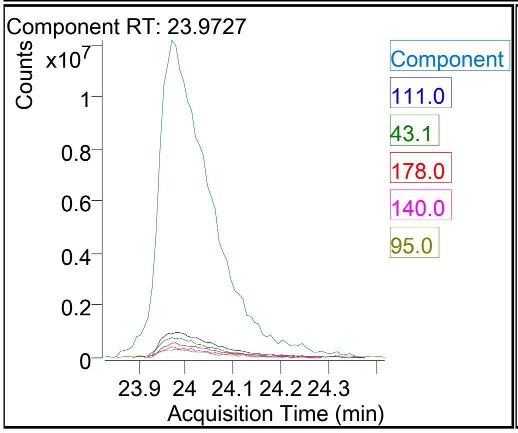

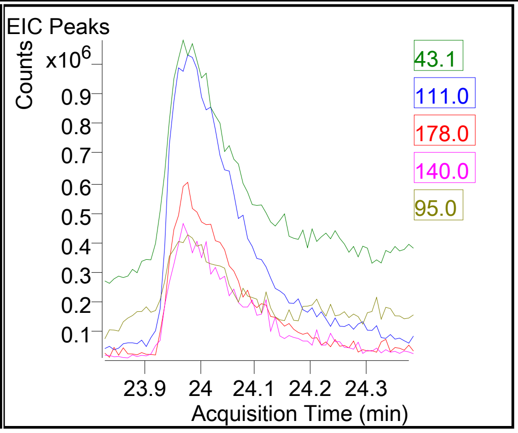

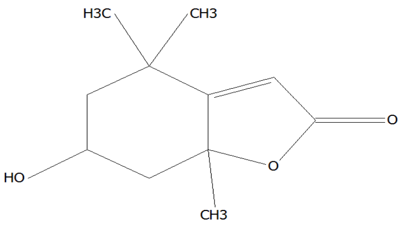

Generated at 12:24 PM on 2/25/2021

Component RT	Compound Name	Component Area	Match Factor	CAS#	Formula	Estimated Conc.
24.1506	2-Ethylhexanal ethylene glycol acetal	52729202.0	74.7	1000431-01-2	C10H20O2	

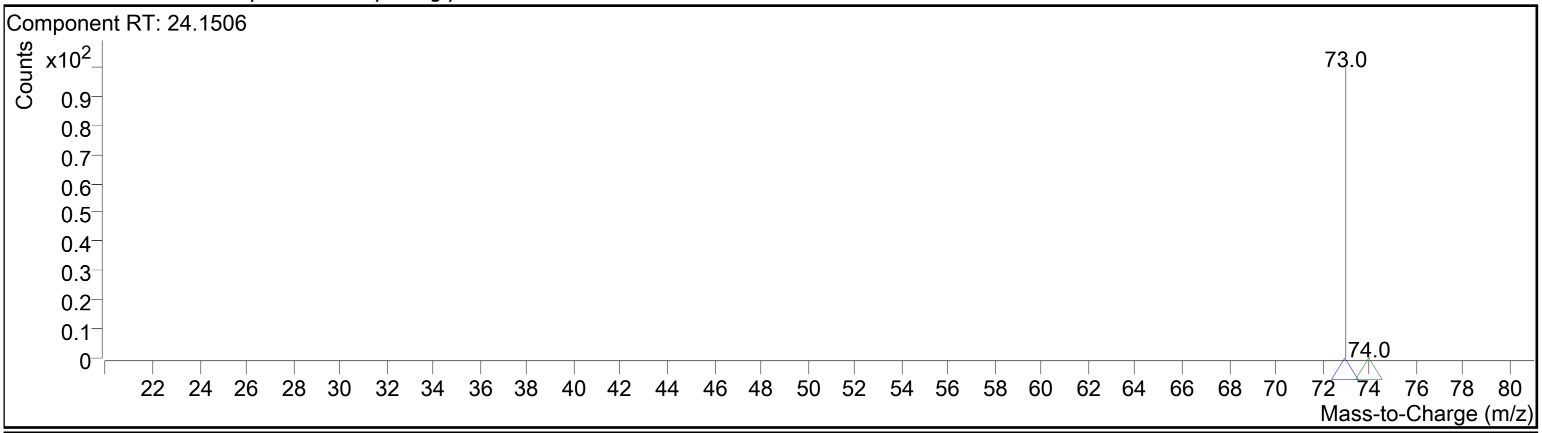

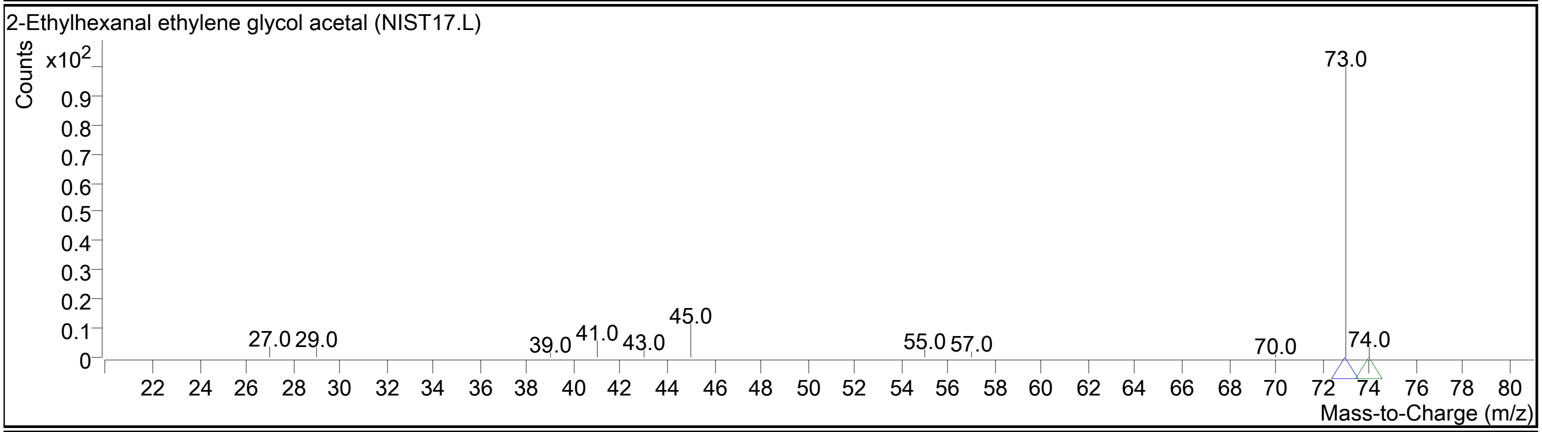

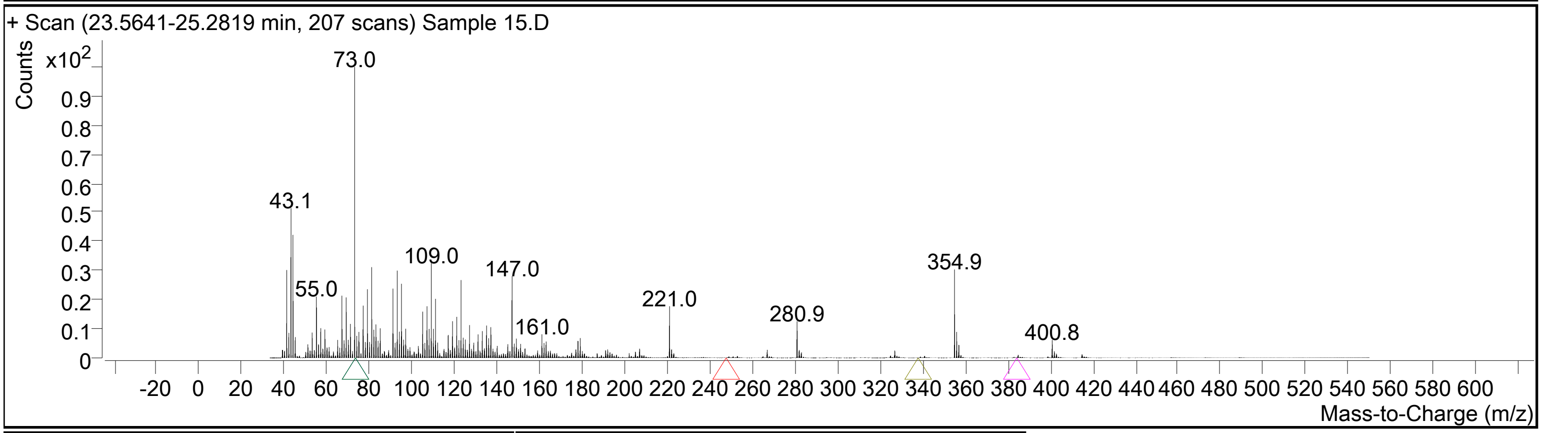

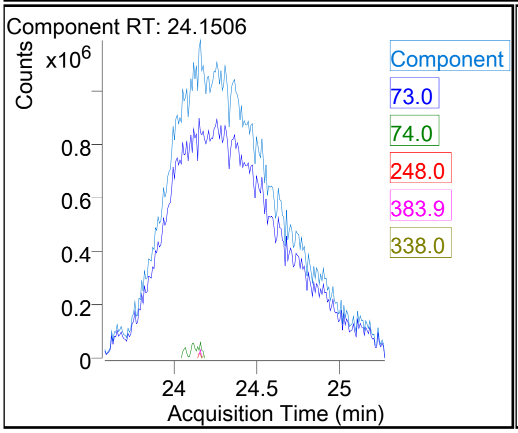

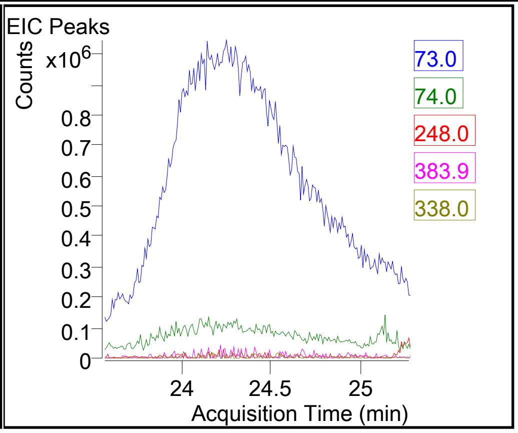

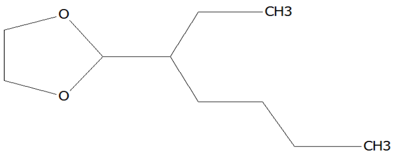

Component RT	Compound Name	Component Area	Match Factor	CAS#	Formula	Estimated Conc.
24.9110	Caryophyllene oxide	94785407.6	77.3	1139-30-6	C15H24O	

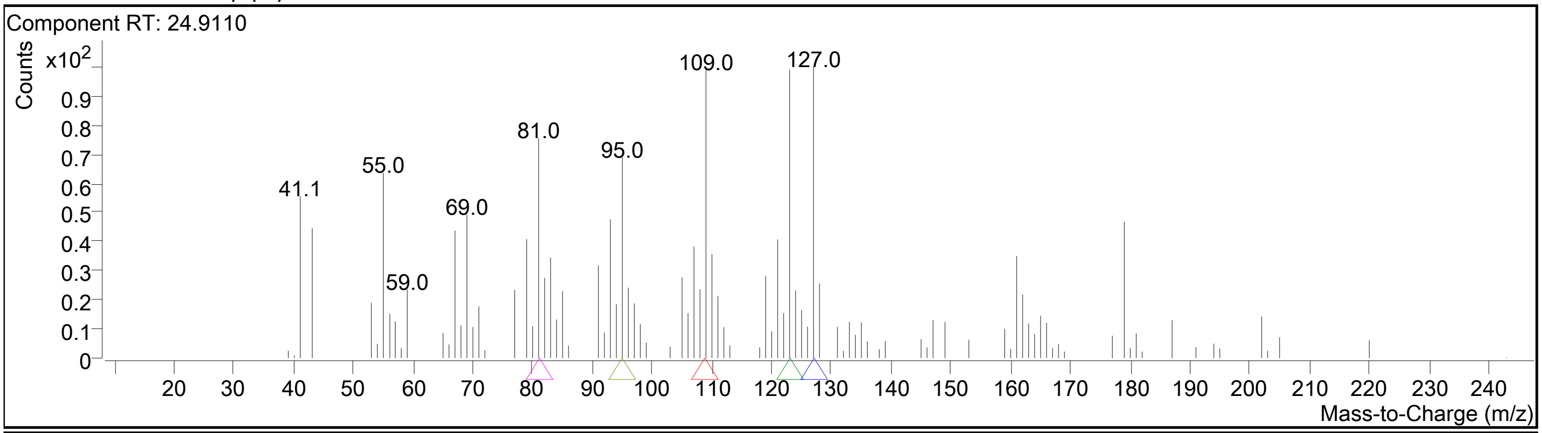

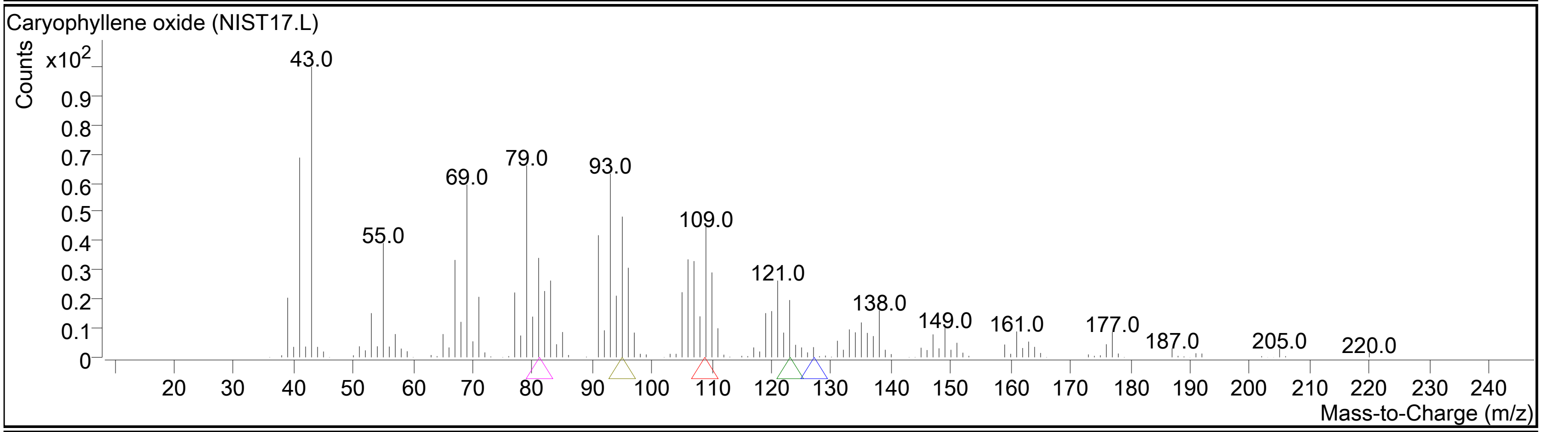

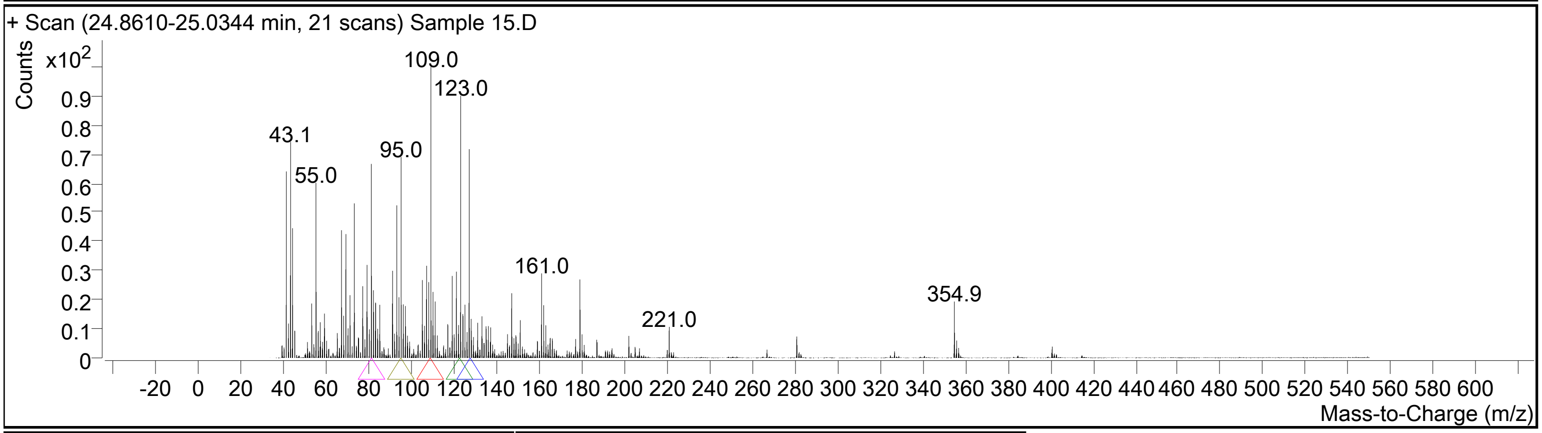

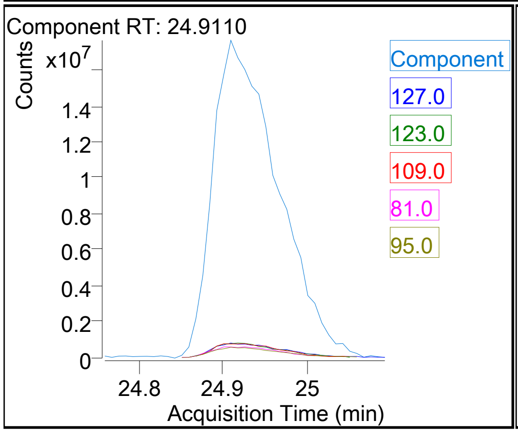

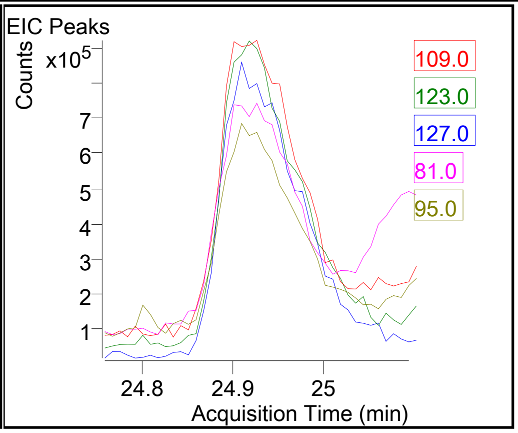

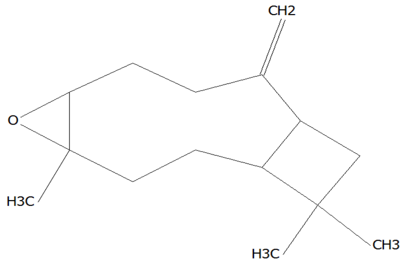

Component RT	Compound Name	Component Area	Match Factor	CAS#	Formula	Estimated Conc.
25.1584	Caryophyllene oxide	120035671.3	82.1	1139-30-6	C15H24O	

Component RT: 25.1584

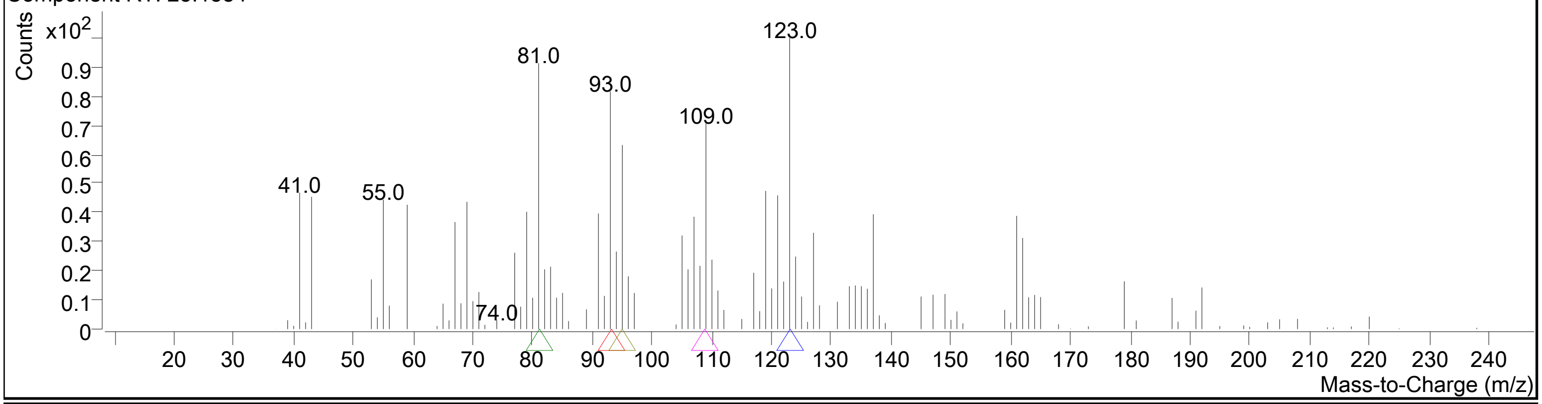

Caryophyllene oxide (NIST17.L)

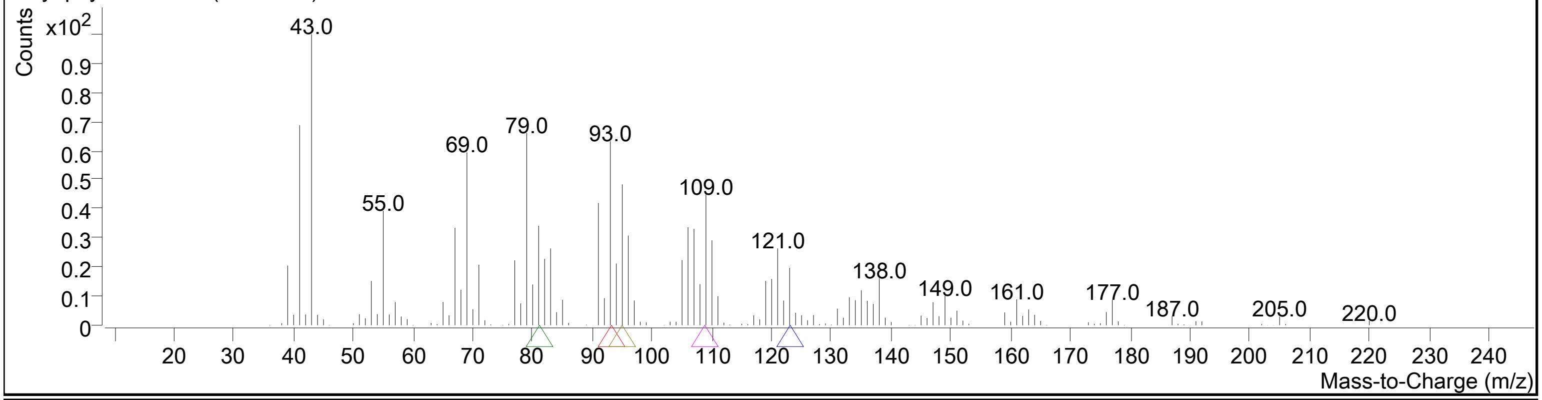

+ Scan (25.0790-25.1841 min, 13 scans) Sample 15.D

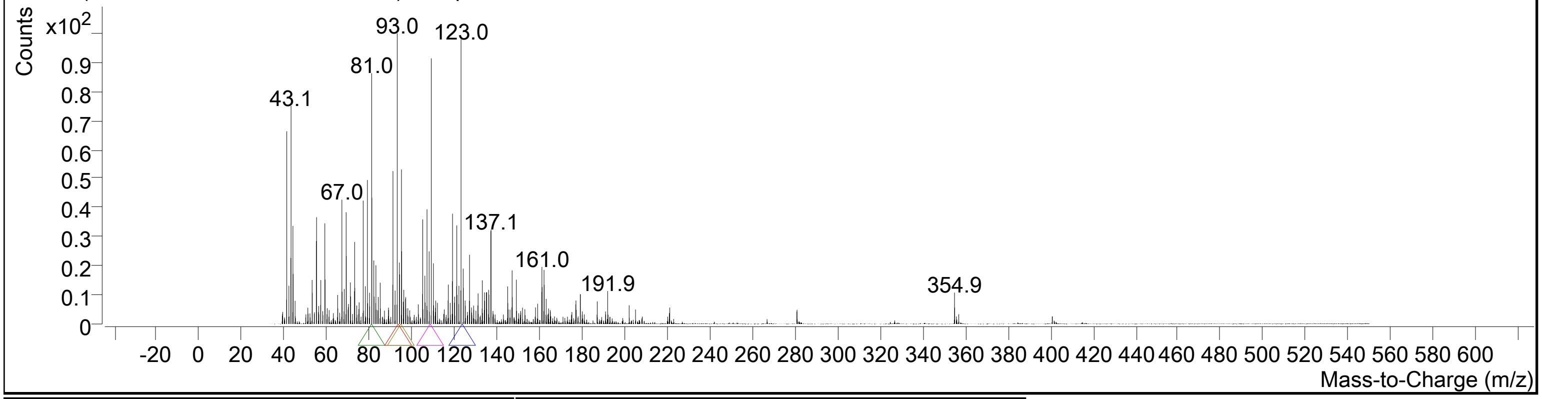

Component RT: 25.1584

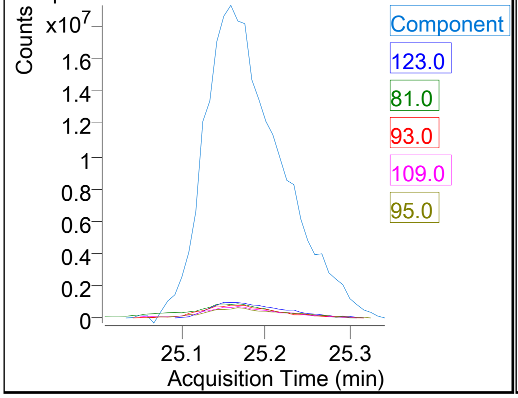

EIC Peaks

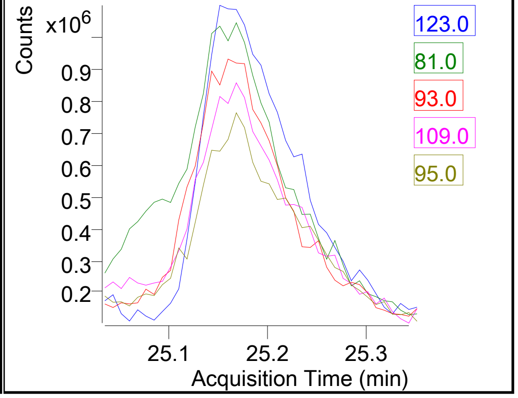

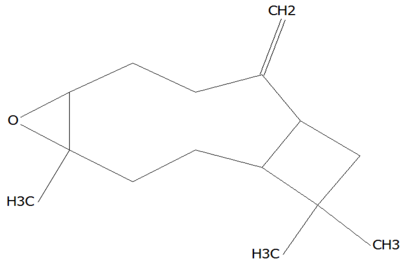

Component RT	Compound Name	Component Area	Match Factor	CAS#	Formula	Estimated Conc.
25.7494	Neophytadiene	19882967.8	75.4	504-96-1	$C_{20}H_{38}$	

Component RT: 25.7494

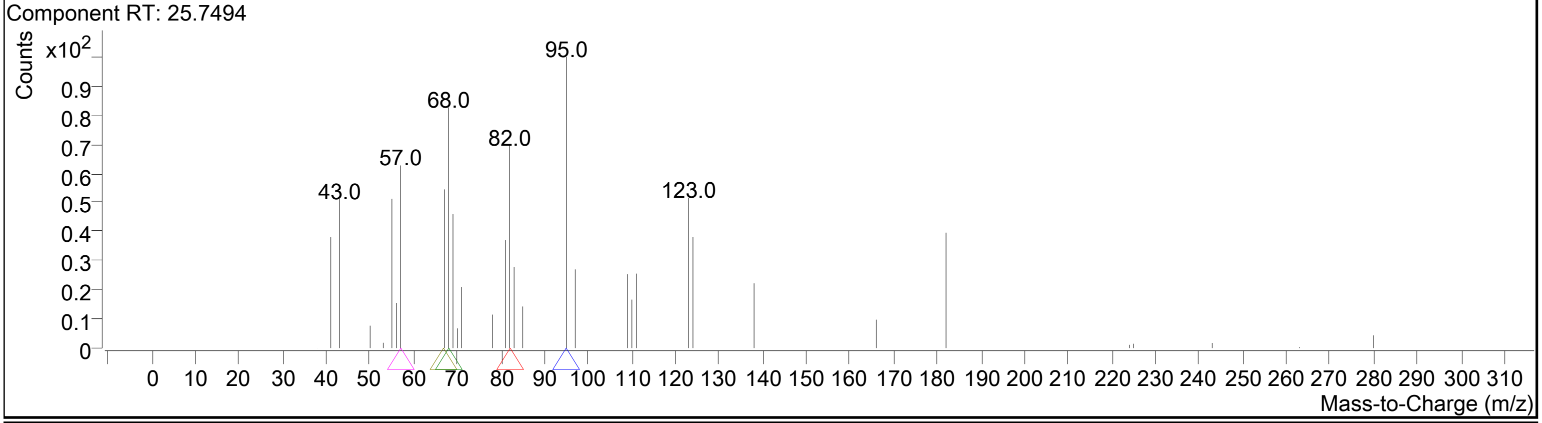

Neophytadiene (NIST17.L)

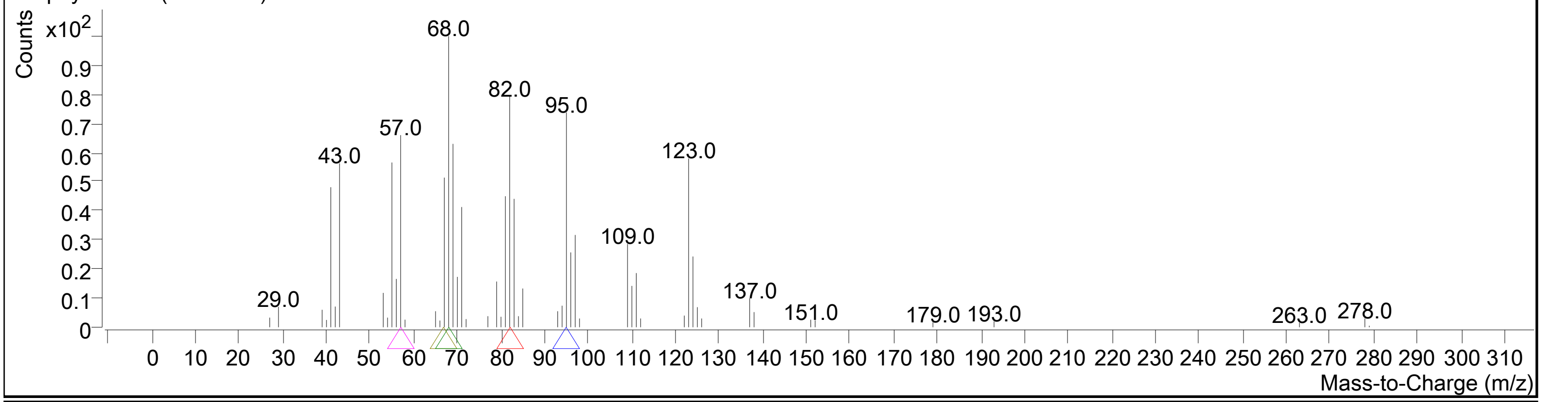

+ Scan (25.7038-25.8160 min, 14 scans) Sample 15.D

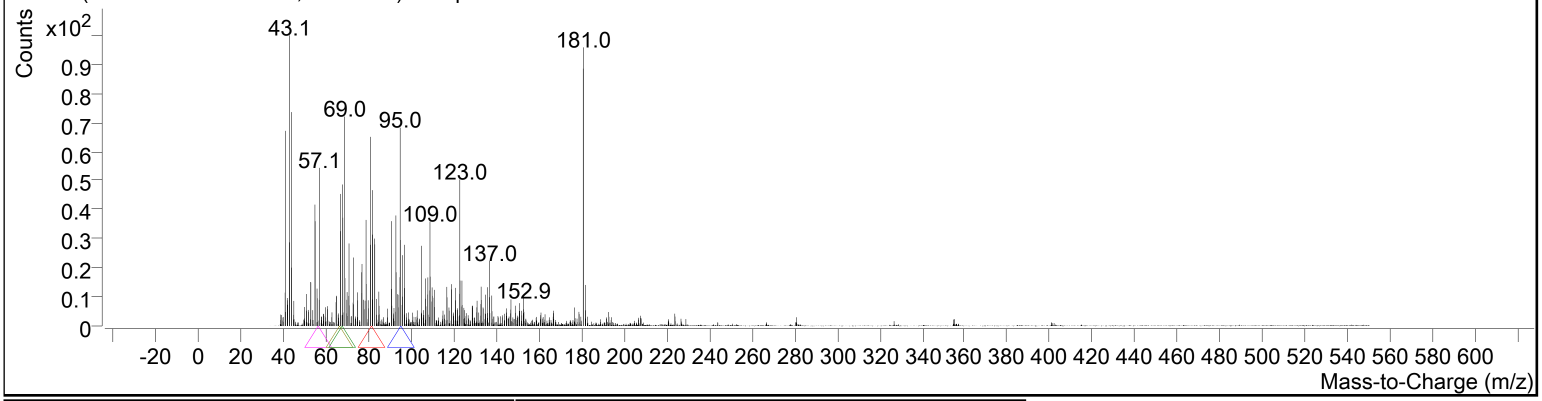

Component RT: 25.7494

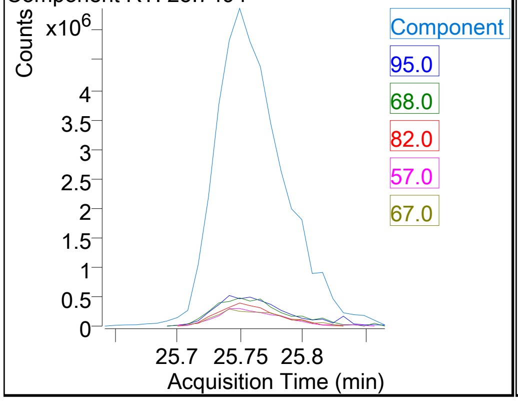

EIC Peaks

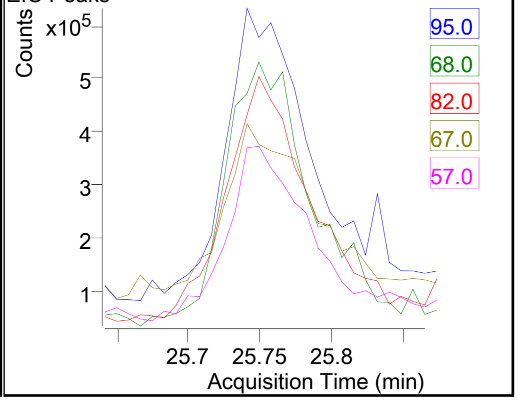

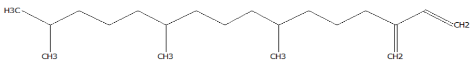

 Generated at 12:24 PM on 2/25/2021

Component RT	Compound Name	Component Area	Match Factor	CAS#	Formula	Estimated Conc.
25.9197	2-Pentadecanone, 6,10,14-trimethyl-	25388415.5	82.7	502-69-2	C18H36O	

Component RT: 25.9197

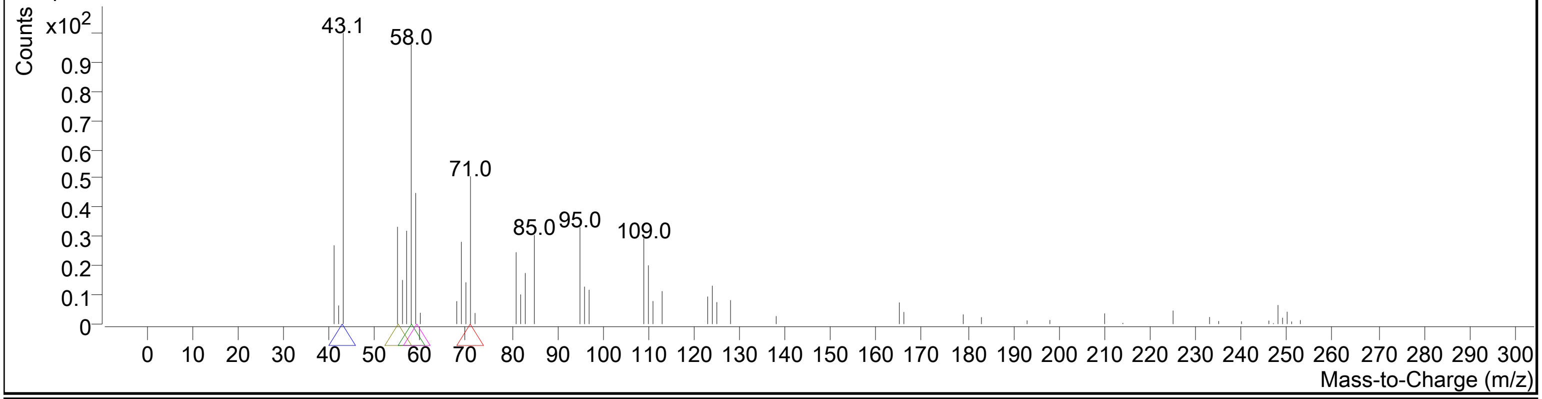

2-Pentadecanone, 6,10,14-trimethyl- (NIST17.L)

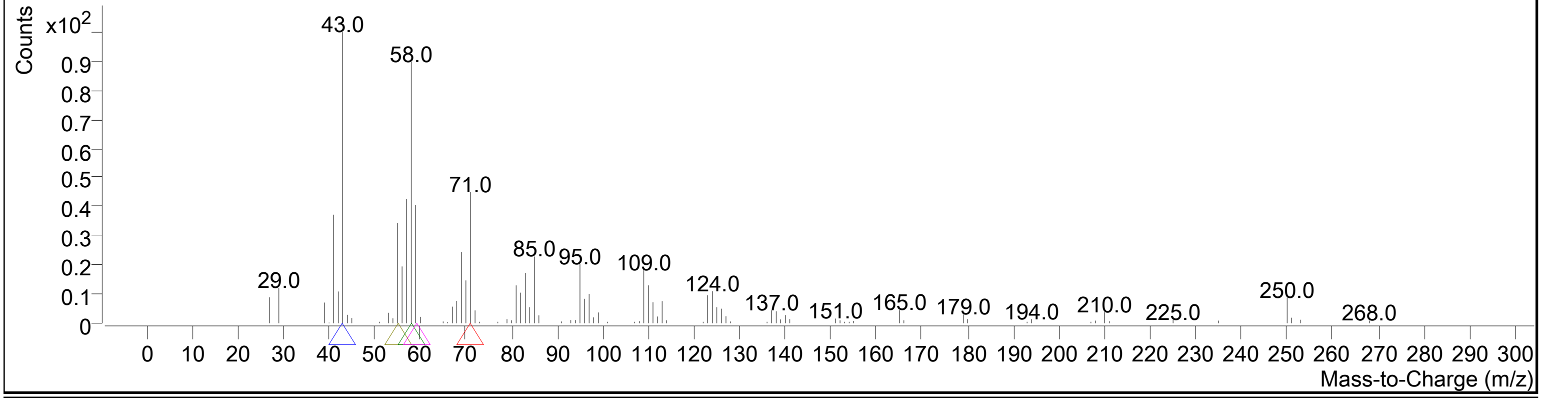

+ Scan (25.8596-26.0550 min, 24 scans) Sample 15.D

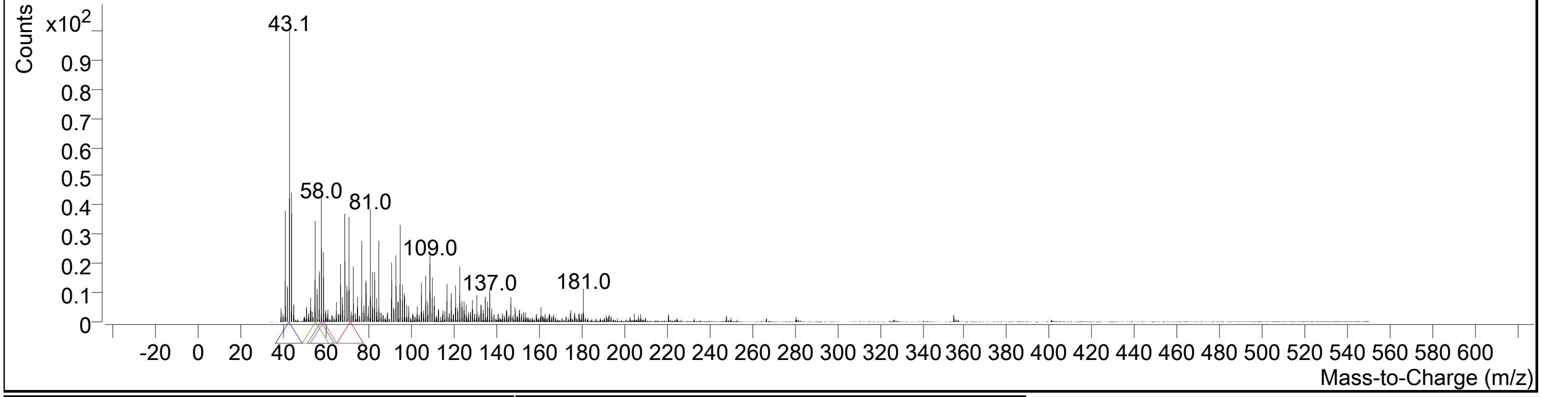

Component RT: 25.9197

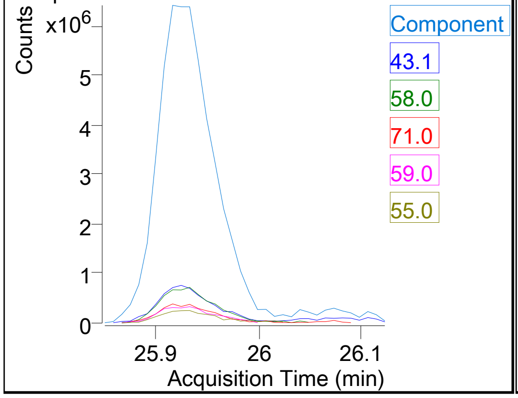

EIC Peaks

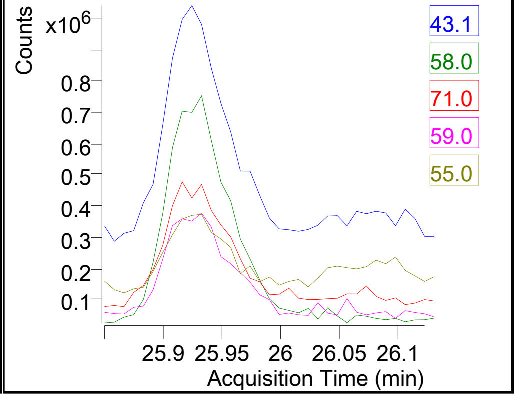

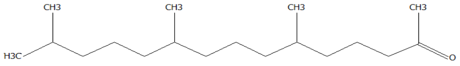

Component RT	Compound Name	Component Area	Match Factor	CAS#	Formula	Estimated Conc.
25.9254	2-Heptanone, 6-methyl-	18963734.4	65.8	928-68-7	C8H16O	

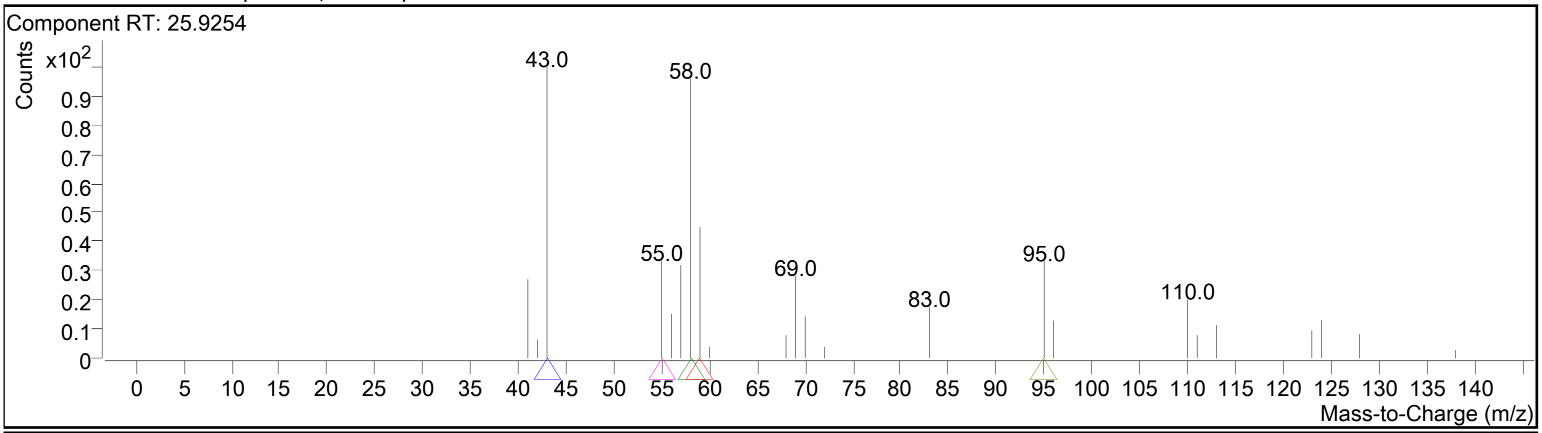

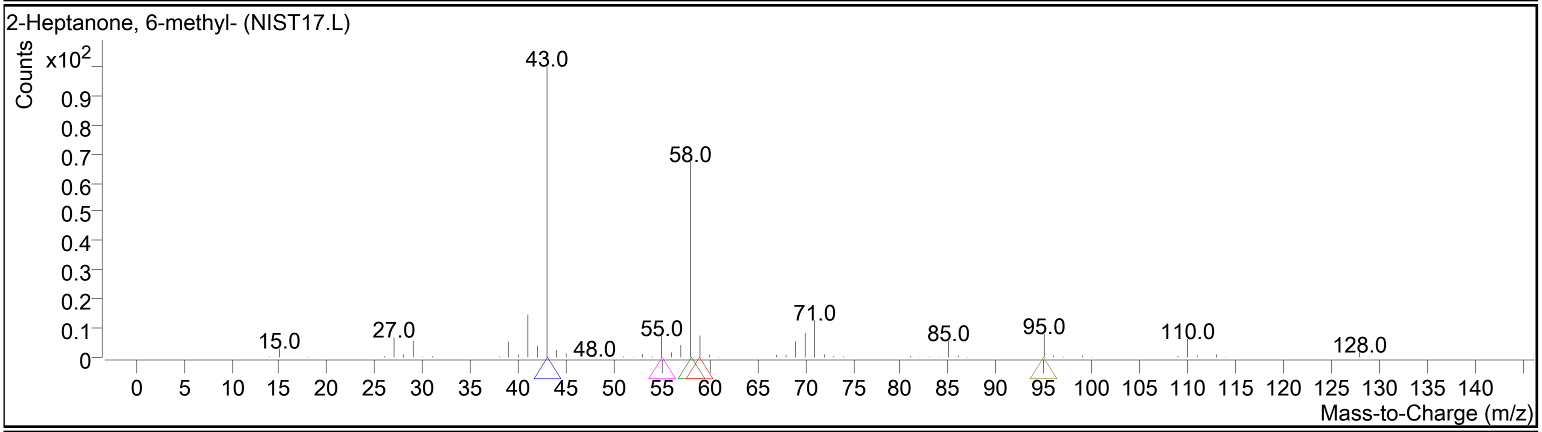

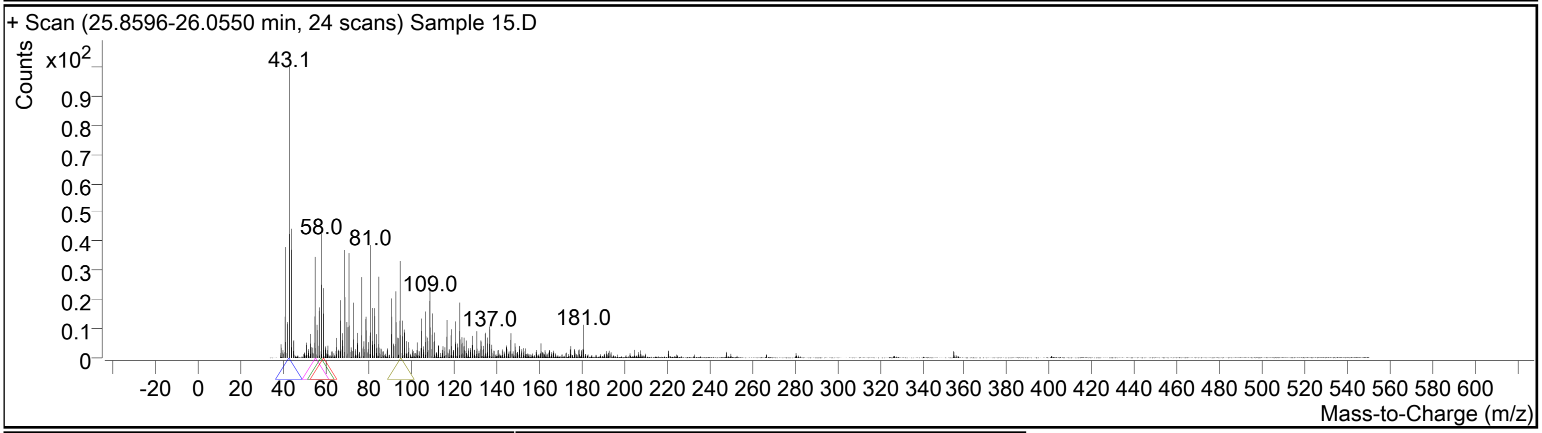

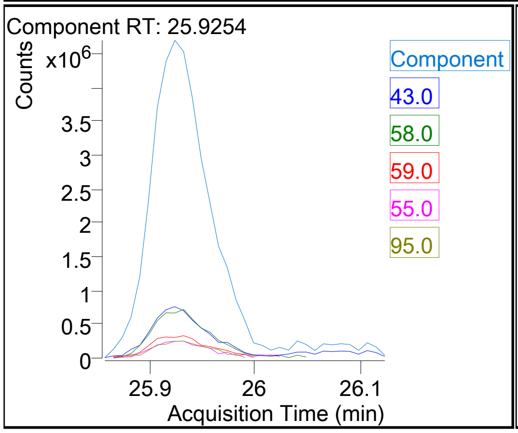

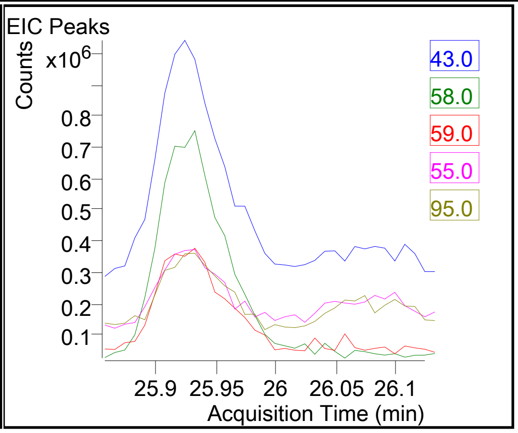

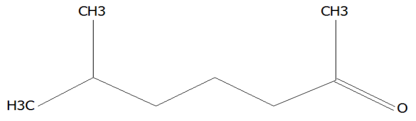

Agilent Technologies

Component RT	Compound Name	Component Area	Match Factor	CAS#	Formula	Estimated Conc.
26.3348	Benzoic acid, 2-hydroxy-, phenylmethyl ester	72961635.6	88.1	118-58-1	C14H12O3	

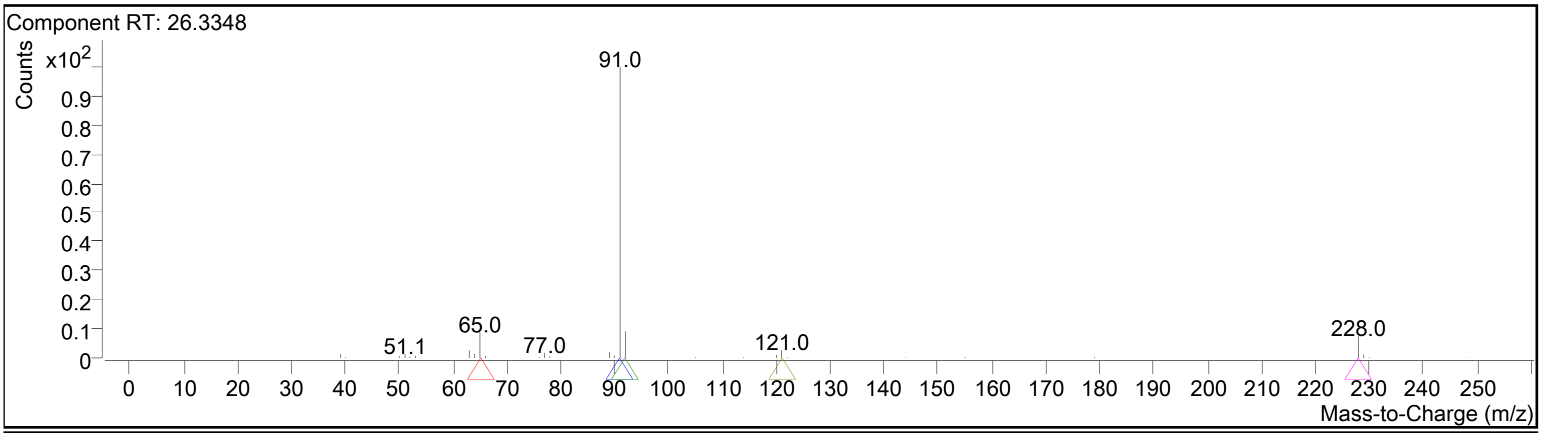

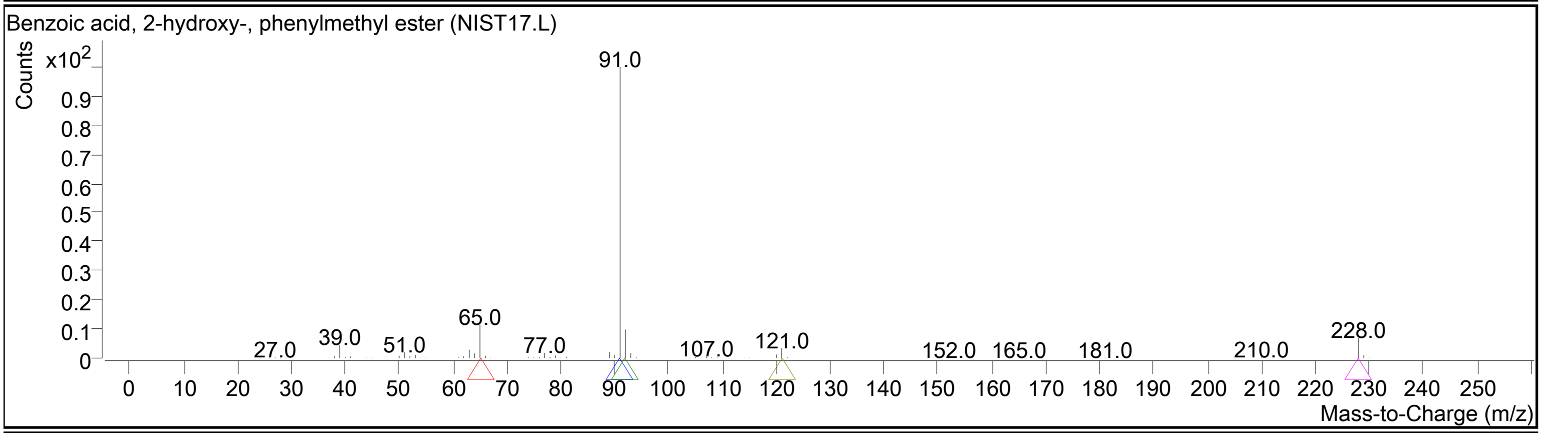

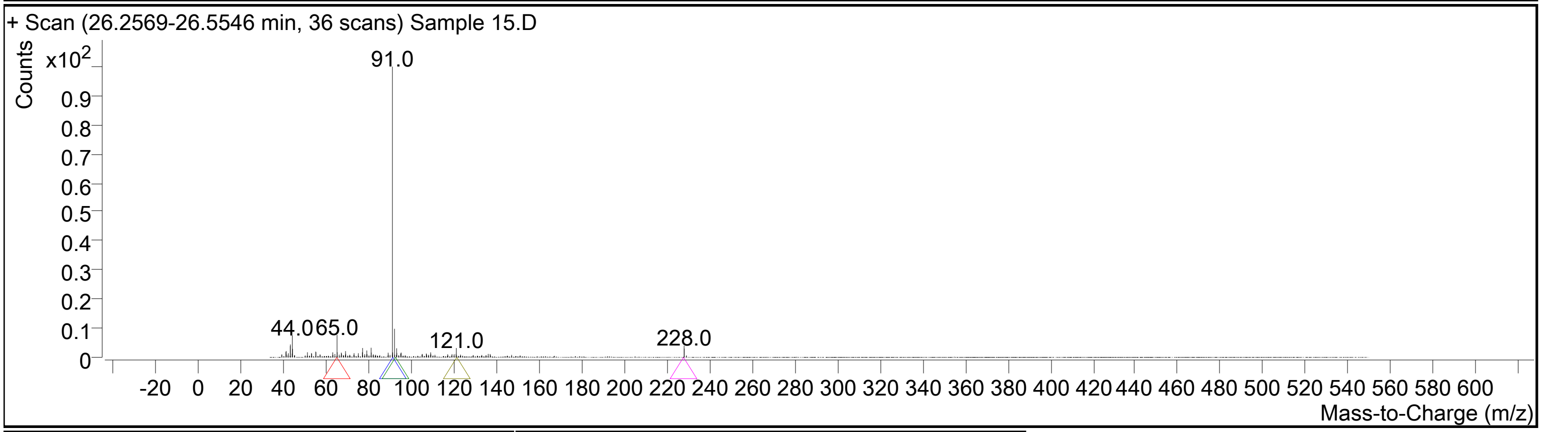

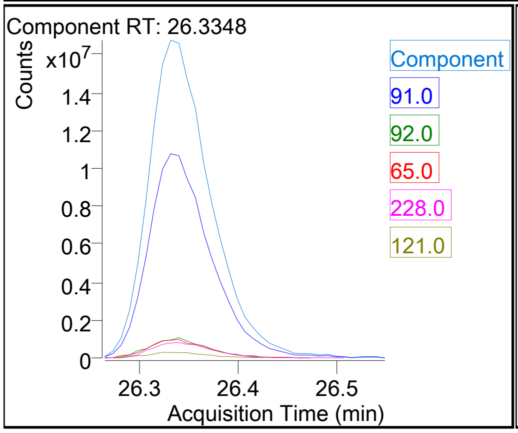

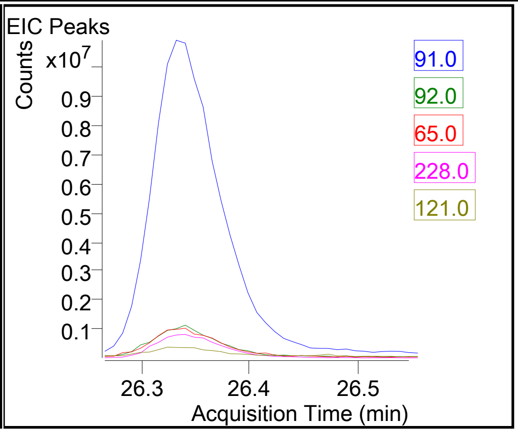

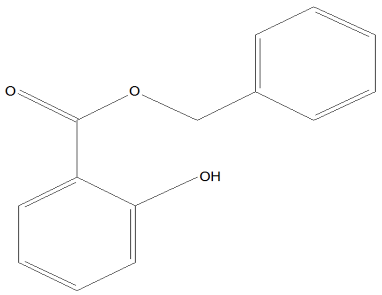

Component RT	Compound Name	Component Area	Match Factor	CAS#	Formula	Estimated Conc.
26.8667	(3S,3aS,6R,7R,9aS)-1,1,7-Trimethyldecahydro-3a,7-methanocyclopenta[8]annulene-3,6-diol	150284933.2	88.9	2649-64-1	C15H26O2	

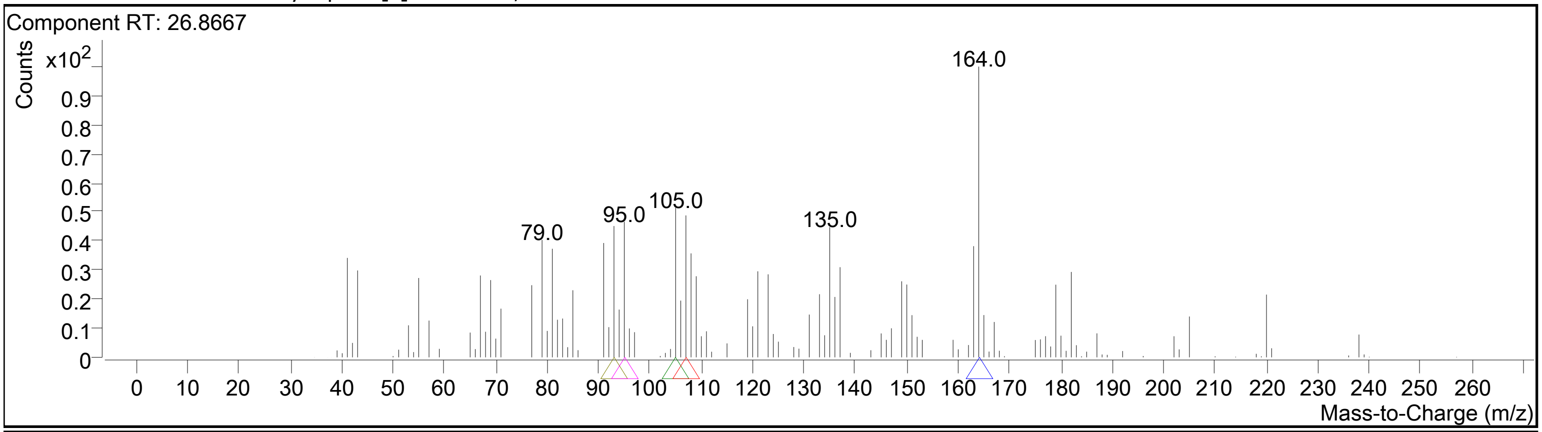

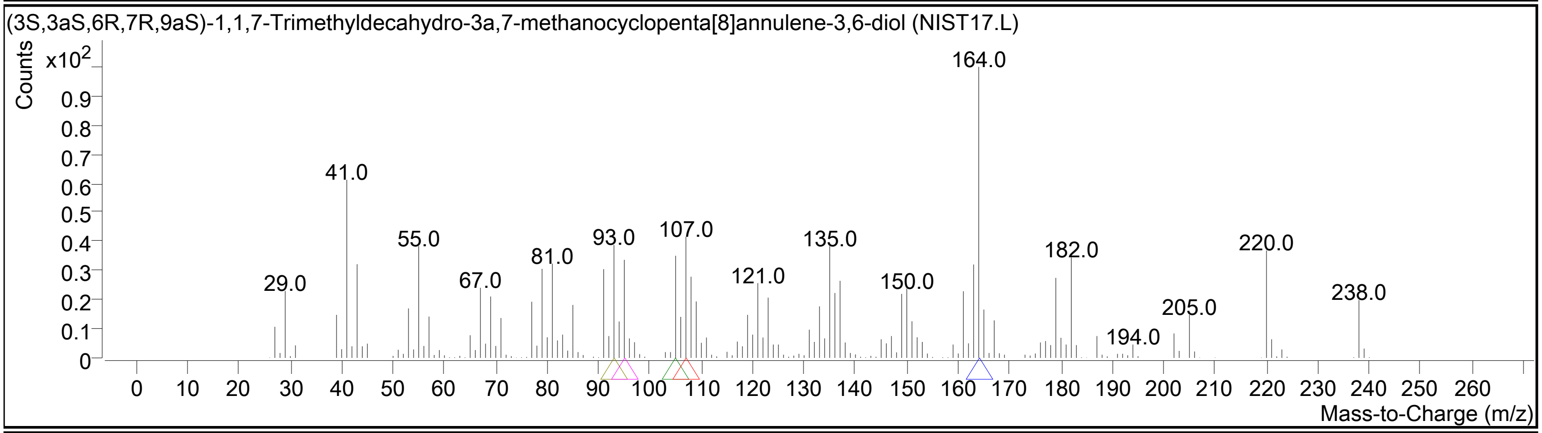

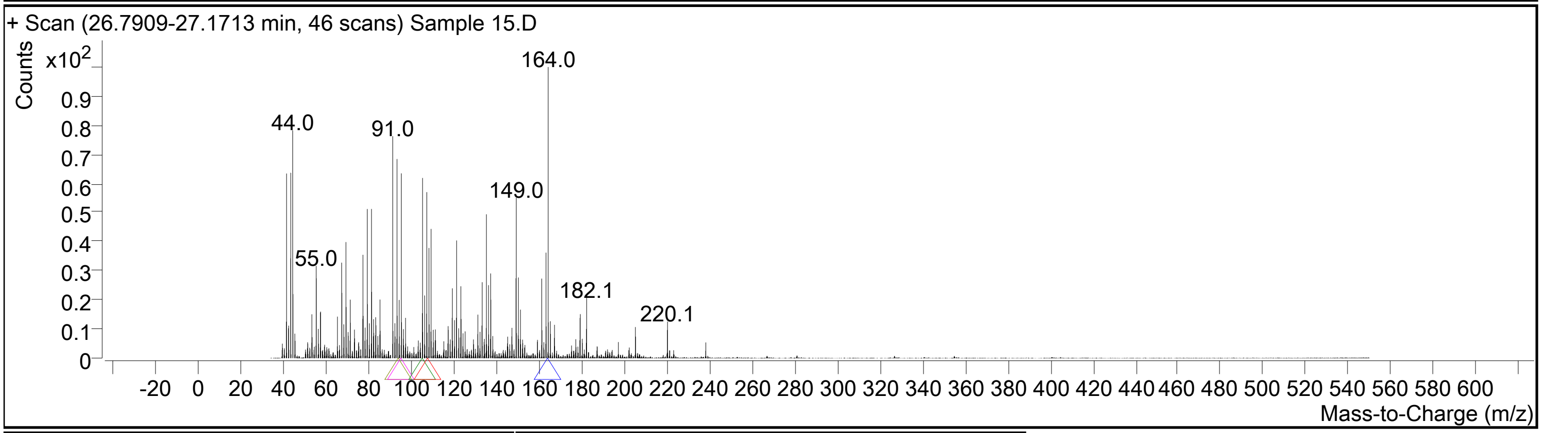

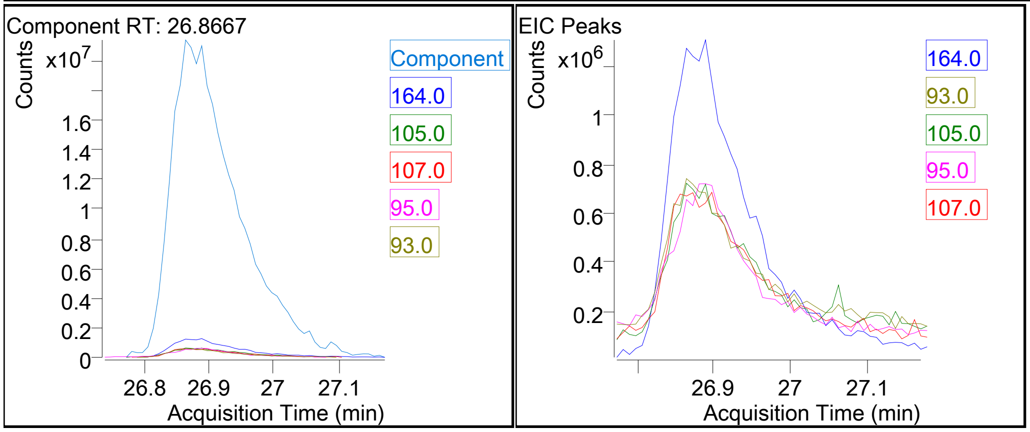

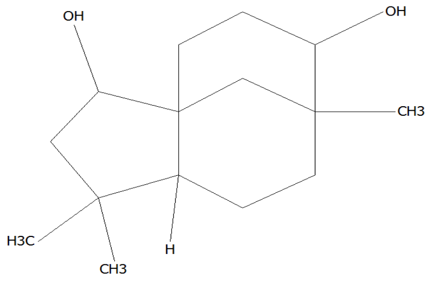

Component RT	Compound Name	Component Area	Match Factor	CAS#	Formula	Estimated Conc.
27.0619	Ethaneperoxoic acid, 1-cyano-1-[2-(2-phenyl-1,3-dioxolan-2-yl)ethyl]pentyl ester	5590160.0	77.8	58422-92-7	C19H25NO5	

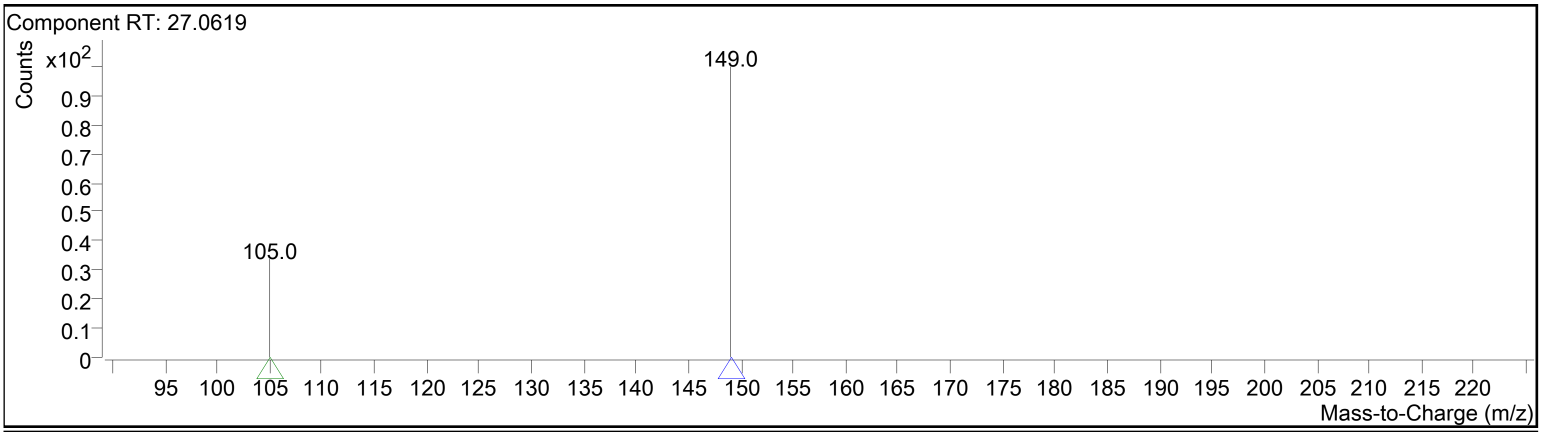

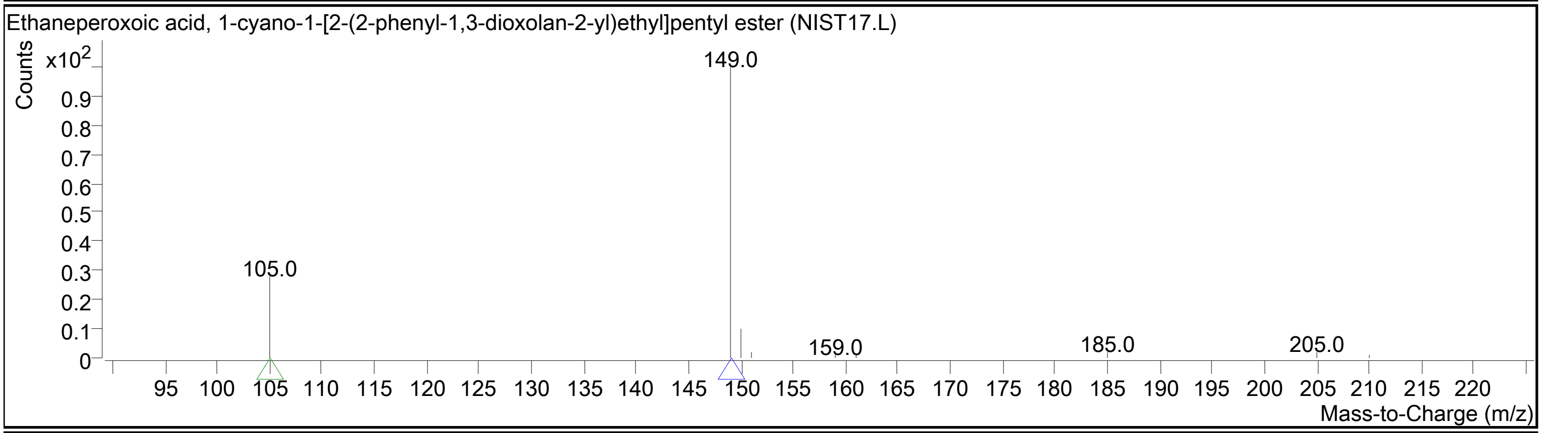

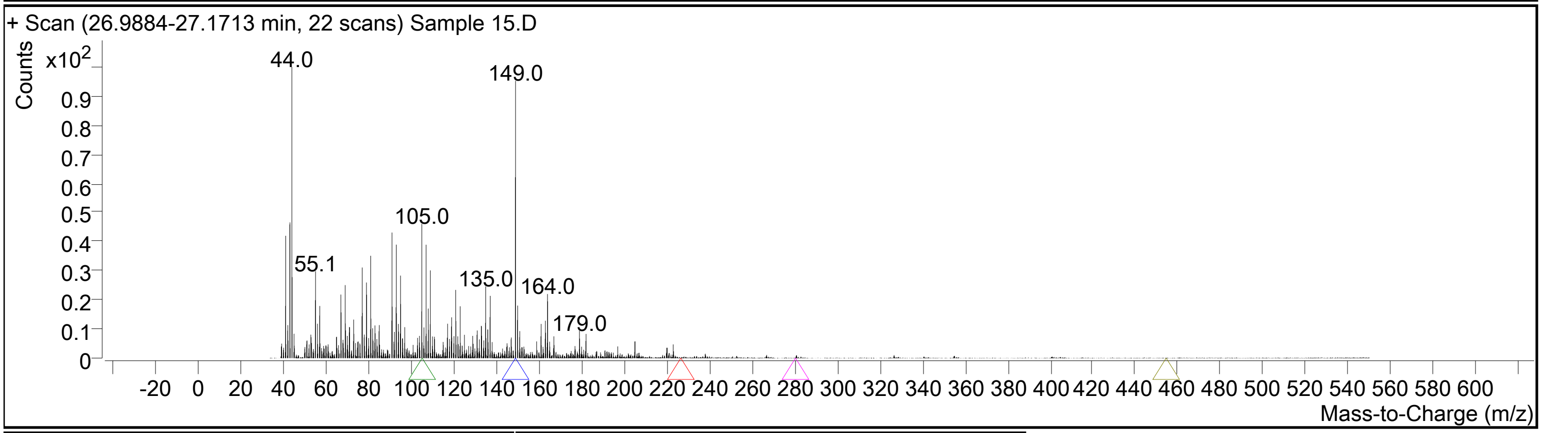

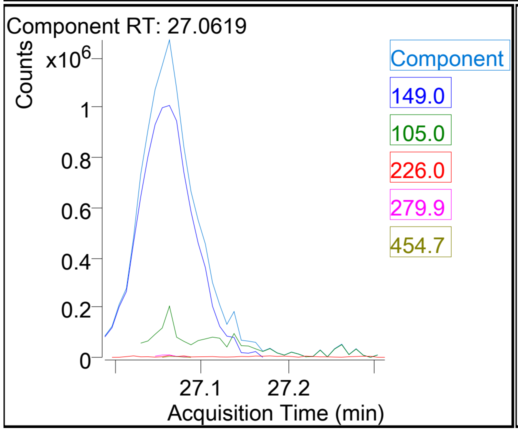

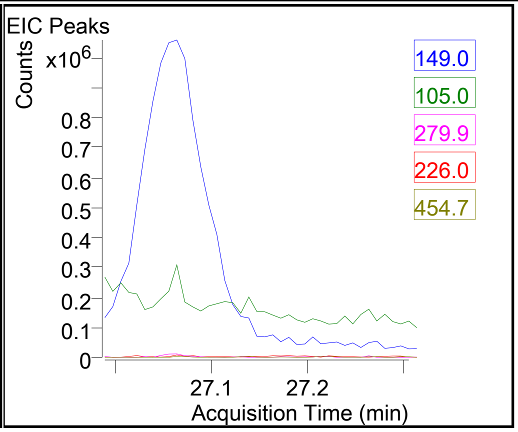

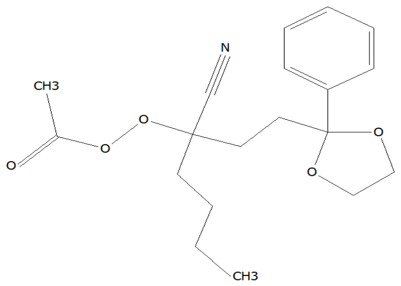

Component RT	Compound Name	Component Area	Match Factor	CAS#	Formula	Estimated Conc.
27.7857	Phenanthrene, 7-ethenyl-1,2,3,4,4a,4b,5,6,7,9,10,10a-dodecahydro-1,1,4a,7-tetramethyl-, [4aS-(4a.alpha.,4b.beta.,7.beta.,10a.beta.)]-	23458675.7	70.3	1686-56-2	C20H32	

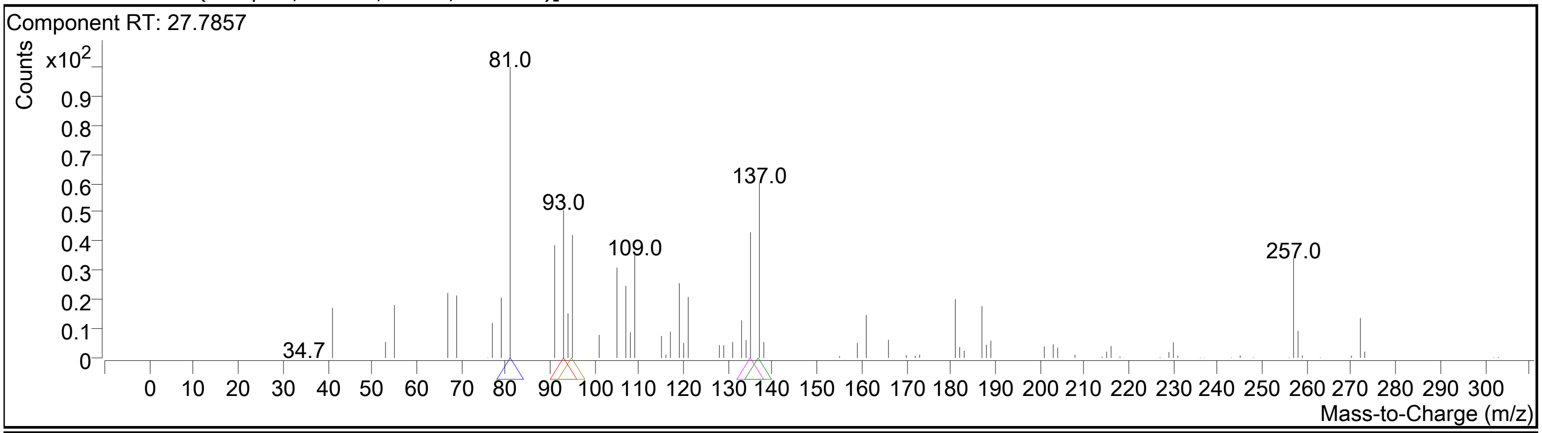

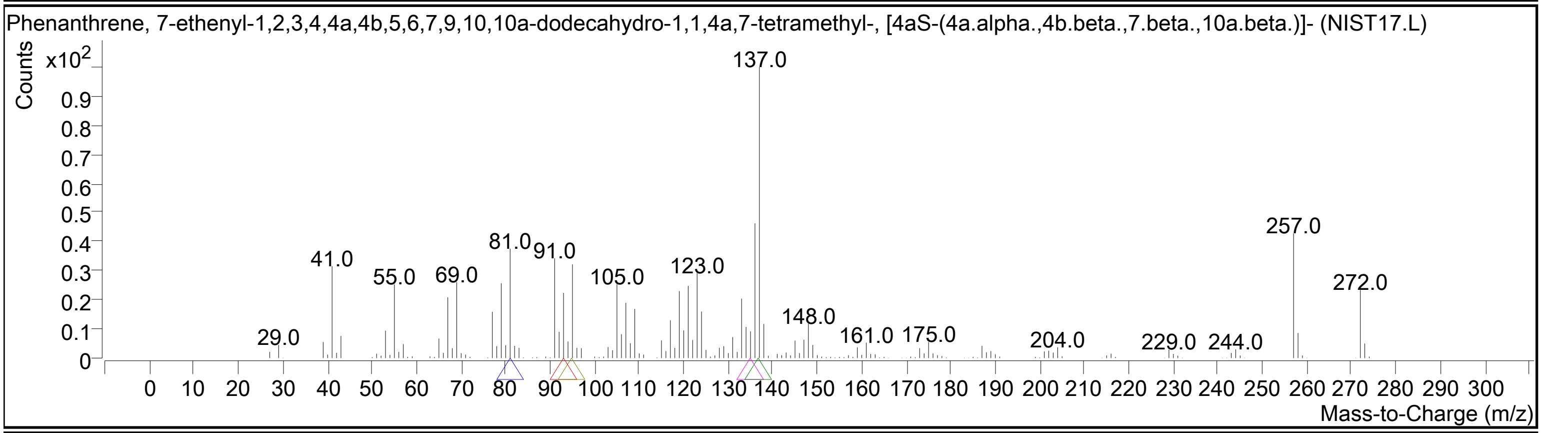

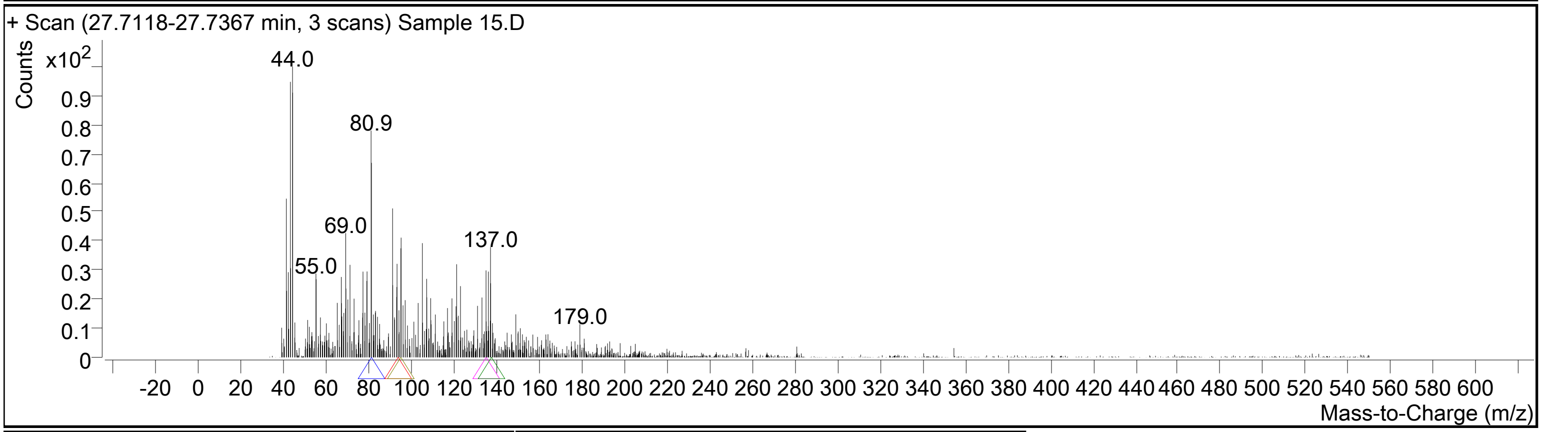

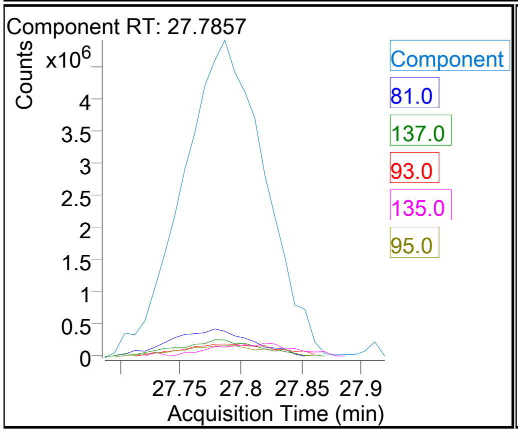

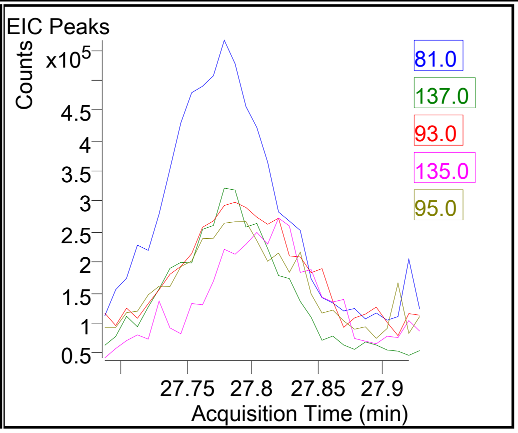

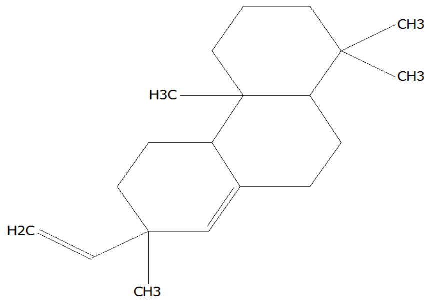

Agilent Technologies

Component RT	Compound Name	Component Area	Match Factor	CAS#	Formula	Estimated Conc.
28.9257	4-(6-Methoxy-3-methyl-2-benzofuranyl)-3-buten-2-one	46521483.9	80.0	10444-37-8	C14H14O3	

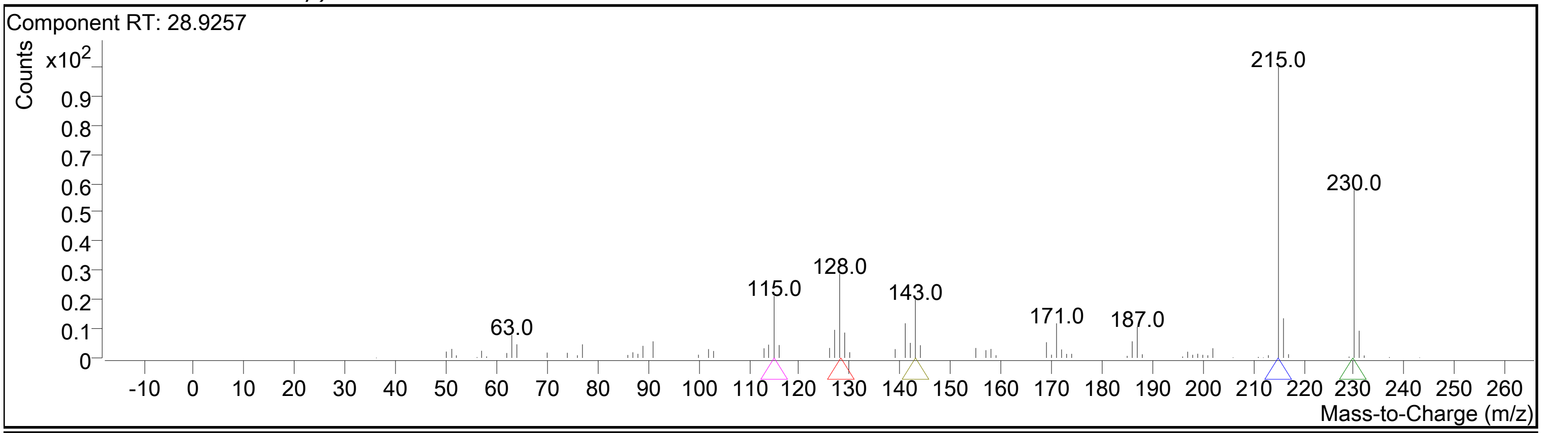

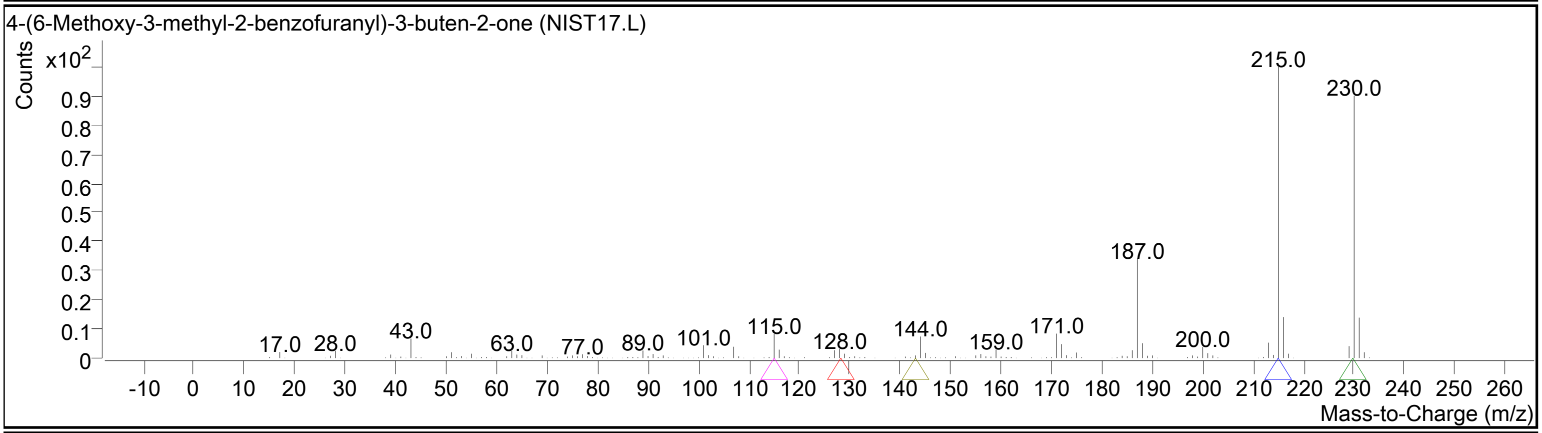

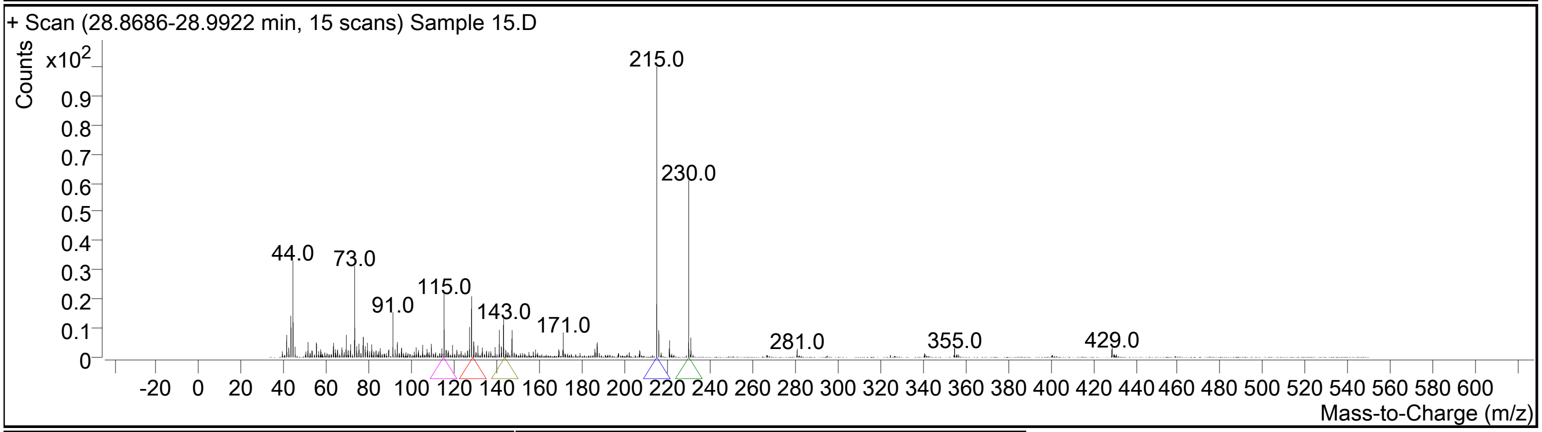

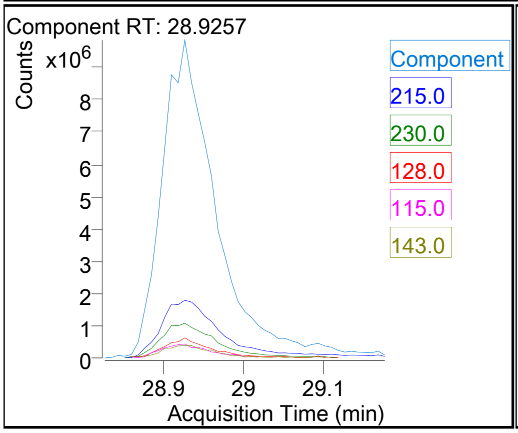

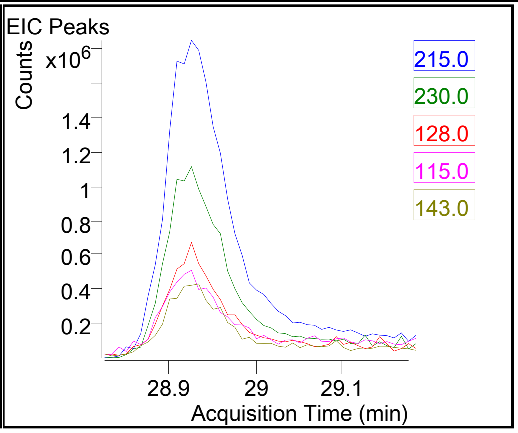

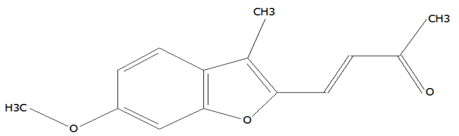

Component RT	Compound Name	Component Area	Match Factor	CAS#	Formula	Estimated Conc.
29.0920	Oxo-(6-phenyl-imidazo[1,2-a]pyridin-2-yl)-acetic acid, ethyl ester	3993381.6	70.4	1000318-40-7	C17H14N2O3	

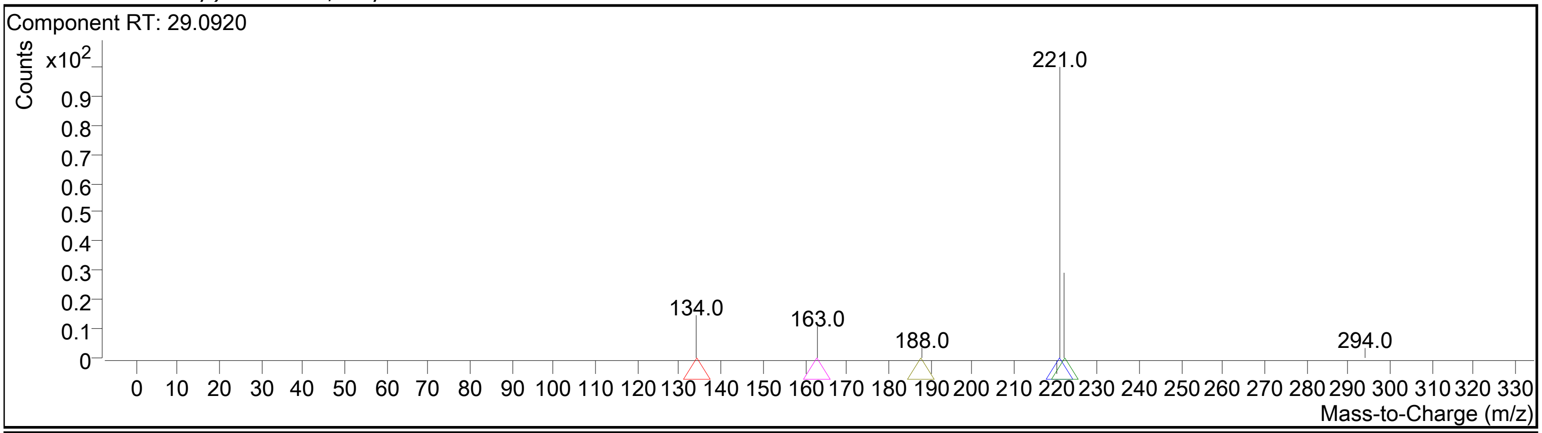

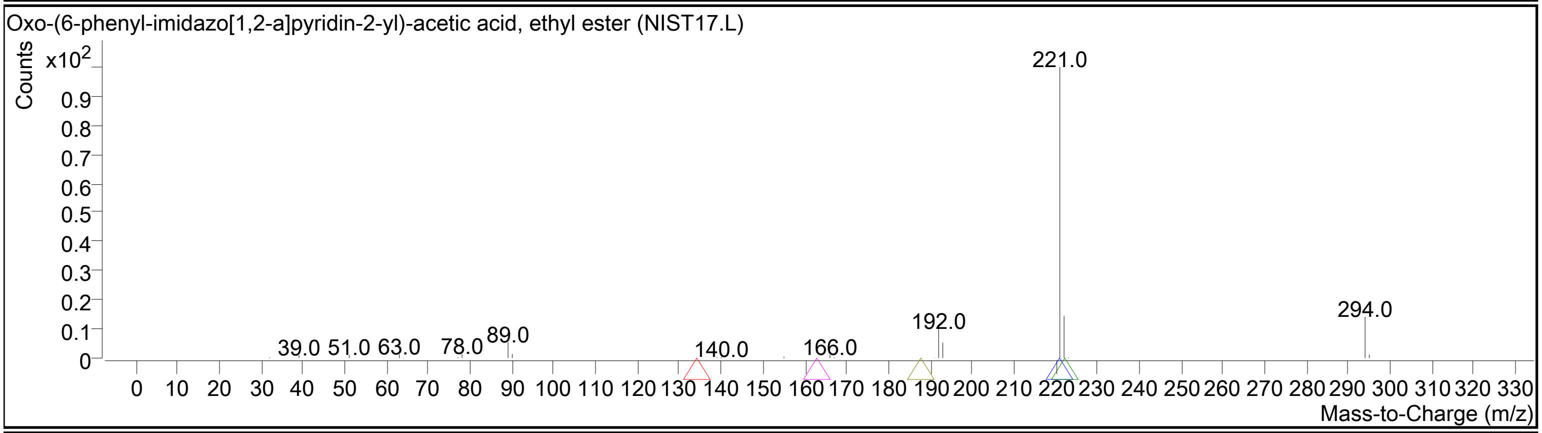

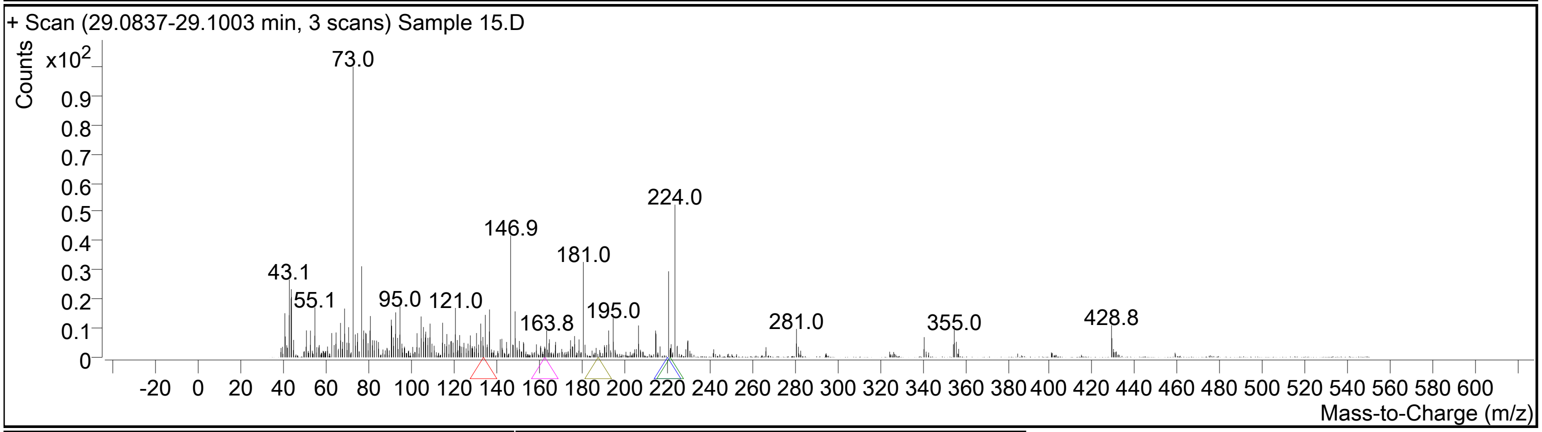

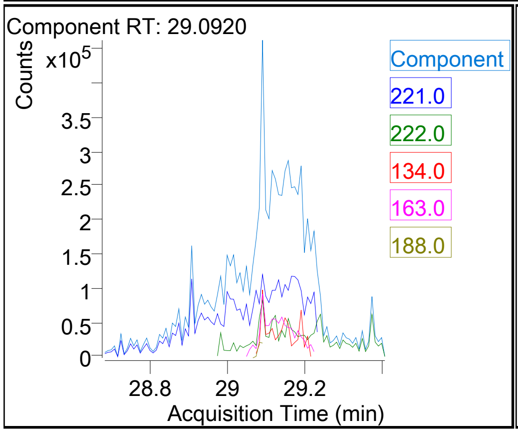

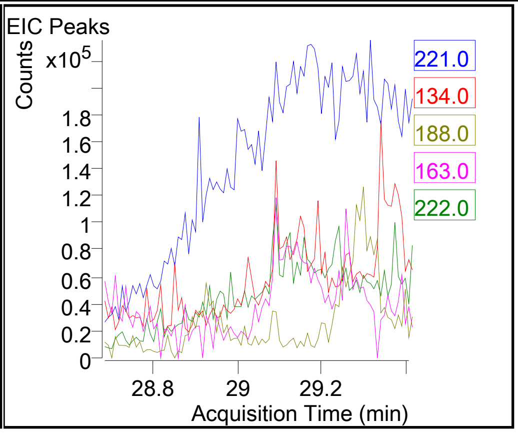

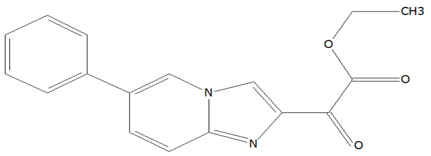

Component RT	Compound Name	Component Area	Match Factor	CAS#	Formula	Estimated Conc.
29.2854	6-(n-Butyl)oxy-4-methylcoumarin	58238616.2	70.1	1000395-87-3	C14H16O3	

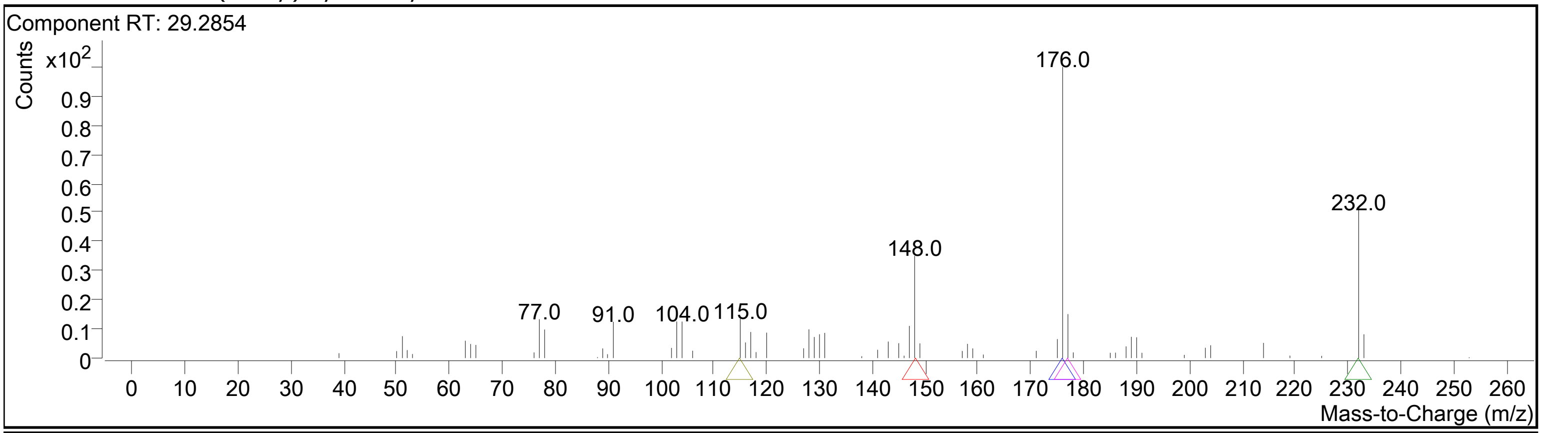

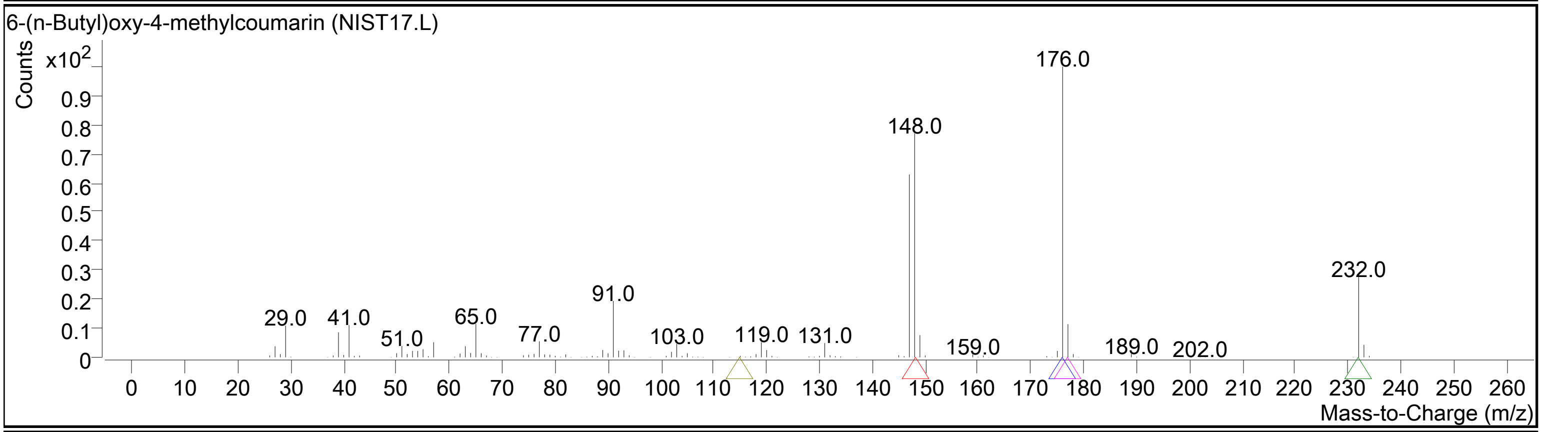

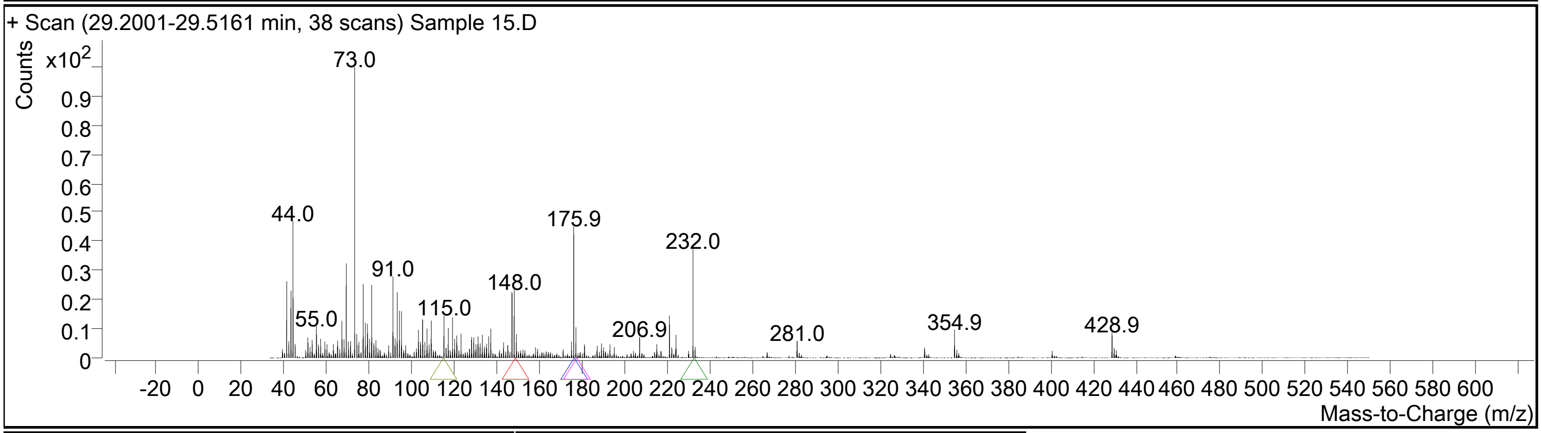

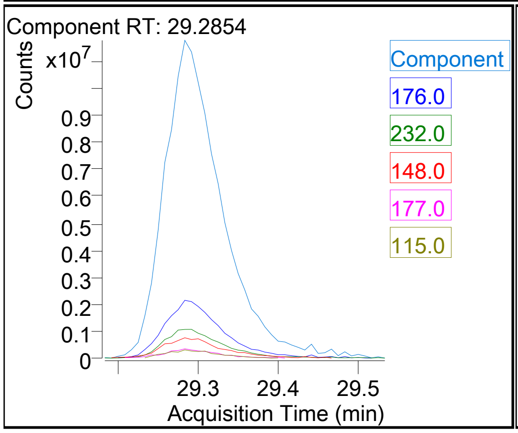

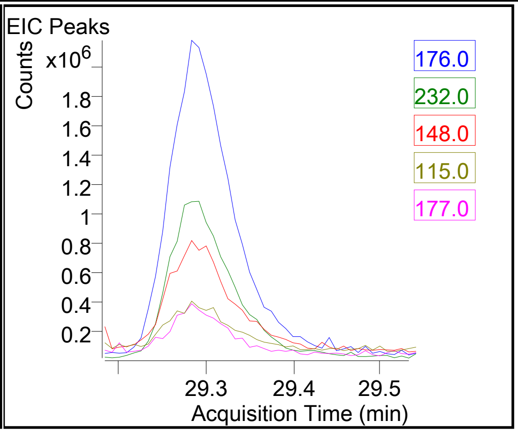

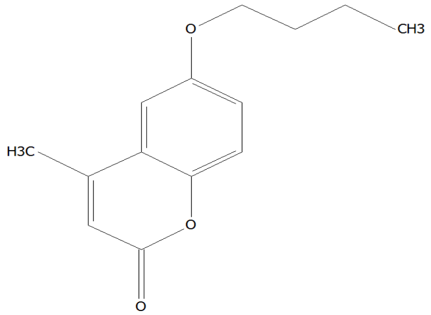

Component RT	Compound Name	Component Area	Match Factor	CAS#	Formula	Estimated Conc.
29.4912	4-Methyl-2,4-bis(p-hydroxyphenyl)pent-1-ene, 2TMS derivative	3763798.4	68.3	1000283-56-8	C24H36O2Si2	

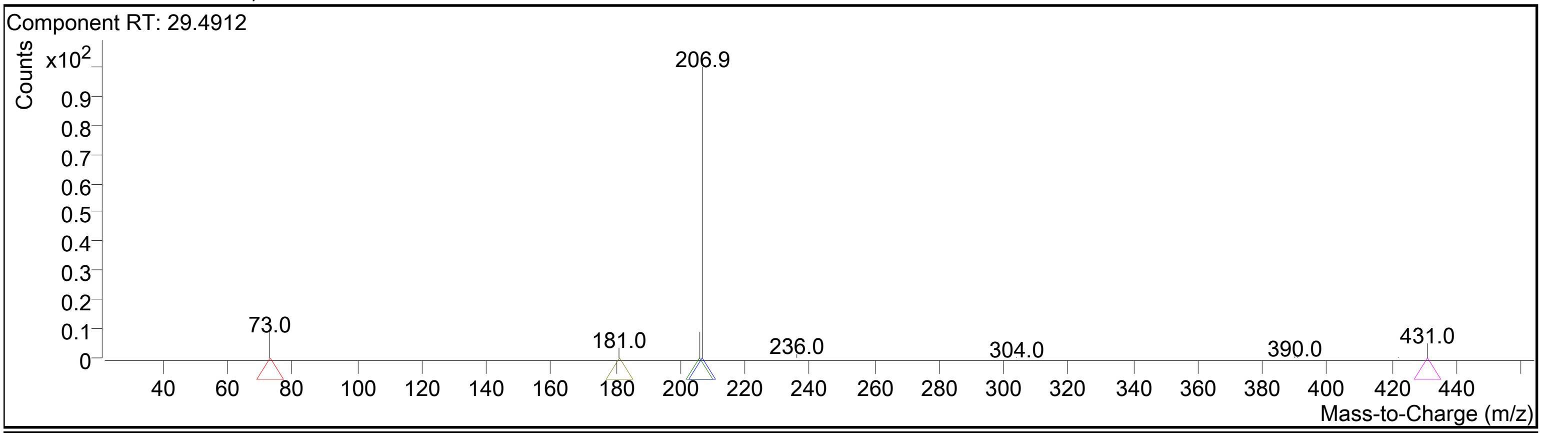

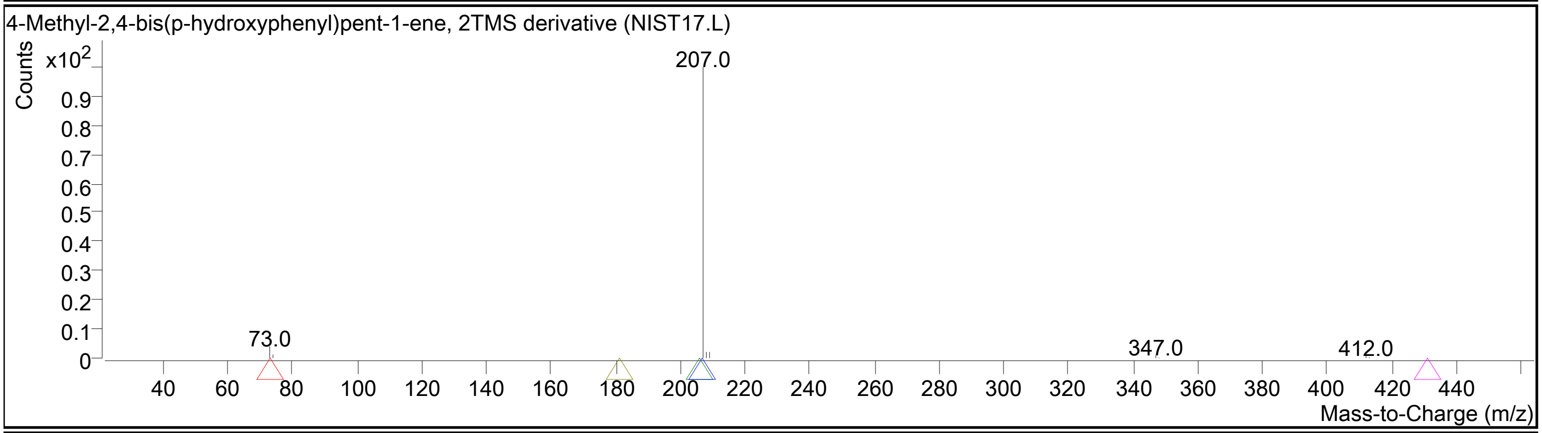

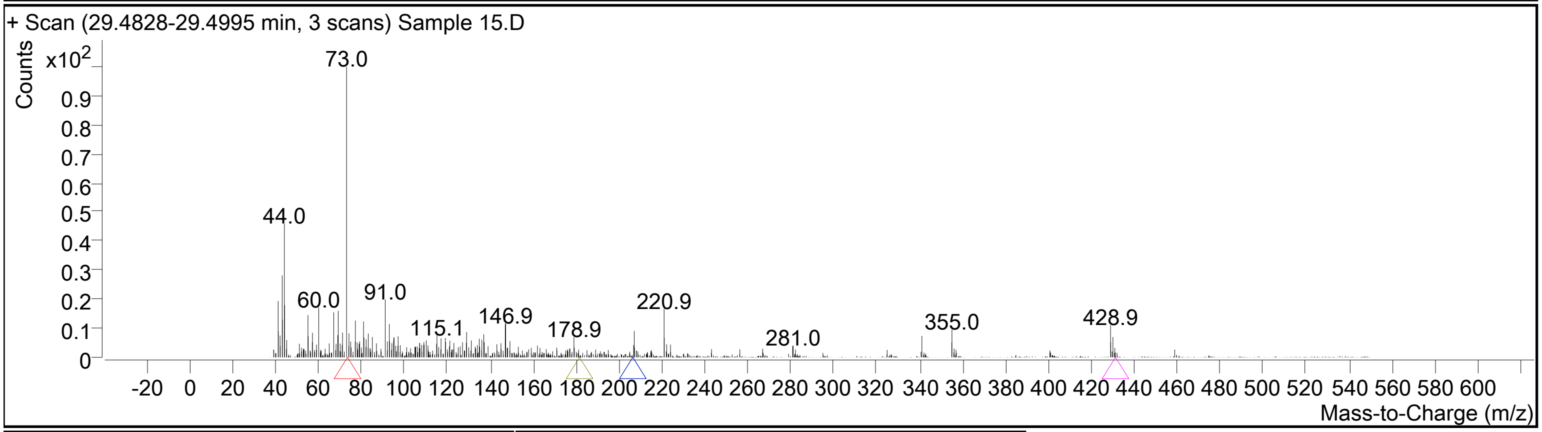

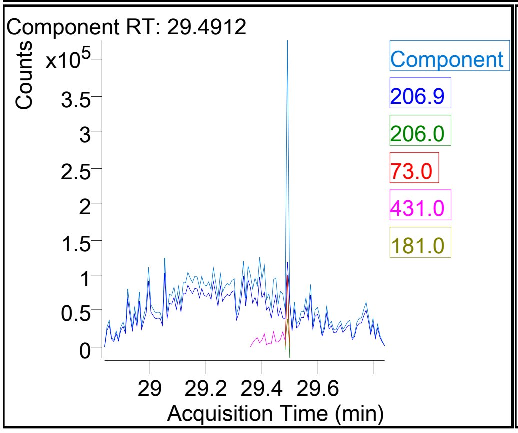

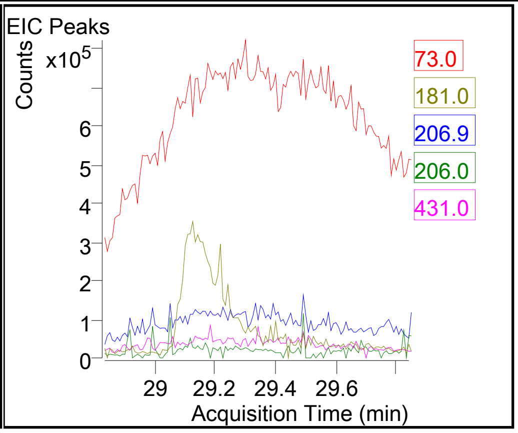

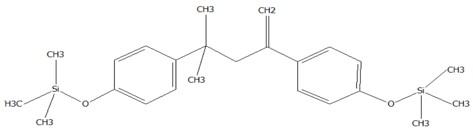

Generated at 12:24 PM on 2/25/2021

Library Search Results - NonTarget Hits with Details

Agilent Technologies

Component RT	Compound Name	Component Area	Match Factor	CAS#	Formula	Estimated Conc.
29.6487	Phthalic acid, butyl cyclobutyl ester	12710075.9	82.7	1000314-89-9	C16H20O4	

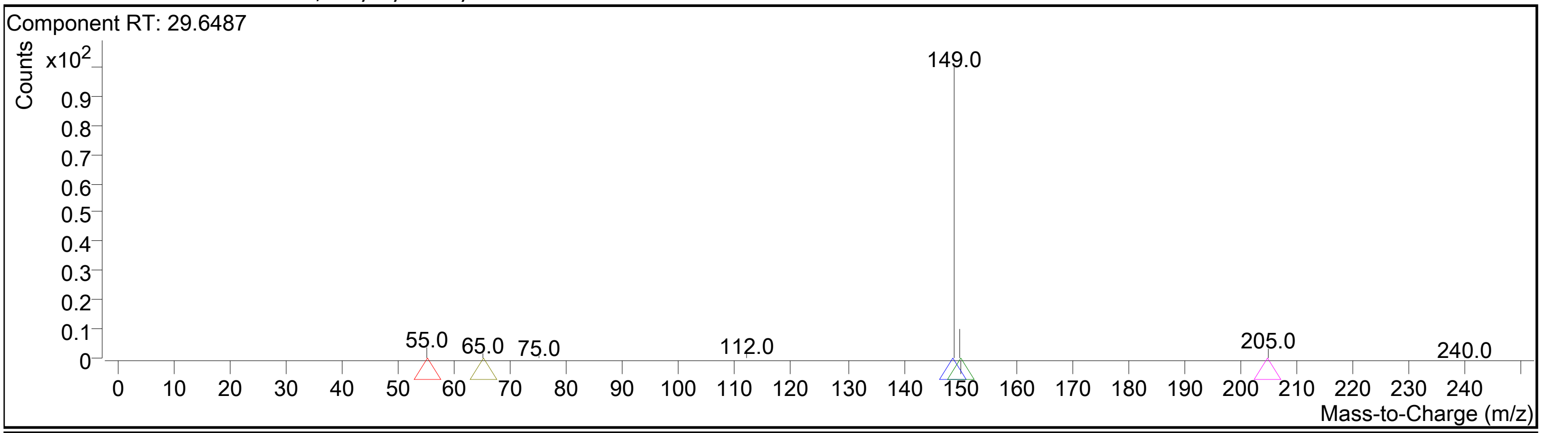

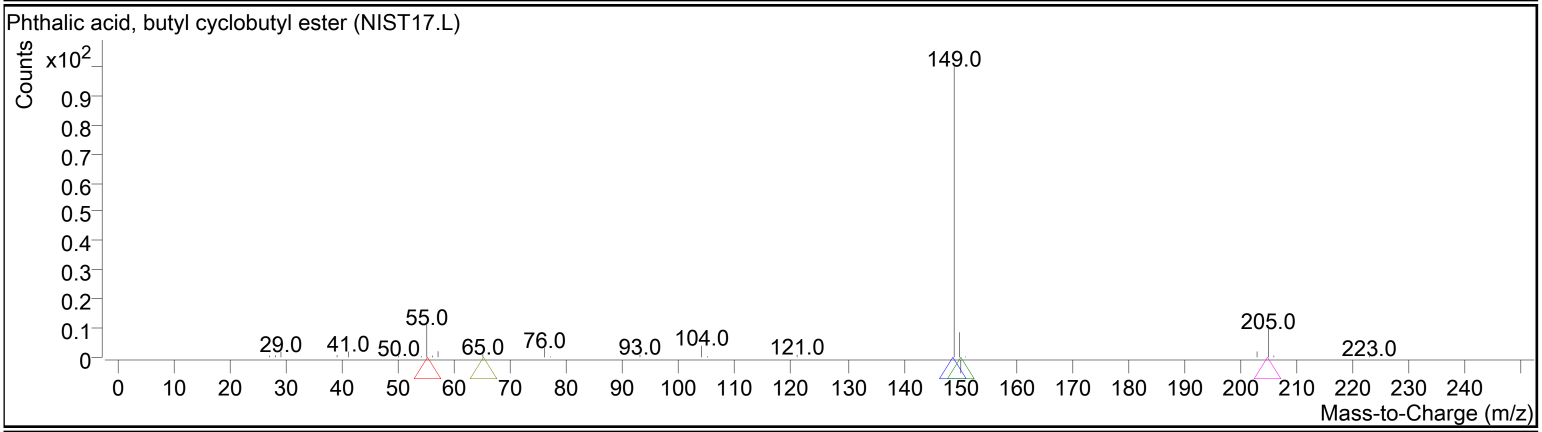

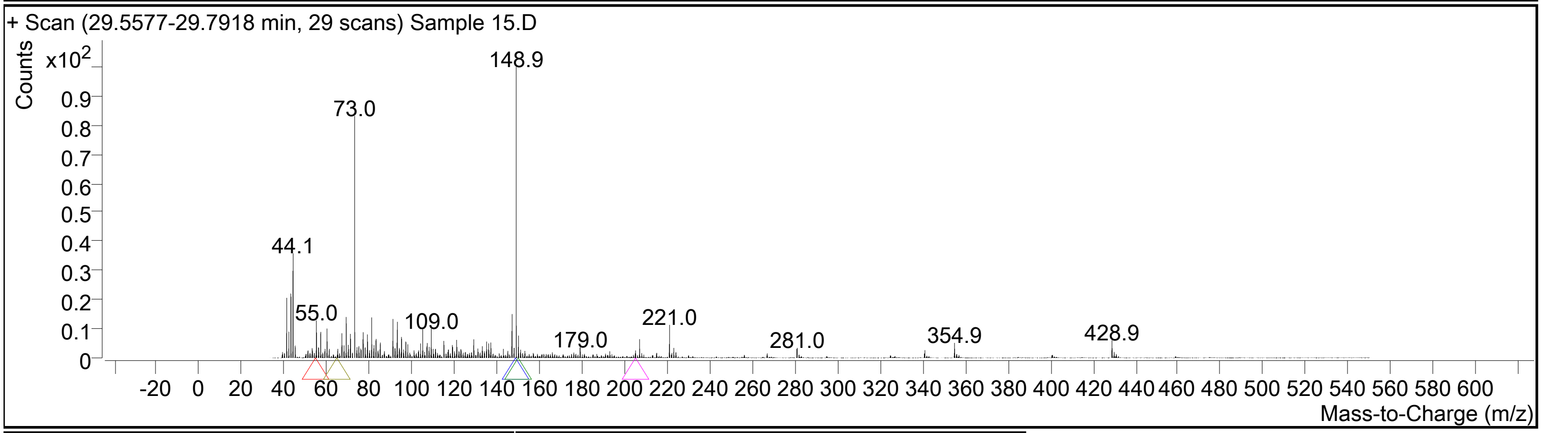

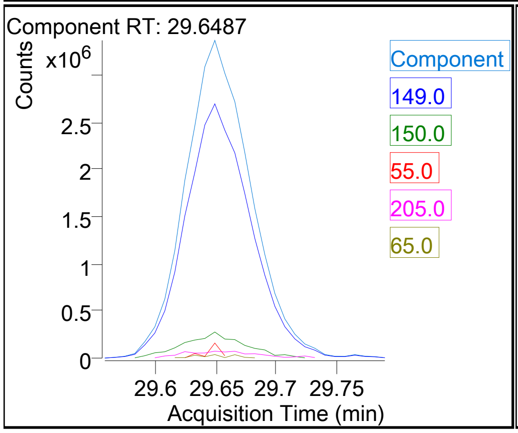

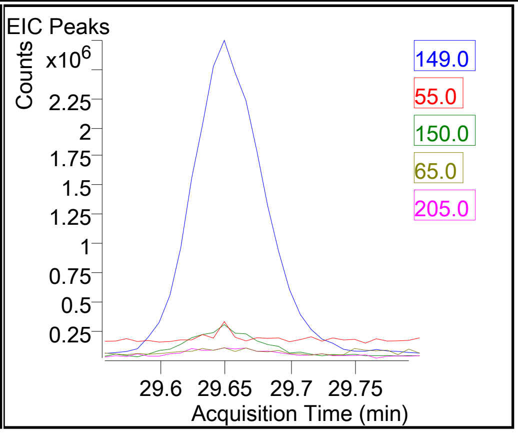

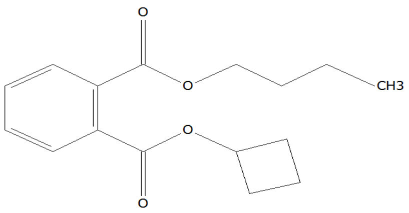

Library Search Results - NonTarget Hits with Details

Agilent Technologies

Component RT	Compound Name	Component Area	Match Factor	CAS#	Formula	Estimated Conc.
30.2811	Kaur-15-ene	33051872.3	77.9	5947-50-2	C20H32	

Component RT: 30.2811

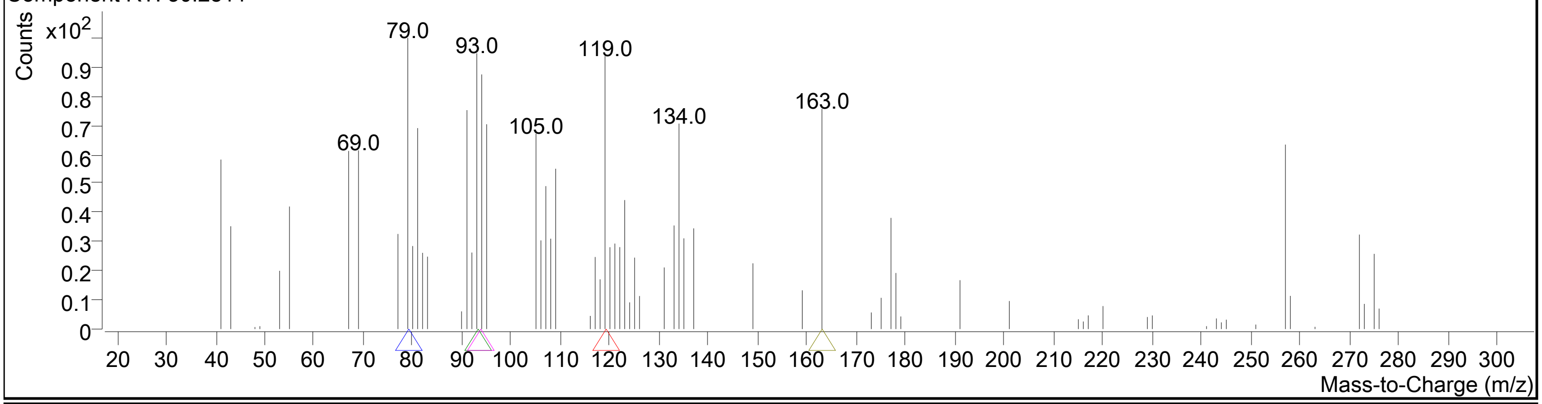

Kaur-15-ene (NIST17.L)

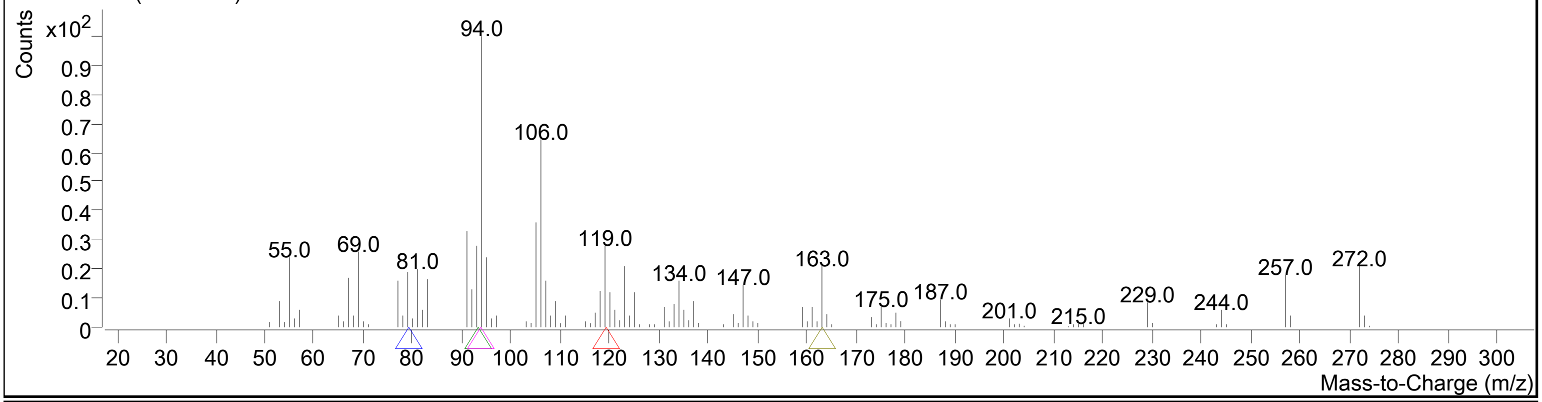

+ Scan (30.2478-30.3632 min, 14 scans) Sample 15.D

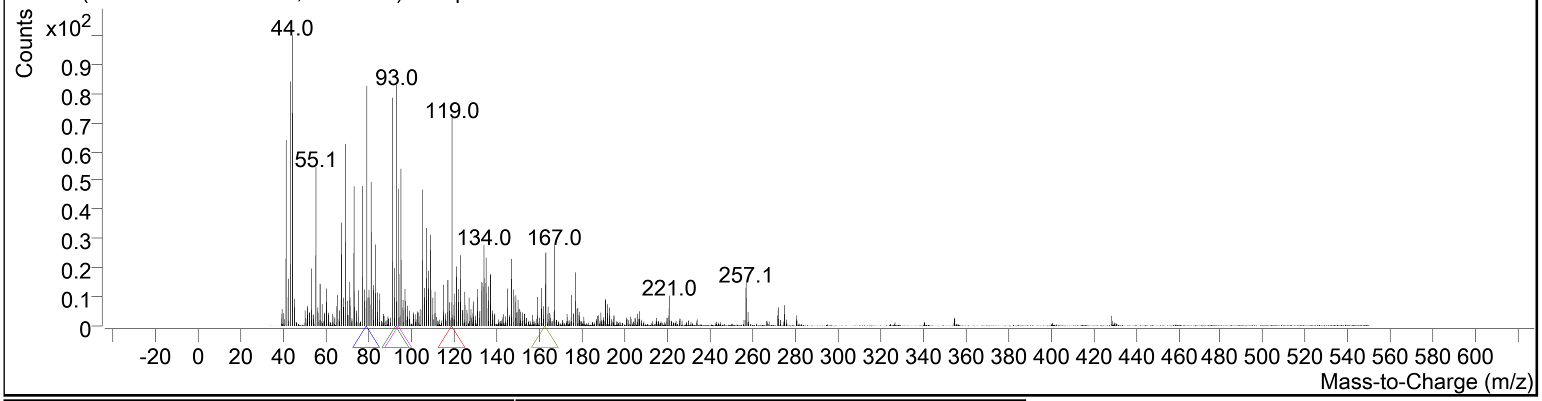

Component RT: 30.2811

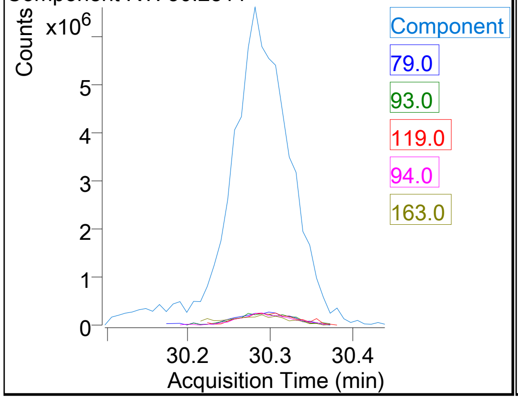

EIC Peaks

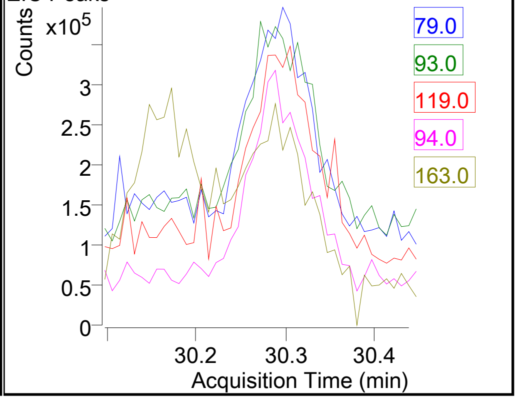

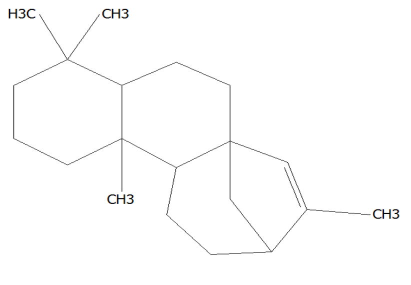

Component RT	Compound Name	Component Area	Match Factor	CAS#	Formula	Estimated Conc.
30.8526	Cannabidivarol	51391774.3	65.4	24274-48-4	C19H26O2	

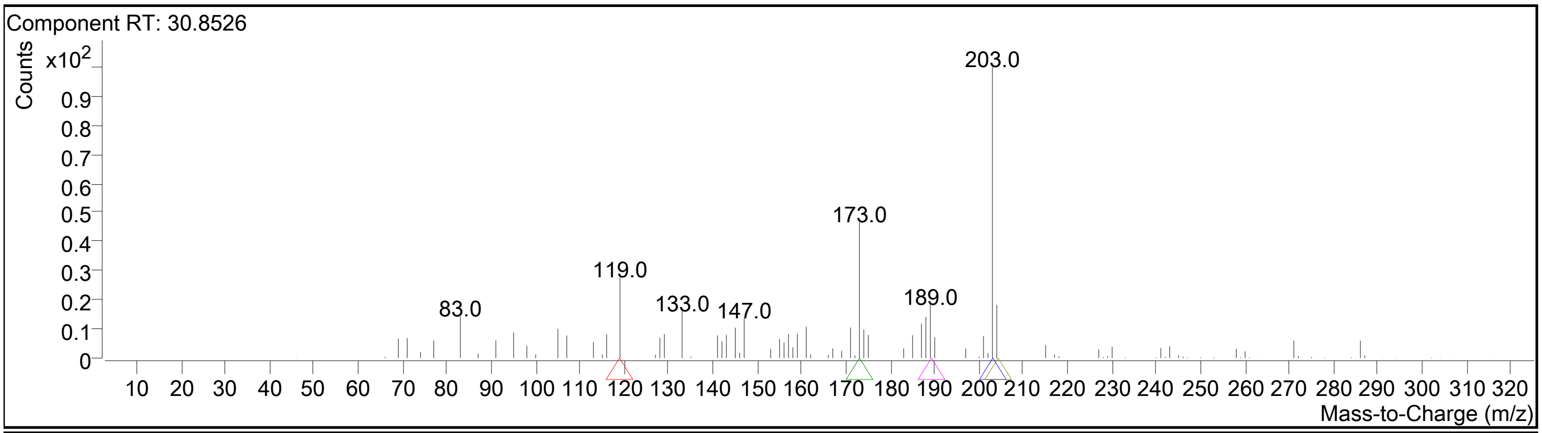

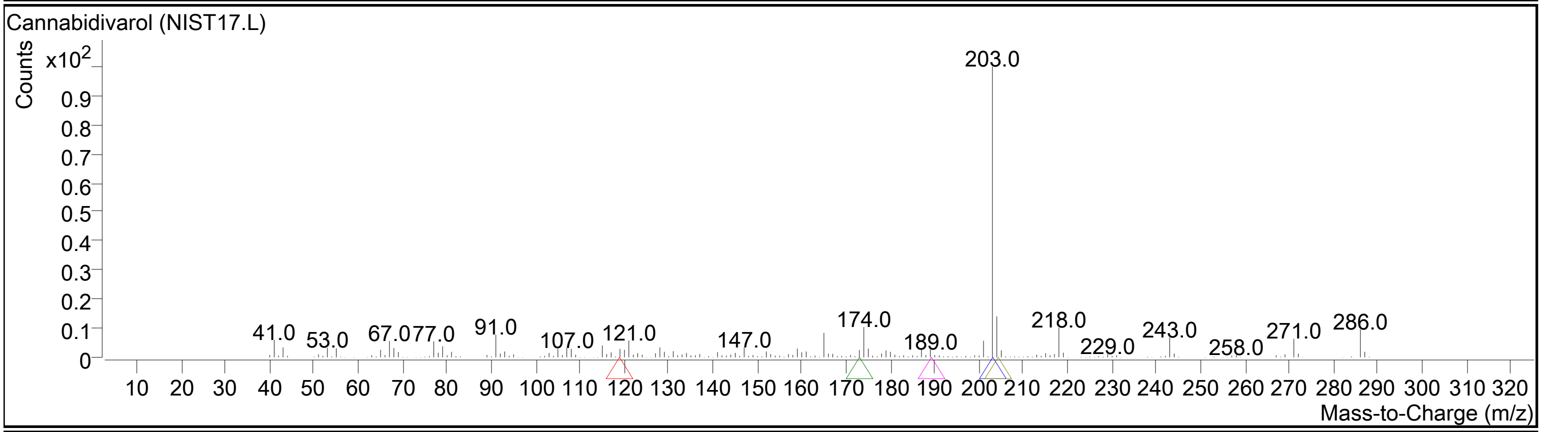

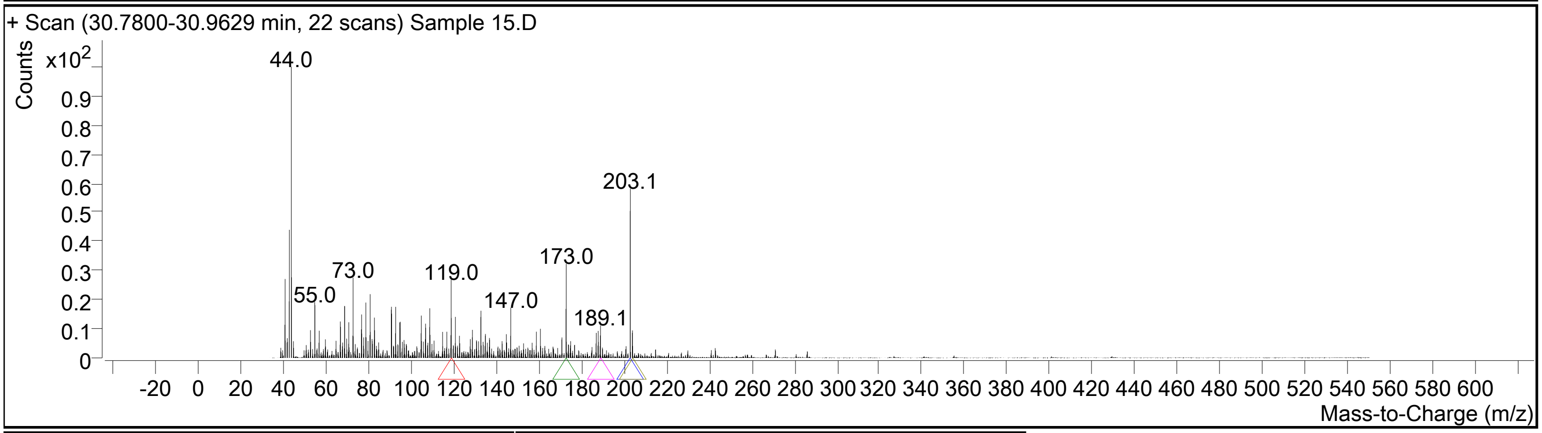

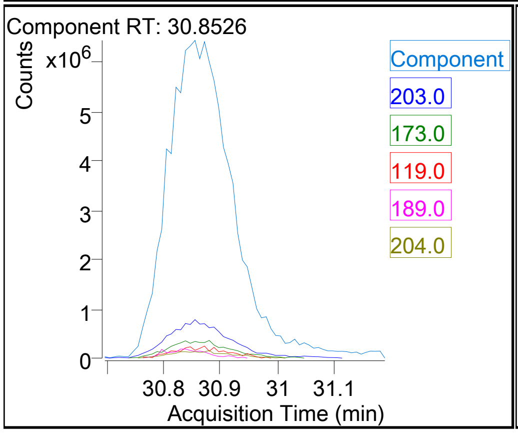

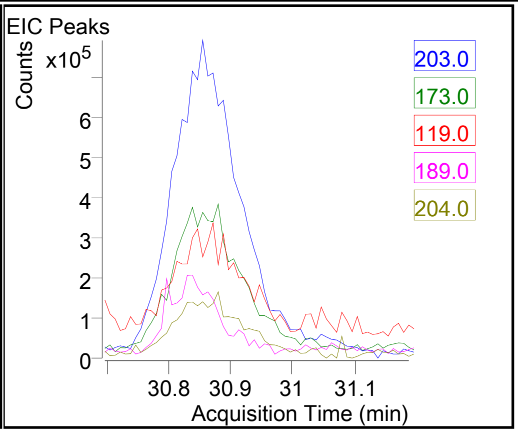

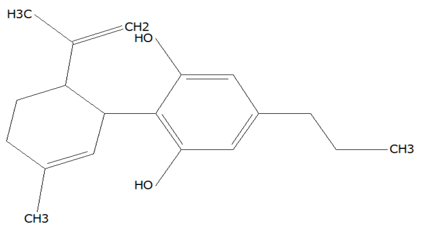

Library Search Results - NonTarget Hits with Details

Component RT	Compound Name	Component Area	Match Factor	CAS#	Formula	Estimated Conc.
31.4334	Verimol K	12745130.2	74.4	85985-75-7	C14H12O4	

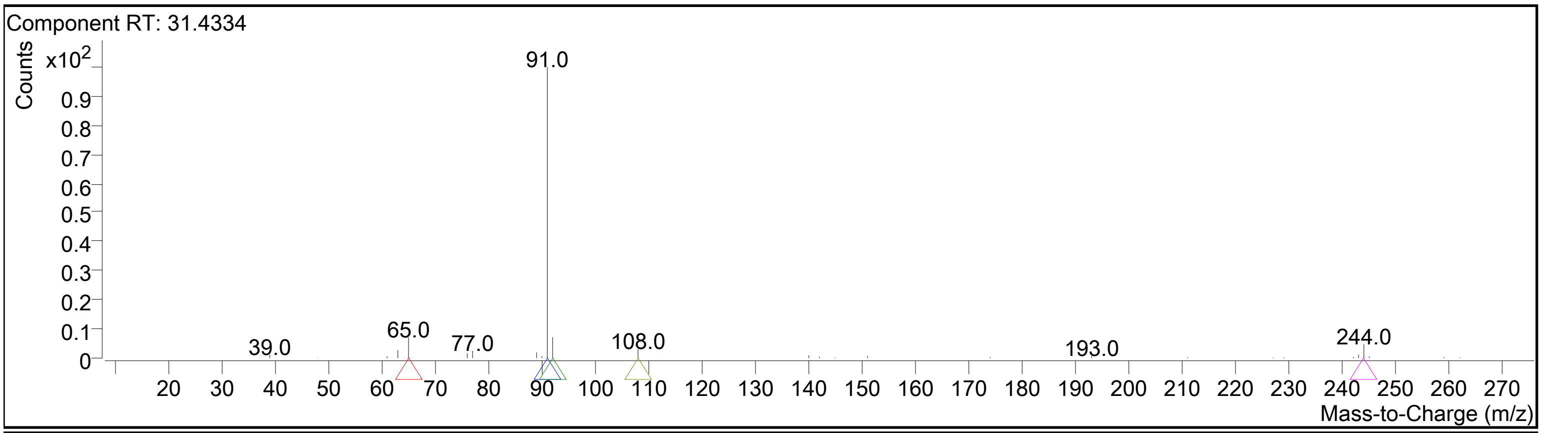

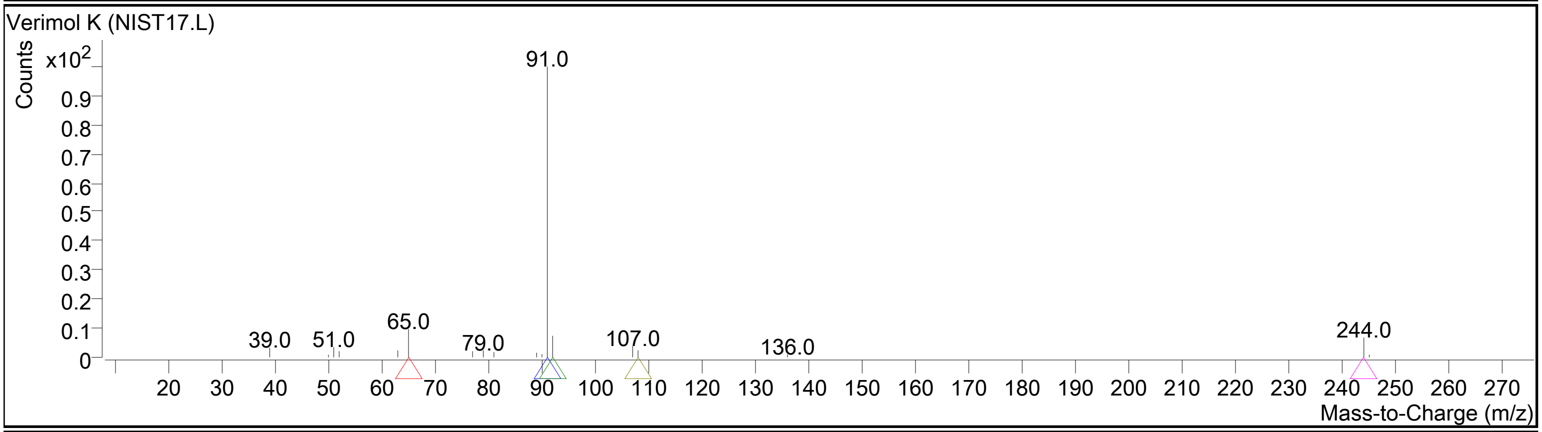

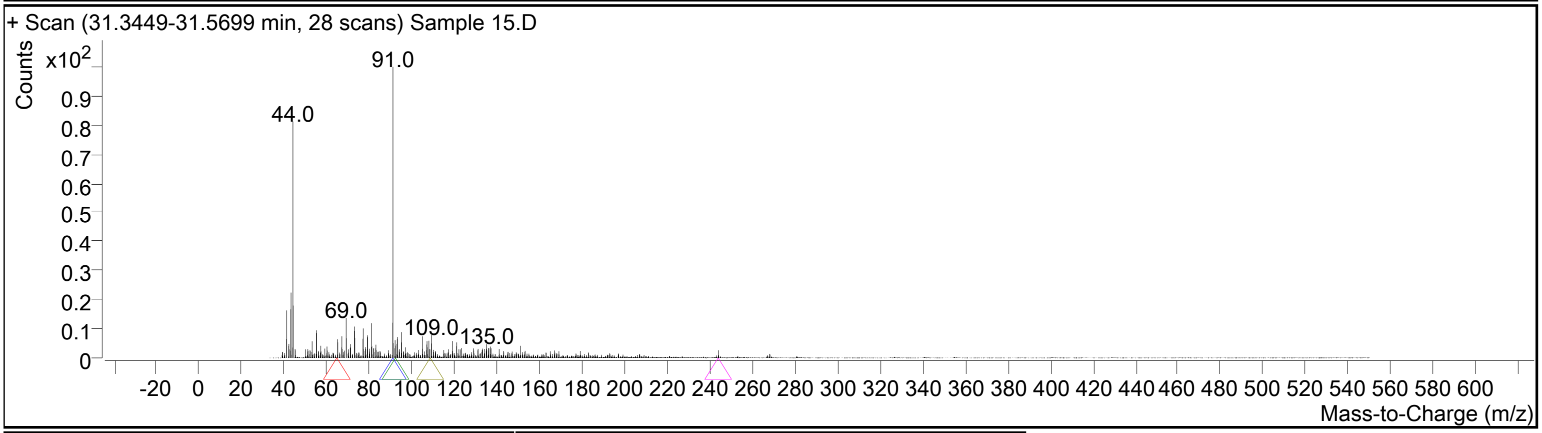

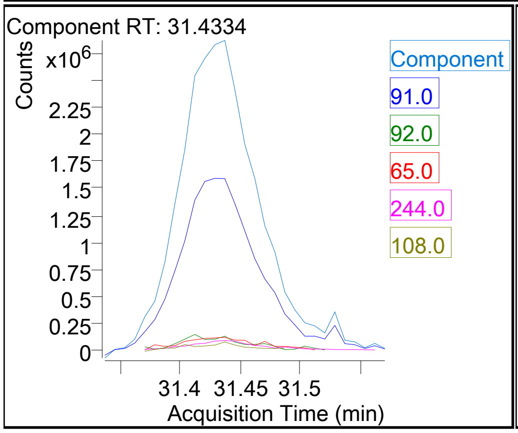

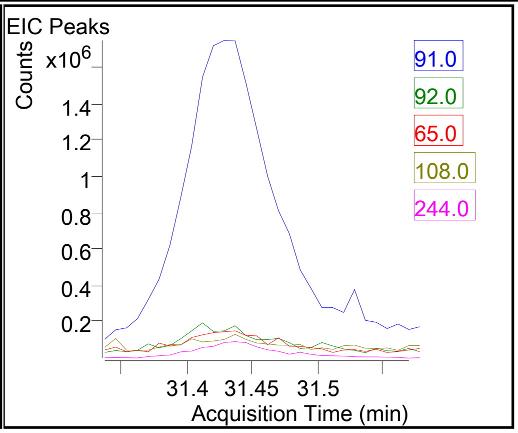

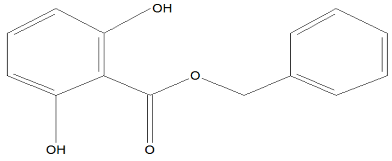

Component RT	Compound Name	Component Area	Match Factor	CAS#	Formula	Estimated Conc.
31.7823	7-Isopropyl-1,1,4a-trimethyl-1,2,3,4,4a,9,10,10a-octahydrophenanthrene	56661634.8	94.7	109680-01-5	C20H30	

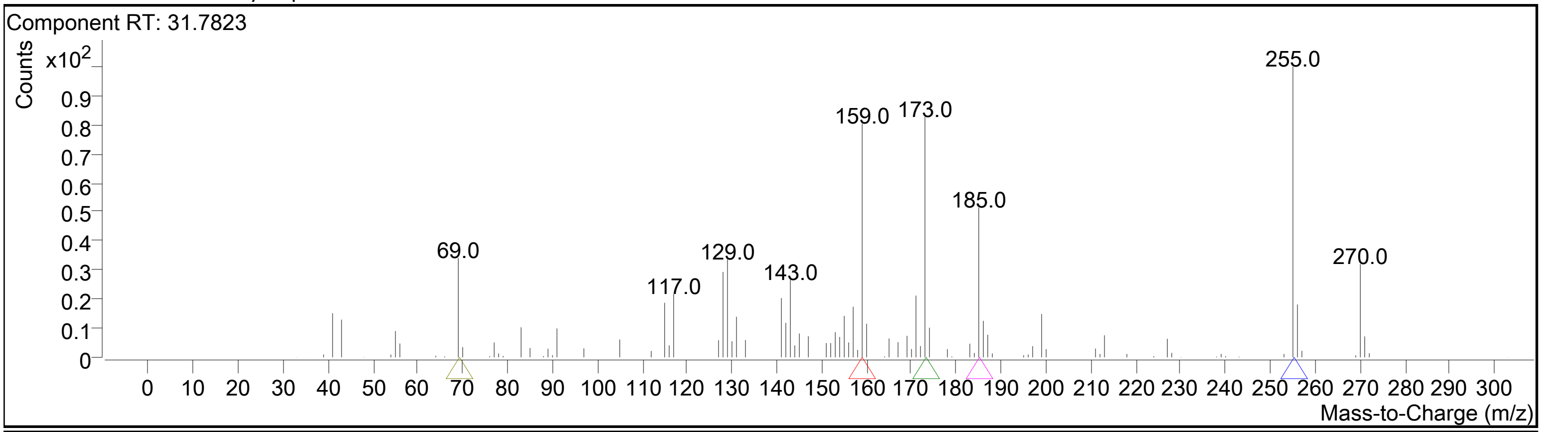

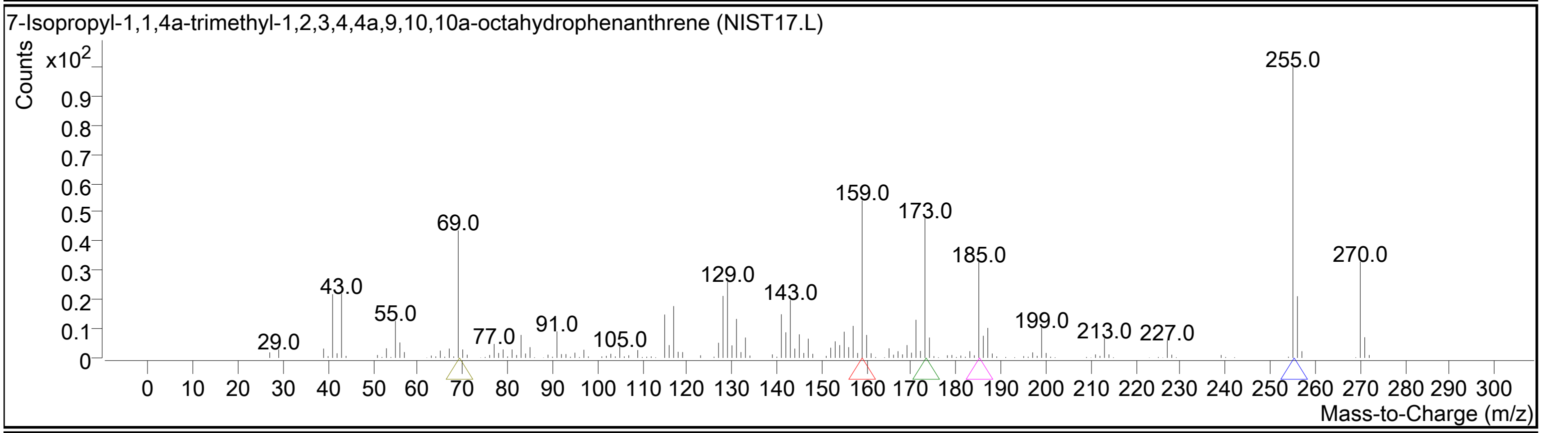

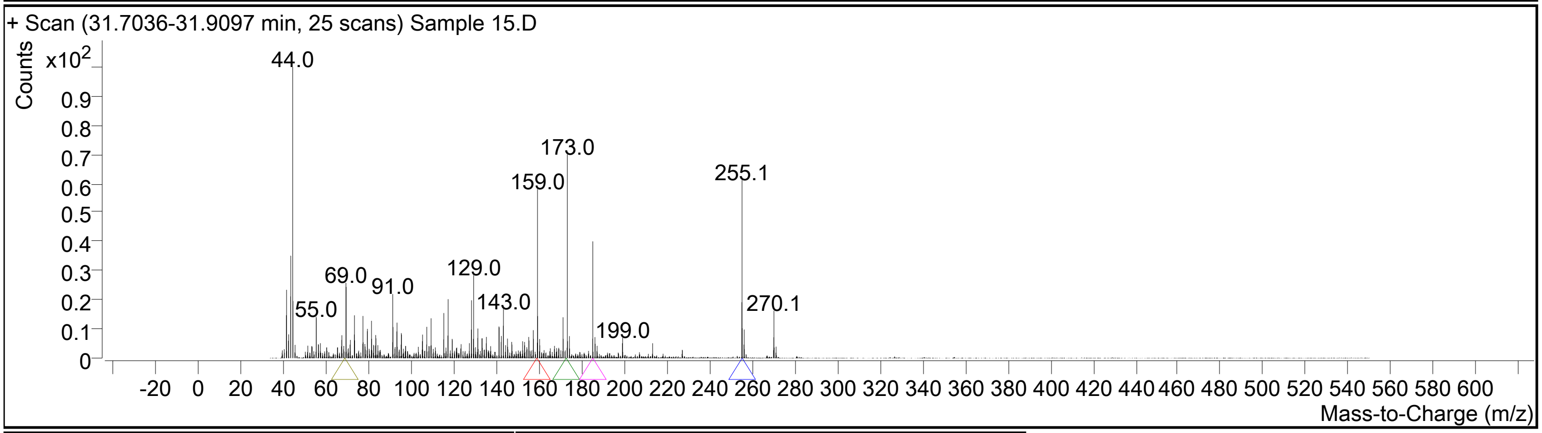

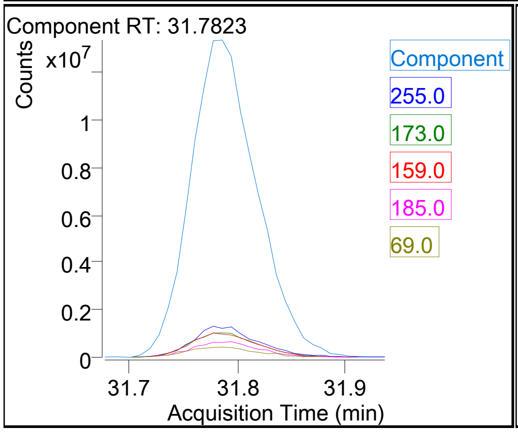

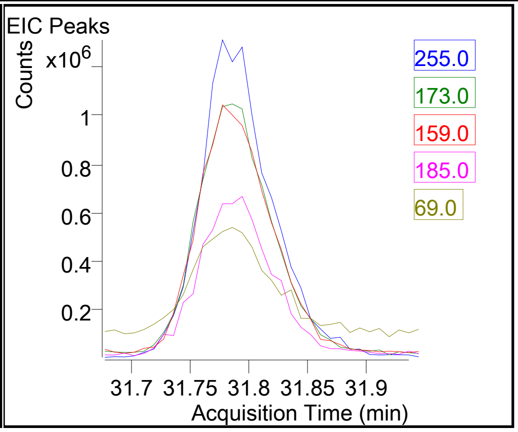

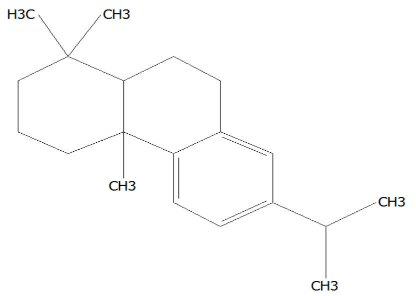

Generated at 12:24 PM on 2/25/2021

Component RT	Compound Name	Component Area	Match Factor	CAS#	Formula	Estimated Conc.
32.6676	(3R,6R)-3-Hydroperoxy-3-methyl-6-(prop-1-en-2-yl)cyclohex-1-ene	10718472.6	68.4	77026-88-1	C10H16O2	

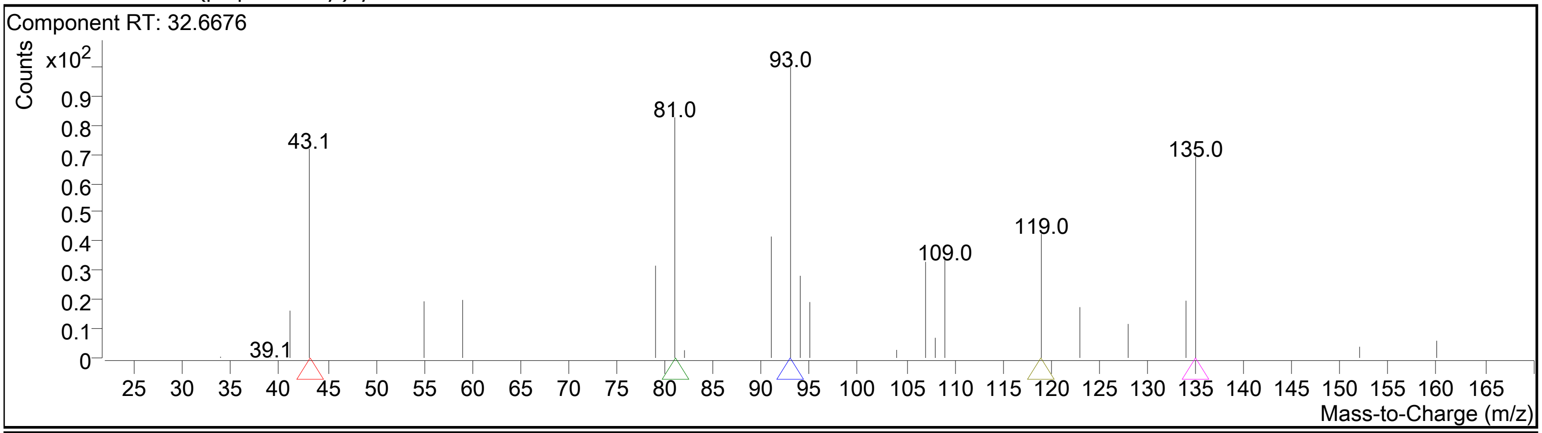

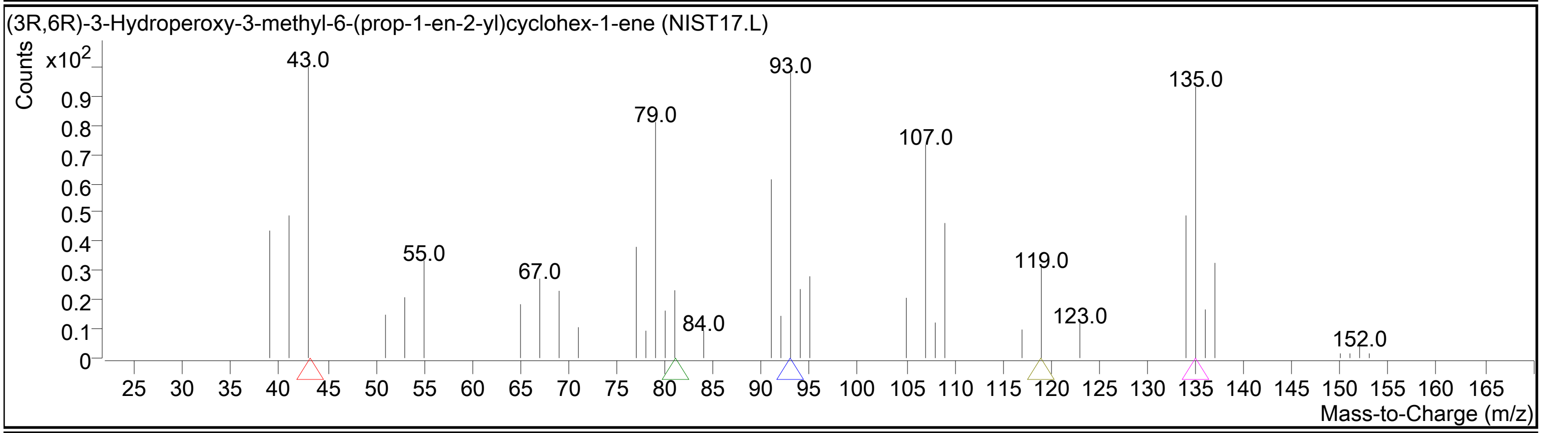

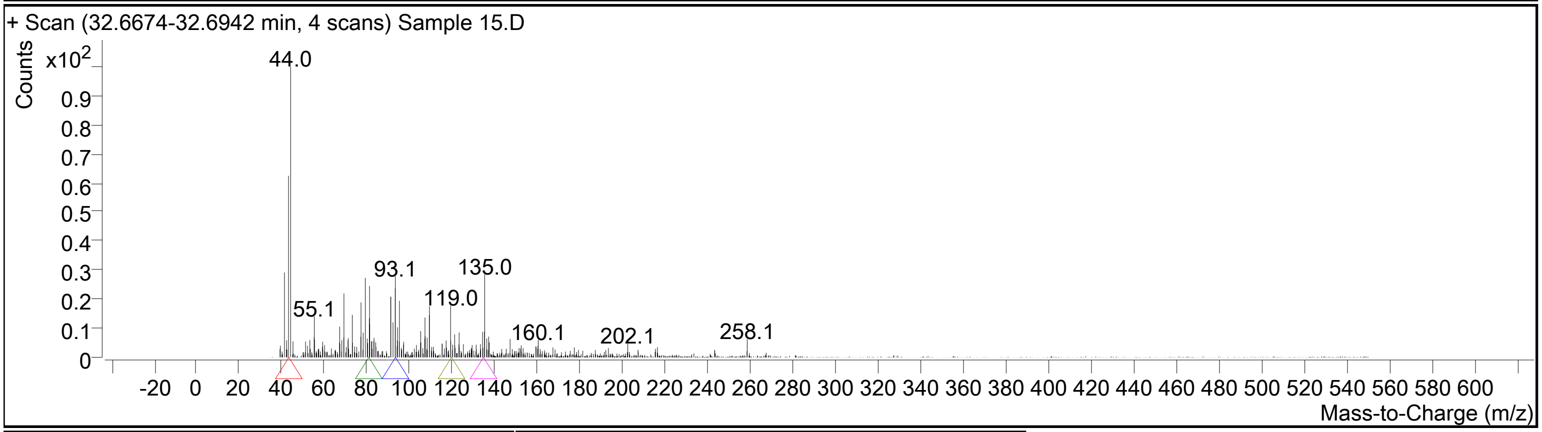

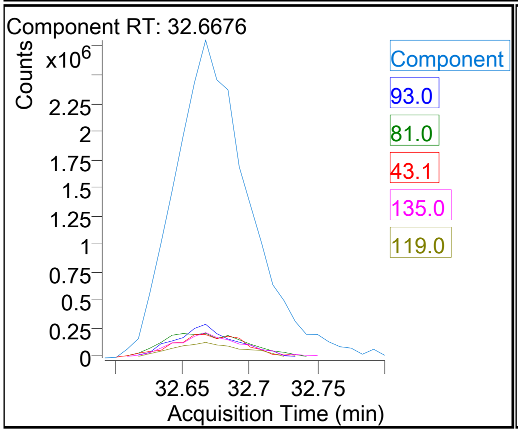

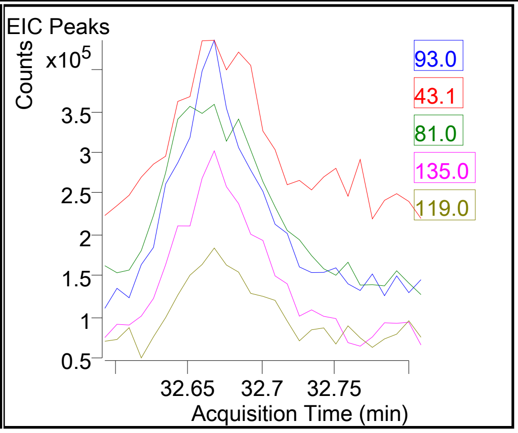

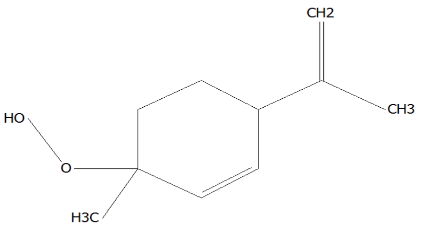

Component RT	Compound Name	Component Area	Match Factor	CAS#	Formula	Estimated Conc.
33.2000	9,12,15-Octadecatrienoic acid, methyl ester, (Z,Z,Z)-	21305253.3	74.8	301-00-8	C19H32O2	

Component RT: 33.2000

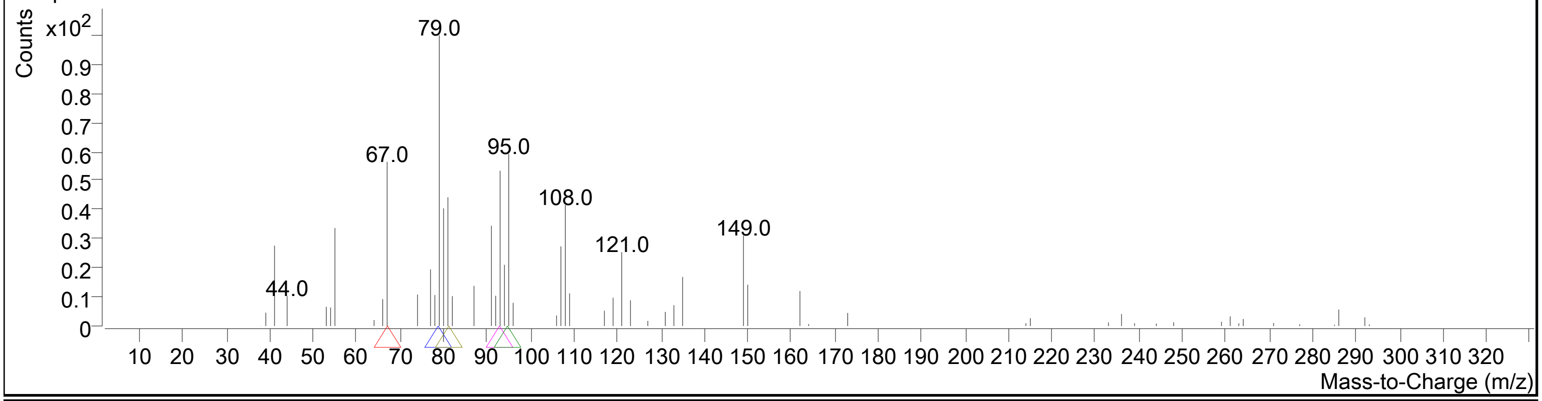

9,12,15-Octadecatrienoic acid, methyl ester, (Z,Z,Z)- (NIST17.L)

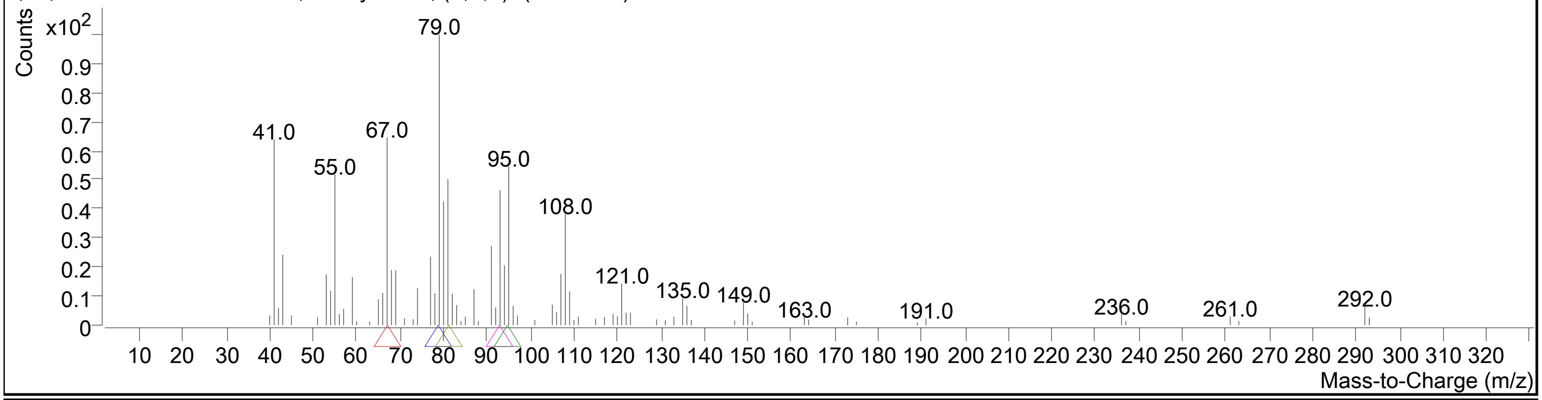

+ Scan (33.1746-33.2328 min, 8 scans) Sample 15.D

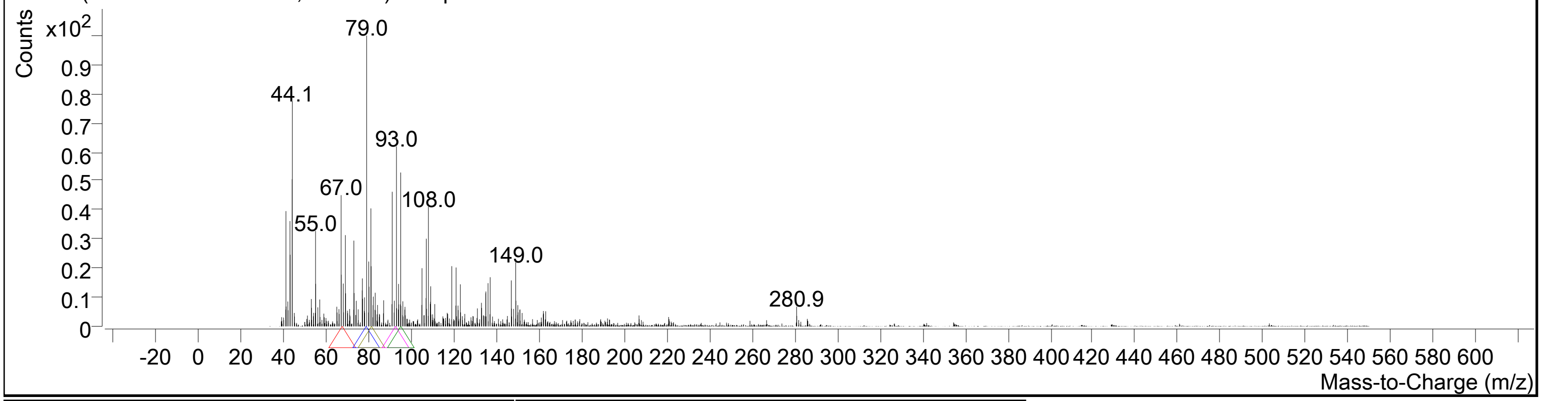

Component RT: 33.2000

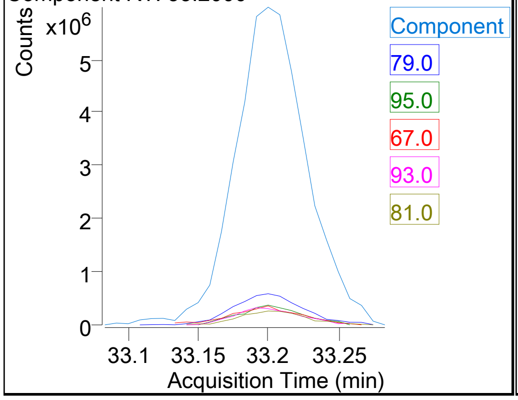

EIC Peaks

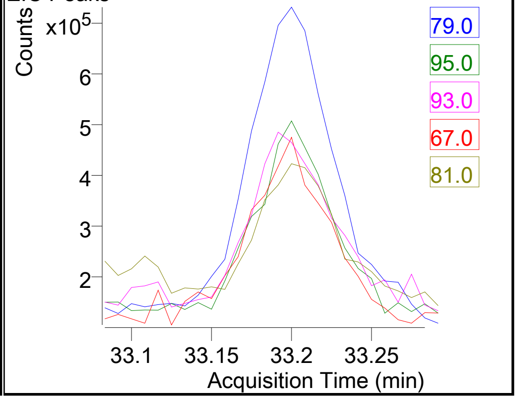

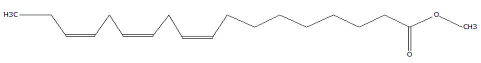

Library Search Results - NonTarget Hits with Details

Component RT	Compound Name	Component Area	Match Factor	CAS#	Formula	Estimated Conc.
35.4762	4,6-Dimethoxy-1-naphthaldehyde	96326222.1	70.0	65565-33-5	C13H12O3	

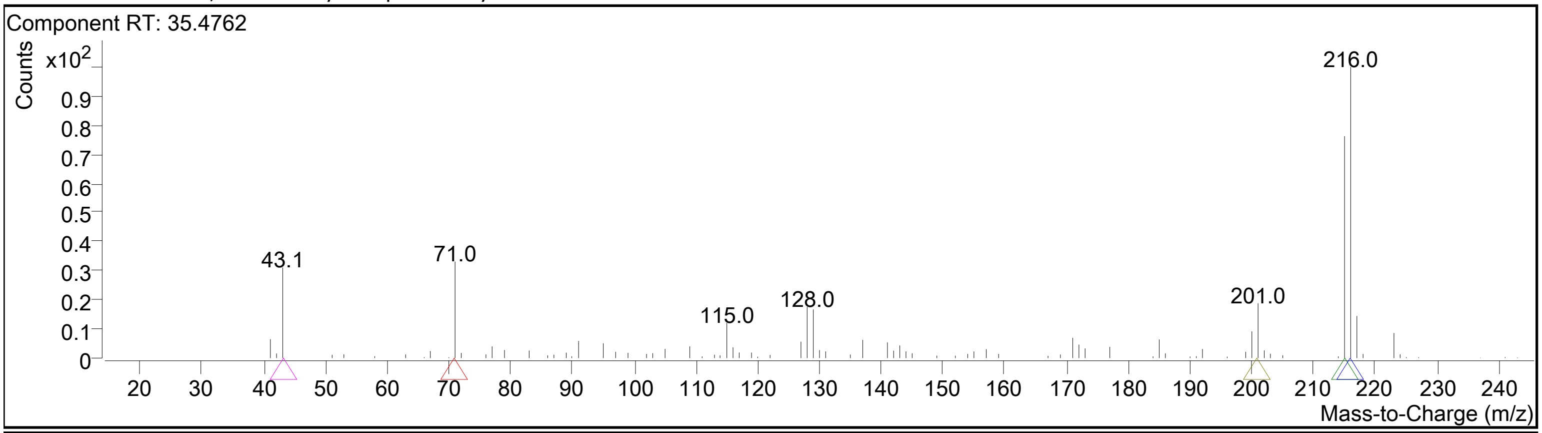

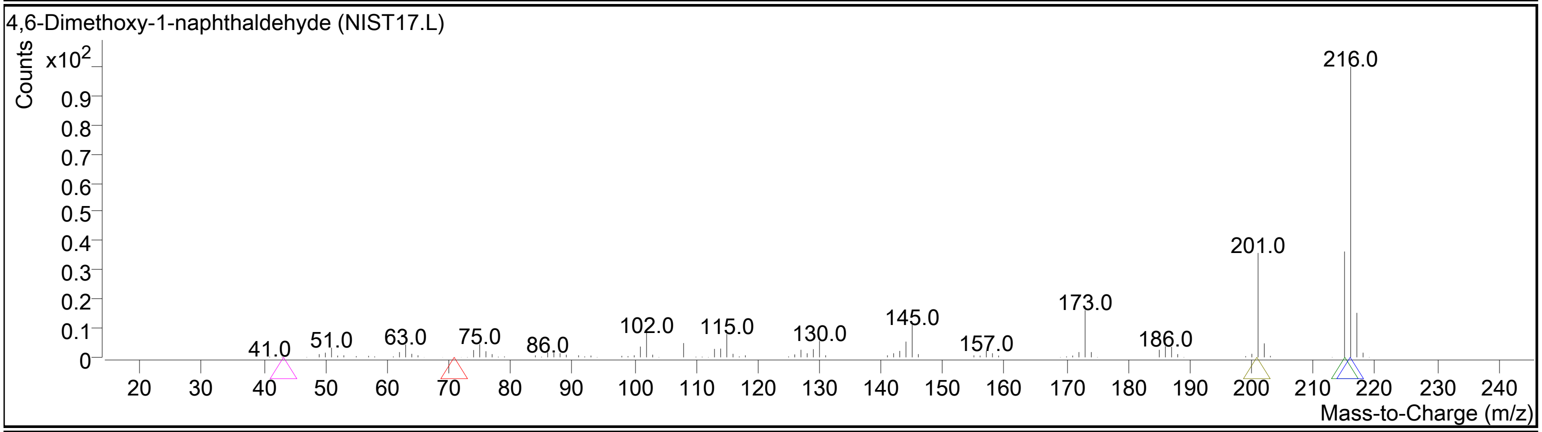

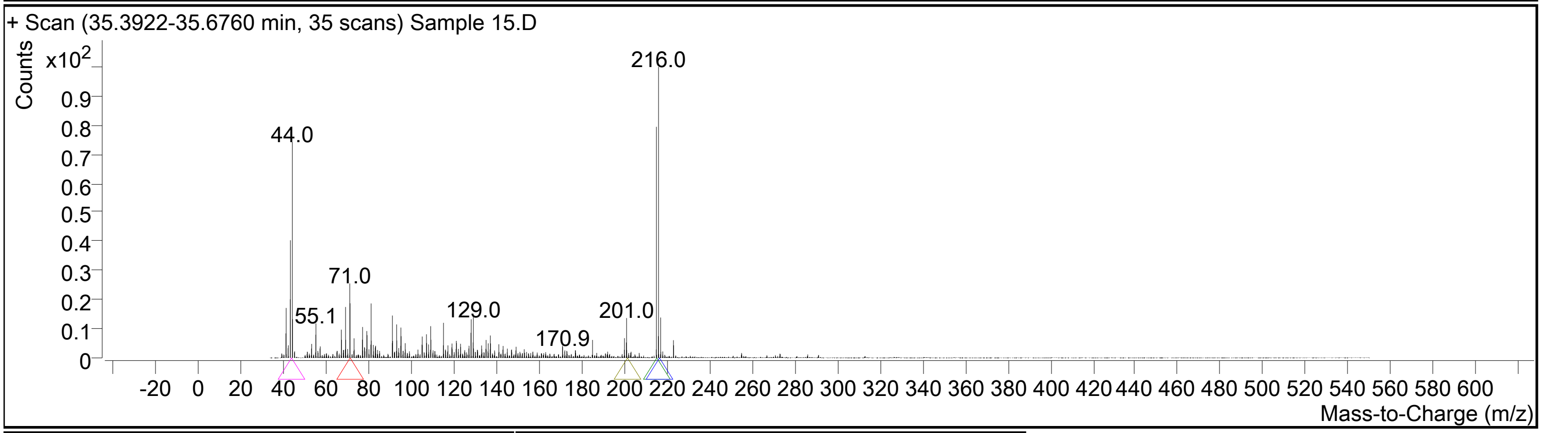

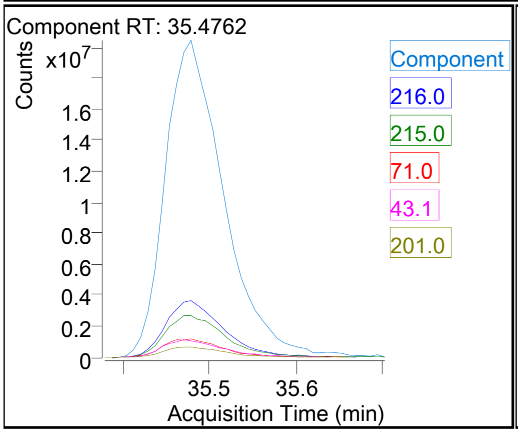

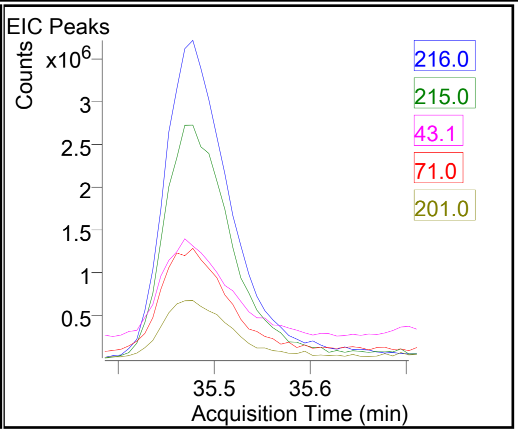

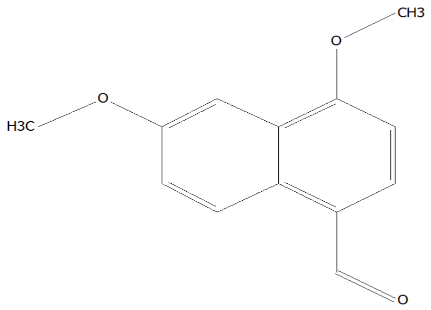

Agilent Technologies

Component RT	Compound Name	Component Area	Match Factor	CAS#	Formula	Estimated Conc.
36.2537	Kaur-16-en-18-ol, (4.alpha.)-	102194459.1	85.5	2300-11-0	C20H32O	

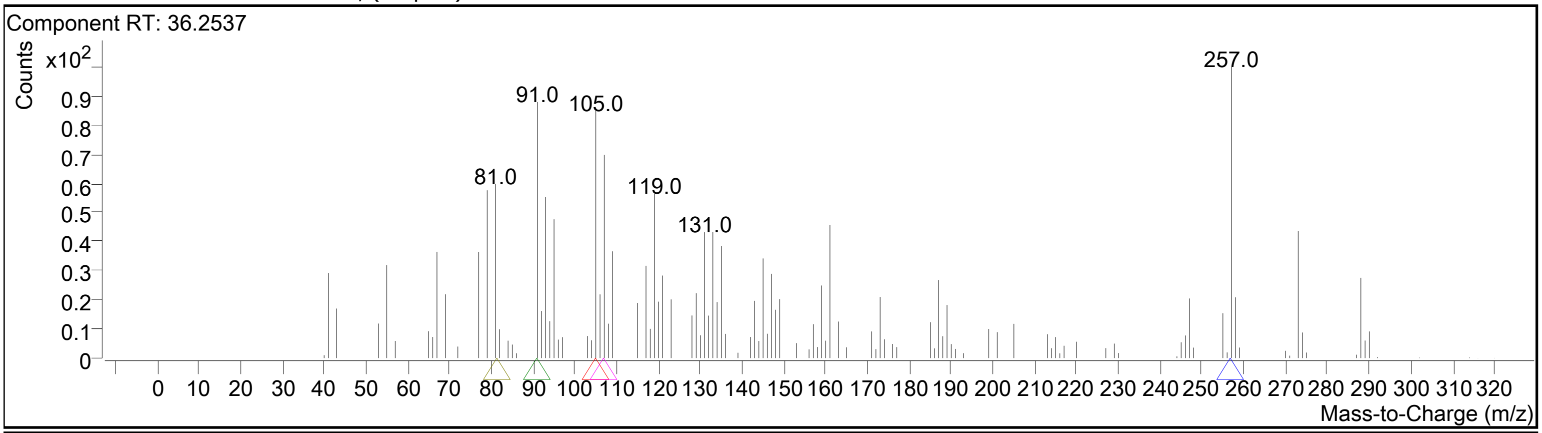

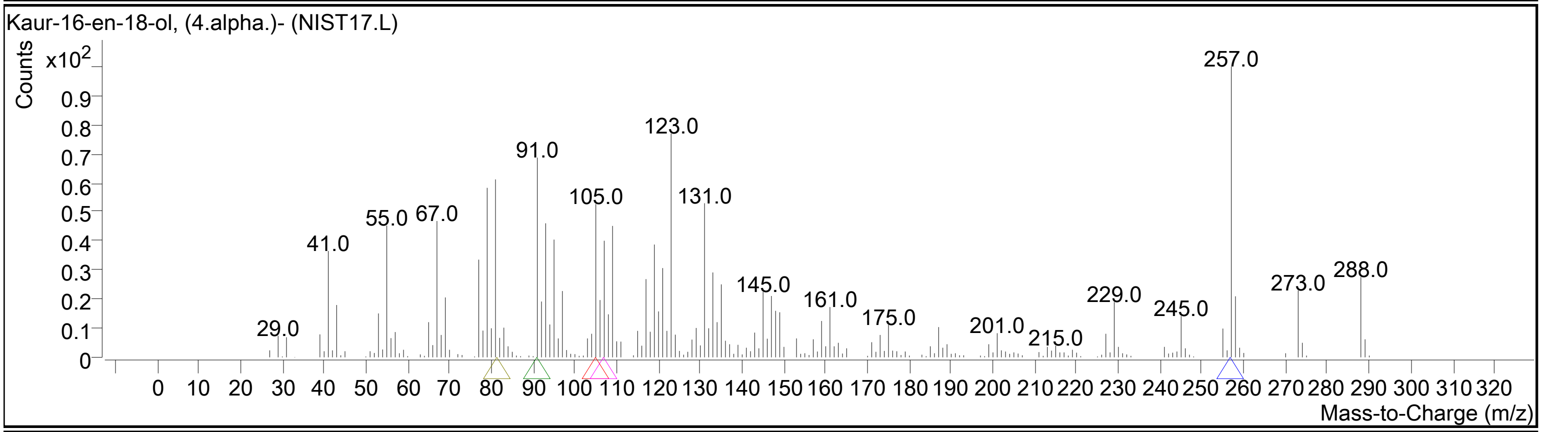

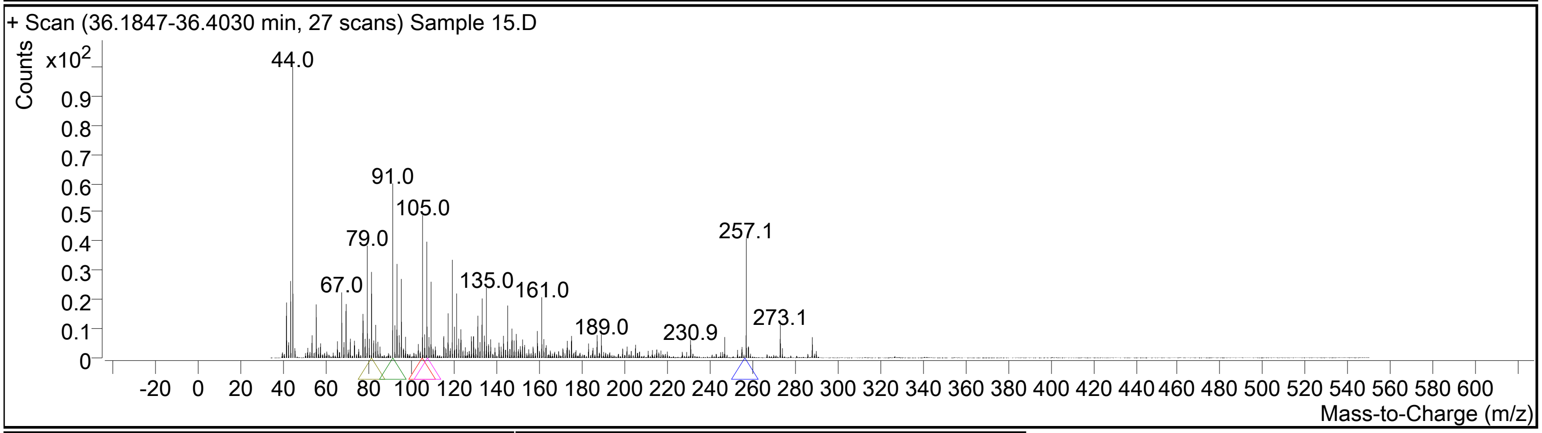

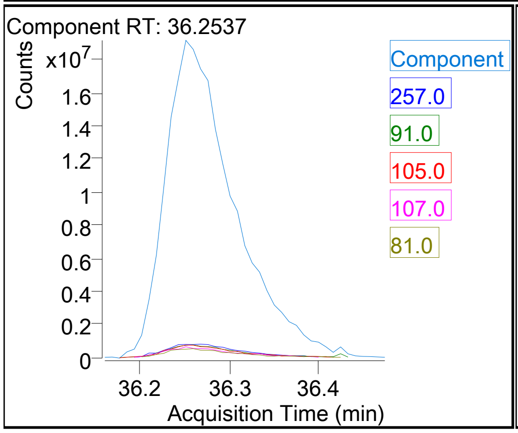

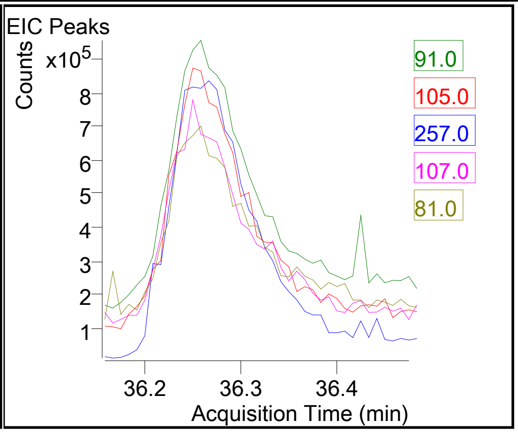

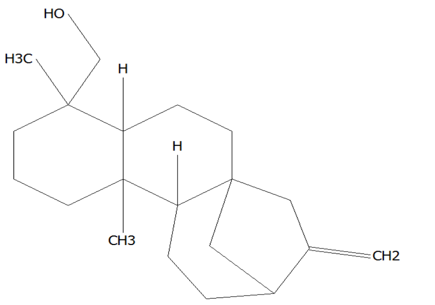

Component RT	Compound Name	Component Area	Match Factor	CAS#	Formula	Estimated Conc.
37.4176	4-Hydroxy-4'-methyldiphenylamine, O-trimethylsilyl ether	215856697.7	67.5	1000417-59-7	C16H21NOSi	

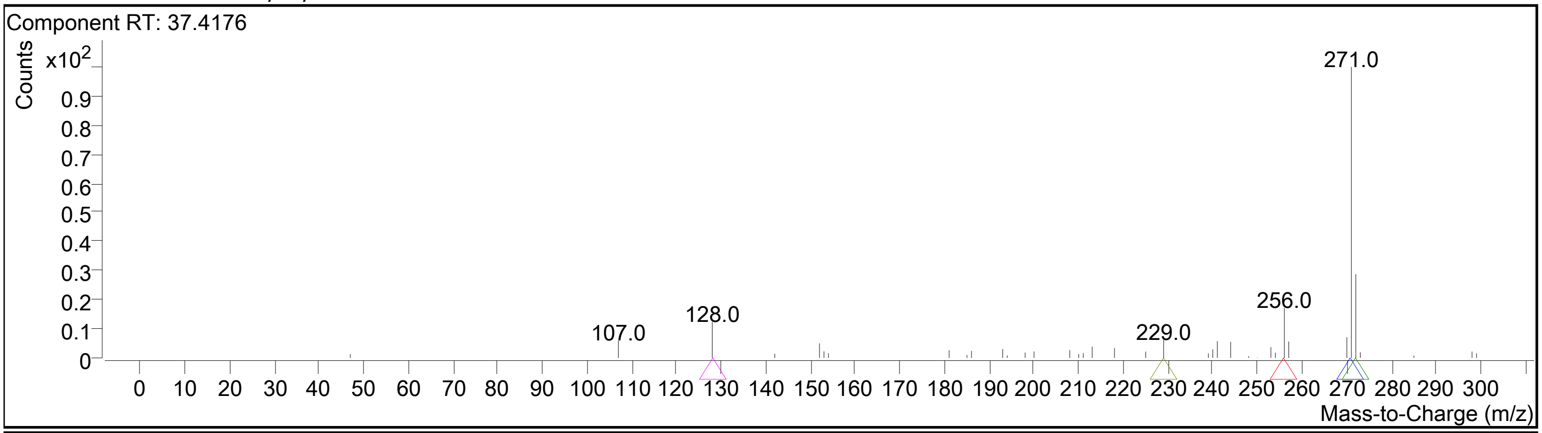

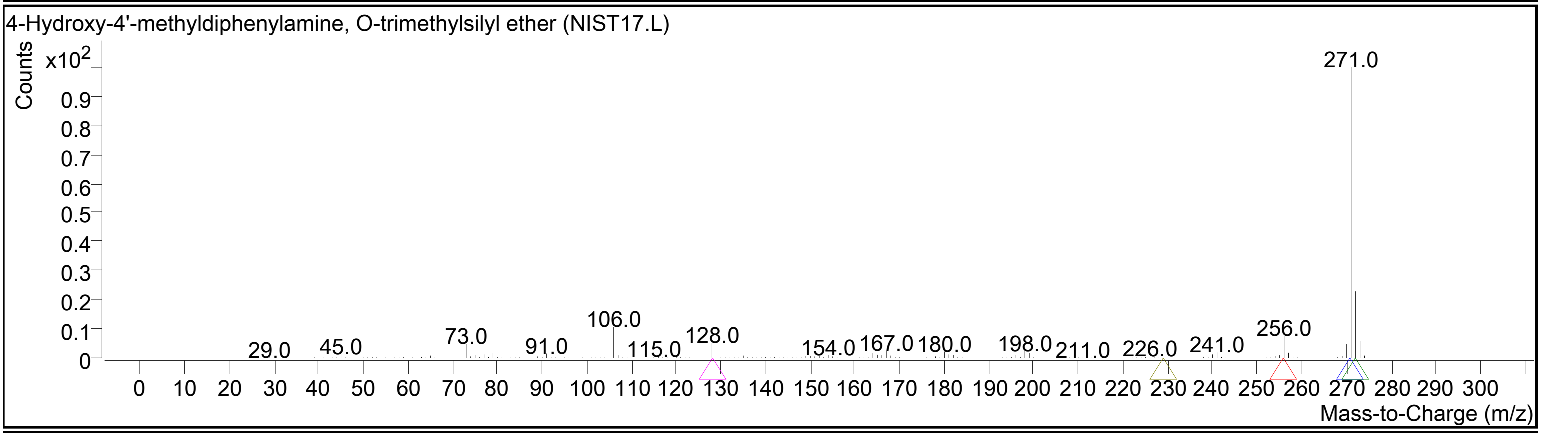

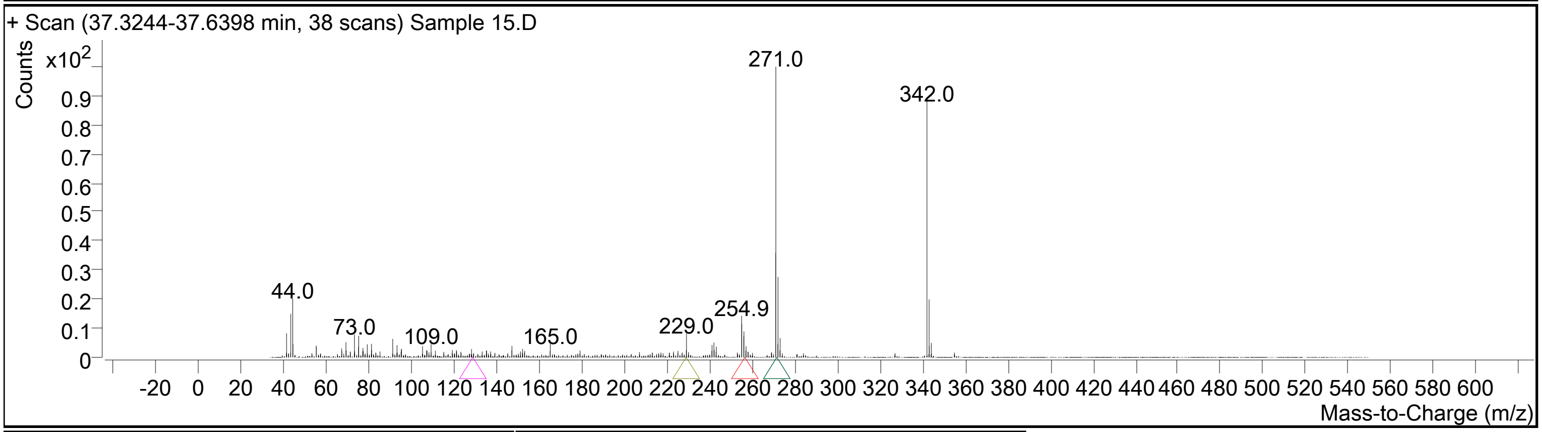

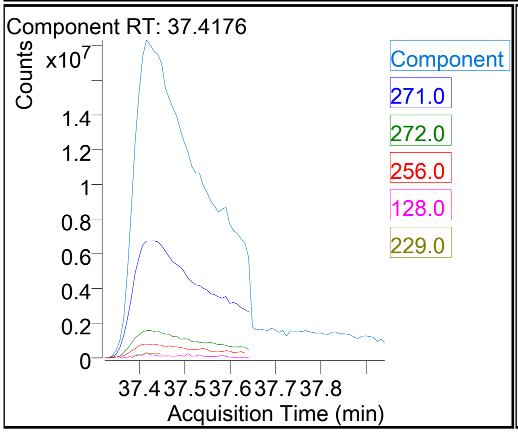

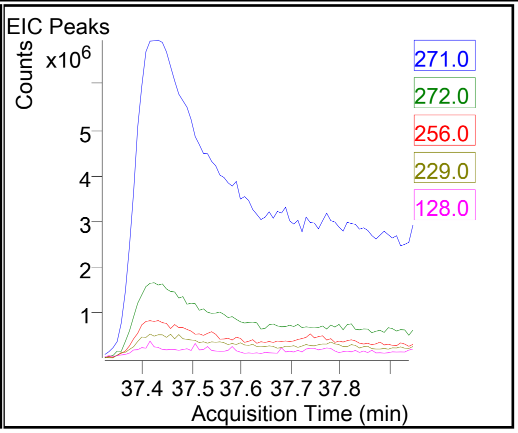

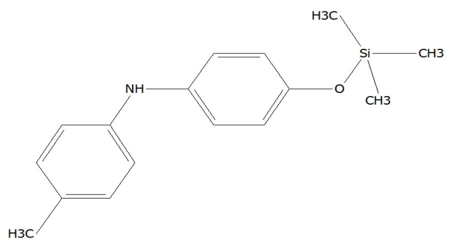

Component RT	Compound Name	Component Area	Match Factor	CAS#	Formula	Estimated Conc.
38.0240	3-Trifluoromethylbenzylamine, N,N-diundecyl	13229292.3	71.2	1000310-29-9	C30H52F3N	

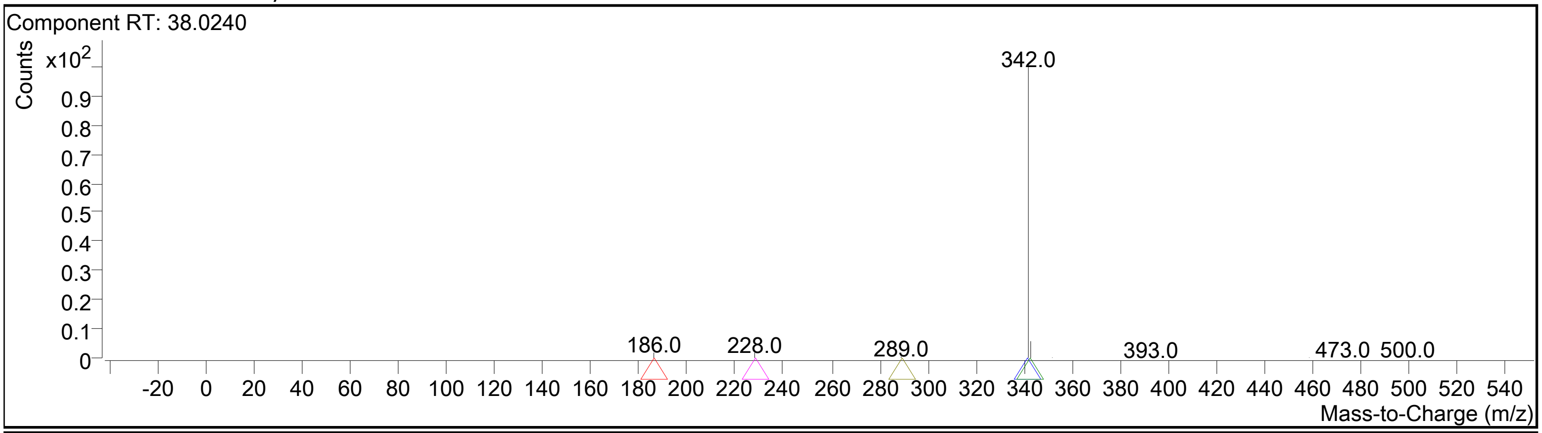

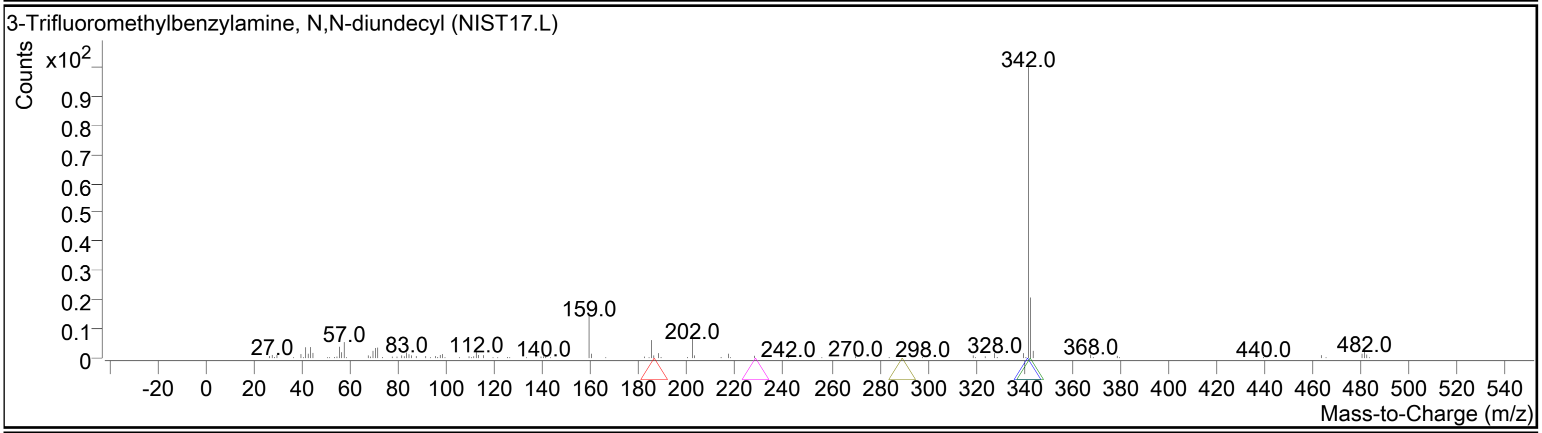

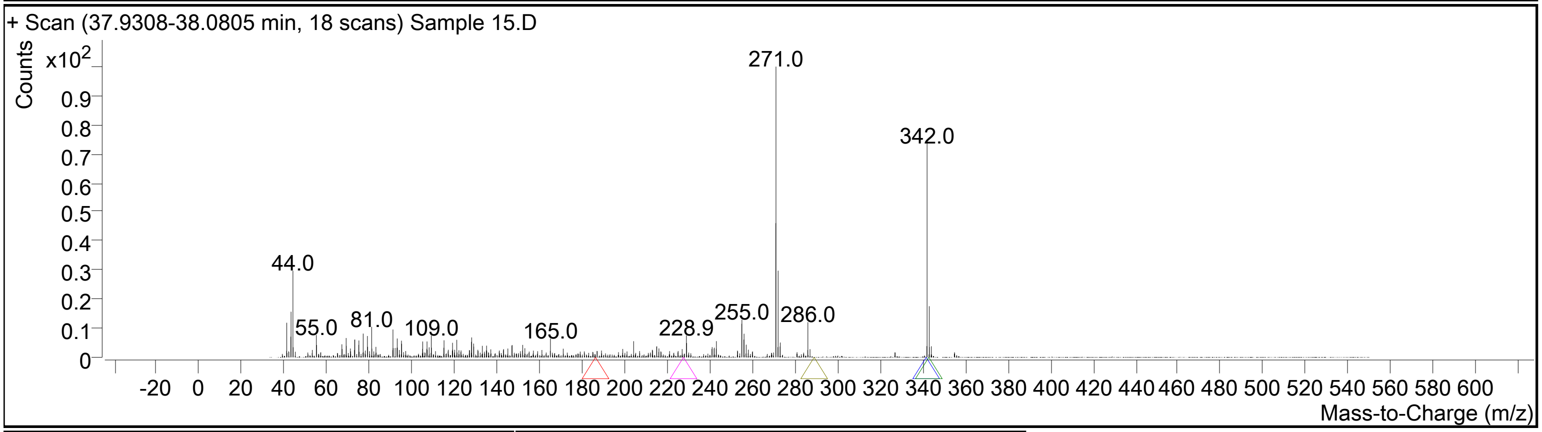

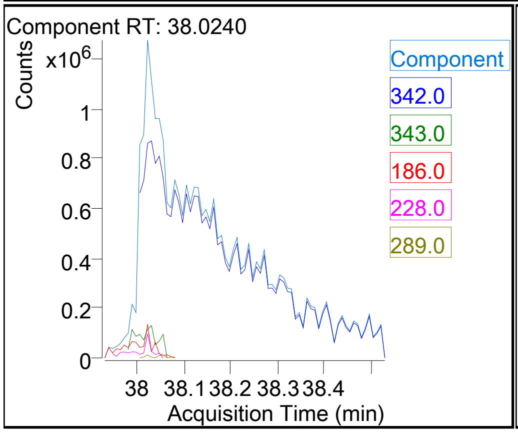

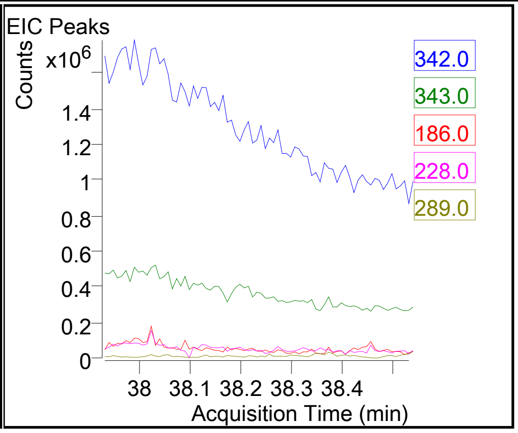

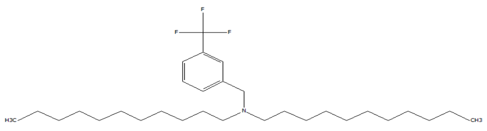

Component RT	Compound Name	Component Area	Match Factor	CAS#	Formula	Estimated Conc.
38.1122	1,4-Dimethyl-8-isopropylidenetricyclo[5.3.0.0(4,10)]decane	75912392.9	78.6	1000140-07-7	C15H24	

Component RT: 38.1122

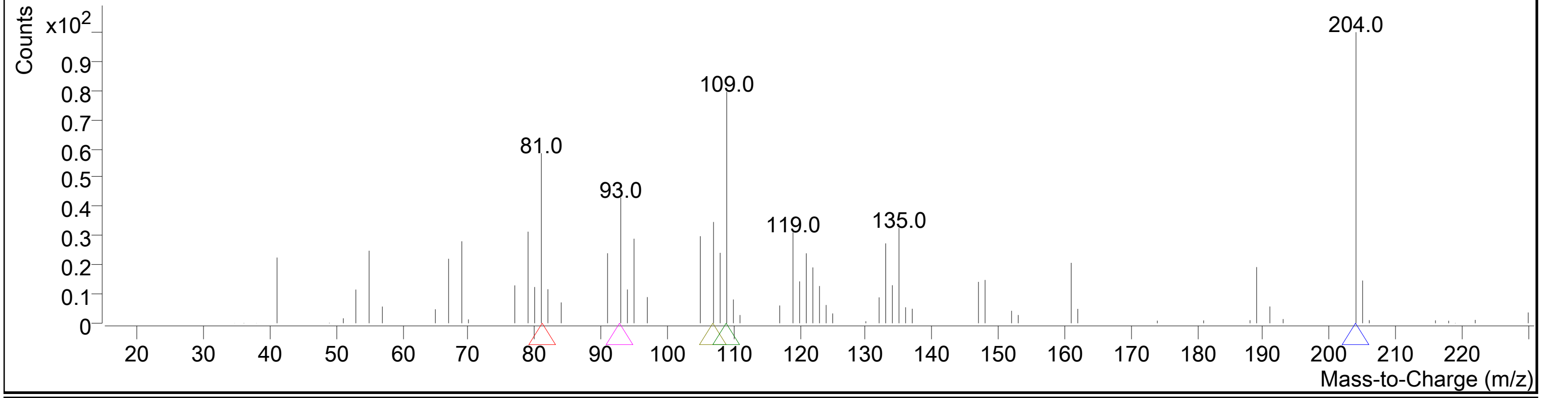

1,4-Dimethyl-8-isopropylidenetricyclo[5.3.0.0(4,10)]decane (NIST17.L)

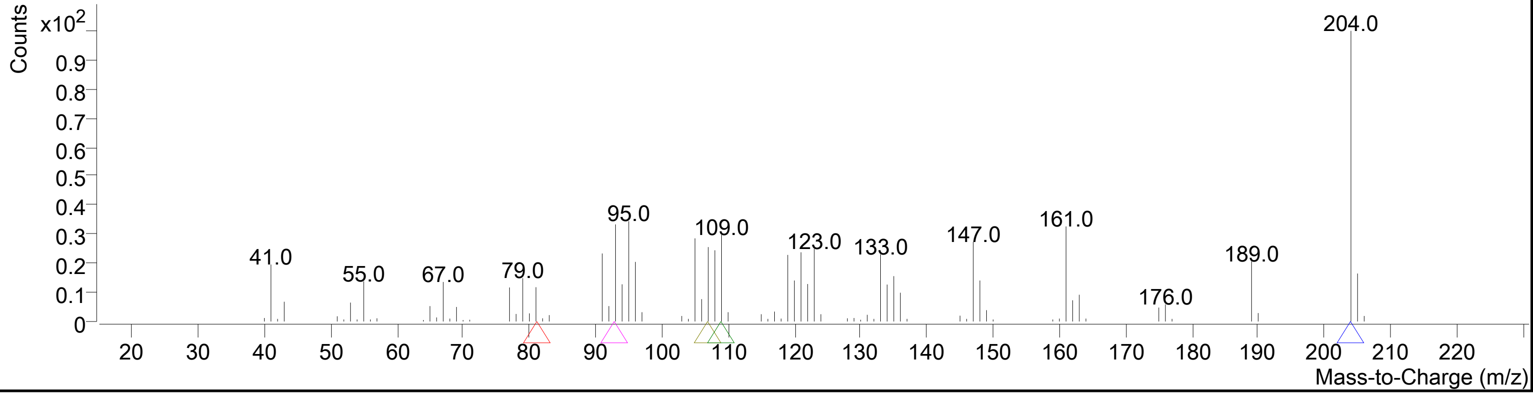

+ Scan (38.0489-38.2385 min, 23 scans) Sample 15.D

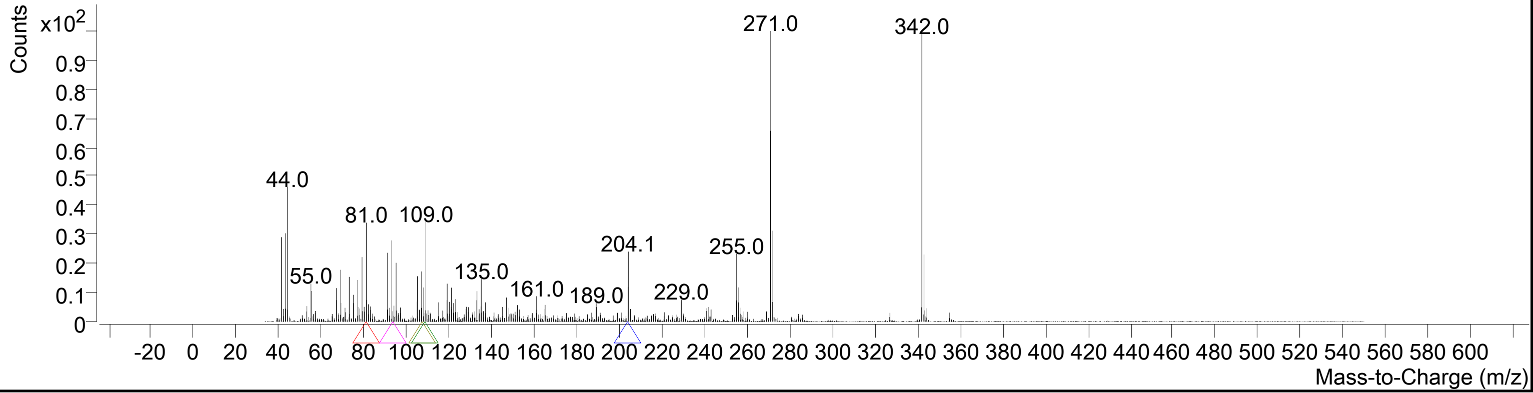

Component RT: 38.1122

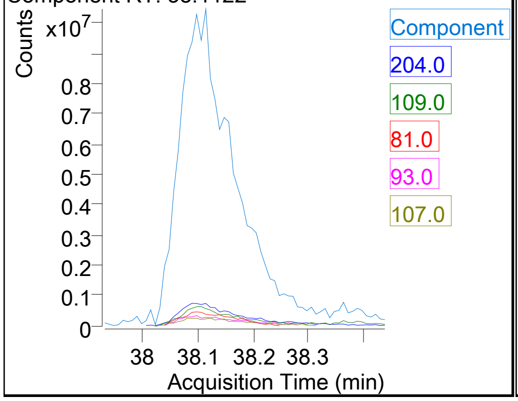

EIC Peaks

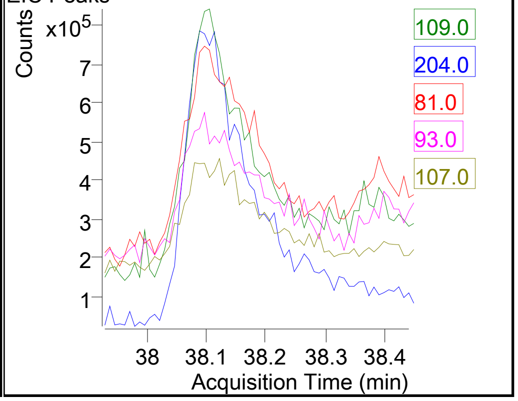

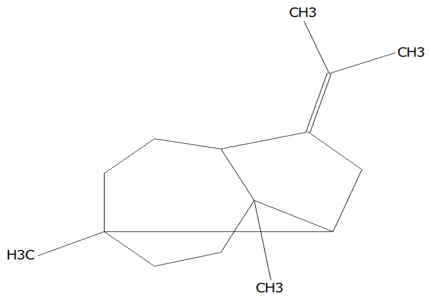

Agilent Technologies

Component RT	Compound Name	Component Area	Match Factor	CAS#	Formula	Estimated Conc.
38.2360	Acetic acid, 2-(1,5-ditert-butyl-3-phenyl-3-piperidyl) ester	8643996.4	66.1	328282-96-8	C21H33NO2	

Component RT: 38.2360

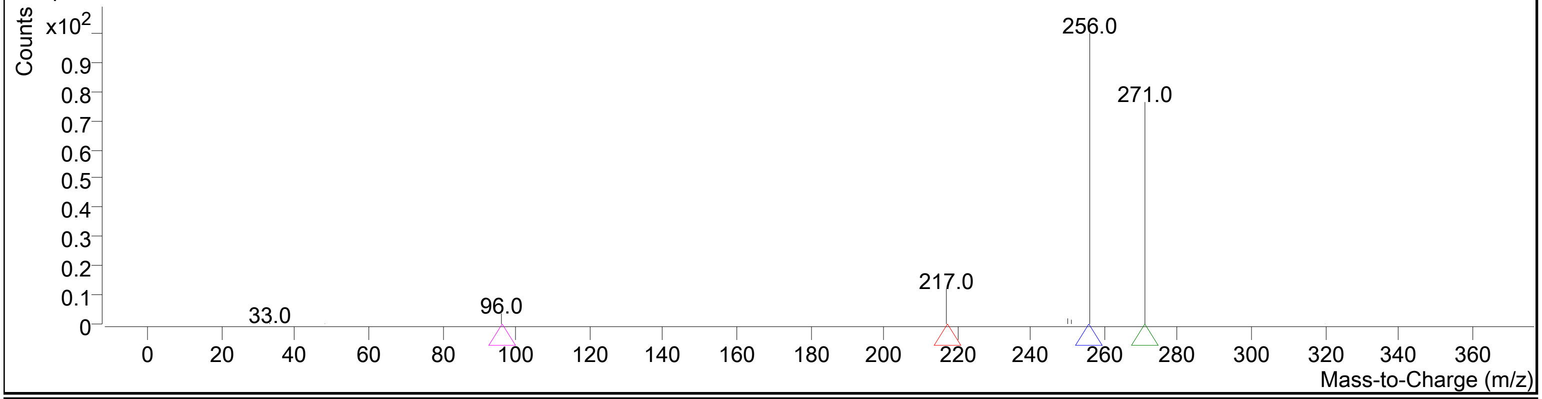

Acetic acid, 2-(1,5-ditert-butyl-3-phenyl-3-piperidyl) ester (NIST17.L)

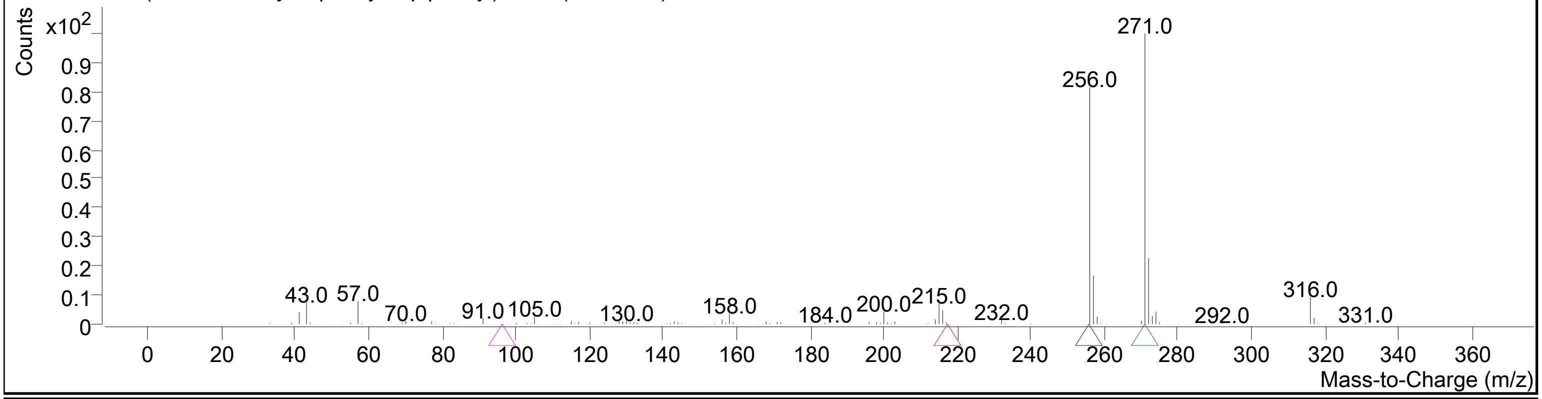

+ Scan (38.2219-38.2551 min, 5 scans) Sample 15.D

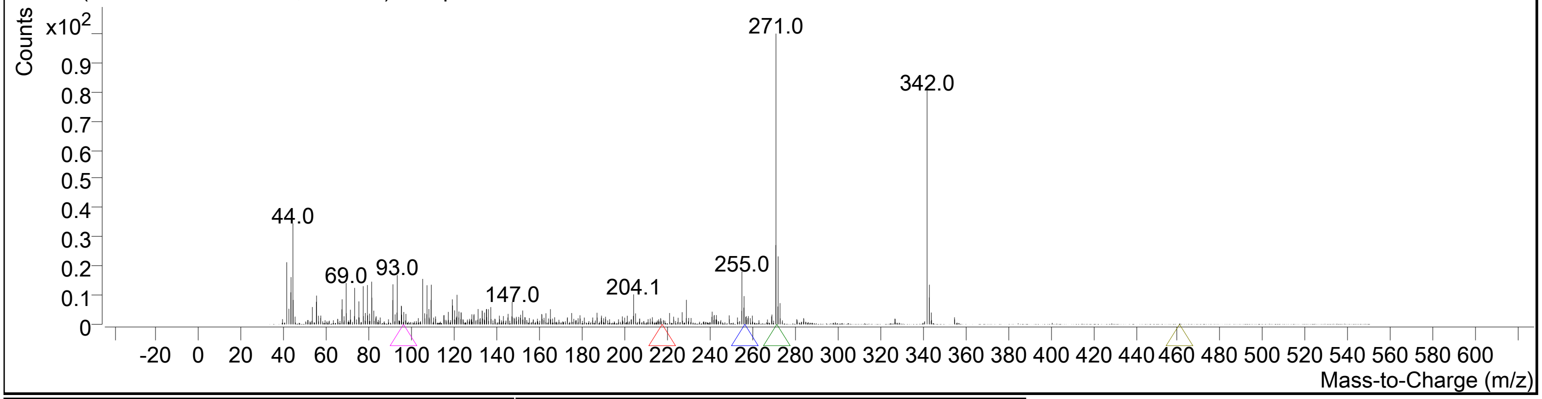

Component RT: 38.2360

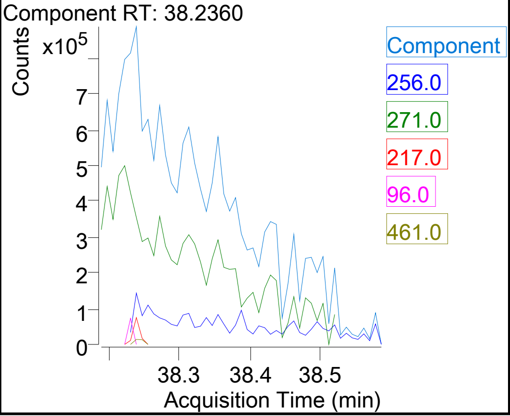

EIC Peaks

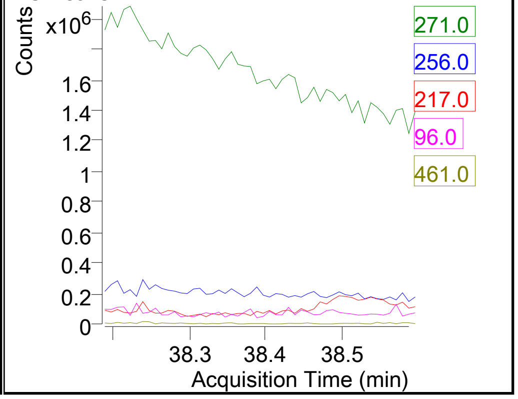

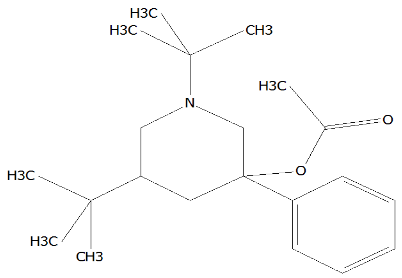

Generated at 12:24 PM on 2/25/2021

Component RT	Compound Name	Component Area	Match Factor	CAS#	Formula	Estimated Conc.
38.4444	1,2-Bis(diethylamino)-1,2-bis(4-cyanophenyl)ethane	1331846.3	66.3	1000193-30-0	C24H30N4	

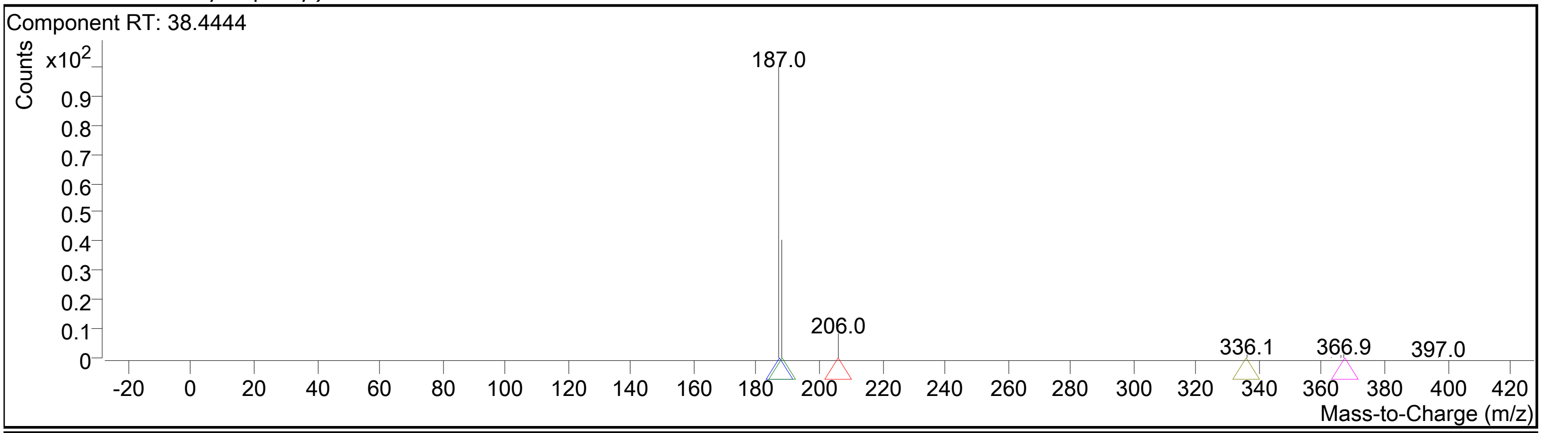

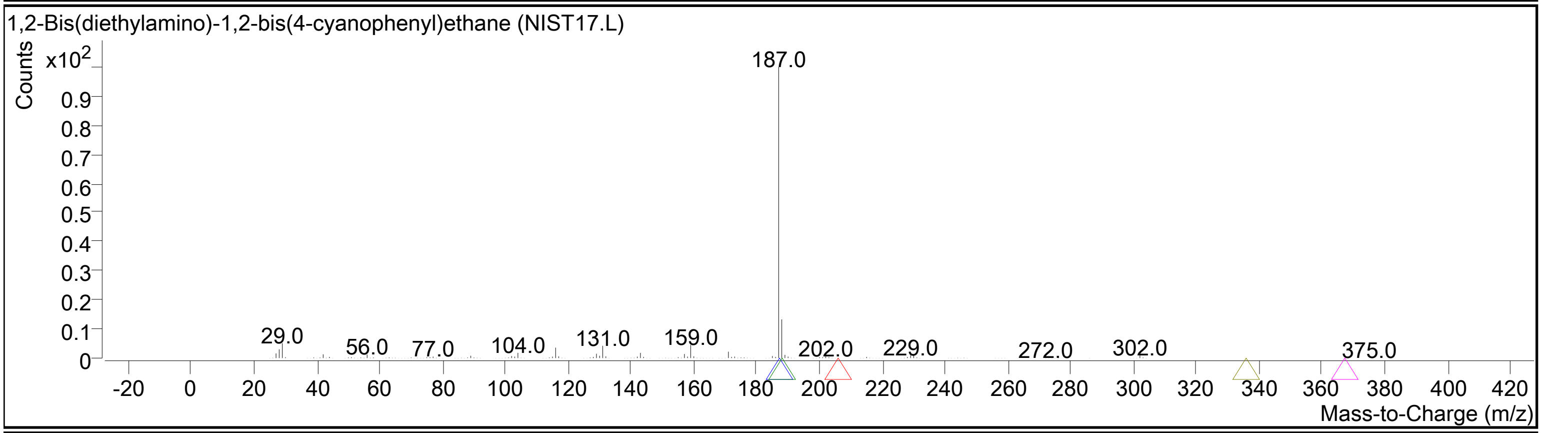

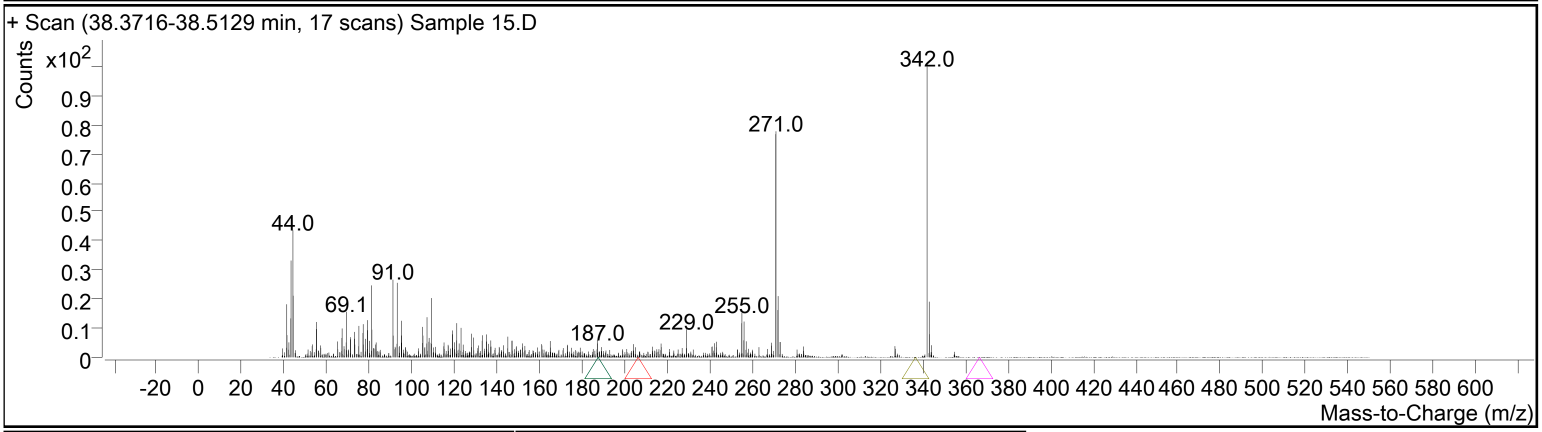

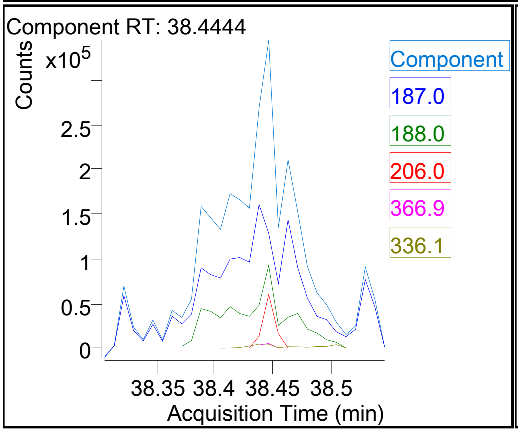

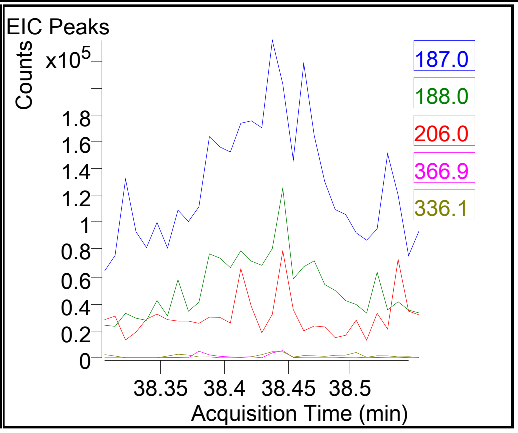

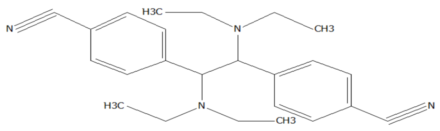

Library Search Results - NonTarget Hits with Details

Component RT	Compound Name	Component Area	Match Factor	CAS#	Formula	Estimated Conc.
38.7934	1,3,6,10-Cyclotetradecatetraene, 3,7,11-trimethyl-14-(1-methylethyl)-, [S-(E,Z,E,E)]-	68877614.6	77.3	1898-13-1	C20H32	

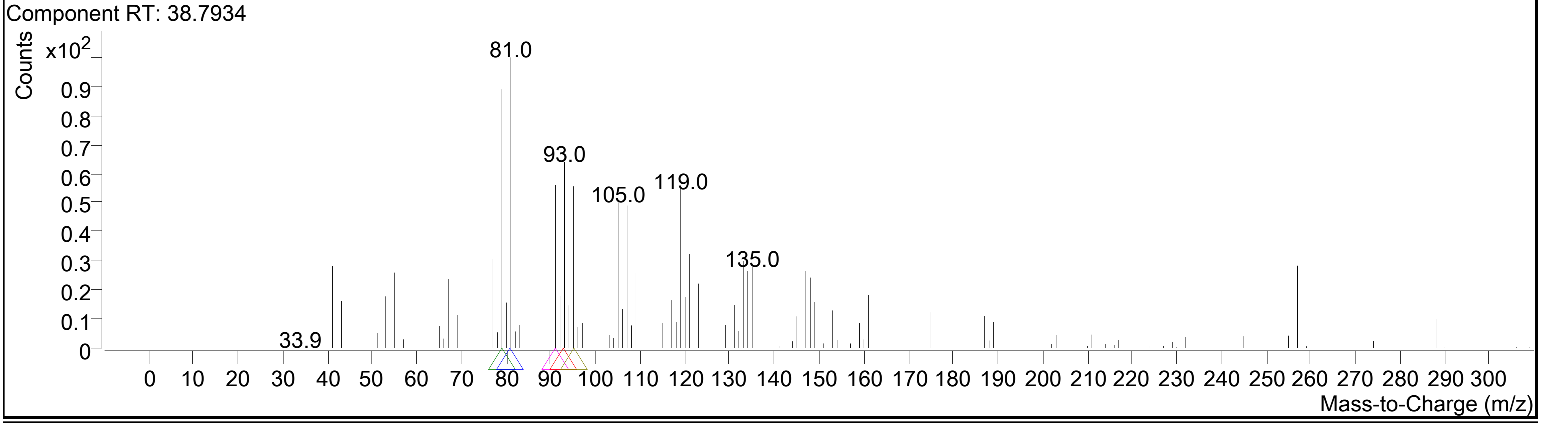

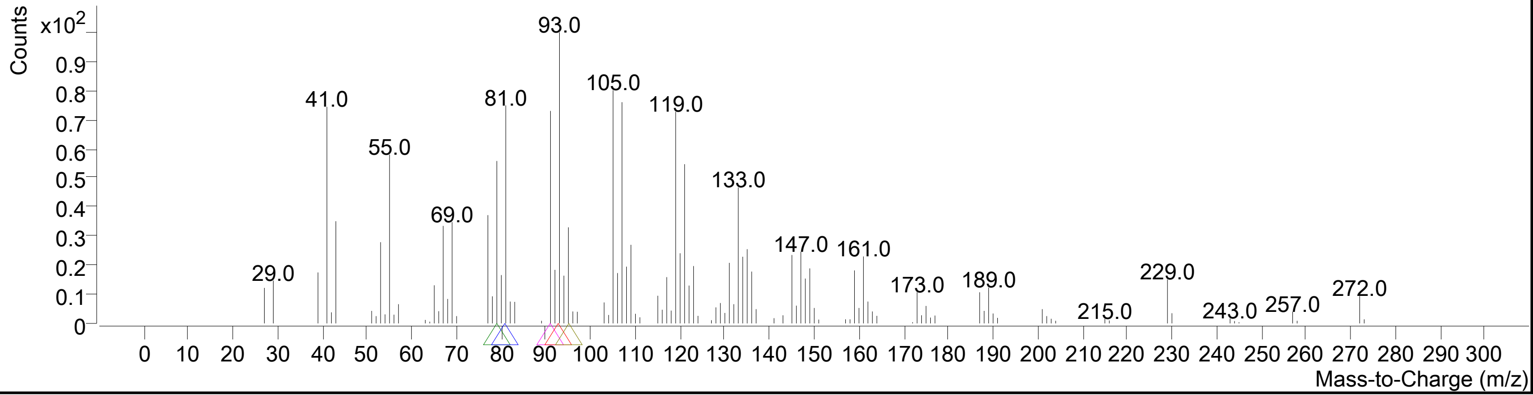

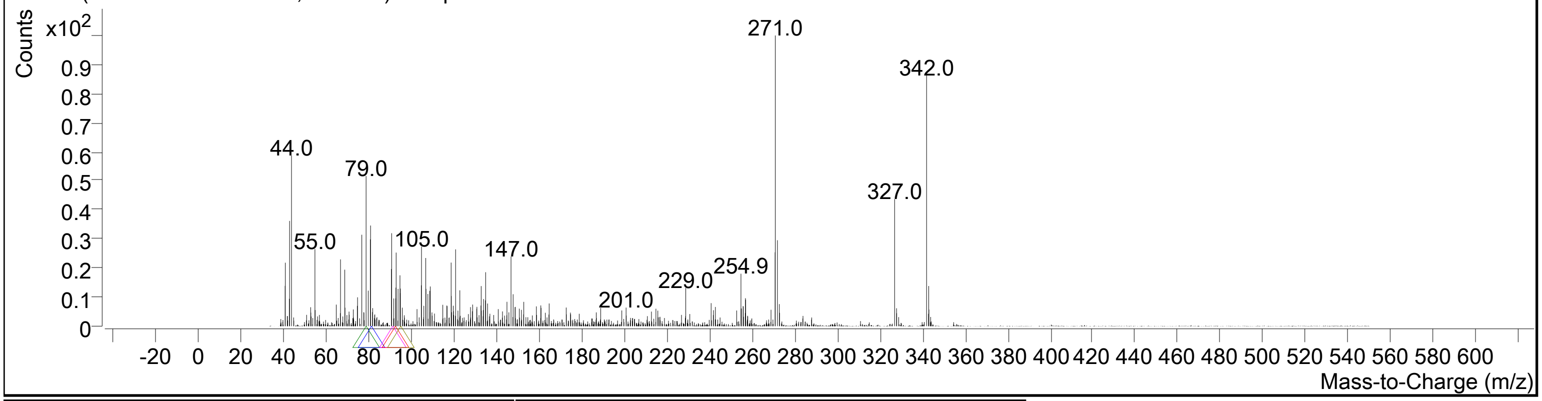

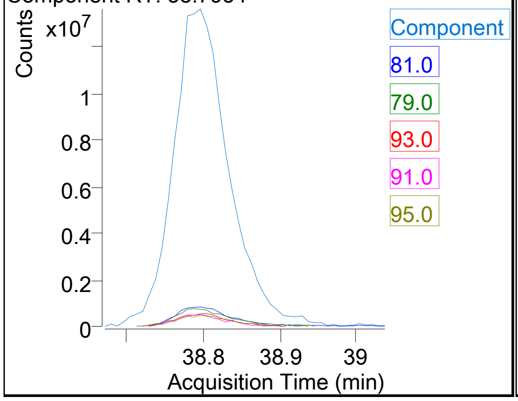

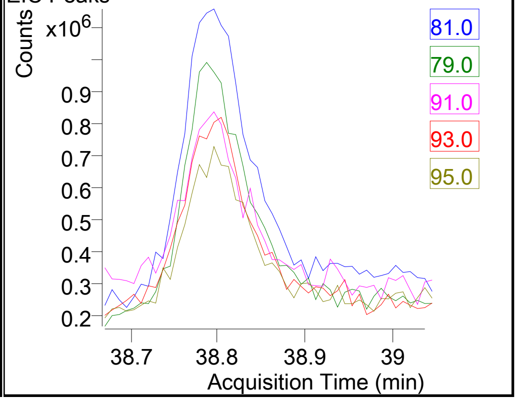

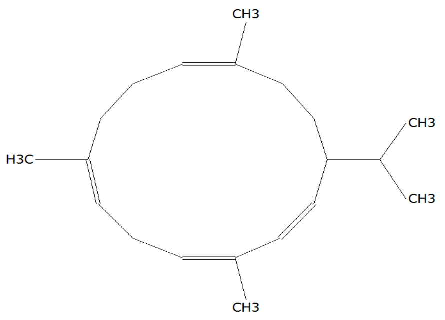

Component RT	Compound Name	Component Area	Match Factor	CAS#	Formula	Estimated Conc.
39.1906	Ferruginol	784231253.5	92.6	514-62-5	C20H30O	

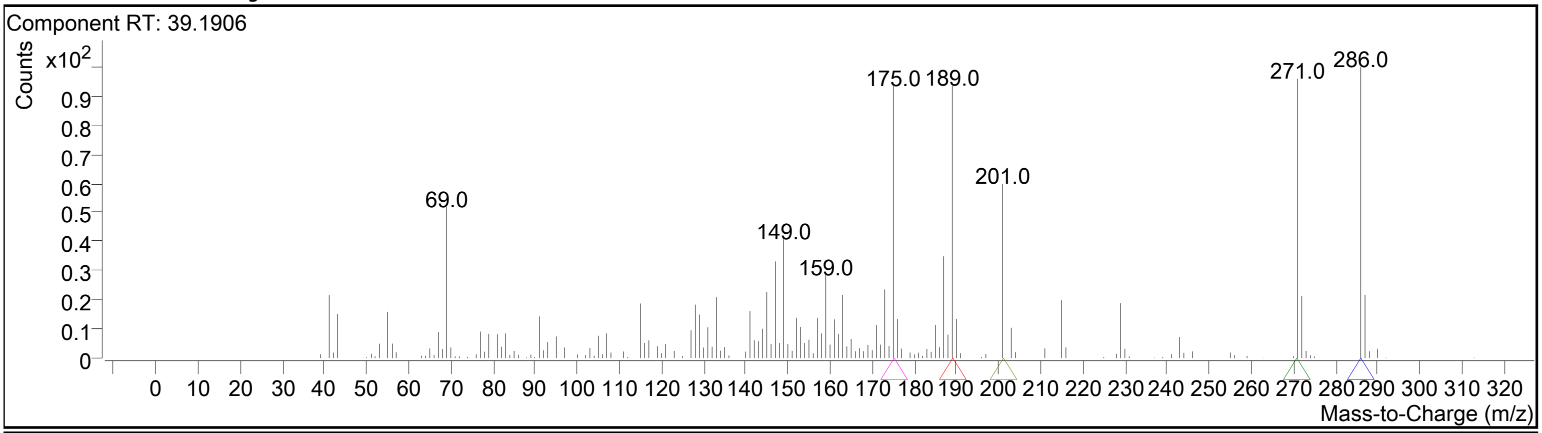

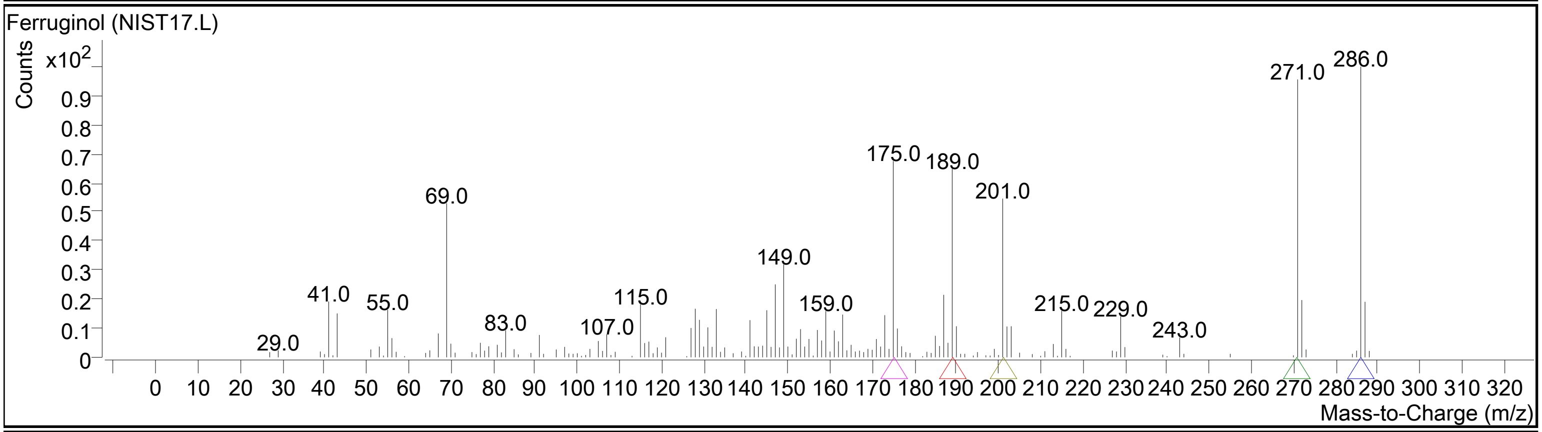

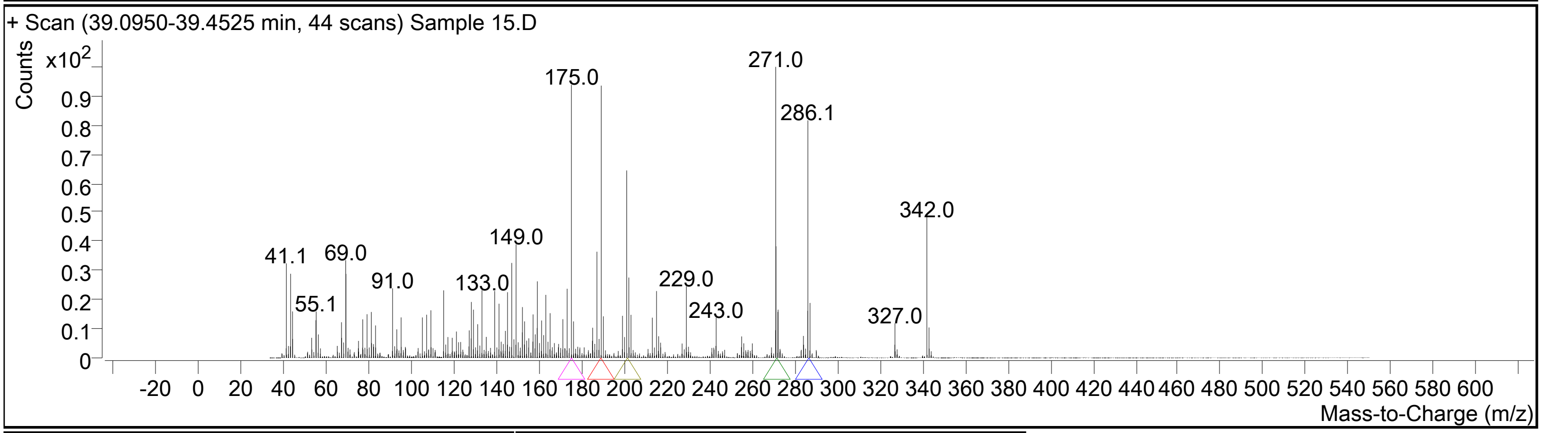

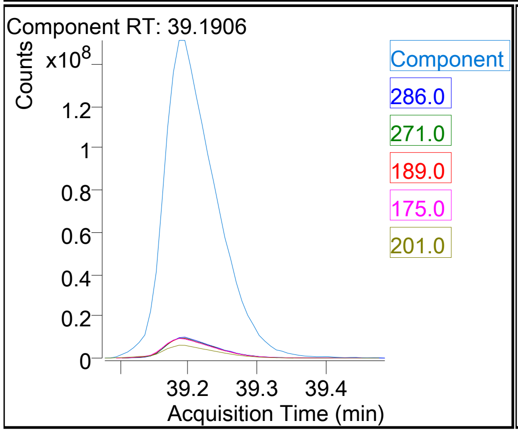

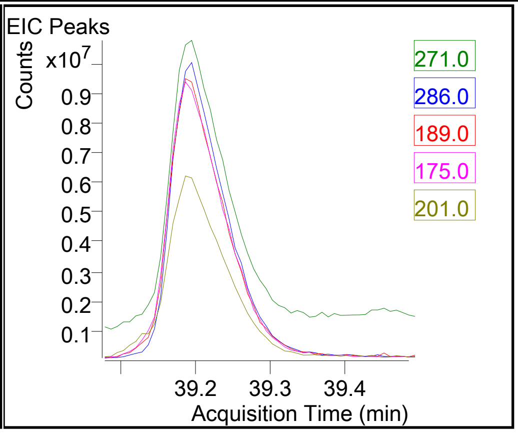

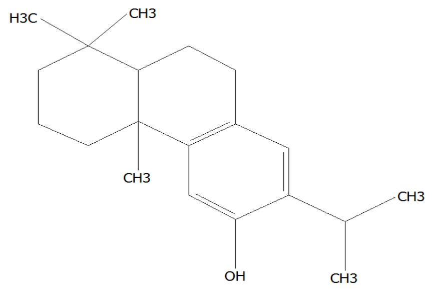

Component RT	Compound Name	Component Area	Match Factor	CAS#	Formula	Estimated Conc.
39.5511	4-Acetylphenyl 5-acetyl-2-methoxyphenyl ether	50766154.7	65.8	7251-24-3	C17H16O4	

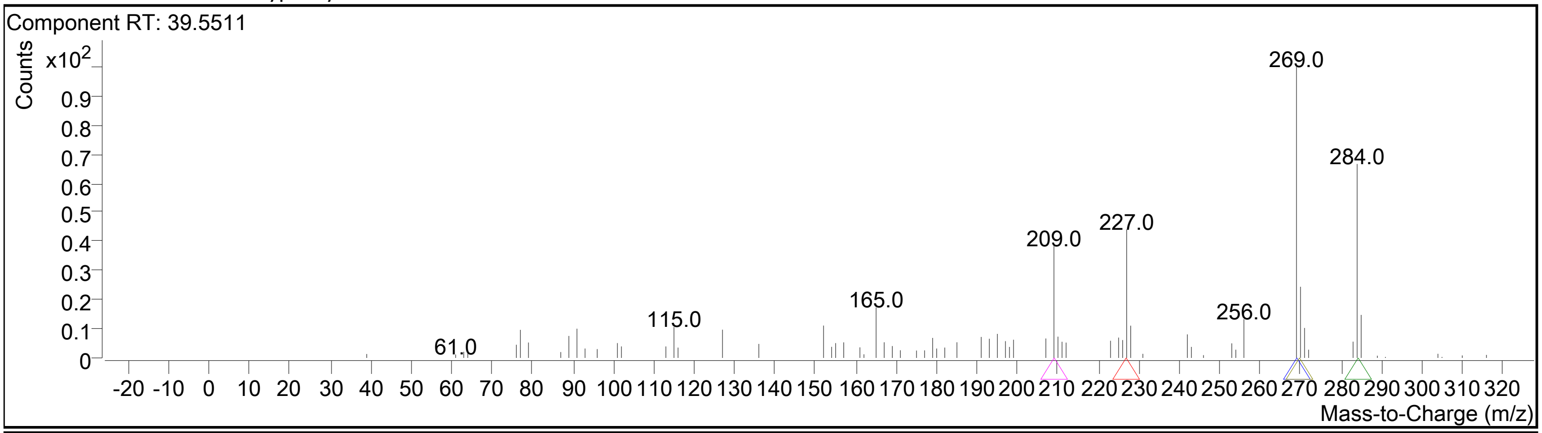

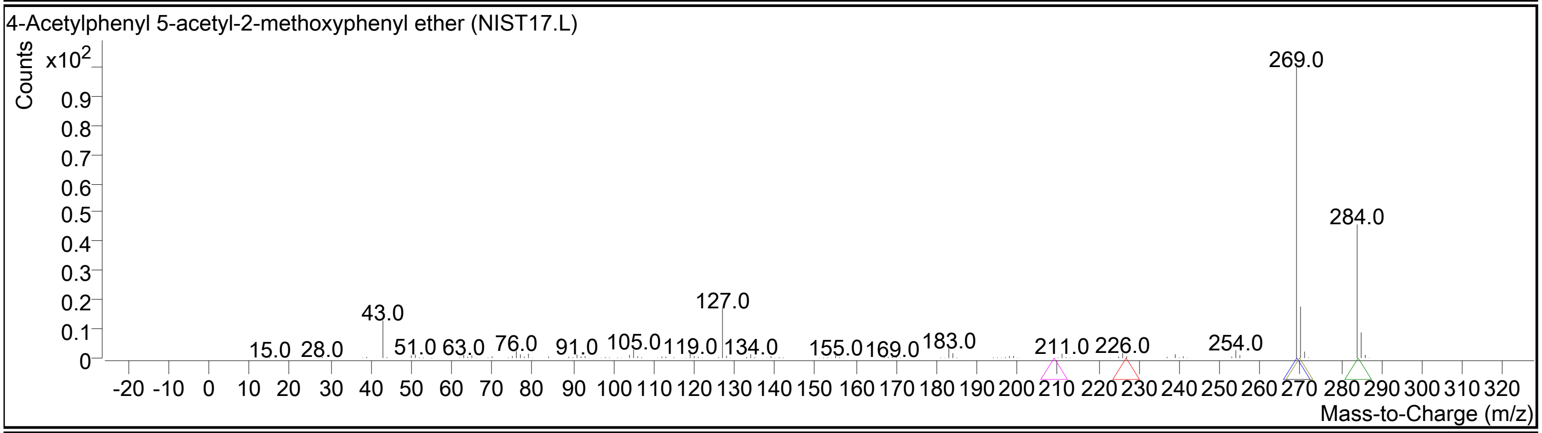

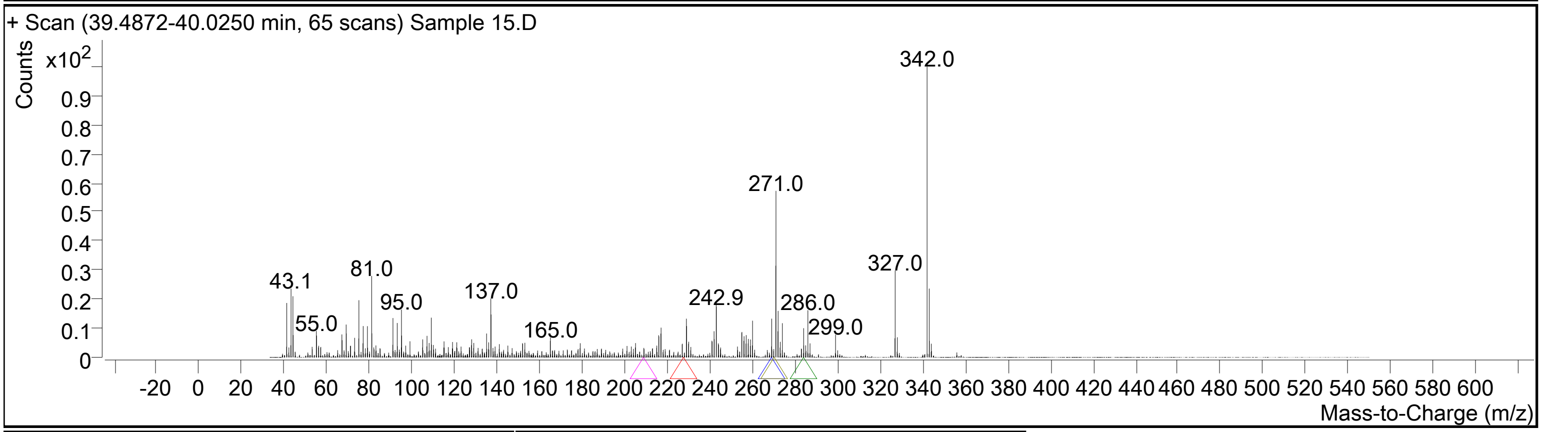

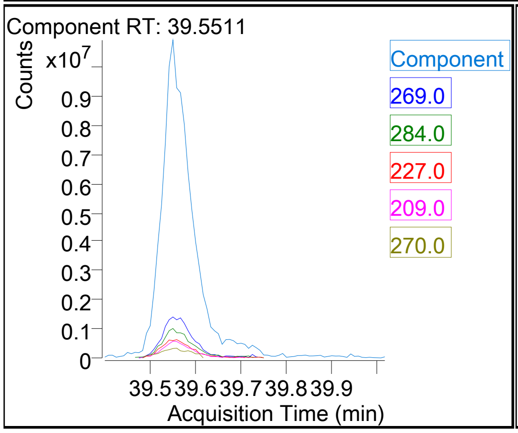

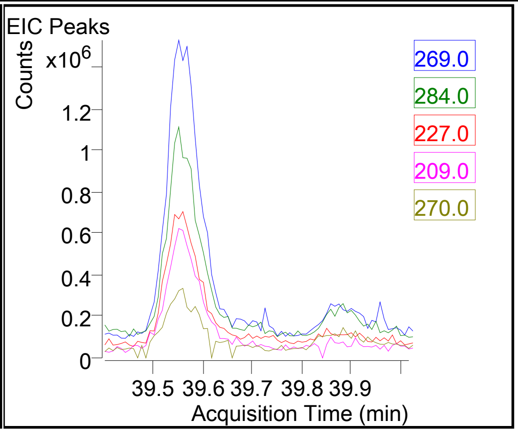

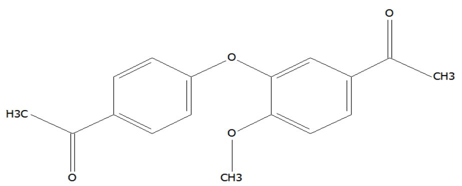

Generated at 12:24 PM on 2/25/2021

Agilent Technologies

Component RT	Compound Name	Component Area	Match Factor	CAS#	Formula	Estimated Conc.
39.6357	Trifluoroacetic acid, 2-methylpropyl ester	12150521.2	75.4	17355-83-8	C6H9F3O2	

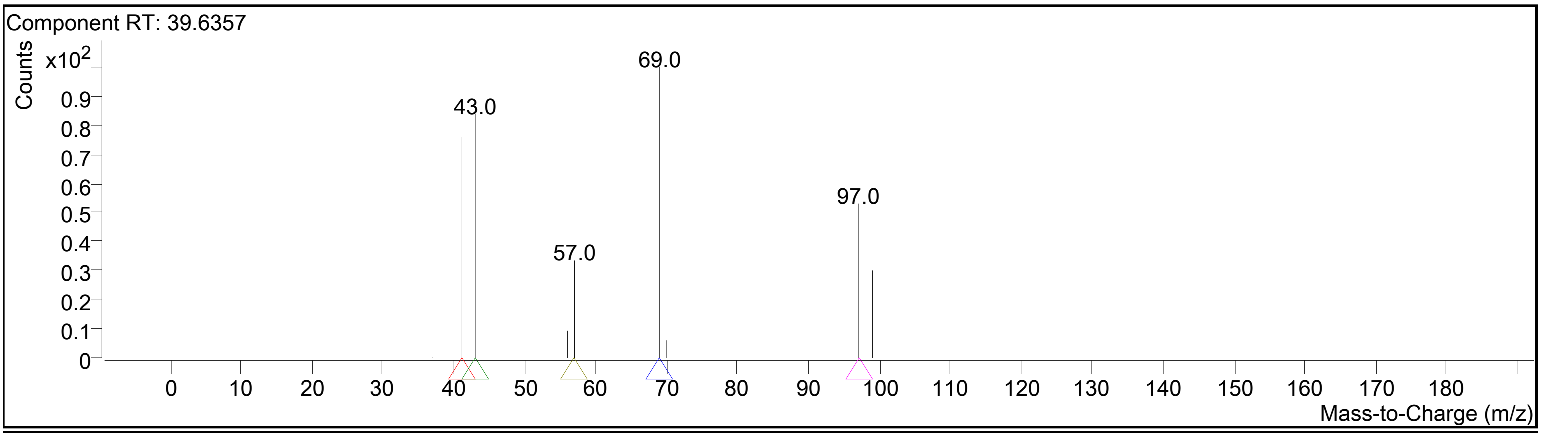

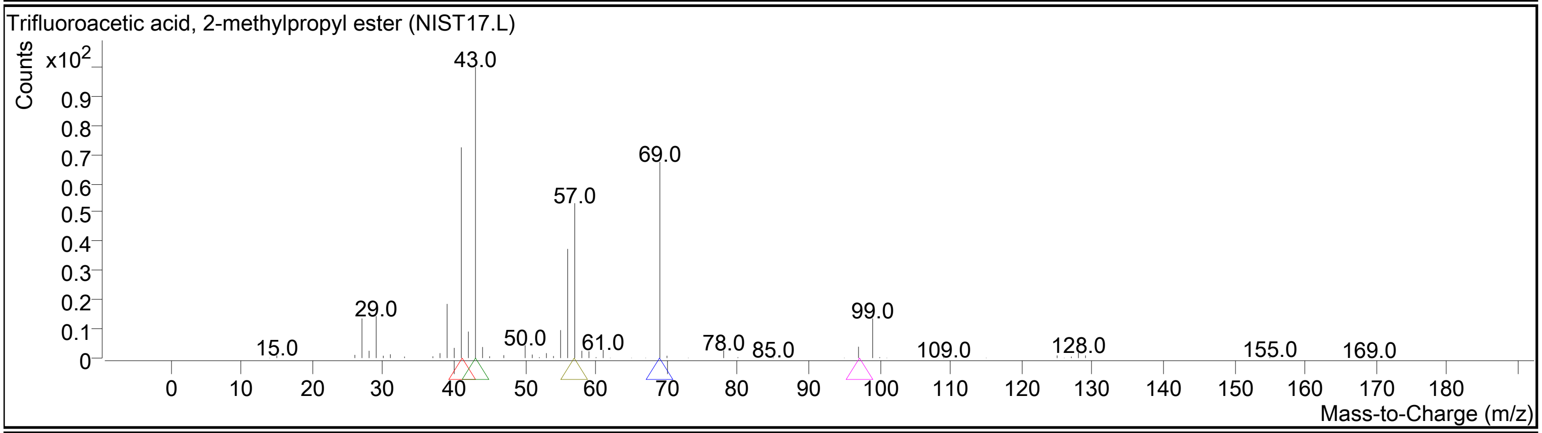

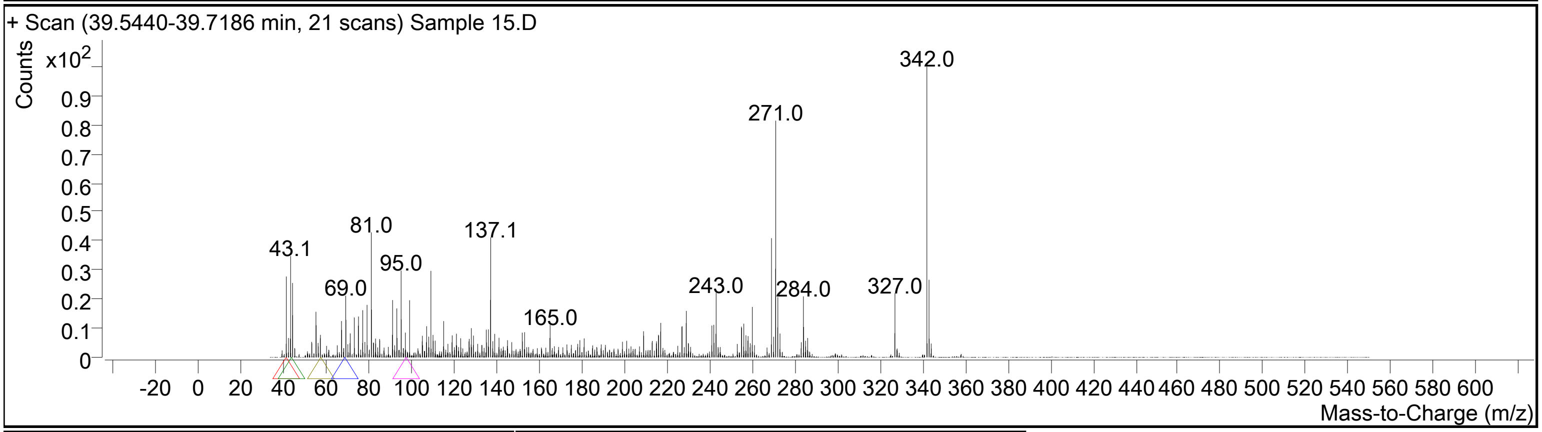

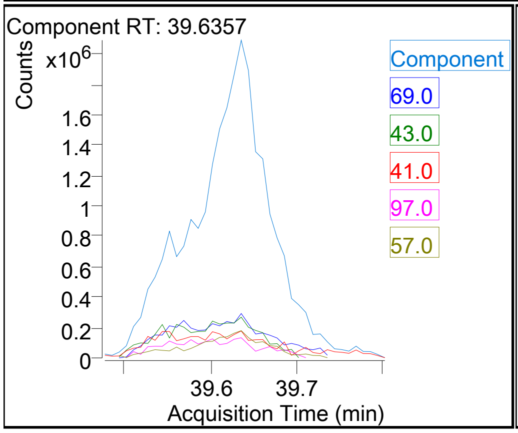

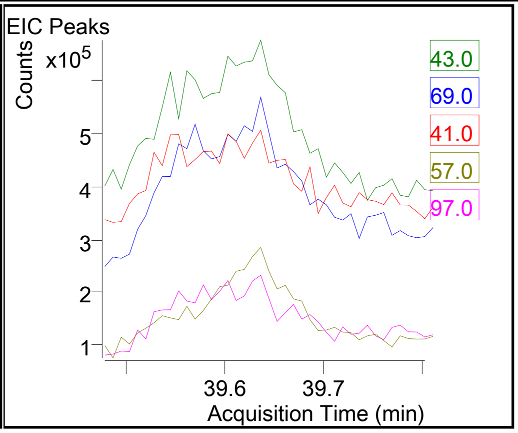

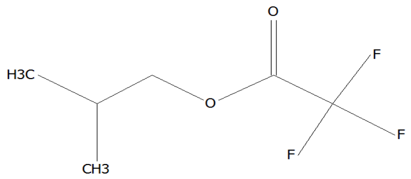

Component RT	Compound Name	Component Area	Match Factor	CAS#	Formula	Estimated Conc.
39.8748	4,5,6,7-Tetrahydroxy-1,8,8,9-tetramethyl-8,9-dihydrophenaleno[1,2-b]furan-3-one	159889981.2	65.4	2582-86-7	C19H18O6	

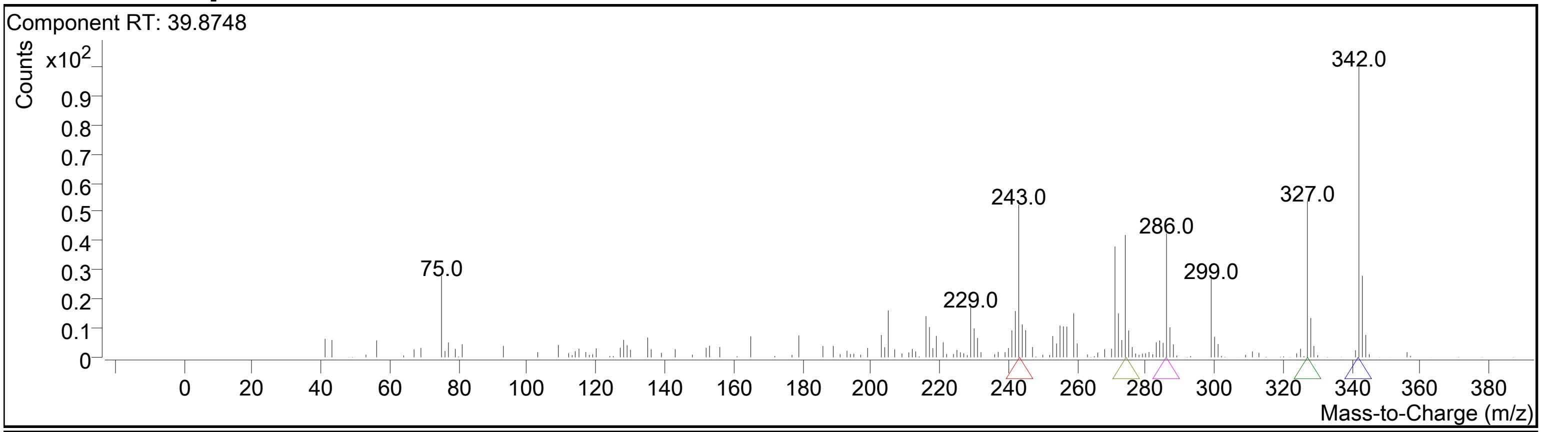

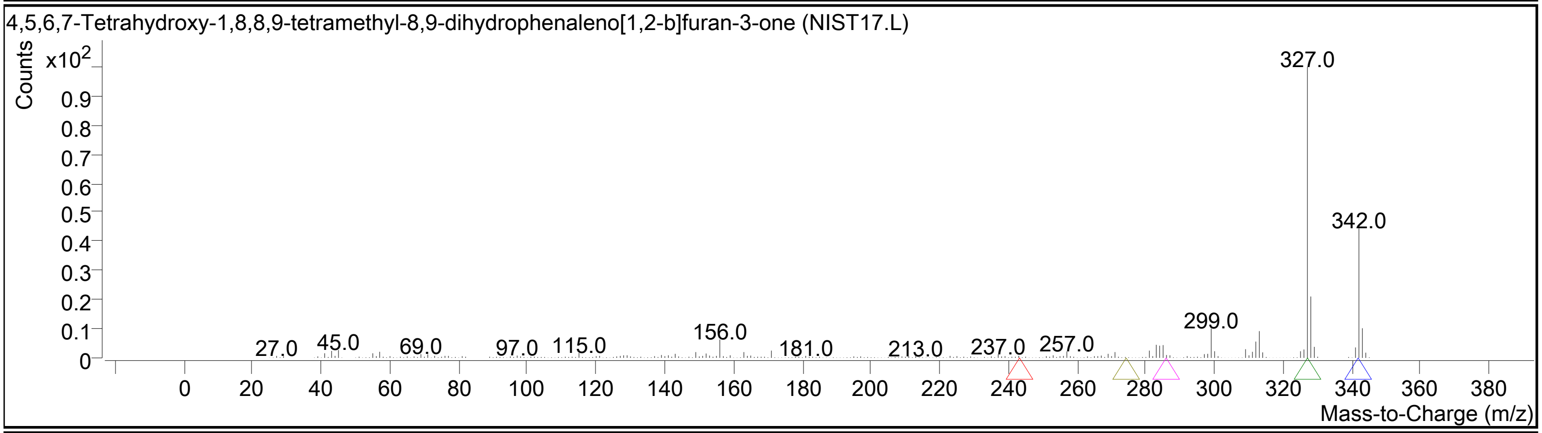

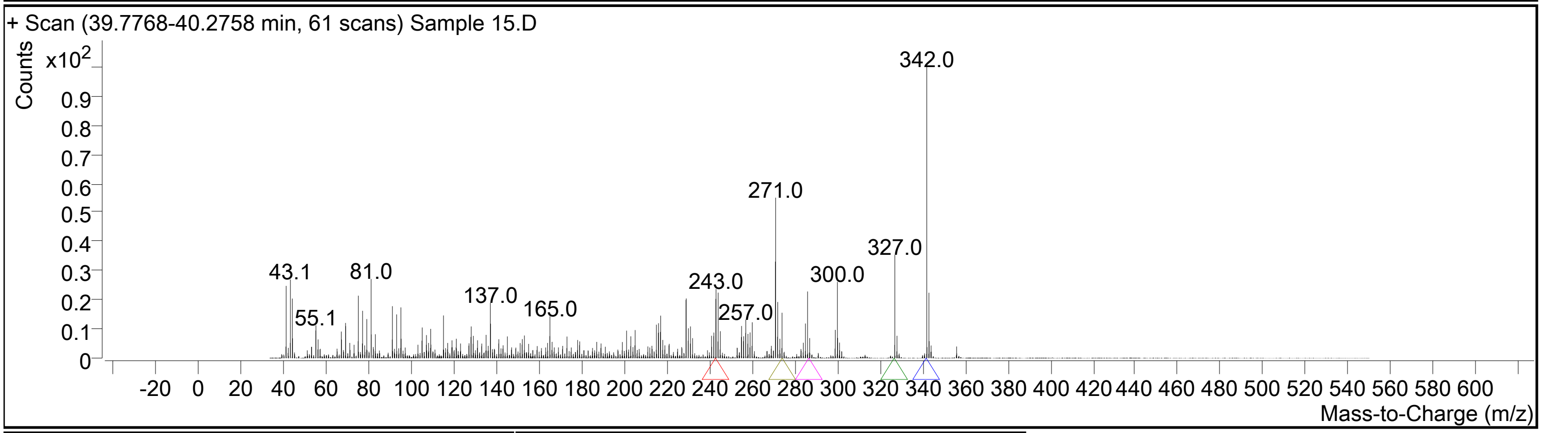

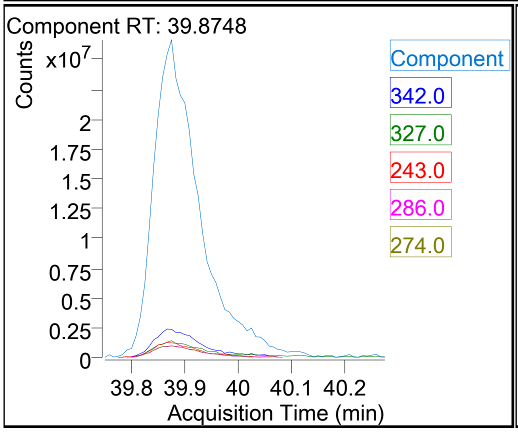

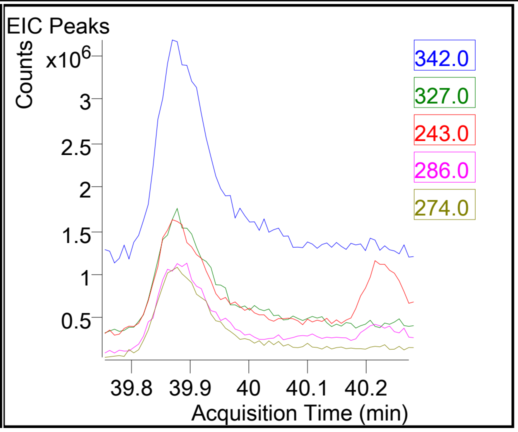

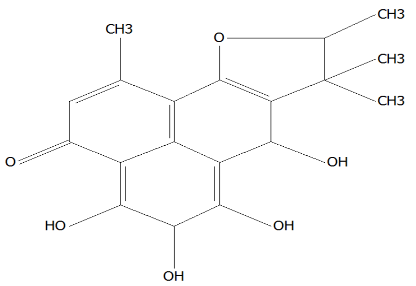

Component RT	Compound Name	Component Area	Match Factor	CAS#	Formula	Estimated Conc.
40.0515	4-Phenoxy-2-phenyl-1-naphthalenol	628144.6	66.9	253801-45-5	C22H16O2	

Component RT: 40.0515

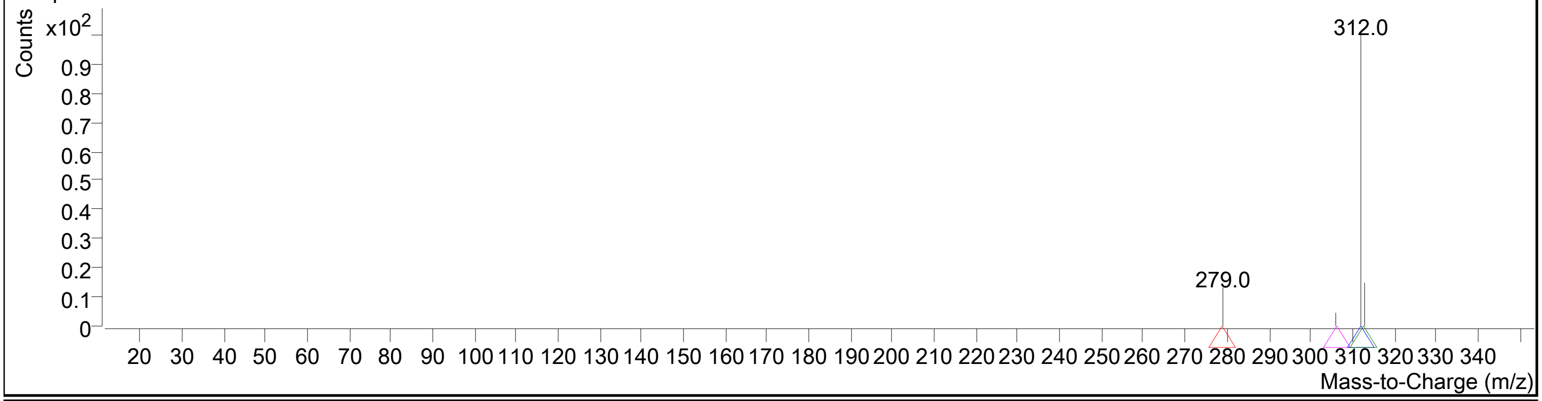

4-Phenoxy-2-phenyl-1-naphthalenol (NIST17.L)

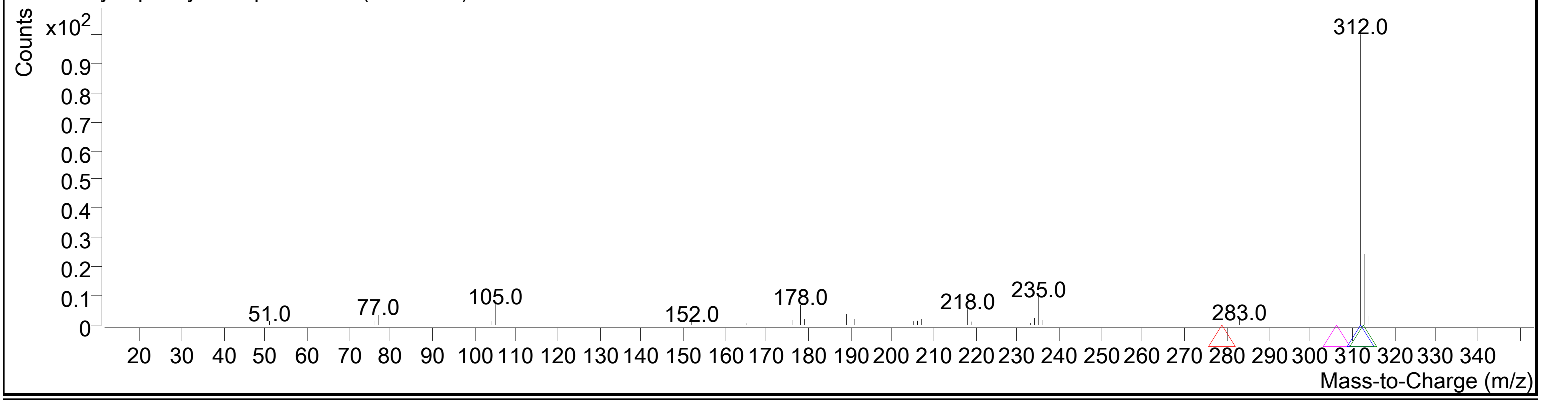

+ Scan (40.0180-40.0679 min, 6 scans) Sample 15.D

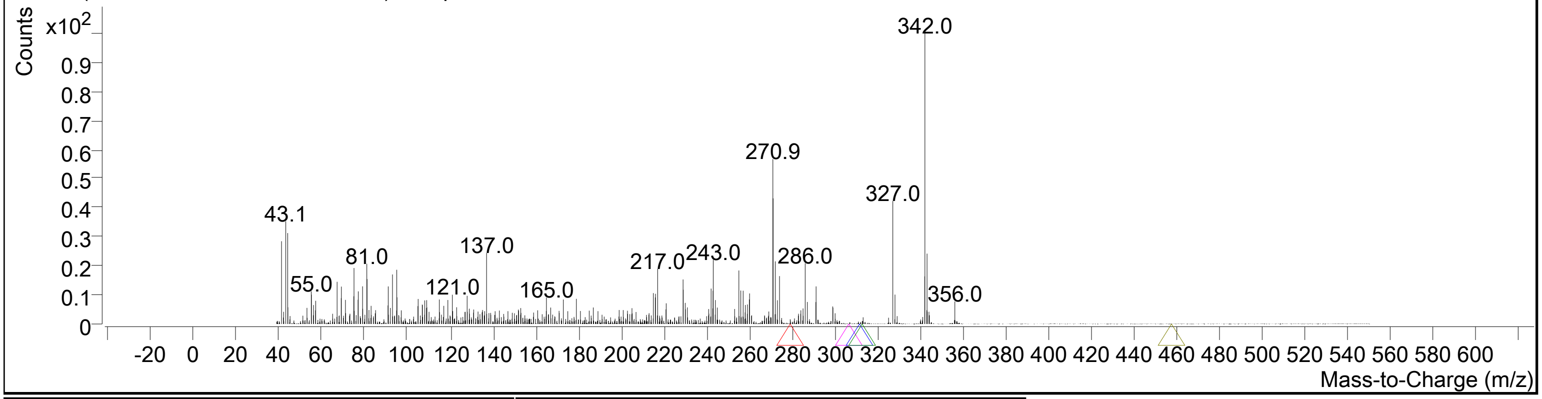

Component RT: 40.0515

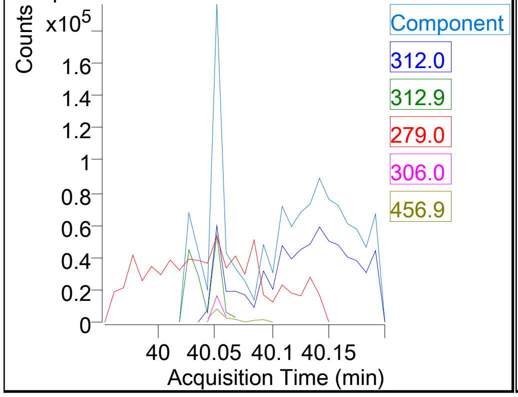

EIC Peaks

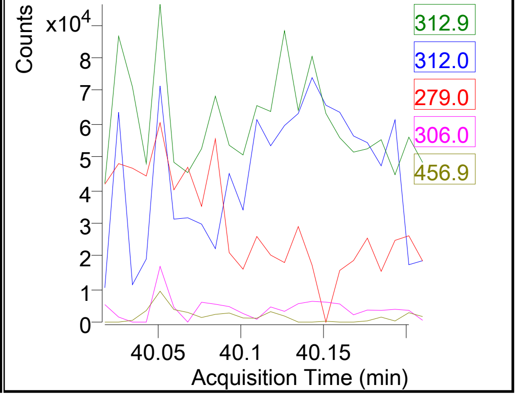

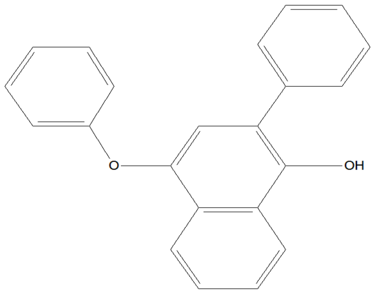

Agilent Technologies

Component RT	Compound Name	Component Area	Match Factor	CAS#	Formula	Estimated Conc.
40.1437	6,8-Dimethyl-5-oxo-2,3,5,8-tetrahydroimidazo[1,2-a]pyrimidine	21959557.6	66.0	26955-15-7	C8H11N3O	

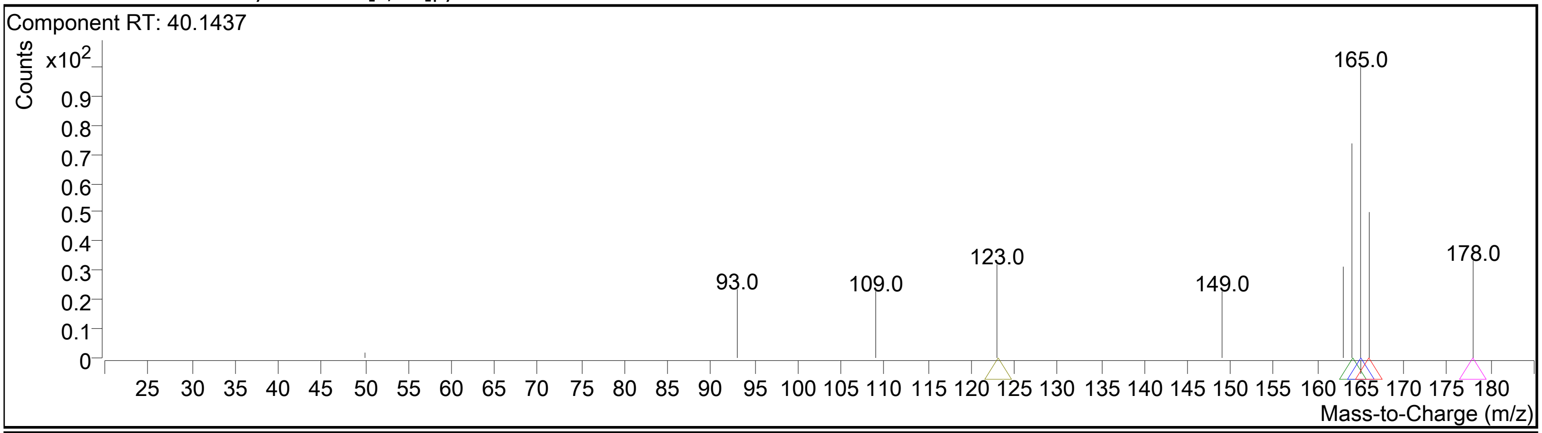

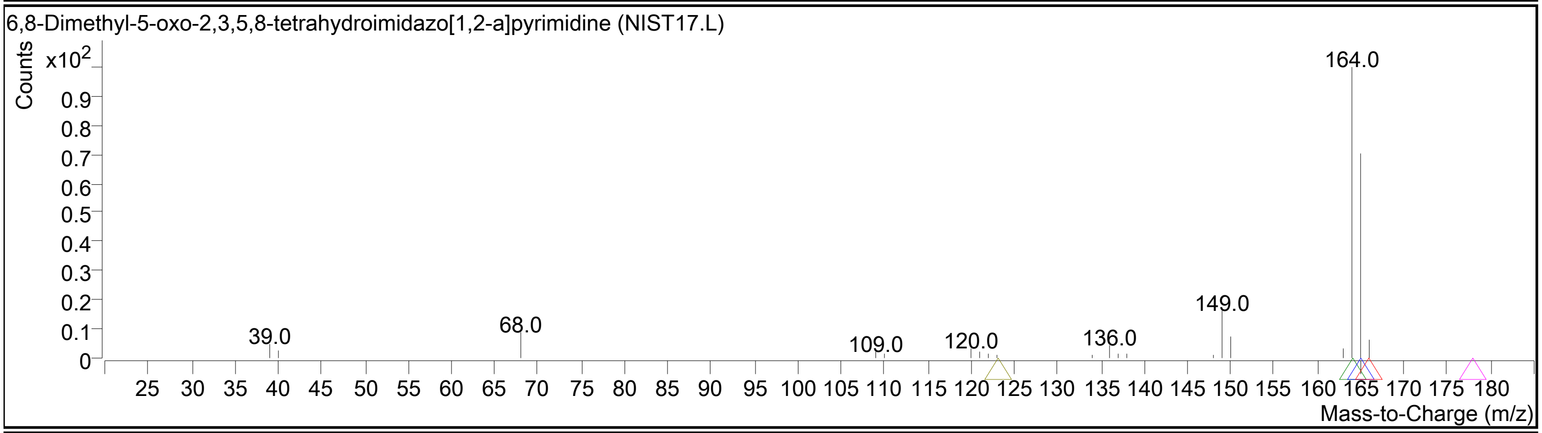

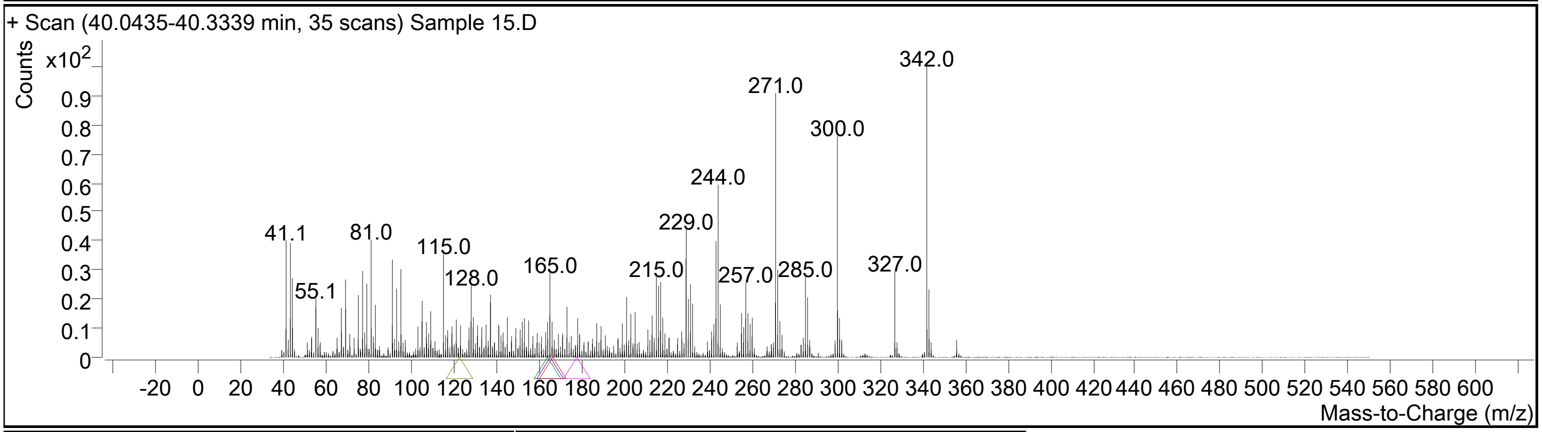

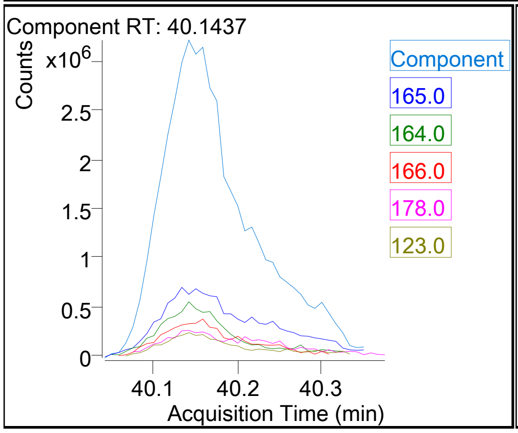

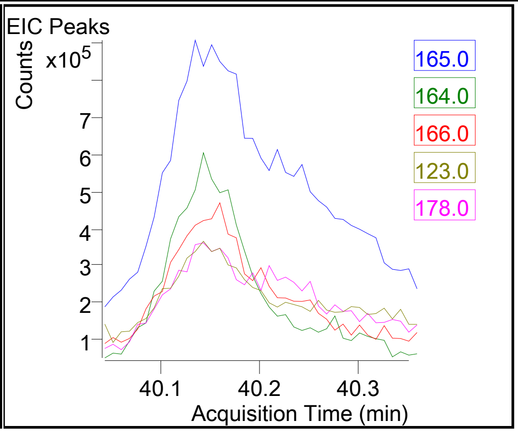

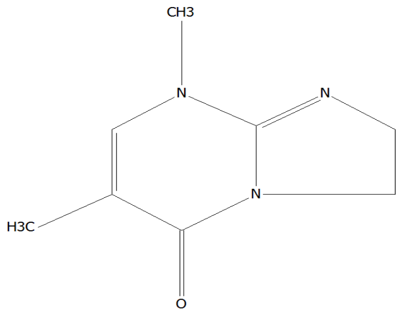

Generated at 12:24 PM on 2/25/2021

Library Search Results - NonTarget Hits with Details

Component RT	Compound Name	Component Area	Match Factor	CAS#	Formula	Estimated Conc.
40.2330	12-O-Methylcarnosol	241176231.5	77.9	85514-27-8	C21H28O4	

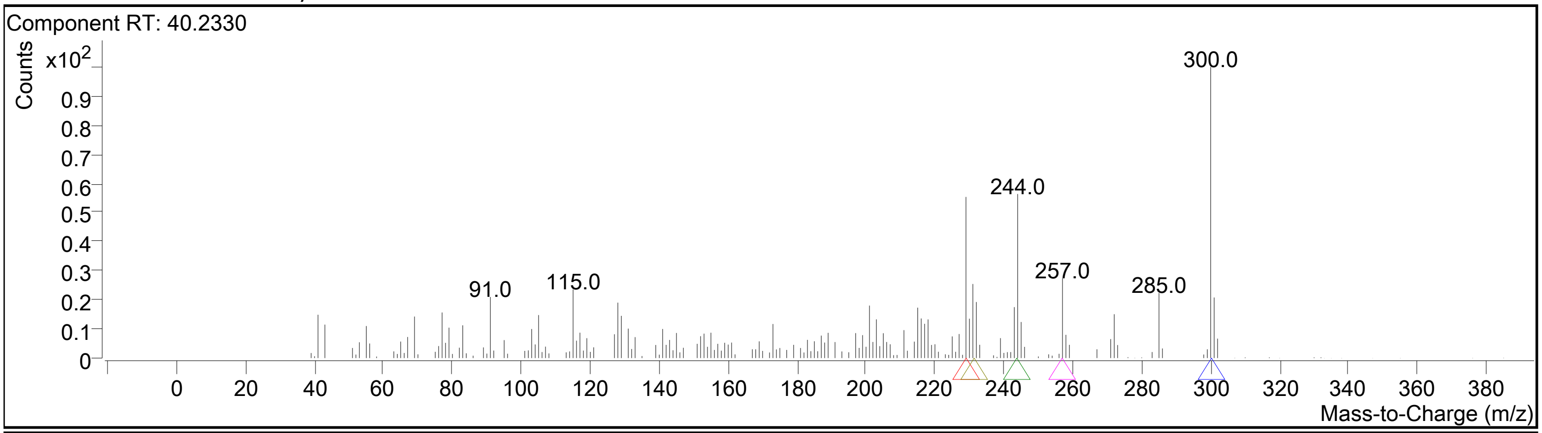

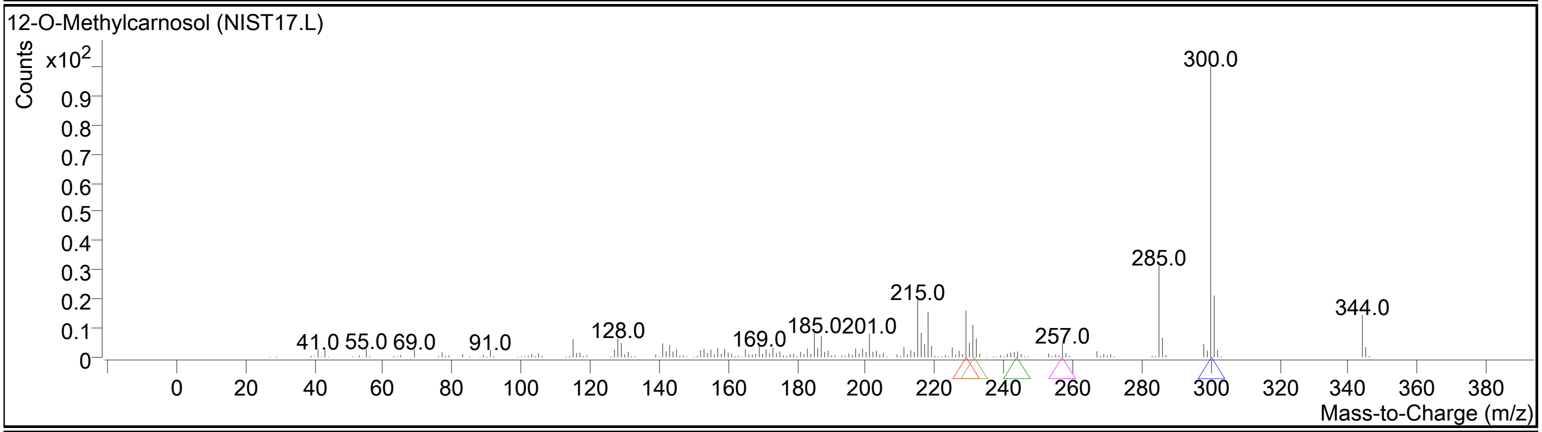

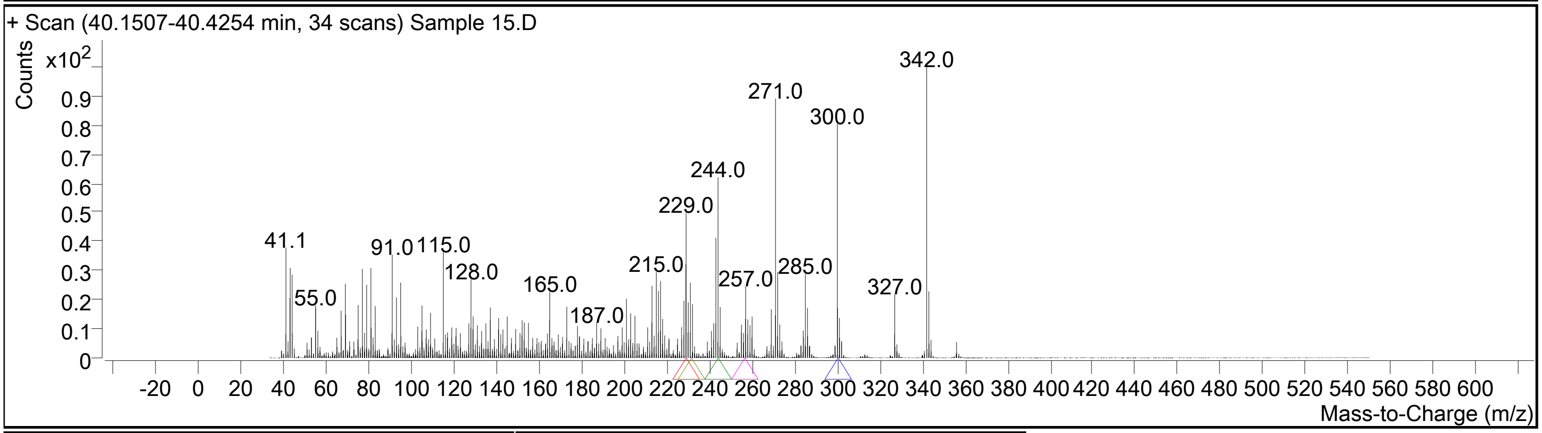

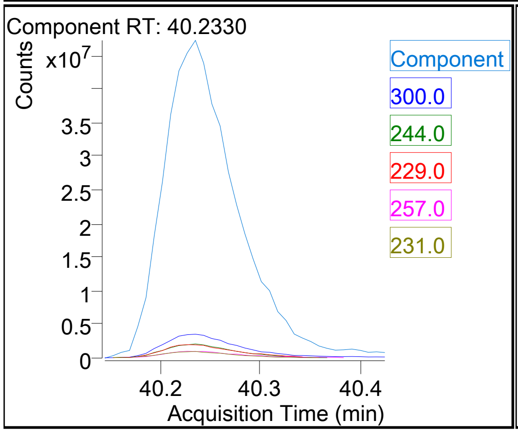

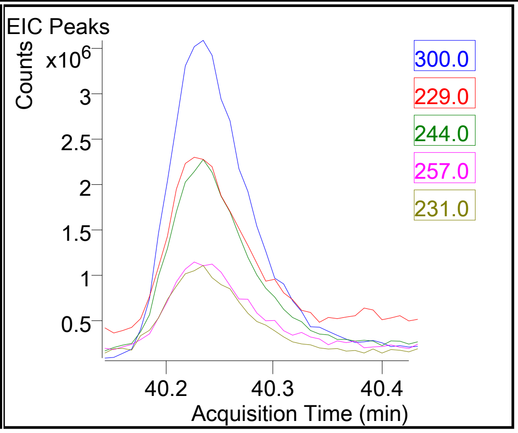

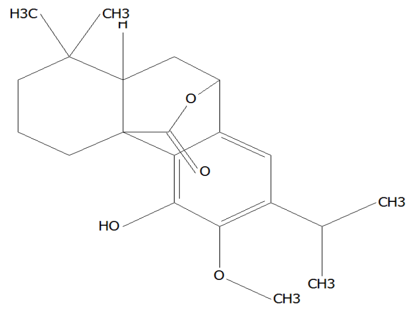

Library Search Results - NonTarget Hits with Details

Component RT	Compound Name	Component Area	Match Factor	CAS#	Formula	Estimated Conc.
40.3933	(tert-Butyl)(4,6-diimidazol-1-yl-[1,3,5]triazin-2-yl)amine	10323728.5	69.8	1000304-62-9	C13H16N8	

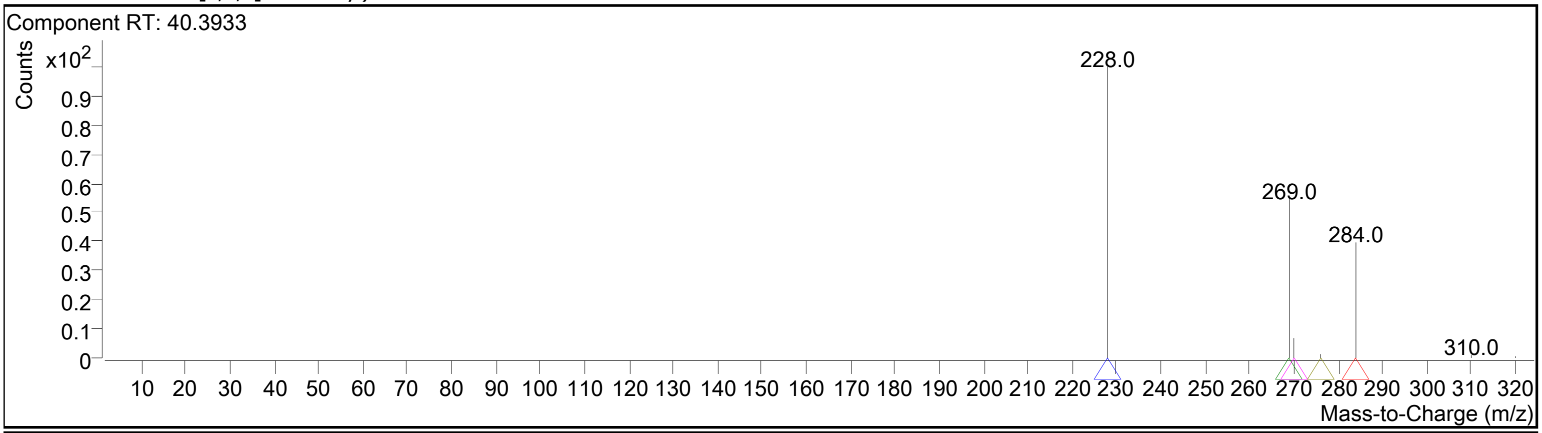

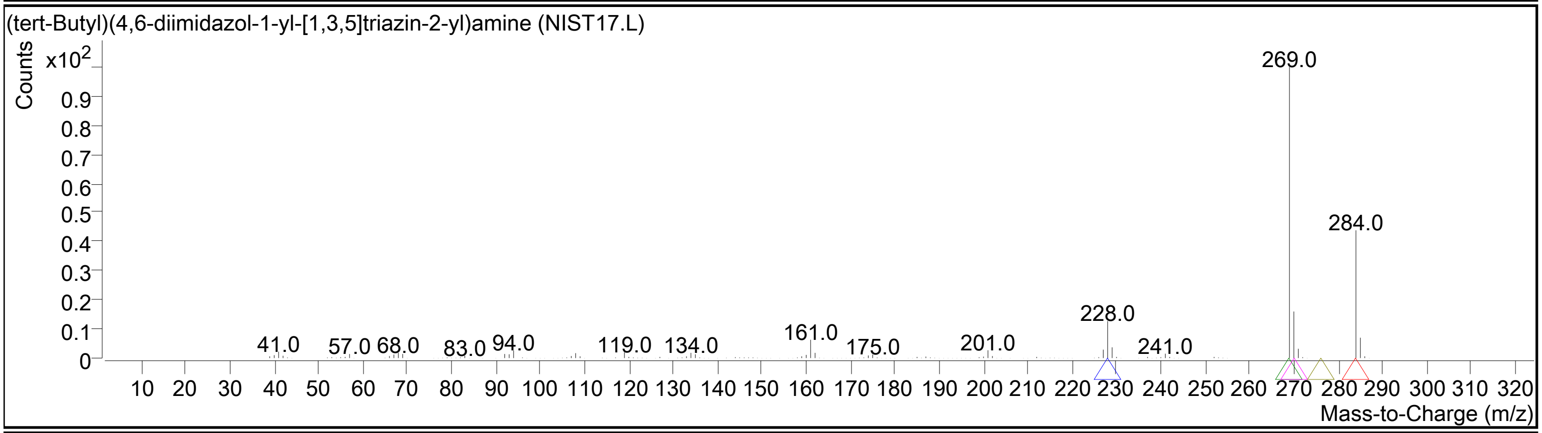

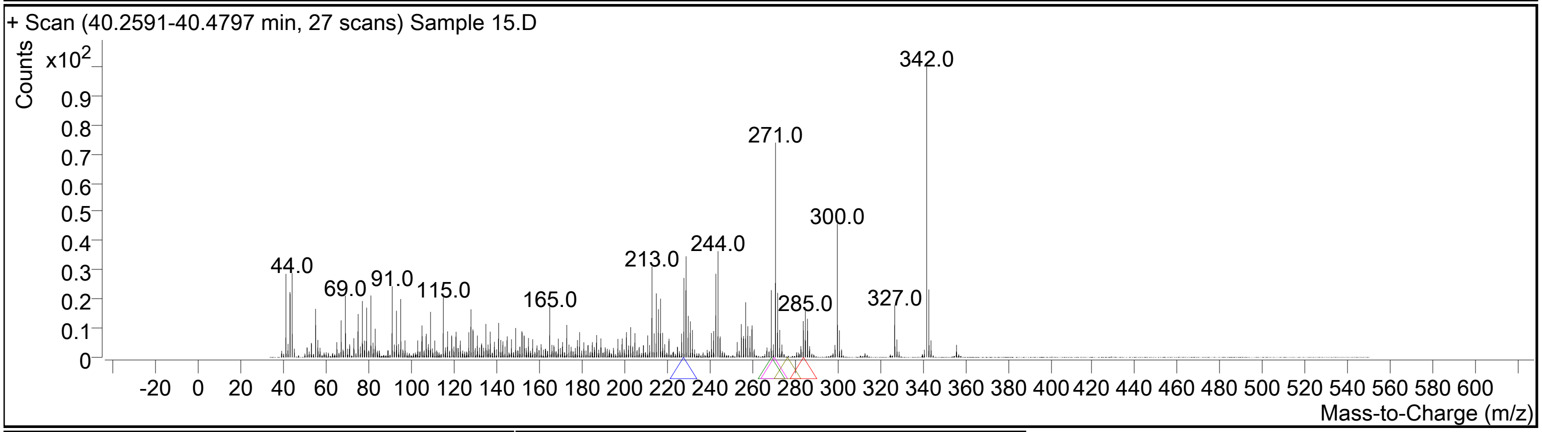

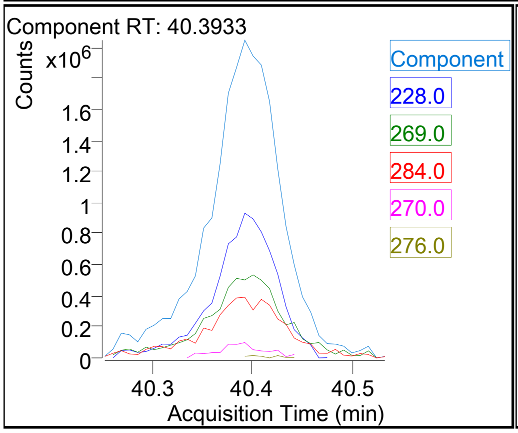

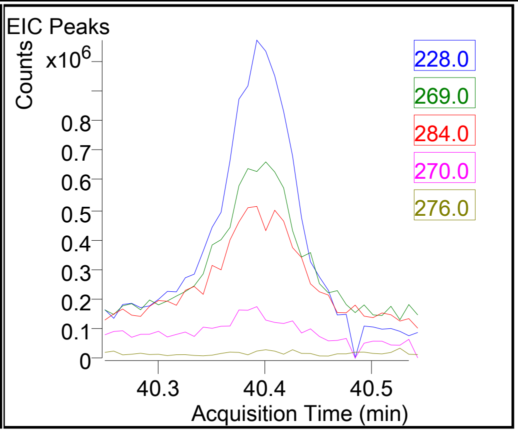

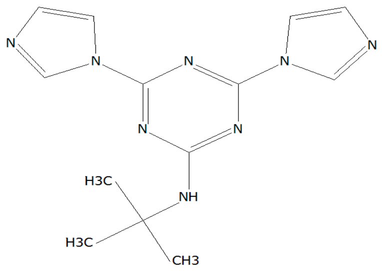

Component RT	Compound Name	Component Area	Match Factor	CAS#	Formula	Estimated Conc.
40.7060	.delta.9-Tetrahydrocannabivarin	1045443725.7	80.7	31262-37-0	C19H26O2	

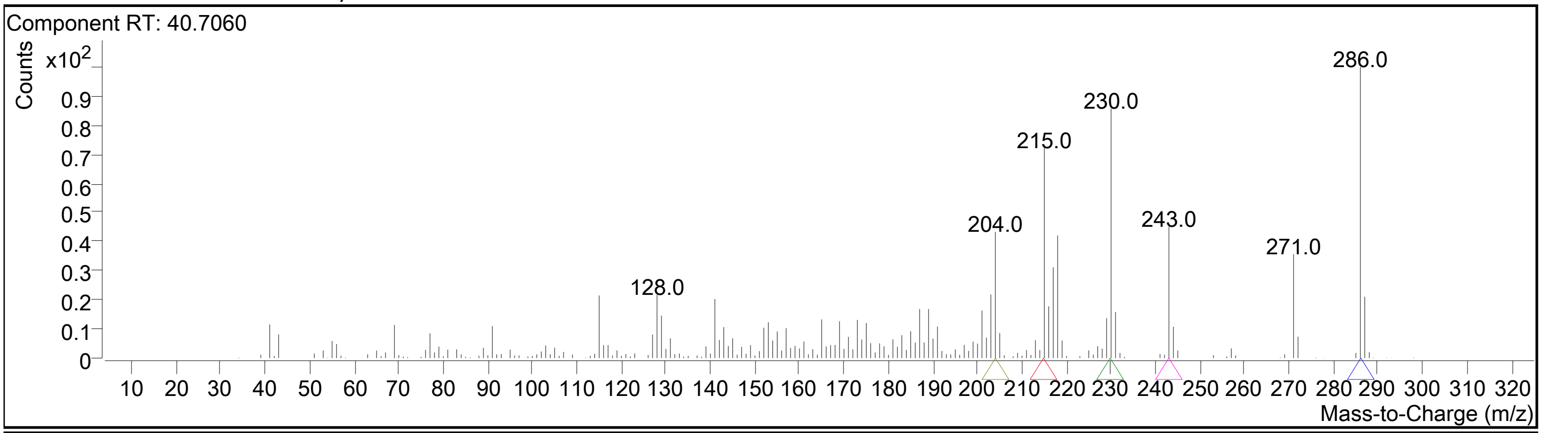

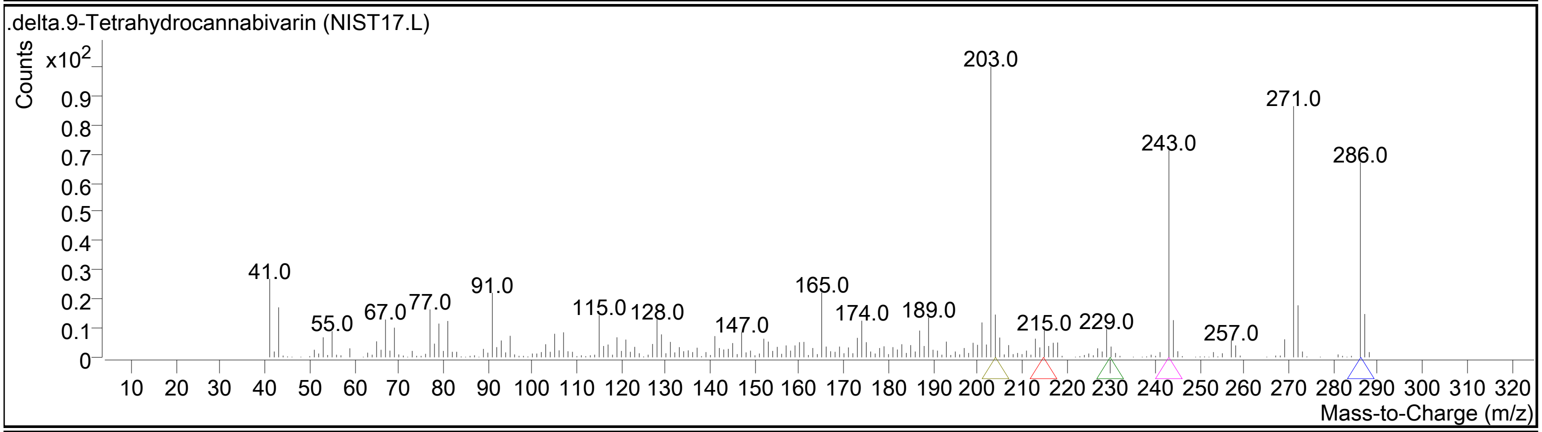

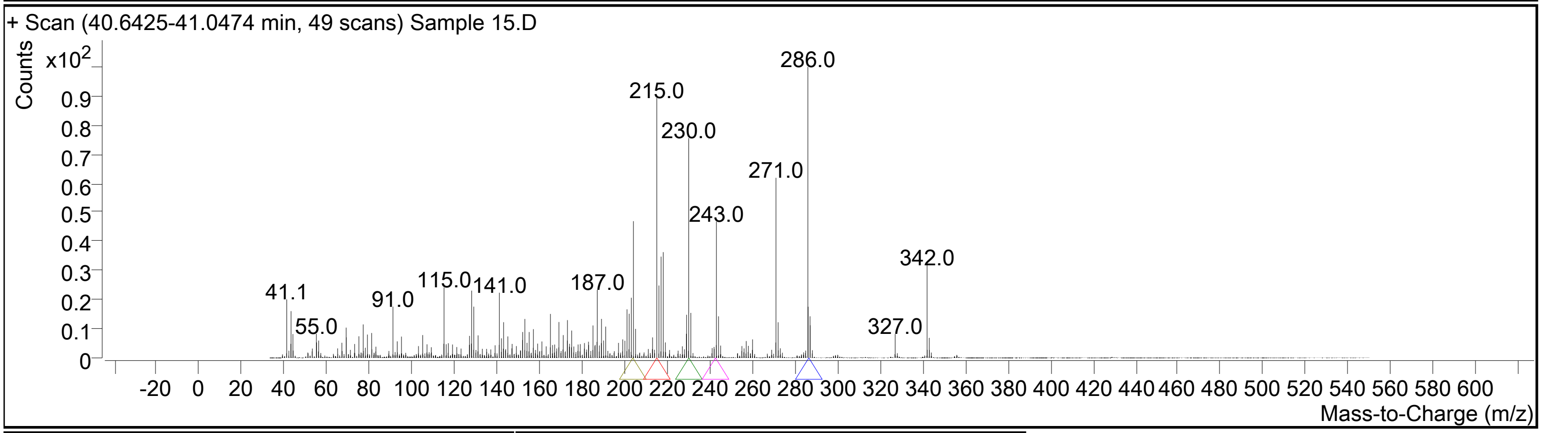

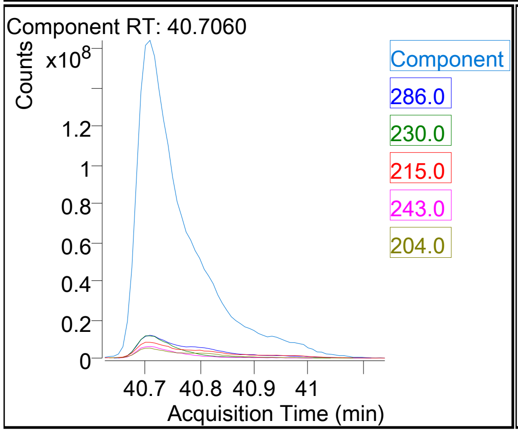

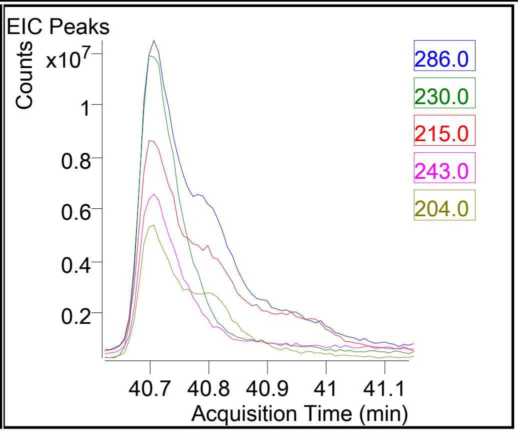

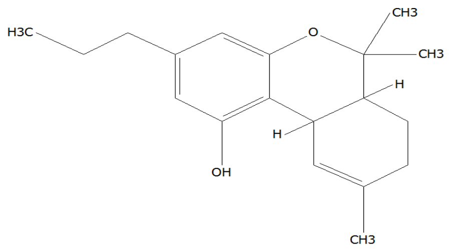

Component RT	Compound Name	Component Area	Match Factor	CAS#	Formula	Estimated Conc.
40.8016	Pyrimidine, 4-(2-hydroxy-5-methoxyphenyl)-	20394122.2	69.5	97630-77-8	C11H10N2O2	

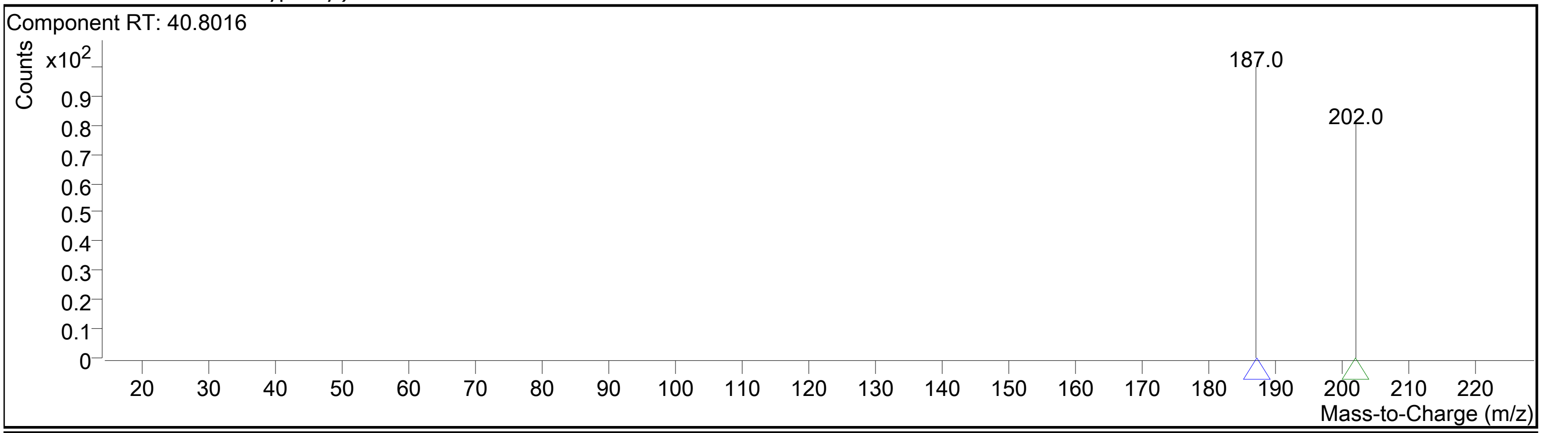

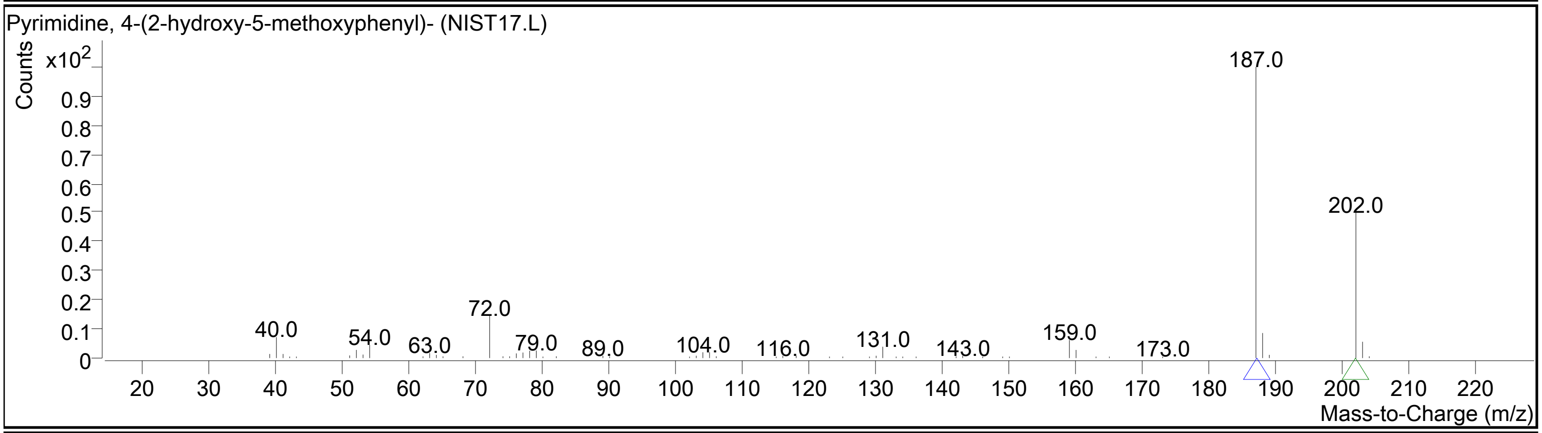

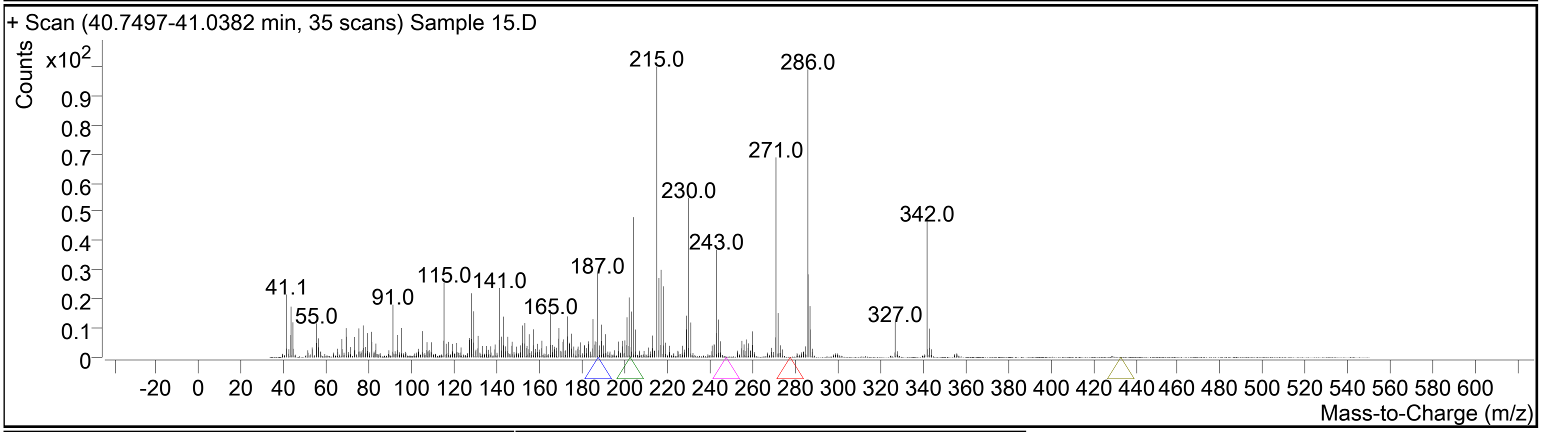

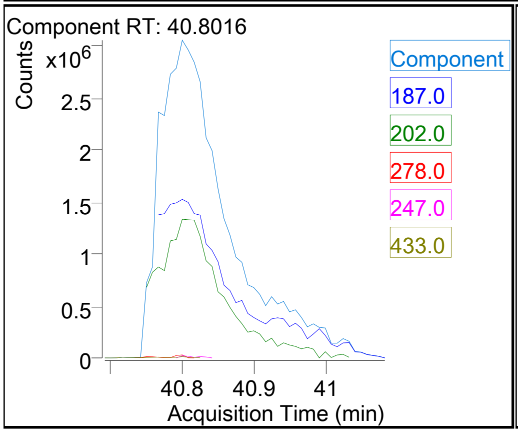

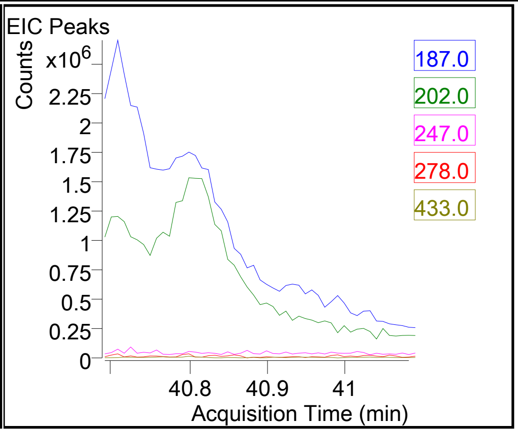

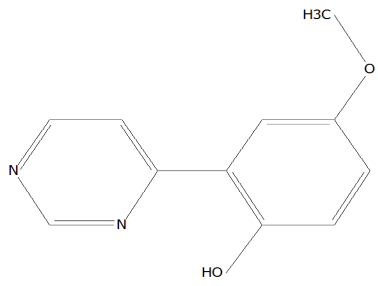

Component RT	Compound Name	Component Area	Match Factor	CAS#	Formula	Estimated Conc.
41.2933	2(1H)-Phenanthrenone, 4a,9,10,10a-tetrahydro-6-hydroxy-1,1,4a-trimethyl-7-(1-methylethyl)-, (4aS-trans)-	2429782318.8	78.4	18326-19-7	C20H26O2	

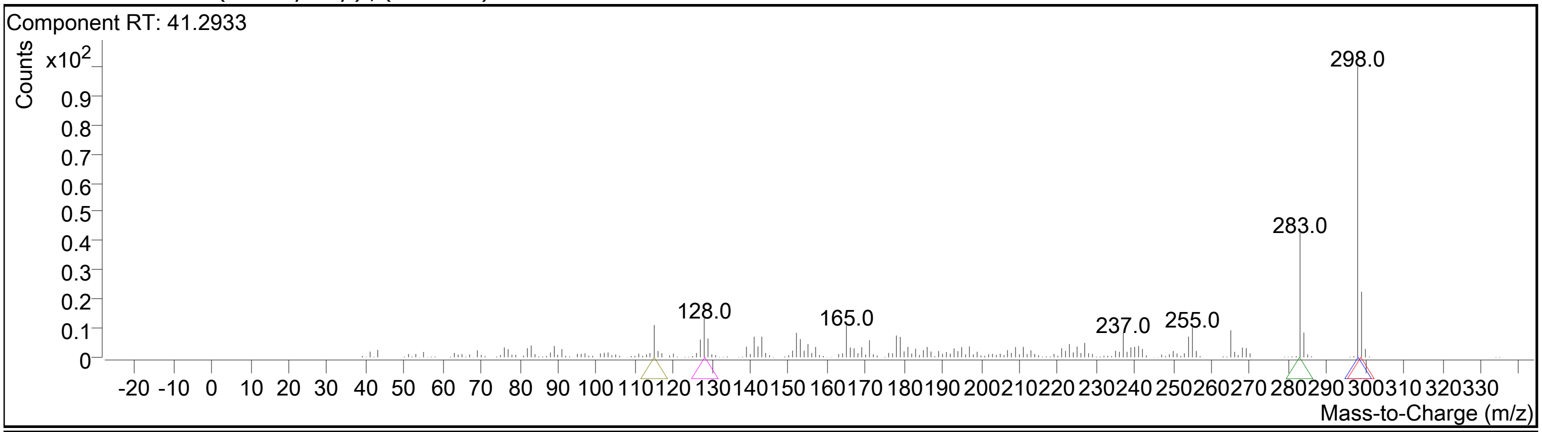

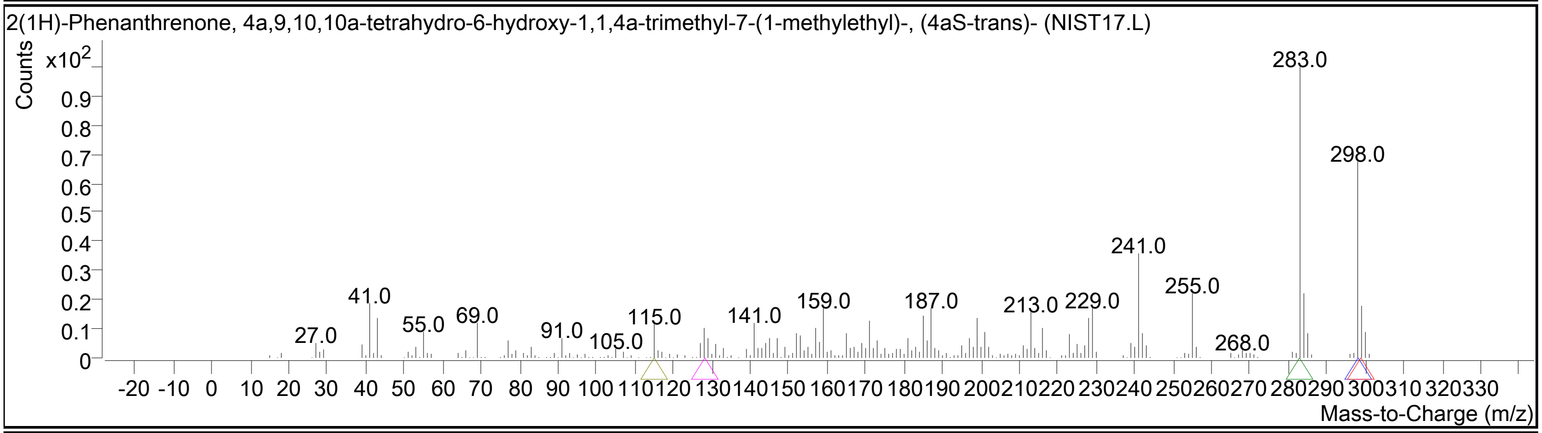

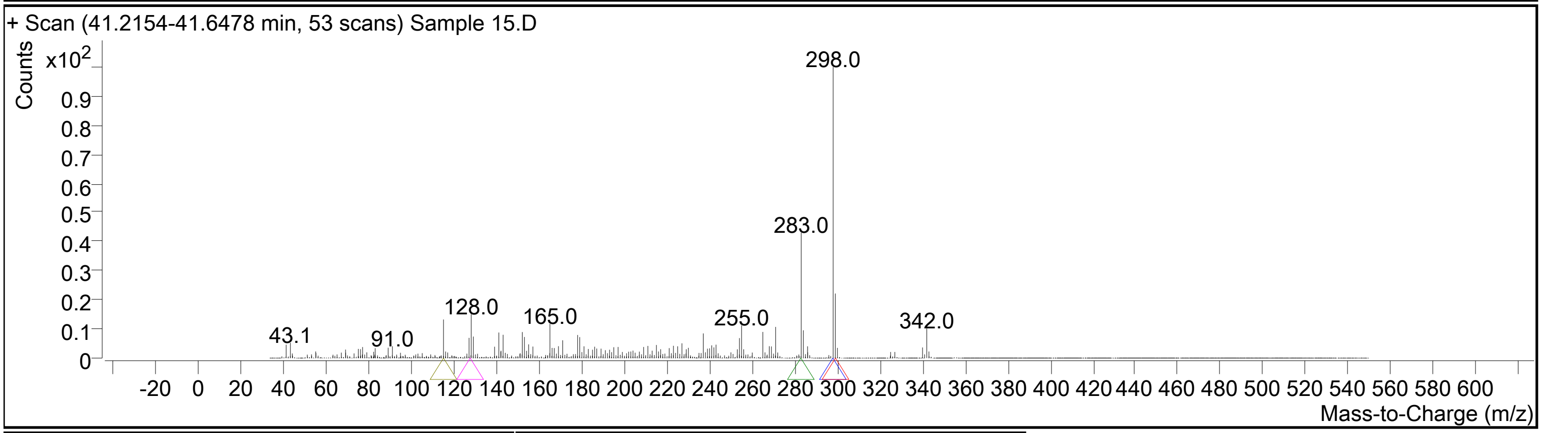

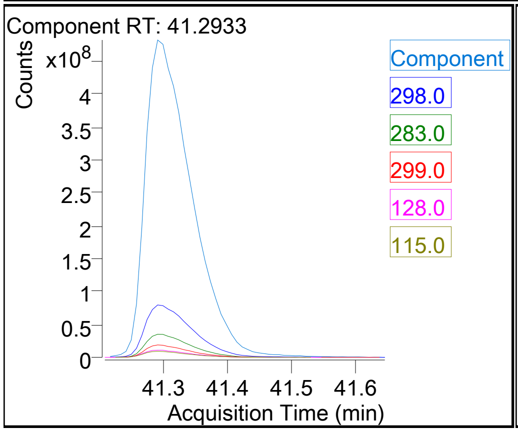

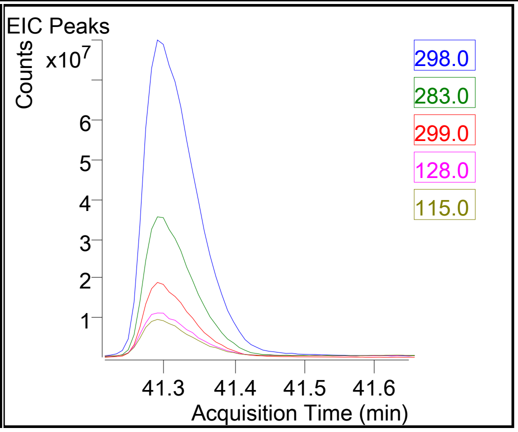

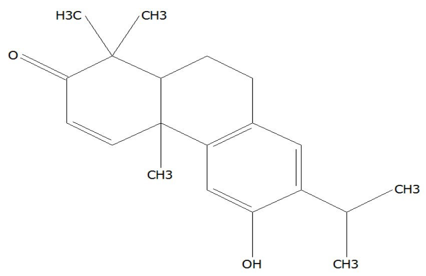

Library Search Results - NonTarget Hits with Details

Agilent Technologies

Component RT	Compound Name	Component Area	Match Factor	CAS#	Formula	Estimated Conc.
42.1646	4'-Amino-6-methoxyaurone	27771080.6	81.1	77764-96-6	C16H13NO3	

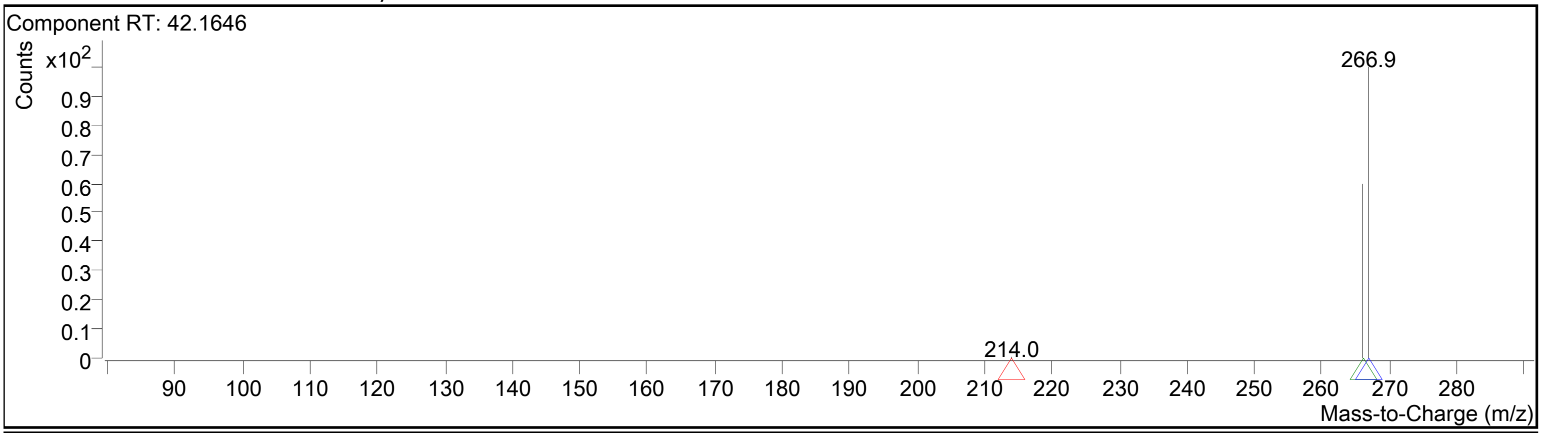

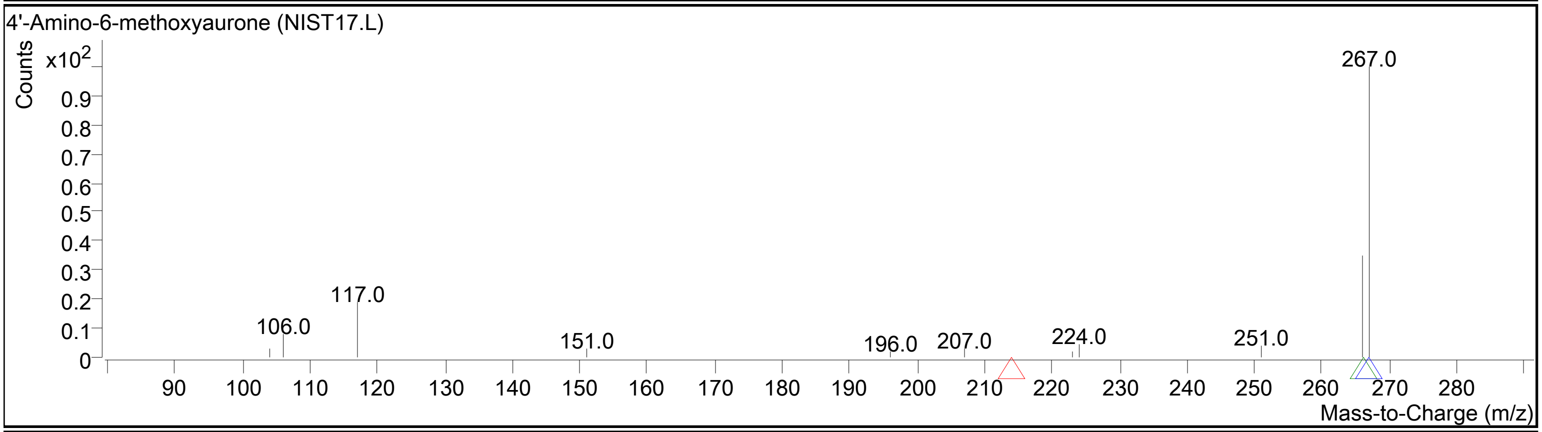

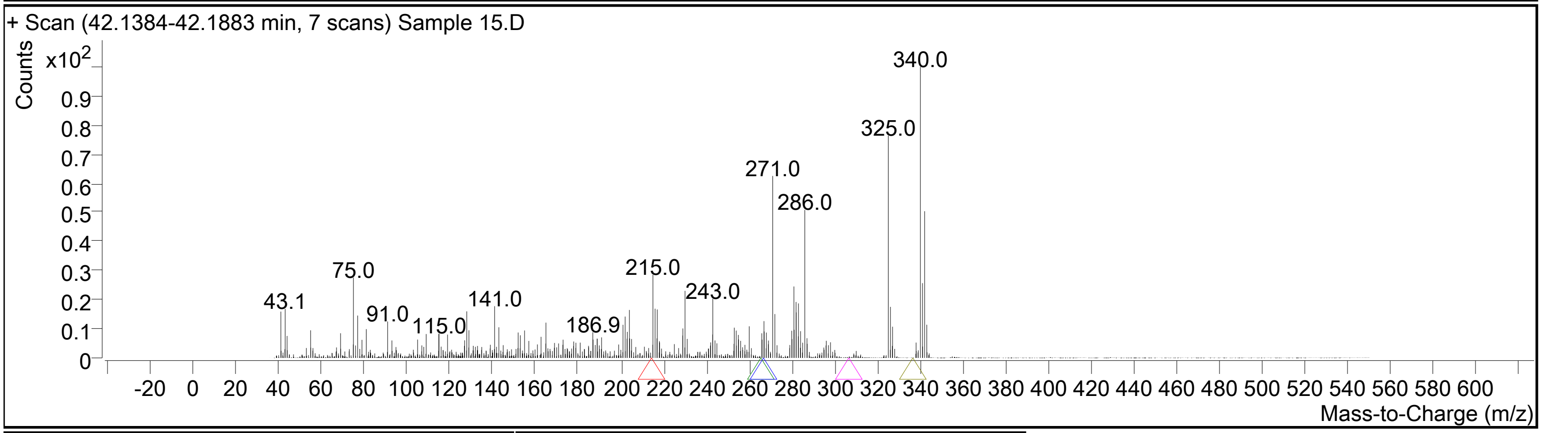

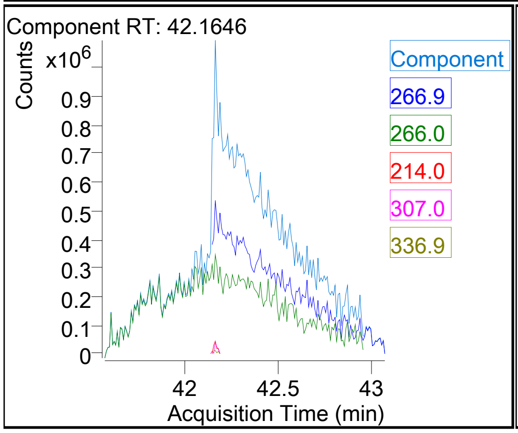

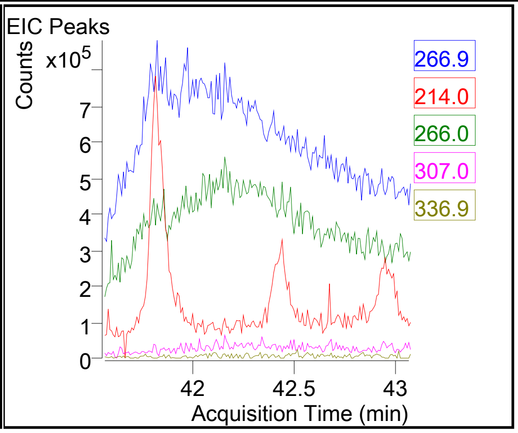

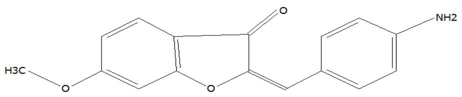

Agilent Technologies

Component RT	Compound Name	Component Area	Match Factor	CAS#	Formula	Estimated Conc.
42.4540	2-(4'-Methoxyphenyl)-2-(3'-methyl-4'methoxyphenyl)propane	3185706.5	66.0	1000283-53-6	C18H22O2	

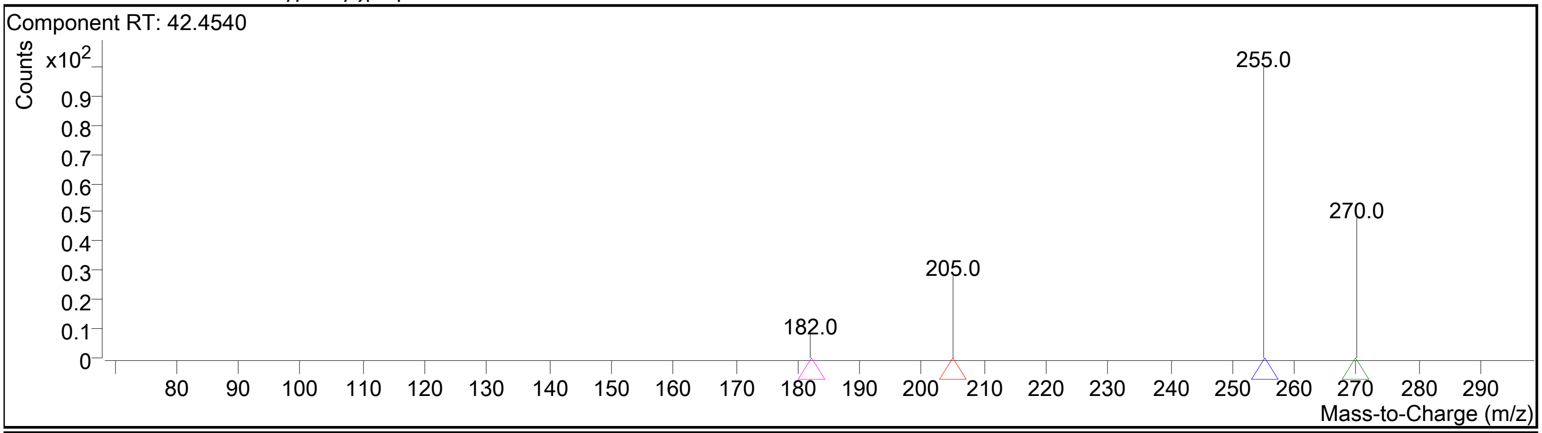

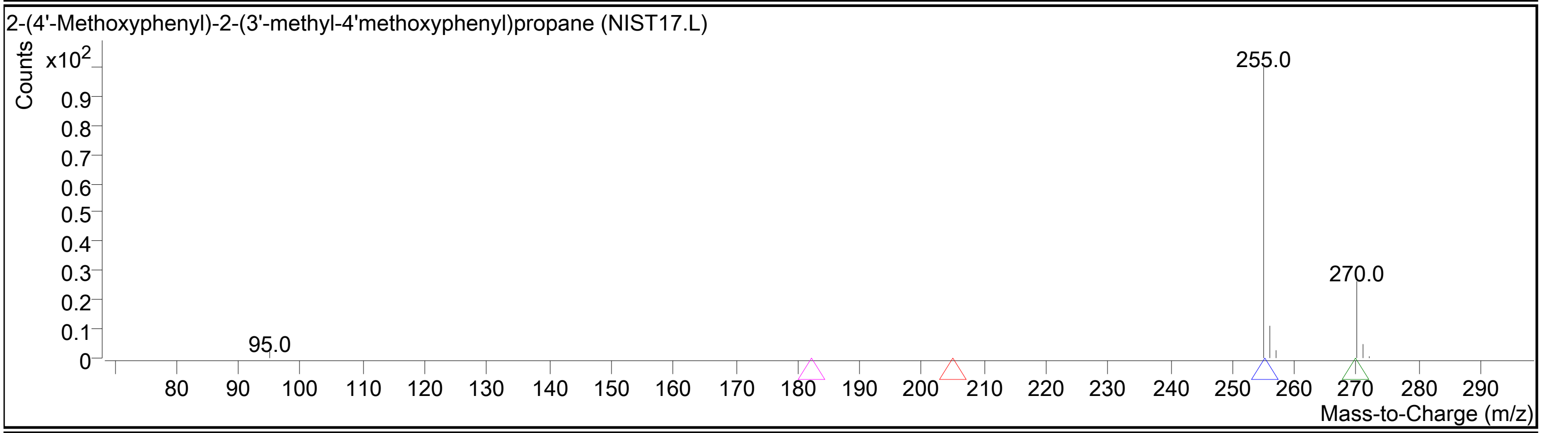

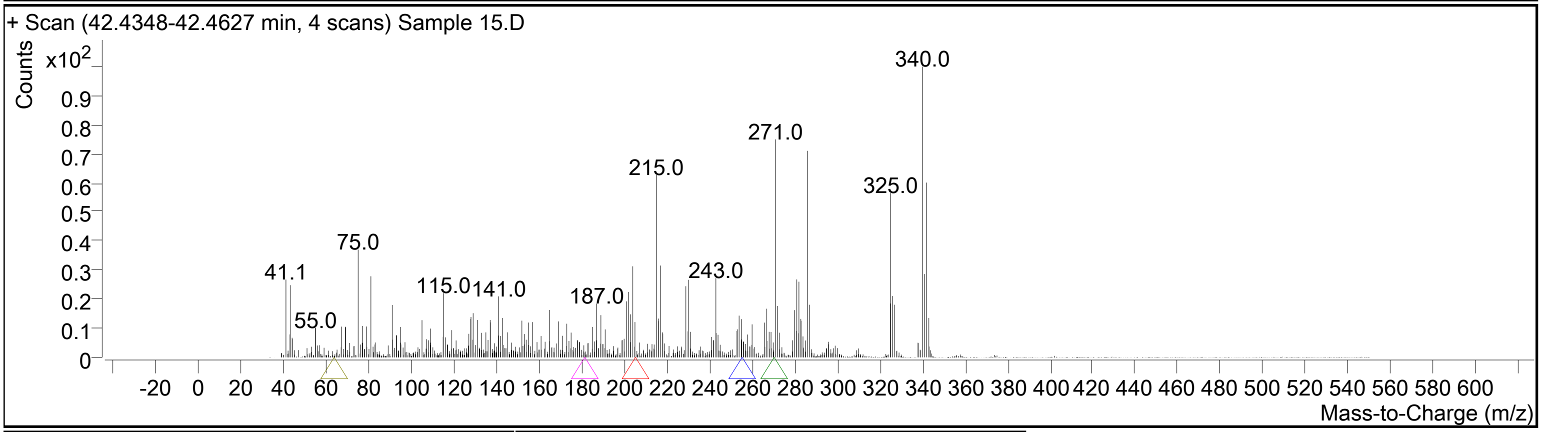

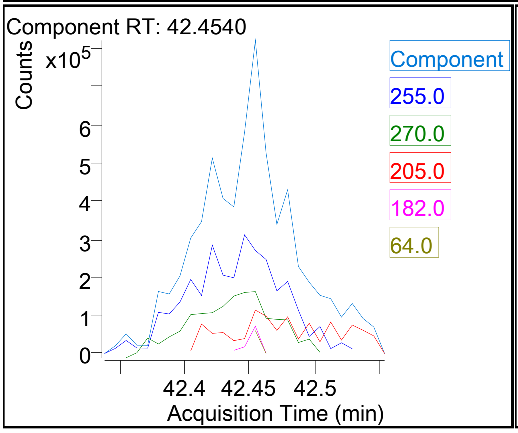

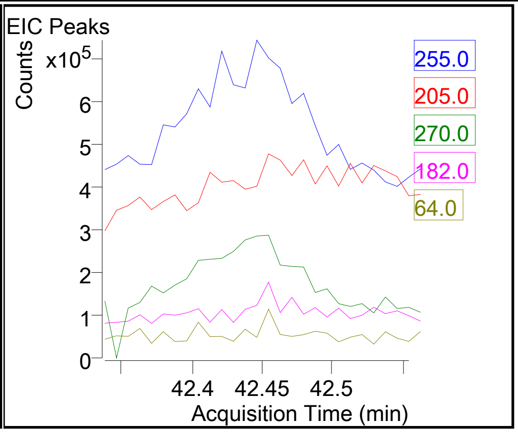

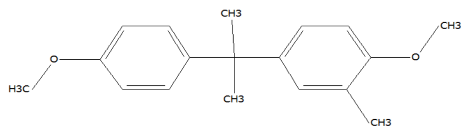

Component RT	Compound Name	Component Area	Match Factor	CAS#	Formula	Estimated Conc.
43.4056	6H-[1,3]Dioxolo[5,6]benzofuro[3,2-c][1]benzopyran-2-ol, 6a,12a-dihydro-3-methoxy-, (6ar-cis)-	27691440.2	67.8	30461-92-8	C17H14O6	

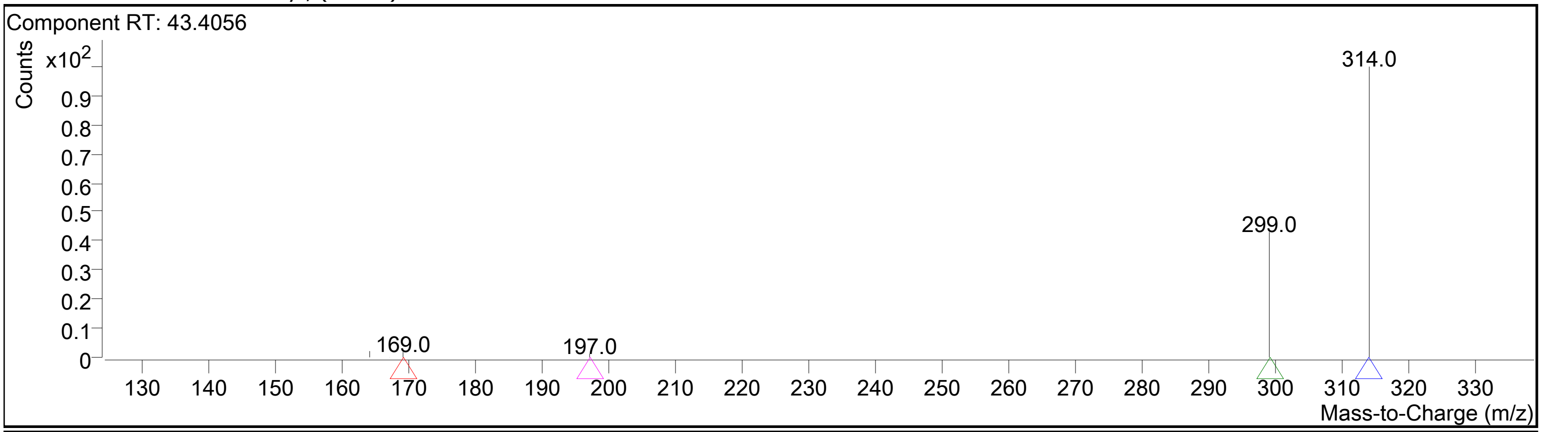

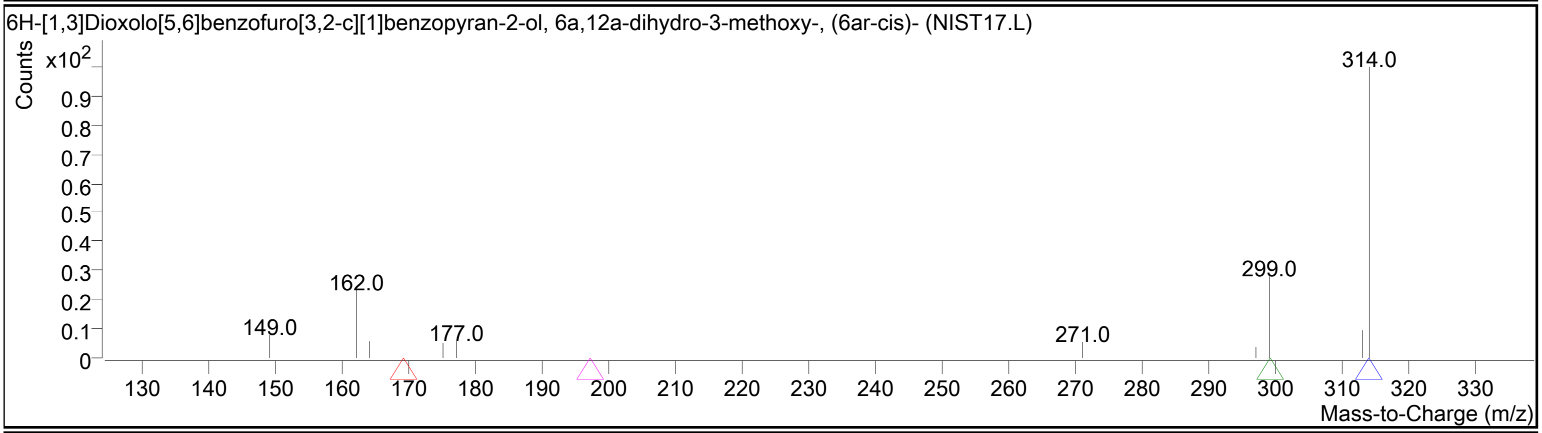

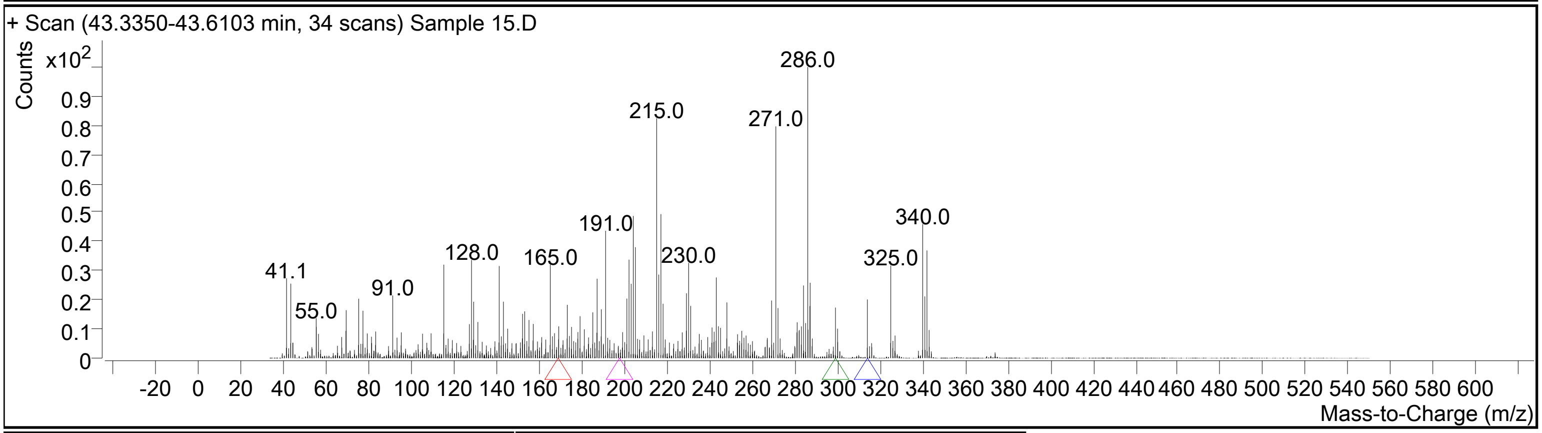

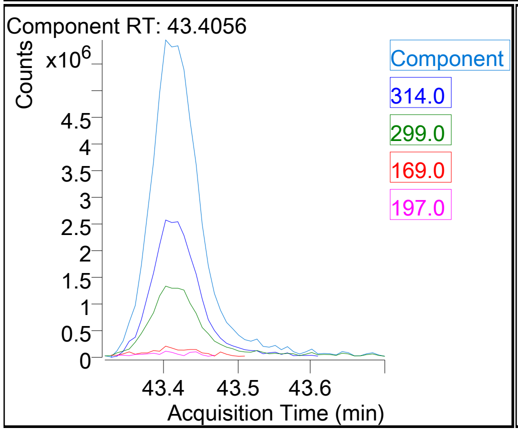

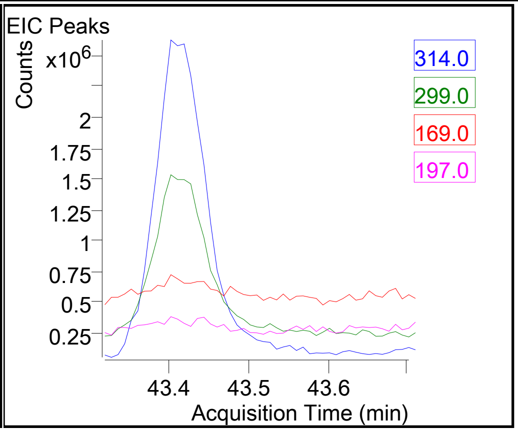

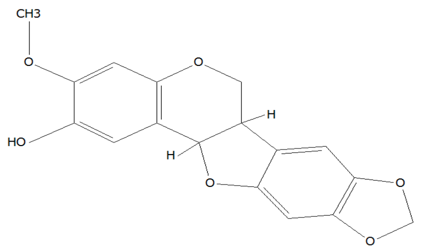

Generated at 12:24 PM on 2/25/2021

Library Search Results - NonTarget Hits with Details

Agilent Technologies

Component RT	Compound Name	Component Area	Match Factor	CAS#	Formula	Estimated Conc.
44.1592	Benzo[1,2-b:5,4-b']difuran-4,8-dione, 5-methyl-2-(1-methylethenyl)-	3015723.7	72.2	26962-40-3	C14H10O4	

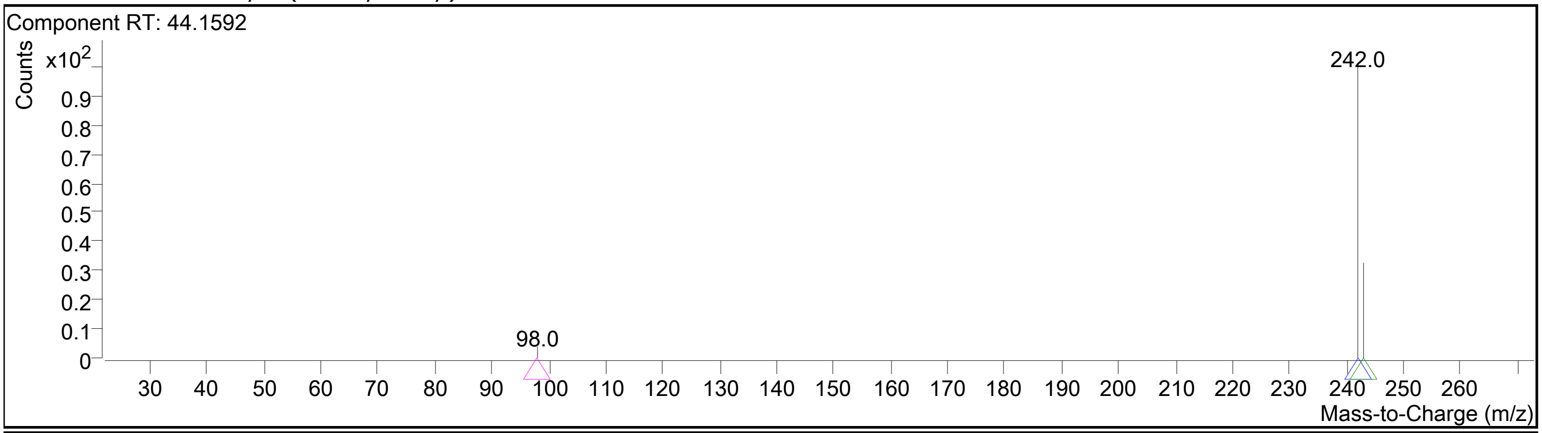

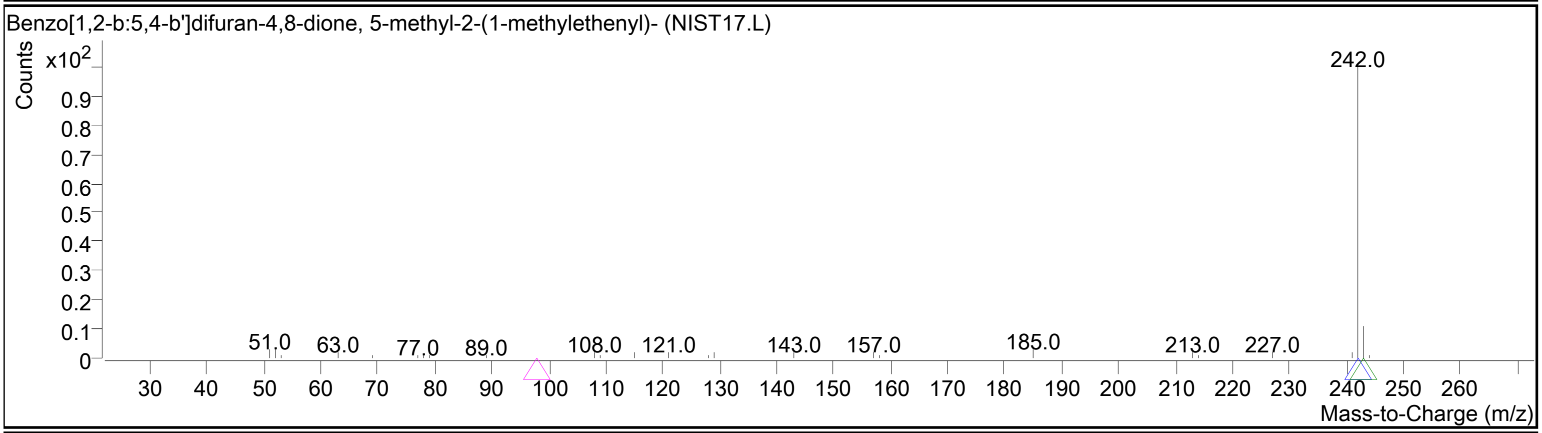

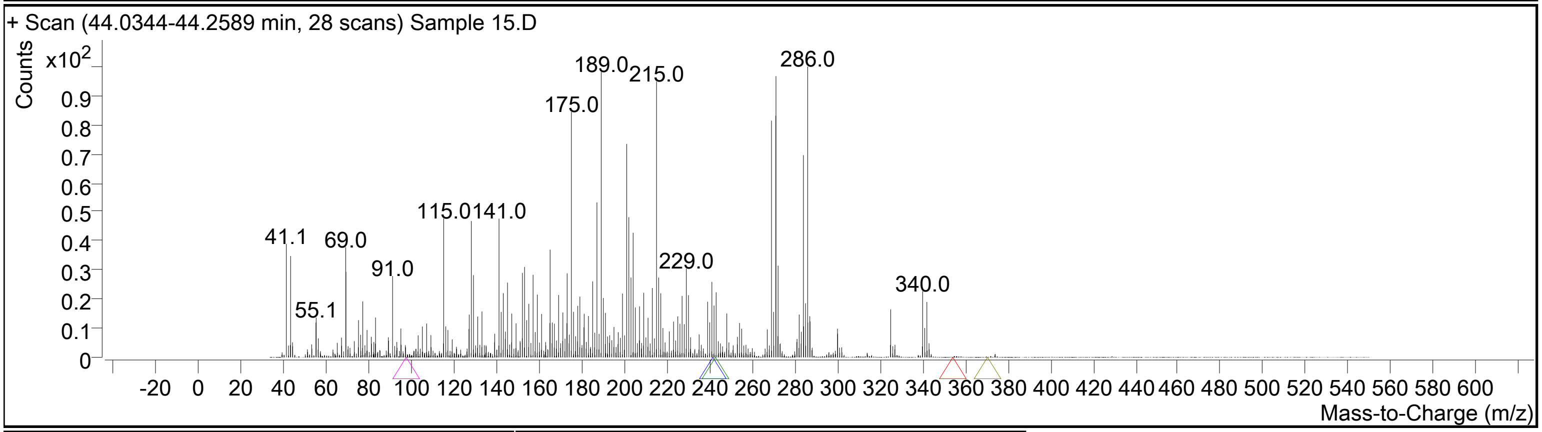

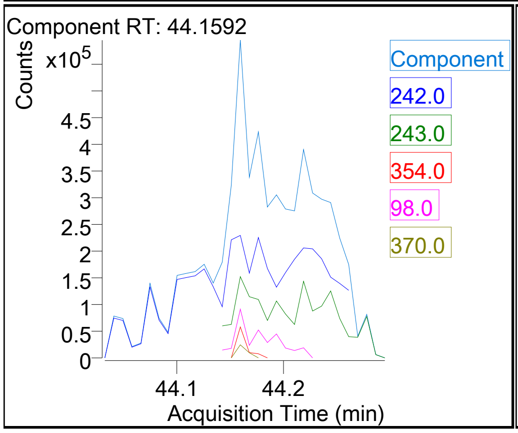

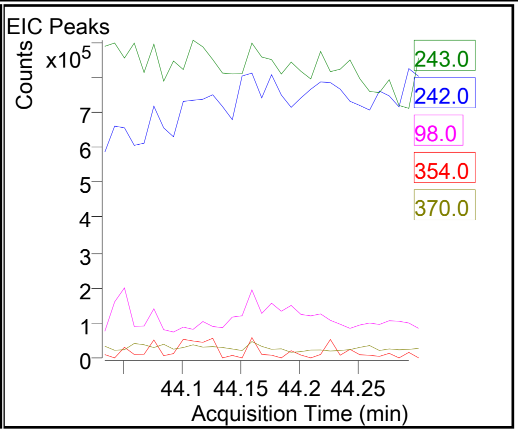

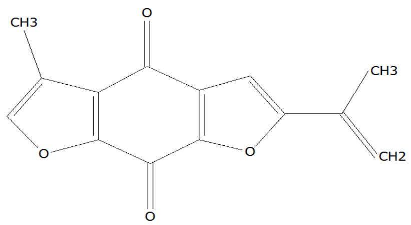

Generated at 12:24 PM on 2/25/2021

Component RT	Compound Name	Component Area	Match Factor	CAS#	Formula	Estimated Conc.
44.1829	13-Isopropylpodocarpen-12-ol-20-al	550679394.3	90.3	24035-37-8	C20H28O2	

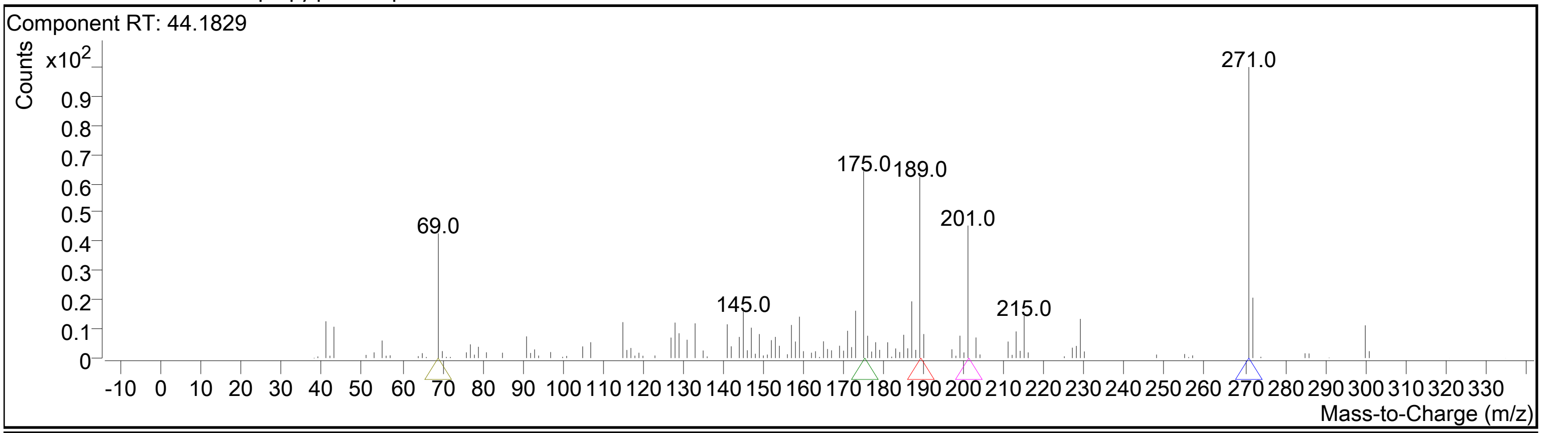

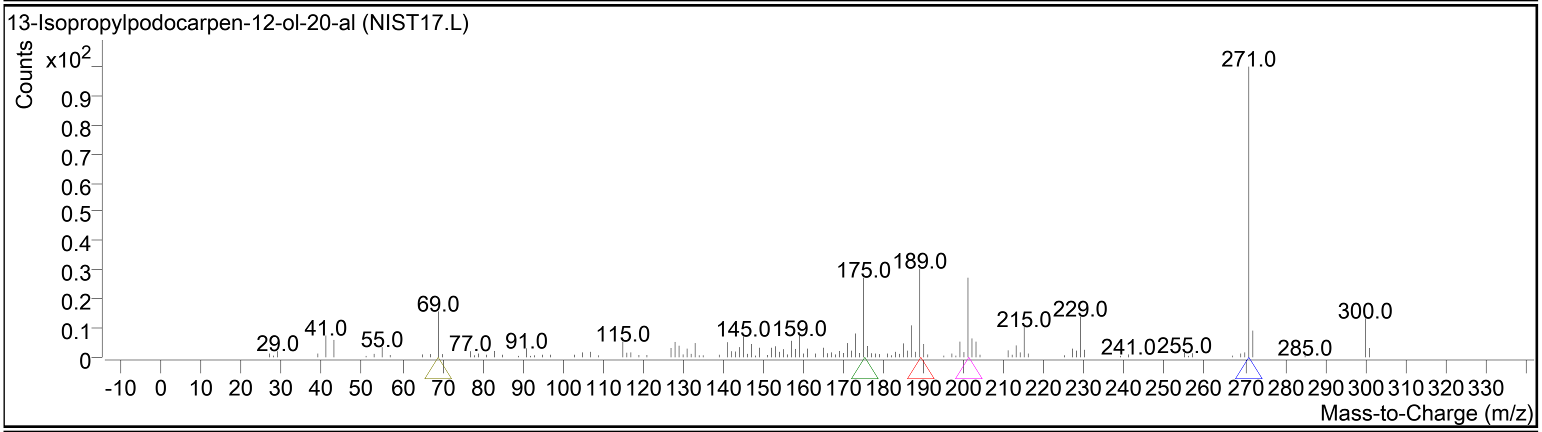

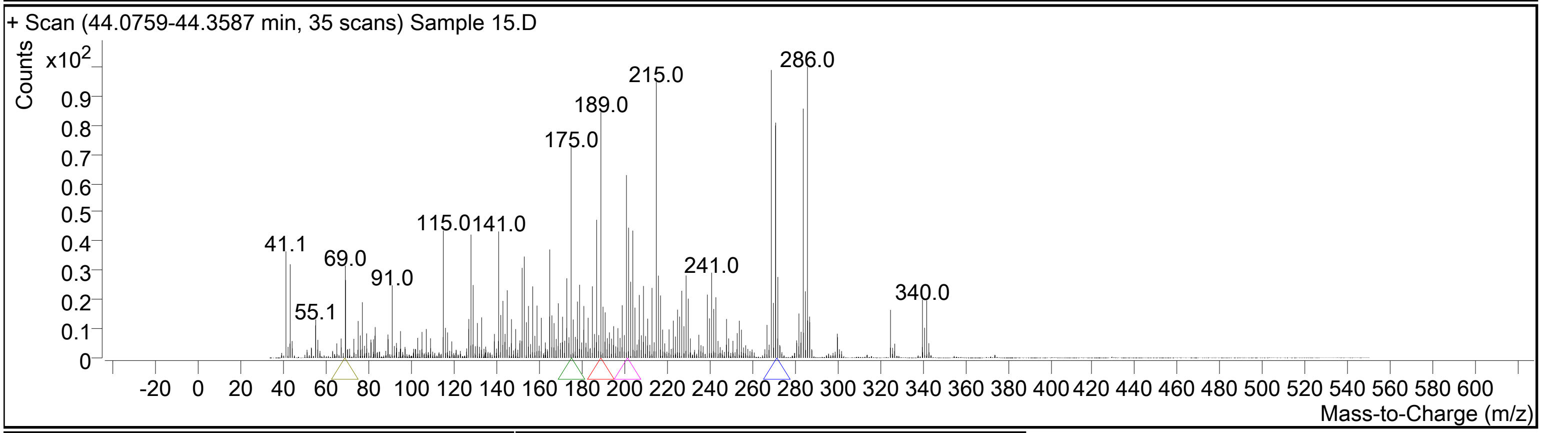

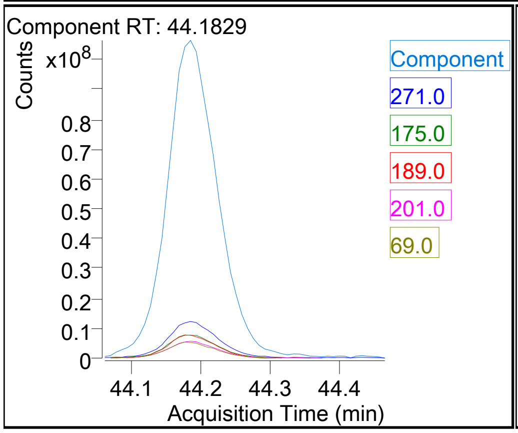

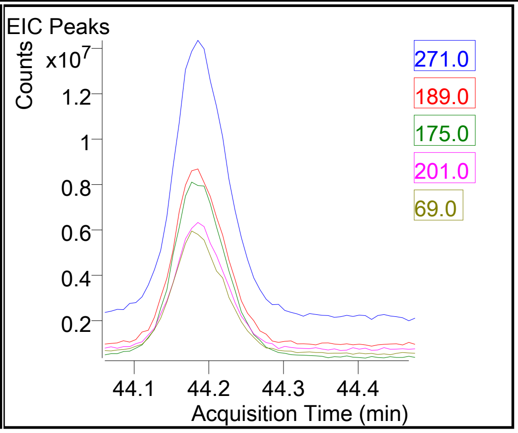

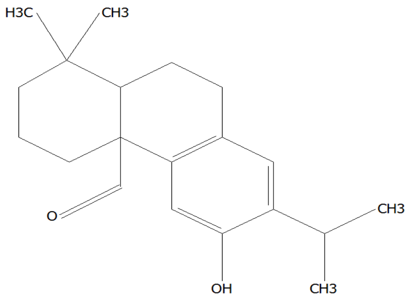

Library Search Results - NonTarget Hits with Details

Component RT	Compound Name	Component Area	Match Factor	CAS#	Formula	Estimated Conc.
44.6501	Phthalic acid, di(2-propylpentyl) ester	47610761.1	76.9	1000377-93-5	C24H38O4	

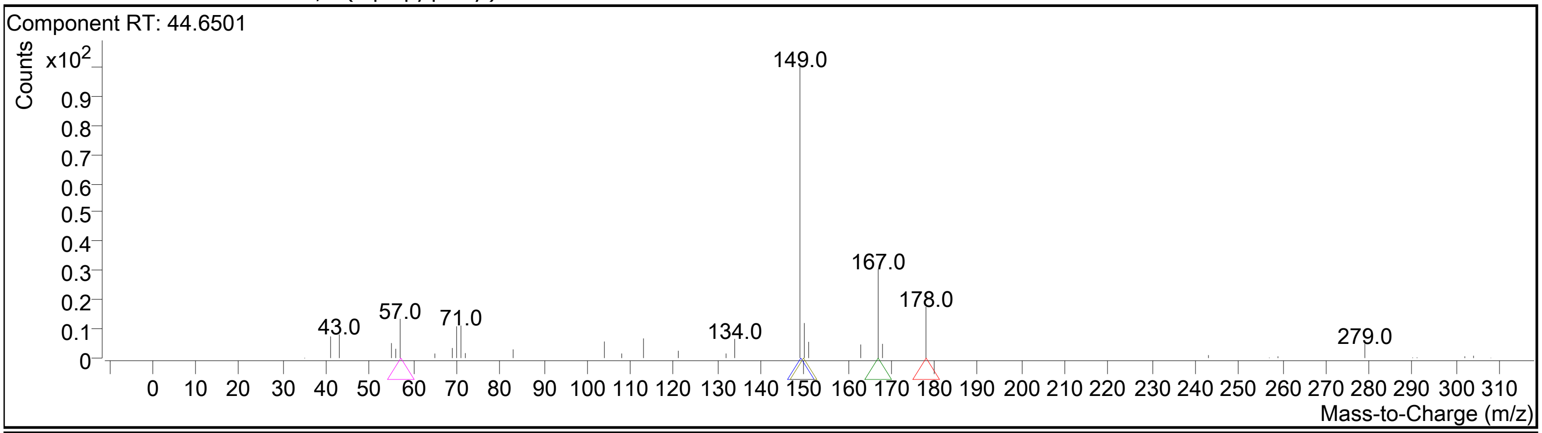

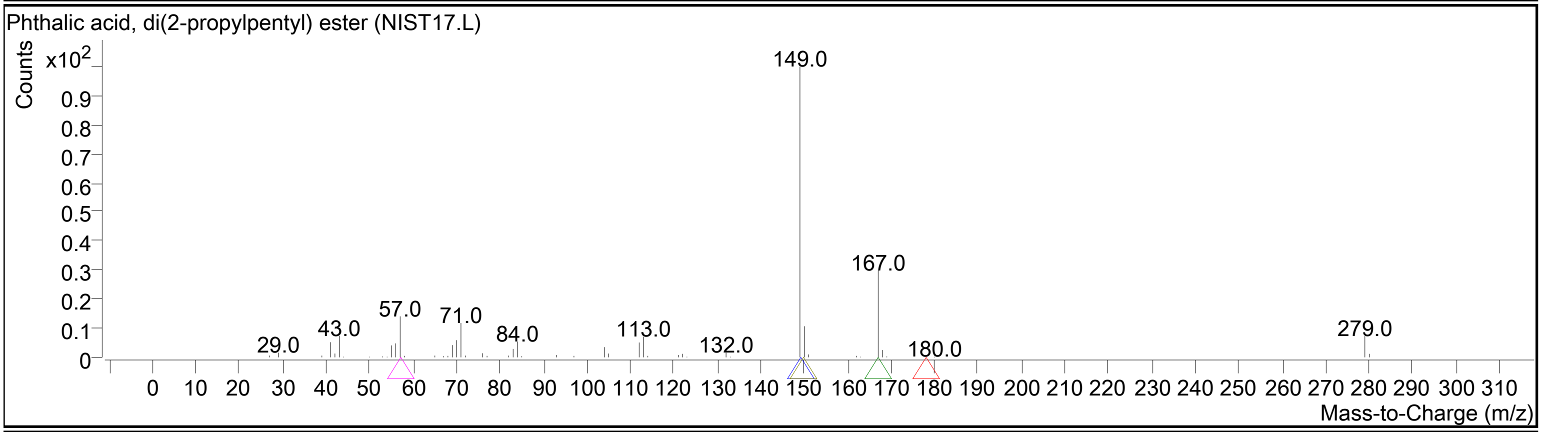

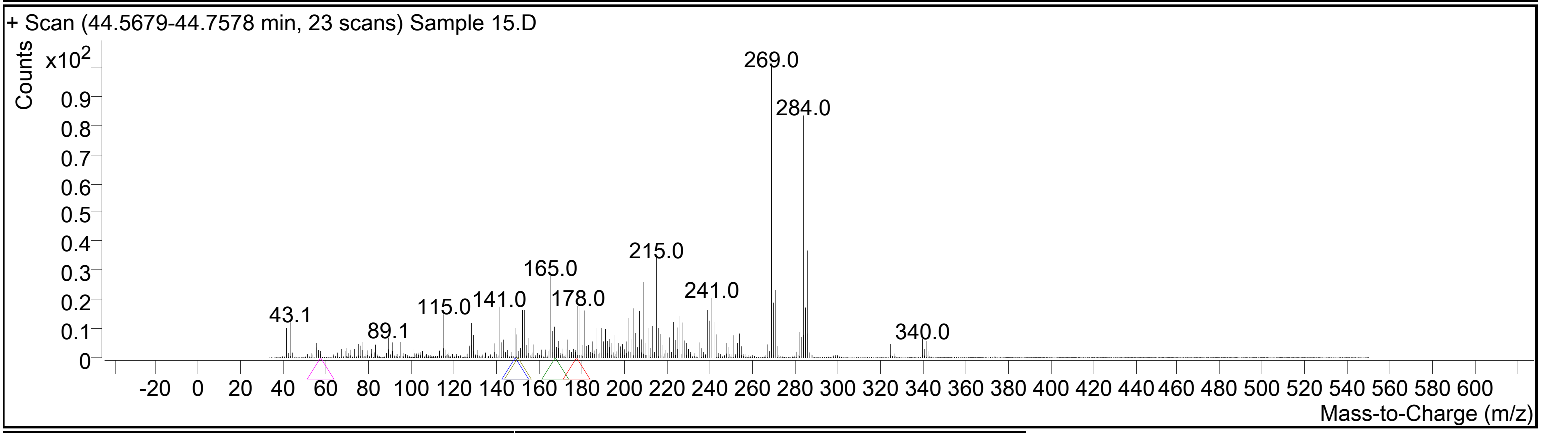

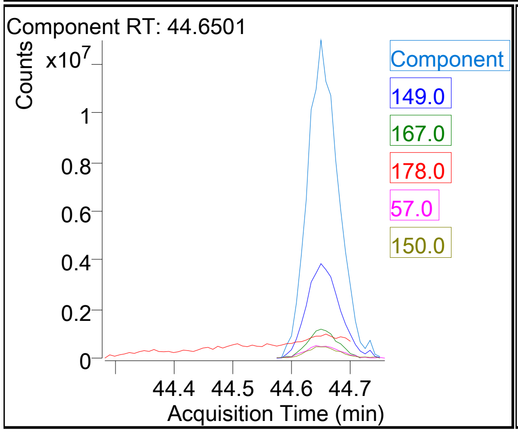

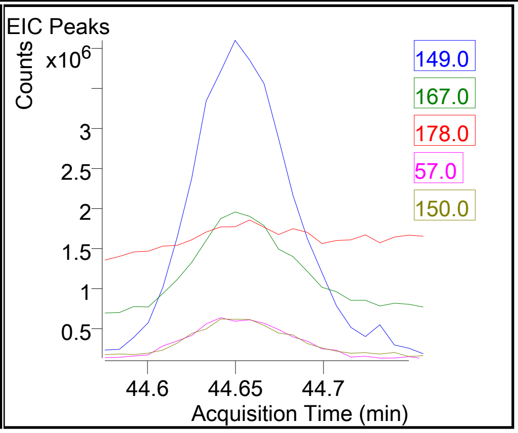

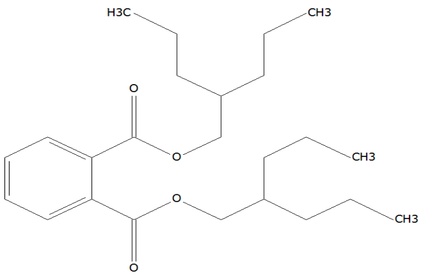

Component RT	Compound Name	Component Area	Match Factor	CAS#	Formula	Estimated Conc.
44.8575	2-Hydroxy-3-methylanthraquinone, O-trimethylsilyl	8921824.7	65.8	91701-15-4	C18H18O3Si	

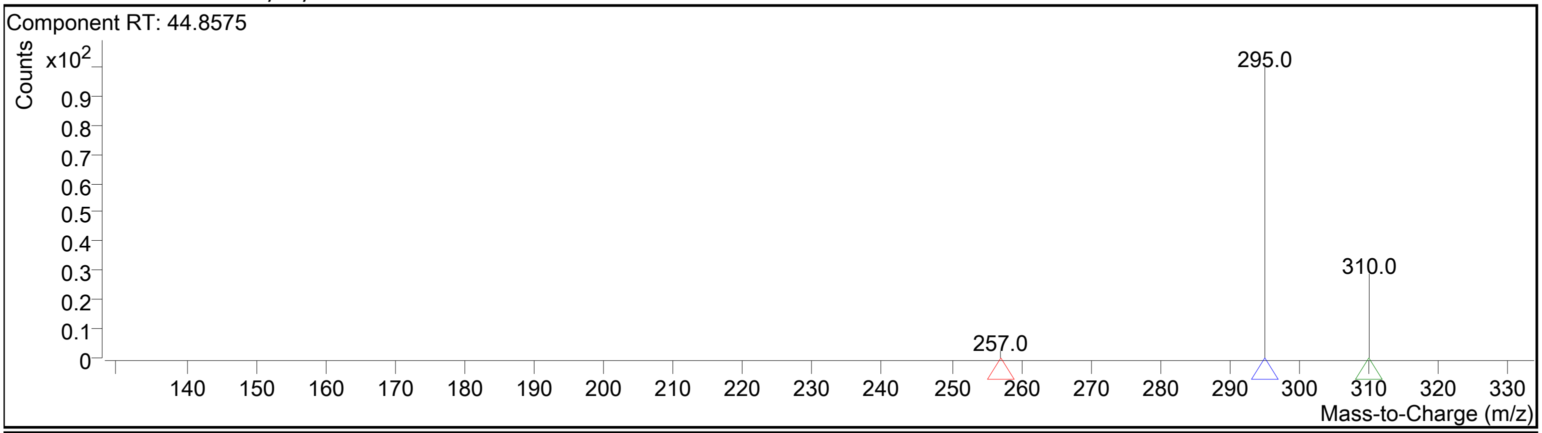

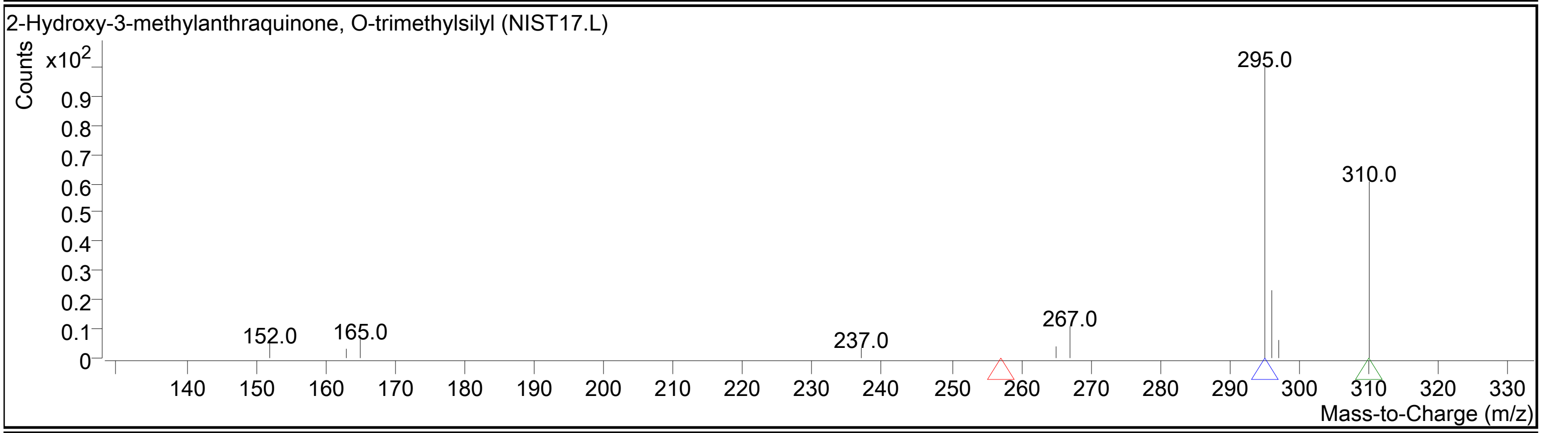

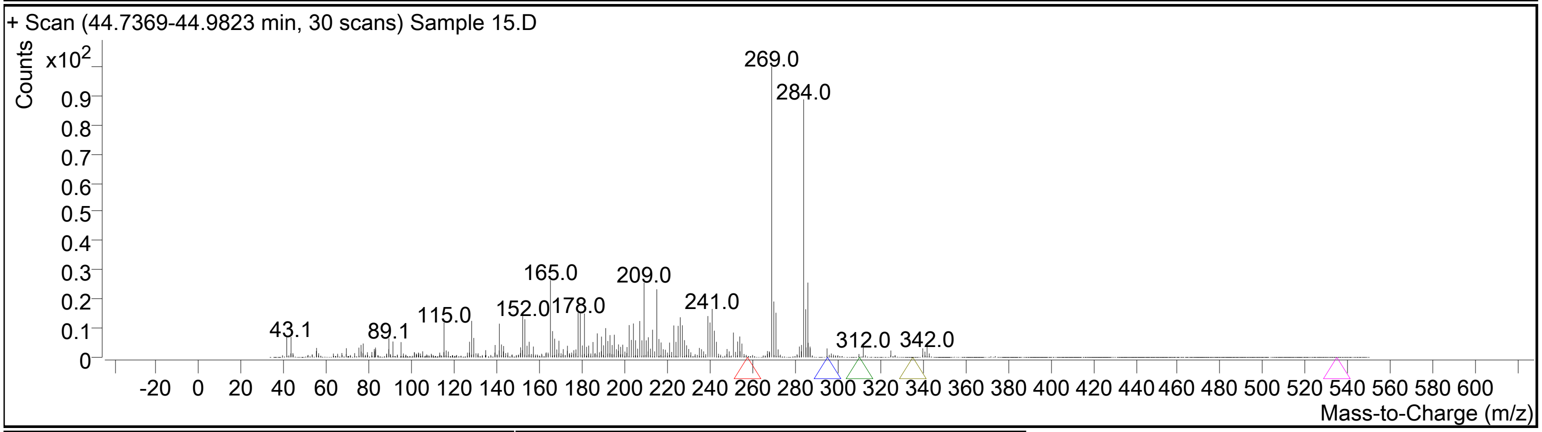

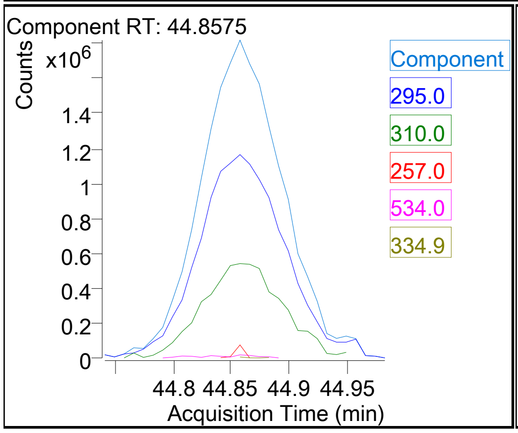

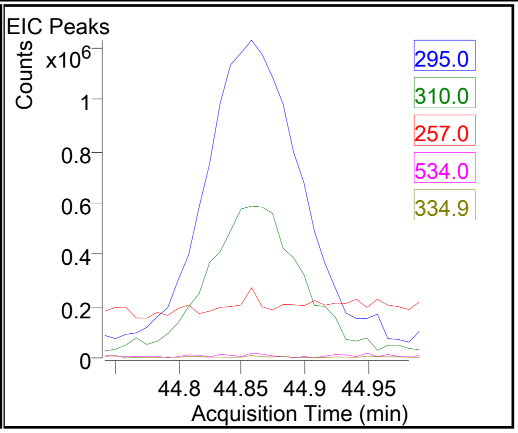

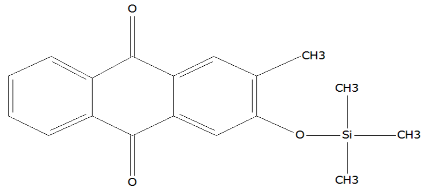

Generated at 12:24 PM on 2/25/2021

Component RT	Compound Name	Component Area	Match Factor	CAS#	Formula	Estimated Conc.
45.1100	2-(2',4'-Dimethoxyphenyl)-6-methoxy-benzofuran	2501061634.3	79.0	67685-23-8	C17H16O4	

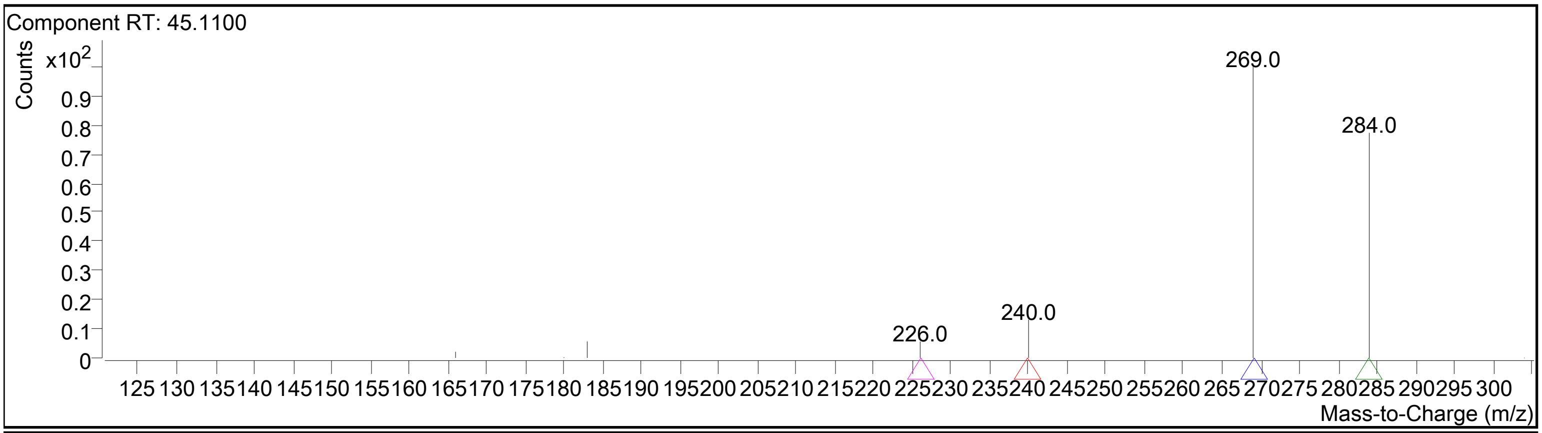

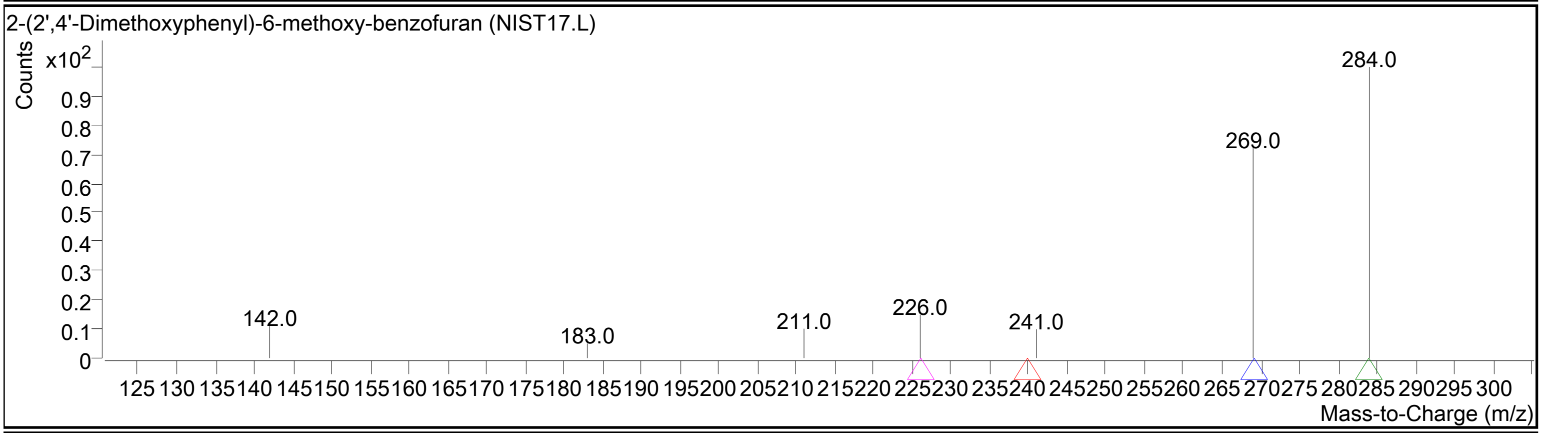

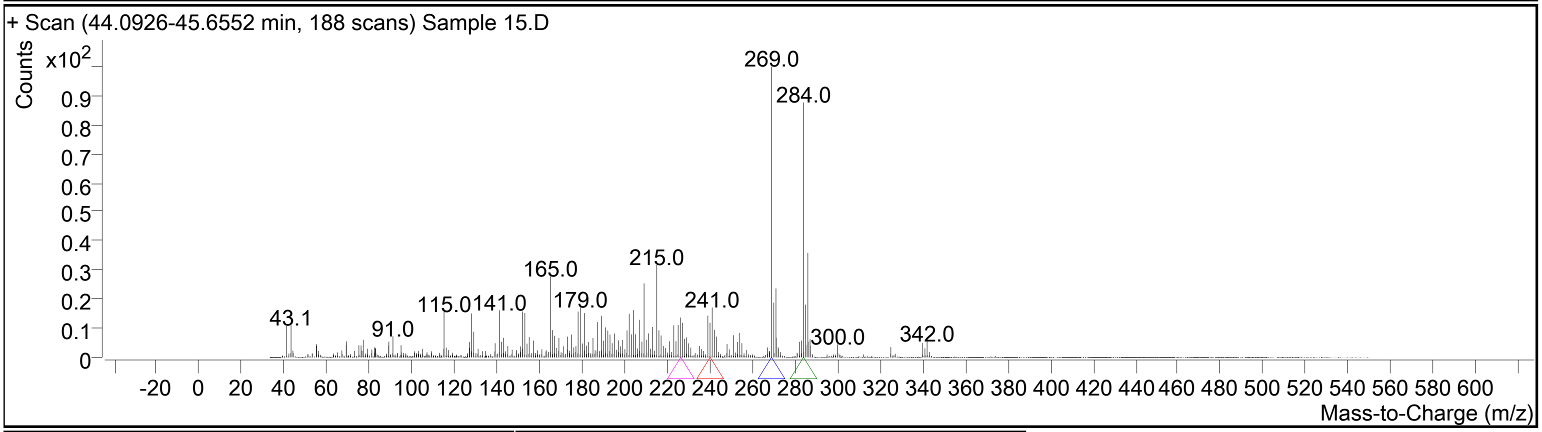

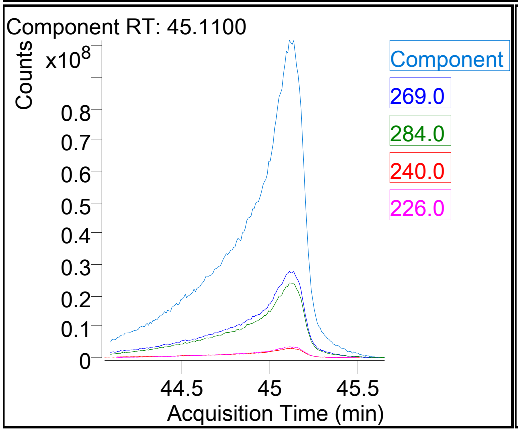

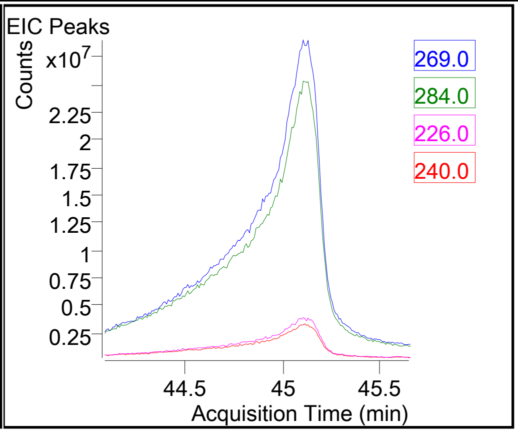

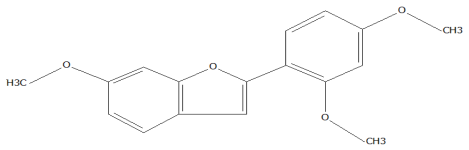

Library Search Results - NonTarget Hits with Details

Agilent Technologies

Component RT	Compound Name	Component Area	Match Factor	CAS#	Formula	Estimated Conc.
45.5944	2(1H)-Phenanthrenone, 3,4,4a,9,10,10a-hexahydro-6-hydroxy-1,1,4a-trimethyl-7-(1-methylethyl)-, (4aS-trans)-	123725426.8	70.2	472-37-7	C20H28O2	

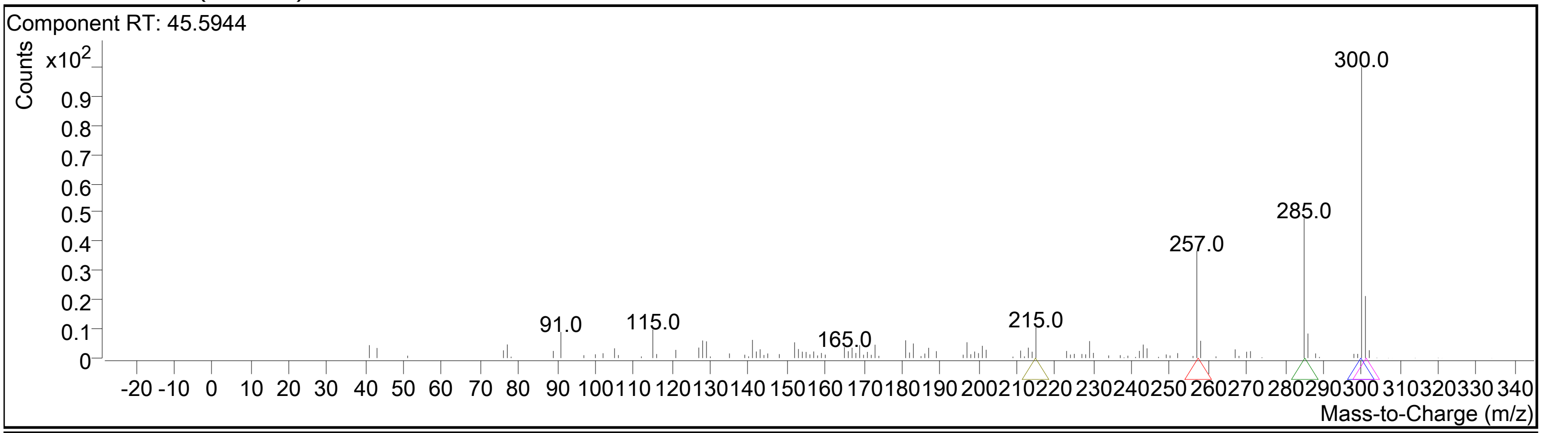

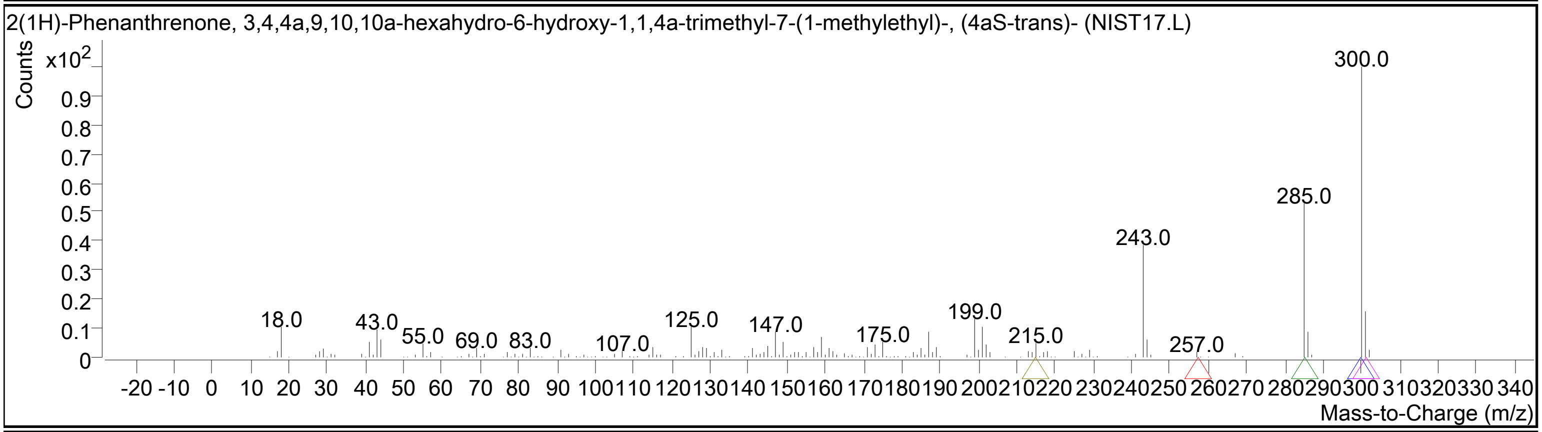

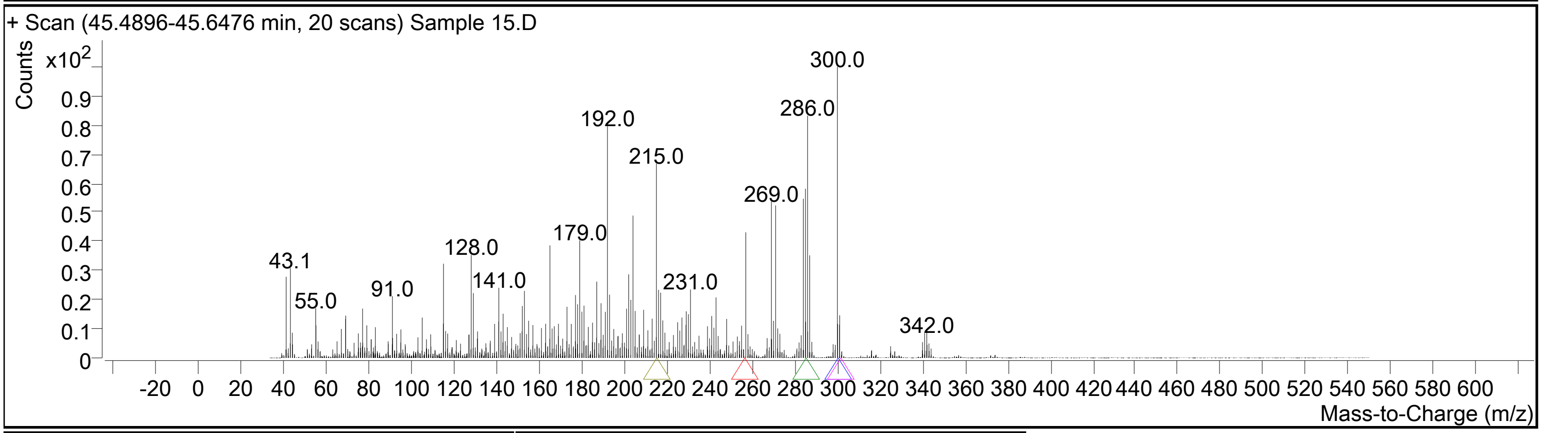

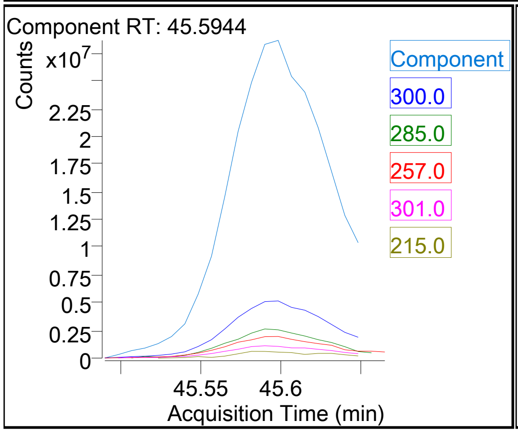

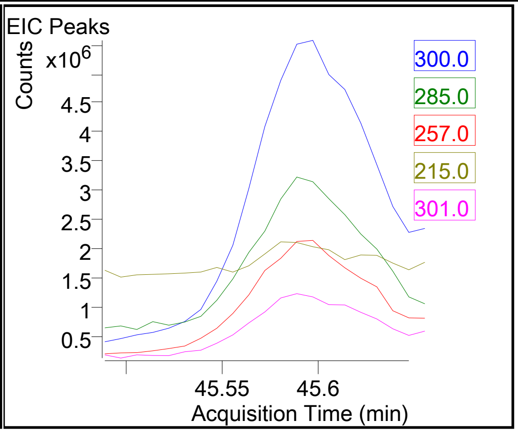

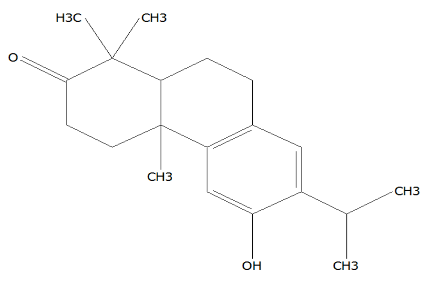

Generated at 12:24 PM on 2/25/2021

Component RT	Compound Name	Component Area	Match Factor	CAS#	Formula	Estimated Conc.
45.7139	(.+/-.)-Demethylsalvicanol	1248028356.4	96.1	177019-45-3	C20H30O3	

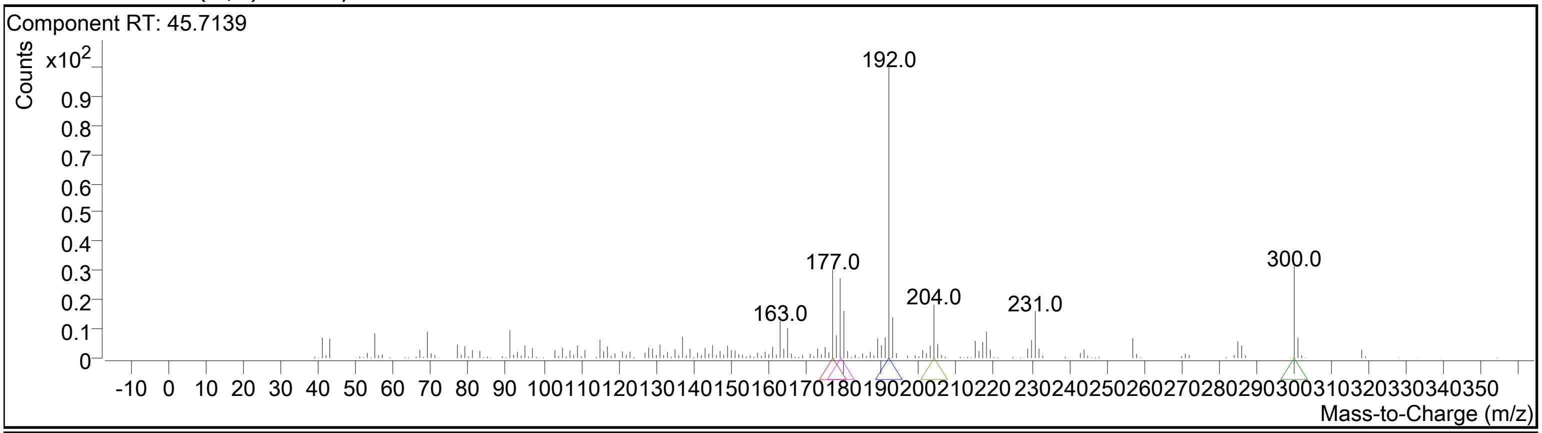

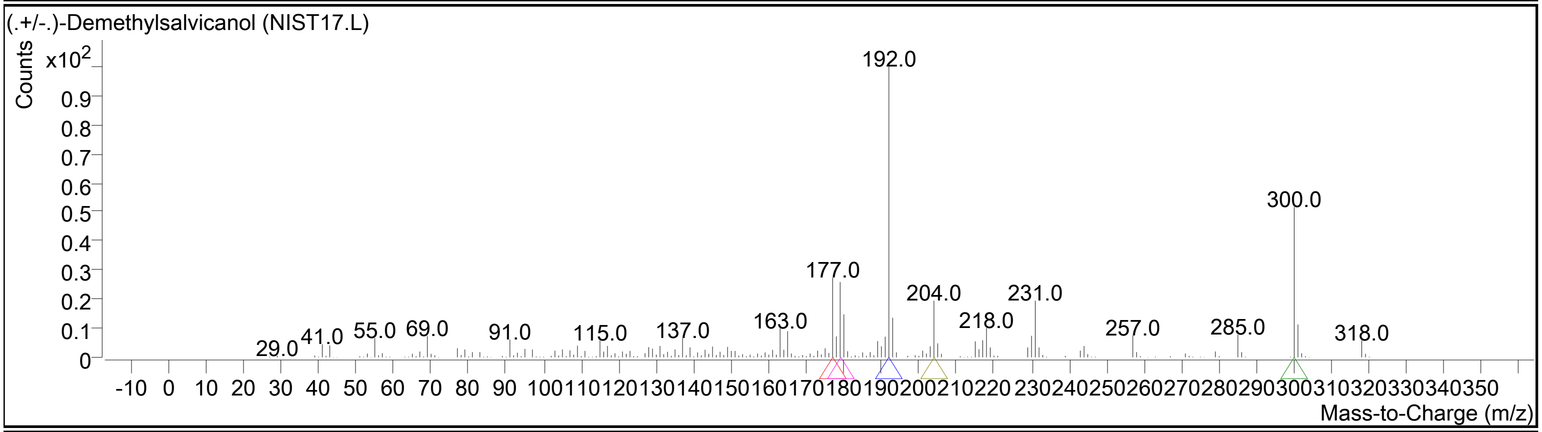

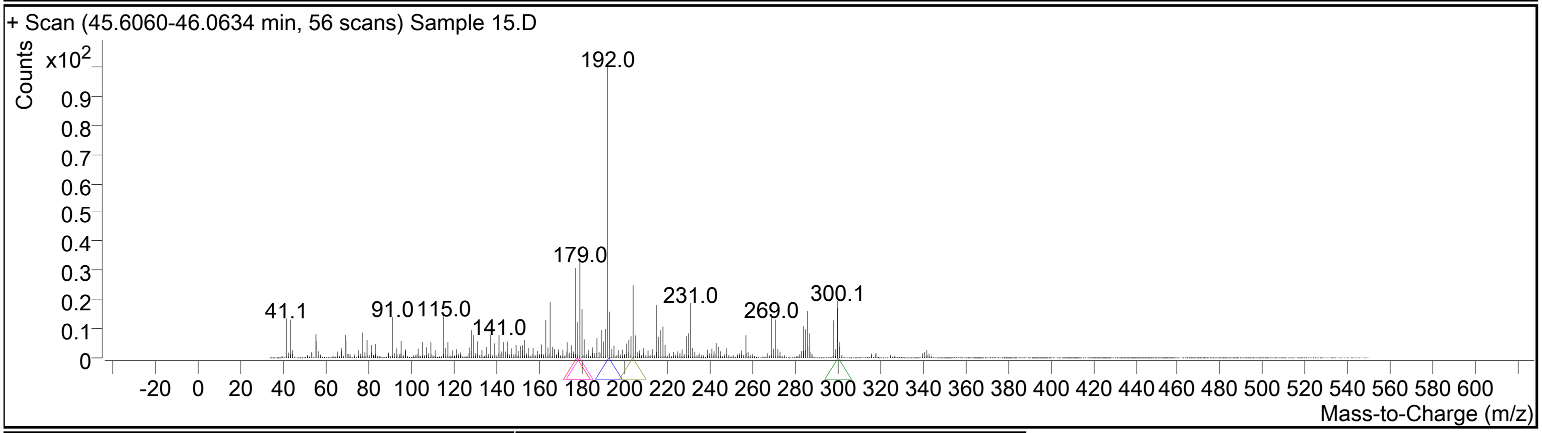

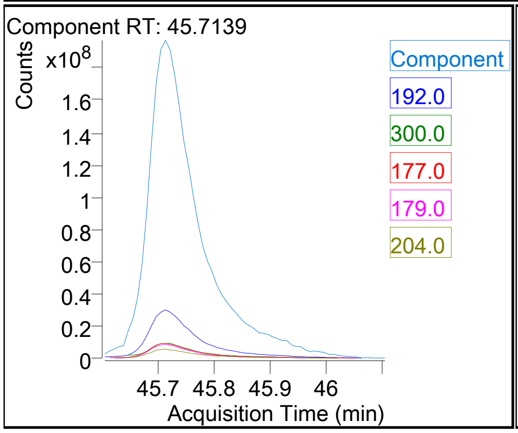

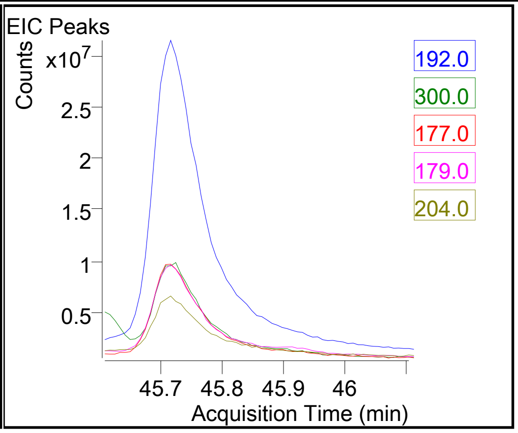

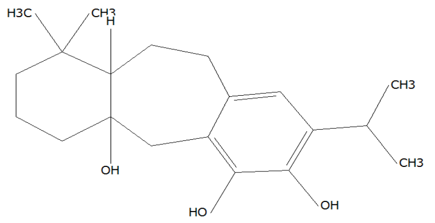

Generated at 12:24 PM on 2/25/2021

Component RT	Compound Name	Component Area	Match Factor	CAS#	Formula	Estimated Conc.
45.9197	Propenoic acid, 2-cyano-3-(1-methyl-5-phenylthio-2-pyrrolyl)-, methyl ester	41650451.7	73.0	94008-41-0	C16H14N2O2S	

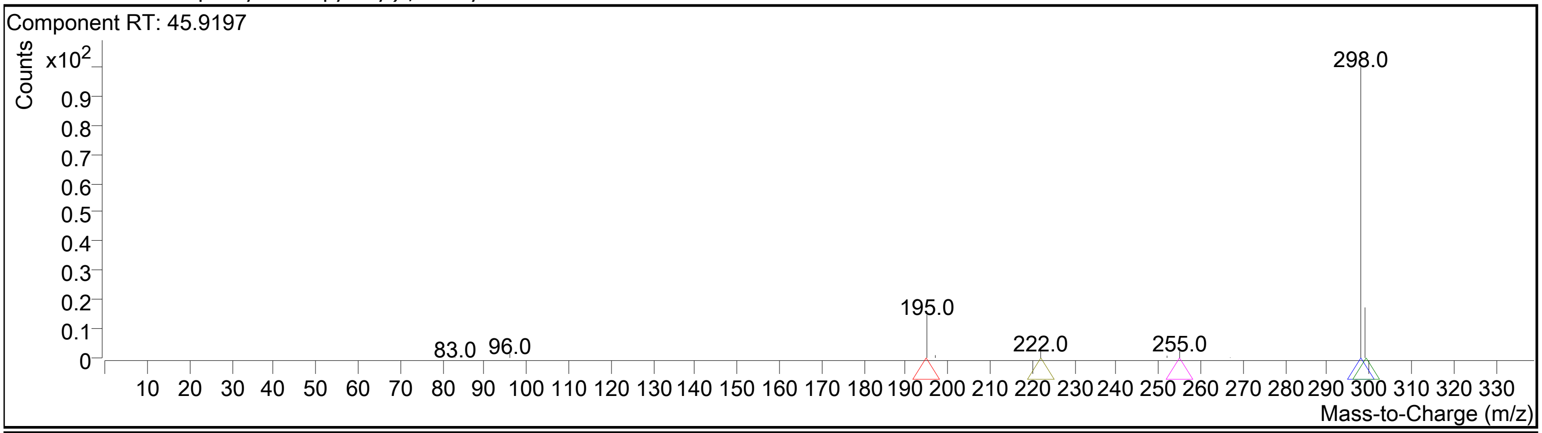

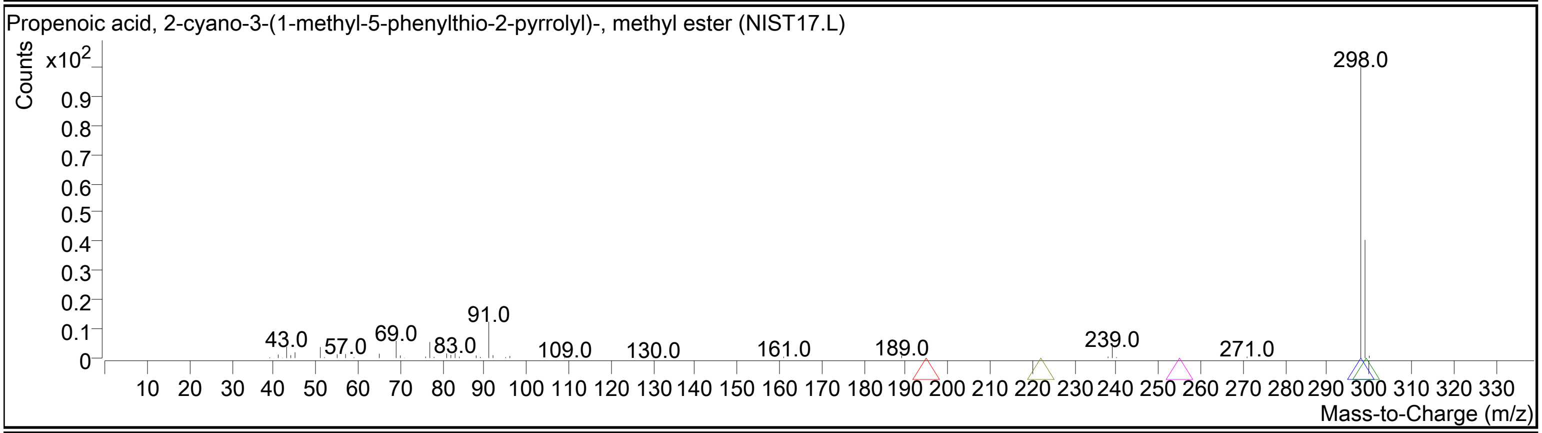

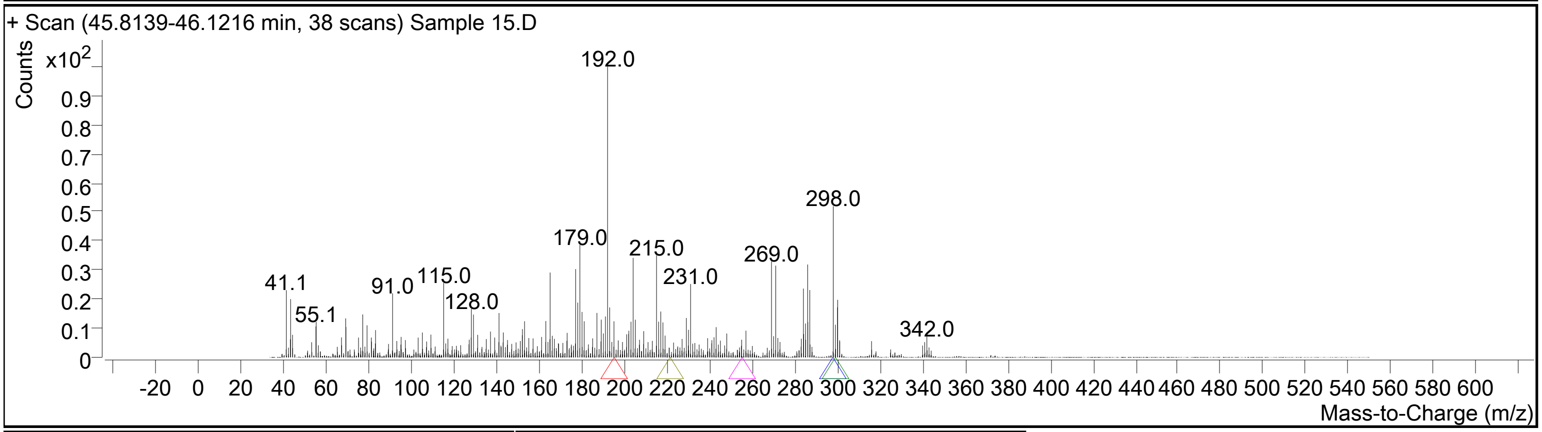

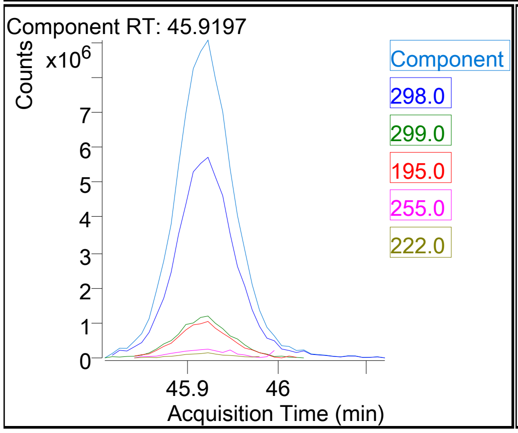

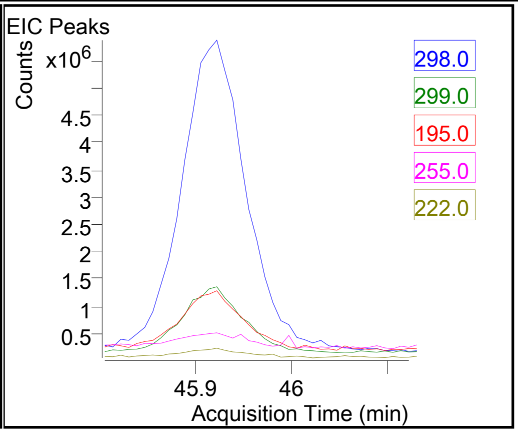

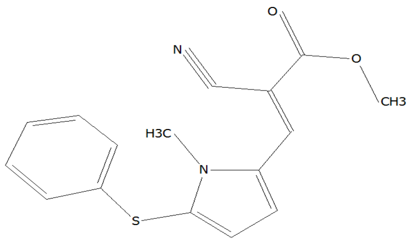

Component RT	Compound Name	Component Area	Match Factor	CAS#	Formula	Estimated Conc.
45.9872	5,6,4'-Trihydroxy-7,8-dimethoxyflavone	1936142.4	70.2	76844-66-1	C17H14O7	

Component RT: 45.9872

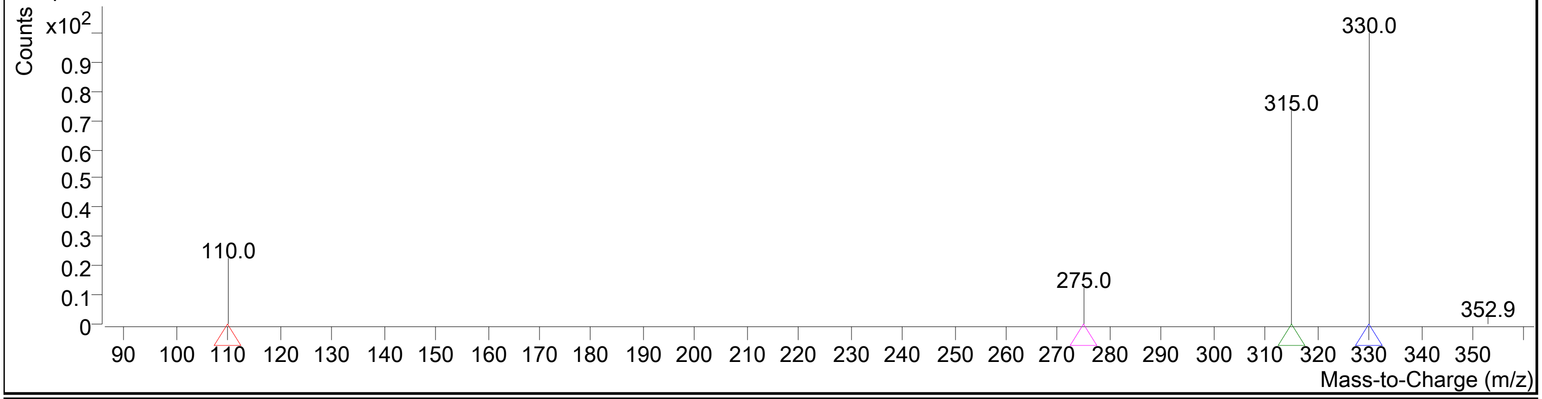

5,6,4'-Trihydroxy-7,8-dimethoxyflavone (NIST17.L)

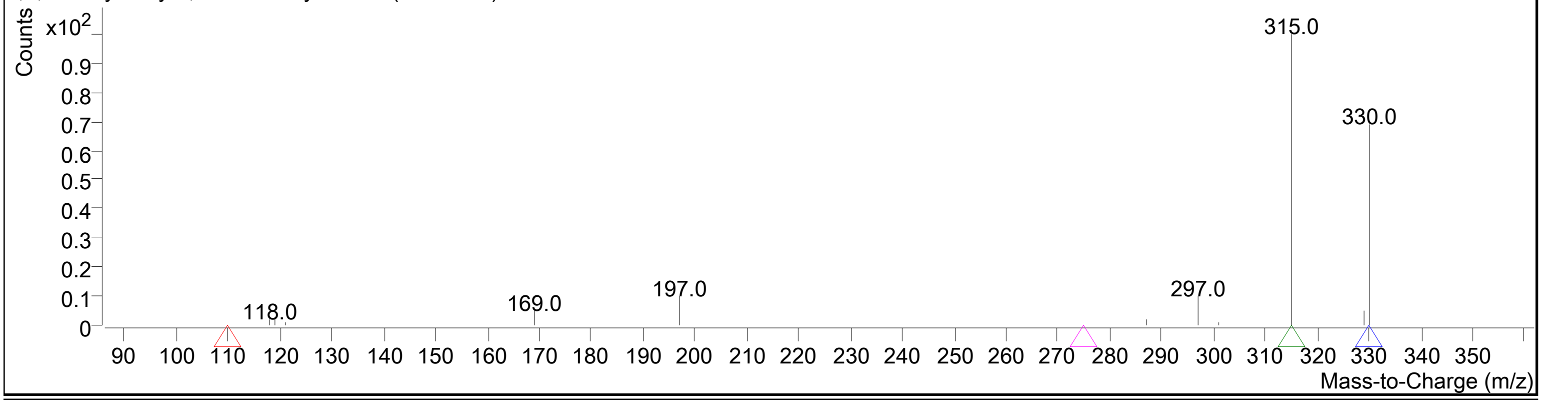

+ Scan (45.9386-46.0627 min, 15 scans) Sample 15.D

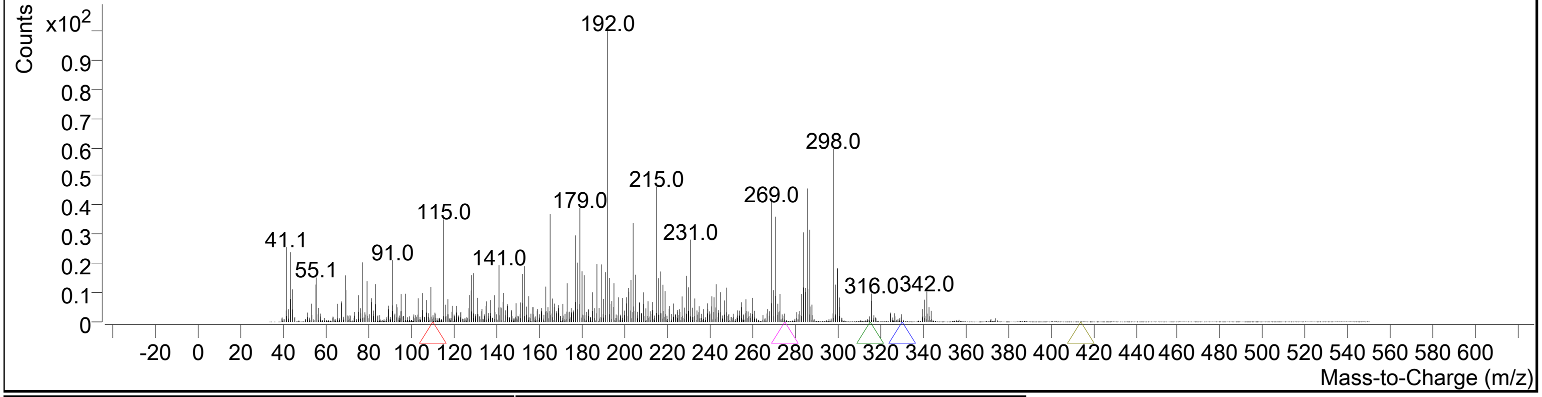

Component RT: 45.9872

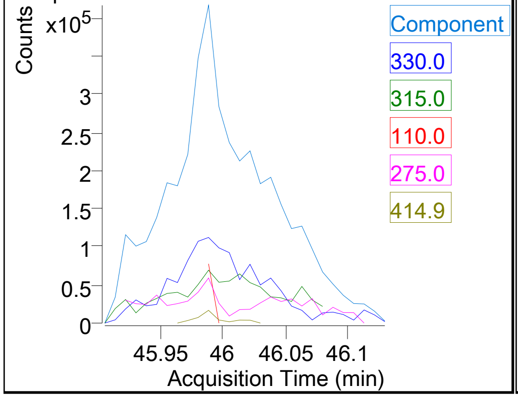

EIC Peaks

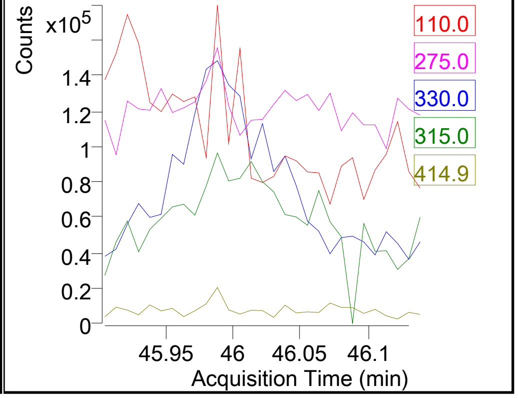

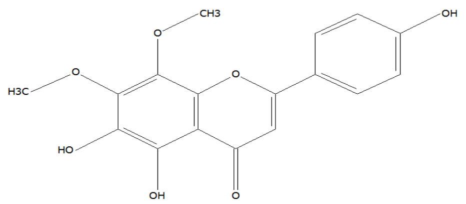

Generated at 12:24 PM on 2/25/2021

 Agilent Technologies

Component RT	Compound Name	Component Area	Match Factor	CAS#	Formula	Estimated Conc.
47.4100	Chroman-4-one, 2,3-dehydro-7-hydroxy-2-methyl-3-(2-pyridyl)-	5350379.7	65.5	65047-28-1	C15H11NO3	

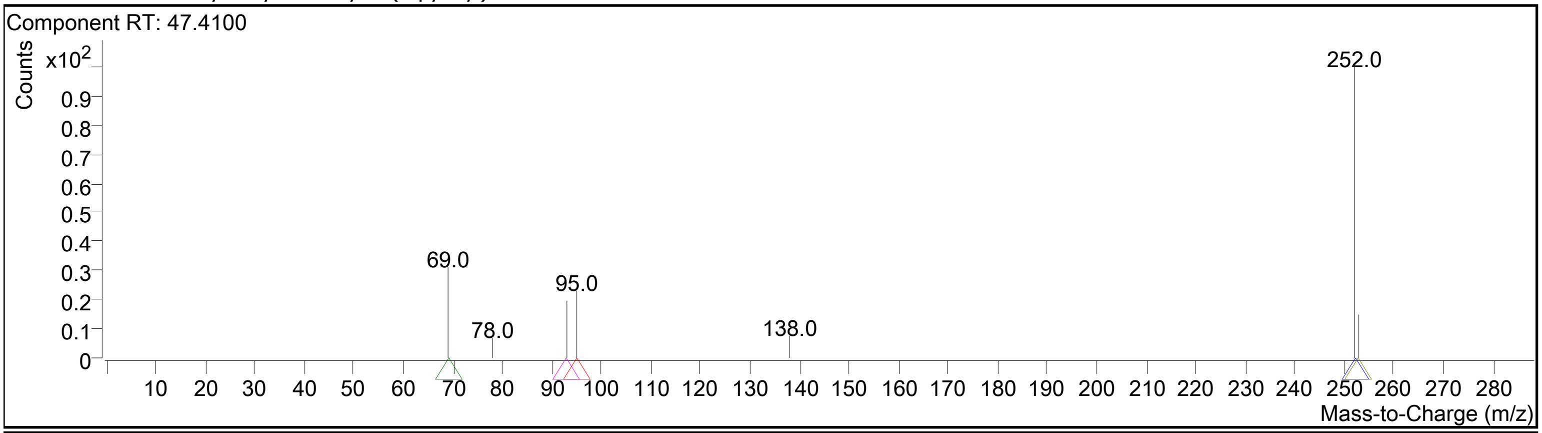

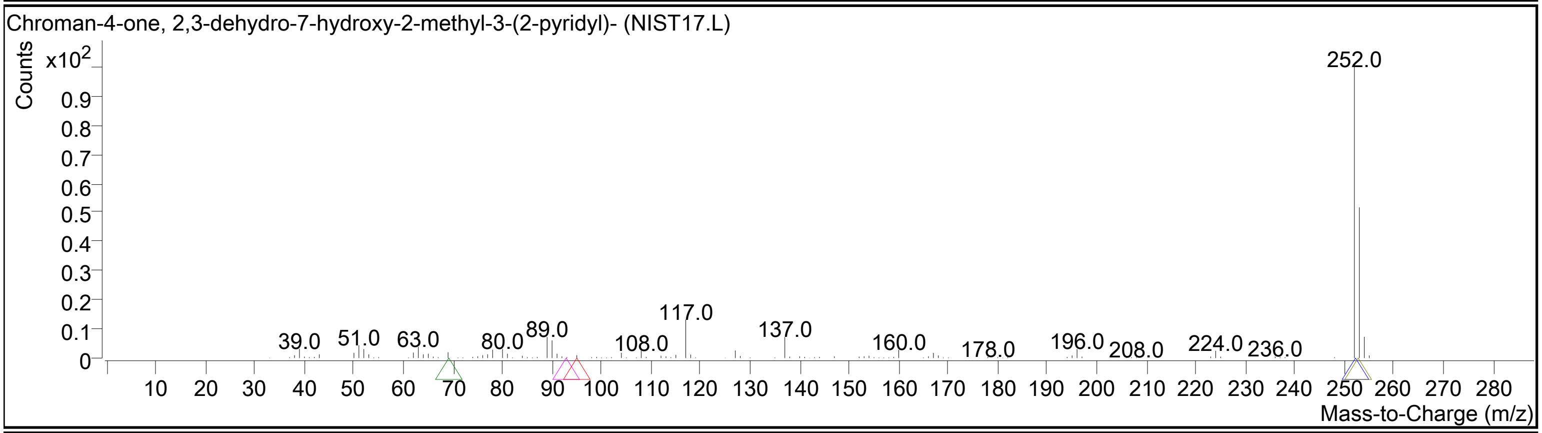

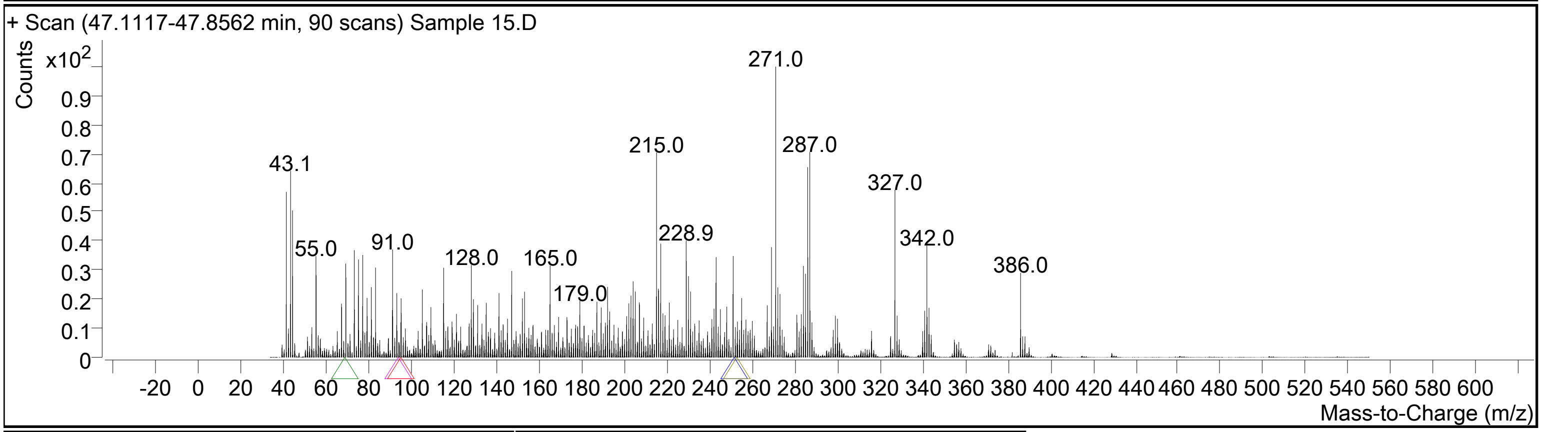

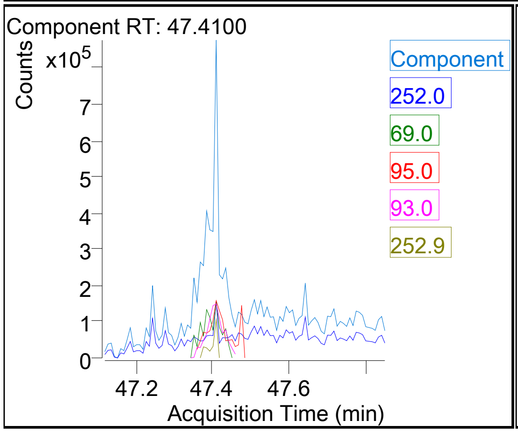

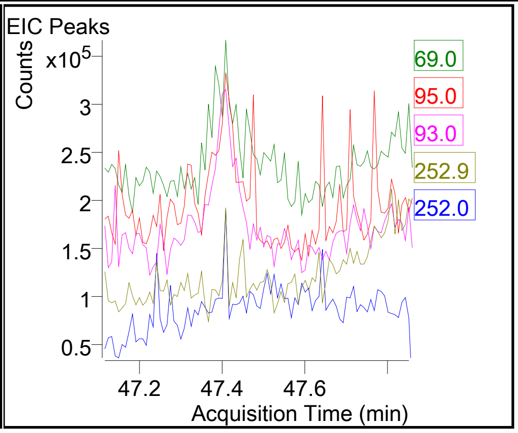

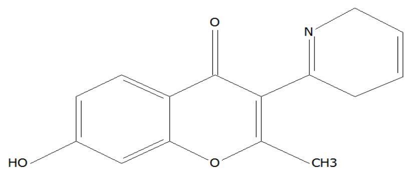

Generated at 12:24 PM on 2/25/2021

Component RT	Compound Name	Component Area	Match Factor	CAS#	Formula	Estimated Conc.
48.0260	2,4-Difluorobenzoic acid, 2-biphenyl ester	8473089.1	73.4	1000331-58-3	C19H12F2O2	

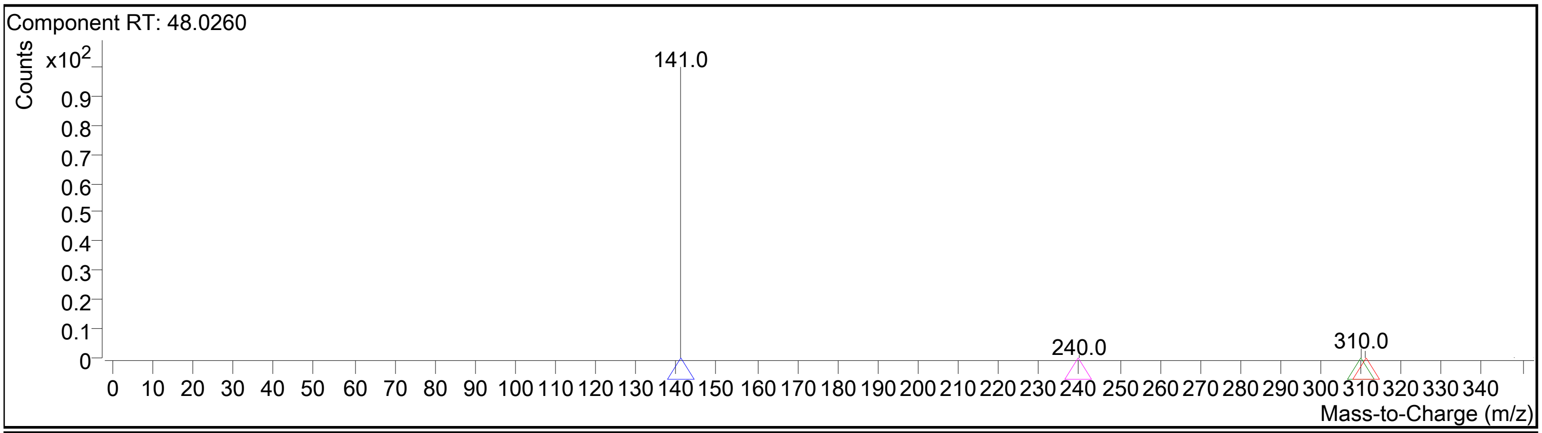

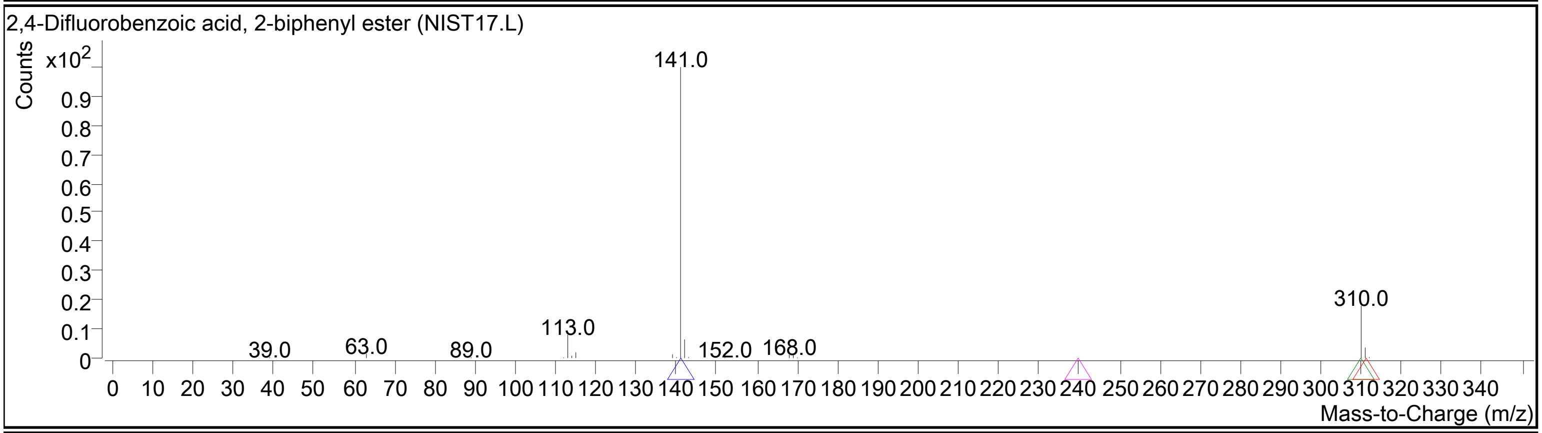

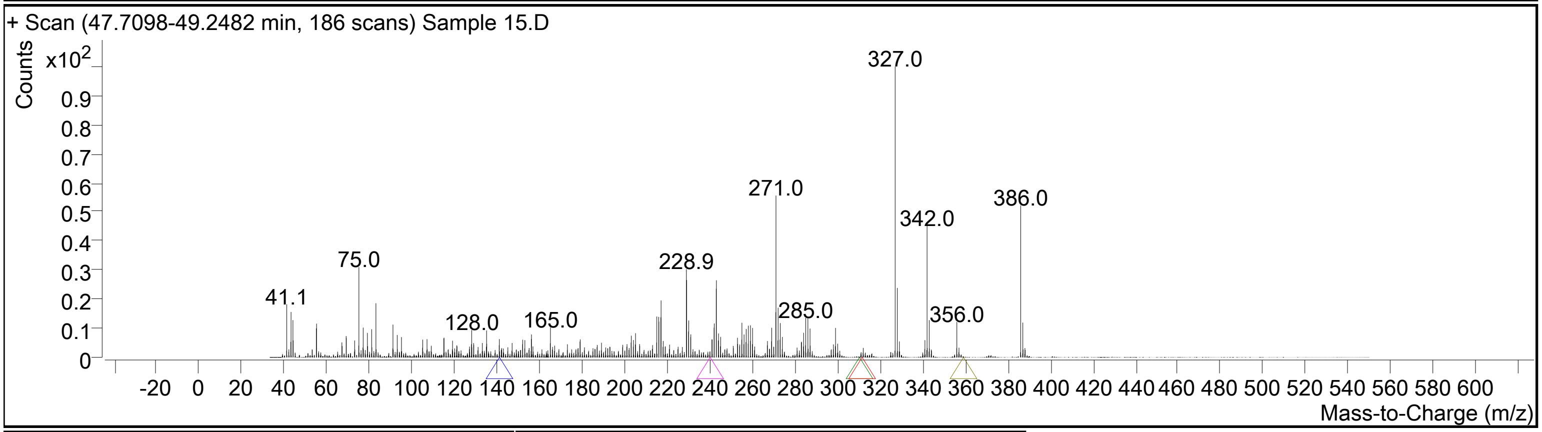

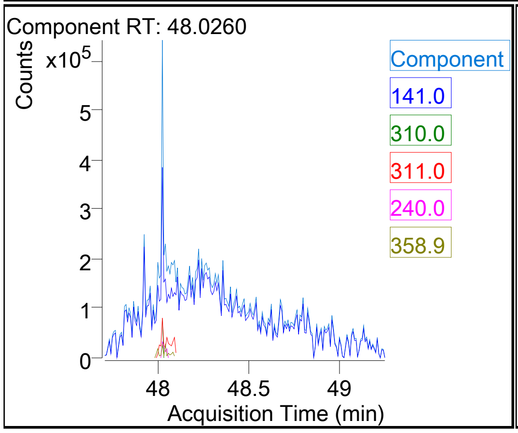

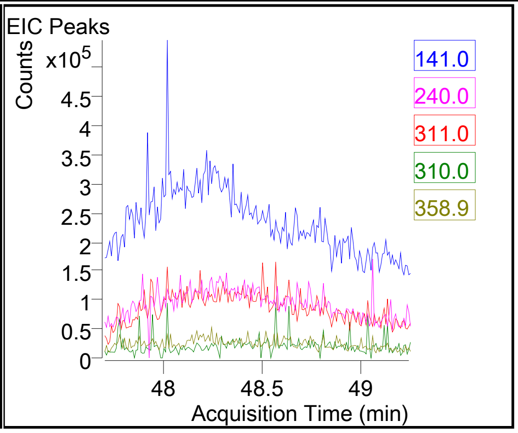

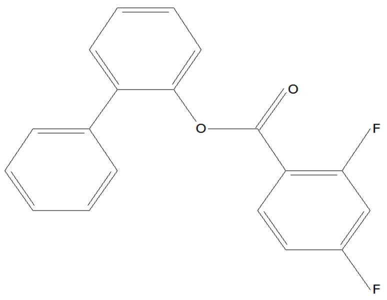

Component RT	Compound Name	Component Area	Match Factor	CAS#	Formula	Estimated Conc.
48.0841	Silane, dimethyl(2-naphthoxy)dodecyloxy-	74691550.2	73.8	1000347-21-9	C24H38O2Si	

Component RT: 48.0841

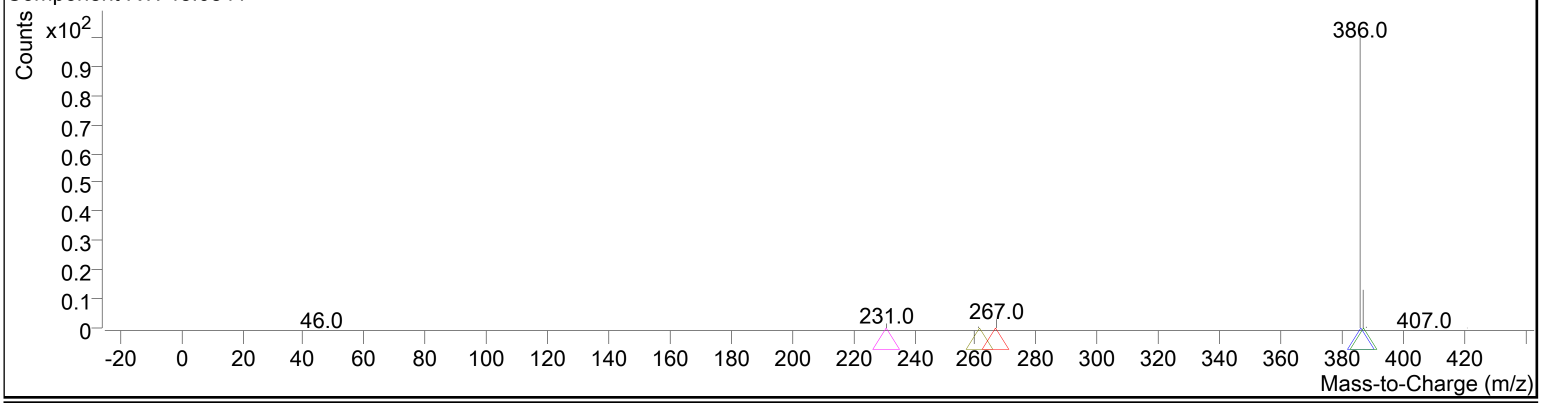

Silane, dimethyl(2-naphthoxy)dodecyloxy- (NIST17.L)

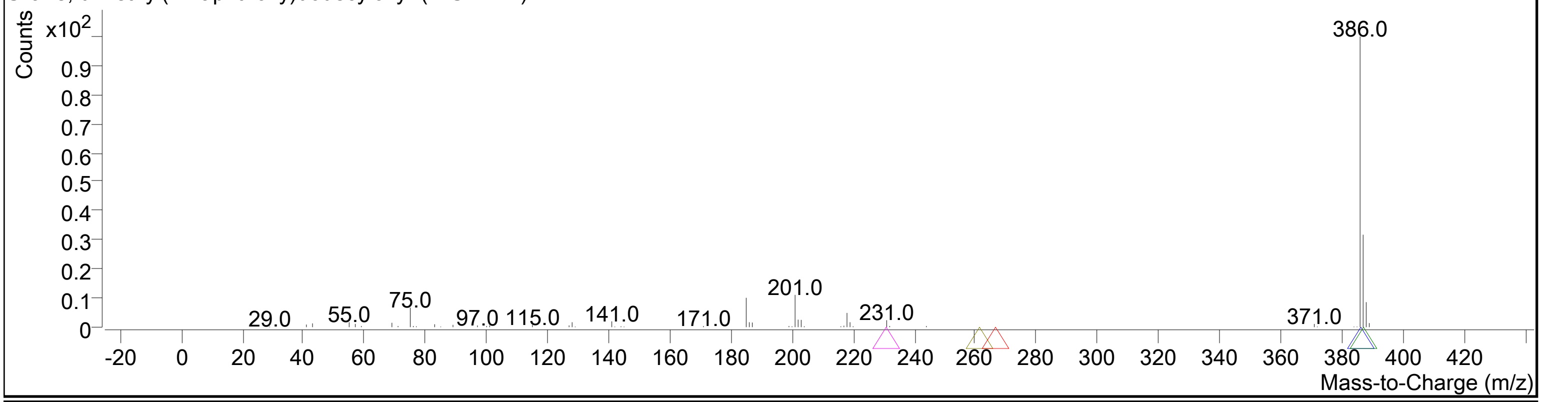

+ Scan (47.8512-48.4249 min, 69 scans) Sample 15.D

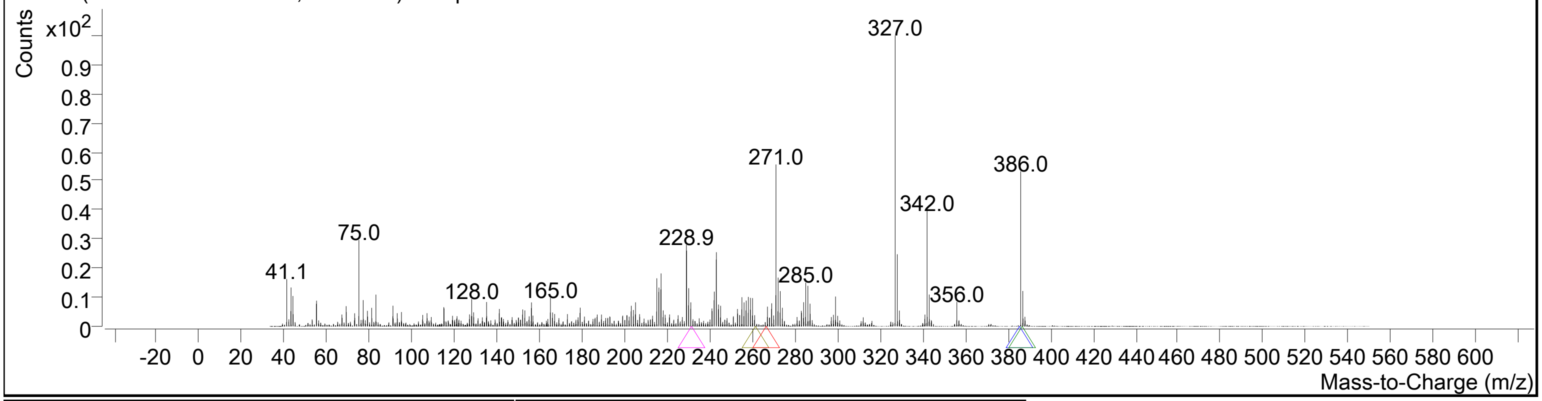

Component RT: 48.0841

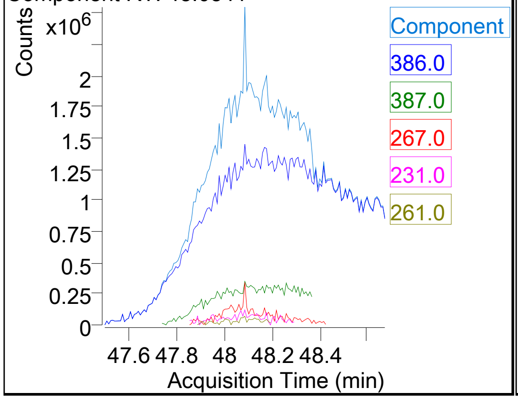

EIC Peaks

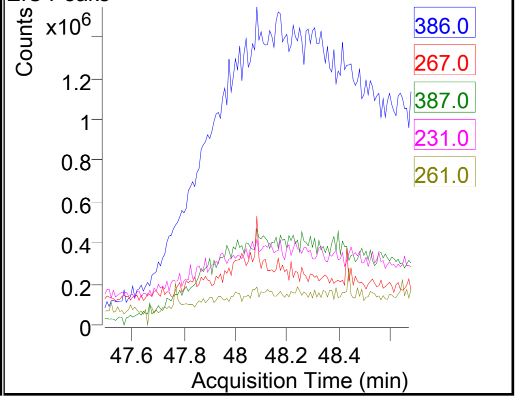

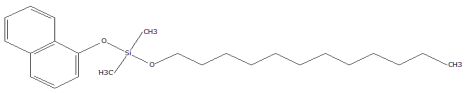

Component RT	Compound Name	Component Area	Match Factor	CAS#	Formula	Estimated Conc.
48.1373	tert-Butyldimethylsilanol	49745965.6	69.5	18173-64-3	C6H16OSi	

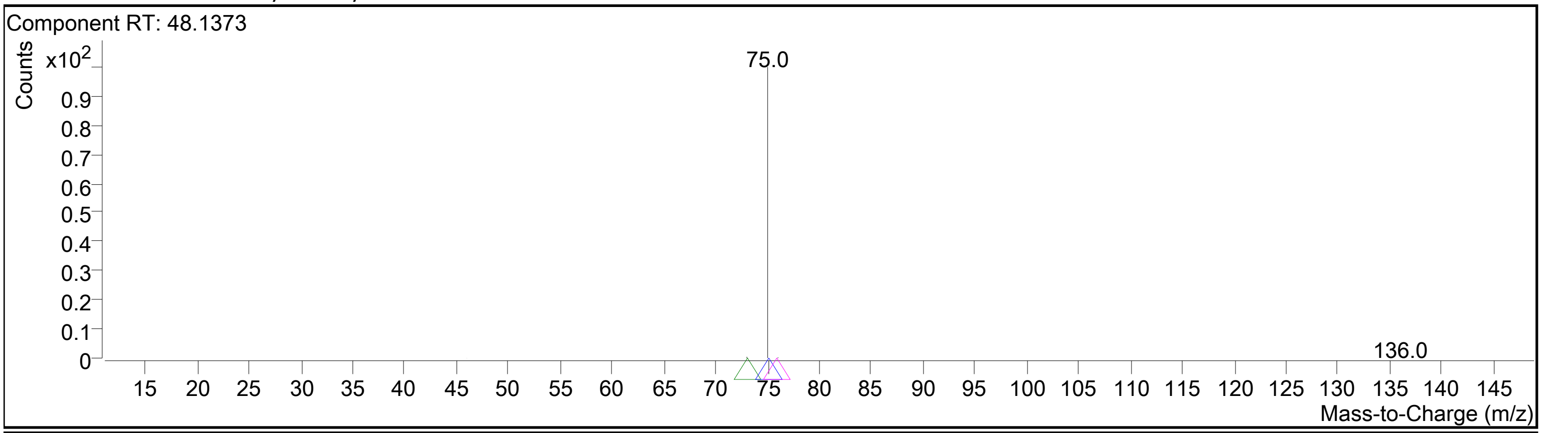

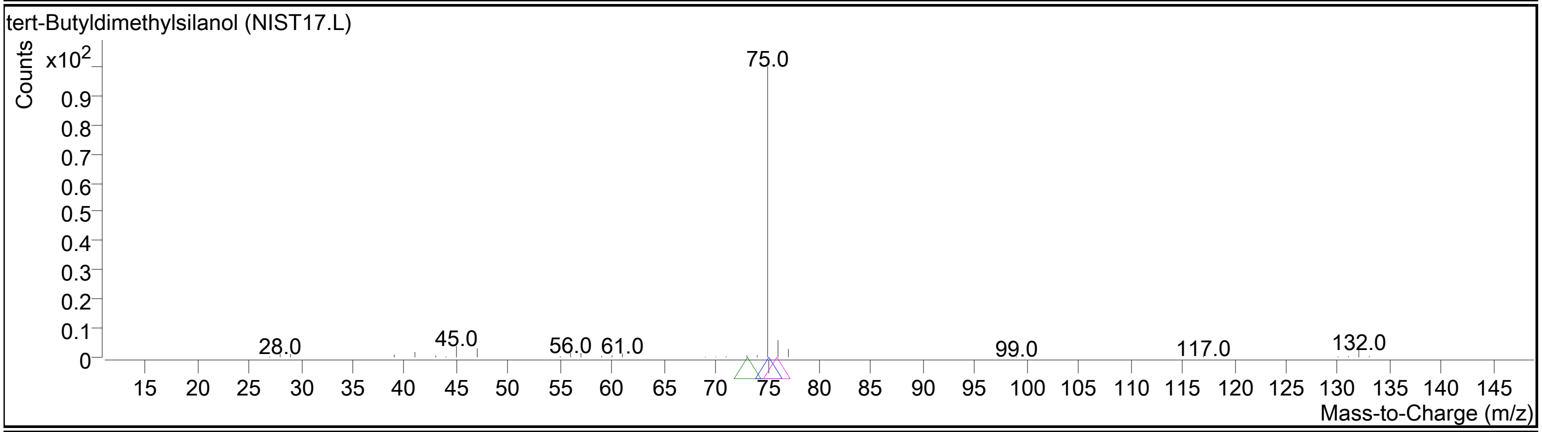

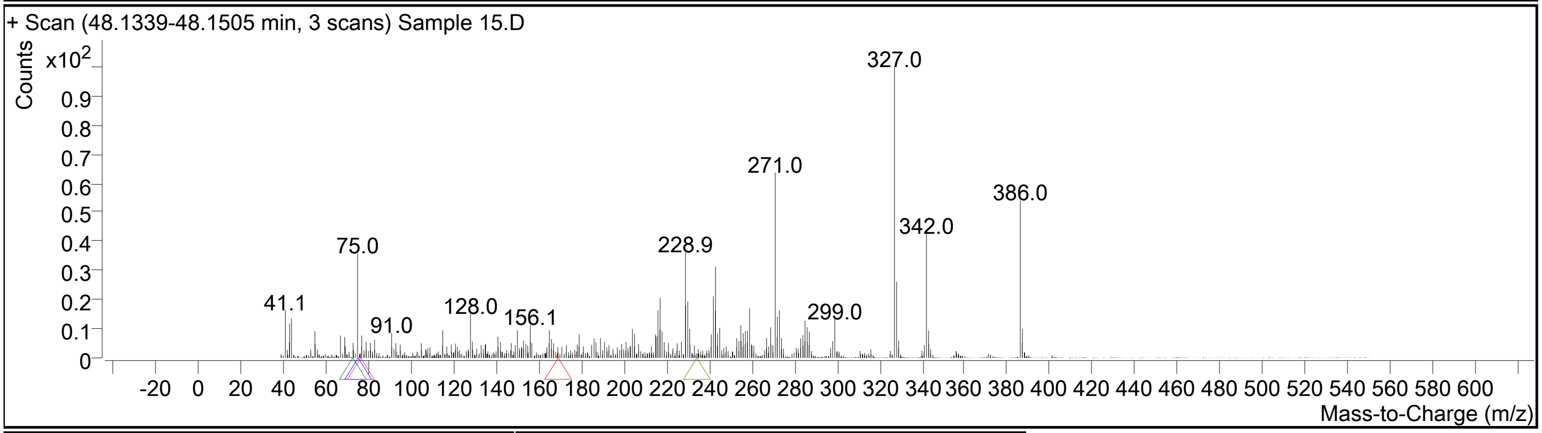

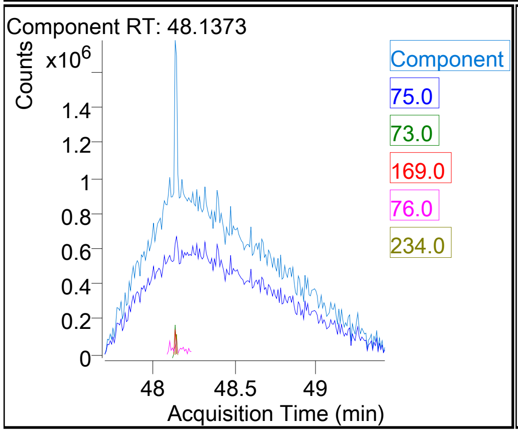

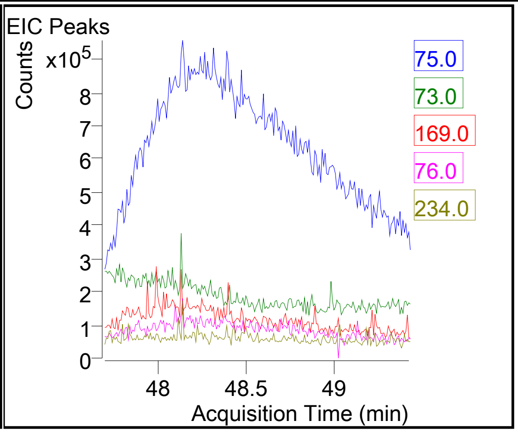

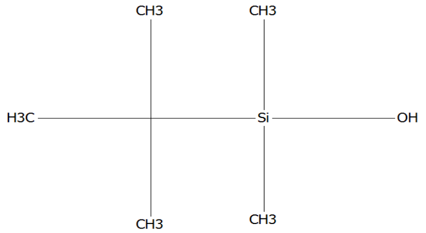

Generated at 12:24 PM on 2/25/2021

Component RT	Compound Name	Component Area	Match Factor	CAS#	Formula	Estimated Conc.
48.7676	Androst-5-en-7-one, 3-(acetyloxy)-, (3.beta.)-	132522809.7	69.8	25845-92-5	C21H30O3	

Component RT: 48.7676

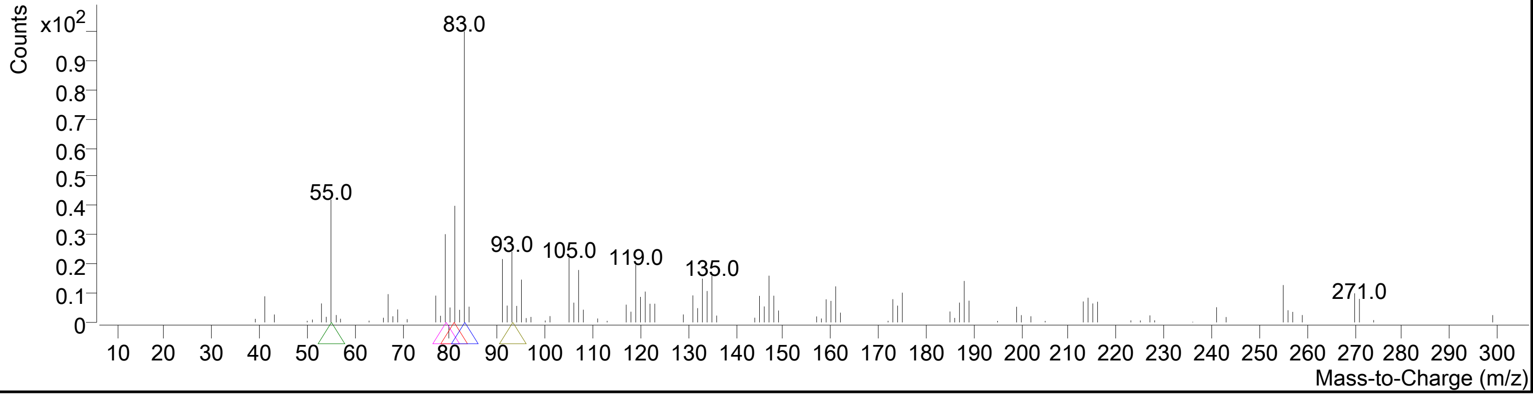

Androst-5-en-7-one, 3-(acetyloxy)-, (3.beta.)- (NIST17.L)

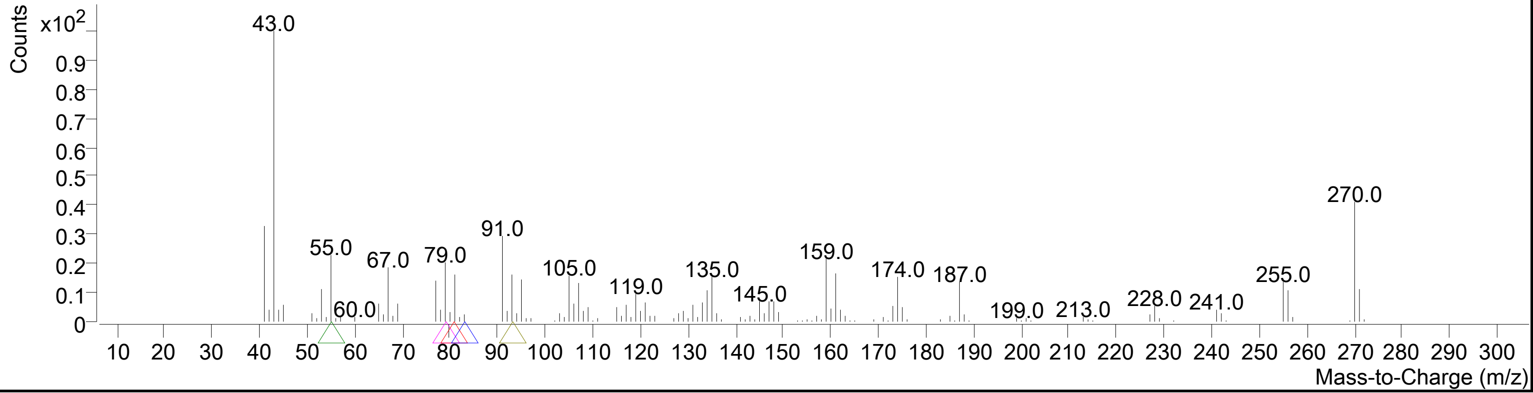

+ Scan (48.6827-48.8823 min, 25 scans) Sample 15.D

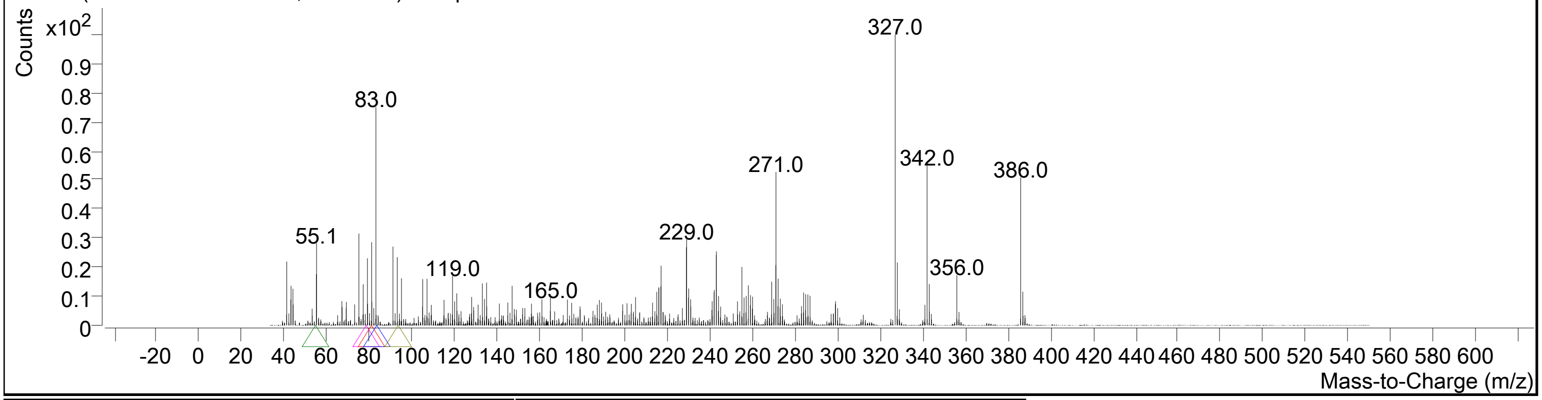

Component RT: 48.7676

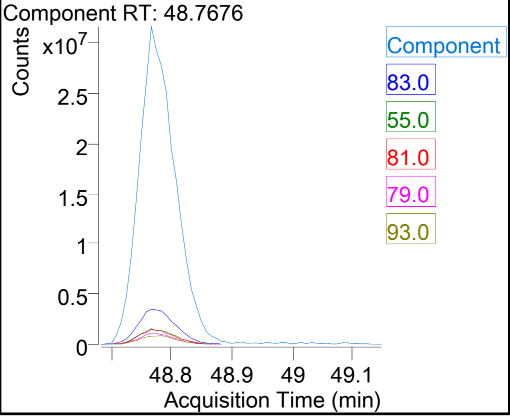

EIC Peaks

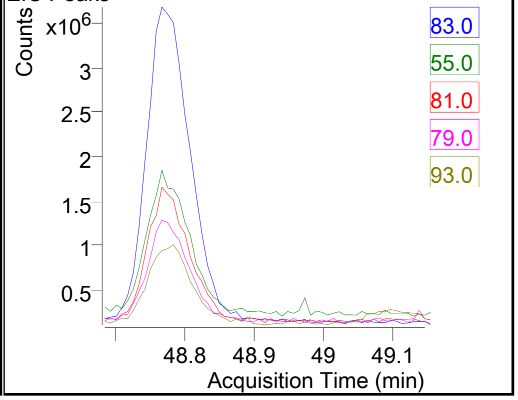

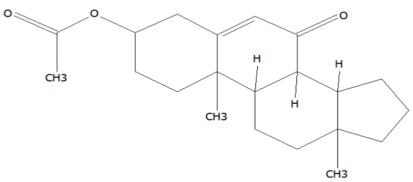

Component RT	Compound Name	Component Area	Match Factor	CAS#	Formula	Estimated Conc.
49.8853	12-O-Methylcarnosol	80971832.8	85.0	85514-27-8	C21H28O4	

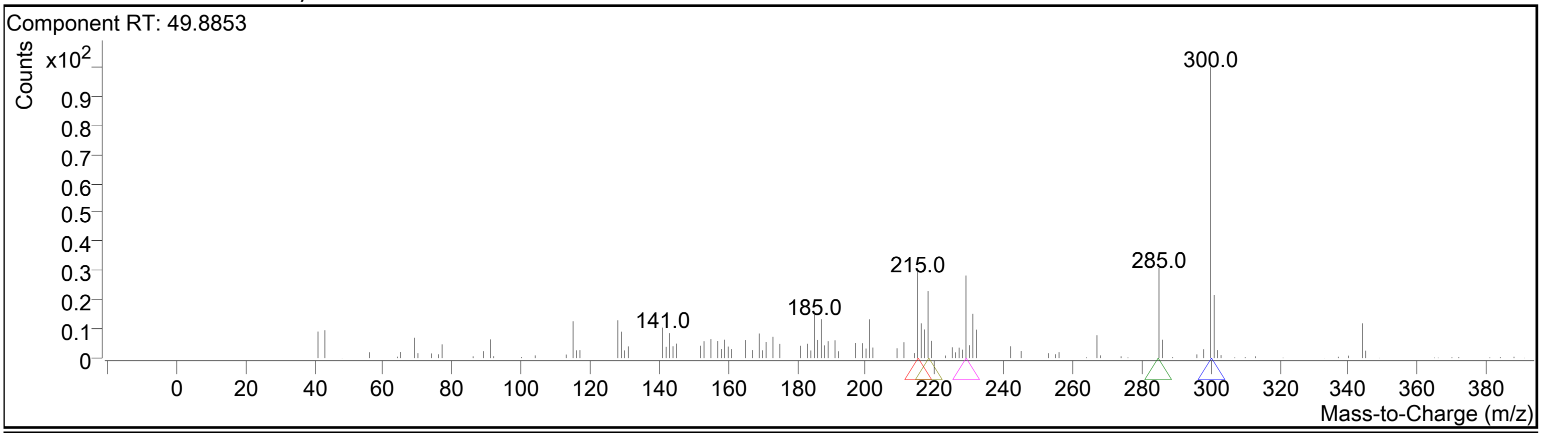

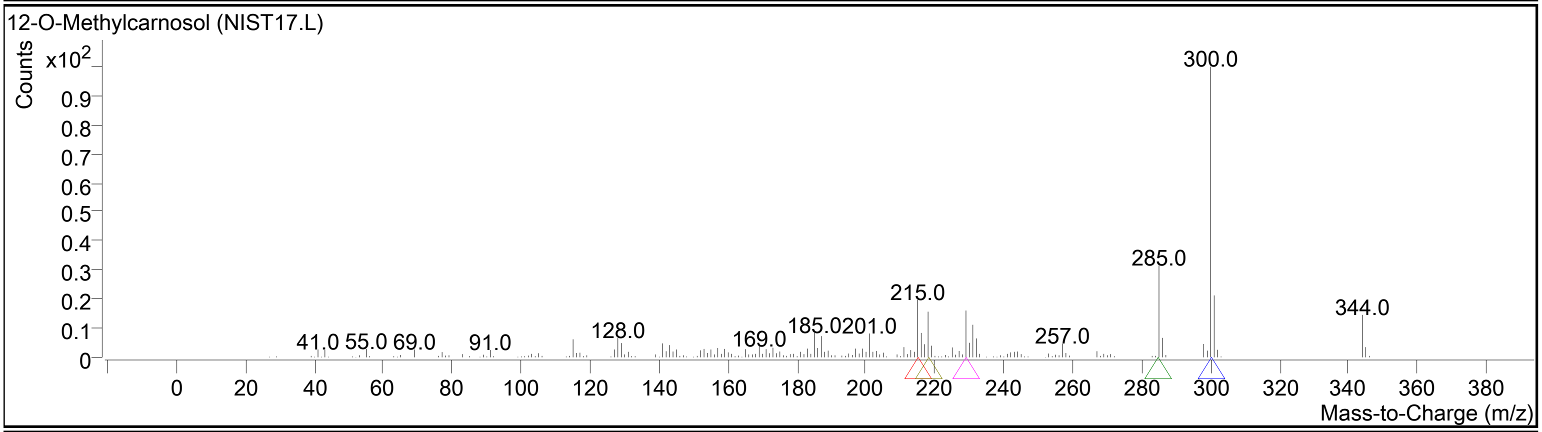

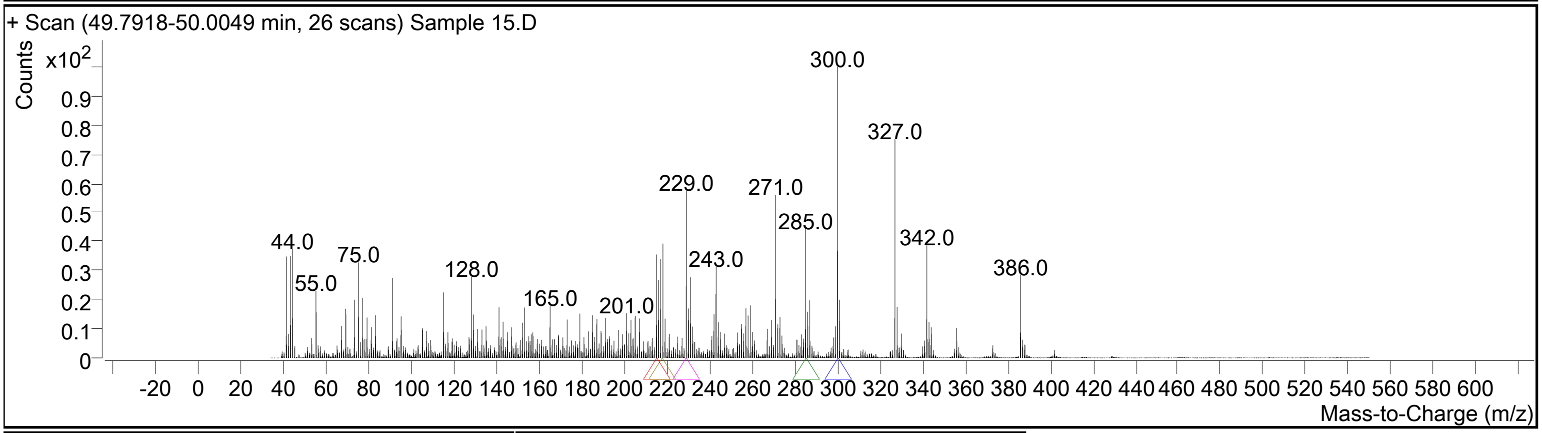

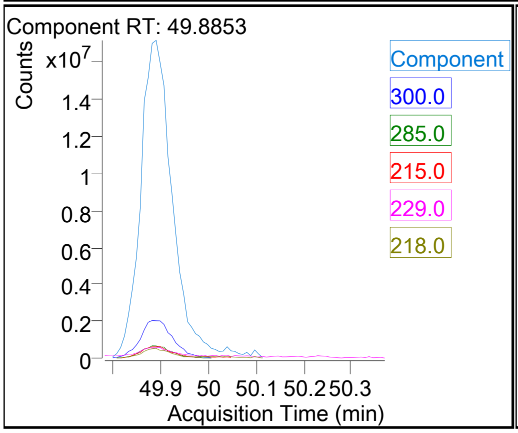

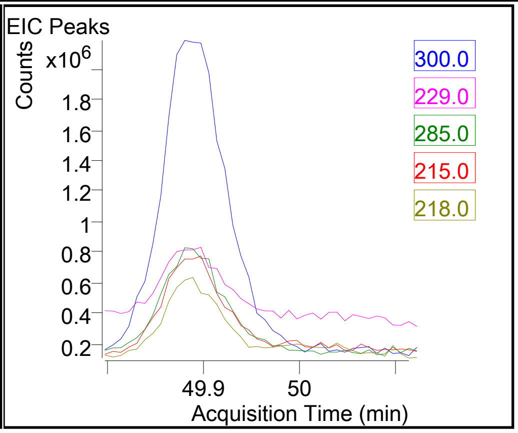

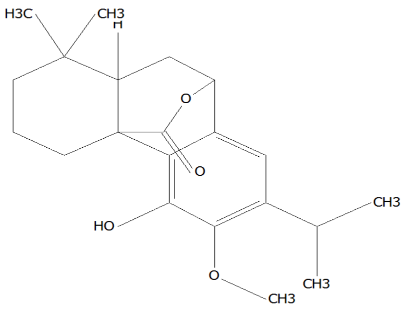

Library Search Results - NonTarget Hits with Details

Agilent Technologies

Component RT	Compound Name	Component Area	Match Factor	CAS#	Formula	Estimated Conc.
50.1294	1,4-Bis-(4-fluorophenylethynyl)-2,3,5,6-tetrafluorobenzene	1967954.4	68.0	332148-91-1	C22H8F6	

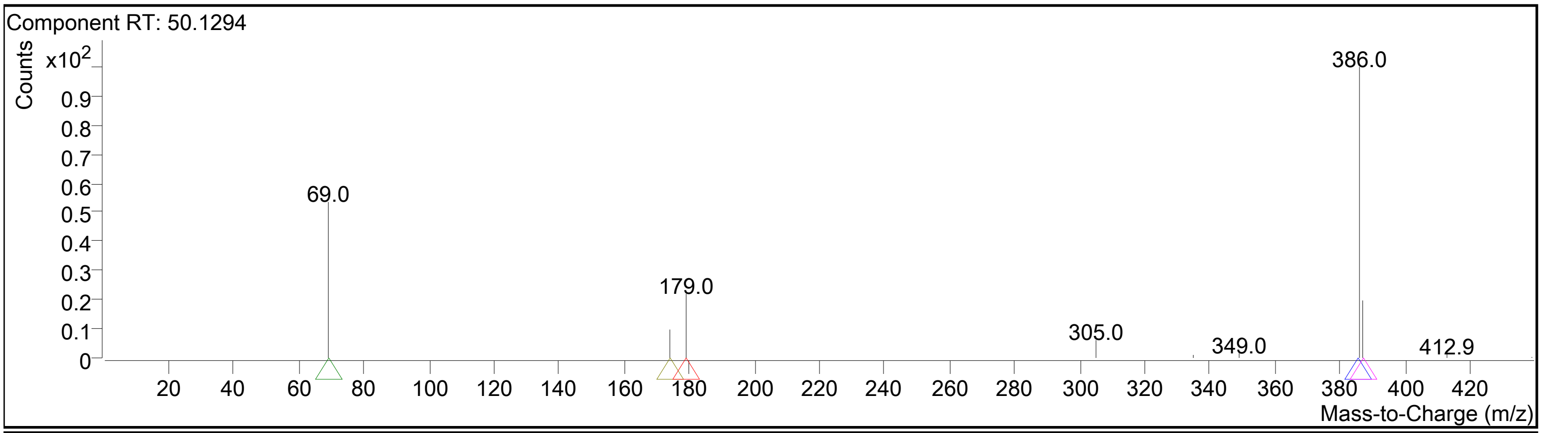

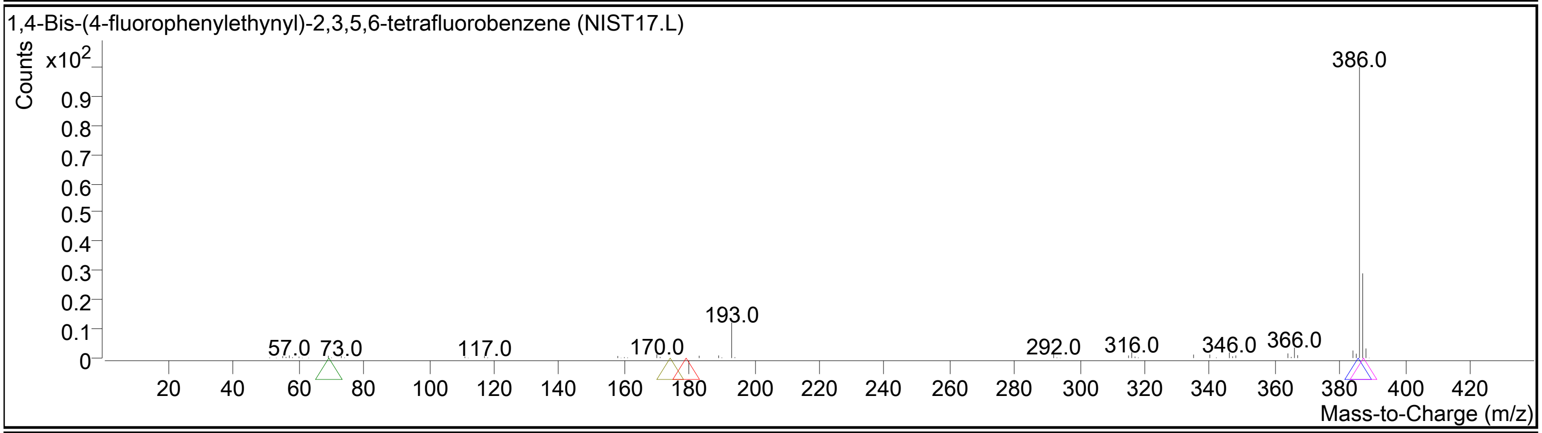

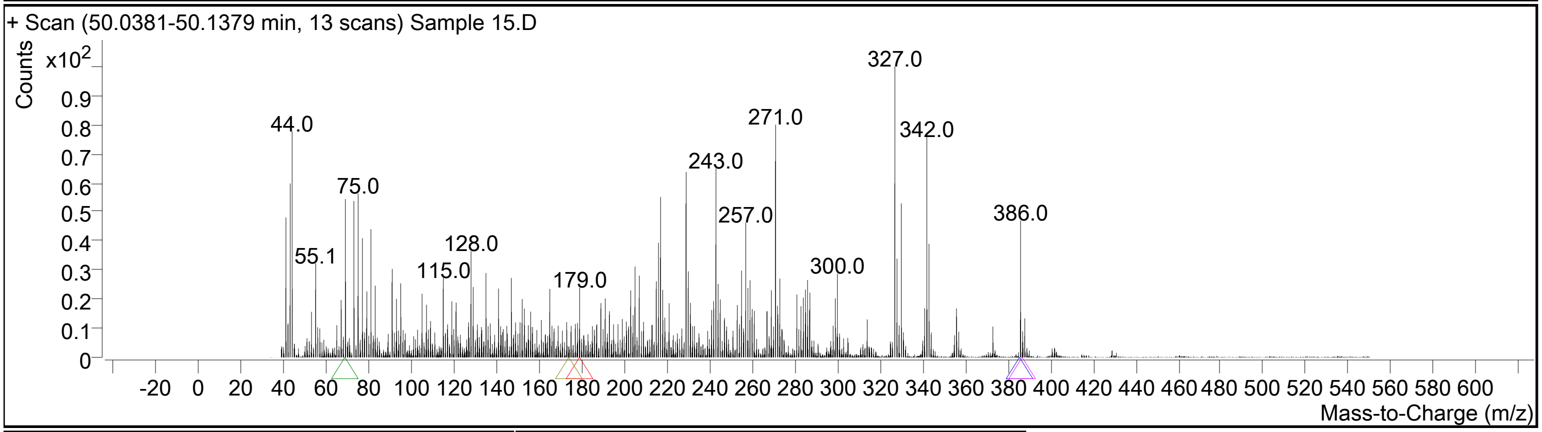

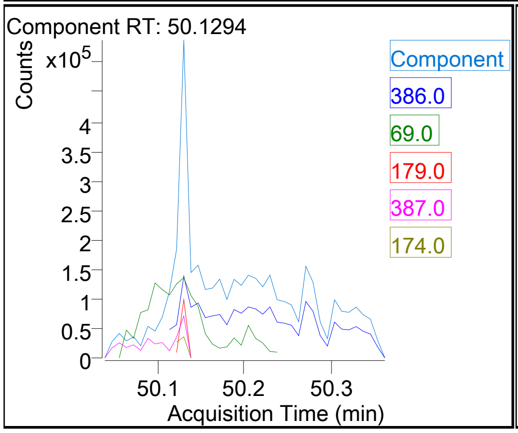

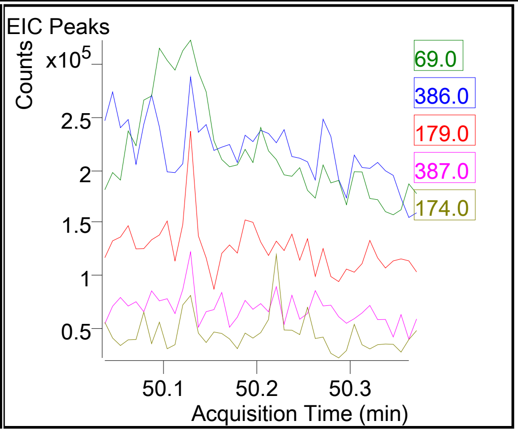

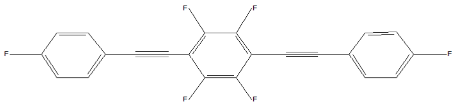

Component RT	Compound Name	Component Area	Match Factor	CAS#	Formula	Estimated Conc.
50.8649	.alpha.-Tocospiro A	124175709.1	78.7	601490-40-8	C29H50O4	

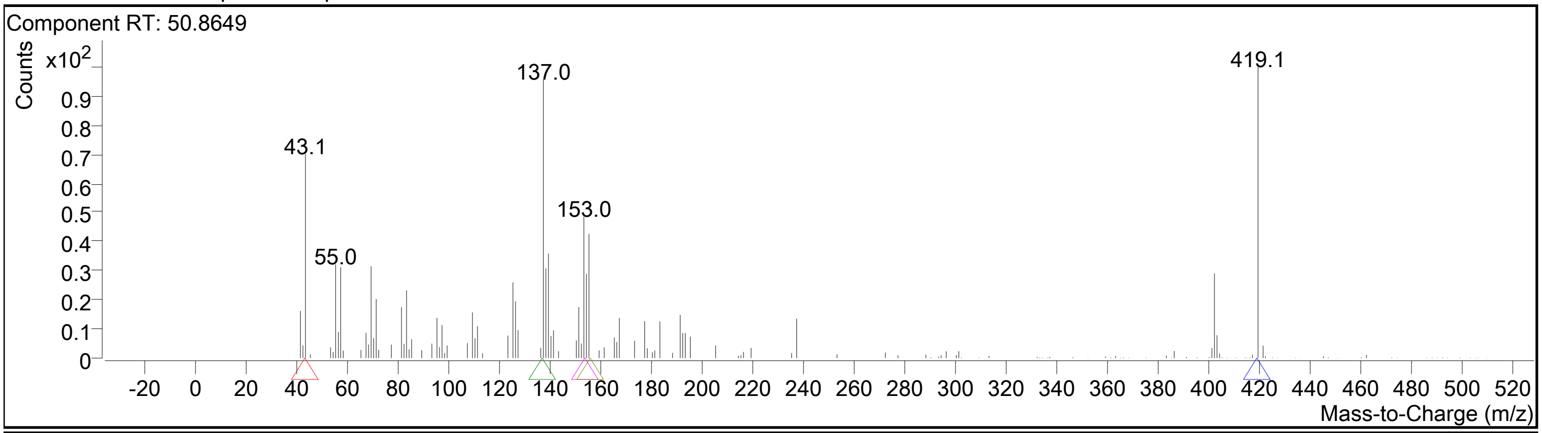

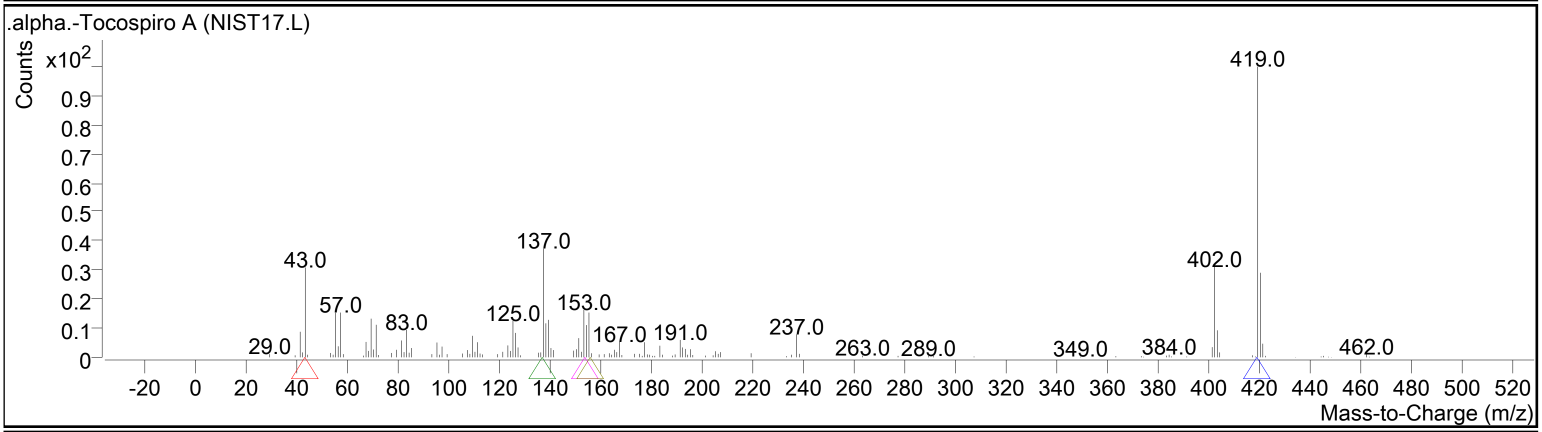

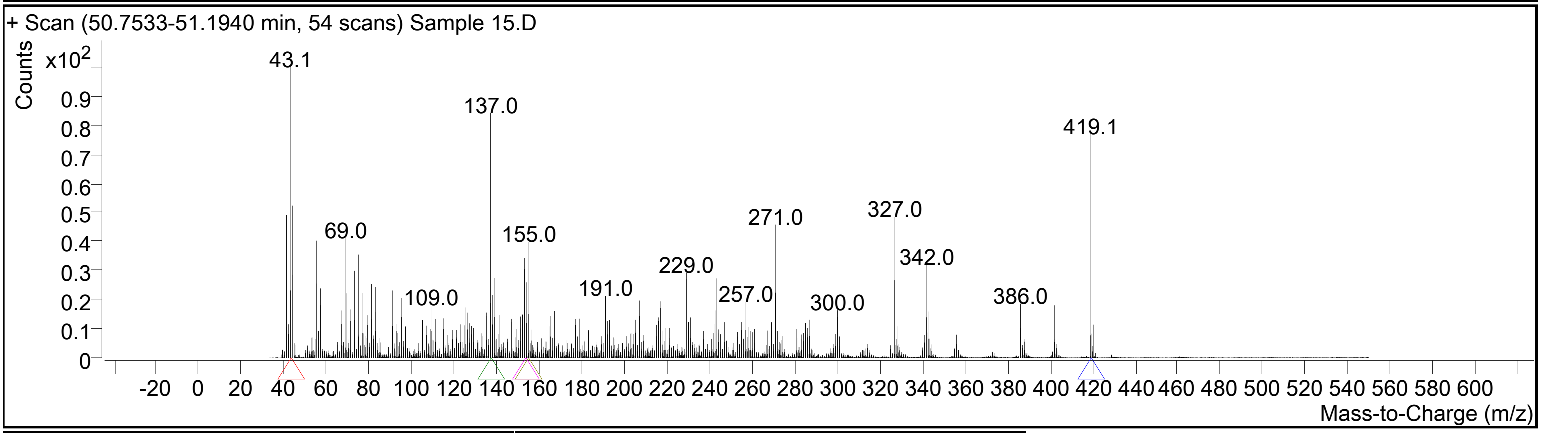

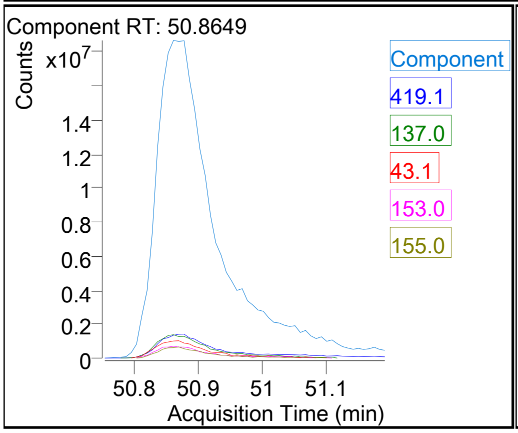

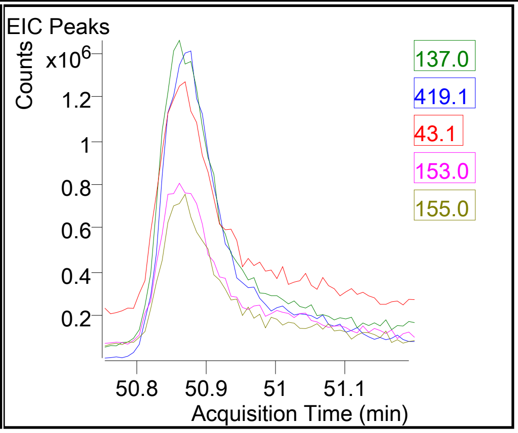

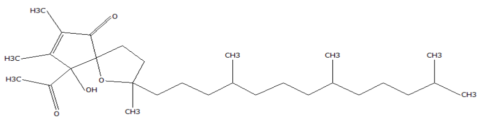

 Generated at 12:24 PM on 2/25/2021

Component RT	Compound Name	Component Area	Match Factor	CAS#	Formula	Estimated Conc.
51.2936	.alpha.-Tocospiro B	136877861.0	90.3	601490-41-9	C29H50O4	

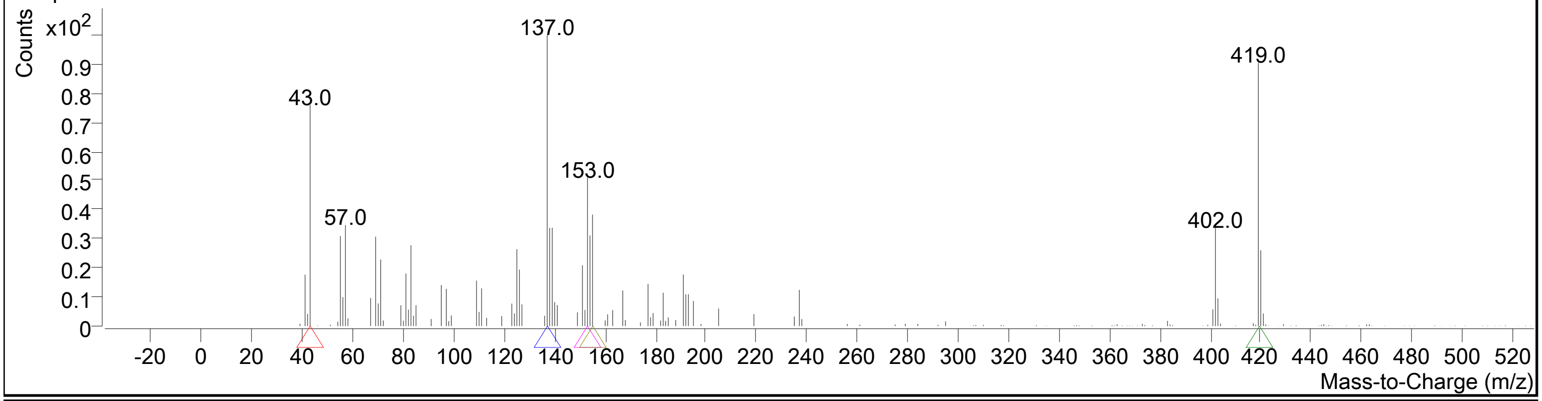

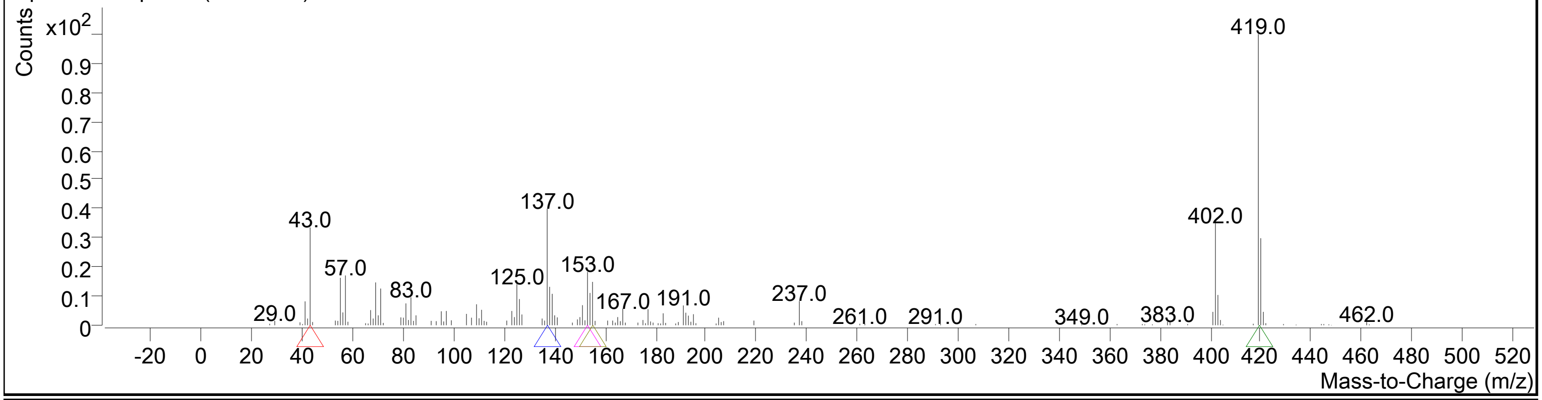

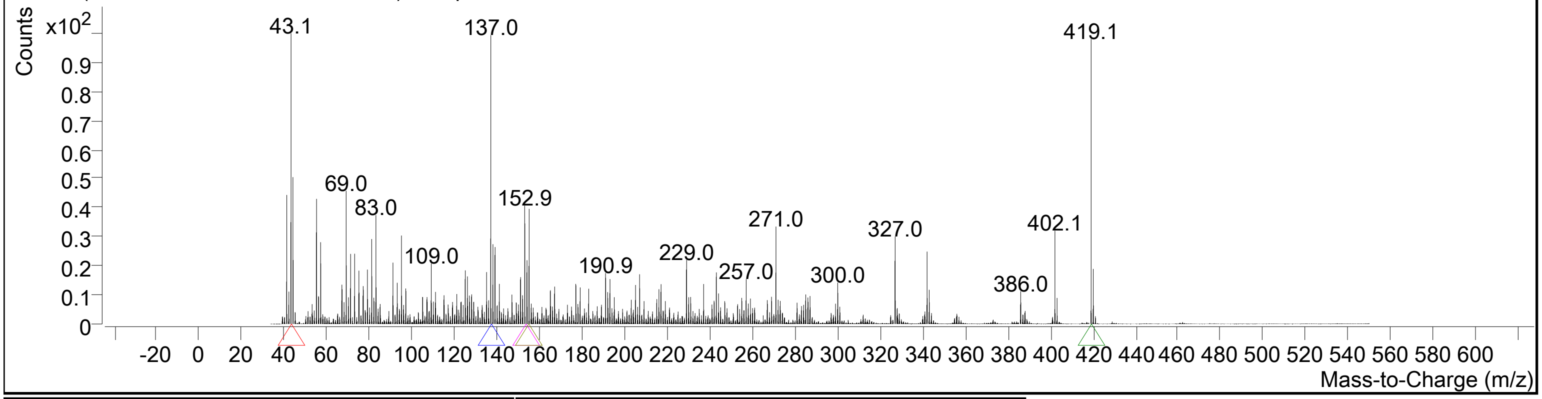

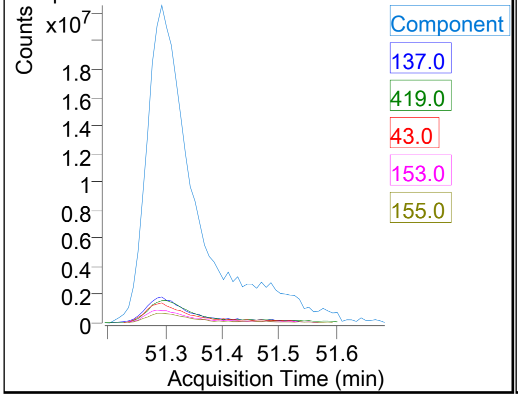

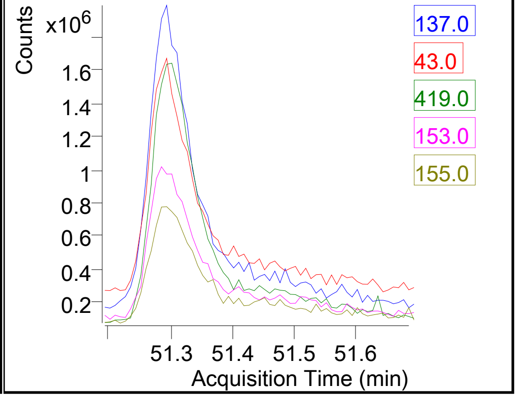

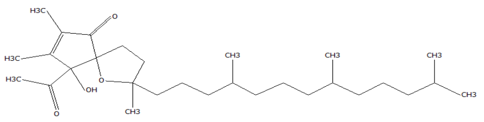

 Generated at 12:24 PM on 2/25/2021

Component RT	Compound Name	Component Area	Match Factor	CAS#	Formula	Estimated Conc.
52.0430	Oxazole, 2-[1,1'-biphenyl]-4-yl-5-phenyl-	1242429.9	72.1	852-37-9	C21H15NO	

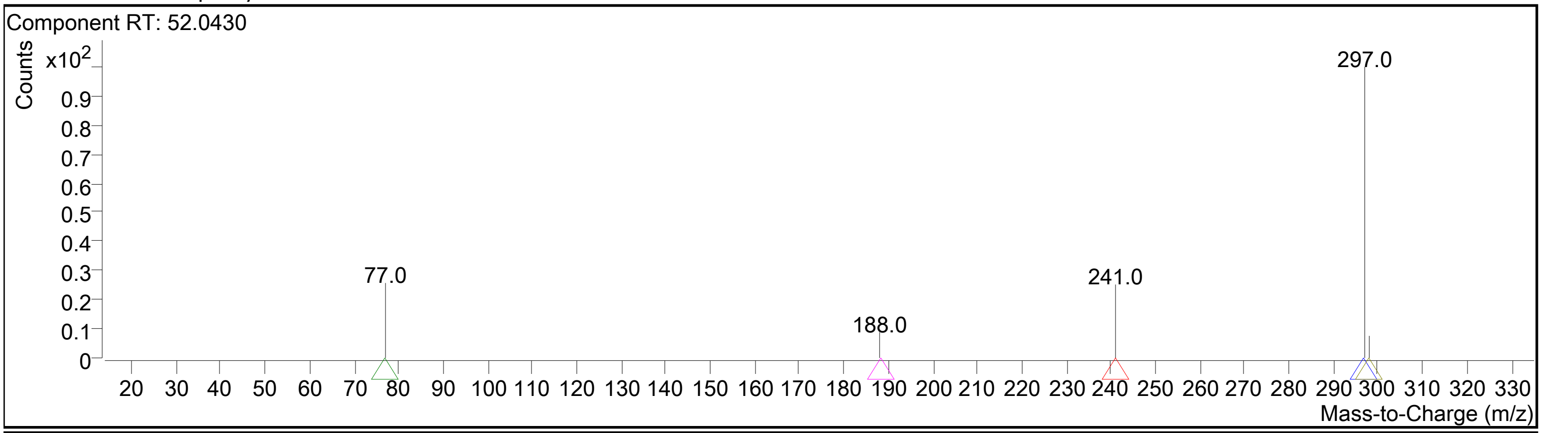

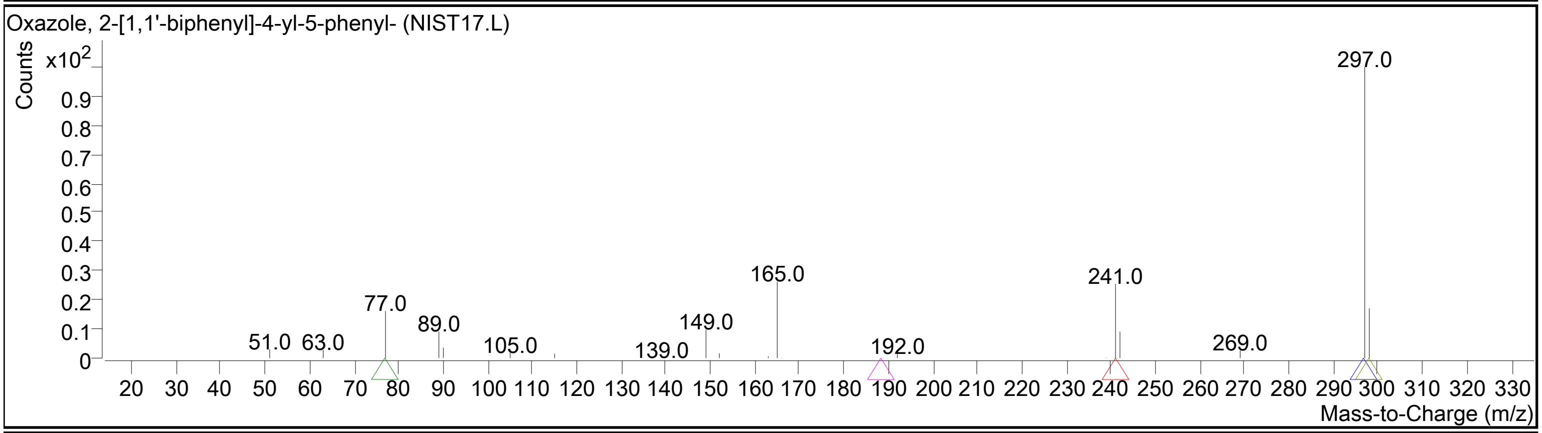

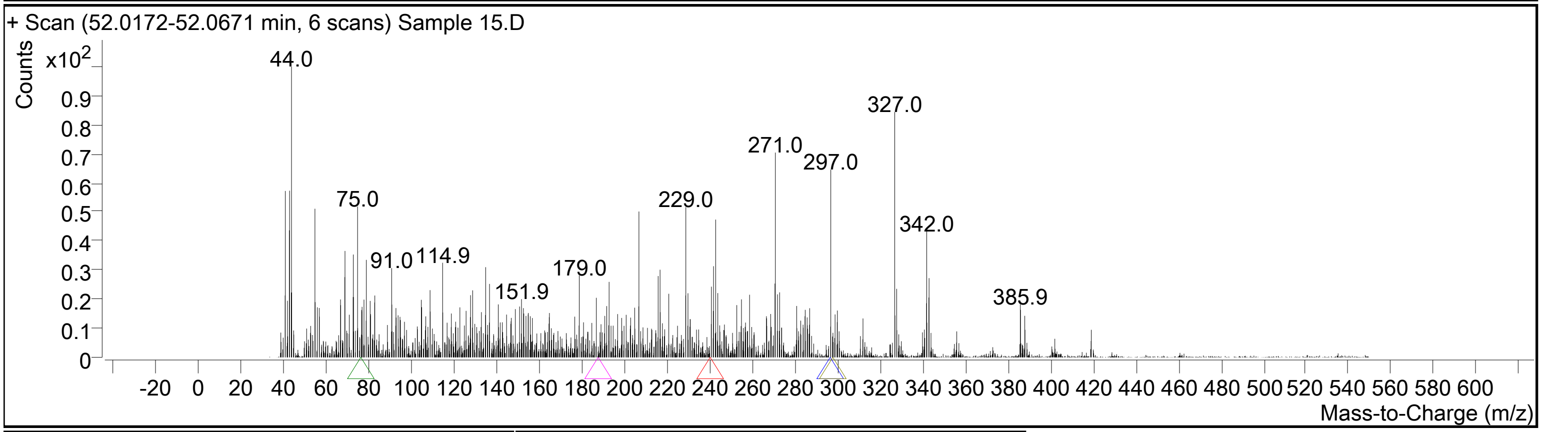

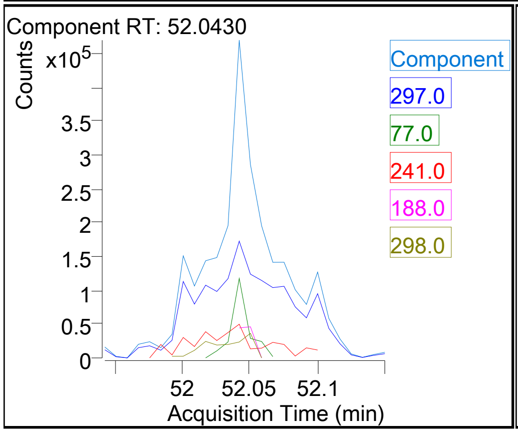

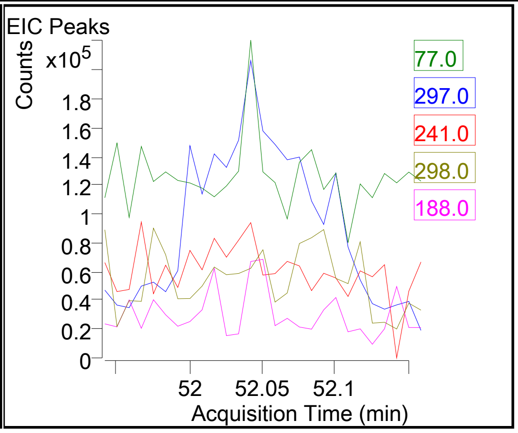

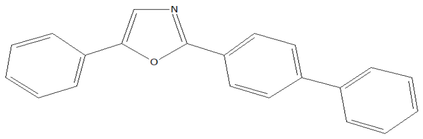

Component RT	Compound Name	Component Area	Match Factor	CAS#	Formula	Estimated Conc.
54.8045	Isophthalic acid, di(2-methylprop-2-en-1-yl) ester	4592828.9	73.8	1000343-95-8	C16H18O4	

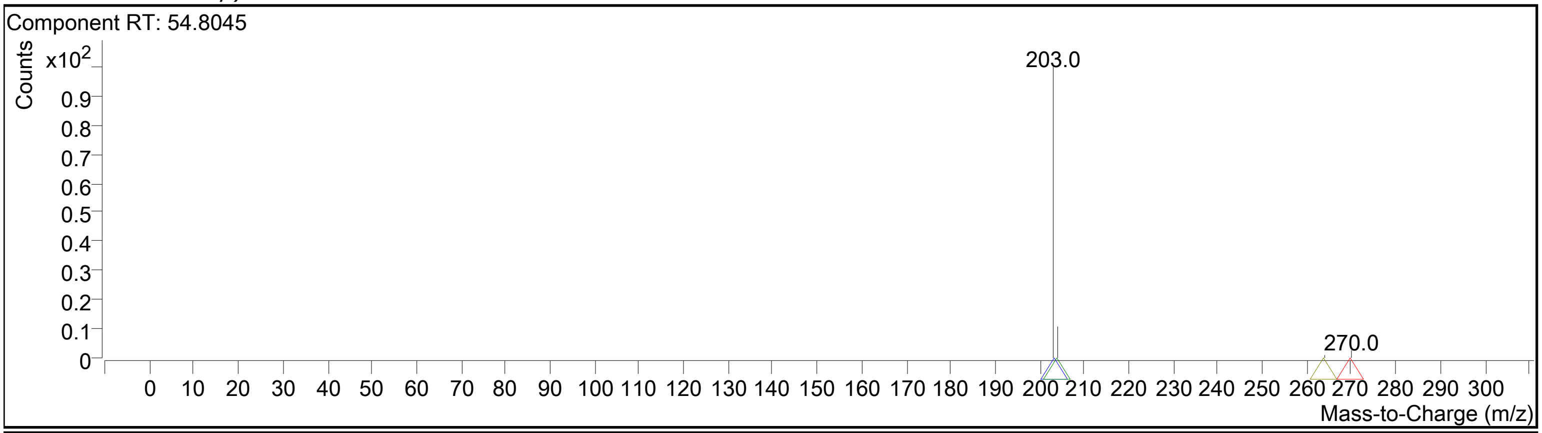

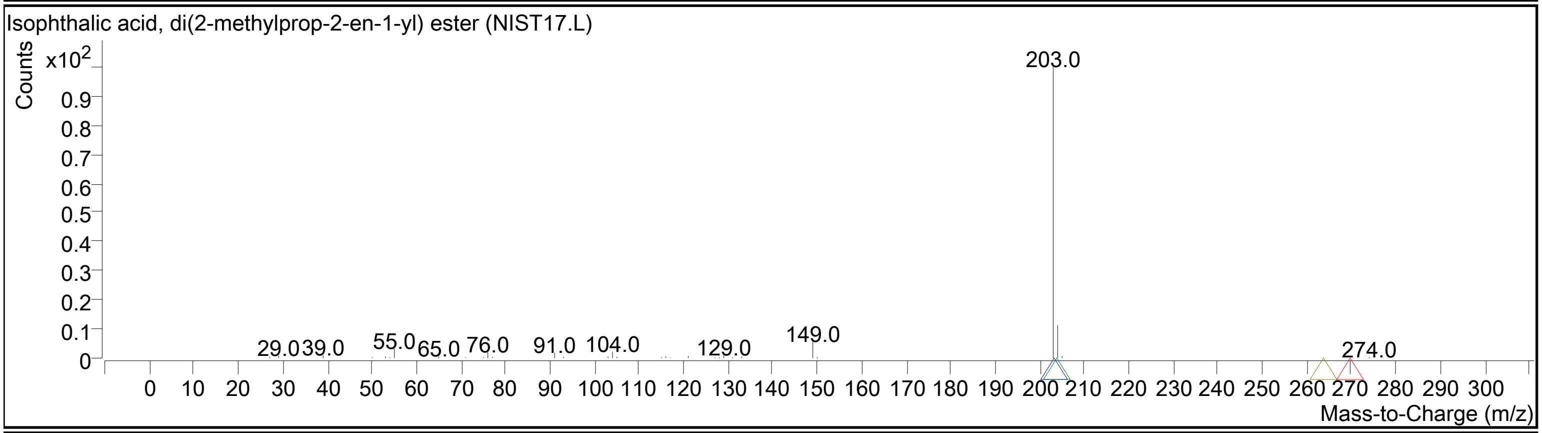

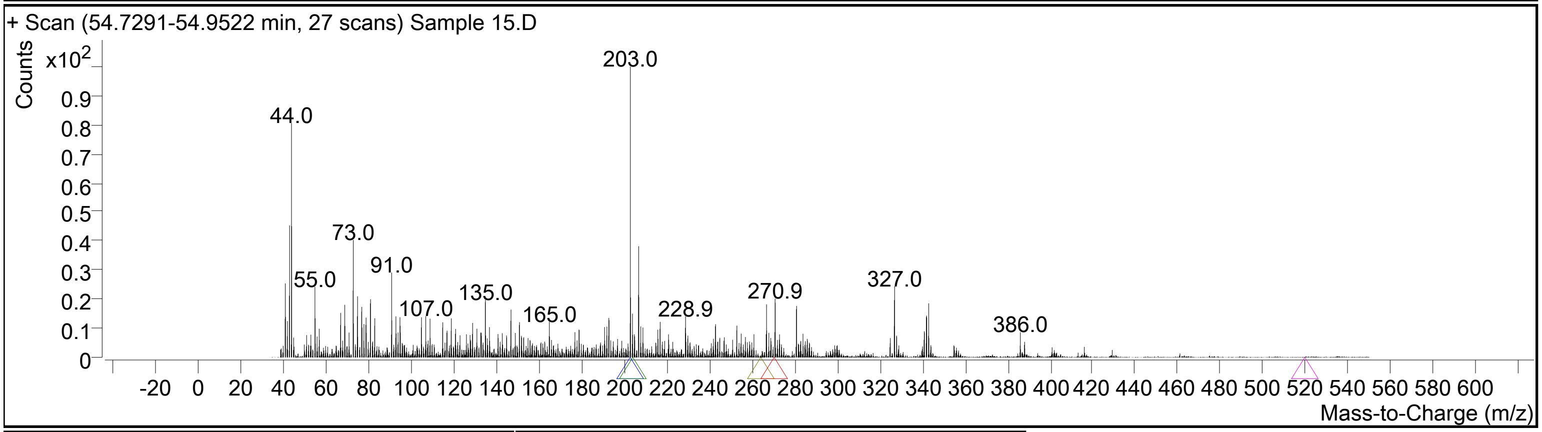

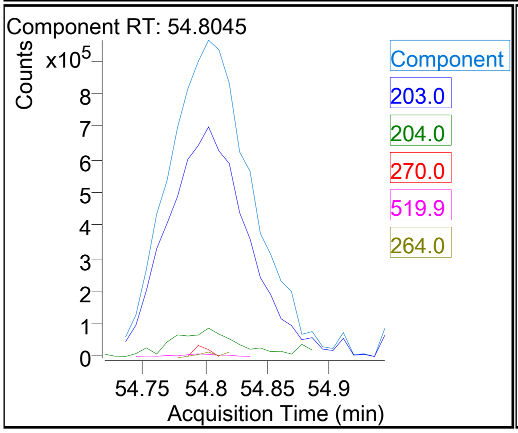

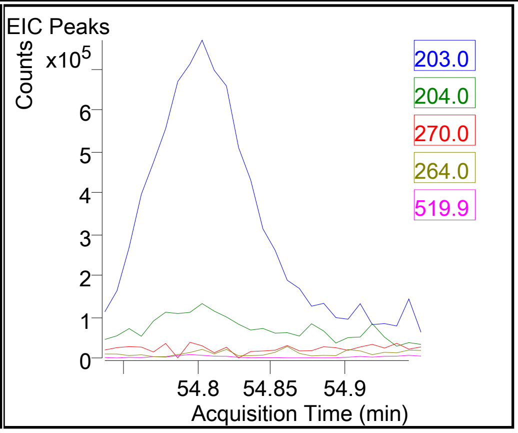

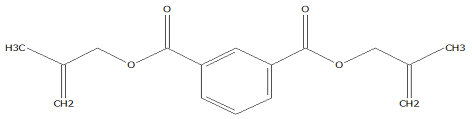

Component RT	Compound Name	Component Area	Match Factor	CAS#	Formula	Estimated Conc.
56.1138	dl-.alpha.-Tocopherol	145133668.8	93.8	10191-41-0	C29H50O2	

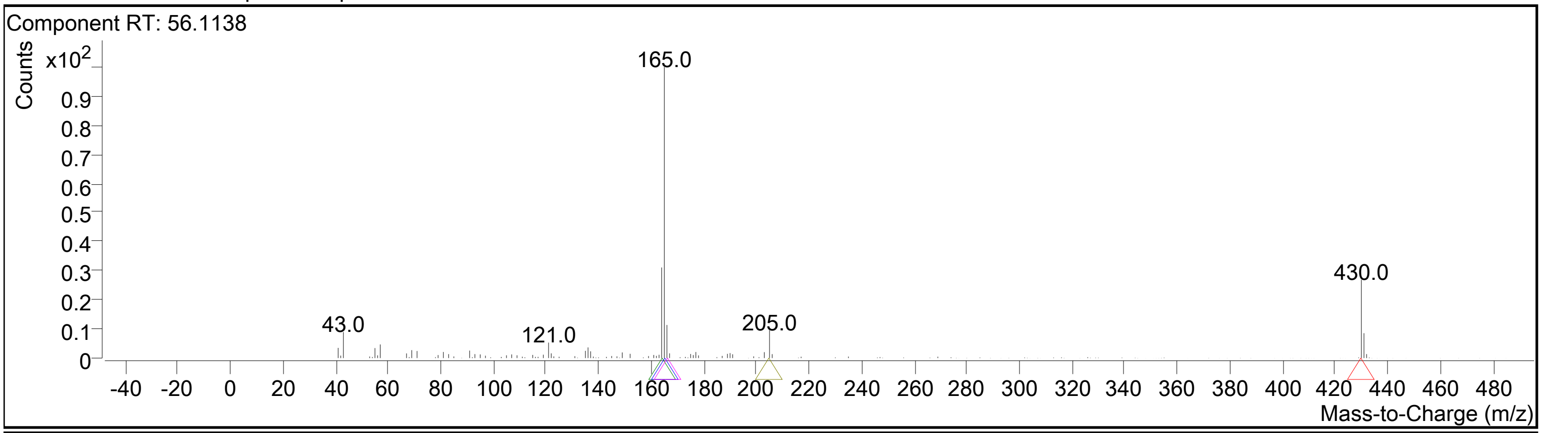

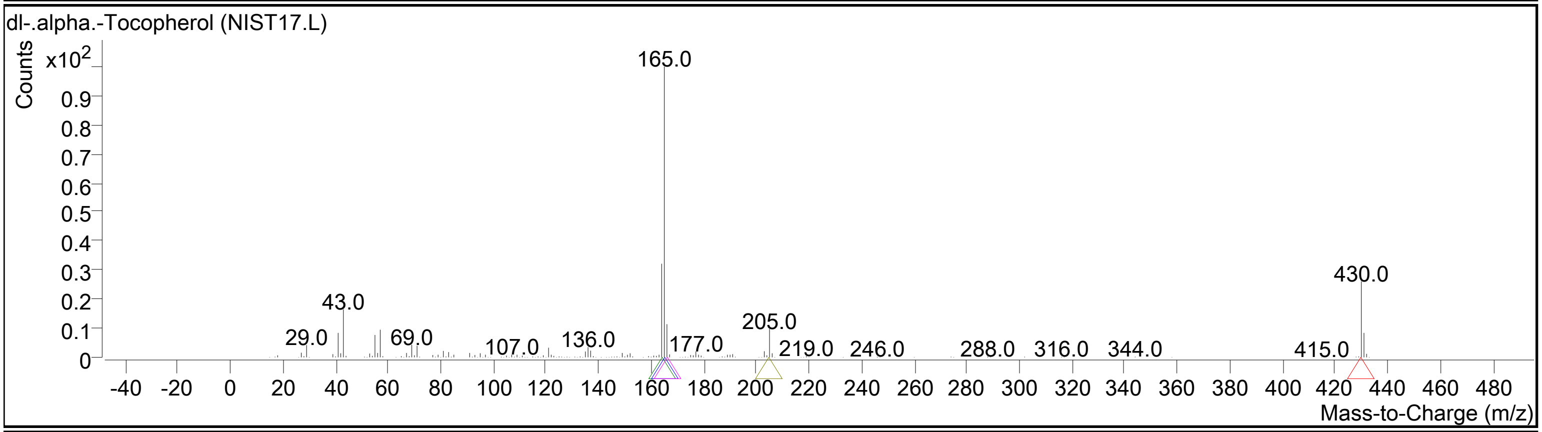

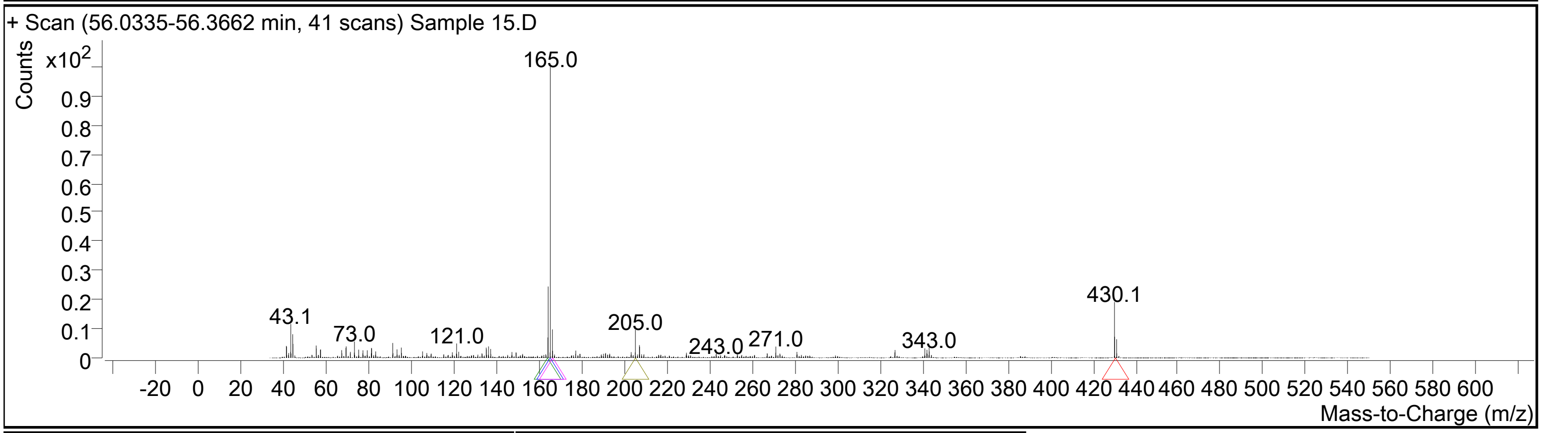

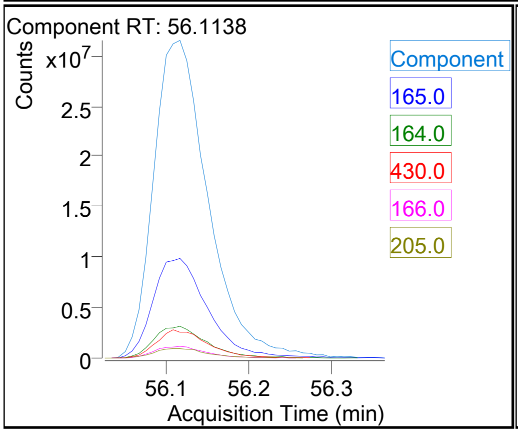

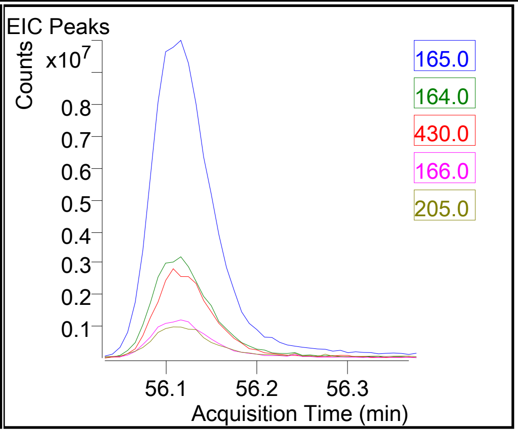

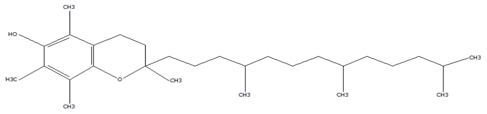

Generated at 12:24 PM on 2/25/2021

Library Search Results - NonTarget Hits with Details

Component RT	Compound Name	Component Area	Match Factor	CAS#	Formula	Estimated Conc.
57.0375	4H-1-Benzopyran-4-one, 5-hydroxy-6,7-dimethoxy-2-(4-methoxyphenyl)-	112768464.1	86.3	19103-54-9	C18H16O6	

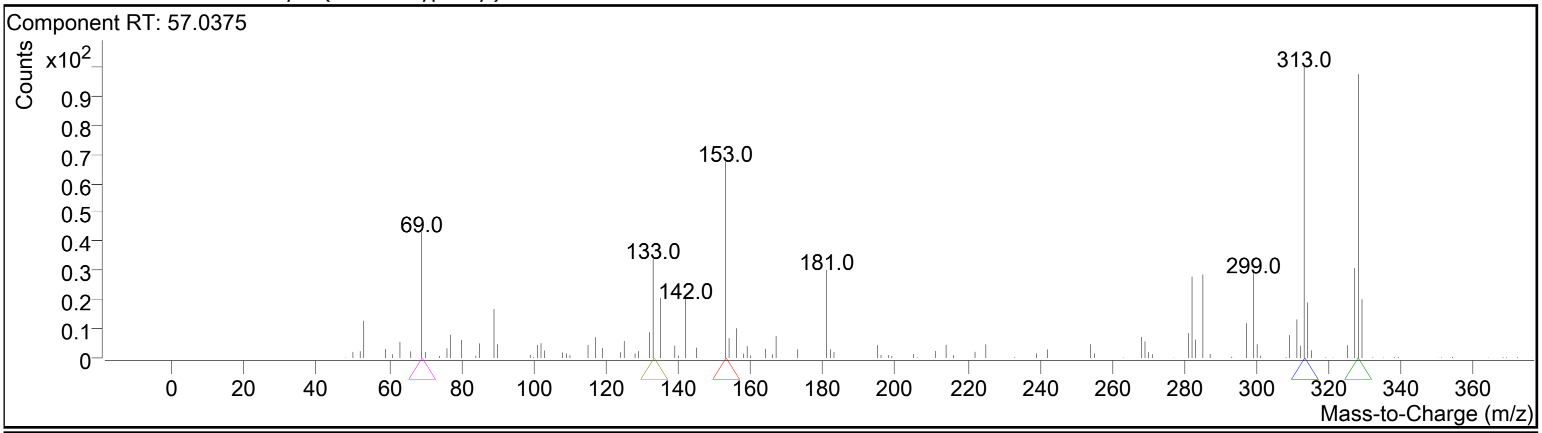

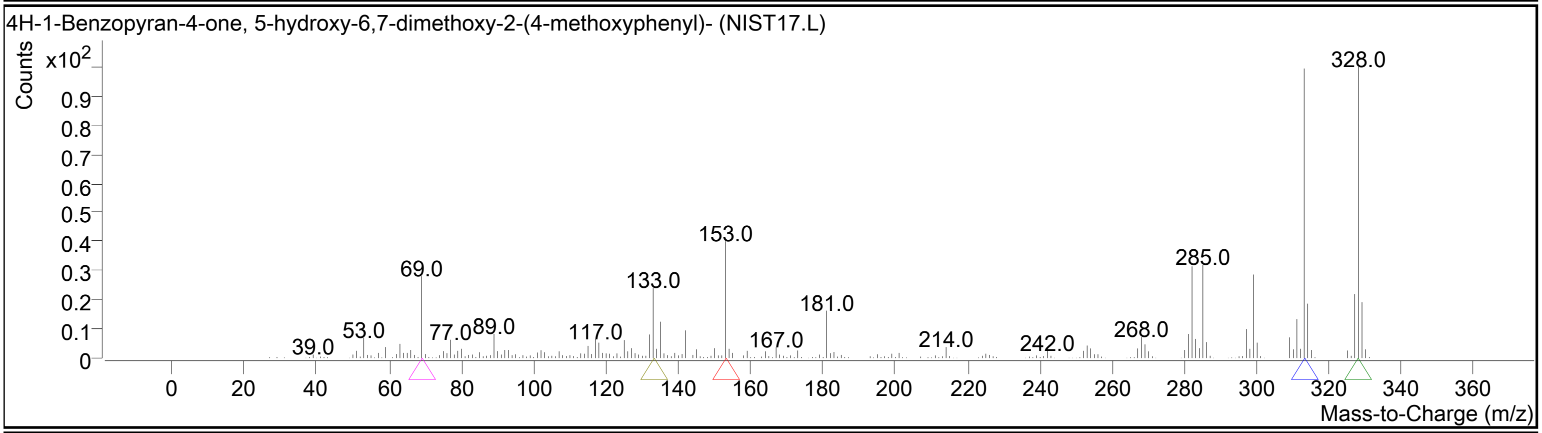

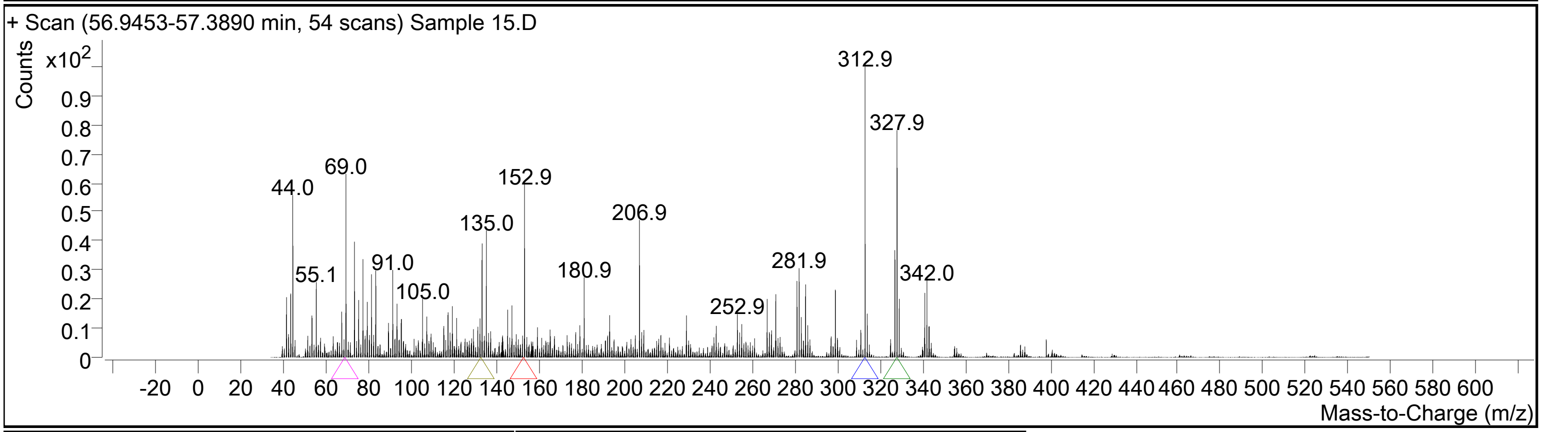

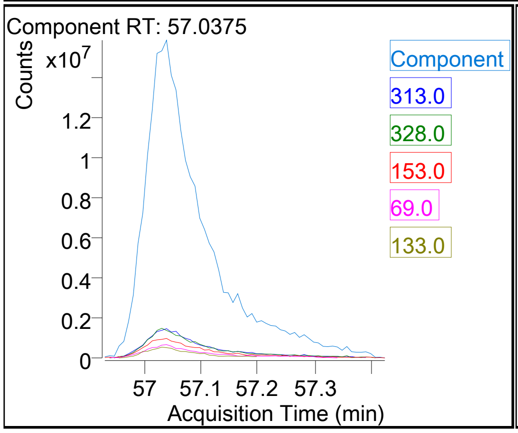

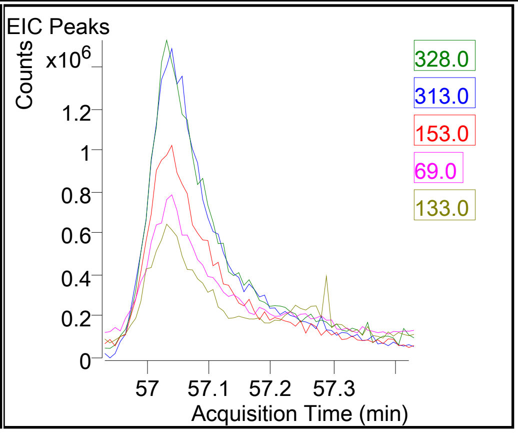

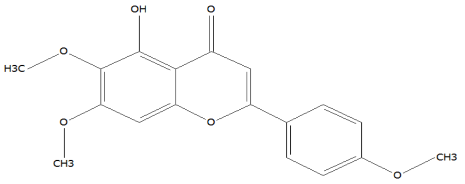

Component RT	Compound Name	Component Area	Match Factor	CAS#	Formula	Estimated Conc.
59.3927	.gamma.-Sitosterol	36034478.5	72.5	83-47-6	C29H50O	

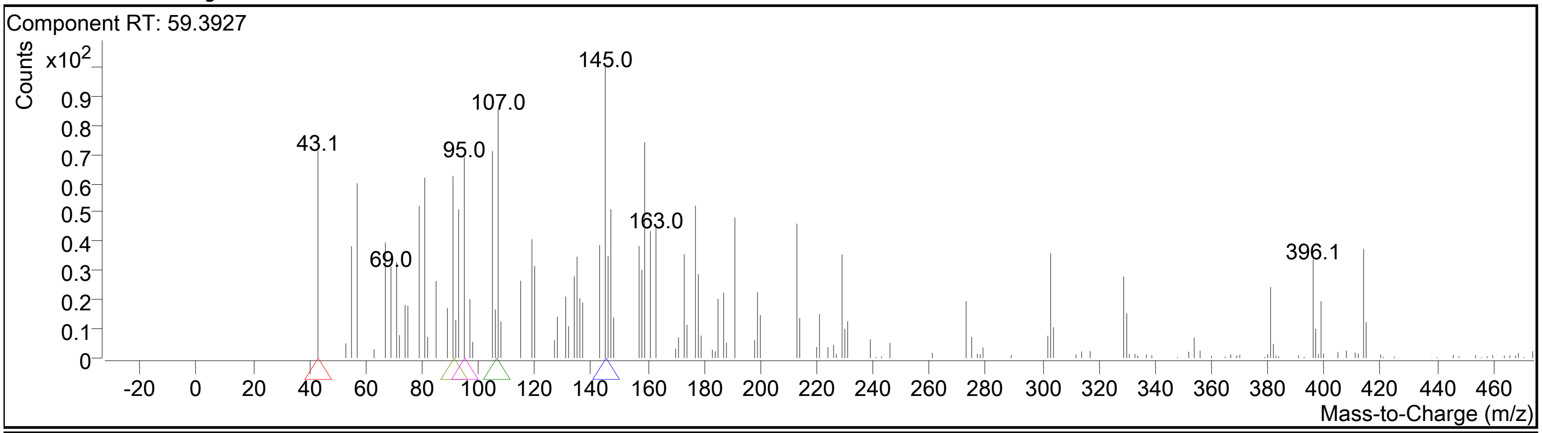

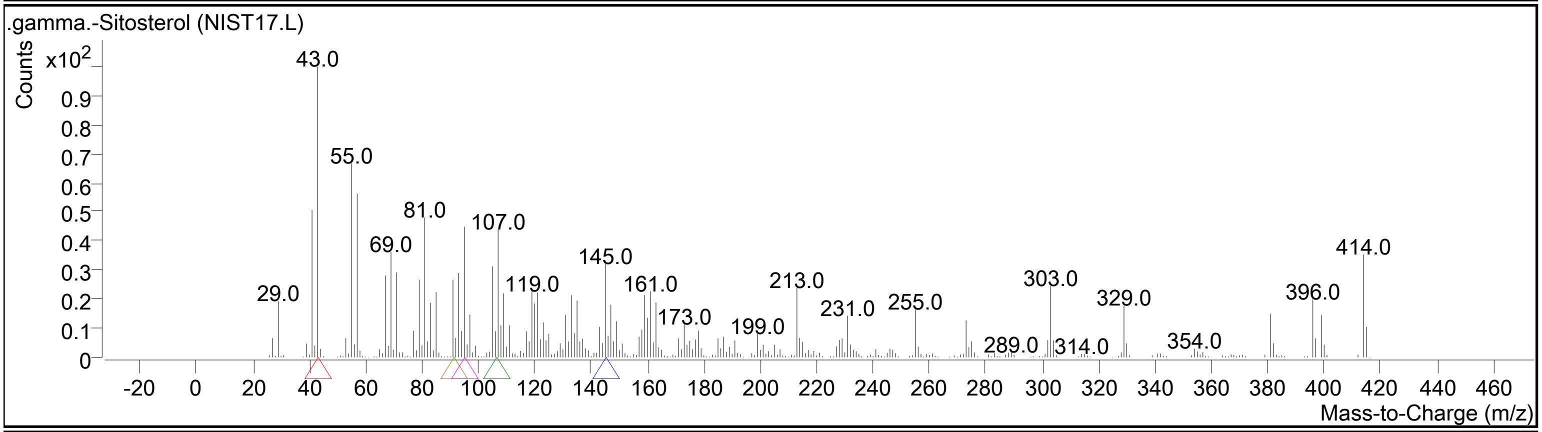

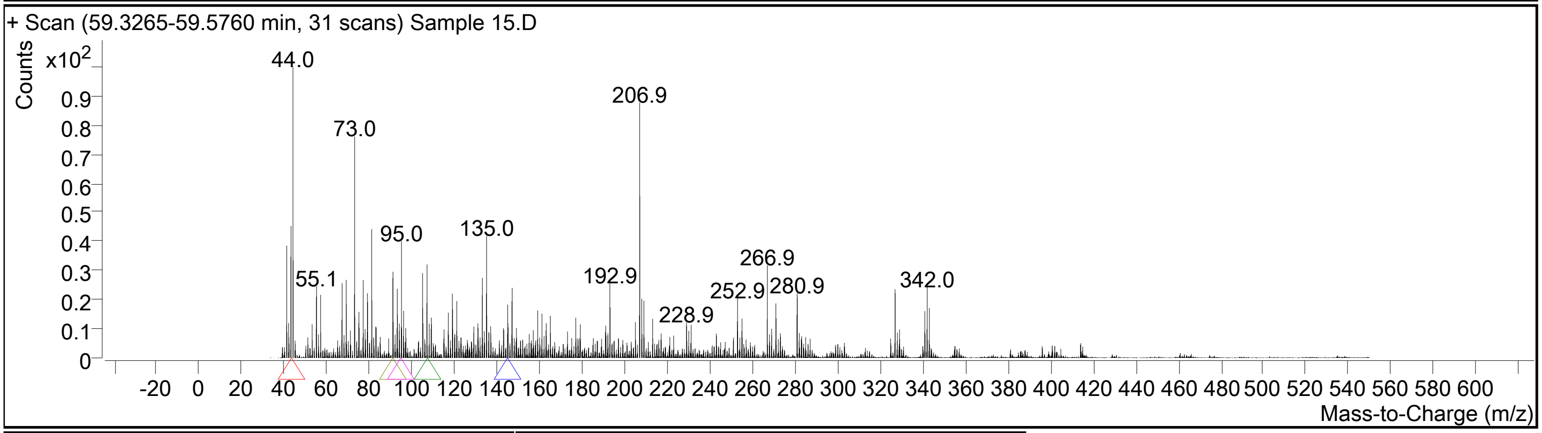

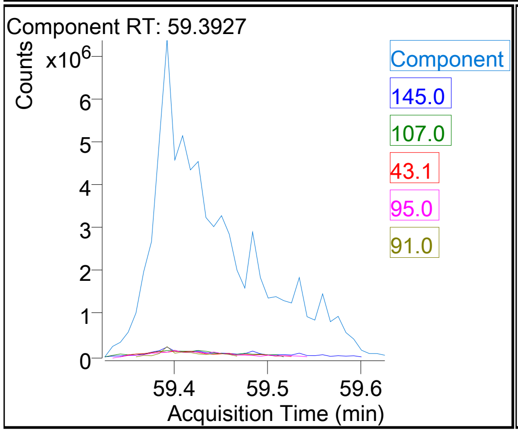

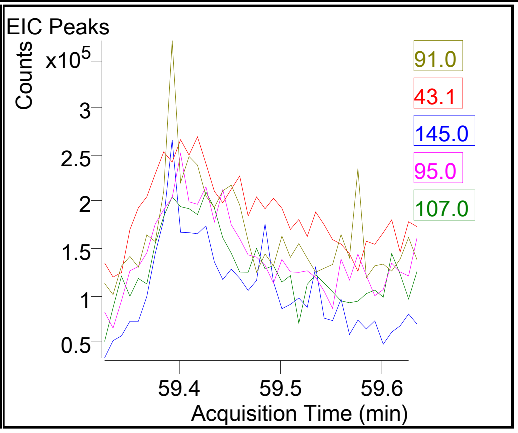

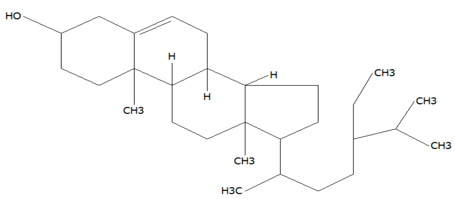

 Generated at 12:24 PM on 2/25/2021

Library Search Results - NonTarget Hits with Details

Component RT	Compound Name	Component Area	Match Factor	CAS#	Formula	Estimated Conc.
61.0418	Lupeol	39047163.6	68.8	545-47-1	C30H50O	

Component RT: 61.0418

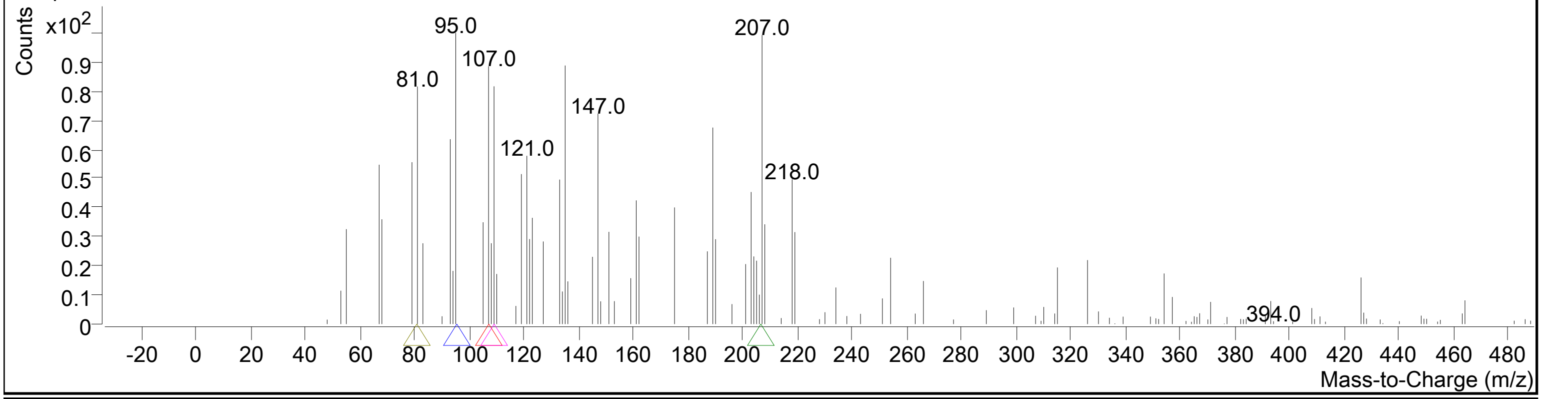

Lupeol (NIST17.L)

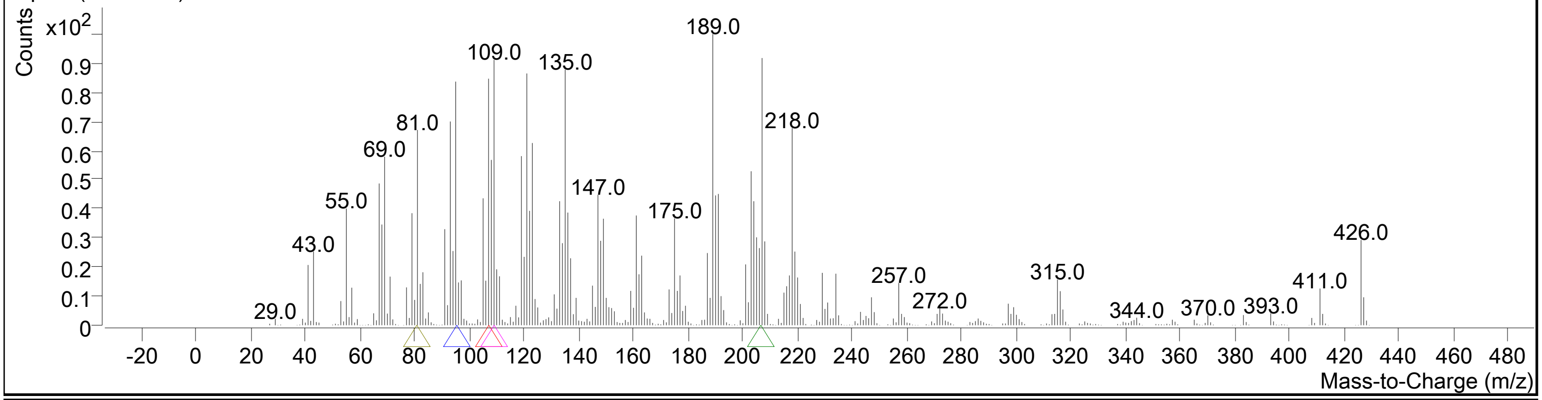

+ Scan (61.0478-61.0645 min, 3 scans) Sample 15.D

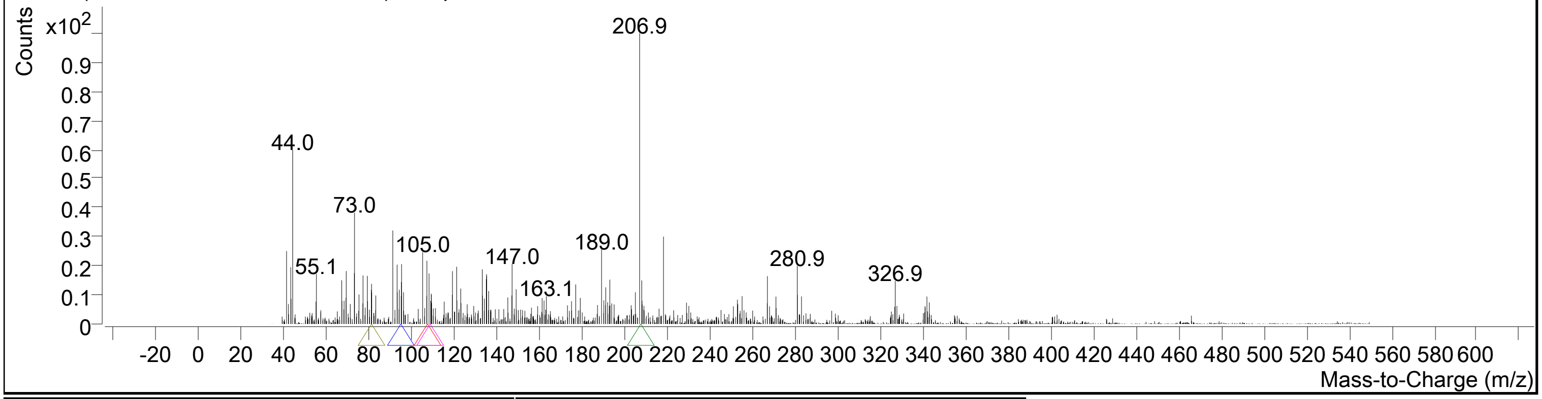

Component RT: 61.0418

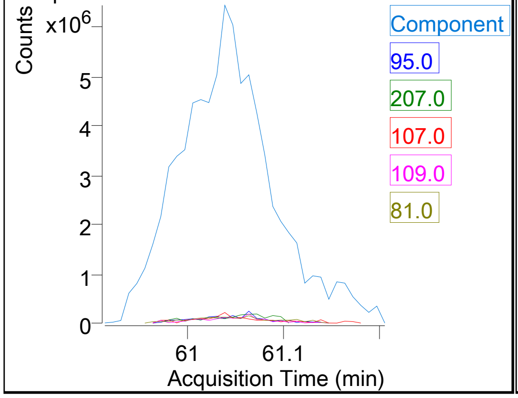

EIC Peaks

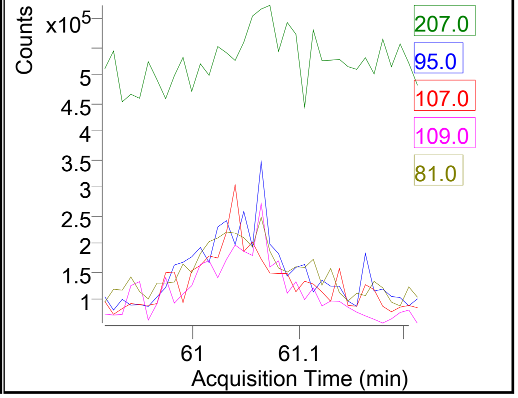

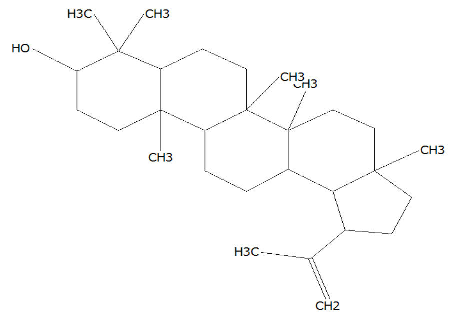

Supplement: Supplemental Information 6 [file peerj-13-18782-s006.pdf]
